# Flash chemistry enables high productivity metalation-substitution of 5-alkyltetrazoles†

**DOI:** 10.1039/d1sc04176b

**Published:** 2021-09-15

**Authors:** Jeff Y. F. Wong, Christopher G. Thomson, Filipe Vilela, Graeme Barker

**Affiliations:** Institute of Chemical Sciences, Heriot-Watt University Riccarton Edinburgh EH14 4AS UK graeme.barker@hw.ac.uk; Continuum Flow Lab, Heriot-Watt University Riccarton Edinburgh EH14 4AS UK

## Abstract

Tetrazoles play a prominent role in medicinal chemistry due to their role as carboxylate bioisosteres but have largely been overlooked as C–H functionalisation substrates. We herein report the development of a high-yielding and general procedure for the heterobenzylic C–H functionalisation of 5-alkyltetrazoles in up to 97% yield under batch conditions using a metalation/electrophilic trapping strategy. Through the use of thermal imaging to identify potentially unsafe exotherms, a continuous flow procedure using a flash chemistry strategy has also been developed, allowing products to be accessed in up to 95% yield. This enabled an extremely high productivity rate of 141 g h^−1^ to be achieved on an entry-level flow system.

## Abstract 2

We report a α-metalation-substitution of readily deprotected 5-alkyltetrazoles under batch and continuous flow conditions. In flow, thermal imaging enabled identification of an unsafe exotherm and optimisation of a productivity rate of 141 g h^−1^.

## Graphical abstract

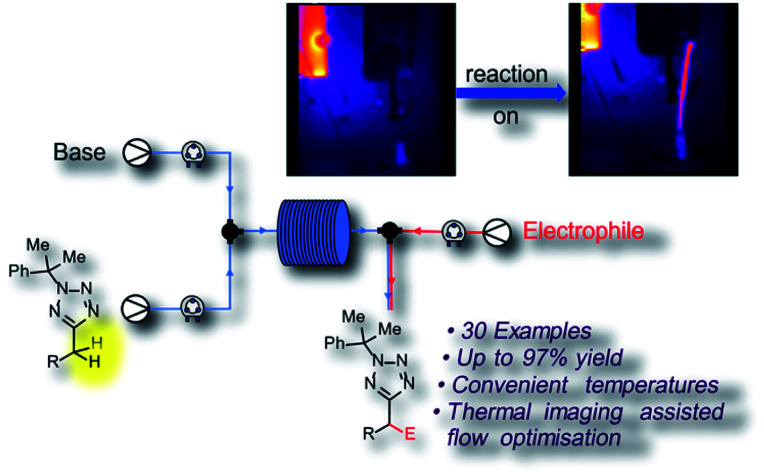

## Introduction

**Fig. 1:**
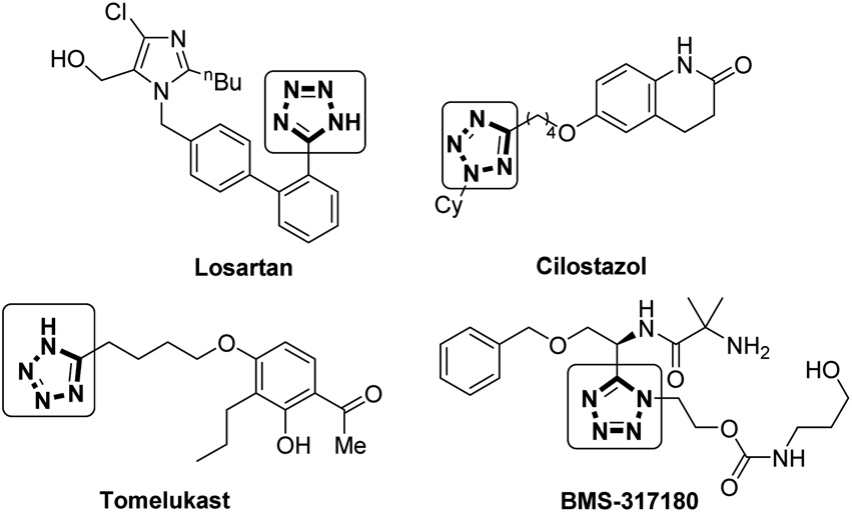
Tetrazole containing drugs.

Tetrazoles are important structural motifs in medicinal chemistry, displaying bioisosterism with carboxylates.^1^ In addition to a similar size, electronic distribution and p*K*_a_,^2^ tetrazoles display increased metabolic stability *vs.* the carboxylate equivalent.^3^ Tetrazole has been shown to be the sixth most common heteroaromatic ring found in drugs listed in the FDA orange book that were commercially available before 2013,^4^ as is evident from their presence in multiple drugs including Losartan (an antihypertensive),^5^ Cilostazol (antithrombotic),^6^ Tomelukast (a leukotriene D4 receptor antagonist),^7^ and BMS-317180 (a growth hormone secretagogue),^8^ shown in Fig. 1.

Despite this privileged position in medicinal chemistry, the α-functionalisation of 5-alkyltetrazoles (analogous to α-functionalisation of carboxylates) remains relatively unexplored.^9^ Previously reported functionalisations of tetrazoles have focused on alkylation and arylation at the *N*1- and *N*2- positions, and require high temperatures and transition metal catalysts.^10–12^ Ugi-azide reactions are often employed to synthesise highly functionalised tetrazoles, typically utilizing toxic and explosive TMSN_3_ as the azide source.^13^

**Scheme 1:**
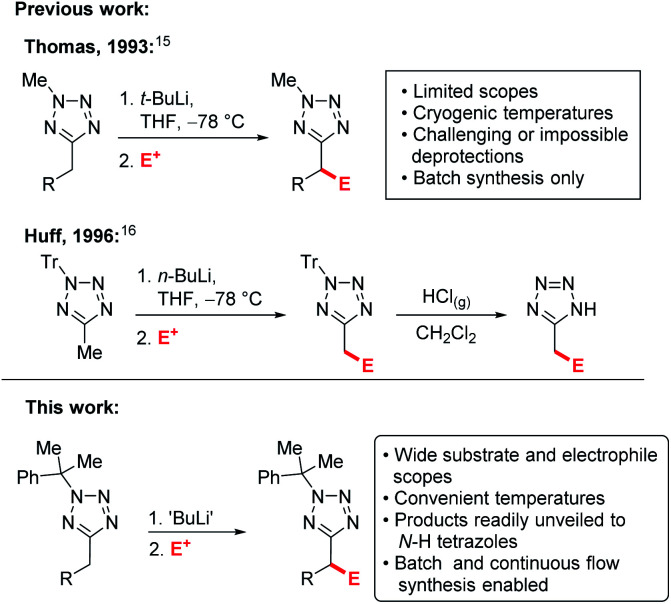
Previous alkyltetrazole lithiations *vs.* this work.

Examples of C–H functionalisation of tetrazole 5-substituents are much rarer.^14^ Flippin has reported *ortho*-lithiation of 5-phenyltetrazoles,^15^ and an early example of lithiation-substitution of *N*-methyl-5-alkyltetrazoles at the α-position was reported by Thomas *et al.*,^16^ however pyrophoric *t*-BuLi and cryogenic temperatures were required and methods for *N*-methyl deprotections have yet to be identified, precluding the products from use as carboxylate bioisosteres. Later, a more convenient lithiation-substitution of trityl protected tetrazoles using *n*-BuLi was reported by Huff *et al.* (Scheme 1).^17^ However, in this case deprotection was challenging, requiring the use of gaseous HCl. In both cases, the substrate and electrophile scopes were extremely limited and industrially inconvenient cryogenic (−78 °C) reaction temperatures are required. C–H functionalisations of tetrazole derivatives under continuous flow conditions have yet to be reported.

Recently, our group has developed a lithiation-substitution protocol for 5-benzyltetrazoles using *n*-BuLi at an industrially convenient temperature.^18^ Unfortunately, 5-alkyltetrazoles proved not to be amenable to derivatisation using this approach, considerably limiting the substrate scope, and continuous flow conditions could not be optimised due to precipitation of an insoluble metalated intermediate, and the metalation-substitution of unprotected or readily deprotected 5-alkyltetrazoles in the α-position has not previously been described. Herein, we report a general and high-yielding strategy for the C–H functionalisation of 5-alkyltetrazoles under both batch and continuous flow conditions.

## Results and discussion

To begin our study, we investigated convenient protecting groups for lithiation-substitution of 5-alkyltetrazoles (Table 1). *N*-Acyl-5-alkyltetrazoles readily undergo rearrangement to form the corresponding 1,3,4-oxadiazoles,^19–22^ so the pivaloyl protecting group was discounted. We have found *N*-Boc tetrazoles challenging to purify and store without slow decomposition, so instead we started with *N*-trityl-5-propyltetrazole **1**, similar to protection employed by Huff *et al.* for the lithiation-substitution of 5-methyltetrazole.^17^ Treatment of **1** with *n*-BuLi at 0 °C for 1 hour followed by addition of acetone as an electrophile gave full recovery of starting material with no evidence for the formation of trapping product **2** (entry 1). Switching base to more reactive *s*-BuLi at 0 °C gave full consumption of starting material, however no identifiable species were observed by ^1^H NMR spectroscopy of the crude reaction mixture, suggesting the lithiated species might be unstable at 0 °C (entry 2). Repeating the lithiation at −78 °C gave trapping product **2** in excellent (84%) yield after trapping with acetone (entry 3).

**Table 1:** Optimisation of the lithiation-substitution of *N*-protected alkyltetrazoles

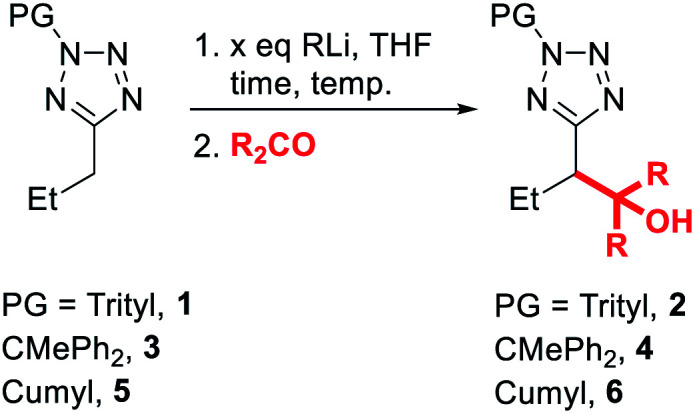							
Entry	PG	*x*	RLi	Time	Temp.	R	Yield^a^
1	Trityl	1.5	*n*-BuLi	1 h	0 °C	Me	No reaction
2	Trityl	1.5	*s*-BuLi	1 h	0 °C	Me	Complex mixture
3	Trityl	1.6	*s*-BuLi	30 min	−78 °C	Me	84%
4	CMePh_2_	2.3	*n*-BuLi	15 min	0 °C	Me	Complex mixture
5	CMePh_2_	2.3	*n*-BuLi	15 min	−78 °C	Me	No reaction
6	CMePh_2_	2.3	*s*-BuLi	15 min	−78 °C	Me	79%
7	Cumyl	1.5	*n*-BuLi	1 h	0 °C	Me	63%
8	Cumyl	2.1	*n*-BuLi	1 h	0 °C	Me	60%
9	Cumyl	2.1	*n*-BuLi	1 h	−30 °C	Me	76%
10	Cumyl	2.1	*n*-BuLi	1 h	−78 °C	Me	No reaction
**11**	**Cumyl**	**1.6**	***s*-BuLi**	**15 min**	**−78 °C**	**Me**	**97%**
12	Cumyl	1.5	*n*-BuLi	15 min	0 °C	Ph	68%
**13**	**Cumyl**	**2.3**	***n*-BuLi**	**15 min**	**0 °C**	**Ph**	**85%**
14	Cumyl	1.5	LDA	15 min	0 °C	Me	No reaction
15	Cumyl	1.5	LDA	15 min	−78 °C	Me	No reaction

a Isolated yield.

Since we aimed to develop reaction conditions without the need for cryogenic temperatures, we continued to screen other protecting groups. The use of a diphenylethyl protecting group led to no significant changes in results, with efficient lithiation-trapping of **3** observed only with *s*-BuLi at −78 °C (entries 4, 5 and 6). To our delight, changing the protecting group to cumyl, which has previously been used by Flippin *et al.* for the nucleophilic aromatic substitution of 5-aryltetrazoles,^23^ dramatically changed the reactivity. The cumyl group is readily installed in high yield using α-methylstyrene and TFA (see ESI† for full details). We note that α-methylstyrene is a cost efficient source of this protecting group (£53.60/2.5 L, Sigma–Aldrich, 2021) and that Flippin has outlined two complementary procedures for high-yielding deprotections of *N*-cumyl tetrazoles.^23^ A modest yield of **6** was obtained when **5** was treated with *n*-BuLi at 0 °C followed by electrophilic trapping with acetone (entry 7). Neither increasing the equivalents of base (entry 8) nor lowering the temperature to −30 °C (entry 9) gave significant improvement to the yield of product **6**. Once again, no lithiation was observed using *n*-BuLi at −78 °C (entry 10), whilst switching the base to *s*-BuLi gave excellent yield (97%) of product **6** (entry 11). We speculated that the decrease in yield of **6** when the lithiation was carried out at 0 °C was due to the lithiated **5** acting as a base to deprotonate acetone instead of reacting as a nucleophile. Thus, the lithiation-trapping of **5** was attempted with benzophenone as the electrophile. Treatment of **5** with 1.5 equivalents of *n*-BuLi at 0 °C gave product **6** in modest (68%) yield after trapping with benzophenone (entry 12). Increasing the loading of *n*-BuLi to 2.3 equivalents gave **6** in an excellent 85% yield (entry 13). We note that this requirement for an extra equivalent of base over the strict stoichiometric requirements during lithiation of tetrazoles appears to be a common issue, and has been encountered by our group during the lithiation of benzyltetrazoles, as well as Flippin during the *ortho*-lithiation of 5-aryltetrazoles.^15,18^ Since no lithiation was observed when milder LDA was used as base (entries 14 and 15), the conditions in entries 11 and 13 were taken as optimal. It is hypothesised that the drop in yield at 0 °C when lengthening reaction time from 15 min to 1 h (entry 13 *vs.* 8) may arise from instability of the lithiated intermediate.

**Scheme 2:**
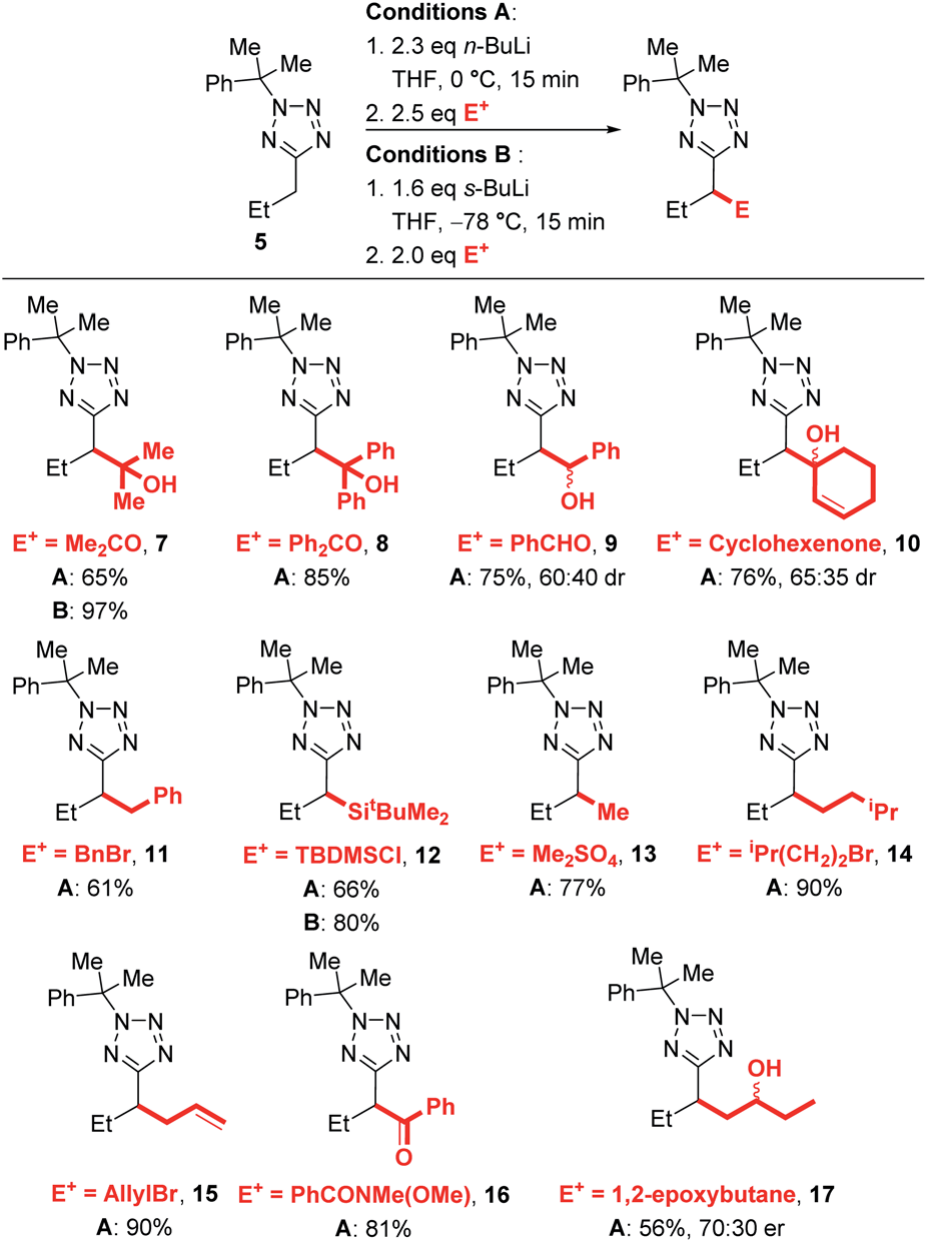
Electrophile scope.

We next explored the electrophile scope under our optimised conditions (Scheme 2). We were pleased to find that almost all electrophiles were compatible with lithiation at 0 °C (conditions A). Alcohol **9** (75%) and **10** (76%) were isolated in good yield and moderate diastereoselectivities after trapping with benzaldehyde and cyclohexanone respectively. A slight drop in yield was observed with benzylation with BnBr (**11**, 61%) and silylation with TBDMSCl (**12**, 66%) with conditions A, however the yields of **12** could be improved to 80% by employing low temperature lithiation conditions with *s*-BuLi (conditions B). TBDMSCl is generally regarded as a sow-trapping electrophile, and this may allow time for partial decomposition of lithiated **5** at 0 °C, accounting for the lower yield under conditions A. Alkylation could be achieved in good yield with Me_2_SO_4_ (**13**, 77%), iso-pentyl bromide (**14**, 90%) and an epoxide (**17**, 56%, 70 : 30 dr) with conditions A. Finally, allylation and acylation could be achieved in excellent yields with allyl bromide (**15**, 90%) and a Weinreb amide (**16**, 81%). Unfortunately, attempted trapping with iodine resulted in no desired product.

**Scheme 3:**
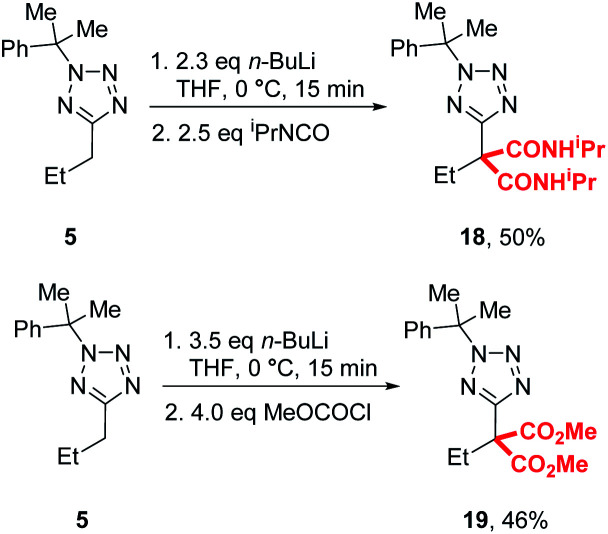
Di-substitution with chloroformates and isocyanates.

During our previously reported lithiation-substitution of alkyl-1,3,4-oxadiazoles, we observed that di-substituted products may be obtained when trapping with chloroformates, a common phenomenon with this class of electrophile in lithiation chemistry.^24,25^ We found that di-carbamoylation of **5** to give **18** could be achieved in modest (50%) yield using our optimised conditions A and trapping with an iso-cyanate, while diester **19** could be isolated in 46% yield using methyl chloroformate by increasing the base and electrophile to 3.5 and 4.0 equivalents, respectively (Scheme 3).

**Scheme 4:**
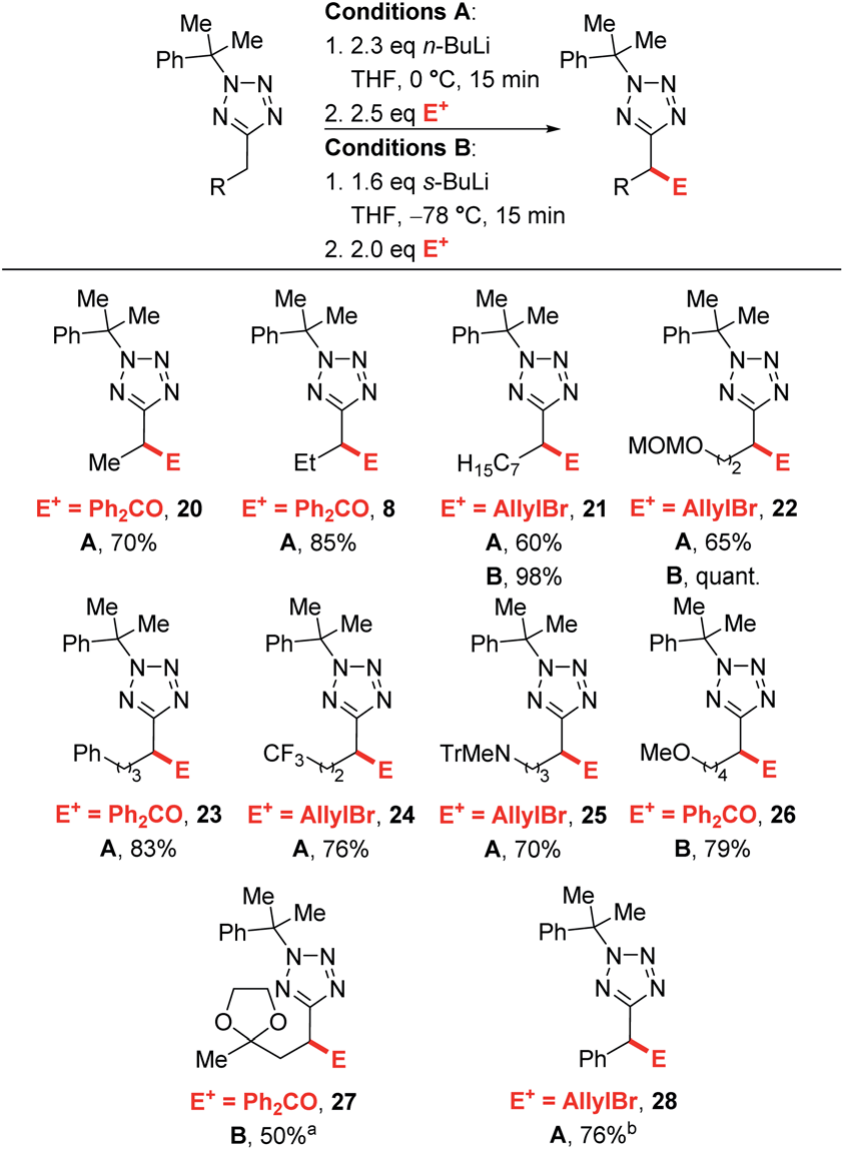
Substrate scope. ^*a*^Using 3.2 eq. *s*-BuLi.LiCl. ^*b*^Using 1.5 eq. *n*-BuLi.

Next, we turned our attention to exploring the substrate scope (Scheme 4). Efficient lithiation-substitution was observed with shortening the chain to an ethyl group (**20**, 70%). **21**, bearing a longer alkyl chain, and MOM-protected alcohol **22** were isolated in moderate yield under conditions A (60% and 65%) after trapping with allyl bromide; yields were improved to 98% and quantitative respectively using conditions B. A phenyl group is also well tolerated, with the lithiation-substitution to form **23** in 83% after trapping with benzophenone at 0 °C. Trifluoromethyl (**24**, 76%) and *N*-trityl amino (**25**, 70%) motifs were also well tolerated under conditions A. In the presence of a methoxy group, an inseparable mixture of desired product **26** and cumyl ring lithiation-substitution product was observed when conditions A were employed. Competing ring lithiation could be avoided under conditions B and **26** was isolated in 79% yield. Acetal-containing product **27** was obtained in 50% yield under conditions B. Our previously reported C–H functionalisations of benzyltetrazoles were not amenable to continuous flow chemistry, so for a direct comparison with an attempted flow experiment, a benzyl substrate was reacted under modified conditions A to give **28** in 76% yield – in this case, reduced base was required to avoid disubstitution (see ESI†).

**Scheme 5:**
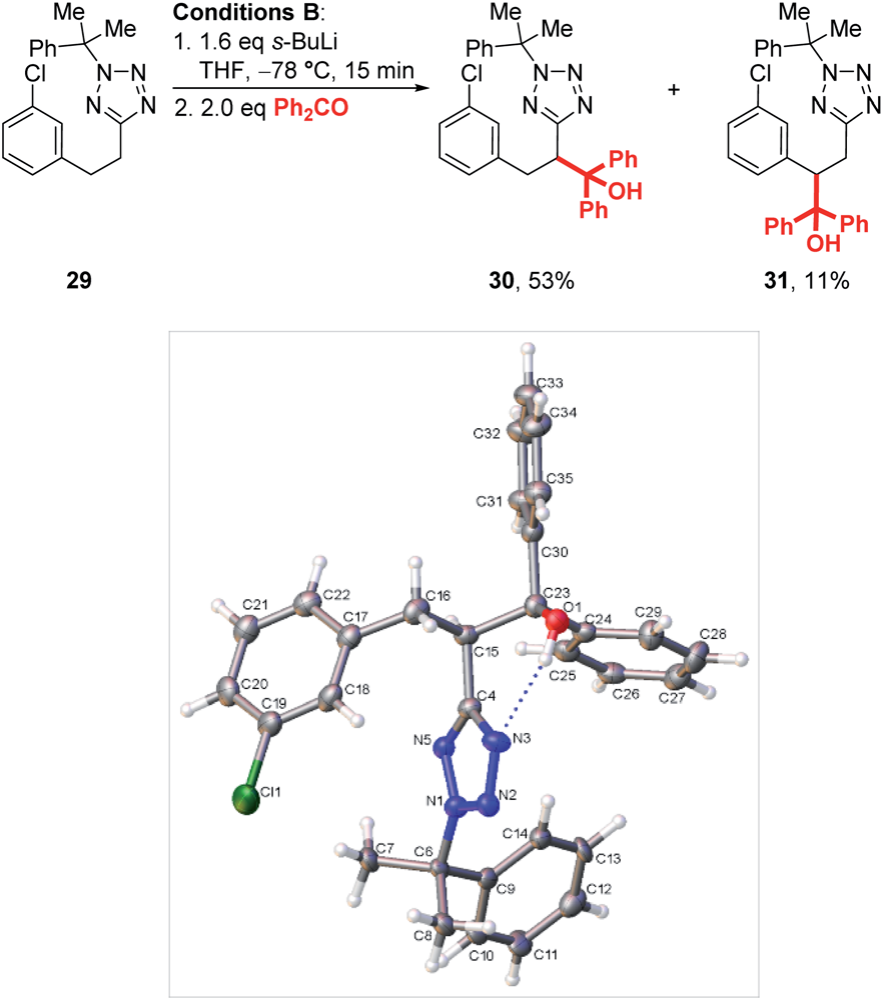
Lithiation-substitution of chlorophenyl substrate **29** and crystal structure of **30**.

Interestingly, when chlorophenyl derivatives were subjected to our standard lithiation conditions, a mixture of heterobenzylic and benzylic trapping products **30** and **31** were obtained, a result which was not observed when reacting purely phenyl substrates (see **23**, Scheme 4). Fortunately, expected product **30** was isolated in 53% *vs.* 11% for **31**. The structure of each product was confirmed by NOESY NMR (see ESI†) and an X-ray crystal structure of **30** (Scheme 5).

**Scheme 6:**
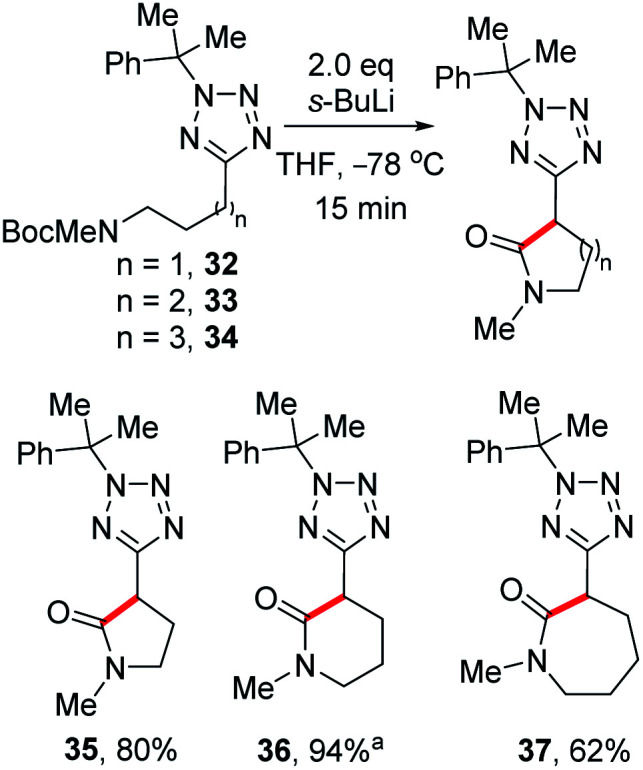
Synthesis of tetrazolyl lactams.^*a*^With 3.2 eq. *s*-BuLi.

While investigating the lithiation-substitution of substrates bearing protected amines, we found that *N*-Boc substrates **32–34** underwent internal electrophilic trapping to give tetrazolyl lactams. Thus, we were able to optimise this synthesis by omitting an external electrophile, and 5-, 6-, and 7-member lactams **35**, **36** and **37** were obtained in 80%, 94% and 62% yield respectively (Scheme 6).^26^

**Scheme 7:**
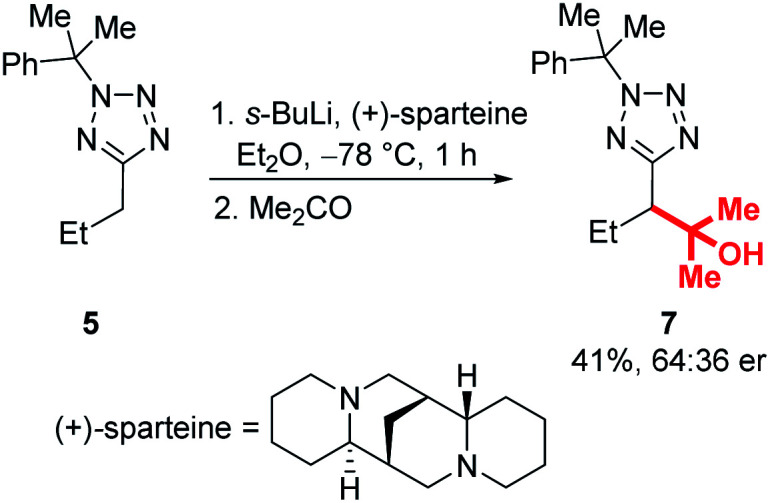
Attempted enantioselective lithiation-substitution.

Enantioselective lithiation-substitution protocols mediated by the chiral diamine sparteine are well established for a range of substrate classes,^27–29^ and we next attempted stereoselective functionalisations of our tetrazoles under the control of a (+)-sparteine/*s*-BuLi complex in Et_2_O at −78 °C before electrophilic trapping with acetone (Scheme 7). Unfortunately, product **7** was obtained in only 41% yield and 64:36 er. Investigation of a range of other chiral ligands known to promote enantioselective lithiation substitutions, including a *trans*-cyclohexane-1,2-diamine pioneered by Alexakis and dipeptides used by Beak and Coldham similarly failed to provide products in satisfactory enantioenrichments.^29–44^ We hypothesised that this may be due to configurational instability of the lithiated tetrazole intermediate – a Hoffman test using racemic and (*S*)-2-(dibenzylamino)-1,3-diphenylpropan-1-one as electrophiles confirmed that this is the case (for full details of ligand screen and Hoffman test, see ESI†).^45^

With a high-yielding batch procedure in hand, we next turned our attention to optimising a continuous flow procedure. Flow chemistry offers significant safety advantages as well as superior control of reaction mixing and thermal transfer,^46,47^ and as such the use of organolithiums under continuous flow conditions as been well reviewed in recent years.^48–52^ Flash chemistry, developed by Yoshida and co-workers,^53–58^ refers to synthesis in which “extremely fast reactions are conducted in a highly controlled manner to produce desired compounds with high selectivity”;^54^ in addition to intrinsically high productivity rates, impressive transformations *via* unstable intermediates (*e.g.* organolithiums bearing highly reactive electrophilic functional groups) may be achieved.^51,59–79^ Despite these advantages, generation of organolithium species in flow is dominated by halogen-lithium exchanges, with C–H metalations (and particularly Csp^3^–H metalations) comparatively underexplored.^24,80–86^

Recently, we reported a continuous flow lithiation-substitutions of alkyl-1,3,4-oxadiazoles with a 1.3 s reactor residence time for lithiation before in-flow electrophilic trapping.^24^ Quantitative yields were obtained at rt which were not replicable under comparable conditions in a batch reactor, and we were therefore inspired to investigate C–H functionalisations of 5-alkyltetrazoles in flow. We began by attempting to replicate our batch synthesis conditions (modified for room temperature) under continuous flow (Table 2).

**Table 2:** Initial flow condition screen

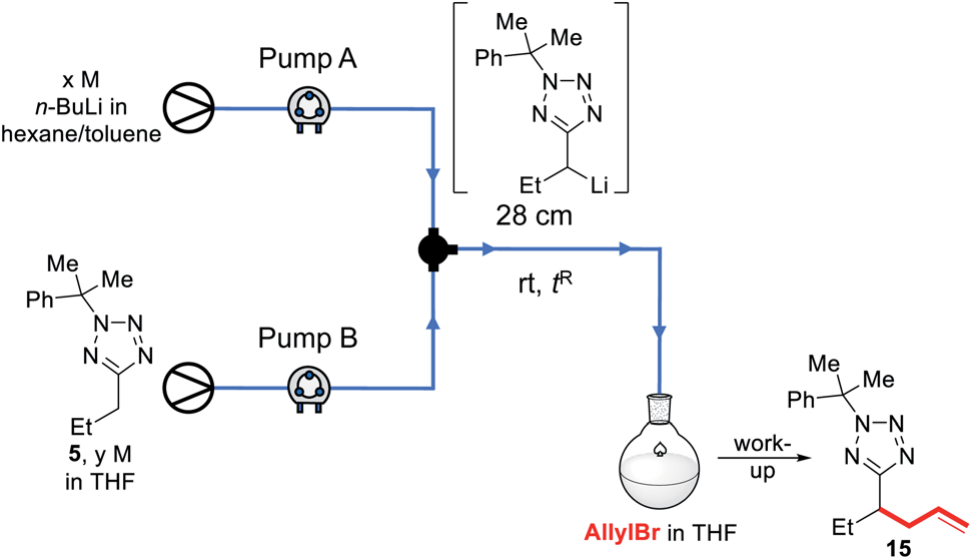						
Entry	*X* (M)	*Y* (M)	Flow rate^a^ (mL min^−1^)	Residence time (s)	Yield^b^	SM^b^^,^^c^
1	2.5	1.1	5	2.6	35%	50%
2	2.5	0.55	5	2.6	Trace	35%
3	1.75	0.76	5	2.6	48%	48%
4	1.25	0.54	5	2.6	12%	75%
5	0.75	0.33	5	2.6	12%	71%
6	0.75	0.33	1	13	12%	56%
7	0.75	0.33	1	10 min	14%	57%

a Flow rate from each pump; after junction, flow rate is doubled.

b As determined by ^1^H NMR spectroscopy in the presence of 1,3,5-trimethoxybenzene.

c Remaining starting material.

First, a 2.5 M solution of *n*-BuLi in hexanes/toluene and a 1.1 M solution of **5** in THF were reacted in flow in a 28 cm length of tubing at rt for 2.6 s before being pumped into a stirred solution of allyl bromide in THF at rt; after work up, a 35% conversion to **15** was observed by ^1^H NMR of the crude reaction mixture (entry 1). Decreasing the concentration of the starting material feedstock solution to 0.55 M resulted in a loss of conversion (entry 2). A 48% conversion was achieved with a 1.75 M concentration of *n*-BuLi, and 0.76 M of **5** (entry 3). Unfortunately, further dilution of the base and starting material solutions, as well as a lower flow rate led to a decrease in yield (entries 4–7). Before further optimisation could be conducted, however, we observed that under the conditions shown in entries 1 and 3, the solvent appeared to be boiling in the reactor, with bubbles seen to be far in excess of that which could be accounted for by evolution of *n*-butane. Alarmed by the safety implications of an exothermic reaction resulting in the boil-off of an ethereal solvent, we resolved to investigate further.

**Fig. 2:**
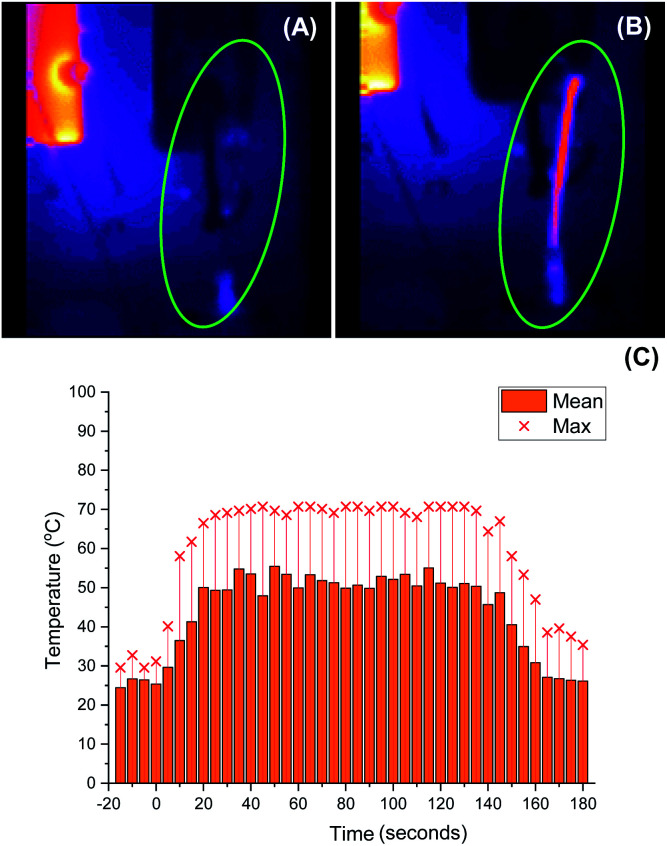
Thermal imaging of flow metalation in THF with the reactor tubing circled in green. (A) While pumping solvent at rt. (B) While reaction is running. (C) Graph of mean and maximum reactor temperatures over time. Only solvent at rt is pumping until 0 s, at which base and starting material solutions (both at rt) are pumped to the reactor. Pumping is switched back to solvent only at rt at 140 s.

As we aimed to develop a continuous flow process with a short reactor residence time, direct temperature measurements of our flow reaction were judged to potentially impact on the mixing of reaction components and/or residence times, and we turned instead to thermal imaging of the reactor. This strategy has previously been successfully employed by Ley *et al.* to monitor the exothermic synthesis and decarboxylative dibromination of a glyoxylic acid oxime,^87^ and by Williams and co-workers to monitor a RANEY^®^ nickel catalyst fixed bed reactor,^88^ but to our knowledge has not previously been used to study metalation processes or those with reactor residence times under 1 min. Thermal imaging of a flow reaction representative of the conditions shown in Table 2, entry **3** revealed a significant exotherm during the reaction, with an increase from rt while pumping solvent alone to ∼55 °C (averaged over the whole reactor) or ∼70 °C (hottest position on reactor) while the reaction was running, validating our concerns that the reaction was at or near the THF boiling point (Fig. 2).

We decided instead to re-optimise our continuous flow reaction using a higher boiling and non-ethereal solvent, toluene. Since alkyllithiums are known to display increased reactivity in coordinating solvents such as THF,^89–91^ TMEDA was added to the feedstock solution of starting material **5** in toluene to achieve the same effect.^92^ Condition screening results are shown below in Table 3.

**Table 3:** Flow screen in toluene/TMEDA

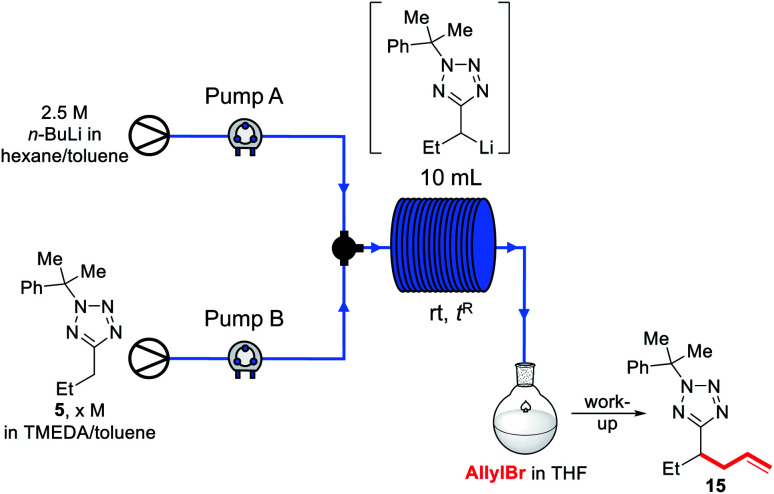						
Entry	*X* (M)	Toluene : TMEDA	Flow rate^a^ (mL min^−1^)	Residence time (min)	Yield^b^	SM^b^^,^^c^
1^d^	1.1	N/A^e^	5	2.6 s	0%	65%
2	1.1	4 : 1	1	5	62%	8%
3	1.1	4 : 1	3	1.67	60%	29%
4	1.1	4 : 1	5	1	59%	29%
5	1.1	4 : 1	10	0.5	49%	40%
6	0.8	4 : 1	5	1	46%	55%
7	1.1	1 : 1	5	1	73%	14%
8	1.1	1 : 1	3	1.67	70%	10%
**9**	**1.1**	**1** **:** **1**	**10**	**0.5**	**70%**	**8%**

a Flow rate from each pump; after junction, flow rate is doubled.

b As determined by ^1^H NMR spectroscopy in the presence of 1,3,5-trimethoxybenzene.

c Remaining starting material.

d Using a 28 cm length of tubing as a reactor.

e No TMEDA used.

In each case, base and substrate were pumped to meet at a T-junction at the same rate. We initially replicated our THF continuous flow conditions as a control (entry 1) using a 28 cm length of tubing to give a 2.6 s reactor residence time and without TMEDA, however this did not lead to appreciable conversion to product **15**, and we instead turned to the use of a 10 mL reactor to give longer residence times. Thus, pumping a 1.1 M solution of **5** in 4 : 1 toluene : TMEDA and base at 1 mL min^−1^ to give a residence time of 5 min before pumping into a stirred solution of allyl bromide in toluene gave a 62% conversion to **15**, and 8% residual starting material (entry 2), indicating that the lithiated intermediate may be unstable over a 5 min timescale. Neither increasing the flow rate to reduce residence times (entries 3–5) nor reducing the concentration of the substrate solution (entry 6) increased the conversion, though more residual substrate was observed. Instead, the TMEDA : toluene ratio was increased to 1 : 1; this indeed led to an increase in yield (entries 7–9). As a higher flow rate corresponds to a higher productivity rate, the conditions shown in entry 9 were taken as optimal.

**Fig. 3:**
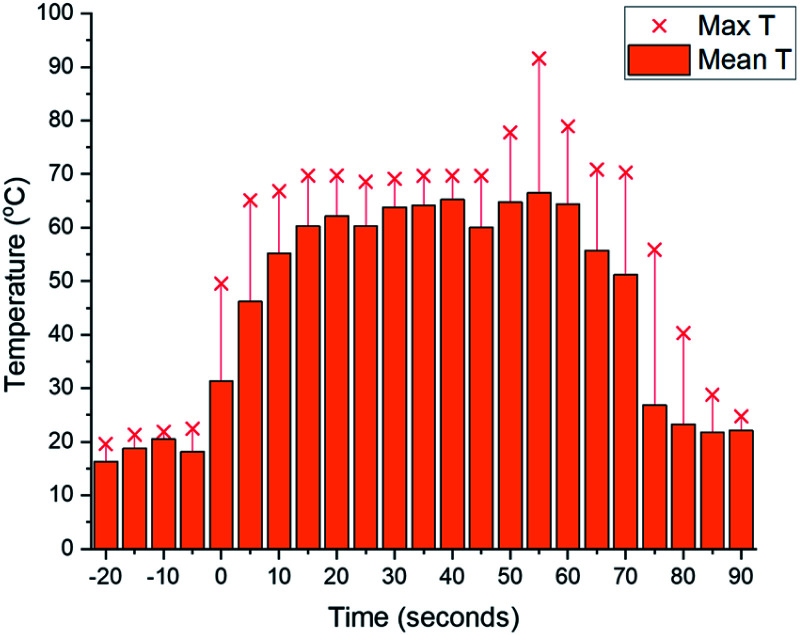
Thermal imaging of flow metalation in toluene/TMEDA: graph of mean and maximum reactor temperatures over time. Only solvent at rt is pumping until 0 s, at which base and starting material solutions (both at rt) are pumped to the reactor. Pumping is switched back to solvent only at rt at 60 s.

Before exemplifying our optimised flow conditions, we first replicated our thermal imaging experiments to probe any exotherms when using toluene (Fig. 3). Under the conditions show in Table 3, entry 9, during the reaction a mean reactor temperature of ∼65 °C, with a maximum temperature of ∼75 °C at the hottest point of the reactor was observed. We note that the differing solution structures and reactivities of organolithiums in THF and toluene are well documented and may account for this difference in exotherm magnitude and also note that the maximum temperature reached by the THF reaction corresponds to the solvent boiling point.^89^ While this represents a higher exotherm then when using THF, the reaction system was now free of ethereal solvent and vapourisation of the solvent within the tubing was not observed. We note that flash chemical C–H metalation processes are underexplored, possibly due to resultant exotherms and the difficulty of temperature monitoring. In this context, thermal imaging may supply a general strategy for overcoming current limitations in the field.

**Fig. 4:**
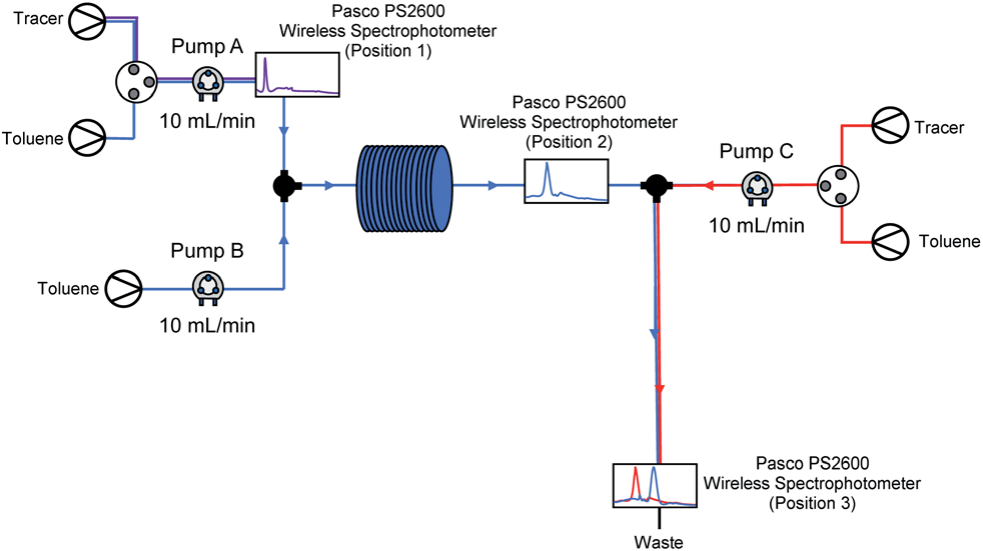
Flow system diagram for RTD determination.

Flash chemistry is commonly asserted to operate under plug flow, in which there is little forward or backward mixing of components in the flow system. Usually, this is taken to mean that residence time distributions (RTDs) are limited to a small range, with a Péclet number (Pe) over 100.^93,94^ As we suspected that our lithiated intermediate may be unstable over longer reactor residence times (see Table 3, entry 2 *vs.* entries 3–5), we decided to investigate RTDs for our optimised system with a third pump added, corresponding to an in-flow electrophilic quenching step in order to determine whether our system was operating under plug flow during (a) metalation (pump A to position 2) and through (b) the metalation/electrophilic trapping sequence (pump A to position 3) representative of conducting the whole procedure in flow – as electrophilic trapping (while typically fast) is non-instantaneous, an unstable metalated intermediate could potentially still exhibit significant decomposition between positions 2 and 3 before trapping takes place (Fig. 4).

Using the method described by Sans and García-Verdugo,^95^ a tracer was injected into the system using a valve just after a pump, and detected downstream using a UV/Vis spectrometer. In each case, all three pumps A–C (corresponding to those pumping substrate, base and electrophile in a synthetic run respectively) are pumping at 10 mL min^−1^. Representative *E*(*t*) curves for pump A only (position 1), the metalation step with just pumps A and B running (position 2), the electrophilic trapping step (pump C to position 3) with all three pumps running and a pathway representative of the path taken by substrate through the lithiation-trapping sequence (pump A to position 3) can be found in the ESI.† After averaging results over several repeat experiments in each case, RTDs were found to be in line with those ascribed to perfect plug flow: an average Pe = 66.46 for pump A to position 1, Pe = 3170.11 for pump A to position 2, Pe = 140.99 for pump C to position 3 and Pe = 769.72 for pump A to position 3. The slightly lower Pe for pump C to position 3 may be due to dispersal at the T-junction where the 10 mL min^−1^ stream from pump C and the 20 mL min^−1^ stream from pumps A and B meet, however we note this is still over the value for plug flow. For a full breakdown of RTD determinations and Pe calculations, see ESI.†

**Scheme 8:**
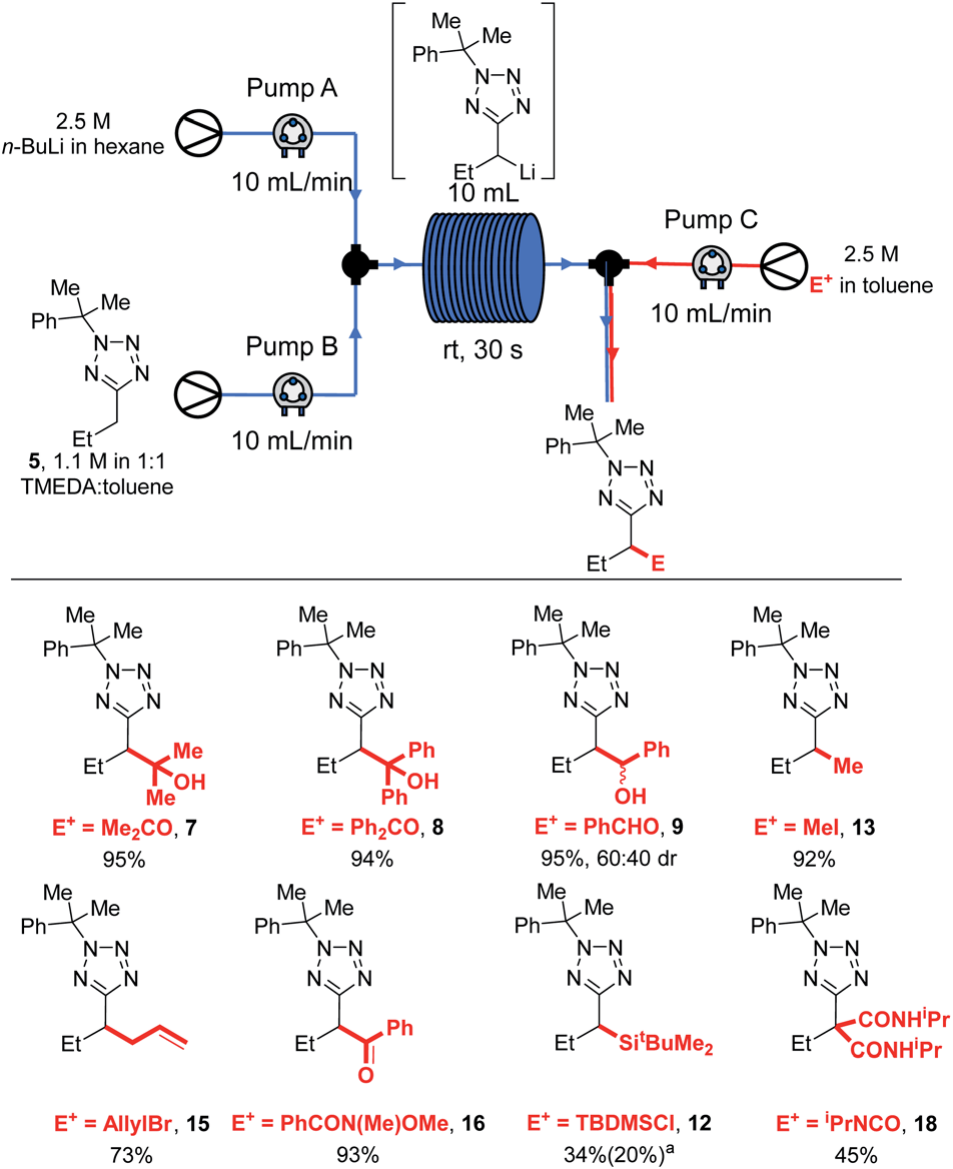
Electrophile scope in flow. ^*a*^34% isolated yield of **12**, 20% recovered **5**.

With these results in hand, we were confident that an in-flow electrophilic trapping step could be added to our system. We first set out to determine the electrophile scope (Scheme 8).

Most electrophiles investigated reacted to give the products in higher yields under our room temperature continuous flow conditions than were obtained in batch conditions. Thus, an 95% yield of **7** was isolated after trapping with acetone, compared to a 65% yield after reaction at 0 °C in batch (Scheme 2). Benzophenone and benzaldehyde gave **8** and **9** in 94% and 95% yields respectively (*vs.* 85% and 75% in batch). We found that lithiation-trapping reactions using Me_2_SO_4_ are incompatible with continuous flow conditions due to the formation of an insoluble by-product, however methylation to give **13** could be achieved in 92% yield using MeI. **15** and **16** were obtained in 73% and 93% yield after trapping with allyl bromide and a Weinreb amide respectively. A lower yield of **12** was obtained after trapping with TBDMSCl – 20% in flow *vs.* 80% isolated from a −78 °C reaction. This result is unsurprising as lithiated **5** is thought to be unstable at rt, and TBDMS reacts more slowly than the other electrophiles used. Finally, di-substituted product **18** was obtained in 45% yield after trapping with an isocyanate.

**Scheme 9:**
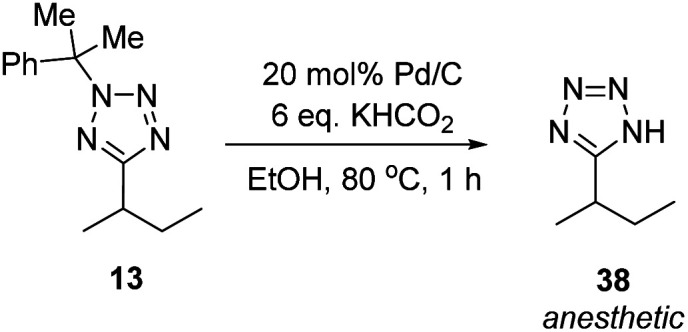
Synthesis of an anaesthetic agent.

Methylated product **13** represents a cumyl-protected analogue of a compound patented in 2018 for use in anesthesia.^96^ To demonstrate the utility of our new synthetic protocol, **13** was deprotected *via* the protocol described by Flippin using palladium on carbon and KHCO_2_ to give **38** in 94% yield (Scheme 9).^23^

**Scheme 10:**
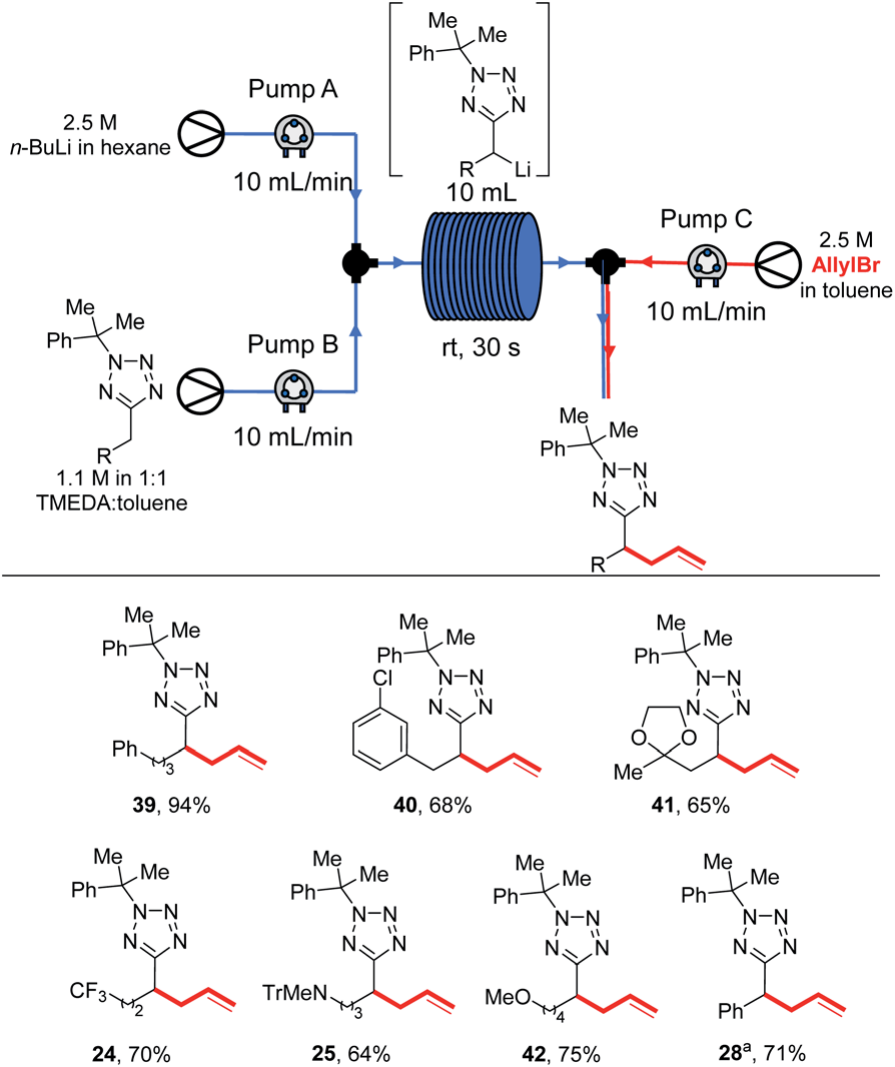
Substrate scope in flow. ^*a*^1.7 M solution of substrate used.

With the electrophile scope in hand, we next investigated the in-flow substrate scope (Scheme 10).

Again, for several substrates superior yields were obtained under continuous flow conditions at rt than under batch results: phenyl product **39** was obtained in 94% yield (*vs.* 83% from an allyl bromide-trapped 0 °C batch reaction), chlorophenyl **40** in 68% (*vs.* 53% from a −78 °C batch reaction trapped with Ph_2_CO) with no evidence of the regioisomer analogous to **31** observed. Acetal **41** was obtained in 65% yield (*vs.* 50% trapping with allyl bromide in batch at −78 °C). Trifluoromethyl, amino and alkoxy products **24**, **25** and **42** were obtained in 70%, 64% and 75% yields respectively. During our previous study of benzyltetrazole lithiations we had been unable to develop a continuous flow protocol and were pleased to be able to isolate **28** in 71% yield.

**Scheme 11:**
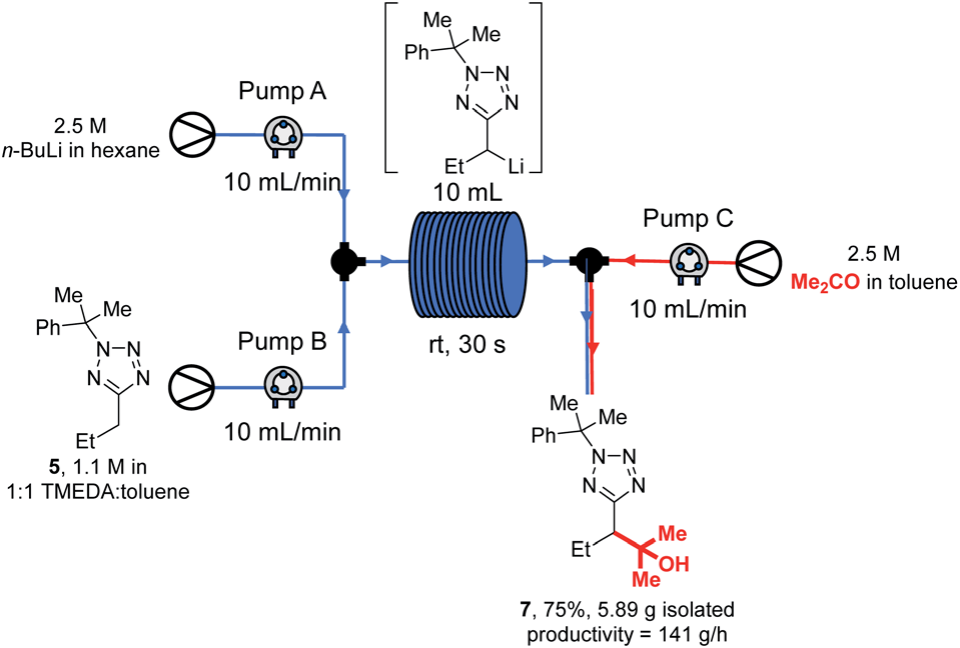
Large scale continuous flow lithiation-trapping.

With our new continuous flow procedure fully exemplified, the scalability was demonstrated with a longer run, and the productivity rate determined (Scheme 11).

Thus, tetrazole **5**, *n*-BuLi and acetone were reacted under our standard flow conditions for 2.5 min, giving 5.89 g of product **7** (75% yield) after work up and purification. This corresponds to a productivity rate of 141 g h^−1^, or alternatively 2.04 h would be required to produce 1 mole of **7**, and 7.09 h would be needed to make 1 kg. This scale of synthesis is in line with that commonly achieved in the kilo lab, the first step in API synthesis scale up, as recently discussed by Casey *et al.*^97^ Precipitation resulting in blockage of continuous flow systems is occasionally observed over extended reaction times.^98^ In our case, careful inspection of the flow system and collection vessel revealed no evidence of precipitate after the large scale experiment. We note that in our experience, precipitates from organolithium reactions are often soluble in THF, and should other researchers encounter this problem a periodic valve-controlled automated flush of the flow system with dry THF may provide a solution.

Finally, we note that the requirement for 20 mol% palladium on carbon in the deprotection protocol originally reported by Flippin may be off-putting to some researchers, particularly those engaged in large-scale synthesis.^23^ In response, we decided to optimise this procedure for lower catalyst loadings (Table 4).

**Table 4:** Optimisation of cumyl deprotection

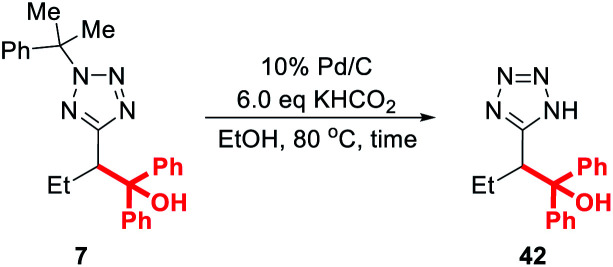				
Entry	Loading of 10% Pd/C (mol%)	Reaction time (h)	Conversion^a^	Yield^b^
1	20	1	100%	80%
2	10	1	100%	78%
3	5	4	100%	75%

a As determined by ^1^H NMR spectroscopy in the presence of 1,3,5-trimethoxybenzene.

b Isolated yield.

Thus, treatment of **7** with 20 mol% of 10% Pd/C and 6 eq. KHCO_2_ in ethanol at 80 °C for 1 h (the conditions reported by Flippin) gave an 80% yield of **42** after purification (entry 1). A comparable yield (78%) was obtained after dropping the catalyst loading to 10 mol% (entry 2). After further dropping the catalyst loading to 5 mol%, an extended reaction time of 4 h was required for complete conversion, and **42** was obtained in 75% yield. Any further reduction of the amount of Pd/C resulted in negligible conversion to the deprotected product.

## Experimental

For full experimental details and analytical data, see ESI.†

### General optimised procedure for batch lithiation-trapping

Base (2.3 eq. *n*-BuLi or 1.6 eq. *s*-BuLi) was added dropwise to a stirred solution of *N*-cumyltetrazole (1.0 eq.) in THF at a specified temperature 0 °C (when using *n*-BuLi) or −78 °C (when using *s*-BuLi) under N_2_. The resulting solution was stirred at the specified temperature for 15 min. Then, electrophile (2.0 eq.) was added and the resulting solution was stirred at the specified temperature for 10 min and then allowed to warm to rt over 16 h. Saturated NH_4_Cl_(aq)_ was then added and the two layers were separated, extracting the aqueous with Et_2_O (×3) The combined organic layers were dried (MgSO_4_) and evaporated under reduced pressure to give the crude product.

### General optimised procedure for continuous flow lithiation-trapping

A 1.1 M solution of *N*-cumyltetrazole (1.0 eq.) in 1 : 1 toluene/TMEDA (2.5 M) (0.5 mL) and a 2.5 M solution of *n*-BuLi in hexanes (0.5 mL) were driven through two separate peristaltic pumps at 10 mL min^−1^ at rt. The solutions were pumped through 1 mm I.D. PTFE tubing and mixed at a T-junction. The resulting solution was passed through a 10 mL PFA reactor (20 mL min^−1^, 10 mL, *t*^R1^ = 30 s) before mixing at a second T-junction with a 2.5 M solution of electrophile pumped at 10 mL min^−1^ through a third peristaltic pump. The resulting solution was passed through a second length of tubing (30 mL min^−1^, 0.47 mL, *t*^R2^ = 1.94 s) and collected as the product solution. A saturated solution of NH_4_Cl_(aq)_ was added to the collected solution and the layers were separated, extracting the aqueous with Et_2_O (×3). The combined organic layers were dried (MgSO_4_) and evaporated under reduced pressure to give the crude product.

## Conclusions

To conclude, we have developed a convenient and general heterobenzylic C–H functionalisation of readily deprotected 5-alkyltetrazoles, affording access to α-functionalised *N*–H tetrazolyl products which are bioisosteric with amino acid derived carboxylates bearing α-stereocentres. Under batch conditions, tetrazoles were metalated in THF using *n*-BuLi at 0 °C or *s*-BuLi at −78 °C before trapping with a wide range of electrophiles in up to 97% yield. Under continuous flow conditions, metalation was achieved in toluene/TMEDA using *n*-BuLi at room temperature at a high flow rate, short reactor residence time and corresponding narrow residence time distribution to avoid decomposition of an unstable organolithium intermediate. Products were obtained in up to 95% yield including in cases where batch synthesis resulted in modest yields, even with reaction cooling. The optimisation of continuous flow conditions was facilitated using thermal imaging to identify potentially unsafe exotherms, alerting us to re-optimise reaction conditions to avoid boiling ethereal solvent. We note that standardisation of flow processes remains a topic of discussion and while the 1 mm internal diameter tubing we have used is ubiquitous in research-scale flow chemistry, researchers wishing to replicate our chemistry with different reactor architectures may wish to re-optimize conditions. For researchers wishing to scale-up this work, we point to numbering-up as a convenient strategy for continuous flow chemistry in which simply using a larger reactor is unattractive.^99,100^ Given the requirement of flash chemistry to take place with short residence times and perfect plug flow, as well as the engineering challenges posed by flash protocols, we propose that thermal imaging has significant advantages over directly measuring reaction temperatures in cases where active thermal control is not being used. Our optimised continuous flow conditions facilitated the synthesis of a functionalised alkyltetrazole with a high productivity rate of 141 g h^−1^ – this has been achieved using an entry-level (Vapourtec E-series) flow system.

## Data availability

All experimental data and procedures are available in the ESI.†

## Author contributions

Author contributions are itemised using the CRediT system below: JYFW: data curation, formal analysis, investigation, methodology, visualisation, writing – original draft. CGT: data curation, formal analysis, investigation, methodology, visualisation. FV: funding acquisition, resources, supervision, writing – review & editing. GB: conceptualisation, data curation, funding acquisition, project administration, resources, supervision, visualisation, writing – original draft, writing – review & editing.

## Conflicts of interest

There are no conflicts to declare.

## Supplementary Material

SC-012-D1SC04176B-s001

SC-012-D1SC04176B-s002
