# Supplementary material for: Flash chemistry enables high productivity metalation-substitution of 5-alkyltetrazoles
Source: Chem Sci. 2021 Sep 15;12(40):13413–24. doi: 10.1039/d1sc04176b (PMC8528014; doi:10.1039/d1sc04176b)
Supplement: SC-012-D1SC04176B-s001 [file SC-012-D1SC04176B-s001.pdf]

Electronic Supplementary Material (ESI) for Chemical Science.
This journal is © The Royal Society of Chemistry 2021

Flash Chemistry Enables High Productivity C-H Functionalisation of Alkyltetrazoles

Jeff Y. F. Wong, Christopher G. Thomson, Filipe Vilela and Graeme Barker*

Institute of Chemical Sciences, Heriot-Watt University, Edinburgh EH14 4AS, Scotland, UK.

graeme.barker@hw.ac.uk

Index

1. Experimental details S2

1.1 General Methods S2

1.2 Starting material synthesis S3

1.3 General Procedures S4

1.4 Setup for the continuous flow lithiation S6

1.5 Experimental Procedures and Characterisation Data S7

2. Single crystal x-ray data S70

3. Thermal imaging analysis S72

4. Residence Time Distribution (RTD) Analysis S74

5. References S80

6. $^{1}H/^{13}C/^{19}F$ NMR Spectra S81

1. Experimental details

1.1 General Methods

All non-aqueous reactions were carried out under oxygen free N_2 using flame-dried glassware. Et_2O was freshly distilled from CaH_2, THF, DMF and PhMe was purified by MBRAUN SPS-800 solvent purification system. Alkyllithiums were titrated against *N*-benzylbenzamide before use. All diamines used in lithiations were distilled over CaH_2 before use. Petroleum ether refers to the fraction of petroleum ether boiling in the range 40-60 °C and was purchased in Winchester quantities. Brine refers to a saturated solution. Water is distilled water. All lithiation reactions under continuous flow conditions were carried out in a Vapourtec E-series flow microreactor equipped with peristaltic pumps, T-junctions, 10 mL PFA reactor and 1 mm I.D. PTFE tubings (L = 28 or 60 cm). The whole reaction system was flushed with nitrogen and dry THF or PhMe before use.

Flash column chromatography was carried out using Matrix silica gel 60 from Fluorochem. Thin layer chromatography was carried out using commercially available Merck F254 aluminium backed silica plates visualized by UV (254 nm) Proton (300 or 400 MHz) and carbon (75.5 or 101 MHz) NMR spectra were recorded on Bruker AV 300 or AVIII HD respectively. Fluorine NMR spectra (376 MHz) were recorded on Bruker AVIII HD. For samples recorded in $CDCl_3$ or acetone-d_6, chemical shifts are quoted on parts per million relative to $CHCl_3$ (δ_H 7.26), acetone-d_6 (δ_H 2.05, central line of quintet) or and $CDCl_3$ (δ_C 77.0, central line of triplet) or acetone-d_6 (δ_C 29.8, central line of septet). Carbon NMR spectra were recorded with broad band decoupling and assigned using DEPT experiments. Coupling constants (*J*) are quoted in Hertz. Melting points were obtained on a Stuart Scientific SMP 10 at ambient pressure. Infrared spectra were recorded on a Perkin-Elmer Spectrum 100 FT-IR Universal ATR Sampling Accessory deposited neat to a diamond/ZnSe plate. Mass spectra were obtained at the EPSRC UK National Mass Spectrometry Facility at Swansea University and SIRCAMS at University of Edinburgh. Chiral stationary phase HPLC was performed on an Agilent Technologies 1120 Compact LC.

1.2 Starting material synthesis

Tetrazoles **ST1–ST8** and ester **SA1** were synthesised according to literature procedures.[1,2]

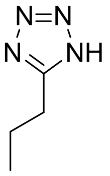

ST1

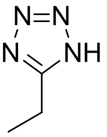

ST2

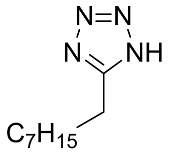

ST3

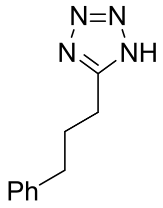

ST4

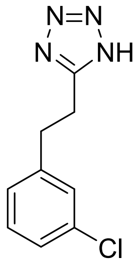

ST5

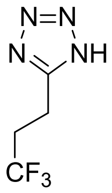

ST6

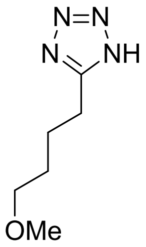

ST7

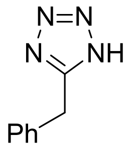

ST8

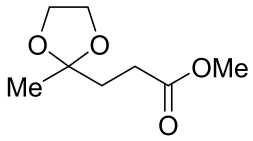

SA1

Alkene **SA2** was synthesised according to literature procedure.[3]

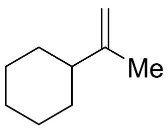

SA2

Amino nitriles **SN1–SN3** was synthesised according to literature procedure from the corresponding amino acids.[4]

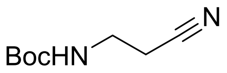

SN1

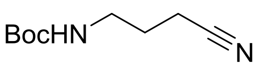

SN2

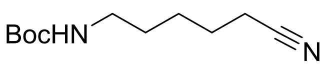

SN3

Ligand (*R*,*R*)-**L2** to (*S*,*S*)-**L9** were synthesised according to literature procedure from the corresponding cyclohexyl-1,2-diamine and amino acids.[5,6]

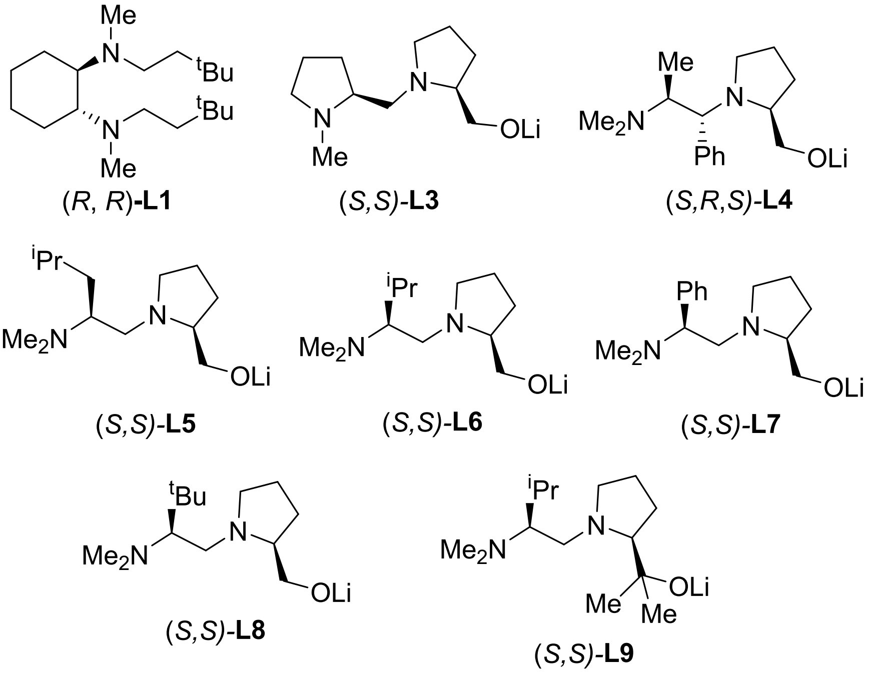

(*R*, *R*)-**L1** (*S*,*S*)-**L3** (*S*,*R*,*S*)-**L4**

(*S*,*S*)-**L5** (*S*,*S*)-**L6** (*S*,*S*)-**L7**

(*S*,*S*)-**L8** (*S*,*S*)-**L9**

1.3 General procedures

General procedure A: synthesis of 5-substituted tetrazoles

Nitrile (1.0 eq) was added to a stirred suspension of NaN_3 (3.0 eq) and $Et_3N{\cdot}HCl$ (3.0 eq) in PhMe (0.3 M) at rt. The resulting suspension was stirred and heated at 110 °C for 16 h. The resulting solution was allowed to cool to rt and then cooled to 0 °C. 6 M $HCl_{(aq)}$ or 1 M citric acid was added and the two layers were separated, extracting the aqueous with EtOAc (×3). The combined organic layers were dried ($MgSO_4$) and evaporated under reduced pressure to give the crude product.

General procedure B: protection of 5-substituted tetrazoles

Alkene (1.02 eq) was added to a stirred solution of tetrazole (1.0 eq) and TFA (2.1 eq) in $CHCl_3$ (0.2 M) at rt, the resulting solution was stirred at rt for 1 h. 2M $NaOH_{(aq)}$ was added the two layers were separated, extracting the aqueous with $CHCl_3$ (×1). The combined organic layers were washed with brine, dried ($MgSO_4$) and evaporated under reduced pressure to give the crude product.

General procedure C: methylation of *N*-Boc carbamates

NaH (60% suspension in mineral oil, 2.0 eq) was added into a stirred solution of tetrazoles (1.0 eq) in THF (0.5 M) at 0 °C in one portion and the resulting suspension was stirred at 0 °C for 10 min. MeI (1.5 eq) was added and the resulting suspension was then then allowed to warm to rt and stirred over 16 h. Saturated $NH_4Cl_{(aq)}$ was added and the two layers were separated, extracting the aqueous with Et_2O (×3) The combined organic layers were washed with 0.5 M $Na_2S_2O_{3(aq)}$, dried ($MgSO_4$) and evaporated under reduced pressure to give the crude product.

General procedure D: lithiation-substitution of 5-substituted tetrazoles

Base (1.5, 1.6, 2.1 or 2.3 eq) was added dropwise to a stirred solution of *N*-protected tetrazole (1.0 eq) in THF at a specified temperature (0 °C, –30 or –78 °C) under N_2. The resulting solution was stirred at a specified temperature (0 °C, –30 or –78 °C) for a specific time (15, 30 or 60 min). Then, electrophile (2.0 or 2.5 eq) was added and the resulting solution was stirred at the specified temperature for 10 min and then allowed to warm to rt and stirred over 16 h. Saturated $NH_4Cl_{(aq)}$ was added and the two layers were separated, extracting the aqueous with Et_2O (×3) The combined organic layers were dried ($MgSO_4$) and evaporated under reduced pressure to give the crude product.

General procedure E: lactam cyclisation of *N*MeBoc aminotetrazoles

s-BuLi (2.0 or 3.2 eq) was added dropwise to a stirred solution of *N*MeBoc aminotetrazoles (1.0 eq) in THF at –78 °C under N_2. The resulting solution was stirred at –78 °C for 15 min. Then, the resulting solution was allowed to warm to rt and stirred over 16 h. Saturated $NH_4Cl_{(aq)}$ was added and the two layers were separated, extracting the aqueous with Et_2O (×3) The combined organic layers were dried ($MgSO_4$) and evaporated under reduced pressure to give the crude product.

General procedure F: lithiation-substitution of 5-substituted tetrazoles in flow

A 1.1 M solution of tetrazole in 1:1 PhMe/TMEDA (2.5-2.9 M) (0.5 mL) and a 2.5 M solution of *n*-BuLi in hexanes (0.5 mL) were driven through two separate peristaltic pumps at 10 $mLmin^{-1}$ at rt. The solutions were pumped through 1 mm I.D. PTFE tubing and mixed at a T-junction. The resulting solution was passed through a 10 mL PFA reactor (20 $mLmin^{-1}$, 10 mL, t^{R1} = 30 s) before mixing at a second T-junction with a 2.5 M solution of electrophile pumped

at 10 $mLmin^{-1}$ through a third peristaltic pump. The resulting solution was passed through a second length of tubing (30 $mLmin^{-1}$, 0.47 mL, t^{R2} = 1.94 s) and collected as the product solution. A saturated solution of $NH_4Cl_{(aq)}$ was added to the collected solution and the layers were separated, extracting the aqueous with Et_2O (×3). The combined organic layers were dried ($MgSO_4$) and evaporated under reduced pressure to give the crude product.

General procedure G: lithiation-substitution of tetrazole 5 with chiral diamines

s-BuLi (1.6 eq for ligand (+)-**L1** and (*R*, *R*)-**L2** and 3.2 eq for ligand (*S*, *S*)-**L3** to (*S*, *S*)-**L9**) was added dropwise to a stirred solution of chiral diamine (1.8 eq.) in Et_2O (2 mL) at –78 °C under N_2. The resulting solution was stirred for 10 min at –78 °C. A solution of tetrazole **5** (1.0 eq) in Et_2O (2 mL) was added dropwise *via* a cannula and the resulting solution was stirred for 1 h. Then, a solution of Me_2CO (2.0 eq.) was added. The resulting solution was stirred at –78 °C for 10 min and then allowed to warm to rt over 16 h. 1 M $HCl_{(aq)}$ (10 mL) was added and the two layers were separated extracting the aqueous with Et_2O (3 × 10 mL) and the combined organic layers were dried ($MgSO_4$) and evaporated under reduced pressure to give the crude product.

1.4 Setup for the continuous flow lithiation

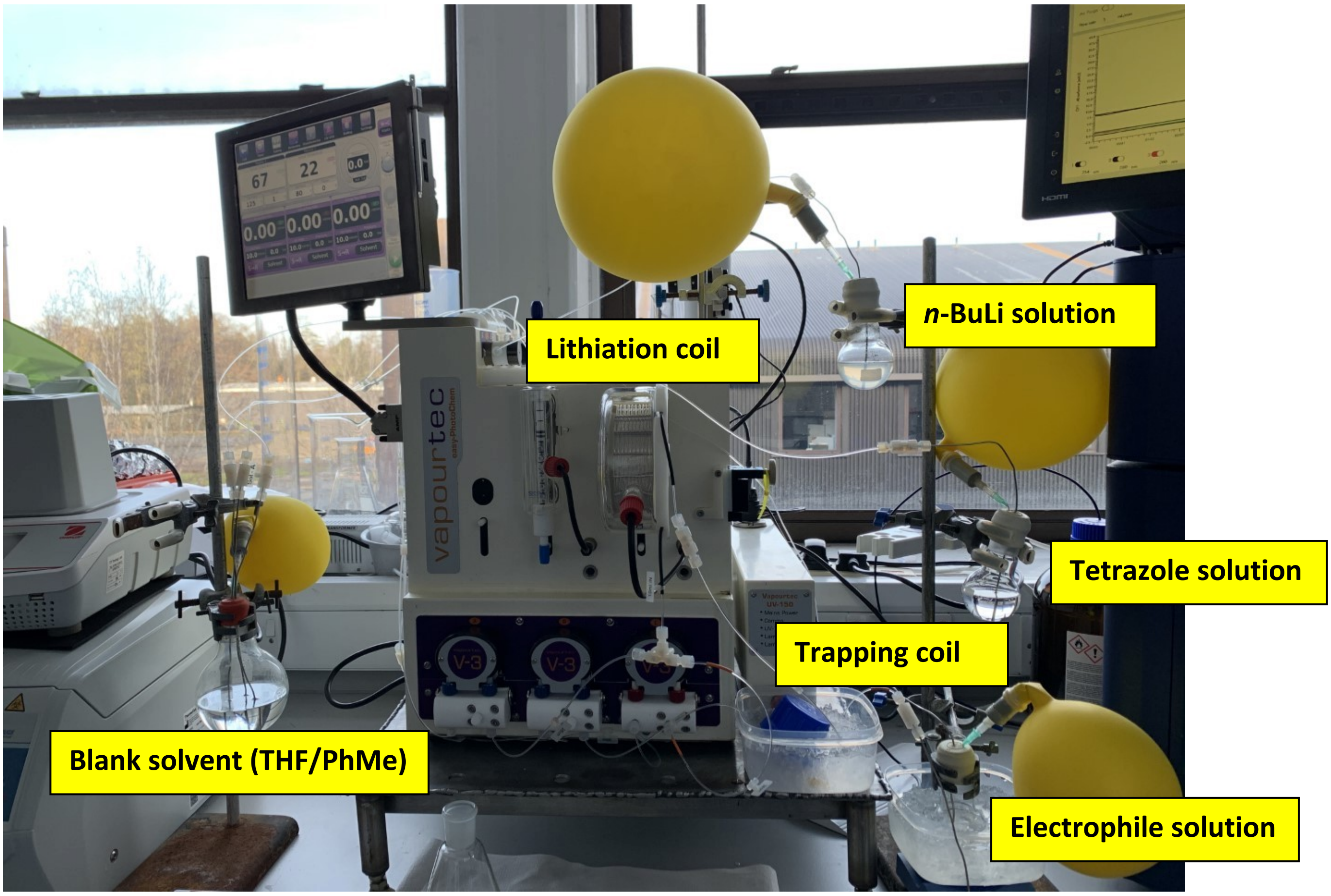

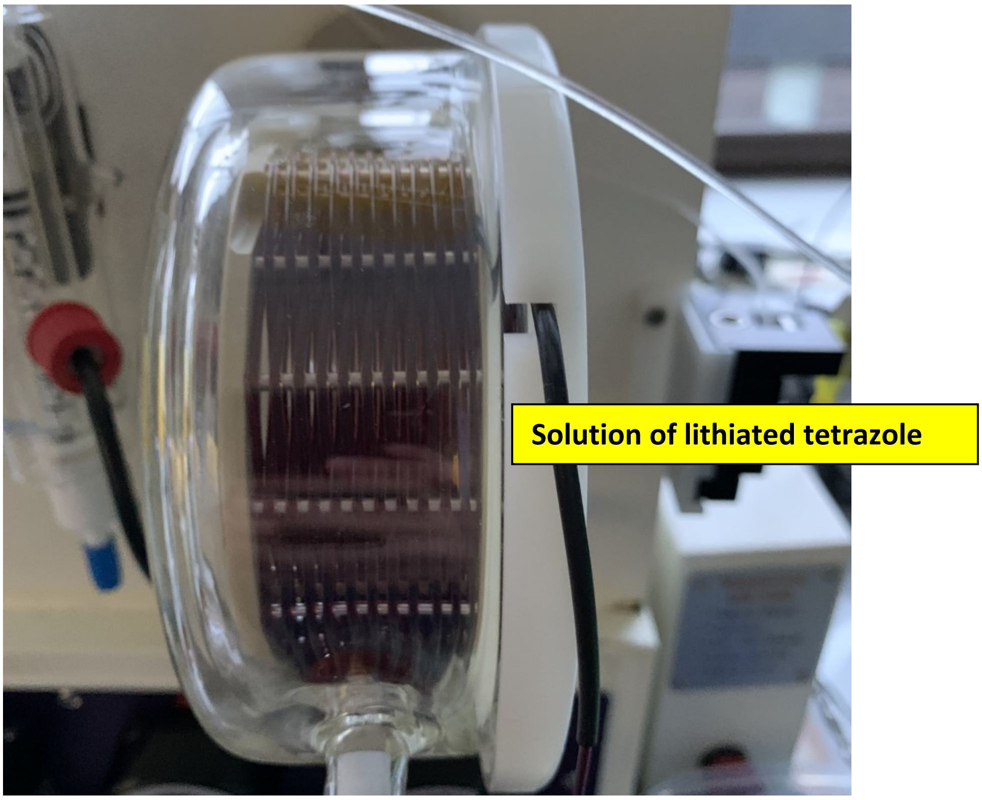

1.5 Experimental Procedures and Characterisation Data

3-(2-Methyl-1,3-dioxolan-2-yl)propenamide SA3

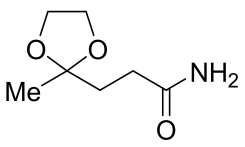

SA3

35% $NH_4OH_{(aq)}$ (4 mL) was added in to a stirred solution of ester **SA1** (1.37 g, 7.85 mmol, 1.0 eq) in MeOH (4 mL) at rt and the resulting solution was stirred at rt for 16 h. The solvent was evaporated under reduced pressure and the aqueous layer was extracted with EtOAc (10 mL × 6). The combined organic layers were dried (Na_2SO_4) and evaporated under reduced pressure to give the crude product. Purification by flash column chromatography on silica with 95:5 CH_2Cl_2-MeOH as eluent gave amide **SA3** (719 mg, 57%) as a colourless oil, R_F (95:5 CH_2Cl_2-MeOH) 0.3; IR (ATR) 3407 (N-H str), 3202 (N-H str), 2983, 2937, 2889, 1660 (C=O str), 1621, 1445, 1409, 1379, 1350, 1304, 1256, 1219, 1141, 1051, 1039, 949 cm^{-1}; ^1H NMR (400 MHz, $CDCl_3$) δ 5.71 (br s, 1H, NH), 5.48 (br s, 1H, NH), 3.99–3.91 (m, 4H, OCH_2), 2.33 (t, J = 7.5 Hz, 2H, CH_2), 2.03 (t, J = 7.5 Hz, 2H, CH_2), 1.33 (s, 3H, Me); ^{13}C NMR (101 MHz, $CDCl_3$) δ 175.2 (C), 109.3 (C), 64.7 (CH_2), 34.2 (CH_2), 30.4 (CH_2), 23.9 (CH_3). Spectroscopic data consistent with those reported in the literature.[7]

4-Hydroxybutanamide SA4

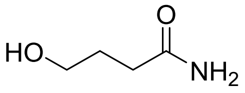

SA4

γ-Butyrolactone (15.4 mL, 17.2 g, 200 mmol, 1.0 eq) was added into 35% $NH_4OH_{(aq)}$(80 mL) at rt and the resulting solution was stirred at rt for 16 h. The solvent was evaporated under reduced pressure to give the crude product. Purification by recrystallisation from hot acetone gave amide **SA4** (15.1 g, 73%) as a white solid, m.p. 43 – 45 °C; IR (ATR) 3338 (N-H str), 3257 (O-H str) 3196 (N-H str), 1940, 2940, 2878, 1652 (C=O str), 1610, 1410, 1350, 1304, 1228, 1174, 1122, 1051, 934, 904 cm^{-1}; ^1H NMR (400 MHz, Acetone-d_6) δ 6.82 (br s, 1H, NH), 6.22 (br s, 1H, NH), 3.82 (br t, J = 6.0 Hz, 1H, OH), 3.54 (td, J = 6.0, 6.0 Hz, 2H), 2.27 (t, J = 7.5 Hz, 2H, CCH_2), 1.76 (tt, J = 7.5, 6.0 Hz, 2H, $CH_2CH_2CH_2$); ^{13}C NMR (101 MHz, Acetone-d_6) δ 174.8

(C), 61.3 (CH_2), 32.1 (CH_2), 28.5 (CH_2). Spectroscopic data consistent with those reported in the literature.[8]

4-[(*tert*-Butyldimethylsilyl)oxy]butanamide SA5

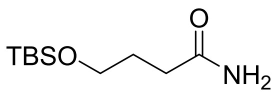

SA5

TBSCl (3.20 g, 21.2 mmol, 1.2 eq) was added into a stirred solution of amide **SA4** and imidazole (2.89 g, 42.5 mmol, 2.2 eq) in DMF (19 mL) at 0 °C and the resulting solution was allowed to warm to rt and stirred for 16 h. Water (20 mL) and EtOAc (20 mL) was added and the two layers were separated. The aqueous layer was extracted with EtOAc (20 mL × 2). The combined organic layers were washed with 1 M $HCl_{(aq)}$ (20 mL) and brine (20 mL), dried ($MgSO_4$) and evaporated under reduced pressure to give the crude product. Purification by flash column chromatography on silica with 10:90 petroleum ether-EtOAc as eluent gave amide **SA5** (1.35 g, 32%) as a white solid, m.p. 34 – 39 °C; R_F (10:90 petroleum ether-EtOAc) 0.2; ; IR (ATR) 3382 (N-H str), 3189 (N-H str), 2950, 2927, 2892, 2855, 1688, 1671 (C=O str), 1637, 1470, 1463, 1448, 1410, 1389, 1350, 1284, 1256, 1248, 1235, 1186, 1086, 1098, 1075, 1053, 1007, 963, 953, 939, 919 cm^{-1}; ^1H NMR (400 MHz, $CDCl_3$) δ 5.70 (br s, 1H, NH), 5.37 (br s, 1H, NH), 3.67 (t, J = 6.0 Hz, 2H, OCH_2), 2.33 (t, J = 7.0 Hz, 2H, CCH_2), 1.85 (tt, J = 7.0, 6.0 Hz, 2H, $CH_2CH_2CH_2$), 0.89 (s, 9H, CMe_3), 0.05 (s, 6H, $SiMe_2$); ^{13}C NMR (101 MHz, CDCl3) δ 175.4 (C), 62.3 (CH_2), 32.5 (CH_2), 28.3 (CH_2), 25.9 (CH_3), 18.3 (C), -5.4 (CH_3); MS (ESI) *m/z* 218 [(M + H)$^+$, 100]; HRMS (ESI) *m/z* $C_{10}H_{24}NO_2Si$ ([M + H]$^+$) calcd for 218.1571, found 218.1575.

4-[(*tert*-Butyldimethylsilyl)oxy]butanenitrile SN4

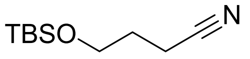

SN4

TFAA (0.21 mL, 315 mg, 1.5 mmol, 1.5 eq), was added dropwise to a solution of amide **SA5** (217 mg, 1.0 mmol, 1.0 eq) and Et_3N (0.29 mL, 212 mg, 2.1 mmol, 2.1 eq) in CH_2Cl_2 (2 mL) at 0 °C. The resulting solution was allowed to warm to rt and stirred for 1 h. Water was added and the two layers were separated. The aqueous layer was extracted with CH_2Cl_2 (5 mL × 3). The combined organic layers were dried ($MgSO_4$) and evaporated under reduced pressure to

give the crude product. Purification by flash column chromatography on silica with 90:10 petroleum ether-EtOAc as eluent gave nitrile **SN4** (160 mg, 80%) as a colourless oil, R_F (90:10 petroleum ether-EtOAc) 0.2; IR (ATR) 2955, 2930, 2885, 2857, 2249 (C≡N str), 1472, 1464, 1426, 1389, 1253, 1194, 1105, 1075, 1007, 977, 939, 916 cm^{-1}; 1H NMR (400 MHz, $CDCl_3$) δ 3.71 (t, *J* = 6.0 Hz, 2H, OCH_2), 2.45 (t, *J* = 7.0 Hz, 2H, CCH_2), 1.85 (tt, *J* = 7.0, 6.0 Hz, 2H, $CH_2CH_2CH_2$), 0.89 (s, 9H, CMe_3), 0.07 (s, 6H, $SiMe_2$); ^{13}C NMR (101 MHz, $CDCl_3$) δ 119.7 (C), 60.6 (CH_2), 28.5 (CH_2), 25.9 (CH_3), 18.6 (C), 13.7 (CH_2), –5.5 (CH_3). Spectroscopic data consistent with those reported in the literature.[9]

4-Hydroxybutanenitrile SN5

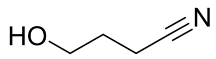

SN5

TBAF (19.4 mL of a 1.0 M solution in THF, 19.4 mmol, 1.5 eq) was added into a stirred solution of nitrile **SN4** (3.22 g, 16.2 mmol, 1.0 eq) in THF (65 mL) at 0 °C. The resulting solution was allowed to warm to rt and stirred for 1 h. Saturated $NH_4Cl_{(aq)}$ was added and the two layers were separated. The aqueous layer was extracted with EtOAc (40 mL × 3). The combined organic layers were dried ($MgSO_4$) and evaporated under reduced pressure to give the crude product. Purification by flash column chromatography on silica with 40:60 petroleum ether-EtOAc as eluent gave nitrile **SN5** (1.17 g, 85%) as a colourless oil, R_F (40:60 petroleum ether-EtOAc) 0.2; IR (ATR) 3394 (O-H str), 2941, 2885, 2250 (C≡N str), 1645, 1425, 1353, 1175, 1058, 939, 904, 835 cm^{-1}; ^1H NMR (400 MHz, $CDCl_3$) δ 3.78 (t, *J* = 6.0 Hz, 2H, OCH_2), 2.50 (t, *J* = 7.0 Hz, 2H, CCH_2), 1.90 (tt, *J* = 7.0, 6.0 Hz, 2H, $CH_2CH_2CH_2$), 1.60 (br s, 1H, OH); ^{13}C NMR (101 MHz, CDCl3) δ 119.6 (C), 60.4 (CH_2), 28.0 (CH_2), 13.8 (CH_2). Spectroscopic data consistent with those reported in the literature.[10]

4-(Methoxymethoxy)butanenitrile SN6

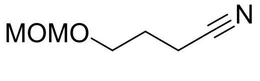

SN6

Nitrile **SN5** (4.17 g, 49 mmol, 1.0 eq) was added into a stirred suspension of LiBr (8.51 g, 98 mmol, 2.0 eq) and *p*-TsOH (932 mg, 4.9 mmol, 10 mol%) in dimethoxymethane (52 mL, 44.7 g, 588 mmol, 12 eq) at rt and the resulting suspension was stirred at rt over 16 h. Water (50

mL) was added and the two layers were separated. The aqueous layer was extracted with EtOAc (50 mL × 3). The combined organic layers were dried ($MgSO_4$) and evaporated under reduced pressure to give the crude product. Purification by flash column chromatography on silica with 99:1 CH_2Cl_2-acetone as eluent gave nitrile **SN6** (3.12 g, 49%) as a colourless oil, R_F (99:1 CH_2Cl_2-acetone) 0.2; IR (ATR) 2939, 2886, 2826, 2778, 2250 (C≡N str), 1468, 1443, 1427, 1388, 1362, 1283, 1216, 1148, 1110, 1091, 1068, 1035, 956, 917 cm^{-1}; ^1H NMR (400 MHz, $CDCl_3$) δ 4.63 (s, 2H, MeOCH_2O), 3.64 (t, J = 6.0 Hz, 2H, OCH_2OCH_2), 3.37 (s, 3H, MeO), 2.49 (t, J = 7.0 Hz, 2H, CCH_2), 1.94 (tt, J = 7.0, 6.0 Hz, 2H, $CH_2CH_2CH_2$); ^{13}C NMR (101 MHz, $CDCl_3$) δ 119.4 (C), 96.6 (CH_2), 65.2 (CH_2), 55.5 (CH_3), 25.9 (CH_2), 14.3 (CH_2); MS (ESI) m/z 152 [(M + Na)$^+$, 100], 152 [(M + Na)$^+$, 30]; HRMS (ESI) m/z $C_6H_{12}NO_2$ ([M + H]$^+$) calcd for 130.0863, found 130.0858.

5,5,5-Trifluoropentanenitrile SN7

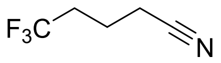

SN7

1-Bromo-4,4,4-trifluorobutane (3.23 mL, 5 g, 26.2 mmol, 1.0 eq) was added into a stirred suspension of NaCN (1.93 g, 39.3 mmol, 1.5 eq) in DMSO (50 mL) at 0 °C and the resulting suspension was allowed to warm to rt and stirred at rt for 16 h. Water (50 mL) and Et_2O (50 mL) was added and the two layers were separated. The aqueous layer was extracted with Et_2O (50 mL × 2). The combined organic layers were washed with water (20 mL × 2), dried ($MgSO_4$) and evaporated under reduced pressure to give nitrile **SN7** (3.00 g, 84%) as a colourless oil, IR (ATR) 2957, 2251 (C≡N str), 1463, 1443, 1399, 1356, 1338, 1312, 1278, 1255, 1218, 1136, 1118, 1018, 996, 914 cm^{-1}; ^1H NMR (400 MHz, $CDCl_3$) δ 2.48 (t, J = 7.0 Hz, 2H, $NCCH_2$), 2.38–2.18 (m, 2H, F_3CCH_2), 1.97 (p, J = 7.0 Hz, 2H, $CH_2CH_2CH_2$); ^{13}C NMR (101 MHz, $CDCl_3$) δ 126.3 (q, J = 276.5 Hz, C), 118.2 (C), 32.5 (q, J = 29.5 Hz, CH_2), 18.5 (q, J = 3.0 Hz, CH_2), 16.5 (CH_2); ^{19}F NMR (376 MHz, $CDCl_3$) δ –66.09 (t, J = 10.0 Hz). Spectroscopic data consistent with those reported in the literature.[11]

3-(2-Methyl-1,3-dioxolan-2-yl)propanenitrile SN8

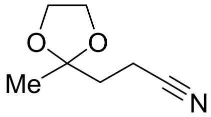

SN8

TFAA (0.95 mL, 1.41 g, 6.74 mmol, 1.1 eq), was added dropwise to a solution of amide **SA3** (714 mg, 4.49 mmol, 1.0 eq) and Et_3N (1.31 mL, 954 mg, 9.43 mmol, 2.1 eq) in CH_2Cl_2 (10 mL) at 0 °C and the resulting solution was allowed to warm to rt and stirred for 1 h. Water was added and the two layers were separated. The aqueous layer was extracted with CH_2Cl_2 (10 mL × 3). The combined organic layers were dried ($MgSO_4$) and evaporated under reduced pressure to give the crude product. Purification by flash column chromatography on silica with 70:30 petroleum ether-EtOAc as eluent gave nitrile **SN8** (594 mg, 94%) as a colourless oil, R_F (70:30 petroleum ether-EtOAc) 0.2; IR (ATR) 2985, 2940, 2890, 2248 (C≡N str), 1682, 1578, 143, 1425, 1379, 1257, 120, 1144, 1105, 1051, 949, 863 cm^{-1}; ^1H NMR (400 MHz, $CDCl_3$) δ 4.01–3.93 (m, 4H, OCH_2), 2.42 (t, *J* = 7.5 Hz, 2H, CH_2), 2.05 (t, *J* = 7.5 Hz, 2H, CH_2), 1.33 (s, 3H, Me); ^{13}C NMR (101 MHz, $CDCl_3$) δ 119.9 (C), 108.1 (C), 64.9 (CH_2), 34.5 (CH_2), 23.9 (CH_2), 11.6 (CH_3); MS (ESI) *m/z* 164 [(M + Na)$^+$, 10], 142 [(M + H)$^+$, 100]; HRMS (ESI) *m/z* $C_5H_8F_3N_4$ ([M + H]$^+$) calcd for 181.0696, found 181.0705.

5-[2-(2-Methyl-1,3-dioxolan-2-yl)ethyl]-1*H*-1,2,3,4-tetrazole ST9

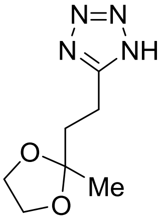

ST9

Using general procedure A, nitrile **SN8** (812 mg, 5.75 mmol, 1.0 eq), NaN_3 (1.12 g, 17.3 mmol, 3.0 eq) and $Et_3N·HCl$ (2.38 g, 17.3 mmol, 3.0 eq) in PhMe (12 mL) gave the crude product (1 M citric acid$_{(aq)}$ was used instead of 6 M $HCl_{(aq)}$). Purification by flash column chromatography on silica with 90:10 CH_2Cl_2-MeOH as eluent gave tetrazole **ST9** (690 mg, 65%) as a white solid, m.p. 111–113 °C; IR (ATR) 2990, 2903, 2692, 2463 (N-H str), 1877, 1586, 1556, 1455, 1405, 1390 ,1356, 1344, 1319, 1236, 1201, 1145, 1092, 1041, 992 cm^{-1}; ^1H NMR (400 MHz, $CDCl_3$) δ 4.11–4.01 (m, 4H, OCH_2), 3.17 (t, *J* = 6.5 Hz, 2H, $TzCH_2$), 2.21 (t, *J* = 6.5 Hz, 2H, CCH_2), 1.40 (s,

3H, Me); ^{13}C NMR (101 MHz, $CDCl_3$) δ 156.6 (C), 109.2 (C), 64.7 (CH_2), 35.6 (CH_2), 23.8 (CH_2), 17.9 (CH_3); MS (ESI) *m/z* 391 [(MM + Na)$^+$, 17], 369 [(MM + H)$^+$, 20], 207 [(M + Na)$^+$, 20], 185 [(M + H)$^+$, 100], 141 [(M – N_3H)$^+$, 21]; HRMS (ESI) *m/z* $C_7H_{13}N_4O_2$ ([M + H]$^+$) calcd for 185.1033, found 185.1049.

Note: Complete acetal deprotection of tetrazole **ST8** was observed after flash column chromatography when the synthesis was carried out on a 0.5 mmol scale.

5-(4,4,4-Trifluorobutyl)-1*H*-1,2,3,4-tetrazole ST10

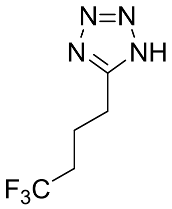

ST10

Using general procedure A, nitrile **SN7** (1.37 g, 10 mmol, 1.0 eq), NaN_3 (1.95 g, 30 mmol, 3.0 eq) and $Et_3N·HCl$ (4.13 g, 30 mmol, 3.0 eq) in PhMe (20 mL) gave tetrazole **ST10** (1.77 g, 98%) as a white solid, m.p. 68–71 °C; IR (ATR) 3135, 2976, 2953, 2863, 2700, 2615 (N-H str), 2471, 2163, 1799, 1581, 1464, 1441, 1430, 1390, 1330, 1317, 1252, 1241, 1199, 1128, 1105, 1049, 1016, 989, 969, 908 cm^{-1}; ^{1}H NMR (400 MHz, Acetone-d$_6$) δ 3.11 (t, *J* = 7.5 Hz, 2H, TzCH_2), 2.09 (m, 2H, F_3CCH_2), 2.15 – 2.07 (p, *J* = 7.5 Hz, 2H, $CH_2CH_2CH_2$), ^{13}C NMR (101 MHz, Acetone-d$_6$) δ 155.6 (C), 127.4 (q, *J* = 275.5 Hz, C), 32.3 (q, *J* = 28.5 Hz, CH_2), 21.98 (CH_2), 19.84 (q, *J* = 3.5 Hz, CH_2), ^{19}F NMR (376 MHz, Acetone-d$_6$) δ –66.94 (t, *J* = 11.0 Hz), MS (ESI) *m/z* 383 [(MM + Na)$^+$, 34], 335 [(MM + H)$^+$, 7], 190 [(M + Na)$^+$, 41], 168 [(M + H)$^+$, 100], 161 [(M – F)$^+$, 3], 138 [(M + H – N_3)$^+$, 10]; HRMS (ESI) *m/z* $C_5H_{10}N_7$ ([M + H]$^+$) calcd for 168.0992, found 168.0991.

5-[3-(Methoxymethoxy)propyl]-1*H*-1,2,3,4-tetrazole ST11

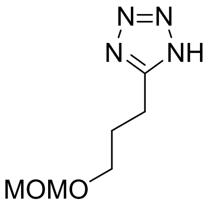

ST11

Using general procedure A, nitrile **SN6** (904 mg, 7 mmol, 1.0 eq), NaN_3 (1.37 g, 21 mmol, 3.0 eq) and $Et_3N·HCl$ (2.89 g, 21 mmol, 3.0 eq) in PhMe (14 mL) gave tetrazole **ST11** (703 mg, 84%)

as a pale yellow oil, IR (ATR) 3139, 2885 (N-H str), 2770, 2626, 2557, 1443, 1416, 1387, 1251, 1214, 1147, 1109, 1032, 951, 917 cm^{-1}; ^{1}H NMR (400 MHz, $CDCl_3$) δ 4.71 (s, 2H, MeOCH_2), 3.71 (t, *J* = 6.0 Hz, 2H, OC$H_2$$CH_2$), 3.41 (s, 3H, OMe), 3.21 (t, *J* = 7.0 Hz, 2H, TzCH_2), 2.12 (tt, *J* = 7.0, 6.0 Hz, 2H, CH_2C$H_2$$CH_2$), ^{13}C NMR (101 MHz, $CDCl_3$) δ 156.1 (C), 96.8 (CH_2), 67.0 (CH_2), 55.5 (CH_3), 27.0 (CH_2), 20.9 (CH_2), MS (ESI) *m/z* 367 [(MM + Na)$^+$, 24], 345 [(MM + H)$^+$, 39], 195 [(M + Na)$^+$, 43], 173 [(M + H)$^+$, 100], 141 [(M – OMe)$^+$, 63]; HRMS (ESI) *m/z* $C_6H_{13}N_4O_2$ ([M + H]$^+$) calcd for 173.1033, found 173.1043.

5-(4-Azidobutyl)-1*H*-1,2,3,4-tetrazole ST12

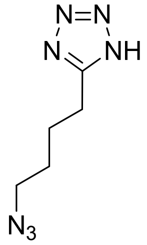

ST12

Using general procedure A, 3-methoxypropionitrile (2.25 mL, 2.35 g, 20 mmol, 1.0 eq), NaN_3 (6.50 g, 100 mmol, 5.0 eq) and Et_3N·HCl (8.24 g, 60 mmol, 3.0 eq) in PhMe (40 mL) gave the crude product. Purification by flash column chromatography on silica with EtOAc as eluent tetrazole **ST12** (2.97 g, 89%) as a yellow oil, R_F (EtOAc) 0.2; IR (ATR) 3482, 3138, 3056, 2924, 2870, 2740, 2624, 2445, 2092 (N=N=N str), 1634, 1557, 1456, 1352, 1247, 1164, 1055, 996, 888, 736, 635 cm^{-1}; ^{1}H NMR (300 MHz, $CDCl_3$) δ 3.59 (t, *J* = 6.0 Hz, 2H, N_3CH_2), 2.41 (t, *J* = 6.0 Hz, 2H, TzCH_2), 2.00–1.80 (m, 4H, CH_2); ^{13}C NMR (75.5 MHz, $CDCl_3$) δ 156.5 (C), 50.8 (CH_2), 28.1 (CH_2), 24.8 (CH_2), 23.0 (CH_2); MS (ESI) *m/z* 357 [(MM + Na)$^+$, 34], 335 [(MM + H)$^+$, 6], 190 [(M + Na)$^+$, 56], 168 [(M + H)$^+$, 100]; HRMS (ESI) *m/z* $C_5H_{10}N_7$ ([M + H]$^+$) calcd for 168.0992, found 168.0991.

Note: The azide motif of **ST12** will slowly decompose to amine at room temperature due to high nitrogen content. It is recommended to keep **ST12** in a freezer as a solid for prolong storage (i.e. >1 week).

***tert*-Butyl *N*-[3-(1*H*-1,2,3,4-tetrazol-5-yl)propyl]carbamate ST13**

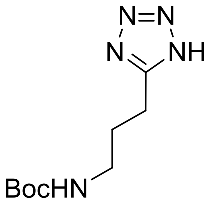

ST13

Using general procedure A, nitrile **SN2** (368 mg, 2 mmol, 1.0 eq), NaN_3 (390 mg, 6 mmol, 3.0 eq) and $Et_3N{\cdot}HCl$ (826 mg, 6 mmol, 3.0 eq) in PhMe (4 mL) gave tetrazole **ST13** (454 mg, 100%) as an off white solid, m.p. 89–93 °C; IR (ATR) 3354 (BocN-H str), 2976, 2935, 2919, 2883 (TzN-H str), 2745, 2616, 1698, 1682 (C=O str), 1560, 1529, 1479, 1435, 1414, 1391, 1366, 1355, 1311, 1293, 1266, 1255, 1194, 1162, 1141, 1115, 1088, 1080, 1061, 1041, 998, 978, 941 cm^{-1}; ^1H NMR (400 MHz, $CDCl_3$) δ 15.02 (br s, 1H, TzNH), 5.13 (br s, 1H, BocNH), 3.21 (td, *J* = 7.0, 6.5 Hz, 2H, NCH_2), 3.08 (t, *J* = 7.0 Hz, 2H, $TzCH_2$), 1.91 (tt, J = 7.0, 7.0 Hz, 2H, $CH_2CH_2CH_2$), 1.49 (s, 9H, CMe_3); ^{13}C NMR (101 MHz, $CDCl^3$) δ 158.1 (C), 155.1 (C), 81.1 (C), 38.2 (CH_2), 28.3 (CH_3), 19.5 (CH_2); MS (ESI) *m/z* 477 [$(MM + Na)^+$, 57], 455 [$(MM + H)^+$, 100], 250 [$(M + Na)^+$, 40], 228 [$(M + H)^+$, 43], 172 [$(M + H - {}^tBu)^+$, 43], 128 [$(M + H_2 - Boc)^+$, 25]; HRMS (ESI) *m/z* $C9H_{18}N_5O_2$ ($[M + H]^+$) calcd for 228.1455, found 228.1450.

***tert*-Butyl *N*-[3-(1*H*-1,2,3,4-tetrazol-5-yl)pentyl]carbamate ST14**

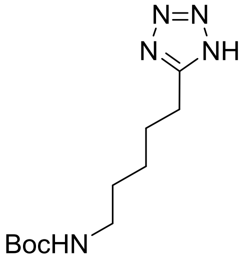

ST14

Using general procedure A, nitrile **SN3** (2.56 g, 12 mmol, 1.0 eq), NaN_3 (2.34 g, 36 mmol, 3.0 eq) and $Et_3N{\cdot}HCl$ (4.96 g, 36 mmol, 3.0 eq) in PhMe (24 mL) gave tetrazole **ST14** (3.03 g, 99%) as a white solid, m.p. 77–79 °C; IR (ATR) 3278 (N-H str), 3115, 3007, 2977, 2937, 2898, 2869, 1662 (C=O str), 1558, 1468, 1452, 1441, 1393, 1364, 1337, 1297, 1283, 1253, 1168, 1080, 1056, 995 cm^{-1}; ^1H NMR (400 MHz, $CDCl_3$)(70:30 mixture of rotamers) δ 15.33 (br s, 1H, TzNH), 4.94 (br s, 1H, BocNH), 3.12 (td, *J* = 7.0, 6.5 Hz, 2H, NCH_2), 3.07 – 3.02 (m, 2H, $TzCH_2$), 1.92 – 1.83 (m, 2H, CH_2), 1.56 – 1.51 (m, 2H, CH_2), 1.45 (s, 3H, CMe_3), 1.43 (s, 6H, CMe_3), 1.41 – 1.36

(m, 2H, CH_2); ^{13}C NMR (101 MHz, CDCl3))(mixture of rotamers) δ 157.0 (C), 156.9 (C), 156.4 (C), 80.0 (C), 79.9 (C), 40.1(CH_2), 40.0 (CH_2), 29.6 (CH_2), 29.5 (CH_2), 28.5 (CH_3), 26.7 (CH_2), 26.5 (CH_2), 25.9 (CH_2), 25.8 (CH_2), 23.3 (CH_2); MS (ESI) *m/z* 278 [$(M + Na)^+$, 90], 256 [$(M + H)^+$, 100], 200 [$(M + H - {}^tBu)^+$, 95], 156 [$(M + H_2 - Boc)^+$, 85]; HRMS (ESI) *m/z* $C_{11}H_{22}N_5O_2$ ($[M + H]^+$) calcd for 256.1768, found 256.1779.

5-Propyl-1-(triphenylmethyl)-2*H*-1,2,3,4-tetrazole 1

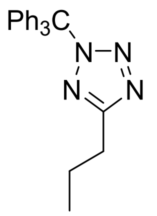

1

Trityl chloride (5.20 g, 18.7 mmol, 1.1 eq) was added to a solution of tetrazole **ST1** (1.9 g, 17 mmol, 1.0 eq), Et_3N (2.58 g, 3.55 mL, 25.5 mmol, 1.5 eq) and DMAP (208 mg, 1.7 mmol, 10 mol%) in CH_2Cl_2 (85 mL) at 0 °C. The resulting solution was warmed to rt and stirred for 16 h. Water (85 mL) was added and the two layers were separated. Extracting the aqueous with CH_2Cl_2 (3 × 60 mL) and the combined organic layers were dried ($MgSO_4$) and evaporated under reduced pressure to give the crude product. Purification by recrystallisation from hot EtOH gave tetrazole **1** (4.74 g, 80%) as a white solid, m.p. 115–117 °C; IR (ATR) 3090, 3035, 2955, 2869, 2359, 2342, 1489, 1464, 1443, 1154, 1036, 905, 761, 674 cm^{-1}; ^1H NMR (400 MHz, $CDCl_3$) δ 7.39–7.32 (m, 9H, Ph), 7.15–7.11 (m, 6H, Ph), 2.93 (t, J = 7.5 Hz, 2H, $ArCH_2$), 1.83 (sextet, J = 7.5 Hz, 2H, CH_2CH_2Me), 0.98 (t, J = 7.5 Hz, 3H, Me); ^{13}C NMR (101 MHz, $CDCl_3$) δ 165.8 (C), 141.6 (C), 130.2 (CH), 128.2 (CH), 127.7 (CH), 82.6 (C), 27.5 (CH_2), 21.6 (CH_2), 13.3 (CH_3); MS (ESI) *m/z* 377 [$(M + Na)^+$, 19], 243 [$(M - C_4H_7N_4)^+$, 100], 174 [$(M - C_{10}H_{13}N_4)^+$, 69]; HRMS (ESI) *m/z* $C_{23}H_{22}N_4Na$ ($[M + Na]^+$) calcd for 377.1737, found 377.1739.

2-(1,1-Diphenylethyl)-5-propyl-2*H*-1,2,3,4-tetrazole 3

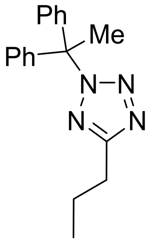

3

Using general procedure B, 1,1-diphenylethylene (0.99 mL, 1.01 g, 5.6 mmol, 1.02 eq), tetrazole **ST1** (617 mg, 5.5 mmol, 1.0 eq) and TFA (0.91 mL, 1.32 g, 11.6 mmol, 2.1 eq) in $CHCl_3$ (11 mL), gave the crude product. Purification by flash column chromatography on silica with 90:10 petroleum ether-EtOAc as eluent gave tetrazole **3** (850 mg, 53%) as a colourless oil, R_F (90:10 petroleum ether-EtOAc) 0.2; IR (ATR) 3060, 3027, 3000, 2962, 2933, 2873, 1599, 1494, 1446, 1390, 1336, 1299, 1275, 1229, 1191, 1168, 1063, 1048, 1029, 1011, 1001, 913 cm^{-1}; ^{1}H NMR (400 MHz, $CDCl_3$) δ 7.34–7.31 (m, 6H, Ph), 7.11–7.06 (m, 4H, Ph), 2.87 (t, *J* = 7.5 Hz, 2H, $TzCH_2$), 2.57 (s, 3H, CMe), 1.80 (tq, *J* = 7.5, 7.5 Hz, 2H, $MeCH_2$), 0.96 (t, *J* = 7.5 Hz, 3H, $MeCH_2$); ^{13}C NMR (101 MHz, $CDCl_3$) δ 166.3 (C), 143.1 (C), 128.3 (CH), 128.1 (CH), 127.4 (CH), 73.9 (C), 29.5 (CH_3), 27.5 (CH_2), 21.6 (CH_2), 13.7 (CH_3); MS (ESI) *m/z* 293 [$(M + H)^+$, 44], 181 [$(Ph_2MeC)^+$, 100]; HRMS (ESI) *m/z* $C_{18}H_{20}N_4$ ($[M + H]^+$) calcd for 293.1761, found 293.1768.

2-(2-Phenylpropan-2-yl)-5-propyl-2*H*-1,2,3,4-tetrazole 5

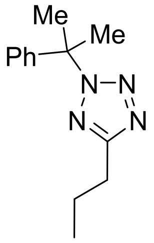

5

Using general procedure B, α-methylstyrene (0.66 mL, 603 mg, 5.1 mmol, 1.02 eq), tetrazole **ST1** (561 mg, 5 mmol, 1.0 eq) and TFA (0.88 mL, 1.31 g, 11.5 mmol, 2.1 eq) in $CHCl_3$ (10 mL), gave the crude product. Purification by flash column chromatography on silica with 90:10 petroleum ether-EtOAc as eluent gave tetrazole **5** (1.00 g, 87%) as a colourless oil, R_F (90:10 petroleum ether-EtOAc) 0.3; IR (ATR) 3063, 3030, 2988, 2963, 2874, 1601, 1496, 1449, 1391, 1370, 1310, 1254, 1210, 1185, 1162, 1071, 1031, 1106, 1002, 943, 926, 909 cm^{-1}; ^{1}H NMR (300

MHz, $CDCl_3$) δ 7.34–7.23 (m, 3H, Ph), 7.09–7.04 (m, 2H, Ph), 2.89 (t, *J* = 7.5 Hz, 2H, $TzCH_2$), 2.18 (s, 6H, CMe_2), 1.83 (tq, *J* = 7.5, 7.5 Hz, 2H, $MeCH_2$), 1.00 (t, *J* = 7.5 Hz, 3H, $MeCH_2$); ^{13}C NMR (75.5 MHz, $CDCl_3$) δ 166.5 (C), 144.1 (C), 128.6 (CH), 127.8 (CH), 124.7 (CH), 67.8 (C), 29.1 (CH_3), 27.5 (CH_2), 21.6 (CH_2), 13.7 (CH_3); MS (ESI) *m/z* 483 [$(MM + Na)^+$, 62], 462 [$(MM + H)^+$, 85], 253 [$(M + Na)^+$, 46], 231 [$(M + H)^+$, 100], 119 [$(PhMe_2C)^+$, 8]; HRMS (ESI) *m/z* $C_{13}H_{19}N_4$ ($[M + H]^+$) calcd for 231.1615, found 231.1613.

2-(2-Phenylpropan-2-yl)-5-ethyl-2*H*-1,2,3,4-tetrazole ST15

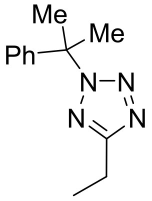

ST15

Using general procedure B, α-methylstyrene (0.66 mL, 603 mg, 5.1 mmol, 1.02 eq), tetrazole **ST2** (491 mg, 5 mmol, 1.0 eq) and TFA (0.88 mL, 1.31 g, 11.5 mmol, 2.1 eq) in $CHCl_3$ (10 mL), gave the crude product. Purification by flash column chromatography on silica with 90:10 petroleum ether-EtOAc as eluent gave tetrazole **ST15** (1.02 g, 94%) as a colourless oil, R_F (90:10 petroleum ether-EtOAc) 0.2; IR (ATR) 3063, 3029, 2982, 2938, 2878, 1602, 1498, 1449, 1392, 1370, 1303, 1254, 1186, 1161, 1106, 1062, 1020, 1002, 973, 944, 926, 910 cm^{-1}; ^1H NMR (400 MHz, $CDCl_3$) δ 7.33–7.24 (m, 3H, Ph), 7.10–7.06 (m, 2H, Ph), 2.91 (q, *J* = 7.5 Hz, 2H, $TzCH_2$), 2.15 (s, 6H, CMe_2), 1.36 (t, *J* = 7.5 Hz, 3H, $MeCH_2$); ^{13}C NMR (101 MHz, $CDCl_3$) δ 166.6 (C), 144.1 (C), 128.6 (CH), 127.8 (CH), 124.7 (CH), 67.9 (C), 29.2 (CH_3), 19.1 (CH_2), 12.5 (CH_3); MS (EI) *m/z* 216 [$(M)^+$, 2], 160 [$(BnTz)^+$, 21], 119 [$(PhMe_2C)^+$, 100], 91 [$(PhCH_2)^+$, 28]; HRMS (ESI) *m/z* $C_{12}H_{16}N_4$ ($[M]^+$) calcd for 216.1370, found 216.1368.

5-Octyl-2-(2-phenylpropan-2-yl)-2*H*-1,2,3,4-tetrazole ST16

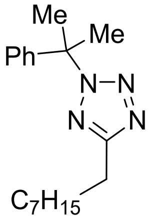

ST16

Using general procedure B, α-methylstyrene (0.66 mL, 603 mg, 5.1 mmol, 1.02 eq), tetrazole **ST3** (843 mg, 5 mmol, 1.0 eq) and TFA (0.88 mL, 1.31 g, 11.5 mmol, 2.1 eq) in $CHCl_3$ (10 mL), gave the crude product. Purification by flash column chromatography on silica with 95:5 petroleum ether-EtOAc as eluent gave tetrazole **ST16** (1.22 g, 81%) as a colourless oil, R_F (95:5 petroleum ether-EtOAc) 0.2; IR (ATR) 2987, 2953, 2925, 2855, 1603, 1497, 1449, 1391, 1370, 1310, 1253, 1187, 1162, 1107, 1072, 1030, 1018, 926, 909 cm^{-1}; ^1H NMR (400 MHz, $CDCl_3$) δ 7.33–7.24 (m, 3H, Ph), 7.08–7.05 (m, 2H, Ph), 2.87 (t, *J* = 7.5 Hz, 2H, $TzCH_2$), 2.14 (s, 6H, CMe_2), 1.76 (tt, *J* = 7.5, 7.5 Hz, 2H, $TzCH_2CH_2$), 1.36–1.22 (m, 10H, $TzCH_2CH_2(CH_2)_5Me$), 0.87 (t, *J* = 7.5 Hz, 3H, $MeCH_2$); ^{13}C NMR (101 MHz, $CDCl_3$) δ 166.7 (C), 144.1 (C), 128.6 (CH), 127.8 (CH), 124.7 (CH), 67.8 (C), 31.8 (CH_2), 29.2 (3 × CH_2), 29.1 (CH_3), 28.2 (CH_2), 25.6 (CH_2), 22.7 (CH_2), 14.1 (CH_3); MS (ESI) *m/z* 601 [$(MM + H)^+$, 100], 301 [$(M + H)^+$, 73], 224 [$(M + H – Ph)^+$, 36]; HRMS (ESI) *m/z* $C_{18}H_{28}N_4$ ($[M + H]^+$) calcd for 301.2387, found 301.2396.

5-[3-(Methoxymethoxy)propyl]-2-(2-phenylpropan-2-yl)-2*H*-1,2,3,4-tetrazole ST17

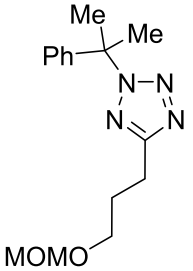

ST17

Using general procedure B, α-methylstyrene (1.80 mL, 1.64 g, 13.9 mmol, 1.02 eq), tetrazole **ST9** (2.28 g, 13.2 mmol, 1.0 eq) and TFA (2.10 mL, 3.16 g, 27.7 mmol, 2.1 eq) in $CHCl_3$ (26 mL), gave the crude product. Purification by flash column chromatography on silica with 70:30 petroleum ether-EtOAc as eluent gave tetrazole **ST17** (3.01 g, 79%) as a colourless oil, R_F (70:30 petroleum ether-EtOAc) 0.2; IR (ATR) 2988, 2937, 2882, 2823, 1496, 1448, 1391, 1371,

1308, 1255, 1214, 1189, 1146, 1109, 1071, 1036, 951, 918 cm^{-1}; ^{1}H NMR (400 MHz, $CDCl_3$) δ 7.35–7.23 (m, 3H, Ph), 7.10–7.05 (m, 2H, Ph), 4.60 (s, 2H, MeOCH_2), 3.57 (t, *J* = 6.5 Hz, 2H, OCH_2CH$_2$), 3.33 (s, 3H, OMe), 2.99 (t, *J* = 7.5 Hz, 2H, TzCH$_2$), 2.14 (s, 6H, CMe$_2$), 2.08 (tt, *J* = 7.5, 6.5 Hz, 2H, CH$_2$CH_2CH$_2$); ^{13}C NMR (101 MHz, $CDCl_3$) δ 166.0 (C), 144.0 (C), 128.6 (CH), 127.8 (CH), 124.7 (CH), 96.4 (CH$_2$), 67.9 (C), 66.5 (CH$_2$), 55.2 (CH$_3$), 29.1 (CH$_3$), 28.1 (CH$_2$), 22.4 (CH$_2$); MS (ESI) *m/z* 313 [$(M + Na)^+$, 61], 291 [$(M + H)^+$, 73], 171 [$(M + H_2 – CMe_2Ph)^+$, 90], 141 [$(M + H – CMe_2Ph – OMe)^+$, 100]; HRMS (ESI) *m/z* $C_{15}H_{23}N_4O_2$ ($[M + H]^+$) calcd for 291.1816, found 291.1813.

2-(2-Phenylpropan-2-yl)-5-(3-phenylpropyl)-2*H*-1,2,3,4-tetrazole ST18

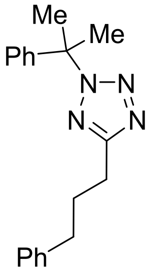

ST18

Using general procedure B, α-methylstyrene (0.66 mL, 603 mg, 5.1 mmol, 1.02 eq), tetrazole **ST4** (941 mg, 5 mmol, 1.0 eq) and TFA (0.88 mL, 1.31 g, 11.5 mmol, 2.1 eq) in $CHCl_3$ (10 mL), gave the crude product. Purification by flash column chromatography on silica with 90:10 petroleum ether-EtOAc as eluent gave tetrazole **ST18** (1.53 g, 100%) as a colourless oil, R_F (90:10 petroleum ether-EtOAc) 0.2; IR (ATR) 3085, 3061, 3026, 2988, 2938, 2861, 1602, 1584, 1496, 1449, 1391, 1370, 1308, 1255, 1186, 1161, 1107, 1073, 1022, 1002, 977, 943, 925, 909 cm^{-1}; ^{1}H NMR (400 MHz, $CDCl_3$) δ 7.33–7.25 (m, 5H, Ph), 7.20–7.16 (m, 3H, Ph), 7.09–7.06 (m, 2H, Ph), 2.91 (t, *J* = 7.5 Hz, 2H, TzCH$_2$), 2.68 (t, *J* = 7.5 Hz, 2H, PhCH$_2$) 2.15 (s, 6H, CMe$_2$), 2.10 (tt, *J* = 7.5, 7.5 Hz, 2H, TzCH$_2$CH_2); ^{13}C NMR (101 MHz, $CDCl_3$) δ 166.3 (C), 144.0 (C), 141.6 (C), 128.6 (2 × CH), 128.4 (CH), 127.8 (CH), 125.9 (CH), 124.7 (CH), 67.9 (C), 35.2 (CH$_2$), 29.8 (CH$_2$), 29.1 (CH$_3$), 25.1 (CH$_2$); MS (EI) *m/z* 306 [$(M)^+$, 4], 188 [$(M + H – CMe_2Ph)^+$, 55], 119 [$(PhCN_2)^+$, 100]; HRMS (EI) *m/z* $C_{19}H_{22}N_4$ ($[M]^+$) calcd for 306.1839, found 306.1853.

2-(2-Phenylpropan-2-yl)-5-(4,4,4-trifluorobutyl)-2*H*-1,2,3,4-tetrazole ST19

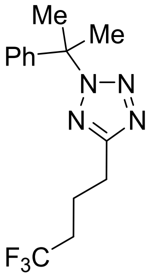

ST19

Using general procedure B, α-methylstyrene (2.40 mL, 2.23 g, 18.9 mmol, 1.02 eq), tetrazole **ST10** (3.10 g, 18.5 mmol, 1.0 eq) and TFA (2.98 mL, 38.9 g, 22.9 mmol, 2.1 eq) in $CHCl_3$ (40 mL), gave the crude product. Purification by flash column chromatography on silica with 93:7 petroleum ether-EtOAc as eluent gave tetrazole **ST19** (1.49 g, 87%) as a colourless oil, R_F (93:7 petroleum ether-EtOAc) 0.2; IR (ATR) 2990, 2946, 1602, 1498, 1449, 1391, 1372, 1309, 1274, 1253, 1206, 1184, 1131, 1107, 1074, 1021, 971, 927, 910 cm^{-1}; ^1H NMR (400 MHz, $CDCl_3$) δ 7.36–7.27 (m, 3H, Ph), 7.11–7.04 (m, 2H, Ph), 2.97 (t, *J* = 7.5 Hz, 2H, $TzCH_2$), 2.26–2.16 (m, 2H, F_3CCH_2), 2.15 (s, 6H, CMe_2), 2.13–1.99 (m, 2H, $CH_2CH_2CH_2$); ^{13}C NMR (101 MHz, $CDCl_3$) δ 165.2 (C), 143.8 (C), 128.7 (CH), 127.92 (CH), 126.9 (q, *J* = 275.5 Hz, C), 124.7 (CH), 68.1 (C), 33.1 (q, J = 29.0 Hz, CH_2), 29.1 (CH_3), 24.5 (CH_2), 20.5 (q, *J* = 3.0 Hz, CH_2); ^{19}F NMR (376 MHz, $CDCl_3$) δ -66.3 (t, *J* = 10.5 Hz); MS (ESI) *m/z* 322 [$(M + Na)^+$, 83], 299 [$(M + H)^+$, 100], 181 [$(M + H_2 - CMe_2Ph)^+$, 63]; HRMS (ESI) *m/z* $C_{14}H_{18}F_3N_4$ ($[M + H]^+$) calcd for 299.1478, found 299.1492.

5-(4-Azidobutyl)-2-(2-phenylpropan-2-yl)-2*H*-1,2,3,4-tetrazole ST20

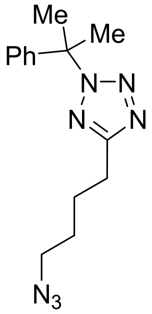

ST20

Using general procedure B, α-methylstyrene (2.40 mL, 2.23 g, 18.9 mmol, 1.02 eq), tetrazole **ST12** (3.10 g, 18.5 mmol, 1.0 eq) and TFA (2.98 mL, 38.9 g, 22.9 mmol, 2.1 eq) in $CHCl_3$ (40 mL), gave the crude product. Purification by flash column chromatography on silica with 80:20

petroleum ether-EtOAc as eluent gave tetrazole **ST20** (4.57 g, 87%) as a colourless oil, R_F (80:20 petroleum ether-EtOAc) 0.2; IR (ATR) 3062, 2989, 2941, 2868, 2092 (N=N=N str), 1601, 1496, 1449, 1392, 1371, 1352, 1305, 1251, 1187, 1161, 1107, 1072, 1019, 1002, 924, 944, 910 cm^{-1}; ^1H NMR (400 MHz, $CDCl_3$) δ 7.34–7.25 (m, 3H, Ph), 7.09–7.06 (m, 2H, Ph), 3.29 (t, *J* = 7.0 Hz, 2H, N_3CH_2), 2.92 (t, *J* = 7.5 Hz, 2H, $TzCH_2$), 2.15 (s, 6H, CMe_2), 1.91–1.83 (m, 2H, CH_2), 1.70–1.62 (m, 2H, CH_2); ^{13}C NMR (101 MHz, $CDCl_3$) δ 156.5 (C), 50.8 (CH_2), 28.1 (CH_2), 24.8 (CH_2), 23.0 (CH_2); 165.9 (C), 143.9 (C), 128.7 (CH), 127.9 (CH), 124.7 (CH), 68.0 (C), 51.0 (CH_2), 29.1 (CH_3), 28.2 (CH_2), 25.2 (CH_2), 25.0 (CH_2); MS (ESI) *m/z* 593 [$(MM + Na)^+$, 53], 571 [$(MM + H)^+$, 17], 308 [$(M + Na)^+$, 63], 286 [$(M + H)^+$, 67], 168 [$(M + H_2 – CMe_2Ph)^+$, 67]; HRMS (ESI) *m/z* $C_{14}H_{20}N_7$ ($[M + H]^+$) calcd for 286.1775, found 286.1786.

4-[2-(2-Phenylpropan-2-yl)-2*H*-1,2,3,4-tetrazol-5-yl]butan-1-amine ST21

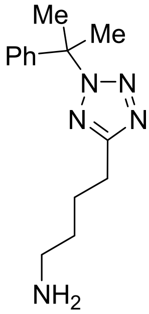

ST21

PPh_3 (1.52 g, 5.80 mmol, 1.0 eq) was added into a stirred solution of **ST20** (1.66 g, 5.80 mmol, 1.0 eq) in THF (17 mL) and H_2O (0.3 mL) in one portion at rt and the resulting solution was stirred at rt for 16 h. The solvent was evaporated under reduced pressure to give the crude product. Purification by flash column chromatography on silica with 84:15:1 CH_2Cl_2-MeOH-$NH_4OH_{(aq)}$ as eluent gave tetrazole **ST21** (1.48 g, 98%) as a colourless oil, R_F (84:15:1 CH_2Cl_2-MeOH-$NH_4OH_{(aq)}$) 0.3; IR (ATR) 3655 (N-H str), 3061, 1988, 2934, 2862, 1584, 1496, 1448, 1392, 1371, 1308, 1253, 1186, 1161, 1106, 1073, 1020, 1002, 910 cm^{-1}; ^1H NMR (400 MHz, $CDCl_3$) δ 7.33–7.24 (m, 3H, Ph), 7.08–7.05 (m, 2H, Ph), 2.89 (t, *J* = 7.5 Hz, 2H, $TzCH_2$), 2.71 (t, *J* = 7.0 Hz, 2H, NCH_2), 2.14 (s, 6H, CMe_2), 1.81 (tt, *J* = 7.5, 7.0 Hz, 2H, CH_2), 1.51 (tt, *J* = 7.5, 7.0 Hz, 2H, CH_2), 1.10 (br s, 2H, NH_2); ^{13}C NMR (101 MHz, $CDCl_3$) δ 166.4 (C), 144.0 (C), 128.6 (CH), 127.8 (CH), 124.7 (CH), 67.9 (C), 41.9 (CH_2), 33.3 (CH_2), 29.1 (CH_3), 25.5 (CH_2), 25.4 (CH_2); MS (ESI) *m/z* 544 [$(MM + Na)^+$, 9], 519 [$(MM + H)^+$, 49], 282 [$(M + Na)^+$, 8], 260 [$(M + H)^+$, 100],

142 [(M + H_2 – $CMe_2Ph)^+$, 29]; HRMS (ESI) *m/z* $C_{14}H_{22}N_5$ ([M + H]$^+$) calcd for 260.1870, found 260.1884.

tert-Butyl _N_-{4-[2-(2-phenylpropan-2-yl)-2_H_-1,2,3,4-tetrazol-5-yl]butyl}carbamate ST22

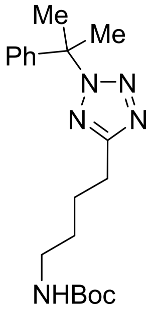

ST22

Boc_2O (1.20 g, 5.5 mmol, 1.1 eq) was added into a stirred solution of **ST21** (1.30 g, 5 mmol, 1.0 eq) in CH_2Cl_2 (10 mL) at rt and the resulting solution was stirred at rt for 16 h. The solvent was evaporated under reduced pressure to give the crude product. Purification by flash column chromatography on silica with 70:30 petroleum ether-EtOAc as eluent gave tetrazole **ST22** (1.68 g, 93%) as a colourless oil, R_F (70:30 petroleum ether-EtOAc) 0.2; IR (ATR) 3353 (N-H str), 2977, 2934, 2866, 1694 (C=O str), 1601, 1497, 1449, 1391, 1365, 1308, 1248, 1164, 1073, 1019, 1002, 943, 926 cm^{-1}; ^{1}H NMR (400 MHz, $CDCl_3$) δ 7.33–7.24 (m, 3H, Ph), 7.09–7.05 (m, 2H, Ph), 4.52 (br s, 1H, NH), 3.13 (br dt, *J* = 6.5, 6.5 Hz, 2H, NCH_2), 2.89 (t, *J* = 7.5 Hz, 2H, $TzCH_2$), 2.14 (s, 6H, CMe_2), 1.80 (tt, *J* = 7.5, 7.0 Hz, 2H, CH_2), 1.54 (tt, *J* = 7.5, 7.0 Hz, 2H, CH_2), 1.43 (s, 9H, CMe_3); ^{13}C NMR (101 MHz, $CDCl_3$) δ 166.2 (C), 155.9 (C), 144.0 (C), 128.6 (CH), 127.8 (CH), 124.7 (CH), 79.1 (C), 67.9 (C), 40.2 (CH_2), 29.5 (CH_2), 29.1 (CH_3), 28.4 (CH_3), 25.3 (CH_2), 25.2 (CH_2); MS (ESI) *m/z* 382 [(M + Na)$^+$, 24], 360 [(M + H)$^+$, 99], 275 [(M + H_3 – N – $O^tBu)^+$, 100], 242 [(M + H_2 – $CMe_2Ph)^+$, 24]; HRMS (ESI) *m/z* $C_{19}H_{30}N_5O_2$ ([M + H]$^+$) calcd for 360.2394, found 360.2419.

***tert*-Butyl**

***N*-methyl-*N*-{4-[2-(2-phenylpropan-2-yl)-2*H*-1,2,3,4-tetrazol-5-yl]butyl}carbamate 33**

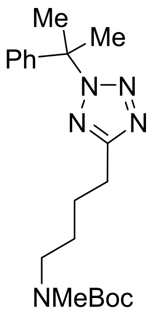

33

Using general procedure C, NaH (40 mg of a 60% suspension in mineral oil, 1.0 mmol, 2.0 eq) and tetrazole **ST22** (165 mg, 0.5 mmol, 1.0 eq) in THF (1 mL) then MeI (0.05 mL, 106 mg, 0.75 mmol, 1.5 eq) gave the crude product. Purification by flash column chromatography on silica with 70:30 petroleum ether-EtOAc as eluent gave tetrazole **33** (137 mg, 73%) as a colourless oil, R_F (70:30 petroleum ether-EtOAc) 0.2; IR (ATR) 2975, 2932, 1866, 1689, 1601, 1496, 1449, 1424, 1392, 1365, 1310, 1217, 1159, 1133, 1072, 1030, 1019, 975, 910 cm^{-1}; ^{1}H NMR (400 MHz, $CDCl_3$) δ 7.32–7.24 (m, 3H, Ph), 7.08–7.05 (m, 2H, Ph), 3.22 (br t, *J* = 6.5 Hz, 2H, NCH_2), 2.90 (t, *J* = 7.5 Hz, 2H, $TzCH_2$), 2.79 (br s, 3H, NMe), 2.14 (s, 6H, CMe_2), 1.76 (tt, *J* = 7.5, 7.5 Hz, 2H, CH_2), 1.56 (tt, *J* = 7.5, 7.0 Hz, 2H, CH_2), 1.43 (s, 9H, CMe_3); ^{13}C NMR (101 MHz, $CDCl_3$) (mixture of rotamers) δ 166.2 (C), 155.8 (C), 144.0 (C), 128.6 (CH), 127.8 (CH), 124.7 (CH), 79.2 (C), 67.9 (C), 48.3 (CH_3), 48.0 (CH_2), 34.0 (CH_2), 33.9 (CH_2), 29.1 (CH_3), 28.5 (CH_3), 27.4 (CH_2), 27.1 (CH_2), 25.3 (CH_2); MS (ESI) *m/z* 396 [(M + Na)$^+$, 4], 374 [(M + H)$^+$, 100], 318 [(M + H_2 – ^{t}Bu)$^+$,6], 275 [(M + H_3 – N – Me – O^{t}Bu)$^+$, 1], 256 [(M + H_2 – CMe_2Ph)$^+$, 4], 200 [(M + H_3 – CMe_2Ph – ^{t}Bu)$^+$, 2], 156 [(M + H_3 – CMe_2Ph – Boc)$^+$, 2]; HRMS (ESI) *m/z* $C_{20}H_{32}N_5O_2$ ([M + H]$^+$) calcd for 374.2551, found 374.2574.

Methyl({4-[2-(2-phenylpropan-2-yl)-2*H*-1,2,3,4-tetrazol-5-yl]butyl})(triphenylmethyl) amine ST24

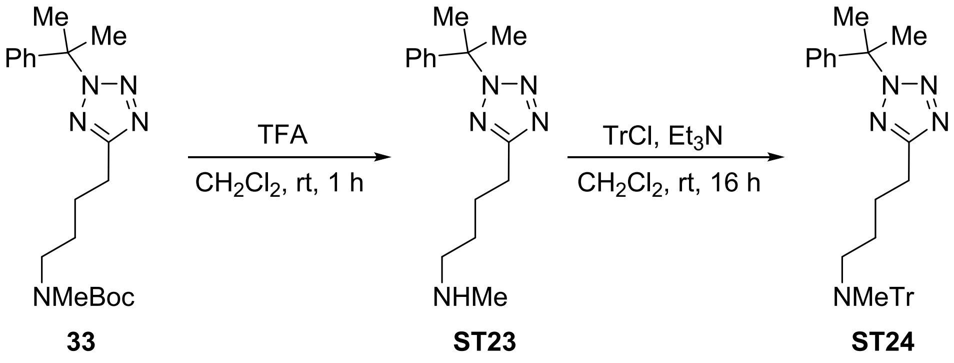

TFA (3.83 mL, 5.70 g, 50 mmol, 10 eq) was added into a stirred solution of tetrazole **33** (1.87 g, 5.0 mmol, 1.0 eq) im CH_2Cl_2 (10 mL) at 0 °C and the resulting solution was allowed to warm to rt and stirred for 1 h. 1 M $NaOH_{(aq)}$ (20 mL) was added and the two layers were separated, extracting the aqueous with CH_2Cl_2 (3 × 10 mL) The combined organic layers were dried (Na_2SO_4) and evaporated under reduced pressure to give crude tetrazole **ST23** as a colourless oil which was used in the next step without further purification, ^{1}H NMR (300 MHz, $CDCl_3$) δ 7.40–7.21 (m, 3H, Ph), 7.14–7.05 (m, 2H, Ph), 2.90 (t, *J* = 7.5 Hz, 2H, $TzCH_2$), 2.6 (t, *J* = 7.5 Hz, 2H, NCH_2), 2.41 (s, 3H, NMe), 2.14 (s, 6H, CMe_2), 1.82 (tt, *J* = 7.5, 7.5 Hz, 2H, CH_2), 1.57 (tt, *J* = 7.5, 7.0 Hz, 2H, CH_2), 1.46 (br s, 1H, NH).

Trityl chloride (1.39 g, 5 mmol, 1.0 eq) was added to a solution of tetrazole **ST23** (max, 5 mmol, 1.0 eq) and Et_3N (1.05 mL, 759 mg, 7.5 mmol, 1.5 eq) in CH_2Cl_2 (25 mL) at 0 °C. The resulting solution was warmed to rt and stirred for 16 h. Water (25 mL) was added and the two layers were separated. Extracting the aqueous with CH_2Cl_2 (3 × 20 mL) and the combined organic layers were dried ($MgSO_4$) and evaporated under reduced pressure to give the crude product. The excess triphenylmethanol from the crude product was then removed *via* Kügelrohr short path distillation (230 °C/1 mmHg) and purification by flash column chromatography on silica with 90:10 petroleum ether-EtOAc as eluent gave tetrazole **ST24** (1.34 g, 52%) as a colourless viscous oil, R_F (90:10 petroleum ether-EtOAc) 0.2; IR (ATR) 3083, 3056, 3030, 2988, 2938, 2859, 2798, 1595, 1489, 1469, 1391, 1370 ,1309, 1256, 1210, 1184, 1123, 1108, 1083, 1073, 1031, 1018, 1005, 927, 905 cm^{-1}; ^{1}H NMR (400 MHz, $CDCl_3$) δ 7.51–7.42 (m, 6H, Ph), 7.35–7.18 (m, 9H, Ph), 7.18–7.09 (tt, *J* = 7.5, 2.0 Hz, 3H, Ph), 7.10–7.04 (m, 2H, Ph), 2.87 (t, *J* = 7.5 Hz, 2H, $TzCH_2$), 2.14 (s, 6H, CMe_2), 2.11 (t, *J* = 8.0 Hz, 2H, NCH_2), 2.00 (s, 3H, NMe), 1.79 (tt, *J* =

7.5, 7.5 Hz, 2H, CH_2), 1.68 (tt, *J* = 7.5, 7.0 Hz, 2H, CH_2); ^{13}C NMR (101 MHz, $CDCl_3$) δ 166.5 (C), 144.1 (C), 143.1 (C), 129.4 (CH), 128.6 (CH), 128.0 (CH), 127.8 (CH), 127.4 (CH), 124.7 (CH), 78.0 (C), 67.9 (C), 52.1 (CH_2), 37.1 (CH_3), 29.1 (CH_3), 27.83 (CH_2), 26.1 (CH_2), 25.7 (CH_2); MS (ESI) *m/z* 516 [$(M + H)^+$, 35], 243 [$(CPh_3)^+$, 100]; HRMS (ESI) *m/z* $C_{34}H_{38}N_5$ ($[M + H]^+$) calcd for 516.3122, found 516.3150.

5-(4-Methoxybutyl)-2-(2-phenylpropan-2-yl)-2*H*-1,2,3,4-tetrazole ST25

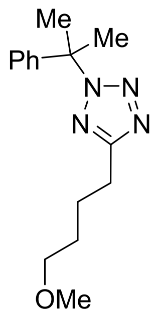

ST25

Using general procedure B, α-methylstyrene (1.08 mL, 973 mg, 8.23 mmol, 1.02 eq), tetrazole **ST7** (1.26 g, 8.07 mmol, 1.0 eq) and TFA (1.29 mL, 1.93 g, 16.9 mmol, 2.1 eq) in $CHCl_3$ (16 mL), gave the crude product. Purification by flash column chromatography on silica with 80:20 petroleum ether-EtOAc as eluent gave tetrazole **ST25** (2.07 g, 93%) as a colourless oil, R_F (80:20 petroleum ether-EtOAc) 0.2; IR (ATR) 2987, 2935, 2867, 2827, 1601, 1496, 1449, 1391, 1371, 1308, 1255, 1187, 1162, 1117, 1072, 1048, 1019, 967, 945, 925, 910 cm^{-1}; ^{1}H NMR (400 MHz, $CDCl_3$) δ 7.33–7.24 (m, 3H, Ph), 7.08–7.05 (m, 2H, Ph), 3.39 (t, *J* = 6.5 Hz, 2H, OCH_2), 3.31 (s, 3H, OMe), 2.90 (t, *J* = 7.5 Hz, 2H, $TzCH_2$), 2.14 (s, 6H, CMe_2), 1.88–1.81 (m, 2H, OCH_2CH_2), 1.68–1.59 (m, 2H, $TzCH_2CH_2$); ^{13}C NMR (101 MHz, $CDCl_3$) δ 166.3 (C), 144.0 (C), 128.6 (CH), 127.8 (CH), 124.7 (CH), 72.3 (CH_2), 67.9 (C), 58.5 (CH_3), 29.1 (CH_3), 29.0 (CH_2), 25.3 (CH_2), 24.8 (CH_2); MS (ESI) *m/z* 297 [$(M + Na)^+$, 60], 275 [$(M + H)^+$, 63], 157 [$(M + H_2 – CMe_2Ph)^+$, 100], 125 [$(M + H – CMe_2Ph – OMe)^+$, 30]; HRMS (ESI) *m/z* $C_{15}H_{23}N_4O$ ($[M + H]^+$) calcd for 275.1866, found 275.1871.

5-[2-(2-Methyl-1,3-dioxolan-2-yl)ethyl]-2-(2-phenylpropan-2-yl)-2*H*-1,2,3,4-tetrazole ST27

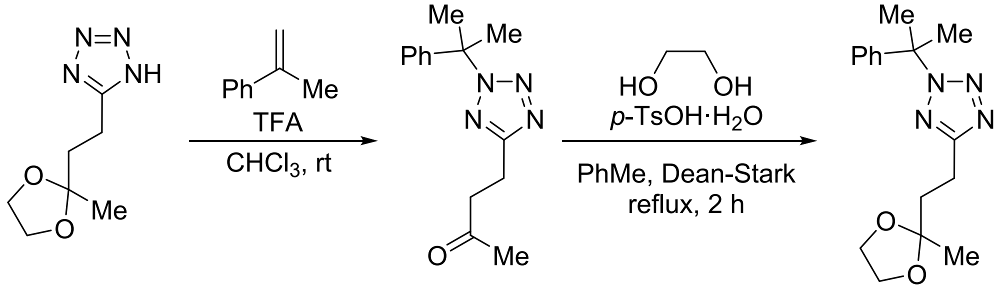

ST8 **ST26** **ST27**

Using general procedure B, α-methylstyrene (0.44 mL, 397 mg, 3.36 mmol, 1.02 eq), tetrazole **ST9** (608 mg, 3.30 mmol, 1.0 eq) and TFA (0.53 mL, 790 g, 6.92 mmol, 2.1 eq) in $CHCl_3$ (7 mL) gave crude tetrazole **ST26** as a yellow oil which was used in the next step without further purification. ^{1}H NMR (300 MHz, $CDCl_3$) 7.35–7.22 (m, 3H, Ph), 7.10–7.04 (m, 2H, Ph), 3.16 (t, J = 7.0 Hz, 2H, $TzCH_2$), 2.97 (t, J = 7.0 Hz, 2H, CCH_2), 2.19 (s, 3H, OCMe), 2.13 (s, 6H, CMe_2).

Ethylene glycol (0.37 mL, 411 mg, 6.6 mmol, 2.0 eq) was added into a solution of **ST26** (max, 3.30 mmol, 1.0 eq), *p*-toluenesulfonic acid monohydrate (63 mg, 0.33 mmol, 10 mol%) and in PhMe (10 mL). The resulting solution was stirred and heated at reflux under Dean-Stark trap for 2 h. After cooling to rt, the resulting solution was washed with saturated $NaHCO_{3(aq)}$ (50 mL), water (50 mL), brine (50 mL) and dried ($MgSO_4$) then evaporated under reduced pressure to give the crude product. Purification by flash column chromatography on silica with 70:30 petroleum ether-EtOAc as eluent gave tetrazole **ST27** (859 mg, 86%) as a colourless oil, R_F (70:30 petroleum ether-EtOAc) 0.2; IR (ATR) 2984, 2881, 1497, 1448, 1371, 1309, 1252, 1220, 1186, 1162, 1139, 1099, 1072, 1054, 1019, 948, 862 cm^{-1}; ^{1}H NMR (400 MHz, $CDCl_3$) δ 7.33–7.24 (m, 3H, Ph), 7.08–7.05 (m, 2H, Ph), 3.97–3.89 (m, 4H, OCH_2), 2.99 (t, J = 8.0 Hz, 2H, $TzCH_2$), 2.16 (t, J = 8.0 Hz, 2H, CCH_2), 2.14 (s, 6H, CMe_2), 1.36 (s, 3H, OCMe); ^{13}C NMR (101 MHz, $CDCl_3$) δ 166.3 (C), 144.1 (C), 128.6 (CH), 127.8 (CH), 124.7 (CH), 109.3 (C), 67.9 (C), 64.8 (CH_2), 37.0 (CH_2), 29.1 (CH_3), 24.0 (CH_3), 20.4 (CH_2)); MS (ESI) *m/z* 325 [$(M + Na)^+$, 25], 303 [$(M + H)^+$, 100], 198 [$(M + H – Ph – N_2)^+$, 11], 185 [$(M + H_2 – CMe_2Ph)^+$, 50], 157 [$(M + H_5 – CMe_2Ph – OCMe)^+$, 50], 141 [$(M + H – CMe_2Ph – H_2COCMe)^+$, 6], 118 [$(M + H_4 – CMe_2Ph – N_3 – CH_2CH_2)^+$, 12]; HRMS (ESI) *m/z* $C_{16}H_{23}N_4O_2$ ($[M + H]^+$) calcd for 303.1816, found 303.1839.

5-Benzyl-2-(2-phenylpropan-2-yl)-2*H*-1,2,3,4-tetrazole ST28

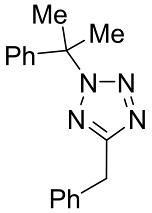

ST28

Using general procedure B, α-methylstyrene (0.66 mL, 603 mg, 5.1 mmol, 1.02 eq), tetrazole **ST8** (801 mg, 5 mmol, 1.0 eq) and TFA (0.88 mL, 1.31 g, 11.5 mmol, 2.1 eq) in $CHCl_3$ (10 mL), gave the crude product. Purification by flash column chromatography on silica with 90:10 petroleum ether-EtOAc as eluent gave tetrazole **ST28** (1.32 g, 95%) as a colourless oil, R_F (90:10 petroleum ether-EtOAc) 0.2; IR (ATR) 3062, 3030, 2988, 2939, 1602, 1496, 1468, 1449, 1391, 1371, 1308, 1253, 1189, 1160, 1106, 1070, 1021, 1002, 925, 910 cm^{-1}; ^1H NMR (400 MHz, $CDCl_3$) δ 7.36–7.18 (m, 8H, Ph), 7.12 – 7.05 (m, 2H, Ph), 4.24 (s, 2H, $TzCH_2$), 2.14 (s, 6H, CMe_2); ^{13}C NMR (101 MHz, $CDCl_3$) δ 165.1 (C), 144.0 (C), 137.0 (C), 128.8 (CH), 128.6 (2 × CH), 127.8 (CH), 126.7 (CH), 124.7 (CH), 68.1 (C), 31.9 (CH_2), 29.1 (CH_3). Spectroscopic data consistent with those reported in the literature. WO 2005075455 A2

***tert*-Butyl *N*-{3-[2-(2-phenylpropan-2-yl)-2*H*-1,2,3,4-tetrazol-5-yl]propyl}carbamate ST31**

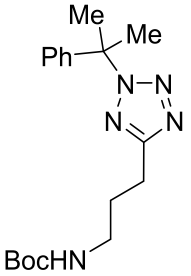

ST31

Using general procedure B, α-methylstyrene (1.30 mL, 1.19 g, 10.0 mmol, 1.02 eq), tetrazole **ST13** (2.17 g, 9.55 mmol, 1.0 eq) and TFA (1.53 mL, 2.28 g, 20.0 mmol, 2.1 eq) in $CHCl_3$ (19 mL), gave the crude product. Purification by flash column chromatography on silica with 70:30 petroleum ether-EtOAc as eluent gave tetrazole **ST31** (2.04 g, 62%) as a colourless oil, R_F (70:30 petroleum ether-EtOAc) 0.2; IR (ATR) 3350 (N-H str), 2977, 2871, 1693 (C=O str), 1601, 1497, 1449, 1391, 1365, 1305, 1248, 1163, 1074, 1020, 1000, 969, 925, 910 cm^{-1}; ^1H NMR (400 MHz, $CDCl_3$) δ 7.37–7.19 (m, 3H, Ph), 7.11–7.03 (m, 2H, Ph), 4.70 (br s, 1H, NH), 3.19 (dt, *J* =

6.5, 7.5 Hz, 2H, NCH_2), 2.92 (t, *J* = 7.5 Hz, 2H, $TzCH_2$), 2.14 (s, 6H, CMe_2), 1.96 (tt, *J* = 7.5, 7.5 Hz, 2H), 1.43 (s, 9H, CMe_3); ^{13}C NMR (101 MHz, $CDCl_3$) δ 165.8 (C), 155.9 (C), 143.9 (C), 128.7 (CH), 127.9 (CH), 124.7 (CH), 79.2 (C), 68.0 (C), 39.8 (CH_2), 29.1 (CH_3), 28.4 (CH_3), 28.27 (CH_2), 22.9 (CH_2); MS (ESI) *m/z* 346 [$(M + Na)^+$, 100], 322 [$(M + H)^+$, 26], 290 [$(M + H_2 - {}^tBu)^+$, 17], 228 [$(M + H_2 - CMe_2Ph)^+$, 20], 172 [$(M + H_3 - CMe_2Ph - {}^tBu)^+$, 24], 128 [$(M + H_3 - CMe_2Ph - Boc)^+$, 5]; HRMS (ESI) *m/z* $C_{18}H_{28}N_5O_2$ ($[M + H]^+$) calcd for 346.2238, found 346.2252.

***tert*-Butyl *N*-{4-[2-(2-phenylpropan-2-yl)-2*H*-1,2,3,4-tetrazol-5-yl]penatyl}carbamate ST32**

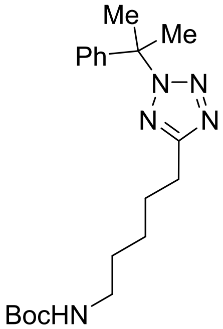

ST32

Using general procedure B, α-methylstyrene (1.63 mL, 1.48 g, 12.5 mmol, 1.02 eq), tetrazole **ST14** (3.03 g, 11.9 mmol, 1.0 eq) and TFA (1.91 mL, 2.85 g, 25 mmol, 2.1 eq) in $CHCl_3$ (24 mL), gave the crude product. Purification by flash column chromatography on silica with 70:30 petroleum ether-EtOAc as eluent gave tetrazole **ST32** (2.73 g, 61%) as a colourless oil, R_F (70:30 petroleum ether-EtOAc) 0.2; IR (ATR) 3350 (N-H str), 2977, 2933, 2863, 1694 (C=O str), 1497, 1449, 1391, 1365, 1308, 1248, 1164, 1106, 1073, 1019, 943, 910 cm^{-1}; ^1H NMR (400 MHz, $CDCl_3$) δ 7.35–7.23 (m, 3H, Ph), 7.10–7.03 (m, 2H, Ph), 4.49 (br s, 1H, NH), 3.09 (td, *J* = 7.5, 6.5 Hz, 2H, NCH_2), 2.87 (t, *J* = 7.5 Hz, 2H, $TzCH_2$), 2.14 (s, 6H, CMe_2), 1.79 (tt, *J* = 7.5, 7.5 Hz, 2H, CH_2), 1.50 (tt, *J* = 7.5, 7.5 Hz, 2H, CH_2), 1.43 (s, 9H, CMe_3), 1.37 (tt, *J* = 7.5, 7.5 Hz, 2H, CH_2); ^{13}C NMR (101 MHz, $CDCl_3$) δ 166.3 (C), 156.0 (C), 144.0 (C), 128.6 (CH), 127.8 (CH), 124.7 (CH), 79.0 (C), 67.9 (C), 40.4 (CH_2), 29.7 (CH_2), 29.1 (CH_3), 28.4 (CH_3), 27.7 (CH_2), 26.3 (CH_2), 25.4 (CH_2); MS (ESI) *m/z* 396 [$(M + Na)^+$, 41], 374 [$(M + H)^+$, 100], 318 [$(M + H_2 - {}^tBu)^+$, 19], 256 [$(M + H_2 - CMe_2Ph)^+$, 15], 200 [$(M + H_3 - CMe_2Ph - {}^tBu)^+$, 19], 128 [$(M + H_3 - CMe_2Ph - Boc)^+$, 10]; HRMS (ESI) *m/z* $C_{20}H_{32}N_5O_2$ ($[M + H]^+$) calcd for 374.2551, found 374.2555.

5-[2-(3-Chlorophenyl)ethyl]-2-(2-phenylpropan-2-yl)-2*H*-1,2,3,4-tetrazole 29

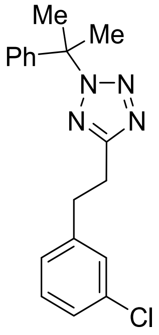

29

Using general procedure B, α-methylstyrene (1.31 mL, 1.44 g, 11.1 mmol, 1.02 eq), tetrazole **ST5** (2.27 g, 10.9 mmol, 1.0 eq) and TFA (1.75 mL, 2.61 g, 22.9 mmol, 2.1 eq) in $CHCl_3$ (20 mL), gave the crude product. Purification by flash column chromatography on silica with 85:15 petroleum ether-EtOAc as eluent gave tetrazole **29** (3.10 g, 87%) as a colourless oil, R_F (85:15 petroleum ether-EtOAc) 0.2; IR (ATR) 3062, 3028, 2988, 2939, 2867, 1599, 1573, 1497, 1478, 1448, 1391, 1370, 1308, 1255, 1187, 1094, 1077, 1073, 1022, 999, 930, 909 cm^{-1}; ^1H NMR (400 MHz, $CDCl_3$) δ 7.33–7.24 (m, 3H, Ph), 7.17–7.16 (m, 3H, Ar), 7.07–7.01 (m, 3H, Ph, Ar), 3.19 (m, 2H, $ArCH_2$), 3.08 (m, 2H, $TzCH_2$); ^{13}C NMR (101 MHz, $CDCl_3$) δ 166.3 (C), 144.0 (C), 141.6 (C), 128.6 (2 × CH), 128.4 (CH), 127.8 (CH), 125.9 (CH), 124.7 (CH), 67.9 (C), 35.2 (CH_2), 29.8 (CH_2), 29.1 (CH_3), 25.1 (CH_2). 165.3 (C), 143.9 (C), 142.5 (C), 134.1 (C), 129.7 (C), 128.7 (2 × CH), 127.8 (CH), 126.7 (CH), 126.4 (CH), 124.7 (CH), 68.1 (C), 33.7 (CH_2), 29.1 (CH_3), 27.1 (CH_2); MS (ESI) *m/z* 349 [$(M + Na)^+$, 28], 327 [$(M + H)^+$, 100], 209 [$(M + H_2 – CMe_2Ph)^+$, 53]; HRMS (ESI) *m/z* $C_{18}H_{19}ClN_4$ ($[M + H]^+$) calcd for 327.1371, found 327.1378.

***tert*-Butyl**

***N*-methyl-*N*-{4-[2-(2-phenylpropan-2-yl)-2*H*-1,2,3,4-tetrazol-5-yl]propyl}carbamate 32**

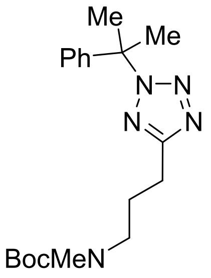

32

Using general procedure C, NaH (432 mg of a 60% suspension in mineral oil, 10.8 mmol, 2.0 eq) and tetrazole **ST31** (1.92 g, 5.4 mmol, 1.0 eq) in THF (11 mL) then MeI (0.4 mL, 920 mg, 6.48 mmol, 1.5 eq) gave the crude product. Purification by flash column chromatography on silica with 70:30 petroleum ether-EtOAc as eluent gave tetrazole **32** (2.04 g, 100%) as a colourless oil, R_F (70:30 petroleum ether-EtOAc) 0.2; IR (ATR) 2976, 2933, 1690 (C=O str), 1602, 1496, 1449, 1391, 1365, 1304, 1250, 1223, 1161, 1134, 1072, 1049, 1019, 970, 924, 910 cm^{-1}; ^1H NMR (400 MHz, $CDCl_3$) δ 7.35–7.22 (m, 3H, Ph), 7.12–7.04 (m, 2H, Ph), 3.28 (br s, 2H, NCH_2), 2.87 (t, *J* = 7.5 Hz, 2H, $TzCH_2$), 2.14 (s, 6H, CMe_2), 1.99 (tt, *J* = 7.5, 7.5 Hz, 2H, $CH_2CH_2CH_2$), 1.43 (s, 9H, CMe_3); ^{13}C NMR (101 MHz, $CDCl_3$) δ 166.0 (C), 155.8 (C), 143.9 (C), 128.6 (CH) , 127.8 (CH), 124.7 (CH), 79.3 (C), 68.0 (C), 48.3 (CH_3), 34.2 (CH_2), 29.1 (CH_3), 28.5 (CH_3), 26.3 (CH_2), 23.0 (CH_2); MS (ESI) *m/z* 382 [(M + Na)$^+$, 50], 360 [(M + H)$^+$, 100], 304 [(M + H_2 – ^{t}Bu)$^+$, 15], 242 [(M + H_2 – CMe_2Ph)$^+$, 15], 186 [(M + H_3 – CMe_2Ph –^{t}Bu)$^+$, 23], 142 [(M + H_3 – CMe_2Ph – Boc)$^+$, 19]; HRMS (ESI) *m/z* $C_{19}H_{30}N_5O_2$ ([M + H]$^+$) calcd for 360.2394, found 360.2395.

***tert*-Butyl**

***N*-methyl-*N*-{4-[2-(2-phenylpropan-2-yl)-2*H*-1,2,3,4-tetrazol-5-yl]pentyl}carbamate 34**

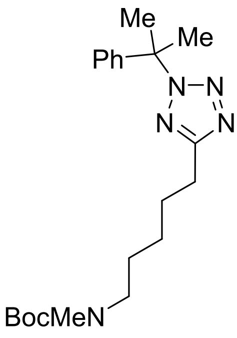

34

Using general procedure C, NaH (432 mg of a 60% suspension in mineral oil, 10.8 mmol, 2.0 eq) and tetrazole **ST32** (1.92 g, 5.4 mmol, 1.0 eq) in THF (11 mL) then MeI (0.4 mL, 920 mg, 6.48 mmol, 1.5 eq) gave the crude product. Purification by flash column chromatography on silica with 70:30 petroleum ether-EtOAc as eluent gave tetrazole **34** (2.06 g, 74%) as a colourless oil, R_F (70:30 petroleum ether-EtOAc) 0.2; IR (ATR) 2975, 2932, 2863, 1690 (C=O str), 1496, 1449, 1392, 1365, 1309, 1251, 1215, 1158, 1072, 1019, 924, 910 cm^{-1}; ^1H NMR (400 MHz, $CDCl_3$) δ 7.37–7.22 (m, 3H, Ph), 7.09–7.04 (m, 2H, Ph), 3.17 (t, *J* = 6.5 Hz, 2H, NCH_2), 2.87 (t, *J* = 7.5 Hz, 2H, $TzCH_2$), 2.81 (s, 3H, NMe), 2.14 (s, 6H, CMe_2), 1.80 (tt, *J* = 7.5, 7.5 Hz, 2H, CH_2), 1.54 (tt, *J* = 7.5 Hz, 3H, CH_2), 1.43 (s, 9H, CMe_3), 1.34 (tt, *J* = 7.5 Hz, 2H, CH_2); ^{13}C NMR (101 MHz, $CDCl_3$) δ 166.4 (C), 155.8 (C), 144.0 (C), 128.6 (CH), 127.8 (CH), 124.7 (CH), 79.1 (C), 67.9 (C), 48.4 (CH_3), 34.1 (CH_2), 29.3 (CH_2), 29.1 (CH_3), 28.5 (CH_3), 27.9 (CH_2), 26.2 (CH_2), 25.5 (CH_2); MS (ESI) *m/z* 410 [(M + Na)$^+$, 40], 388 [(M + H)$^+$, 100], 348 [(M + H_5 – Me_3)$^+$, 8], 331 [(M + H_2 – ^{t}Bu)$^+$, 10], 288 [(M + H_2 – Boc)$^+$, 5], 270 [(M + H_2 – CMe_2Ph)$^+$, 10], 226 [(M + H_5 – CMe_2Ph – Me)$^+$, 5], 214 [(M + H_3 – CMe_2Ph – ^{t}Bu)$^+$, 5], 170 [(M + H_3 – CMe_2Ph – Boc)$^+$, 10]; HRMS (ESI) *m/z* $C_{21}H_{34}N_5O_2$ ([M + H]$^+$) calcd for 388.2707, found 388.2714.

Attempted synthesis of 2-Methyl-3-[2-(triphenylmethyl)-2*H*-1,2,3,4-tetrazol-5-yl]pentan-2-ol 2

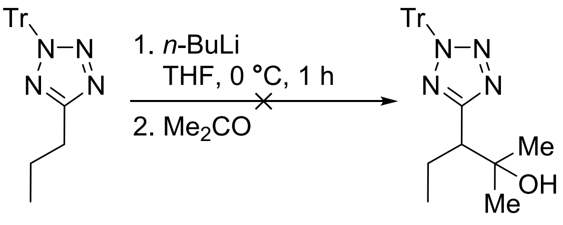

1 **2**

Using general procedure C, *n*-BuLi (0.6 mL of a 2.5 M solution in hexanes, 1.5 mmol, 1.5 eq) and **1** (354 mg, 1.0 mmol, 1.0 eq) in THF (7 mL) at 0 °C for 1 hour then Me_2CO (0.15 mL, 116 mg, 2.0 mmol, 2.0 eq) gave the crude product. Only starting **1** was observed from the ^{1}H NMR spectrum of the crude mixture and therefore further purifications were not attempted.

Attempted synthesis of 2-Methyl-3-[2-(triphenylmethyl)-2*H*-1,2,3,4-tetrazol-5-yl]pentan-2-ol 2

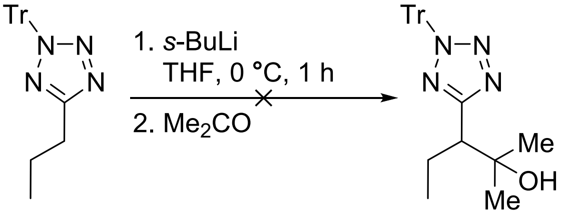

1 **2**

Using general procedure C, *s*-BuLi (1.15 mL of a 1.3 M solution in cyclohexanes/hexanes, 1.5 mmol, 1.5 eq) and **1** (354 mg, 1.0 mmol, 1.0 eq) in THF (7 mL) at 0 °C for 1 hour then Me_2CO (0.15 mL, 116 mg, 2.0 mmol, 2.0 eq) gave the crude product. ^{1}H NMR spectrum of the crude mixture revealed a complex mixture with no identifiable products and therefore further purifications were not attempted.

2-Methyl-3-[2-(triphenylmethyl)-2*H*-1,2,3,4-tetrazol-5-yl]pentan-2-ol 2

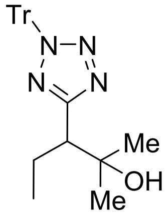

2

Using general procedure C, *s*-BuLi (1.2 mL of a 1.3 M solution in cyclohexane/hexanes, 1.56 mmol, 1.6 eq) and **1** (354 mg, 1.0 mmol, 1.0 eq) in THF (7 mL) at –78 °C for 30 min then Me_2CO (0.15 mL, 116 mg, 2.0 mmol, 2.0 eq) gave the crude product. Purification by flash column chromatography on silica with 70:30 petroleum ether-EtOAc as eluent gave tetrazole **2** (346 mg, 84%) as a colourless oil, R_F (70:30 petroleum ether-EtOAc) 0.2; IR (ATR) 3400 (O-H str), 3064, 2960, 2931, 2874, 1488, 1445, 1404, 1375, 1361, 1341, 1327, 1303, 1271, 1192, 1184, 1143, 1085, 1070, 1035, 1018, 1001, 971, 931, 906 cm^{-1}; ^1H NMR (400 MHz, $CDCl_3$) δ 7.39–7.29 (m, 9H, Ph), 7.16–7.09 (m, 6H, Ph), 3.01 (dd, *J* = 10.5, 4.5 Hz, 1H, TzCH), 2.53 (s, 1H, OH), 2.02–1.84 (m, 2H, CH_2), 1.21 (s, 3H, HOC$Me_A$$Me_B$), 1.11 (s, 3H, HOC$Me_A$$Me_B$), 0.75 (t, *J* = 7.5Hz, 3H, *Me*CH_2); ^{13}C NMR (101 MHz, $CDCl_3$) δ 166.6 (C), 141.3 (C), 130.1 (CH), 128.4 (CH), 127.8 (CH), 82.9 (C), 72.1 (C), 50.0 (CH), 28.8 (CH_3), 27.0 (CH_3), 21.9 (CH_2), 12.4 (CH_3); MS (ESI) *m/z* 435 [(M + Na)$^+$, 24], 243 [(Ph_3C)$^+$, 100]; HRMS (ESI) *m/z* $C_{26}H_{28}N_4ONa$ ([M + Na]$^+$) calcd for 435.2155, found 435.2155.

Attempted synthesis of 3-[2-(1,1-Diphenylethyl)-2*H*-1,2,3,4-tetrazol-5-yl]-2-methylpentan-2-ol 4

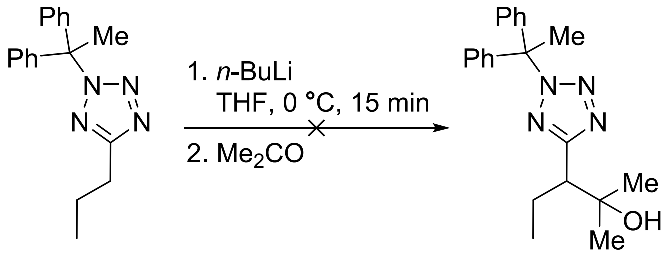

3 **4**

Using general procedure C, *n*-BuLi (0.46 mL of a 2.5 M solution in hexanes, 1.15 mmol, 2.3 eq) and **3** (146 mg, 1.0 mmol, 1.0 eq) in THF (7 mL) at 0 °C for 15 min then Me_2CO (0.07 mL, 58 mg, 1.0 mmol, 2.0 eq) gave the crude product. ^1H NMR spectrum of the crude mixture

revealed a complex mixture with no identifiable products and therefore further purifications were not attempted.

Attempted synthesis of 3-[2-(1,1-Diphenylethyl)-2*H*-1,2,3,4-tetrazol-5-yl]-2-methylpentan-2-ol 4

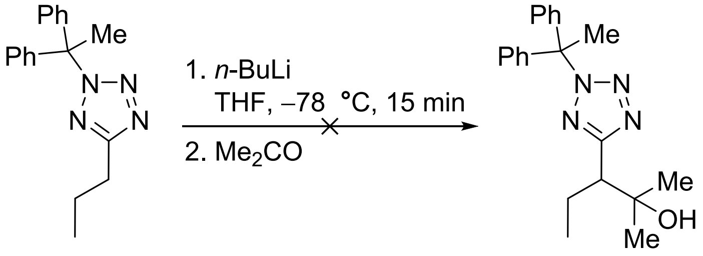

3 **4**

Using general procedure C, *n*-BuLi (0.46 mL of a 2.5 M solution in hexanes, 1.15 mmol, 2.3 eq) and **3** (146 mg, 1.0 mmol, 1.0 eq) in THF (7 mL) at –78 °C for 15 min then Me_2CO (0.07 mL, 58 mg, 1.0 mmol, 2.0 eq) gave the crude product. Only starting **3** was observed from the ^1H NMR spectrum of the crude mixture and therefore further purifications were not attempted.

3-[2-(1,1-Diphenylethyl)-2*H*-1,2,3,4-tetrazol-5-yl]-2-methylpentan-2-ol 4

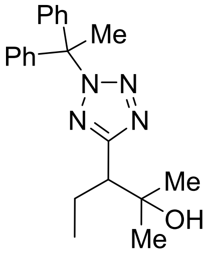

4

Using general procedure C, *s*-BuLi (0.6 mL of a 1.3 M solution in cyclohexane/hexanes, 0.79 mmol, 1.6 eq) and **2** (146 mg, 0.5 mmol, 1.0 eq) in THF (4 mL) at –78 °C for 15 min then Me_2CO (0.07 mL, 58 mg, 1.0 mmol, 2.0 eq) gave the crude product. Purification by flash column chromatography on silica with 70:30 petroleum ether-EtOAc as eluent gave tetrazole **4** (138 mg, 79%) as a colourless oil, R_F (70:30 petroleum ether-EtOAc) 0.2; IR (ATR) 3452 (O-H str), 3014, 2948, 2958, 2889, 1456, 1433, 1401, 1388, 1354, 1337, 1320, 1300, 1244, 1163, 1174, 1124, 1071, 1063, 1025, 1007, 1002, 989, 914, 901 cm^{-1}; ^1H NMR (400 MHz, $CDCl_3$) δ 7.36–7.29 (m, 6H, Ph), 7.12–7.05 (m, 4H, Ph), 2.98 (dd, *J* = 10.5, 4.5 Hz, 1H, TzCH), 2.59 (s, 3H, Ph_2CMe), 2.54 (s, 1H, OH), 1.97–1.84 (m, 2H, CH_2), 1.21 (s, 3H, $HOCMe_AMe_B$), 1.11 (s, 3H,

HOCMe$_A$*Me*$_B$), 0.74 (t, *J* = 7.5 Hz, 3H, *Me*CH$_2$); 13C NMR (101 MHz, CDCl3) δ 167.0 (C), 142.9 (C), 142.8 (C), 128.3 (2 × CH), 128.2 (2 × CH), 127.4 (CH), 127.3 (CH), 74.3 (C), 72.1 (C), 50.0 (CH), 29.4 (CH_3), 28.8 (CH_3), 27.0 (CH_3), 21.9 (CH_2), 12.5 (CH_3); MS (ESI) *m/z* 373 [(M + Na)$^+$, 6], 351 [(M + H)$^+$, 83], 181 [(Ph_2MeC)$^+$, 50], 171 [(M – Ph_2MeC)$^+$, 100]; HRMS (ESI) *m/z* $C_{21}H_{26}N_4O$ ([M + H]$^+$) calcd for 351.2179, found 351.2181.

Attempted synthesis of 2-Methyl-3-[2-(2-phenylpropan-2-yl)-2*H*-1,2,3,4-tetrazol-5-yl]pentan-2-ol 7

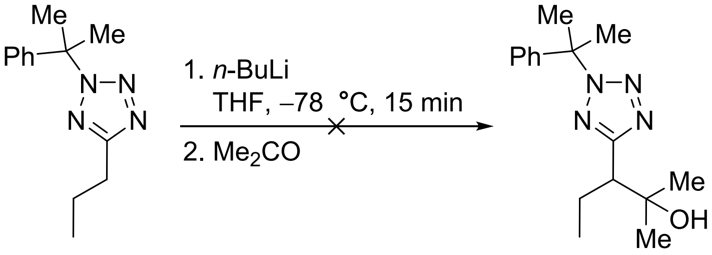

5 **7**

Using general procedure C, *n*-BuLi (0.46 mL of a 2.5 M solution in hexanes, 1.15 mmol, 2.3 eq) and **3** (146 mg, 1.0 mmol, 1.0 eq) in THF (7 mL) at –78 °C for 15 min then Me$_2$CO (0.07 mL, 58 mg, 1.0 mmol, 2.0 eq) gave the crude product. Only starting **5** was observed from the ^{1}H NMR spectrum of the crude mixture and therefore further purifications were not attempted.

2-Methyl-3-[2-(2-phenylpropan-2-yl)-2*H*-1,2,3,4-tetrazol-5-yl]pentan-2-ol 7

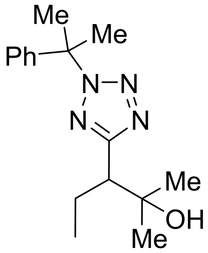

7

Using general procedure C, *n*-BuLi (0.6 mL of a 2.5 M solution in cyclohexane/hexanes, 1.5 mmol, 1.5 eq) and **5** (230 mg, 1.0 mmol, 1.0 eq) in THF (7 mL) at 0 °C for 1 hour then Me$_2$CO (0.15 mL, 116 mg, 2.0 mmol, 2.0 eq) gave the crude product. Purification by flash column chromatography on silica with 80:20 petroleum ether-EtOAc as eluent gave tetrazole **7** (181 mg, 63%) as a colourless oil, R$_F$ (80:20 petroleum ether-EtOAc) 0.2; IR (ATR) 3423 (O-H str), 2986, 2936, 2875, 1497, 1485, 1461, 1449, 1391, 1371, 1337, 1306, 1253, 1234, 1186, 1161,

1105, 1078, 1063, 1031, 1019, 1001, 983, 971, 930, 907 cm^{-1}; ^{1}H NMR (300 MHz, $CDCl_3$) δ 7.32–7.21 (m, 3H, Ph), 7.04–7.00 (m, 2H, Ph), 2.96 (dd, *J* = 10.0, 5.0 Hz, 1H, TzCH), 2.67 (br s, 1H, OH), 2.14 (s, 6H, PhCMe_2), 1.96–1.85 (m, 2H, MeCH_2), 1.20 (s, 3H, HOC$Me_A$$Me_B$), 1.11 (s, 3H, HOC$Me_A$$Me_B$), 0.71 (t, *J* = 7.5 Hz, 3H, *Me*CH_2); ^{13}C NMR (75.5 MHz, $CDCl_3$) δ 167.2 (C), 144.1 (C), 128.7 (CH), 127.9 (CH), 124.5 (CH), 72.1 (C), 68.3 (C), 50.0 (CH), 29.1 (2 × CH_3), 28.8 (CH_3), 27.0 (CH_3), 21.9 (CH_2), 12.5 (CH_3). MS (ESI) *m/z* 600 [(MM + Na)$^+$, 55], 577 [(MM + H)$^+$, 56], 311 [(M + Na)$^+$, 45], 289 [(M + H)$^+$, 100], 171 [(M + H_2 – CMe_2Ph)$^+$, 53], 153 [(M – CMe2OH – Ph)$^+$, 23]; HRMS (ESI) *m/z* $C_{16}H_{24}N_4O$ ([M + H]$^+$) calcd for 289.2021, found 289.2023.

Using general procedure C, *n*-BuLi (0.84 mL of a 2.5 M solution in cyclohexane/hexanes, 2.1 mmol, 2.1 eq) and **5** (230 mg, 1.0 mmol, 1.0 eq) in THF (7 mL) at 0 °C for 1 hour then Me_2CO (0.15 mL, 116 mg, 2.0 mmol, 2.0 eq) gave the crude product. Purification by flash column chromatography on silica with 80:20 petroleum ether-EtOAc as eluent gave tetrazole **7** (173 mg, 60%) as a colourless oil.

Using general procedure C, *n*-BuLi (0.84 mL of a 2.5 M solution in cyclohexane/hexanes, 2.1 mmol, 2.1 eq) and **5** (230 mg, 1.0 mmol, 1.0 eq) in THF (7 mL) at –30 °C for 1 hour then Me_2CO (0.15 mL, 116 mg, 2.0 mmol, 2.0 eq) gave the crude product. Purification by flash column chromatography on silica with 80:20 petroleum ether-EtOAc as eluent gave tetrazole **7** (219 mg, 76%) as a colourless oil.

Using general procedure C, *s*-BuLi (1.2 mL of a 1.3 M solution in cyclohexane/hexanes, 1.56 mmol, 1.6 eq) and **5** (230 mg, 1.0 mmol, 1.0 eq) in THF (7 mL) at –78 °C for 15 min then Me_2CO (0.15 mL, 116 mg, 2.0 mmol, 2.0 eq) gave the crude product. Purification by flash column chromatography on silica with 80:20 petroleum ether-EtOAc as eluent gave tetrazole **7** (279 mg, 97%) as a colourless oil.

Using general procedure F, 1.1 M solution of tetrazole **5** in 1:1 PhMe/TMEDA (0.5 mL, 0.55 mmol, 1.0 eq) and a solution of 2.5 M *n*-BuLi in hexanes (0.5 mL, 1.15 mmol, 2.3 eq) were mixed for 30 s at rt followed by mixing with a 2.5 M Me_2CO solution in PhMe gave the crude product. Purification by flash column chromatography on silica with 80:20 petroleum ether-EtOAc as eluent gave tetrazole **7** (151 mg, 95%) as a colourless oil.

Using general procedure F, 1.1 M solution of tetrazole **5** in 1:1 PhMe/TMEDA (25 mL, 27.2 mmol, 1.0 eq) and a solution of 2.5 M *n*-BuLi in hexanes (25 mL, 62.6 mmol, 2.3 eq) were

mixed for 30 s at rt followed by mixing with a 2.5 M allylBr solution in PhMe gave the crude product. Purification by flash column chromatography on silica with 80:20 petroleum ether-EtOAc as eluent gave tetrazole **7** (5.89 mg, 75%) as a colourless oil.

Attempted synthesis of 2-Methyl-3-[2-(2-phenylpropan-2-yl)-2*H*-1,2,3,4-tetrazol-5-yl]pentan-2-ol 7

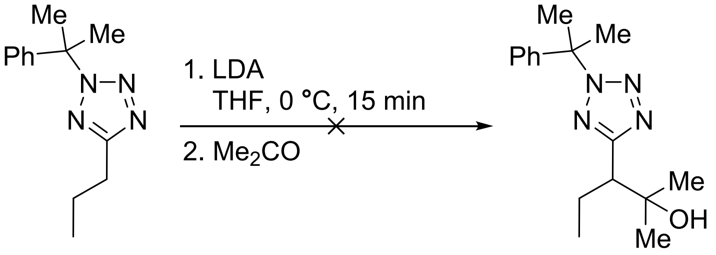

5 **7**

Using general procedure C, LDA (0.75 mL of a 1 M solution in THF, 0.75 mmol, 1.5 eq) and **3** (115 mg, 0.5 mmol, 1.0 eq) in THF (4 mL) at 0 °C for 15 min then Me_2CO (0.07 mL, 58 mg, 1.0 mmol, 2.0 eq) gave the crude product. Only starting **5** was observed from the ^1H NMR spectrum of the crude mixture and therefore further purifications were not attempted.

Attempted synthesis of 2-Methyl-3-[2-(2-phenylpropan-2-yl)-2*H*-1,2,3,4-tetrazol-5-yl]pentan-2-ol 7

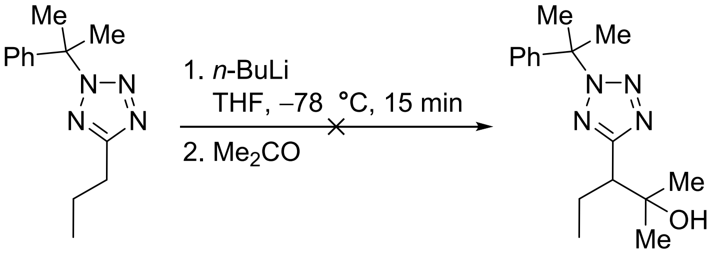

5 **7**

Using general procedure C, LDA (0.75 mL of a 1 M solution in THF, 0.75 mmol, 1.5 eq) and **3** (115 mg, 0.5 mmol, 1.0 eq) in THF (4 mL) at –78 °C for 15 min then Me_2CO (0.07 mL, 58 mg, 1.0 mmol, 2.0 eq) gave the crude product. Only starting **5** was observed from the ^1H NMR spectrum of the crude mixture and therefore further purifications were not attempted.

Attempted enantioselective lithiation-substitution of 5

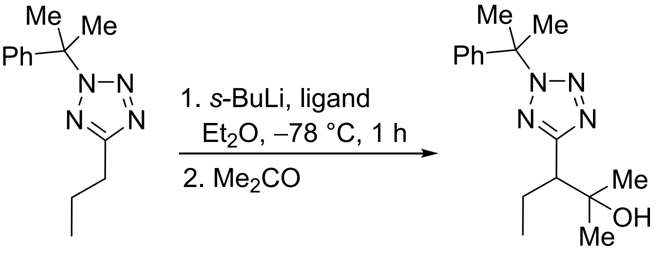

5 **7**

Entry	Ligand	Isolated yield	er
1	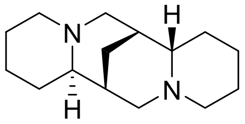 (+)-sparteine	41%	64:36
2	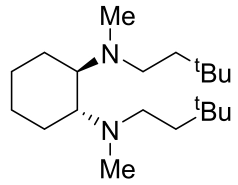 (*R*, *R*)-**L1**	94%	43:57
3	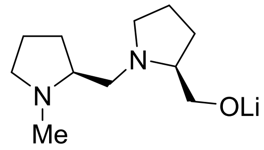 (*S*,*S*)-**L3**	88%	66:34
4	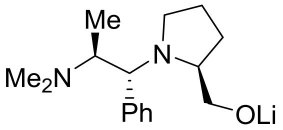 (*S*,*R*,*S*)-**L4**	97%	67:33
5	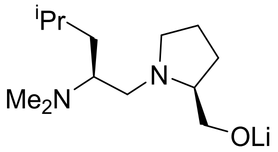 (*S*,*S*)-**L5**	56%	69:31
6	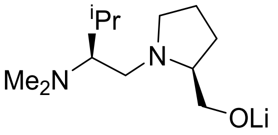 (*S*,*S*)-**L6**	21%	60:40
7	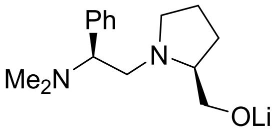 (*S*,*S*)-**L7**	80%	71:29
8	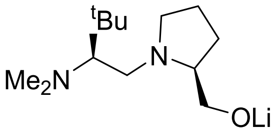 (*S*,*S*)-**L8**	52%	67:33

9	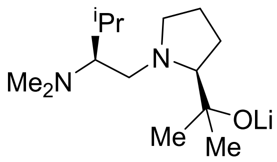	(*S*,*S*)-**L9**	27%	65:35

Using general procedure G, *s*-BuLi (0.6 mL of a 1.3 M solution in cyclohexane/hexane, 0.8 mmol, 1.6 eq), (+)-**L1** (0.21 mL, 211 mg, 0.9 mmol, 1.8 eq) and tetrazole **5** (115 mg, 0.5 mmol, 1.0 eq) in Et_2O (4 mL) at –78 °C for 1 hour then Me_2CO (0.07 mL, 58 mg, 1 mmol, 2.0 eq) gave the crude product. Purification by flash column chromatography on silica with 70:30 petroleum ether-EtOAc as eluent gave tetrazole **7** (59 mg, 41%, 64:36 er by CSP-HPLC) as a colourless oil, CSP-HPLC: Chiralpak IA (95:5 hexane-*i*-PrOH, 1.0 mLmin^{-1}) enantiomer 1: 14.3 min, enantiomer 2: 15.9 min.

Using general procedure G, *s*-BuLi (0.6 mL of a 1.3 M solution in cyclohexane/hexane, 0.8 mmol, 1.6 eq), (*R*,*R*)-**L2** (280 mg, 0.9 mmol, 1.8 eq) and tetrazole **5** (115 mg, 0.5 mmol, 1.0 eq) in Et_2O (4 mL) at –78 °C for 1 hour then Me_2CO (0.07 mL, 58 mg, 1 mmol, 2.0 eq) gave the crude product. Purification by flash column chromatography on silica with 70:30 petroleum ether-EtOAc as eluent gave tetrazole **7** (135 mg, 94%, 43:57 er by CSP-HPLC) as a colourless oil, CSP-HPLC: Chiralpak IA (95:5 hexane-*i*-PrOH, 1.0 mLmin^{-1}) enantiomer 1: 19.7 min, enantiomer 2: 20.4 min.

Using general procedure G, *s*-BuLi (1.2 mL of a 1.3 M solution in cyclohexane/hexane, 1.6 mmol, 3.2 eq), (*S*,*S*)-**L3** (178 mg, 0.9 mmol, 1.8 eq) and tetrazole **5** (115 mg, 0.5 mmol, 1.0 eq) in Et_2O (4 mL) at –78 °C for 1 hour then Me_2CO (0.07 mL, 58 mg, 1 mmol, 2.0 eq) gave the crude product. Purification by flash column chromatography on silica with 70:30 petroleum ether-EtOAc as eluent gave tetrazole **7** (127 mg, 88%, 66:34 er by CSP-HPLC) as a colourless oil, CSP-HPLC: Chiralpak IA (95:5 hexane-*i*-PrOH, 1.0 mLmin^{-1}) enantiomer 1: 15.4 min, enantiomer 2: 16.8 min.

Using general procedure G, *s*-BuLi (1.2 mL of a 1.3 M solution in cyclohexane/hexane, 1.6 mmol, 3.2 eq), (*S*,*R*,*S*)-**L4** (236 mg, 0.9 mmol, 1.8 eq) and tetrazole **5** (115 mg, 0.5 mmol, 1.0 eq) in Et_2O (4 mL) at –78 °C for 1 hour then Me_2CO (0.07 mL, 58 mg, 1 mmol, 2.0 eq) gave the crude product. Purification by flash column chromatography on silica with 70:30 petroleum ether-EtOAc as eluent gave tetrazole **7** (140 mg, 97%, 67:33 er by CSP-HPLC) as a colourless

oil, CSP-HPLC: Chiralpak IA (95:5 hexane-*i*-PrOH, 1.0 mLmin^{-1}) enantiomer 1: 16.7 min, enantiomer 2: 18.3 min.

Using general procedure G, *s*-BuLi (1.2 mL of a 1.3 M solution in cyclohexane/hexane, 1.6 mmol, 3.2 eq), (*S*,*S*)-**L5** (206 mg, 0.9 mmol, 1.8 eq) and tetrazole **5** (115 mg, 0.5 mmol, 1.0 eq) in Et_2O (4 mL) at –78 °C for 1 hour then Me_2CO (0.07 mL, 58 mg, 1 mmol, 2.0 eq) gave the crude product. Purification by flash column chromatography on silica with 70:30 petroleum ether-EtOAc as eluent gave tetrazole **7** (81 mg, 56%, 69:31 er by CSP-HPLC) as a colourless oil, CSP-HPLC: Chiralpak IA (95:5 hexane-*i*-PrOH, 1.0 mLmin^{-1}) enantiomer 1: 16.1 min, enantiomer 2: 17.7 min.

Using general procedure G, *s*-BuLi (1.2 mL of a 1.3 M solution in cyclohexane/hexane, 1.6 mmol, 3.2 eq), (*S*,*S*)-**L6** (193 mg, 0.9 mmol, 1.8 eq) and tetrazole **5** (115 mg, 0.5 mmol, 1.0 eq) in Et_2O (4 mL) at –78 °C for 1 hour then Me_2CO (0.07 mL, 58 mg, 1 mmol, 2.0 eq) gave the crude product. Purification by flash column chromatography on silica with 70:30 petroleum ether-EtOAc as eluent gave tetrazole **7** (31 mg, 21%, 60:40 er by CSP-HPLC) as a colourless oil, CSP-HPLC: Chiralpak IA (95:5 hexane-*i*-PrOH, 1.0 mLmin^{-1}) enantiomer 1: 18.2 min, enantiomer 2: 19.5 min.

Using general procedure G, *s*-BuLi (1.2 mL of a 1.3 M solution in cyclohexane/hexane, 1.6 mmol, 3.2 eq), (*S*,*S*)-**L7** (224 mg, 0.9 mmol, 1.8 eq) and tetrazole **5** (115 mg, 0.5 mmol, 1.0 eq) in Et_2O (4 mL) at –78 °C for 1 hour then Me_2CO (0.07 mL, 58 mg, 1 mmol, 2.0 eq) gave the crude product. Purification by flash column chromatography on silica with 70:30 petroleum ether-EtOAc as eluent gave tetrazole **7** (115 mg, 80%, 71:29 er by CSP-HPLC) as a colourless oil, CSP-HPLC: Chiralpak IA (95:5 hexane-*i*-PrOH, 1.0 mLmin^{-1}) enantiomer 1: 16.7 min, enantiomer 2: 18.3 min.

Using general procedure G, *s*-BuLi (1.2 mL of a 1.3 M solution in cyclohexane/hexane, 1.6 mmol, 3.2 eq), (*S*,*S*)-**L8** (mg, 0.9 mmol, 1.8 eq) and tetrazole **5** (206 mg, 0.5 mmol, 1.0 eq) in Et_2O (4 mL) at –78 °C for 1 hour then Me_2CO (0.07 mL, 58 mg, 1 mmol, 2.0 eq) gave the crude product. Purification by flash column chromatography on silica with 70:30 petroleum ether-EtOAc as eluent gave tetrazole **7** (75 mg, 52%, 67:33 er by CSP-HPLC) as a colourless oil, CSP-HPLC: Chiralpak IA (95:5 hexane-*i*-PrOH, 1.0 mLmin^{-1}) enantiomer 1: 17.2 min, enantiomer 2: 19.0 min.

Using general procedure G, *s*-BuLi (1.2 mL of a 1.3 M solution in cyclohexane/hexane, 1.6 mmol, 3.2 eq), (*S*,*S*)-**L9** (218 mg, 0.9 mmol, 1.8 eq) and tetrazole **5** (115 mg, 0.5 mmol, 1.0 eq) in Et_2O (4 mL) at –78 °C for 1 hour then Me_2CO (0.07 mL, 58 mg, 1 mmol, 2.0 eq) gave the crude product. Purification by flash column chromatography on silica with 70:30 petroleum ether-EtOAc as eluent gave tetrazole **7** (39 mg, 27%, 65:35 er by CSP-HPLC) as a colourless oil, CSP-HPLC: Chiralpak IA (95:5 hexane-*i*-PrOH, 1.0 mLmin^{-1}) enantiomer 1: 18.7 min, enantiomer 2: 19.5 min.

1,1-Diphenyl-2-[2-(2-phenylpropan-2-yl)-2*H*-1,2,3,4-tetrazol-5-yl]butan-1-ol 8

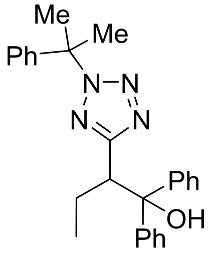

8

Using general procedure C, *n*-BuLi (0.3 mL of a 2.5 M solution in hexanes, 0.75 mmol, 1.5 eq) and **5** (115 mg, 0.5 mmol, 1.0 eq) in THF (4 mL) at 0 °C for 15 min then a solution of Ph_2CO (186 mg, 1.0 mmol, 2.0 eq) in THF (2 mL) gave the crude product. Purification by flash column chromatography on silica with 95:5 petroleum ether-EtOAc as eluent gave tetrazole **8** (141 mg, 68%) as a white solid., m.p. 80–83 °C; R_F (95:5 petroleum ether-EtOAc) 0.2; IR (ATR) 3428 (O-H str), 3060, 3035, 2994, 2965, 2931, 2874, 1596, 1493, 1446, 1394, 1382, 1372, 1321, 1272, 1250 ,1232, 1191, 1176, 1165, 1153, 1131, 1103, 1091, 1072, 1055, 1025, 1000, 975, 946, 920 cm^{-1}; ^{1}H NMR (300 MHz, $CDCl_3$) δ 7.63–7.59 (m, 2H, Ph), 7.46–7.42 (m, 2H, Ph), 7.35–7.28 (m, 2H, Ph), 7.23–7.02 (m, 7H, Ph), 6.62–6.57 (m, 2H, Ph), 4.73 (s, 1H, OH), 4.21 (dd, *J* = 11.0, 3.5 Hz, 1H, TzCH), 2.04 (s, 3H, $C\mathit{Me}_A Me_B$), 2.04 (s, 3H, $CMe_A \mathit{Me}_B$), 1.99–1.86 (m, 1H, $MeC\mathit{H}_A CH_B$), 1.75–1.62 (m, 1H, $MeCH_A C\mathit{H}_B$), 0.73 (t, *J* = 7.5 Hz, 3H, $\mathit{Me}CH_2$); ^{13}C NMR (75.5 MHz, $CDCl_3$) δ 167.3 (C), 147.1 (C), 144.8 (C), 144.1 (C), 128.7 (CH), 128.3 (CH), 128.0 (CH), 127.6 (CH), 126.5 (CH), 126.3 (CH), 125.7 (CH), 125.3 (CH), 124.2 (CH), 79.5 (C), 68.6 (C), 47.4 (CH), 29.3 (CH_3), 29.1 (CH_3), 22.8 (CH_2), 12.1 (CH_3); MS (ESI) *m/z* 435 [(M + Na)$^+$, 37], 413 [(M + H)$^+$, 100], 395 [(M – OH)$^+$, 80], 277 [(M + H_2 – CMe_2Ph – OH)$^+$, 27]; HRMS (ESI) *m/z* $C_{26}H_{28}N_4O$ ([M + H]$^+$) calcd for 413.2336, found 413.2320.

Using general procedure C, *n*-BuLi (0.46 mL of a 2.5 M solution in hexanes, 1.15 mmol, 2.3 eq) and **5** (115 mg, 0.5 mmol, 1.0 eq) in THF (4 mL) at 0 °C for 15 min then a solution of Ph_2CO (228 mg, 1.25 mmol, 2.5 eq) in THF (2 mL) gave the crude product. Purification by flash column chromatography on silica with 95:5 petroleum ether-EtOAc as eluent gave tetrazole **8** (176 mg, 85%) as a white solid.

Using general procedure F, 1.1 M solution of tetrazole **5** in 1:1 PhMe/TMEDA (0.5 mL, 0.55 mmol, 1.0 eq) and a solution of 2.5 M *n*-BuLi in hexanes (0.5 mL, 1.15 mmol, 2.3 eq) were mixed for 30 s at rt followed by mixing with a 2.5 M Ph_2CO solution in PhMe gave the crude product. Purification by flash column chromatography on silica with 95:5 petroleum ether-EtOAc as eluent gave tetrazole **8** (213 mg, 94%) as a white solid.

1-Phenyl-2-[2-(2-phenylpropan-2-yl)-2*H*-1,2,3,4-tetrazol-5-yl]butan-1-ol 9

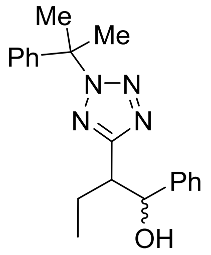

9

Using general procedure C, *n*-BuLi (0.46 mL of a 2.5 M solution in hexanes, 1.15 mmol, 2.3 eq) and **5** (115 mg, 0.5 mmol, 1.0 eq) in THF (4 mL) at 0 °C for 15 min then PhCHO (0.12 mL, 127 mg, 1.25 mmol, 2.5 eq) gave the crude product. Purification by flash column chromatography on silica with 80:20 petroleum ether-EtOAc as eluent gave a 60:40 diastereomeric mixture of tetrazole **9** (126 mg, 45% of major and 30% of minor) as a yellow oil, R_F (80:20 petroleum ether-EtOAc) 0.2; IR (ATR) 3359 (O-H str), 3062, 3033, 2967, 2933, 2876, 1601, 1496, 1455, 1446, 1393, 1371, 1318, 1295, 1280, 1250, 1216, 1201, 1167, 1104, 1076, 1047, 1027, 1001, 921 cm^{-1}; ^1H NMR (400 MHz, $CDCl_3$)(60:40 mixtures of diastereomers) δ 7.32–7.21 (m, 8H, Ph), 6.93–6.89 (m, 2H, Ph), 5.09–5.05 (m, 1H, OCH), 3.42–3.34 (m, 1H, TzCH), 3.04 (br s, 1H, OH), 2.13 (s, 2.4H, CMe_2), 2.11 (s, 1.8H, C$Me_A$$Me_B$), 2.10 (s, 1.8H, C$Me_A$$Me_B$), 1.98–1.76 (m, 2H, MeC$H_2$), 0.84 (t, J = 7.5 Hz, 1.2H, *Me*CH_2), 0.78 (t, J = 7.5 Hz, 1.8H, *Me*CH_2); ^{13}C NMR (101 MHz, $CDCl_3$) δ 168.9 (C), 144.4 (C), 135.9 (CH), 128.6 (CH), 127.7 (CH), 124.6 (CH), 116.9 (CH_2), 68.0 (C), 38.4 (CH), 38.3 (CH_2), 29.2 (2 × CH_3), 26.8 (CH_2), 11.6 (CH_3). 167.0 (C), 166.7 (C), 144.0

(C), 142.5 (C), 141.9(2 × C), 128.6 (2 × CH), 128.3 (CH), 128.1 (CH), 127.8 (CH), 127.5 (CH), 127.4 (CH), 126.4 (2 × CH), 126.1 (CH), 124.5 (2 × CH), 75.7 (CH), 75.5 (CH), 68.3 (C), 68.2 (C), 46.4 (2 × CH), 29.2 (CH_3), 29.1 (2 × CH_3), 24.7 (CH_2), 22.0 (CH_2), 11.7 (2 × CH_3); MS (ESI) *m/z* 359 [$(M + Na)^+$, 30], 337 [$(M + H)^+$, 100], 219 [$(M + H_2 - CMe_2Ph)^+$, 30]; HRMS (ESI) *m/z* $C_{20}H_{24}N_4O$ ($[M + H]^+$) calcd for 337.2023, found 337.2020.

Using general procedure F, 1.1 M solution of tetrazole **5** in 1:1 PhMe/TMEDA (0.5 mL, 0.55 mmol, 1.0 eq) and a solution of 2.5 M *n*-BuLi in hexanes (0.5 mL, 1.15 mmol, 2.3 eq) were mixed for 30 s at rt followed by mixing with a 2.5 M PhCHO solution in PhMe gave the crude product. Purification by flash column chromatography on silica with 80:20 petroleum ether-EtOAc as eluent gave a 60:40 diastereomeric mixture of tetrazole **9** (175 mg, 57% of major and 38% of minor) as a colourless oil.

1-{1-[2-(2-Phenylpropan-2-yl)-2*H*-1,2,3,4-tetrazol-5-yl]propyl}cyclohex-2-en-1-ol 10

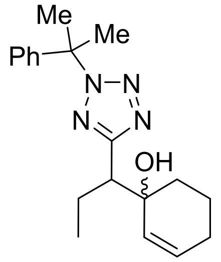

10

Using general procedure C, *n*-BuLi (0.46 mL of a 2.5 M solution in hexanes, 1.15 mmol, 2.3 eq) and **5** (115 mg, 0.5 mmol, 1.0 eq) in THF (4 mL) at 0 °C for 15 min then 2-cyclohexen-1-one (0.12 mL, 120 mg, 1.25 mmol, 2.5 eq) gave the crude product. Purification by flash column chromatography on silica with 70:30 petroleum ether-EtOAc as eluent gave a 65:35 diastereomeric mixture of tetrazole **10** (128 mg, 46% of major and 27% of minor) as a yellow oil, R_F (70:30 petroleum ether-EtOAc) 0.2; IR (ATR) cm^{-1}; ^{1}H NMR (400 MHz, $CDCl_3$)(65:35 mixtures of diastereomers) δ 7.32–7.23 (m, 3H, Ph), 7.04–7.01 (m, 1.3H, Ph), 6.99–6.97 (m, 0.7H, Ph), 5.84–5.75 (m, 1.3H, HC=CH), 5.38–5.34 (m, 0.7H, HC=CH), 3.12 (dd, *J* = 11.5, 4.0 Hz, 0.35H, TzCH), 3.05 (dd, *J* = 11.0, 4.0 Hz, 0.65H, TzCH), 2.77 (s, 0.65H, OH), 2.39 (s, 0.35H, OH), 2.16 (s, 3.9H, CMe_2), 2.15 (s, 2.1H, CMe_2), 2.07–1.81 (m, 4H, CH_2), 1.75–1.51 (m, 4H, CH_2), 0.75 (t, *J* = 7.5 Hz, 2H, *Me*CH_2), 0.72(t, *J* = 7.5 Hz, 1H, *Me*CH_2); ^{13}C NMR (101 MHz, $CDCl_3$)(mixtures of diastereomers) δ 166.8 (C), 166.6 (C), 144.3 (C), 144.1 (C), 131.4 (CH), 131.1 (CH), 130.9 (CH), 130.6 (CH), 128.6 (2 × CH), 127.8 (2 × CH), 124.5 (2 × CH), 71.1 (C), 71.0 (C), 68.3 (C), 68.2

(C), 49.3(2 × CH), 33.8 (CH_2), 32.4 (CH_2), 29.2 (CH_3), 29.1 (3 × CH_3), 25.1 (CH_2), 25.0 (CH_2), 21.7 (CH_2), 21.2 (CH_2), 18.6 (2 × CH_2), 12.5 (CH_3), 12.4 (CH_3);); MS (ESI) *m/z* 323 [$(M + H)^+$, 100], 309 [$(M – OH)^+$, 50], 232 [$(M – OH – Ph)^+$, 15], 191 [$(M – OH – CMe_2Ph)^+$, 13]; HRMS (ESI) *m/z* $C_{19}H_{26}N_4O$ ($[M + H]^+$) calcd for 327.2179, found 327.2192.

5-(1-Phenylbutan-2-yl)-2-(2-phenylpropan-2-yl)-2*H*-1,2,3,4-tetrazole 11

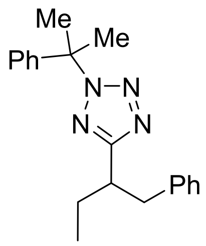

11

Using general procedure C, *n*-BuLi (0.46 mL of a 2.5 M solution in hexanes, 1.15 mmol, 2.3 eq) and **5** (115 mg, 0.5 mmol, 1.0 eq) in THF (4 mL) at 0 °C for 15 min then BnBr (0.15 mL, 214 mg, 1.25 mmol, 2.5 eq) gave the crude product. Purification by flash column chromatography on silica with 85:5:10 petroleum ether-EtOAc-PhMe as eluent gave tetrazole **11** (97 mg, 61%) as a colourless oil, R_F (85:5:10 petroleum ether-EtOAc-PhMe) 0.2; IR (ATR) 3087, 3058, 3028, 2988, 2966, 2953, 2919, 2871, 1496, 1455, 1447, 1390, 1366, 1334, 1311, 1250 ,1238, 1206, 1192, 1168, 1146, 1105, 1063, 1030, 1020, 972, 948, 922 cm^{-1}; ^1H NMR (400 MHz, $CDCl_3$) δ 7.29–7.24 (m, 3H, Ph), 7.23–7.13 (m, 3H, Ph), 7.06-7.03 (m, 2H, Ph), 6.91–6.88 (m, 2H, Ph), 3.28 (dddd, *J* = 8.5, 8.5, 7.5, 7.5 Hz, 1H, TzCH), 3.10 (dd, *J* = 13.5, 8.5 Hz, 1H, $PhCH_AH_B$), 3.03 (dd, *J* = 13.5, 7.5 Hz, 1H, $PhCH_AH_B$), 2.09 (s, 6H, CMe_2), 1.87–1.77 (m, 2H, $MeCH_2$), 0.83 (t, *J* = 7.5 Hz, 3H, $MeCH_2$); ^{13}C NMR (101 MHz, $CDCl_3$) δ 169.7 (C), 144.5 (C), 128.6 (CH), 127.7 (CH), 124.5 (CH), 67.9 (C), 38.9 (CH), 36.4 (CH_2), 31.8 (CH_2), 29.2 (CH_3), 29.1 (CH_3), 27.9 (CH), 27.4 (CH_2), 22.7 (CH_3), 22.4 (CH_3), 11.7 (CH_3). 168.7 (C), 163.1 (C), 144.0 (C), 128.6 (CH), 127.8 (CH), 124.6 (CH), 68.7 (C), 58.7 (C), 53.1 (CH_3), 29.1 (CH_3), 28.1 (CH_2), 9.5 (CH_3). 168.6 (C), 144.3 (C), 139.7 (C), 129.1 (CH), 128.6 (CH), 128.2 (CH), 127.6 (CH), 126.0 (CH), 124.5 (CH), 67.9 (C), 40.7 (CH), 40.32 (CH_2), 29.2 (2 × CH_3), 27.2 (CH_2), 11.7 (CH_3); MS (ESI) *m/z* 321 [$(M + H)^+$, 100], 244

[(M + H – Ph)$^+$, 42], 203 [(M + H_2 – CMe_2Ph)$^+$, 30]; HRMS (ESI) *m/z* $C_{20}H_{25}N_4$ ([M + H]$^+$) calcd for 321.2074, found 321.2080.

5-[1-(*tert*-Butyldimethylsilyl)propyl]-2-(2-phenylpropan-2-yl)-2*H*-1,2,3,4-tetrazole 12

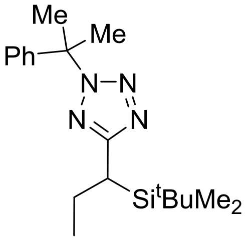

12

Using general procedure C, *n*-BuLi (0.46 mL of a 2.5 M solution in hexanes, 1.15 mmol, 2.3 eq) and **5** (115 mg, 0.5 mmol, 1.0 eq) in THF (4 mL) at 0 °C for 15 min then a solution of TBDMSCl (188 mg, 1.25 mmol, 2.5 eq) in THF (2 mL) gave the crude product. Purification by flash column chromatography on silica with 97:3 petroleum ether-EtOAc as eluent gave tetrazole **12** (115 mg, 66%) as a colourless oil, R_F (97:3 petroleum ether-EtOAc) 0.2; IR (ATR) 2958, 2929, 2884, 2857, 1497, 1471, 1449, 1390, 1369, 1316, 1300, 1186, 1155, 1075, 1075, 1050, 1011, 937, 907 cm^{-1}; ^{1}H NMR (300 MHz, $CDCl_3$) δ 7.32–7.21 (m, 3H, Ph), 7.09–7.04 (m, 2H, Ph), 2.49 (dd, *J* = 12.0, 3.5 Hz, 1H, TzCH), 2.12 (s, 6H, PhCMe_2), 2.07–1.72 (m, 2H, MeCH_2), 0.78 (t, *J* = 7.5 Hz, 3H, MeCH$_2$), 0.76 (s, 9H, CMe_3), 0.05 (s, 3H, SiMe_AMe$_B$), –0.15 (s, 3H, SiMe$_A$$Me_B$); ^{13}C NMR (75.5 MHz, $CDCl_3$) δ 168.9 (C), 144.3 (C), 128.5 (CH), 127.6 (CH), 124.7 (CH), 67.6 (C), 29.1 (2 × CH_3), 26.8 (CH_3), 26.6 (CH), 23.0 (CH_2), 17.4 (C), 14.3 (CH_3), –6.9 (CH_3), –7.1 (CH_3); MS (ESI) *m/z* 367 [(M + Na)$^+$, 17], 345 [(M + H)$^+$, 100], 227 [(M + H_2 – CMe_2Ph)$^+$, 53]; HRMS (ESI) *m/z* $C_{19}H_{32}N_4Si$ ([M + H]$^+$) calcd for 345.2469, found 345.2461.

Using general procedure C, *s*-BuLi (1.2 mL of a 2.3 M solution in cyclohexane/hexanes, 1.56 mmol, 1.6 eq) and **5** (230 mg, 1.0 mmol, 1.0 eq) in THF (7 mL) at –78 °C for 15 min then a

solution of TBDMSCl (301 mg, 2.0 mmol, 2.0 eq) in THF (2 mL) gave the crude product. Purification by flash column chromatography on silica with 97:3 petroleum ether-EtOAc as eluent gave tetrazole **12** (276 mg, 80%) as a colourless oil.

Using general procedure F, 1.1 M solution of tetrazole **5** in 1:1 PhMe/TMEDA (0.5 mL, 0.55 mmol, 1.0 eq) and a solution of 2.5 M *n*-BuLi in hexanes (0.5 mL, 1.15 mmol, 2.3 eq) were mixed for 30 s at rt followed by mixing with a 2.5 M TBDMSCl solution in PhMe gave the crude product. Purification by flash column chromatography on silica with 97:3 petroleum ether-EtOAc as eluent gave tetrazole **12** (65 mg, 34%) as a colourless oil.

5-(Butan-2-yl)-2-(2-phenylpropan-2-yl)-2*H*-1,2,3,4-tetrazole 13

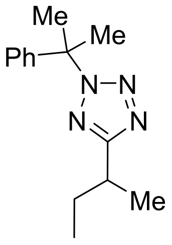

13

Using general procedure C, *n*-BuLi (0.46 mL of a 2.5 M solution in hexanes, 1.15 mmol, 2.3 eq) and **5** (115 mg, 0.5 mmol, 1.0 eq) in THF (4 mL) at 0 °C for 15 min then Me_2SO_4 (0.12 mL, 158 mg, 1.25 mmol, 2.5 eq) gave the crude product. Purification by flash column chromatography on silica with 95:5 petroleum ether-EtOAc as eluent gave tetrazole **13** (94 mg, 71%) as a colourless oil, R_F (95:5 petroleum ether-EtOAc) 0.2; IR (ATR) 3063, 2967, 2933, 2876, 1602, 1496, 1449, 1391, 1370, 1310 ,1253, 1187, 1165, 1107, 1078, 1059, 1019, 1002, 961, 925, 919 cm^{-1}; ^{1}H NMR (400 MHz, $CDCl_3$) δ 7.32–7.22 (m, 3H, Ph), 7.05–7.02 (m, 2H, Ph), 3.05 (ddq, *J* = 7.0, 7.0, 7.0 Hz, 1H, TzCH), 2.14 (s, 6H, CMe_2), 1.89–1.78 (m, 1H, $MeCH_AH_B$ with H_A italic), 1.74–1.63 (m, 1H, $MeCH_AH_B$), 1.36 (d, *J* = 7.0 Hz, 3H, *Me*CH), 0.86 (t, *J* = 7.5 Hz, 3H, *Me*CH_2); ^{13}C NMR (101 MHz, $CDCl_3$) δ 170.5 (C), 144.3 (C), 128.6 (CH), 127.7 (CH), 124.6 (CH), 67.8 (C), 32.9 (CH), 29.2 (2 × CH_3), 29.0 (CH_2), 19.1 (CH_3), 11.6 (CH_3); MS (ESI) *m/z* 511 [$(MM + Na)^+$, 17], 489 [$(MM + H)^+$, 49], 267 [$(M + Na)^+$, 20], 245 [$(M + H)^+$, 100], 168 [$(M + H – Ph)^+$, 16], 127 [$(M + H_2 – CMe_2Ph)^+$, 17]; HRMS (ESI) *m/z* $C_{14}H_{21}N_4$ ($[M + H]^+$) calcd for 245.1761, found 245.1770.

Using general procedure F, 1.1 M solution of tetrazole **5** in 1:1 PhMe/TMEDA (0.5 mL, 0.55 mmol, 1.0 eq) and a solution of 2.5 M *n*-BuLi in hexanes (0.5 mL, 1.15 mmol, 2.3 eq) were

mixed for 30 s at rt followed by mixing with a 2.5 M MeI solution in PhMe gave the crude product. Purification by flash column chromatography on silica with 95:5 petroleum ether-EtOAc as eluent gave tetrazole **13** (124 mg, 92%) as a colourless oil.

5-(6-Methylheptan-3-yl)-2-(2-phenylpropan-2-yl)-2*H*-1,2,3,4-tetrazole 14

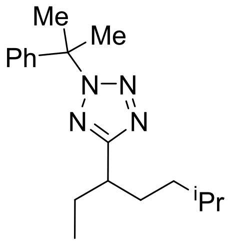

14

Using general procedure C, *n*-BuLi (0.46 mL of a 2.5 M solution in hexanes, 1.15 mmol, 2.3 eq) and **5** (115 mg, 0.5 mmol, 1.0 eq) in THF (4 mL) at 0 °C for 15 min then isopentyl bromide (0.15 mL, 189 mg, 1.25 mmol, 2.5 eq) gave the crude product. Purification by flash column chromatography on silica with 97:3 petroleum ether-EtOAc as eluent gave tetrazole **14** (94 mg, 71%) as a colourless oil, R_F (97:3 petroleum ether-EtOAc) 0.2; IR (ATR) 3064, 2956, 2934, 2870, 1602, 1497, 1488, 1449, 1384, 1369, 1312, 1253, 1185, 1164, 1107, 1077, 1064, 1031, 1017, 1002, 970, 943, 926, 908 cm^{-1}; ^{1}H NMR (400 MHz, $CDCl_3$) δ 7.31–7.23 (m, 3H, Ph), 7.02–7.00 (m, 2H, Ph), 2.90 (dddd, *J* = 8.5, 8.5, 6.0, 6.0 Hz, 1H, TzCH), 2.15 (s, 6H, CMe_2), 1.81–1.67 (m, 4H, ^{i}PrCH_2CH_2), 1.47 (t sept, *J* = 6.5, 6.5 Hz, 1H C*H*Me_2), 1.17–1.07 (m, 1H, MeC$H_A$$H_B$), 1.00–0.88 (m, 1H, MeC$H_A$$H_B$), 0.82–0.79 (m, 9H, CH*Me*$_2$, *Me*CH_2); ^{13}C NMR (101 MHz, $CDCl_3$) δ 169.7 (C), 144.5 (C), 128.6 (CH), 127.7 (CH), 124.5 (CH), 67.9 (C), 38.9 (CH), 36.4 (CH_2), 31.8 (CH_2), 29.2 (CH_3), 29.1 (CH_3), 27.9 (CH), 27.4 (CH_2), 22.7 (CH_3), 22.4 (CH_3), 11.7 (CH_3); MS (ESI) *m/z* 323 [(M + Na)$^+$, 19], 301 [(M + H)$^+$, 100], 224 [(M + H – Ph)$^+$, 37], 183 [(M + H_2 – CMe_2Ph)$^+$, 34]; HRMS (ESI) *m/z* $C_{18}H_{29}N_4$ ([M + H]$^+$) calcd for 301.2387, found 301.2398.

5-(Hex-5-en-3-yl)-2-(2-phenylpropan-2-yl)-2*H*-1,2,3,4-tetrazole 15

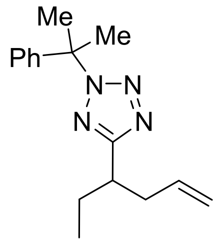

15

Using general procedure C, *n*-BuLi (0.46 mL of a 2.5 M solution in hexanes, 1.15 mmol, 2.3 eq) and **5** (115 mg, 0.5 mmol, 1.0 eq) in THF (4 mL) at 0 °C for 15 min then AllylBr (0.11 mL, 151 mg, 1.25 mmol, 2.5 eq) gave the crude product. Purification by flash column chromatography on silica with 90:10 petroleum ether-EtOAc as eluent gave tetrazole **15** (121 mg, 90%) as a colourless oil, R_F (90:10 petroleum ether-EtOAc) 0.2; IR (ATR) 3065, 2963, 2934, 2875, 1641, 1602, 1497, 1448, 1416, 1391, 1371 ,1307, 1252, 1212, 1185, 1163, 1106, 1077, 1063, 1031, 1019, 994, 943, 913 cm^{-1}; ^{1}H NMR (400 MHz, $CDCl_3$) δ 7.31–7.23 (m, 3H, Ph), 7.02–6.98 (m, 2H, Ph), 5.69 (dddd, *J* = 17.0, 10.0, 7.0, 7.0 Hz, 1H, $H_2C{=}CH$), 4.97–4.90 (m, 2H, $H_2C{=}CH$), 3.05 (dddd, *J* = 7.5, 7.5, 6.5, 6.5 Hz, 1H, TzCH), 2.57–2.54 (m, 2H, $CH_2CH{=}CH_2$), 2.14 (s, 6H, CMe_2), 1.83–1.76 (m, 2H, $MeCH_2$), 0.83 (t, *J* = 7.5 Hz, 3H, $MeCH_2$); ^{13}C NMR (101 MHz, $CDCl_3$) δ 168.9 (C), 144.4 (C), 135.9 (CH), 128.6 (CH), 127.7 (CH), 124.6 (CH), 116.9 (CH_2), 68.0 (C), 38.4 (CH), 38.3 (CH_2), 29.2 (2 × CH_3), 26.8 (CH_2), 11.6 (CH_3); MS (ESI) *m/z* 563 [$(MM + Na)^+$, 38], 541 [$(MM + H)^+$, 70], 303 [$(M + Na)^+$, 15], 271 [$(M + H)^+$, 100], 153 [$(M + H_2 - CMe_2Ph)^+$/ $(M - CH_2CH{=}CH_2 - Ph)^+$, 83]; HRMS (ESI) *m/z* $C_{16}H_{24}N_4$ ($[M + H]^+$) calcd for 271.1917, found 271.1914.

Using general procedure F, 1.1 M solution of tetrazole **5** in THF (0.5 mL, 0.55 mmol, 1.0 eq) and a solution of 2.5 M *n*-BuLi in hexanes (0.5 mL, 1.15 mmol, 2.3 eq) were mixed 2.6 s at rt followed by mixing with a 2.5 M allylBr solution in THF gave the crude product. Inspection of the ^{1}H NMR spectrum of the crude mixture in the presence of 1,3,5-trimethoxybenzene as internal standard showed that tetrazole **15** was present in 35% conversion.

Using general procedure F, 0.55 M solution of tetrazole **5** in THF (0.5 mL, 0.28 mmol, 1.0 eq) and a solution of 2.5 M *n*-BuLi in hexanes/ PhMe (0.5 mL, 1.15 mmol, 4.1 eq) were mixed 2.6 s at rt followed by mixing with a 2.5 M allylBr solution in THF gave the crude product. Inspection of the ^{1}H NMR spectrum of the crude mixture in the presence of 1,3,5-trimethoxybenzene as internal standard showed that tetrazole **15** was present in trace conversion.

Using general procedure F, 0.76 M solution of tetrazole **5** in THF (0.5 mL, 0.38 mmol, 1.0 eq) and a solution of 1.75 M *n*-BuLi in hexanes/ PhMe (0.5 mL, 0.88 mmol, 2.3 eq) were mixed 2.6 s at rt followed by mixing with a 2.0 M allylBr solution in THF gave the crude product. Inspection of the ^{1}H NMR spectrum of the crude mixture in the presence of 1,3,5-trimethoxybenzene as internal standard showed that tetrazole **15** was present in 48% conversion.

Using general procedure F, 0.54 M solution of tetrazole **5** in THF (0.5 mL, 0.27 mmol, 1.0 eq) and a solution of 1.25 M *n*-BuLi in hexanes/ PhMe (0.5 mL, 0.63 mmol, 2.3 eq) were mixed 2.6 s at rt followed by mixing with a 1.5 M allylBr solution in THF gave the crude product. Inspection of the ^{1}H NMR spectrum of the crude mixture in the presence of 1,3,5-trimethoxybenzene as internal standard showed that tetrazole **15** was present in 12% conversion.

Using general procedure F, 0.33 M solution of tetrazole **5** in THF (0.5 mL, 0.17 mmol, 1.0 eq) and a solution of 0.75 M *n*-BuLi in hexanes/ PhMe (0.5 mL, 0.38 mmol, 2.3 eq) were mixed 2.6 s at rt followed by mixing with a 2.5 M allylBr solution in THF gave the crude product. Inspection of the ^{1}H NMR spectrum of the crude mixture in the presence of 1,3,5-trimethoxybenzene as internal standard showed that tetrazole **15** was present in 12% conversion.

Using general procedure F, 0.33 M solution of tetrazole **5** in THF (0.5 mL, 0.17 mmol, 1.0 eq) and a solution of 0.75 M *n*-BuLi in hexanes/ PhMe (0.5 mL, 0.38 mmol, 2.3 eq) were mixed 13 s at rt followed by mixing with a 1.0 M allylBr solution in THF gave the crude product. Inspection of the ^{1}H NMR spectrum of the crude mixture in the presence of 1,3,5-trimethoxybenzene as internal standard showed that tetrazole **15** was present in 12% conversion.

Using general procedure F, 0.33 M solution of tetrazole **5** in THF (0.5 mL, 0.17 mmol, 1.0 eq) and a solution of 0.75 M *n*-BuLi in hexanes/ PhMe (0.5 mL, 0.38 mmol, 2.3 eq) were mixed 10 min at rt followed by mixing with a 1.0 M allylBr solution in THF gave the crude product. Inspection of the ^{1}H NMR spectrum of the crude mixture in the presence of 1,3,5-trimethoxybenzene as internal standard showed that tetrazole **15** was present in 14% conversion.

Using general procedure F, 1.1 M solution of tetrazole **5** in PhMe (0.5 mL, 0.55 mmol, 1.0 eq) and a solution of 2.5 M *n*-BuLi in hexanes (0.5 mL, 1.15 mmol, 2.3 eq) were mixed 2.6 s at rt followed by mixing with a 2.5 M allylBr solution in THF gave the crude product. Inspection of the ^{1}H NMR spectrum of the crude mixture in the presence of 1,3,5-trimethoxybenzene as internal standard showed that tetrazole **15** was present in 0% conversion.

Using general procedure F, 1.1 M solution of tetrazole **5** in 4:1 PhMe/TMEDA (0.5 mL, 0.55 mmol, 1.0 eq) and a solution of 2.5 M *n*-BuLi in hexanes (0.5 mL, 1.15 mmol, 2.3 eq) were mixed for 5 min at rt followed by mixing with a 2.5 M allylBr solution in PhMe gave the crude product. Inspection of the ^{1}H NMR spectrum of the crude mixture in the presence of 1,3,5-trimethoxybenzene as internal standard showed that tetrazole **15** was present in 62% conversion.

Using general procedure F, 1.1 M solution of tetrazole **5** in 4:1 PhMe/TMEDA (0.5 mL, 0.55 mmol, 1.0 eq) and a solution of 2.5 M *n*-BuLi in hexanes (0.5 mL, 1.15 mmol, 2.3 eq) were mixed for 1.67 min at rt followed by mixing with a 2.5 M allylBr solution in PhMe gave the crude product. Inspection of the ^{1}H NMR spectrum of the crude mixture in the presence of 1,3,5-trimethoxybenzene as internal standard showed that tetrazole **15** was present in 60% conversion.

Using general procedure F, 1.1 M solution of tetrazole **5** in 4:1 PhMe/TMEDA (0.5 mL, 0.55 mmol, 1.0 eq) and a solution of 2.5 M *n*-BuLi in hexanes (0.5 mL, 1.15 mmol, 2.3 eq) were mixed for 1 min at rt followed by mixing with a 2.5 M allylBr solution in PhMe gave the crude product. Inspection of the ^{1}H NMR spectrum of the crude mixture in the presence of 1,3,5-trimethoxybenzene as internal standard showed that tetrazole **15** was present in 59% conversion.

Using general procedure F, 1.1 M solution of tetrazole **5** in 4:1 PhMe/TMEDA (0.5 mL, 0.55 mmol, 1.0 eq) and a solution of 2.5 M *n*-BuLi in hexanes (0.5 mL, 1.15 mmol, 2.3 eq) were mixed for 30 s at rt followed by mixing with a 2.5 M allylBr solution in PhMe gave the crude product. Inspection of the ^{1}H NMR spectrum of the crude mixture in the presence of 1,3,5-trimethoxybenzene as internal standard showed that tetrazole **15** was present in 49% conversion.

Using general procedure F, 0.8 M solution of tetrazole **5** in 4:1 PhMe/TMEDA (0.5 mL, 0.4 mmol, 1.0 eq) and a solution of 2.5 M *n*-BuLi in hexanes (0.5 mL, 1.15 mmol, 2.9 eq) were mixed for 1 min at rt followed by mixing with a 2.5 M allylBr solution in PhMe gave the crude product. Inspection of the ^{1}H NMR spectrum of the crude mixture in the presence of 1,3,5-trimethoxybenzene as internal standard showed that tetrazole **15** was present in 46% conversion.

Using general procedure F, 1.1 M solution of tetrazole **5** in 1:1 PhMe/TMEDA (0.5 mL, 0.55 mmol, 1.0 eq) and a solution of 2.5 M *n*-BuLi in hexanes (0.5 mL, 1.15 mmol, 2.3 eq) were mixed for 1 min at rt followed by mixing with a 2.5 M allylBr solution in PhMe gave the crude product. Inspection of the ^{1}H NMR spectrum of the crude mixture in the presence of 1,3,5-trimethoxybenzene as internal standard showed that tetrazole **15** was present in 73% conversion.

Using general procedure F, 1.1 M solution of tetrazole **5** in 1:1 PhMe/TMEDA (0.5 mL, 0.55 mmol, 1.0 eq) and a solution of 2.5 M *n*-BuLi in hexanes (0.5 mL, 1.15 mmol, 2.3 eq) were mixed for 1.67 min at rt followed by mixing with a 2.5 M allylBr solution in PhMe gave the crude product. Inspection of the ^{1}H NMR spectrum of the crude mixture in the presence of 1,3,5-trimethoxybenzene as internal standard showed that tetrazole **15** was present in 70% conversion.

Using general procedure F, 1.1 M solution of tetrazole **5** in 1:1 PhMe/TMEDA (0.5 mL, 0.55 mmol, 1.0 eq) and a solution of 2.5 M *n*-BuLi in hexanes (0.5 mL, 1.15 mmol, 2.3 eq) were mixed for 30 s at rt followed by mixing with a 2.5 M allylBr solution in PhMe gave the crude product. Inspection of the ^{1}H NMR spectrum of the crude mixture in the presence of 1,3,5-trimethoxybenzene as internal standard showed that tetrazole **15** was present in 70% conversion.

Using general procedure F, 1.1 M solution of tetrazole **5** in 1:1 PhMe/TMEDA (0.5 mL, 0.55 mmol, 1.0 eq) and a solution of 2.5 M *n*-BuLi in hexanes (0.5 mL, 1.15 mmol, 2.3 eq) were mixed for 30 s at rt followed by mixing with a 2.5 M allylBr solution in PhMe gave the crude product. Purification by flash column chromatography on silica with 90:10 petroleum ether-EtOAc as eluent gave tetrazole **15** (113 mg, 73%) as a colourless oil.

1-Phenyl-2-[2-(2-phenylpropan-2-yl)-2*H*-1,2,3,4-tetrazol-5-yl]butan-1-one 16

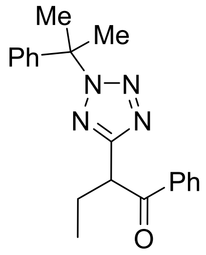

16

Using general procedure C, *n*-BuLi (0.46 mL of a 2.5 M solution in hexanes, 1.15 mmol, 2.3 eq) and **5** (115 mg, 0.5 mmol, 1.0 eq) in THF (4 mL) at 0 °C for 15 min then *N*-methoxy-*N*-methylbenzamide (0.19 mL, 206 mg, 1.25 mmol, 2.5 eq) gave the crude product. Purification by flash column chromatography on silica with 80:10:10 petroleum ether-EtOAc-PhMe as eluent gave tetrazole **16** (136 mg, 81%) as a colourless oil, R_F (80:10:10 petroleum ether-EtOAc-PhMe) 0.2; IR (ATR) 3061, 2968, 2936, 2876, 1689 (C=O str), 1597, 1580, 1497, 1480, 1447, 1392, 1371, 1344, 1308, 1260 ,1224, 1206, 1183, 1161, 1105, 1077, 1066, 1021, 1001, 987, 933, 908 cm^{-1}; ^1H NMR (400 MHz, $CDCl_3$) δ 7.98–7.95 (m, 2H, Ph), 7.25 (tt *J* = 7.5, 1.5 Hz, 1H, Ph), 7.43–7.38 (m, 2H, Ph), 7.24–7.20 (m, 3H, Ph), 6.92–6.88 (m, 2H, Ph), 4.90 (dd, *J* = 8.0, 6.5 Hz, 1H, TzCH), 2.33–2.26 (m, 1H, $MeCH_AH_B$), 2.19–2.13 (m, 1H, $MeCH_AH_B$), 2.11 (s, 3H, CMe_AMe_B), 2.10 (s, 3H, CMe_AMe_B), 0.97 (t, *J* = 7.5 Hz, 3H, $MeCH_2$); ^{13}C NMR (101 MHz, $CDCl_3$) δ 196.5 (C), 164.4 (C), 144.1 (C), 136.2 (C), 133.1 (CH), 128.8 (CH), 128.6 (2 × CH), 127.7 (CH), 124.5 (CH), 68.5 (C), 47.0 (CH), 29.2 (CH_3), 29.0 (CH_3), 24.1 (CH_2), 12.1 (CH_3); MS (ESI) *m/z* 335 [$(M + H)^+$, 100], 258 [$(M + H - Ph)^+$, 15]; HRMS (ESI) *m/z* $C_{20}H_{23}N_4O$ ($[M + H]^+$) calcd for 335.1866, found 335.1877.

Using general procedure F, 1.1 M solution of tetrazole **5** in 1:1 PhMe/TMEDA (0.5 mL, 0.55 mmol, 1.0 eq) and a solution of 2.5 M *n*-BuLi in hexanes (0.5 mL, 1.15 mmol, 2.3 eq) were mixed for 30 s at rt followed by mixing with a 2.5 M *N*-methoxy-*N*-methylbenzamide solution in PhMe gave the crude product. Purification by flash column chromatography on silica with 80:10:10 petroleum ether-EtOAc-PhMe as eluent gave tetrazole **16** (161 mg, 93%) as a colourless oil.

Attempted synthesis of 5-(1-iodopropyl)-2-(2-phenylpropan-2-yl)-2*H*-1,2,3,4-tetrazole

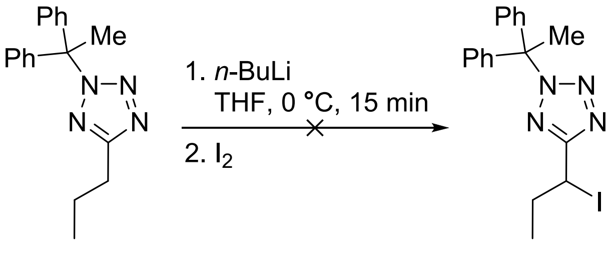

3 **3'**

Using general procedure C, n-BuLi (0.46 mL of a 2.5 M solution in hexanes, 1.15 mmol, 2.3 eq) and **3** (146 mg, 1.0 mmol, 1.0 eq) in THF (7 mL) at 0 °C for 15 min then I_2 (318 mg, 1.25 mmol, 2.5 eq) in THF (2 mL) gave the crude product. ^{1}H NMR spectrum of the crude mixture revealed a complex mixture with no identifiable products and therefore further purifications were not attempted.

5-[2-(2-Phenylpropan-2-yl)-2*H*-1,2,3,4-tetrazol-5-yl]heptan-3-ol 17

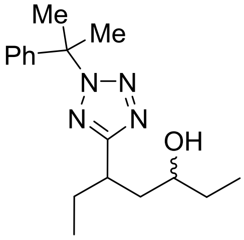

17

Using general procedure C, n-BuLi (0.46 mL of a 2.5 M solution in hexanes, 1.15 mmol, 2.3 eq) and **5** (115 mg, 0.5 mmol, 1.0 eq) in THF (4 mL) at 0 °C for 15 min then 1,2-epoxybutane (0.11 mL, 90 mg, 1.25 mmol, 2.5 eq) gave the crude product. Purification by flash column chromatography on silica with 60:40 petroleum ether-EtOAc as eluent gave diastereomeric mixture of tetrazole **17** (85 mg, 39% of major and 17% of minor) as a colourless oil, R_F (60:40 petroleum ether-EtOAc) 0.2; IR (ATR) 3410 (O-H str), 2962, 1934, 1875, 1497, 1449, 1391, 1381, 1371, 1355, 1307, 1253, 1184, 1163, 1142, 1108, 1076, 1061, 1019, 981, 967, 924, 910 cm^{-1}; ^{1}H NMR (400 MHz, $CDCl_3$)(70:30 mixture of diastereomers) δ 7.34–7.22 (m, 3H, Ph), 7.07–7.00 (m, 2H, Ph), 3.54 (ddd, J = 7.5, 7.5, 5.0 Hz, 0.3H, OCH), 3.26 (dddd, J = 8.5, 6.0, 4.5, 2.0 Hz, 0.7H, OCH), 3.21–3.09 (m, 1H, TzCH), 2.15 (s, 4.2H, CMe_3), 2.14 (s, 2.8H, CMe_3), 1.99–1.65 (m, 4H), 1.55–1.46 (m, 0.3H, CH_2), 1.46–1.37 (m, 1.7H, CH_2), 0.91 (t, J = 7.5 Hz, 1H, Me), 0.88–0.78 (m, 5H, Me); ^{13}C NMR (101 MHz, $CDCl_3$)(mixture of diastereomers) δ 169.6 (C), 169.1 (C), 144.2 (C), 128.6 (CH), 127.8 (2 × CH), 124.6 (CH), 124.5 (CH), 71.7 (CH), 70.6 (CH),

68.1 (C), 41.3 (CH_2), 40.9 (CH_2), 35.7 (CH), 35.2 (CH), 30.5 (CH_2), 30.4 (CH_2), 29.1 (4 × CH_3), 27.8 (CH_2), 27.3 (CH_2), 11.8 (CH_3), 11.3 (CH_3), 10.0 (CH_3), 9.8 (CH_3); MS (ESI) *m/z* 325 [$(M + Na)^+$, 4], 303 [$(M + H)^+$, 85], 185 [$(M + H_2 – CMe_2Ph)^+$, 100], 167 [$(M + H – CMe_2Ph – OH)^+$, 38]; HRMS (ESI) *m/z* $C_{17}H_{27}N_4O$ ($[M + H]^+$) calcd for 303.2185, found 303.2187.

2-Ethyl-2-[2-(2-phenylpropan-2-yl)-2*H*-1,2,3,4-tetrazol-5-yl]-*N*,*N*'-bis(propan-2-yl)propanediamide 18

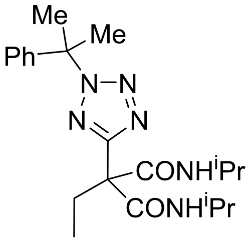

18

Using general procedure C, *n*-BuLi (0.46 mL of a 2.5 M solution in hexanes, 1.15 mmol, 2.3 eq) and **5** (115 mg, 0.5 mmol, 1.0 eq) in THF (4 mL) at 0 °C for 15 min then ^{i}PrNCO (0.12 mL, 106 mg, 1.25 mmol, 2.5 eq) gave the crude product. Purification by flash column chromatography on silica with 85:15 petroleum ether-EtOAc as eluent gave tetrazole **18** (101 mg, 50%) as a colourless oil, R_F (85:15 petroleum ether-EtOAc) 0.2; IR (ATR) 3229 (N-H str), 2971, 2937, 2877, 1674 (C=O str), 1517, 1457, 1387, 1368, 1318, 1293, 1254, 1238, 1189, 1171, 1130, 1069, 1028, 1001, 956, 924 cm^{-1}; ^{1}H NMR (400 MHz, $CDCl_3$) δ 7.54 (br d, *J* = 7.5 Hz, 2H, NH), 7.34–7.25 (m, 3H, Ph), 7.09–7.07 (m, 2H, Ph), 4.03 (d sept, *J* = 7.5, 6.5 Hz, 2H, CH), 2.56 (q, *J* = 7.5 Hz, 2H, CH_2), 2.19 (s, 6H, CMe_2), 1.12 (d, *J* = 6.5 Hz, 6H, $CH(Me_2)_A$), 1.10 (d, *J* = 6.5 Hz, 6H, $CH(Me_2)_B$) 0.89 (t, *J* = 7.5 Hz, 3H, *Me*CH_2); ^{13}C NMR (101 MHz, $CDCl_3$) δ 168.9 (C), 144.4 (C), 135.9 (CH), 128.6 (CH), 127.7 (CH), 124.6 (CH), 116.9 (CH_2), 68.0 (C), 38.4 (CH), 38.3 (CH_2), 29.2 (2 × CH_3), 26.8 (CH_2), 11.6 (CH_3). 168.1 (C), 164.7 (C), 143.8 (C), 128.7 (CH), 127.9 (CH), 124.6

(CH), 68.8 (C), 59.1 (C), 42.0 (CH), 30.3 (CH_2), 29.1 (CH_3), 22.4 (CH_3), 22.3 (CH_3), 10.0 (CH_3); MS (ESI) *m/z* 423 [(M + Na)$^+$, 16], 401 [(M + H)$^+$, 100]; HRMS (ESI) *m/z* $C_{21}H_{32}N_6O_2$ ([M + H]$^+$) calcd for 401.2660, found 401.2661.

Using general procedure F, 1.1 M solution of tetrazole **5** in 1:1 PhMe/TMEDA (0.5 mL, 0.55 mmol, 1.0 eq) and a solution of 2.5 M *n*-BuLi in hexanes (0.5 mL, 1.15 mmol, 2.3 eq) were mixed for 30 s at rt followed by mixing with a 2.5 M ^{i}PrNCO solution in PhMe gave the crude product. Purification by flash column chromatography on silica with 85:15 petroleum ether-EtOAc as eluent gave tetrazole **18** (99 mg, 45%) as a colourless oil.

1,3-Dimethyl 2-ethyl-2-[2-(2-phenylpropan-2-yl)-2*H*-1,2,3,4-tetrazol-5-yl]propanedioate 19

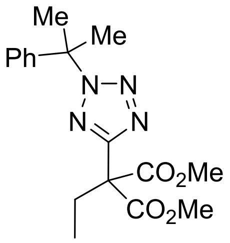

19

Using general procedure C, *n*-BuLi (0.7 mL of a 2.5 M solution in hexanes, 1.75 mmol, 3.5 eq) and **5** (115 mg, 0.5 mmol, 1.0 eq) in THF (4 mL) at 0 °C for 15 min then isopentyl bromide (0.15 mL, 189 mg, 2.0 mmol, 4.0 eq) gave the crude product. Purification by flash column chromatography on silica with 80:20 petroleum ether-EtOAc as eluent gave tetrazole **19** (80 mg, 46%) as a colourless oil, R_F (80:20 petroleum ether-EtOAc) 0.2; IR (ATR) 3063, 2990, 2953, 2884, 1739 (C=O str), 1602, 1559, 1698, 1448, 1436, 1392, 1370, 1337, 1295, 1241, 1190, 1166, 1120, 1091, 1064, 1025, 1001, 944, 918 cm^{-1}; ^{1}H NMR (400 MHz, $CDCl_3$) δ 7.32–7.23 (m, 3H, Ph), 7.04–7.01 (m, 2H, Ph), 3.78 (s, 6H, OMe), 2.46 (q, *J* = 7.5 Hz, 2H, CH_2), 2.16 (s, 6H, CMe_2), 1.03 (t, *J* = 7.5 Hz, 3H, *Me*CH_2); ^{13}C NMR (101 MHz, $CDCl_3$) δ 169.7 (C), 144.5 (C), 128.6 (CH), 127.7 (CH), 124.5 (CH), 67.9 (C), 38.9 (CH), 36.4 (CH_2), 31.8 (CH_2), 29.2 (CH_3), 29.1 (CH_3), 27.9 (CH), 27.4 (CH_2), 22.7 (CH_3), 22.4 (CH_3), 11.7 (CH_3). 168.7 (C), 163.1 (C), 144.0 (C), 128.6 (CH), 127.8 (CH), 124.6 (CH), 68.7 (C), 58.7 (C), 53.1 (CH_3), 29.1 (CH_3), 28.1 (CH_2), 9.5 (CH_3); MS

(ESI) *m/z* 369 [$(M + Na)^+$, 27], 345 [$(M + H)^+$, 100], 229 [$(M + H_2 – CMe_2Ph)^+$, 35]; HRMS (ESI) *m/z* $C_{17}H_{23}N_4O_4$ ($[M + H]^+$) calcd for 347.1714, found 347.1729.

1,1-Diphenyl-2-[2-(2-phenylpropan-2-yl)-2*H*-1,2,3,4-tetrazol-5-yl]propan-1-ol 20

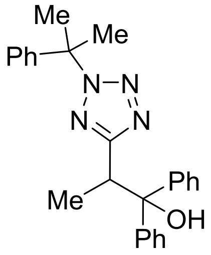

20

Using general procedure C, *n*-BuLi (0.46 mL of a 2.5 M solution in hexanes, 1.15 mmol, 2.3 eq) and **ST15** (108 mg, 0.5 mmol, 1.0 eq) in THF (4 mL) at 0 °C for 15 min then a solution of Ph_2CO (228 mg, 1.25 mmol, 2.5 eq) in THF (2 mL) gave the crude product. Purification by flash column chromatography on silica with 90:10 petroleum ether-Et_2O as eluent gave tetrazole **20** (141 mg, 70%) as a white solid, m.p. 115–119 °C; R_F (90:10 petroleum ether-Et_2O) 0.2; IR (ATR) 3469 (O-H str), 3087, 3062, 3024, 3006, 2988, 2963, 2940, 2874, 1598, 1495, 1447, 1388, 1369, 1336, 1317, 1292, 1272, 1241, 1189, 1177, 1166, 1159, 1136, 1096, 1079, 1069, 1031, 1020, 1001, 978, 959, 921, 911 cm^{-1}; ^1H NMR (400 MHz, $CDCl_3$) δ 7.62–7.59 (m, 2H, Ph), 7.48–7.45 (m, 2H, Ph), 7.34–7.30 (m, 2H, Ph), 7.25–7.05 (m, 7H, Ph), 6.61–6.58 (m, 2H, Ph), 4.67 (s, 1H, OH), 4.50 (q, *J* = 7.0 Hz, 1H, TzCH), 2.05 (s, 3H, $C\mathit{Me}_A Me_B$), 2.03 (s, 3H, $CMe_A \mathit{Me}_B$), 1.32 (d, *J* = 7.0 Hz, 3H, *Me*CH); ^{13}C NMR (101 MHz, $CDCl_3$) δ 168.7 (C), 147.0 (C), 144.6 (C), 144.0 (C), 128.6 (CH), 128.2 (CH), 128.0 (CH), 127.6 (CH), 126.5 (CH), 126.4 (CH), 125.8 (CH), 125.5 (CH), 124.2 (CH), 79.3 (C), 68.6 (C), 39.8 (CH), 29.3 (CH_3), 29.1 (CH_3), 15.8 (CH_3); MS (ESI) *m/z* 413 [$(M + Na)^+$, 21], 399 [$(M + H)^+$, 100], 381 [$(M – OH)^+$, 61], 322 [$(M + H – Ph)^+$, 20]; HRMS (ESI) *m/z* $C_{25}H_{27}N_4O$ ($[M + H]^+$) calcd for 399.2179, found 399.2188.

2-(2-Phenylpropan-2-yl)-5-(undec-1-en-4-yl)-2*H*-1,2,3,4-tetrazole 21

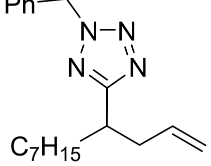

21

Using general procedure C, *n*-BuLi (0.46 mL of a 2.5 M solution in hexanes, 1.15 mmol, 2.3 eq) and **ST16** (150 mg, 0.5 mmol, 1.0 eq) in THF (4 mL) at 0 °C for 15 min then AllylBr (0.11 mL, 151 mg, 1.25 mmol, 2.5 eq) gave the crude product. Purification by flash column chromatography on silica with 95:5 petroleum ether-EtOAc as eluent gave tetrazole **21** (102 mg, 60%) as a colourless oil, R_F (95:5 petroleum ether-EtOAc) 0.2; IR (ATR) 3065, 2986, 2926, 2855, 1641, 1602, 1497, 1466, 1448, 1416, 1391, 1370, 1314, 1254, 1186, 1164, 1108, 1076, 1031, 1018, 993, 913 cm^{-1}; ^{1}H NMR (400 MHz, $CDCl_3$) δ 7.31–7.23 (m, 3H, Ph), 7.01–6.97 (m, 2H, Ph), 5.68 (dddd, *J* = 17.0, 10.0, 7.0, 7.0 Hz, 1H, $H_2C{=}C$*H*), 6.96–6.90 (m, 2H, H_2C=CH), 3.12 (dddd, *J* = 8.5, 8.5, 6.0, 6.0 Hz, 1H, TzCH), 2.56–2.42 (m, 2H, $H_2CH_2C{=}CH$), 2.14 (s, 6H, CMe_2), 1.80–1.69 (m, 2H, TzCHCH_2), 1.28–1.13 (m, 10H, TzCHCH_2(CH_2)$_5$), 0.86 (t, *J* = 7.0 Hz, 3H, *Me*CH_2); ^{13}C NMR (101 MHz, $CDCl_3$) δ 168.7 (C), 147.0 (C), 144.6 (C), 144.0 (C), 128.6 (CH), 128.2 (CH), 128.0 (CH), 127.6 (CH), 126.5 (CH), 126.4 (CH), 125.8 (CH), 125.5 (CH), 124.2 (CH), 79.3 (C), 68.6 (C), 39.8 (CH), 29.3 (CH_3), 29.1 (CH_3), 15.8 (CH_3). 169.2 (C), 144.4 (C), 135.9 (CH), 128.53 (CH), 127.7 (CH), 124.6 (CH), 116.6 (CH_2), 68.0 (C), 38.7 (CH_2), 36.8 (CH), 33.8 (CH_2), 31.8 (CH_2), 29.4 (2 × CH_2), 29.2 (CH_3), 27.1 (CH_2), 22.6 (CH_2), 14.1 (CH_3); MS (ESI) *m/z* 363 [(M + Na)$^+$, 17], 341 [(M + H)$^+$, 75], 223 [(M + H_2 – CMe_2Ph)$^+$, 100]; HRMS (ESI) *m/z* $C_{21}H_{33}N_4$ ([M + H]$^+$) calcd for 341.2700, found 341.2715.

Using general procedure C, *s*-BuLi (0.6 mL of a 1.3 M solution in cyclohexane/hexanes, 0.78 mmol, 1.6 eq) and **ST16** (150 mg, 0.5 mmol, 1.0 eq) in THF (4 mL) at –78 °C for 15 min then AllylBr (0.09 mL, 121 mg, 1.0 mmol, 2.0 eq) gave the crude product. Purification by flash column chromatography on silica with 95:5 petroleum ether-EtOAc as eluent gave tetrazole **21** (167 mg, 98%) as a colourless oil.

5-[1-(Methoxymethoxy)hex-5-en-3-yl]-2-(2-phenylpropan-2-yl)-2*H*-1,2,3,4-tetrazole 22

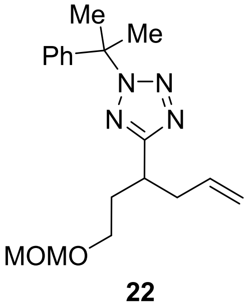

22

Using general procedure C, *n*-BuLi (0.46 mL of a 2.5 M solution in hexanes, 1.15 mmol, 2.3 eq) and **ST17** (124 mg, 0.5 mmol, 1.0 eq) in THF (4 mL) at 0 °C for 15 min then AllylBr (0.11 mL, 151 mg, 1.25 mmol, 2.5 eq) gave the crude product. Purification by flash column chromatography on silica with 80:20 petroleum ether-EtOAc as eluent gave tetrazole **22** (92 mg, 65%) as a colourless oil, R_F (80:20 petroleum ether-EtOAc) 0.2; IR (ATR) 3065, 2987, 2934, 2823, 1641, 1602, 1497, 1468, 1391, 1371, 1309, 1254, 1217, 1184, 1149, 1077, 1040, 1020, 824 cm^{-1}; ^1H NMR (400 MHz, $CDCl_3$) δ 7.37–7.19 (m, 3H, Ph), 7.04–6.97 (m, 2H, Ph), 5.69 (dddd, *J* = 17.5, 10.5, 7.0, 7.0 Hz, 1H, $H_2C{=}CH$), 5.00–4.89 (m, 2H, $H_2C{=}CH$), 4.53 (d, *J* = 6.5 Hz, 1H, $MeOCH_AH_B$), 4.50 (d, *J* = 6.5 Hz, 1H, $MeOCH_AH_B$), 3.53–3.44 (m, 1H, TzCH), 3.42–3.31 (m, 2H, OCH_2CH_2), 3.27 (s, 3H, OMe), 2.61–2.42 (m, 2H, CH_2), 2.14 (s, 6H, CMe_2), 2.12–2.02 (m, 2H, CH_2); ^{13}C NMR (101 MHz, CDCl3) δ 168.5 (C), 144.3 (C), 135.5 (CH), 128.6 (CH), 127.7 (CH), 124.6 (CH), 117.0 (CH_2), 96.4 (CH_2), 68.0 (C), 65.2 (CH_2), 55.1 (CH_2), 38.8 (CH), 33.6 (CH_2), 33.5 (CH_3), 29.1 (CH_3); MS (ESI) *m/z* 353 [$(M + Na)^+$, 10], 339 [$(M + H)^+$, 80], 213 [$(M + H_2 - CMe_2Ph)^+$, 100], 181 [$(M + H - CMe_2Ph - OMe)^+$, 75], 151 [$(M + H_3 - CMe_2Ph - OMOM)^+$, 19]; HRMS (ESI) *m/z* $C_{18}H_{27}N_4O_2$ ($[M + H]^+$) calcd for 331.2129, found 331.2122.

Using general procedure C, *s*-BuLi (1.0 mL of a 1.3 M solution in cyclohexane/hexanes, 1.3 mmol, 2.6 eq) and **ST17** (124 mg, 0.5 mmol, 1.0 eq) in THF (4 mL) at –78 °C for 15 min then a solution of Ph_2CO (273 mg, 1.5 mmol, 3.0 eq) in THF (2 mL) gave the crude product. Purification by flash column chromatography on silica with 80:20 petroleum ether-EtOAc as eluent gave tetrazole **22** (142 mg, 100%) as a colourless oil.

1,1,4-Triphenyl-2-[2-(2-phenylpropan-2-yl)-2*H*-1,2,3,4-tetrazol-5-yl]butan-1-ol 23

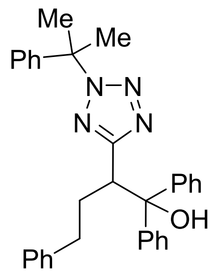

23

Using general procedure C, *n*-BuLi (0.46 mL of a 2.5 M solution in hexanes, 1.15 mmol, 2.3 eq) and **ST18** (153 mg, 0.5 mmol, 1.0 eq) in THF (4 mL) at 0 °C for 15 min then a solution of Ph_2CO (228 mg, 1.25 mmol, 2.5 eq) in THF (2 mL) gave the crude product. Purification by flash column

chromatography on silica with 97:3 petroleum ether-EtOAc as eluent gave tetrazole **23** (141 mg, 70%) as a white solid, m.p. 115–120 °C; R_F (97:3 petroleum ether-EtOAc) 0.2; IR (ATR) 3469 (O-H str), 3087, 3062, 3024, 3006, 2988, 2963, 2940, 2874, 1598, 1495, 1447, 1388, 1369, 1336, 1317, 1292, 1272, 1241, 1189, 1177, 1166, 1159, 1552, 1096, 1079, 1069, 1031, 1020 ,1001, 978, 959, 921, 911 cm^{-1}; ^1H NMR (400 MHz, $CDCl_3$) δ 7.48–7.38 (m, 2H, Ph), 7.40–7.15 (m, 9H, Ph), 7.12–7.08 (m, 2H, Ph), 7.04–7.00 (m, 3H, Ph), 6.61–6.58 (m, 2H, Ph), 4.71 (s, 1H, OH), 4.33 (dd, *J* = 11.5, 3.0 Hz, 1H, TzCH), 2.51–2.44 (m, 1H, $CHCH_AH_B$ with H_A italic), 2.40–2.24 (m, 2H, $PhCH_2$), 2.07 (s, 3H, $C\mathit{Me}_A Me_B$), 2.04 (s, 3H, $CMe_A\mathit{Me}_B$), 2.02–1.96 (m, 1H, $CHCH_A\mathit{H}_B$); ^{13}C NMR (101 MHz, $CDCl_3$) δ 167.3 (C), 146.9 (C), 144.5 (C), 144.1 (C), 141.2 (C), 128.7 (CH), 128.6 (CH), 128.3 (2 × CH), 128.0 (CH), 127.6 (CH), 126.5 (CH), 126.3 (CH), 125.9 (CH), 125.5 (CH), 125.3 (CH), 124.2 (CH), 79.6 (C), 68.6 (C), 44.5 (CH), 33.3 (CH_2), 31.0 (CH_2), 29.2 (CH_3), 29.1 (CH_3); MS (ESI) *m/z* 511 [$(M + Na)^+$, 38], 489 [$(M + H)^+$, 100], 471 [$(M - OH)^+$, 52], 353 [$(M - OH - CMe_2Ph)^+$, 24], 157 [$(M + H_5 - 3 \times Ph - CH_2CH_2Ph)^+$, 31]; HRMS (ESI) *m/z* $C_{32}H_{32}N_4O$ ($[M + H]^+$) calcd for 489.2649, found 489.2631.

2-(2-Phenylpropan-2-yl)-5-(7,7,7-trifluorohept-1-en-4-yl)-2*H*-1,2,3,4-tetrazole 24

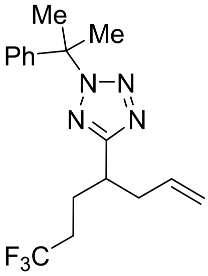

24

Using general procedure C, *n*-BuLi (0.46 mL of a 2.5 M solution in hexanes, 1.15 mmol, 2.3 eq) and **ST19** (149 mg, 0.5 mmol, 1.0 eq) in THF (4 mL) at 0 °C for 15 min then AllylBr (0.11 mL, 151 mg, 1.25 mmol, 2.5 eq) gave the crude product. Purification by flash column chromatography on silica with 95:5 petroleum ether-EtOAc as eluent gave tetrazole **24** (129 mg, 76%) as a colourless oil, R_F (95:5 petroleum ether-EtOAc) 0.2; IR (ATR) 3066, 2988, 2944, 1642, 1602, 1497, 1449, 1417, 1392, 1372, 1339, 1316, 1301, 1254, 1220, 1185, 1143, 1078, 1061, 1022, 985, 918 cm^{-1}; ^1H NMR (400 MHz, $CDCl_3$) δ 7.35–7.22 (m, 3H, Ph), 7.05–6.98 (m, 2H, Ph), 5.68 (dddd, *J* = 19.0, 9.5, 7.0, 7.0 Hz, 1H, $H_2C{=}C\mathit{H}$), 5.04–4.94 (m, 2H, $\mathit{H}_2C{=}CH$), 3.19 (dddd, *J* = 7.0, 7.0, 7.0, 7.0 Hz, 2H, TzCH), 2.63–2.53 (ddddd, *J* = 14.0, 7.0, 7.0, 1.0, 1.0 Hz, 1H, $C\mathit{H}_AH_BCH{=}CH_2$), 2.48 (ddddd, *J* = 14.0, 7.0, 7.0, 1.0, 1.0 Hz, 1H, $CH_A\mathit{H}_BCH{=}CH_2$), 2.15 (s, 6H,

CMe_2), 2.09 – 1.91 (m, 4H, $F_3CCH_2CH_2$); ^{13}C NMR (101 MHz, $CDCl_3$) δ 167.8 (C), 144.0 (C), 134.8 (CH), 128.6 (CH), 127.9 (CH), 127.0 (q, *J* = 276.5 Hz, C), 124.5 (CH), 117.6 (CH_2), 68.3 (C), 38.4 (CH_2), 35.7 (CH), 31.4 (q, *J* = 29.0 Hz, CH_2), 29.1 (CH_3), 29.0 (CH_3), 25.6 (q, J = 3.0 Hz, CH_2); ^{19}F NMR (376 MHz, $CDCl_3$) δ –66.46 (t, *J* = 10.0 Hz); MS (ESI) *m/z* 361 [(M + Na)$^+$, 41], 339 [(M + H)$^+$, 100], 221 [(M + H_2 – CMe_2Ph)$^+$, 83]; HRMS (ESI) *m/z* $C_{17}H_{22}F_3N_4$ ([M + H]$^+$) calcd for 339.1791, found 339.1796.

Using general procedure F, 1.1 M solution of tetrazole **ST19** in 1:1 PhMe/TMEDA (0.5 mL, 0.55 mmol, 1.0 eq) and a solution of 2.5 M *n*-BuLi in hexanes (0.5 mL, 1.15 mmol, 2.3 eq) were mixed for 30 s at rt followed by mixing with a 2.5 M allylBr solution in PhMe gave the crude product. Purification by flash column chromatography on silica with 95:5 petroleum ether-EtOAc as eluent gave tetrazole **24** (130 mg, 70%) as a colourless oil.

Methyl({4-[2-(2-phenylpropan-2-yl)-2H-1,2,3,4-tetrazol-5-yl]hept-6-en-1-yl})(triphenylmethyl)amine 25

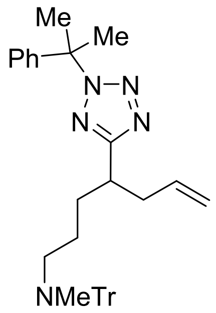

25

Using general procedure C, *n*-BuLi (0.46 mL of a 2.5 M solution in hexanes, 1.15 mmol, 2.3 eq) and **ST24** (258 mg, 0.5 mmol, 1.0 eq) in THF (4 mL) at 0 °C for 15 min then AllylBr (0.11 mL, 151 mg, 1.25 mmol, 2.5 eq) gave the crude product. Purification by flash column chromatography on silica with 95:5 petroleum ether-EtOAc as eluent gave tetrazole **25** (194 mg, 70%) as a colourless oil, R_F (95:5 petroleum ether-EtOAc) 0.2; IR (ATR) 3058, 3030, 2936, 2859, 2799, 1641, 1595, 1488, 1468, 1447, 1418, 1391, 1370, 1310, 1255, 1213, 1183, 1160, 1106, 1083, 1031, 1019, 1004, 907 cm^{-1}; ^{1}H NMR (400 MHz, $CDCl_3$) δ 7.49–7.40 (m, 6H, Ph), 7.34–7.18 (m, 9H, Ph), 7.17–7.08 (m, 3H, Ph), 7.05–6.97 (m, 2H, Ph), 5.69 (dddd, *J* = 17.0, 10.0, 7.0 Hz, 1H, H_2C=C*H*), 5.01–4.90 (m, 2H, *H*$_2$C=CH), 3.13 (dddd, *J* = 8.5, 8.5, 6.0, 6.0 Hz, 1H, TzCH),

2.62–2.43 (m, 2H, NCH_2), 2.14 (s, 6H, CMe_2), 2.06 (t, *J* = 7.5 Hz, 2H, CH_2), 1.94 (s, 3H, NMe), 1.83–1.69 (m, 2H, CH_2); ^{13}C NMR (101 MHz, $CDCl_3$) δ 169.0 (C), 146.9 (C), 144.4 (C), 143.1 (CH), 135.8 (CH), 129.4 (CH), 128.6 (CH), 128.0 (CH), 127.7 (CH), 127.3 (3 × CH), 125.8 (CH), 124.5 (CH), 116.8 (CH_2), 78.0 (C), 68.0 (C), 52.3 (CH_2), 38.6 (CH_2), 37.1 (CH), 36.8 (CH_3), 31.4 (CH_2), 29.2 (CH_3), 25.8 (CH_2); MS (ESI) *m/z* 556 [(M + H)$^+$, 60], 243 [(CPh_3)$^+$, 100], 169 [(M + H_4 – CMe_2Ph – Allyl – Ph_3)$^+$, 75]; HRMS (ESI) *m/z* $C_{37}H_{42}N_5$ ([M + H]$^+$) calcd for 556.3435, found 556.3448.

Using general procedure F, 1.1 M solution of tetrazole **ST24** in 1:1 PhMe/TMEDA (0.5 mL, 0.55 mmol, 1.0 eq) and a solution of 2.5 M *n*-BuLi in hexanes (0.5 mL, 1.15 mmol, 2.3 eq) were mixed for 30 s at rt followed by mixing with a 2.5 M allylBr solution in PhMe gave the crude product. Purification by flash column chromatography on silica with 95:5 petroleum ether-EtOAc as eluent gave tetrazole **25** (197 mg, 64%) as a colourless oil.

5-Methoxy-1,1-diphenyl-2-[2-(2-phenylpropan-2-yl)-2*H*-1,2,3,4-tetrazol-5-yl]pentan-1-ol 26

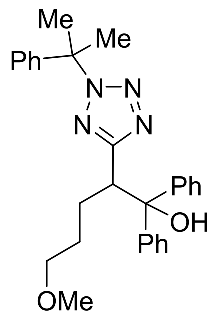

26

Using general procedure C, *s*-BuLi (0.6 mL of a 1.3 M solution in cyclohexane/hexanes, 0.78 mmol, 1.6 eq) and **ST25** (124 mg, 0.5 mmol, 1.0 eq) in THF (4 mL) at –78 °C for 15 min then a solution of Ph_2CO (228 mg, 1.25 mmol, 2.5 eq) in THF (2 mL) gave the crude product. Purification by flash column chromatography on silica with 80:20 petroleum ether-EtOAc as eluent gave tetrazole **26** (181 mg, 79%) as a white solid, m.p. 105–107 °C; R_F (80:20 petroleum ether-EtOAc) 0.2; IR (ATR) 3449 (O-H str), 3051, 3031, 2986, 2938, 2877, 1496, 1462, 1446, 1392, 1373, 1312, 1287, 1262, 1247, 1235, 1192, 1175, 1165, 1114, 1084, 1048, 1031, 1022, 1001, 985, 933, 916, 908 cm^{-1}; ^{1}H NMR (400 MHz, $CDCl_3$) δ 7.64–7.61 (m, 2H, Ph), 7.45–7.43 (m, 2H, Ph), 7.34–7.30 (m, 2H, Ph), 7.24–7.16 (m, 4H, Ph), 7.13–7.09 (m, 2H, Ph), 7.07–7.03 (m, 1H, Ph), 6.58–6.55 (m, 2H, Ph), 4.72 (s, 1H, OH), 4.34 (dd, *J* = 11.5, 3.0 Hz, 1H, TzCH), 3.25–

3.21 (m, 2H, OCH_2), 3.21 (s, 3H, OMe), 2.04 (s, 3H, C*Me*$_A$Me$_B$), 2.02 (s, 3H, CMe$_A$*Me*$_B$), 2.00–1.94 (m, 1H, CH$_A$*H*$_B$), 1.75–1.66 (m, 1H, CH$_A$*H*$_B$), 1.48–1.39 (m, 1H, CH$_A$*H*$_B$), 1.36–1.27 (m, 1H, CH$_A$*H*$_B$); ^{13}C NMR (101 MHz, $CDCl_3$) δ 167.3 (C), 147.0 (C), 144.6 (C), 144.0 (C), 128.7 (CH), 128.3 (CH), 128.0 (CH), 127.6 (CH), 126.6 (CH), 126.3 (CH), 125.6 (CH), 125.3 (CH), 124.2 (CH), 79.6 (C), 72.3 (CH_2), 68.6 (C), 58.4 (CH_3), 45.4 (CH), 29.3 (CH_3), 29.1 (CH_3), 27.5 (CH_2), 26.2 (CH_2); MS (ESI) *m/z* 479 [(M + Na)$^+$, 20], 457 [(M + H)$^+$, 100], 439 [(M – OH)$^+$, 30], 321 [(M – OH – CMe$_2$Ph)$^+$, 25], 157 [(M + H$_5$ – 3 × Ph – CH$_2$CH$_2$CH$_2$OMe)$^+$, 4]; HRMS (ESI) *m/z* $C_{28}H_{33}N_4O_2$ ([M + H]$^+$) calcd for 457.2598, found 457.2628.

3-(2-Methyl-1,3-dioxolan-2-yl)-1,1-diphenyl-2-[2-(2-phenylpropan-2-yl)-2*H*-1,2,3,4-tetrazol-5-yl]propan-1-ol 27

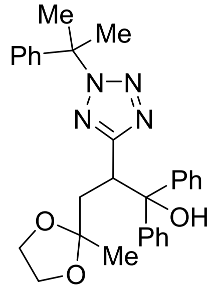

27

Using general procedure C, *s*-BuLi (1.0 mL of a 1.3 M solution in cyclohexane/hexanes, 1.3 mmol, 2.6 eq), LiCl (55 mg, 1.3 mmol, 2.6 eq) and **ST27** (151 mg, 0.5 mmol, 1.0 eq) in THF (4 mL) at –78 °C for 15 min then a solution of Ph_2CO (273 mg, 1.5 mmol, 3.0 eq) in THF (2 mL) gave the crude product. Purification by flash column chromatography on silica with 80:20 petroleum ether-EtOAc as eluent gave tetrazole **27** (121 mg, 50%) as a white solid, m.p. 110–113 °C; R$_F$ (80:20 petroleum ether-EtOAc) 0.2; IR (ATR) cm^{-1}; ^{1}H NMR (400 MHz, $CDCl_3$) δ 7.72–7.60 (m, 2H, Ph), 7.42–7.39 (m, 1H, Ph), 7.37–7.27 (m, 2H, Ph), 7.24–7.15 (m, 4H, Ph), 7.12–7.06 (m, 2H, Ph), 7.05–6.99 (m, 1H, Ph), 6.68–6.59 (m, 2H, Ph), 4.64 (s, 1H, OH), 4.61 (dd, *J* = 11.0, 2.0 Hz, 1H, TzCH), 3.88–3.76 (m, 2H, OC*H*$_A$H$_B$C*H*$_C$H$_D$O), 3.66 (ddd, *J* = 7.5, 6.5, 6.5 Hz, 1H, OCH$_A$*H*$_B$CH$_C$H$_D$O), 3.44 (ddd, *J* = 7.5, 7.0, 6.0 Hz, 1H, OCH$_A$H$_B$CH$_C$*H*$_D$O), 2.47 (dd, *J* = 15.0, 11.0 Hz, 1H, TzCHC*H*$_A$H$_B$), 2.13 (dd, *J* = 15.0, 2.5 Hz, 1H, TzCHCH$_A$*H*$_B$) 2.00 (s, 3H, C*Me*$_A$Me$_B$), 1.95 (s, 3H, CMe$_A$*Me*$_B$), 1.15 (s, 3H, OCMe); ^{13}C NMR (101 MHz, $CDCl_3$) δ 167.9 (C), 146.4 (C), 144.3

(C), 144.0 (C), 128.6 (CH), 128.4 (CH), 127.9 (CH), 127.5 (CH), 126.7 (CH), 126.2 (CH), 125.6 (CH), 125.5 (CH), 124.4 (CH), 109.4 (C), 80.1 (C), 68.3 (C), 64.6 (CH_2), 64.0 (CH_2), 40.4 (CH), 38.6 (CH_2), 29.3 (CH_3), 29.1 (CH_3), 24.4 (CH_3); MS (ESI) *m/z* 507 [$(M + Na)^+$, 31], 485 [$(M + H)^+$, 75], 491 [$(M - OH)^+$, 13], 423 [$(M - H - C(OCH_2CH_2O))^+$, 100], 305 [$(M - C(OCH_2CH_2O) - CMe_2Ph)^+$, 38] 185 [$(M + H_2 - CMe_2Ph - CPh_2OH)^+$, 6]; HRMS (ESI) *m/z* $C_{29}H_{33}N_4O_3$ ($[M + H]^+$) calcd for 485.2547, found 485.2552.

5-(1-phenylbut-3-en-1-yl)-2-(2-phenylpropan-2-yl)-2*H*-1,2,3,4-tetrazole 28 and 5-(4-Phenylhepta-1,6-dien-4-yl)-2-(2-phenylpropan-2-yl)-2*H*-1,2,3,4-tetrazole 28'

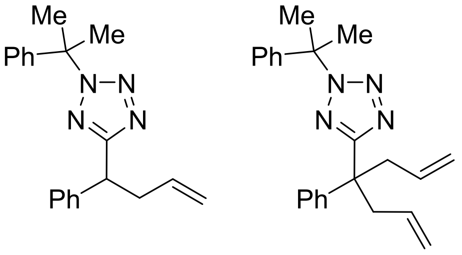

28 **28'**

Using general procedure C, *n*-BuLi (0.46 mL of a 2.5 M solution in hexanes, 1.15 mmol, 2.3 eq) and **ST28** (139 mg, 0.5 mmol, 1.0 eq) in THF (4 mL) at 0 °C for 15 min then AllylBr (0.11 mL, 151 mg, 1.25 mmol, 2.5 eq) gave the crude product. Purification by flash column chromatography on silica with 30:70 petroleum ether-CH_2Cl_2 as eluent gave tetrazole **28'** (62 mg, 35%) as a colourless oil, R_F (30:70 petroleum ether-CH_2Cl_2) 0.3; IR (ATR) 3063, 3027, 2981, 2927, 2859, 1639, 1600, 1497, 1466, 1445, 1415, 1391, 1369, 1313, 1291, 1256, 1188, 1164, 1107, 1074, 1026, 997, 915 cm^{-1}; ^1H NMR (400 MHz, $CDCl_3$) δ 7.32–7.23 (m, 6H, Ph), 7.21–7.15 (m, 2H, Ph), 7.04–6.97 (m, 2H, Ph), 5.50 (ddt, *J* = 17.5, 10.0, 7.0 Hz, 2H, $H_2C{=}CH$), 5.05–4.92 (m, 4H, $H_2C{=}CH$), 3.12–2.96 (m, 4H, $CH_2CH{=}CH_2$), 2.14 (s, 6H, CMe_2); ^{13}C NMR (101 MHz, $CDCl_3$) δ 171.0 (C), 144.4 (C), 144.0 (C), 133.6 (CH), 128.5 (CH), 128.1 (CH), 127.7 (CH), 127.1 (CH), 126.4 (CH), 124.6 (CH), 118.5 (CH_2), 68.1 (C), 45.8 (C), 41.1 (CH_2), 29.2 (CH_3); MS (ESI) *m/z* 381 [$(M + Na)^+$, 41], 359 [$(M + H)^+$, 88], 241 [$(M + H_2 - CMe_2Ph)^+$, 100]; HRMS (ESI) *m/z* $C_{23}H_{27}N_4$ ($[M + H]^+$) calcd for 359.2230, found 359.2247 and tetrazole **28** (91 mg, 57%) as a colourless

oil, R_F (30:70 petroleum ether-CH_2Cl_2) 0.2; IR (ATR) 3062, 3029, 2987, 2938, 1641, 1601, 1495, 1449, 1416, 1391, 1370, 1313, 1252, 1182, 1162, 1106, 1074, 1029, 1020, 993, 954, 916 cm^{-1}; ^1H NMR (400 MHz, $CDCl_3$) δ 7.40–7.34 (m, 2H, Ph), 7.33–7.25 (m, 5H, Ph), 7.21 (tt, *J* = 7.0, 1.5 Hz, 1H, Ph), 7.04–6.97 (m, 2H, Ph), 5.68 (dddd, J = 17.0, 10.0, 7.5, 6.5 Hz, 1H, $H_2C{=}CH$), 4.97 (dddd, *J* = 17.0, 1.5, 1.5, 1.5 Hz, 1H, $H_AH_BC{=}CH$), 4.92 (dddd, J = 10.0, 1.5, 1.5, 1.5 Hz, 1H, $H_AH_BC{=}CH$), 4.39 (dd, *J* = 9.0, 7.0 Hz, 1H, TzCH), 2.97 (ddddd, J = 14.0, 9.0, 7.5, 1.5, 1.5 Hz, 1H, $CH_AH_BCH{=}CH_2$), 2.80 (ddddd, J = 14.0, 9.0, 7.5, 1.5, 1.5 Hz, 1H, $CH_AH_BCH{=}CH_2$), 2.14 (s, 3H, CMe_AMe_B), 2.13 (s, 3H, CMe_AMe_B); ^{13}C NMR (101 MHz, $CDCl_3$) δ 167.9 (C), 144.2 (C), 141.3 (C), 135.5 (CH), 128.6 (CH), 128.5 (CH), 128.0 (CH), 127.7 (CH), 126.9 (CH), 124.6 (CH), 117.1 (CH_2), 68.2 (C), 43.3 (CH), 40.2 (CH_2), 29.2 (CH_3), 29.1 (CH_3); MS (ESI) *m/z* 341 [$(M + Na)^+$, 13], 319 [$(M + H)^+$, 58], 201 [$(M + H_2 - CMe_2Ph)^+$, 100], 167 [$(M + H_3 - Ph_3)^+$, 8]; HRMS (ESI) *m/z* $C_{20}H_{23}N_4$ ($[M + H]^+$) calcd for 319.1917, found 319.1933.

Using general procedure C, *n*-BuLi (0.32 mL of a 2.5 M solution in hexanes, 0.8 mmol, 1.6 eq) and **ST28** (139 mg, 0.5 mmol, 1.0 eq) in THF (4 mL) at 0 °C for 15 min then AllylBr (0.09 mL, 121 mg, 1.0 mmol, 2.0 eq) gave the crude product. Purification by flash column chromatography on silica with 30:70 petroleum ether-CH_2Cl_2 as eluent gave tetrazole **28** (121 mg, 76%) as a colourless oil.

Using general procedure F, 1.7 M solution of tetrazole **ST28** in 1:1 PhMe/TMEDA (0.5 mL, 0.85 mmol, 1.0 eq) and a solution of 2.5 M *n*-BuLi in hexanes (0.5 mL, 1.15 mmol, 2.3 eq) were mixed for 30 s at rt followed by mixing with a 2.5 M AllylBr solution in PhMe gave the crude product. Purification by flash column chromatography on silica with 30:70 petroleum ether-CH_2Cl_2 as eluent gave tetrazole **28** (191 mg, 71%) as a colourless oil.

3-(3-Chlorophenyl)-1,1-diphenyl-2-[2-(2-phenylpropan-2-yl)-2*H*-1,2,3,4-tetrazol-5-yl]propan-1-ol 30 and 2-(3-chlorophenyl)-1,1-diphenyl-3-[2-(2-phenylpropan-2-yl)-2H-1,2,3,4-tetrazol-5-yl]propan-1-ol 31

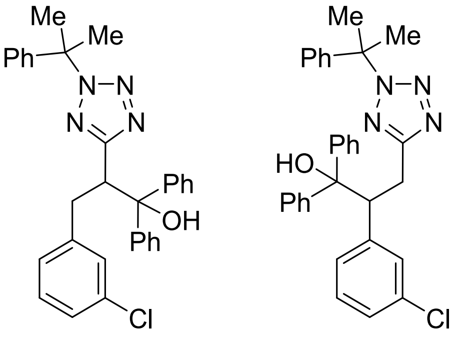

30 **31**

Using general procedure C, *s*-BuLi (0.6 mL of a 1.3 M solution in cyclohexane/hexanes, 0.78 mmol, 1.6 eq) and **29** (163 mg, 0.5 mmol, 1.0 eq) in THF (4 mL) at –78 °C for 15 min then a solution of Ph_2CO (228 mg, 1.25 mmol, 2.5 eq) in THF (2 mL) gave the crude product. Purification by flash column chromatography on silica with 95:5 petroleum ether-EtOAc as eluent gave tetrazole **30** (135 mg, 53%) as a white solid, m.p. 123–126 °C; R_F (95:5 petroleum ether-EtOAc) 0.2; IR (ATR) 3452 (O-H str), 3063, 3029, 2988, 2939, 1596, 1572, 1493, 1447, 1389, 1370 ,1313, 1269, 1243, 1187, 1160, 1146, 1102, 1080, 1062, 1027, 1001, 947, 907 cm^{-1}; ^1H NMR (400 MHz, $CDCl_3$) δ 7.83–7.51 (m, 2H, Ph), 7.41–7.37 (m, 2H, Ph), 7.35–7.27 (m, 2H, Ph), 7.22–7.08 (m, 3H, Ph), 7.07–6.90 (m, 4H, Ph, Ar), 6.72–6.55 (m, 2H, Ar), 6.52–6.41 (m, 2H, Ph), 4.78 (s, 1H, OH), 4.46 (dd, *J* = 11.5, 3.5 Hz, 1H, TzCH), 3.13 (dd, *J* = 13.0, 11.5 Hz, 1H, ArC$H_A$$H_B$), 2.86 (dd, *J* = 13.0, 3.5 Hz, 1H, ArC$H_A$$H_B$), 1.86 (s, 3H, C$Me_A$$Me_B$), 1.80 (s, 3H, C$Me_A$$Me_B$); ^{13}C NMR (101 MHz, $CDCl_3$) δ 166.3 (C), 146.5 (C), 144.3 (C), 143.8 (C), 141.5 (C), 133.8 (C), 129.4 (CH), 129.1 (CH), 128.6 (2 × CH), 128.1 (CH), 127.6 (CH), 127.2 (CH), 126.9 (CH), 126.5 (CH), 126.3 (CH), 125.7 (CH), 125.3 (CH), 124.2 (CH), 79.3 (C), 68.6 (C), 47.9 (CH), 35.8 (CH_2), 29.3 (CH_3), 29.0 (CH_3); MS (ESI) *m/z* 531 [$(M + Na)^+$, 13], 509 [$(M + H)^+$, 100], 491 [$(M – OH)^+$, 40], 373 [$(M – OH – CMe_2Ph)^+$, 23]; HRMS (ESI) *m/z* $C_{31}H_{30}N_4OCl$ ($[M + H]^+$) calcd for 509.2103, found 509.2122 and tetrazole **31** (13 mg, 11%) as a white solid m.p. 156–159 °C; R_F (95:5 petroleum ether-EtOAc) 0.3; IR (ATR) 3461 (O-H str), 3056, 3034, 2987,1597, 1574, 1469, 1475, 1447, 1433, 1392, 1370, 1338, 1306, 1278, 1242, 1167, 1080, 1025, 999, 973, 950, 912 cm^{-1}; 1H NMR (400 MHz, $CDCl_3$) δ 7.77–7.67 (m, 2H, Ph), 7.43–7.35 (m, 2H, Ph), 7.29–7.23 (m, 3H, Ph), 7.22–7.17 (m, 3H, Ph), 7.16–7.09 (m, 3H, Ph, Ar), 7.07–7.00 (m, 1H, Ar), 7.00–6.95 (m, 2H, Ph), 6.93–6.87 (m, 1H, Ar), 6.73–6.68 (m, 2H, Ph), 4.47 (dd, *J* = 11.5, 3.5 Hz, 1H, TzCH), 3.50 (dd, J = 15.0, 11.5 Hz, 1H, ArC$H_A$$H_B$), 3.28 (dd, J = 15.0, 3.5 Hz, 1H, ArC$H_A$$H_B$), 2.83 (s, 1H, OH), 1.98 (s, 3H, C$Me_A$$Me_B$), 1.97 (s, 3H, C$Me_A$$Me_B$); ^{13}C NMR (101 MHz, $CDCl_3$) δ 164.7 (C),

145.6 (C), 145.2 (C), 144.1 (C), 140.8 (C), 133.3 (C), 130.4 (CH), 128.7 (CH), 128.6 (2 × CH), 128.5 (CH), 127.9 (CH), 127.6 (CH), 127.1 (CH), 126.7 (CH), 126.6 (CH), 126.1 (CH), 125.6 (CH), 124.4 (CH), 80.5 (C), 68.0 (C), 52.4 (CH), 29.1 (CH_3), 29.0 (CH_3), 27.3 (CH_2). MS (ESI) *m/z* 531 [$(M + Na)^+$, 22], 509 [$(M + H)^+$, 100], 491 [$(M – OH)^+$, 20], 373 [$(M – OH – CMe_2Ph)^+$, 35]; HRMS (ESI) *m/z* $C_{31}H_{30}N_4OCl$ ($[M + H]^+$) calcd for 509.2103, found 509.2104.

NOSEY of **30**:

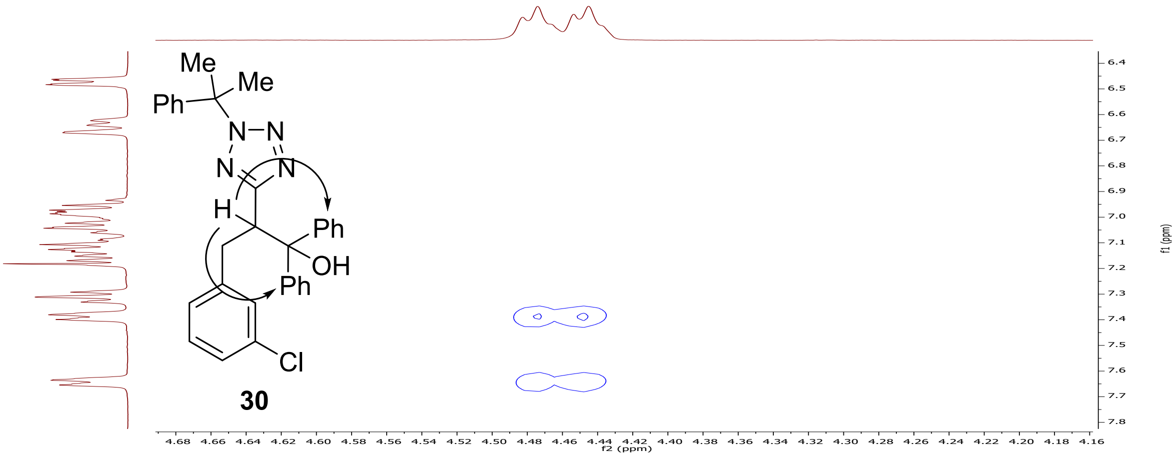

NOSEY of **31**:

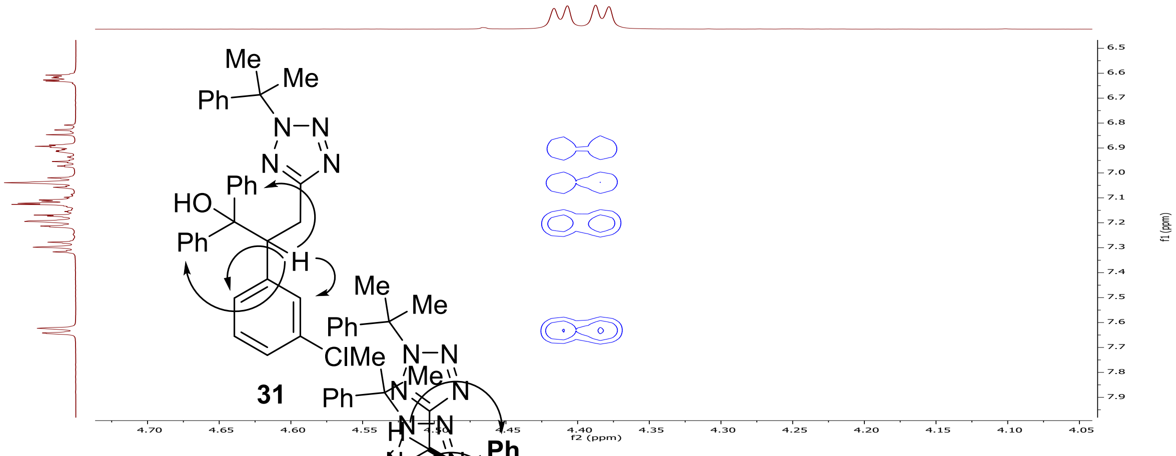

X-ray structure of **31**:

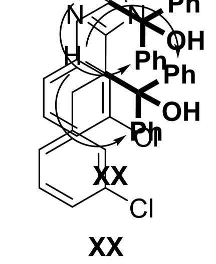

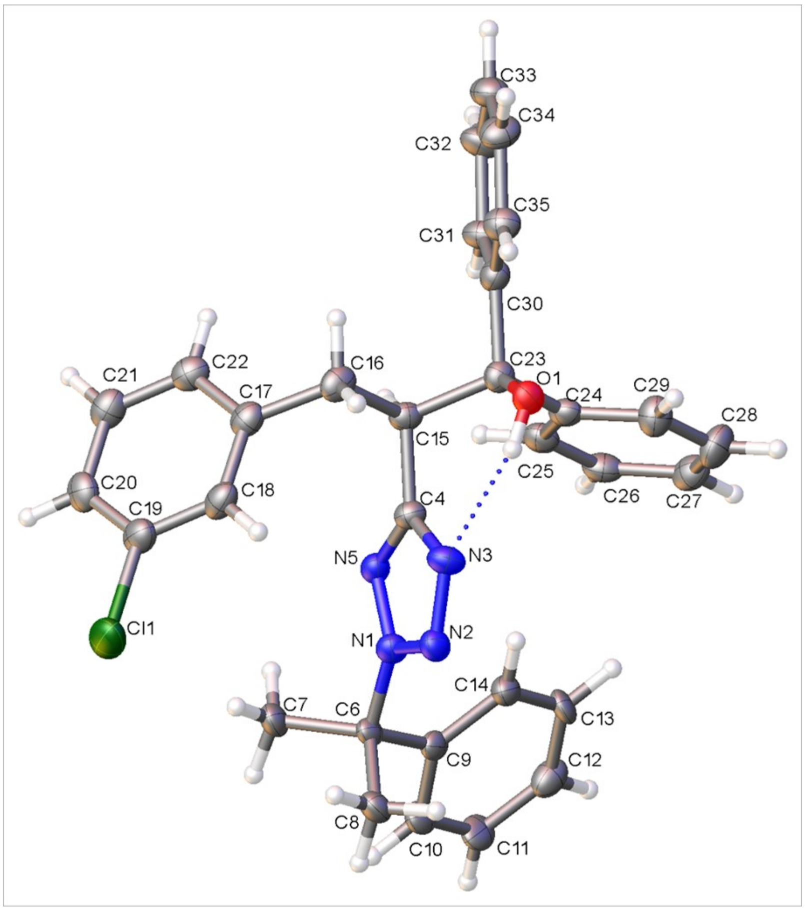

1-Methyl-3-[2-(2-phenylpropan-2-yl)-2*H*-1,2,3,4-tetrazol-5-yl]pyrrolidin-2-one 35

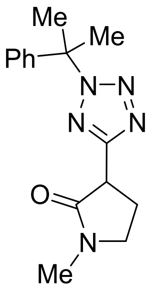

35

Using general procedure D, *s*-BuLi (0.77 mL of a 1.3 M solution in cyclohexane/hexanes, 1.0 mmol, 2.0 eq) and **32** (180 mg, 0.5 mmol, 1.0 eq) in THF (4 mL) gave the crude product. Purification by flash column chromatography on silica with 10:90 petroleum ether-EtOAc as eluent gave tetrazole **35** (115 mg, 80%) as a colourless oil, R_F (10:90 petroleum ether-CH_2Cl_2)

0.2; IR (ATR) 3092, 2899, 2940, 2881, 1689 (C=O str), 1601, 1495, 1448, 1434, 1403, 1371, 1301, 1281, 1254, 1185, 1161, 1106, 1077, 1021, 984, 958, 911 cm^{-1}; ^{1}H NMR (400 MHz, $CDCl_3$) δ 7.35–7.22 (m, 3H, Ph), 7.12–7.06 (m, 2H, Ph), 4.06 (ddd, *J* = 9.0, 8.5, 1.0 Hz, 1H, TzCH), 3.59 (ddd, *J* = 9.5, 8.0, 5.0 Hz, 1H, NCH_AH_B ($C\mathit{H}_A$)), 3.46 (ddd, *J* = 9.5, 7.5, 7.5 Hz, 1H, $NCH_A\mathit{H}_B$), 2.92 (s, 3H, NMe), 2.58–2.44 (m, 2H, $CHC\mathit{H}_2$), 2.15 (s, 3H, $C\mathit{Me}_AMe_B$), 2.15 (s, 3H, $CMe_A\mathit{Me}_B$); ^{13}C NMR (101 MHz, $CDCl_3$) δ 171.5 (C), 164.4 (C), 143.9 (C), 128.7 (CH), 127.8 (CH), 124.8 (CH), 68.4 (C), 47.8 (CH_2), 40.0 (CH), 30.2 (CH_3), 29.2 (CH_3), 29.1 (CH_3), 25.0 (CH_2); MS (ESI) *m/z* 308 [(M + Na)$^+$, 37], 286 [(M + H)$^+$, 100], 168 [(M + H_2 – CMe_2Ph)$^+$, 77]; HRMS (ESI) *m/z* $C_{15}H_{20}N_5O$ ([M + H]$^+$) calcd for 286.1662, found 286.1670.

1-Methyl-3-[2-(2-phenylpropan-2-yl)-2*H*-1,2,3,4-tetrazol-5-yl]piperidin-2-one 36

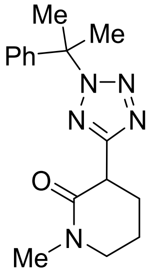

36

Using general procedure D, *s*-BuLi (1.25 mL of a 1.3 M solution in cyclohexane/hexanes, 1.6 mmol, 3.2 eq) and **33** (187 mg, 0.5 mmol, 1.0 eq) in THF (4 mL) gave the crude product. Purification by flash column chromatography on silica with EtOAc as eluent gave tetrazole **36** (115 mg, 80%) as a colourless oil, R_F (EtOAc) 0.2; IR (ATR) 2989, 2940, 2870, 1644, 1497, 1465, 1447, 1401, 1371, 1355, 1328, 1273, 1243, 1230, 1186, 1162, 1126, 1106, 1022, 1001, 969, 909 cm^{-1}; 1H NMR (400 MHz, $CDCl_3$) δ 7.34–7.21 (m, 3H, Ph), 7.09–7.04 (m, 2H, Ph), 4.07 (dd, *J* = 8.5, 6.0 Hz, 1H, TzCH), 3.47 (ddd, *J* = 12.0, 8.5, 5.0 Hz, 1H, $NC\mathit{H}_AH_B$), 3.36 (ddd, *J* = 12.0, 5.5, 5.5 Hz, 1H, $NCH_A\mathit{H}_B$), 2.99 (s, 3H, NMe), 2.29–2.16 (m, 2H, CH_2), 2.15 (s, 3H, $C\mathit{Me}_AMe_B$), 2.14 (s, 3H, $CMe_A\mathit{Me}_B$), 2.11–2.01 (m, 1H, $C\mathit{H}_AH_B$), 1.93–1.81 (m, 1H, $CH_A\mathit{H}_B$); ^{13}C NMR (101 MHz, $CDCl_3$) δ 167.3 (C), 165.5 (C), 144.1 (C), 128.6 (CH), 127.7 (CH), 124.7 (CH), 68.3 (C), 50.1 (CH_2), 40.4 (CH), 35.2 (CH_3), 29.3 (CH_3), 29.2 (CH_3), 27.7 (CH_2), 21.3 (CH_2); MS (ESI) *m/z* 322 [(M + Na)$^+$, 12], 300 [(M + H)$^+$, 100], 182 [(M + H_2 – CMe_2Ph)$^+$, 36]; HRMS (ESI) *m/z* $C_{16}H_{22}N_5O$ ([M + H]$^+$) calcd for 300.1819, found 300.1838.

1-Methyl-3-[2-(2-phenylpropan-2-yl)-2*H*-1,2,3,4-tetrazol-5-yl]azepan-2-one 37

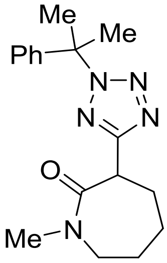

37

Using general procedure D, *s*-BuLi (0.77 mL of a 1.3 M solution in cyclohexane/hexanes, 1.0 mmol, 2.0 eq) and **34** (194 mg, 0.5 mmol, 1.0 eq) in THF (4 mL) gave the crude product. Purification by flash column chromatography on silica with 30:70 petroleum ether-EtOAc as eluent gave tetrazole **37** (97 mg, 62%) as a colourless oil, R_F (30:70 petroleum ether-EtOAc) 0.2; IR (ATR) 3060, 2989, 2933, 2863, 1645 (C=O str), 1488, 1447, 1432, 1392, 1370, 1336, 1317, 1296, 1258, 1218, 1202, 1186, 1156, 1113, 1078, 1024, 973, 995, 941, 922, 911 cm^{-1}; 1H NMR (400 MHz, $CDCl_3$) δ 7.35–7.21 (m, 3H, Ph), 7.09–7.04 (m, 2H, Ph), 4.33 (dd, *J* = 10.5, 2.5 Hz, 1H, TzCH), 3.67 (dd, *J* = 15.5, 11.0 Hz, 1H, NCH_AH$_B$), 3.26 (dddd, *J* = 15.5, 6.0, 1.5, 1.5 Hz, 1H, NCH$_A$$H_B$), 3.02 (s, 3H, NMe), 2.28–2.10 (m, 2H, CH_2), 2.16 (s, 3H, CMe_AMe$_B$), 2.15 (s, 3H, CMe$_A$$Me_B$), 2.09–1.98 (m, 1H, CH_2), 1.84 (dddd, *J* = 14.0, 4.5, 4.5, 4.5 Hz, 1H, CH_2), 1.77–1.63 (m, 1H, CH_2), 1.57 (ddddd, *J* = 16.0, 14.0, 11.5, 3.5, 2.0 Hz, 1H, CH_2); ^{13}C NMR (101 MHz, $CDCl_3$) δ 172.7 (C), 165.7 (C), 144.3 (C), 128.6 (CH), 127.7 (CH), 124.7 (CH), 68.2 (C), 50.7 (CH_2), 43.2 (CH), 36.3 (CH_3), 29.3 (2 × CH_3), 28.7 (CH_2), 28.2 (CH_2), 27.1 (CH_2); MS (ESI) *m/z* 336 [(M + Na)$^+$, 25], 314 [(M + H)$^+$, 100], 196 [(M + H_2 – CMe_2Ph)$^+$, 80]; HRMS (ESI) *m/z* $C_{17}H_{24}N_5O$ ([M + H]$^+$) calcd for 314.1975, found 314.1986.

5-(Butan-2-yl)-1*H*-1,2,3,4-tetrazole 38

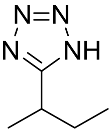

38

10% palladium on carbon (56 mg, 0.05 mmol, 20 mol%) were added to a stirred solution of tetrazole **13** (65 mg, 0.27 mmol) and KCO_2H (115 mg, 1.60 mmol, 6.0 eq) in EtOH (1.5 mL) at rt. The resulting solution was stirred and heated at 80 °C for 1 h before being allowed to cool to rt. The resulting solution was filtered over Celite®, washing with EtOAc (2 × 5 mL) and the filtrate evaporated under reduced pressure to give the crude product. Purification by flash

column chromatography on silica with 9:1 CH_2Cl_2:MeOH as eluent gave **38** (32 mg, 94%) as a colourless oil, R_F = 0.4 (9:1 CH_2Cl_2:MeOH); IR ($CHCl_3$) 2970 (N-H str), 2934, 2878, 2762, 2619, 1555, 1462, 1074, 1020, 1003, 962, 757 cm^{-1}; ^1H NMR (300 MHz, $CDCl_3$) δ 9.72 (s, 1H, NH), 3.34 (tq, J = 7.0, 7.0 Hz, 1H, TzCH), 2.25–1.73 (m, 2H, CH_2), 1.48 (d, J = 7.0 Hz, 3H, CH*Me*), 0.92 (t, J = 7.5 Hz, 3H, CH_2*Me*); ^{13}C NMR (75 MHz, $CDCl_3$) δ 161.0 (C), 31.4 (CH), 28.8 (CH_2), 18.7 (CH_3), 11.5 (CH_3); MS (ESI) *m/z* 149 [(M + Na)$^+$, 86], 127 [(M + H)$^+$, 100]; HRMS (ESI) *m/z* $C_5H_{11}N_4$ ([M + H)$^+$] calcd for 127.0978, found 127.0980.

5-(1-Phenylhex-5-en-3-yl)-2-(2-phenylpropan-2-yl)-2*H*-1,2,3,4-tetrazole 39

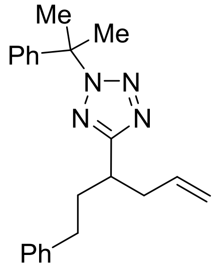

39

Using general procedure F, 1.1 M solution of tetrazole **ST18** in 1:1 PhMe/TMEDA (0.5 mL, 0.55 mmol, 1.0 eq) and a solution of 2.5 M *n*-BuLi in hexanes (0.5 mL, 1.15 mmol, 2.3 eq) were mixed for 30 s at rt followed by mixing with a 2.5 M allylBr solution in PhMe gave the crude product. Purification by flash column chromatography on silica with 93:7 petroleum ether-EtOAc as eluent gave tetrazole **39** (179 mg, 94%) as a colourless oil, R_F (93:7 petroleum ether-EtOAc) 0.2; IR (ATR) 3062, 3026, 2987, 2938, 2859, 1461, 1602, 1496, 1449, 1416, 1391, 1370, 1309, 1254, 1186, 1162, 1107, 1077, 1030, 1019, 992, 912 cm^{-1}; ^1H NMR (400 MHz, $CDCl_3$) δ 7.34–7.21 (m, 5H, Ph), 7.16 (tt, *J* = 7.5, 1.5 Hz, 1H, Ph), 7.11–7.07 (m, 2H, Ph), 7.06–7.01 (m, 2H, Ph), 5.68 (dddd, *J* = 17.5, 10.5, 7.0, 7.0 Hz, 1H, H_2C=C*H*), 4.98–4.91 (m, 2H, H_2C=CH), 3.18 (dddd, J = 9.0, 7.5, 6.5, 5.0 Hz, 1H, TzCH), 2.62–2.44 (m, 4H, CH_2), 2.16 (s, 6H, CMe_2), 2.14–1.99 (m, 2H, CH_2); ^{13}C NMR (101 MHz, $CDCl_3$) δ 168.8 (C), 144.3 (C), 141.9 (C), 135.6 (CH), 128.6 (CH), 128.5 (CH), 128.33 (CH), 127.7 (CH), 125.8 (CH), 124.6 (CH), 116.9 (CH_2), 68.0 (C), 38.7 (CH), 36.3 (CH_2), 35.5 (CH_2), 33.4 (CH_2), 29.1 (CH_3); MS (ESI) *m/z* 369 [(M + Na)$^+$, 20], 347 [(M + H)$^+$, 72], 229 [(M + H_2 – CMe_2Ph)$^+$, 100]; HRMS (ESI) *m/z* $C_{22}H_{27}N_4$ ([M + H]$^+$) calcd for 347.2230, found 347.2252.

5-[1-(3-Chlorophenyl)pent-4-en-2-yl]-2-(2-phenylpropan-2-yl)-2*H*-1,2,3,4-tetrazole 40

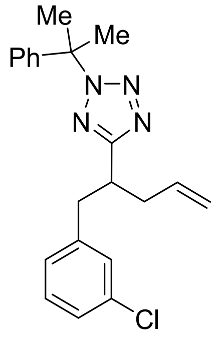

40

Using general procedure F, 1.1 M solution of tetrazole **29** in 1:1 PhMe/TMEDA (0.5 mL, 0.55 mmol, 1.0 eq) and a solution of 2.5 M *n*-BuLi in hexanes (0.5 mL, 1.15 mmol, 2.3 eq) were mixed for 30 s at rt followed by mixing with a 2.5 M AllylBr solution in PhMe gave the crude product. Purification by flash column chromatography on silica with 90:10 petroleum ether-EtOAc as eluent gave tetrazole **40** (137 mg, 68%) as a colourless oil, R_F (90:10 petroleum ether-EtOAc) 0.2; IR (ATR) 3063, 2987, 2933, 2860, 1641, 1599, 1573, 1497, 1477, 1447, 1429, 1391, 1313, 1255, 1207, 1187, 1163, 1104, 1078, 1020, 998, 992, 956, 916 cm^{-1}; ^{1}H NMR (400 MHz, $CDCl_3$) δ 7.33–7.24 (m, 4H, Ar, Ph), 7.15–7.06 (m, 2H, Ar), 7.03 (d, *J* = 2.0 Hz, 1H, Ar), 6.94–6.89 (m, 2H, Ph), 5.71 (dddd, *J* = 17.0, 10.0, 6.5, 6.5 Hz, 1H, $H_2C{=}CH$), 5.04–4.94 (m, 2H, $H_2C{=}CH$), 3.43 (tt, *J* = 8.0, 6.5 Hz, 2H, TzCH), 3.06 (dd, *J* = 8.0, 2.0 Hz, 2H, $ArCH_2$), 2.64–2.44 (m, 2H, $H_2CH_2C{=}CH$), 2.09 (s, 3H, CMe_AMe_B), 2.09 (s, 3H, CMe_AMe_B); ^{13}C NMR (101 MHz, $CDCl_3$) δ 167.8 (C), 144.1 (C), 141.4 (C), 135.2 (CH), 133.9 (C), 129.4 (CH), 129.2 (CH), 128.6 (CH), 127.7 (CH), 127.3 (CH), 126.4 (CH), 124.5 (CH), 117.4 (CH_2), 68.1 (C), 39.4 (CH_2), 38.6 (CH), 38.4 (CH_2), 29.2 (2 × CH_3); MS (ESI) *m/z* 388 [$(M + Na)^+$, 18], 367 [$(M + H)^+$, 64], 249 [$(M + H_2 – CMe_2Ph)^+$, 100]; HRMS (ESI) *m/z* $C_{21}H_{24}ClN_4$ ($[M + H]^+$) calcd for 367.1684, found 367.1688.

5-[1-(2-Methyl-1,3-dioxolan-2-yl)pent-4-en-2-yl]-2-(2-phenylpropan-2-yl)-2*H*-1,2,3,4-tetrazole 41

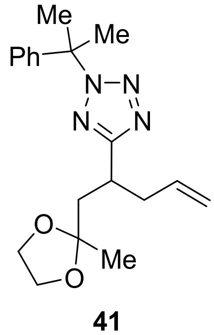

41

Using general procedure F, 1.1 M solution of tetrazole **ST27** in 1:1 PhMe/TMEDA (0.5 mL, 0.55 mmol, 1.0 eq) and a solution of 2.5 M *n*-BuLi in hexanes (0.5 mL, 1.15 mmol, 2.3 eq) were mixed for 30 s at rt followed by mixing with a 2.5 M AllylBr solution in PhMe gave the crude product. Purification by flash column chromatography on silica with 80:20 petroleum ether-EtOAc as eluent gave tetrazole **41** (123 mg, 65%) as a colourless oil, R_F (80:20 petroleum ether-EtOAc) 0.2; IR (ATR) 3064, 2982, 2938, 1720, 1641, 1602, 1467, 1448, 1417, 1371, 1310, 1252, 1218, 1186, 1161, 1147, 1106, 1076, 1044, 1020, 993, 948 cm^{-1}; ^1H NMR (400 MHz, $CDCl_3$) δ 7.37–7.20 (m, 3H, Ph), 7.08–7.01 (m, 2H, Ph), 5.76–5.59 (m, 1H, $H_2C{=}CH$), 5.02–4.89 (m, 2H, $H_2C{=}CH$), 3.88–3.73 (m, 3H, $OCH_AH_BCH_CH_DO$), 3.72–3.65 (m, 1H, $OCH_AH_BCH_CH_DO$), 3.40 (dddd, J = 10.0, 7.0, 7.0, 3.5 Hz, 1H, TzCH), 2.48 (dddd, J = 7.0, 7.0, 1.5, 1.5 Hz, 2H, $H_2CH_2C{=}CH$), 2.41 (dd, J = 14.5, 10.0 Hz, 1H, CCH_AH_B), 2.15 (s, 6H, CMe_2), 2.07 (dd, J = 14.5, 3.5 Hz, 1H, CCH_AH_B), 1.26 (s, 3H, CMe); ^{13}C NMR (101 MHz, $CDCl_3$) δ 169.5 (C), 144.5 (C), 135.5 (CH), 128.5 (CH), 127.6 (CH), 124.7 (CH), 117.1 (CH), 109.4 (CH_2), 67.9 (C), 64.5 (CH_2), 64.3 (CH_2), 42.1 (CH_2), 40.2 (CH_2), 32.2 (CH), 29.2 (2 × CH_3), 24.4 (CH_3); MS (ESI) *m/z* 365 [$(M + Na)^+$, 45], 343 [$(M + H)^+$, 95], 279 [$(M - H_3 - OCH_2CH_2O)^+$, 21], 225 [$(M + H_2 - CMe_2Ph)^+$, 100], 181 [$(M + H - CMe_2Ph - OCH_2CH_2)^+$, 16]; HRMS (ESI) *m/z* $C_{19}H_{27}N_4O_2$ ($[M + H]^+$) calcd for 343.2129, found 343.2147.

5-(7-Methoxyhept-1-en-4-yl)-2-(2-phenylpropan-2-yl)-2*H*-1,2,3,4-tetrazole 42

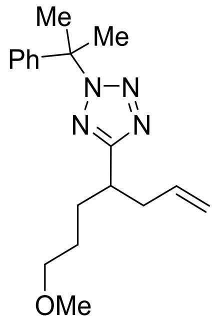

42

Using general procedure F, 1.1 M solution of tetrazole **ST25** in 1:1 PhMe/TMEDA (0.5 mL, 0.55 mmol, 1.0 eq) and a solution of 2.5 M *n*-BuLi in hexanes (0.5 mL, 1.15 mmol, 2.3 eq) were mixed for 30 s at rt followed by mixing with a 2.5 M AllylBr solution in PhMe gave the crude product. Purification by flash column chromatography on silica with 80:20 petroleum ether-

EtOAc as eluent gave tetrazole **42** (130 mg, 75%) as a colourless oil, R_F (80:20 petroleum ether-EtOAc) 0.2; IR (ATR) 3064, 2985, 2929, 2864, 2827, 1641, 1497, 1448, 1416, 1391, 1371, 1310, 1254, 1207, 1186, 1163, 1116, 1079, 1019, 994, 914 cm^{-1}; ^{1}H NMR (400 MHz, $CDCl_3$) δ 7.32–7.22 (m, 3H, Ph), 7.03–6.97 (m, 2H, Ph), 5.68 (dddd, *J* = 17.0, 10.0, 7.0, 7.0 Hz, 1H, $H_2C{=}CH$), 5.03 – 4.85 (m, 2H, $H_2C{=}CH$), 3.32 (m, 2H, OCH_2), 3.27 (s, 3H, OMe), 3.14 (dddd, *J* = 8.0, 8.0, 6.5, 6.5 Hz, 1H, TzCH), 2.61–2.34 (m, 2H, CH_2, $H_2C{=}CH$), 2.13 (s, 6H, CMe_2), 1.87–1.79 (m, 2H, CH_2), 1.57–1.39 (m, 2H); ^{13}C NMR (101 MHz, $CDCl_3$) δ 168.8 (C), 144.3 (C), 135.7 (CH), 128.6 (CH), 127.7 (CH), 124.6 (CH), 116.8 (CH_2), 72.4 (CH_2), 68.0 (C), 58.5 (CH_3), 38.7 (CH_2), 36.6 (CH), 30.2 (CH_2), 29.2 (CH_3), 29.1 (CH_3), 27.2 (CH_2); MS (ESI) *m/z* 337 [$(M + Na)^+$, 22], 315 [$(M + H)^+$, 69], 197 [$(M + H_2 - CMe_2Ph)^+$, 100], 165 [$(M + H - CMe_2Ph - OMe)^+$, 34]; HRMS (ESI) *m/z* $C_{18}H_{27}N_4O$ ($[M + H]^+$) calcd for 315.2179, found 315.2195.

1-Diphenyl-2-(1*H*-1,2,3,4-tetrazol-5-yl)butan-1-ol 42

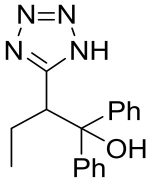

42

Pd/C (10% w/w, 108 mg, 0.1 mmol, 20 mol%) was added into a stirred solution of tetrazole **7** (206 mg, 0.5 mmol, 1.0 eq) and KCO_2H (252 mg, 3 mmol, 6.0 eq) in EtOH (2.5 mL) and the resulting suspension was stirred and heated at 80 °C for 1 h. After cooling to rt, the suspension was filtered through Celite© and washed with EtOAc (2 × 10 mL). The filtrate was evaporated under reduced pressure to give the crude product. Purification by flash column chromatography on silica with 60:40 petroleum ether-EtOAc as eluent gave tetrazole **42** (125 mg, 75%) as a white solid. m.p. 124-127 °C; R_F (60:40 petroleum ether-EtOAc) 0.4; IR (ATR) 3486 (O-H str), 3310, 3059, 2988, 2876, 2360, 2342, 1548 , 1448, 1174, 1049, 876 cm^{-1}; ^{1}H NMR (400 MHz, $CDCl_3$) δ 7.65 (dd, *J* = 8.5, 1.0 Hz, 2H, Ph), 7.47–7.42 (m, 4H, Ph), 7.31–7.27 (m, 1H, Ph), 7.12–7.01 (m, 3H, Ph), 4.60 (dd, *J* = 10.5, 4.0 Hz, 1H, TzCH), 4.40 (br s, 1H, OH), 1.97–1.87 (m, 2H, CH_2), 0.79 (t, *J* = 7.5 Hz, 3H, Me); ^{13}C NMR (101 MHz, $CDCl_3$) δ 157.4 (C), 145.0 (C), 144.2 (C), 128.7 (CH), 128.4 (CH), 127.3 (CH), 127.1 (CH), 125.4 (CH), 125.0 (CH), 79.9 (C), 46.6 (CH), 23.1 (CH_2), 12.0 (CH_3). Spectroscopic data consistent with those reported in the literature.[2]

Pd/C (10% w/w, 54 mg, 0.05 mmol, 10 mol%) was added into a stirred solution of tetrazole **7** (206 mg, 0.5 mmol, 1.0 eq) and KCO_2H (252 mg, 3 mmol, 6.0 eq) in EtOH (2.5 mL) and the resulting suspension was stirred and heated at 80 °C for 1 h. After cooling to rt, the suspension was filtered through Celite© and washed with EtOAc (2 × 10 mL). The filtrate was evaporated under reduced pressure to give the crude product. Purification by flash column chromatography on silica with 60:40 petroleum ether-EtOAc as eluent gave tetrazole **42** (114 mg, 78%) as a white solid.

Pd/C (10% w/w, 27 mg, 0.025 mmol, 5 mol%) was added into a stirred solution of tetrazole **7** (206 mg, 0.5 mmol, 1.0 eq) and KCO_2H (252 mg, 3 mmol, 6.0 eq) in EtOH (2.5 mL) and the resulting suspension was stirred and heated at 80 °C for 4 h. After cooling to rt, the suspension was filtered through Celite© and washed with EtOAc (2 × 10 mL). The filtrate was evaporated under reduced pressure to give the crude product. Purification by flash column chromatography on silica with 60:40 petroleum ether-EtOAc as eluent gave tetrazole **42** (110 mg, 75%) as a white solid.

2. Single crystal x-ray data

Identification code	jwb192p3
Empirical formula	$C_{31}H_{29}ClN_4O$
Formula weight	509.03
Temperature/K	100.0
Crystal system	orthorhombic
Space group	$Pca2_1$
a/Å	17.7597(7)
b/Å	14.3832(5)
c/Å	10.4096(4)
α/°	90
β/°	90

γ/°	90
Volume/Å^3	2659.04(17)
Z	4
ρ_{calc}g/cm^3	1.272
μ/mm^{-1}	1.511
F(000)	1072.0
Crystal size/mm^3	0.36 × 0.18 × 0.06
Radiation	CuKα (λ = 1.54178)
2Θ range for data collection/°	6.144 to 149.206
Index ranges	-22 ≤ h ≤ 20, -17 ≤ k ≤ 17, -12 ≤ l ≤ 13
Reflections collected	37580
Independent reflections	5421 [R_{int} = 0.0802, R_{sigma} = 0.0492]
Data/restraints/parameters	5421/1/338
Goodness-of-fit on F^2	1.195
Final R indexes [I>=2σ (I)]	R_1 = 0.0735, wR_2 = 0.1876
Final R indexes [all data]	R_1 = 0.0749, wR_2 = 0.1885
Largest diff. peak/hole / e Å^{-3}	0.35/-0.39
Flack parameter	0.039(16)

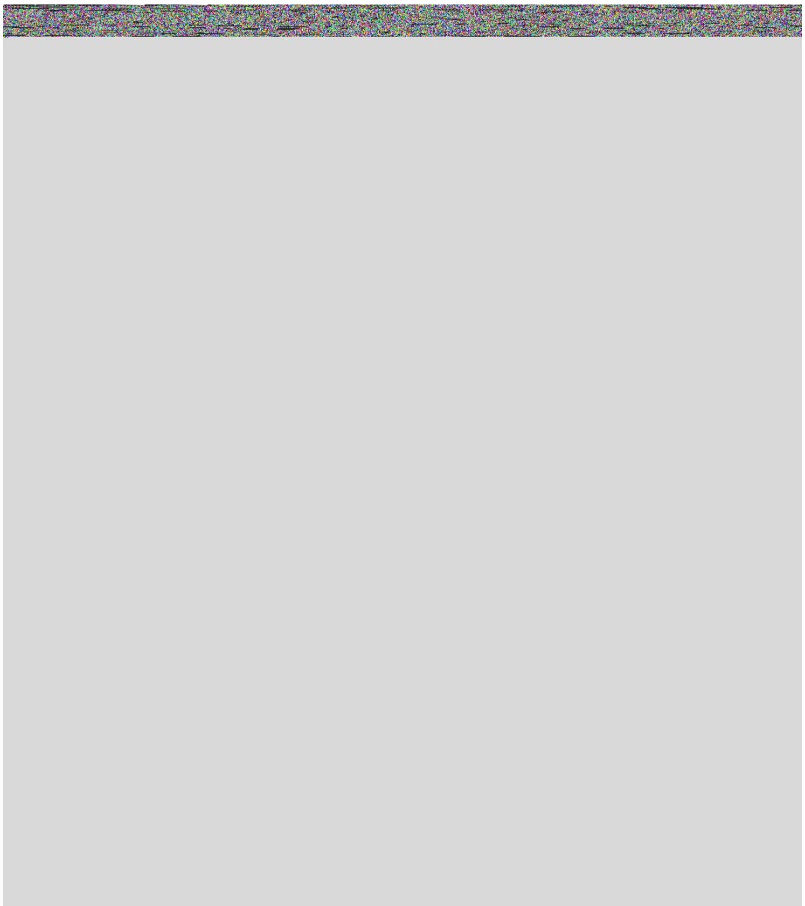

3. Thermal imaging analysis

Thermal imaging was performed with an FLIR TG167 spot thermal camera. Images were collected manually at set time intervals using a stopwatch. The fixed bed reactor of the Vapourtec E-Series flow system was packed with glass beads (0.5 cm diameter) and placed within a Pyrex jacket connected to the flow systems air oven. The jacket was heated to 125 ºC by flowing hot air through the jacket and regulated with a thermocouple. The fixed bed was used as an internal reference to compare the signal intensity of the flowing solution with the defined temperature of the fixed bed reactor. The individual images were collated and analysed by randomly sampling pixels using Image-J software.[12,13] The highest and lowest pixel intensities were calibrated against the defined temperatures of the fixed bed reactor jacket and room temperature (Figure S1).

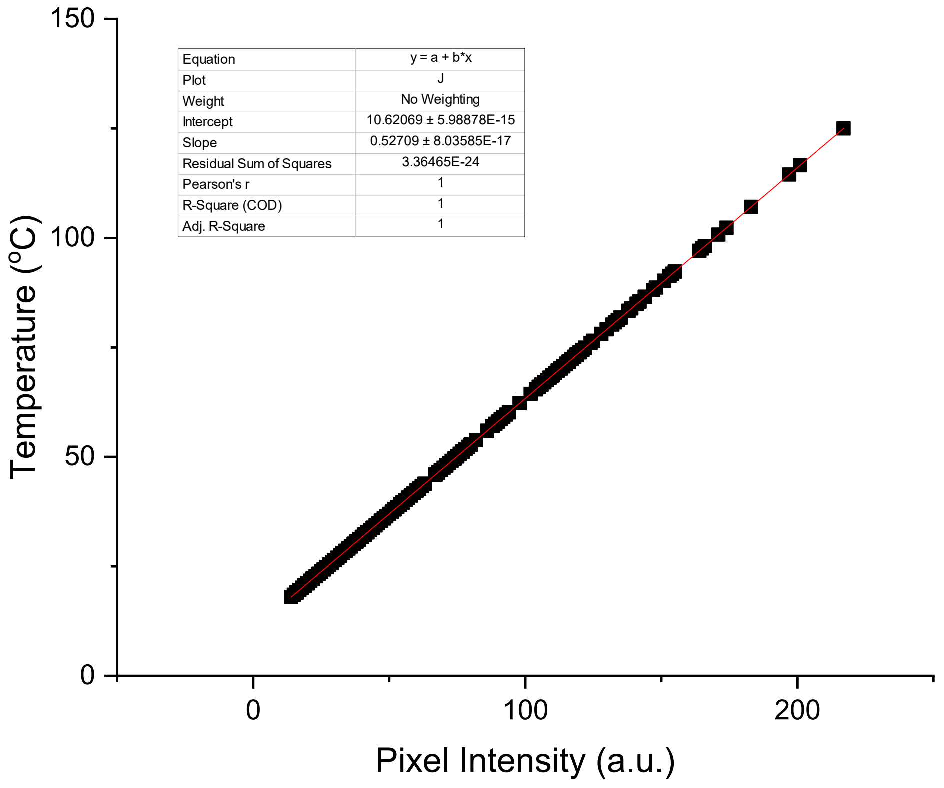

Figure S1. Calibration plot of temperature *vs* pixel intensity.

The conversion factor of pixel intensity to temperature was obtained from the gradient of the linear fit as 0.527 ºC/a.u.. The outline of the length of tubing between the T-junction mixer and the 10 mL reactor coil inlet was selected and used to extract the values of the minimum, mean, and maximum pixel intensity for the selected area (**Figure S2**). Due to the likelihood of accidentally selecting a background pixel, the minimum pixel intensity data was disregarded.

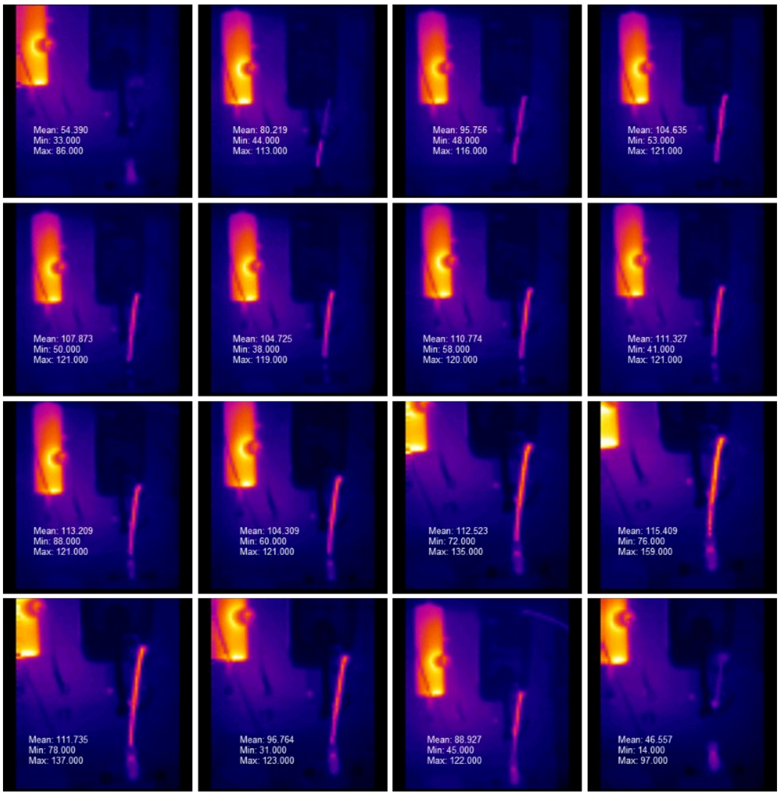

Figure S2. Example of collated thermal camera images used for temperature-intensity calibration and recording the temperature rise of the reactor tubing over time. The recorded mean, minimum and maximum pixel intensity is displayed as text on each image.

4. Residence Time Distribution (RTD) Analysis

RTD analysis was performed as previously reported by Xuan, Vilela and co-workers.[14] Residence time distribution curves were obtained by injecting a tracer solution (1,3,5,7-Tetramethyl-8-phenyl-4,4-difluoroboradiazaindacene (4.62 x10^{-4} M) in toluene) into the reactor inlet stream, using the Vapourtec flow system's solvent-reagent selection valves. The flow reactor set-up used for the measurements is illustrated in Figure S3 and described below.

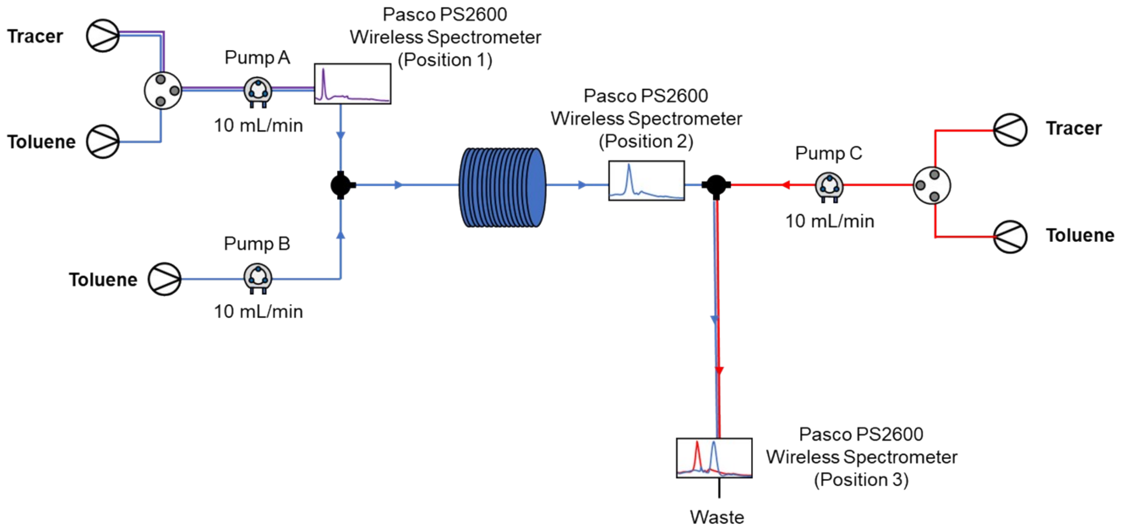

Figure S3. Schematic flow diagram used for RTD analysis.

Whilst pumping blank solvent at 10 mL/min from all three pumps, the valve for either Pump A or Pump C was switched for 1 second to inject 167 µL of tracer solution into the flow stream, before returning the valve to its original position.

A UV-Vis. spectrophotometer (Pasco PS2600 Wireless Spectrophotometer) was connected to the flow system using a flow-through cuvette (Hellma GmbH & Co. KG; 176-761-15-40, 5 mm pathlength) at one of the three positions illustrated in Figure S3. The concentration of tracer exiting the reactor over time was measured by monitoring the absorption intensity of the tracer's maximum absorption intensity wavelength (506 nm) at a sample rate of 5 Hz and 1 nm bandwidth.

The RTD curve, also known as E(t) curve, can be defined as:[15]

$$E(t) = \frac{C(t)}{\int_0^\infty C(t)\,dt} \qquad (1)$$

Where C(t) is the concentration of tracer detected by the spectrometer at time t. The concentration of tracer is assumed to be directly proportional to the measured absorption intensity. The integral of the denominator is obtained from the area of the C(t) curve. The RTD curve must also satisfy the material balance check:

$$\int_0^\infty E(t)\,dt = 1 \qquad (2)$$

The mean residence time ($\bar{t}$) and the variance (σ[2]) of the residence time distribution profile are two important characteristic parameters of the E(t) curve, where $\bar{t}$ is the first moment of the RTD curve and is given by:

$$\bar{t} = \frac{\int_0^\infty tC(t)\,dt}{\int_0^\infty C(t)\,dt} \qquad (3)$$

The second moment - σ^2, is the measure of the spread of the E(t) curve and is useful for matching experimental curves to a family of the theoretical curves:[15]

$$\sigma^2 = \frac{\int_0^\infty (t-\bar{t})^2 C(t)\,dt}{\int_0^\infty C(t)\,dt} \qquad (4)$$

In order to compare the segments of the flow system independently of the mean residence times, the E(t) curve can be normalized by using experimentally obtained $\bar{t}$ values:

$$\theta = \frac{t}{\bar{t}} \qquad (5)$$

$$E(\theta) = \bar{t}E(t) \qquad (6)$$

Whilst dimensionless variance (σ_θ^2) can be defined as:[15]

$$\sigma_\theta^2 = \frac{\sigma^2}{\bar{t}^2} \qquad (7)$$

The dimensionless group can be represented by the Péclet module (Pe), which provides a measure of plug-flow character. The theoretical Péclet number of the system was calculated

as described by Sans, Verdugo and co-workers.[16] The analytical solution to E(θ) under open boundary conditions is described by:

$$E(\theta) = \frac{1}{2}\sqrt{\frac{Pe}{\pi\theta}} e^{-\frac{Pe(1-\theta)^2}{4\theta}} \quad (8)$$

The experimental RTD results were adjusted to equation (8) by solving equation (9) for the value of Pe which minimised the average value of the sum of the squares:

$$\varepsilon = \frac{\sum_{0}^{t_{max}} [y(t) - y_{calc}(t)]^2}{n} \quad (9)$$

Where n represents the total number of time intervals measured. The key values extracted from the RTD analysis for the properties described above are described in **Table S1**. The E(t) and E(θ) curves are displayed separately in Figures S4-8.

Table S1. Summary of key values extracted from RTD analysis.

**Entry**	**Flow Path**	**Net-Flow Rate @ Spectrometer (mL/min)**	$\bar{t}$ /s	**σ²** /s²	σ_θ^2	**Pe**
**1**	Pump A Tracer → Spectrometer Position 1	10	8.57	2.70	0.037	66.46
**2**	Pump A Tracer → Spectrometer Position 2	20	32.19	1.20	0.001	3170.11
**3**	Pump A Tracer → Spectrometer Position 3	30	35.91	3.38	0.003	769.72
**4**	Pump C Tracer → Spectrometer Position 3	30	9.59	1.32	0.014	140.99

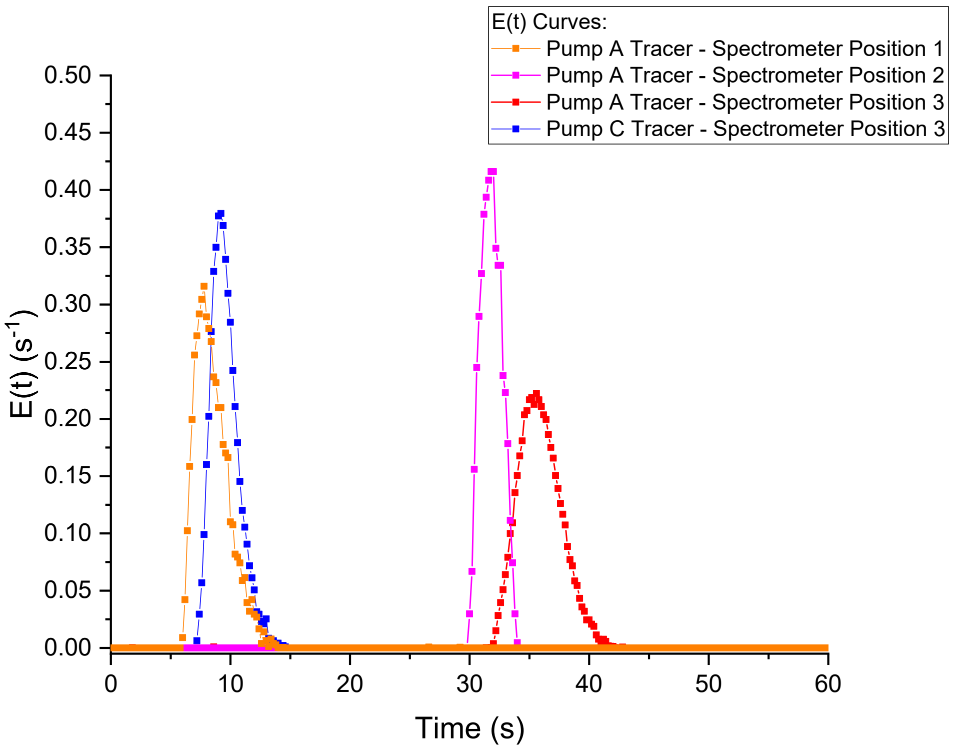

Figure S4. Stacked plot of E(t) *vs* time for each of the flow paths in Table S1.

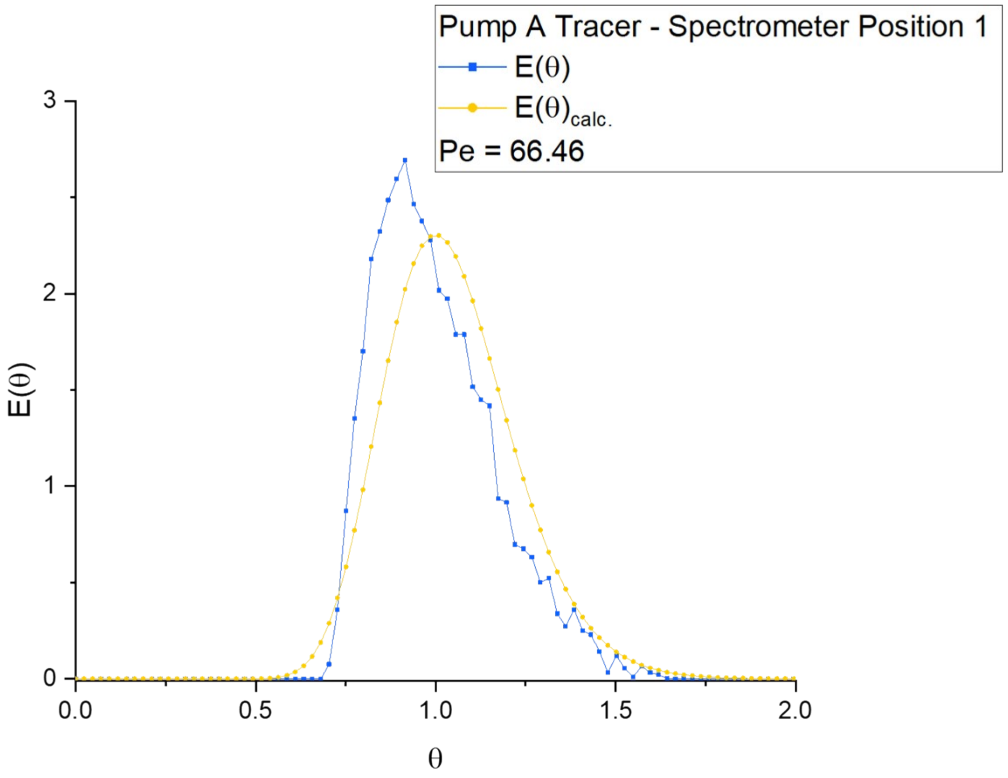

Figure S5. Stacked plot of E(θ) and $E(\theta)_{calc.}$ *vs* time for Entry 1, Table S1.

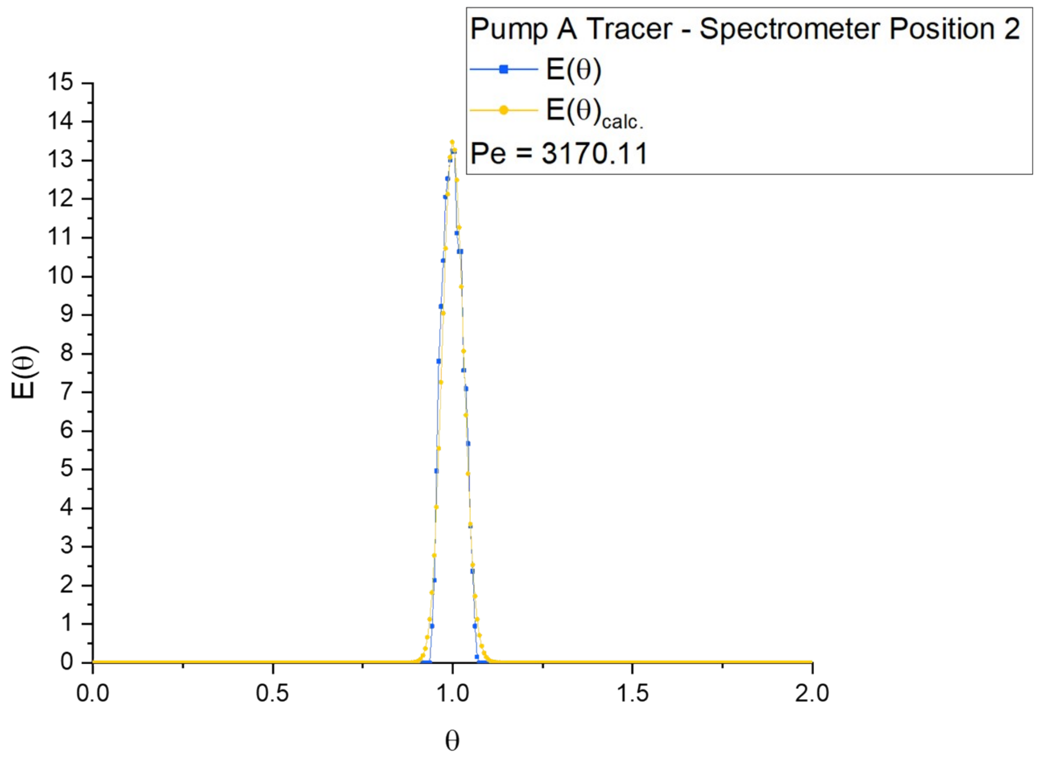

Figure S6. Stacked plot of E(θ) and $E(θ)_{calc.}$ *vs* time for Entry 2, Table S1.

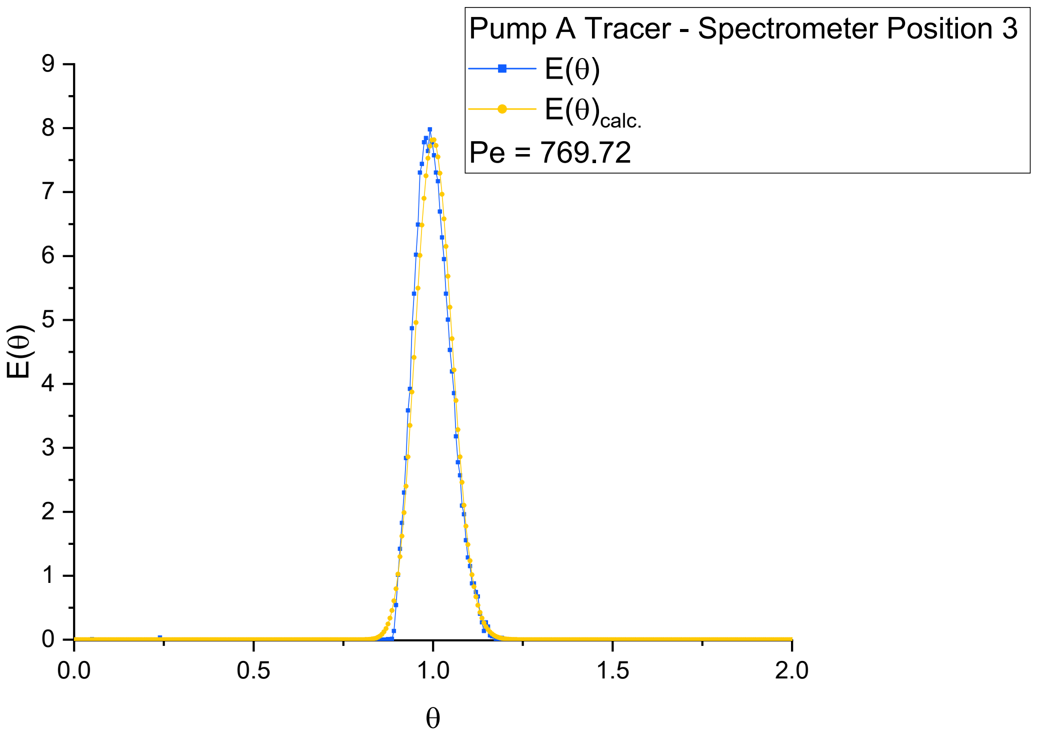

Figure S7. Stacked plot of E(θ) and $E(θ)_{calc.}$ *vs* time for Entry 3, Table S1.

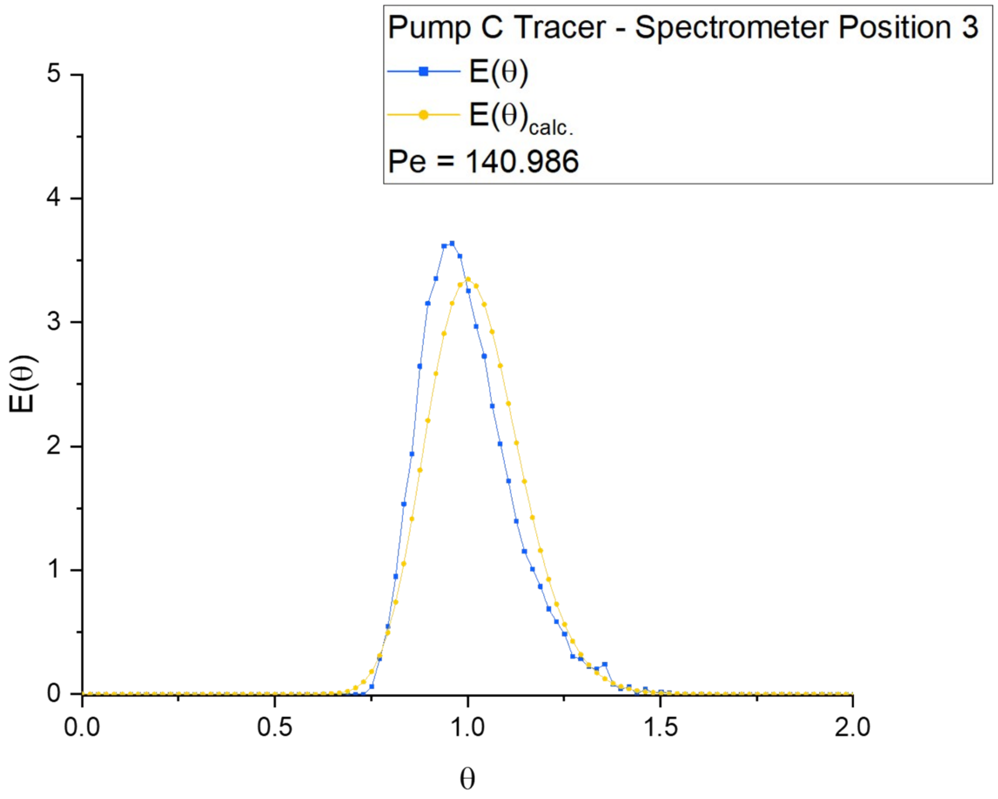

Figure S8. Stacked plot of E(θ) and $E(\theta)_{calc.}$ *vs* time for Entry 4, Table S1.

5. Hoffman Test

Methyl 2-(dibenzylamino)-3-phenylpropanoate *rac*-HT1

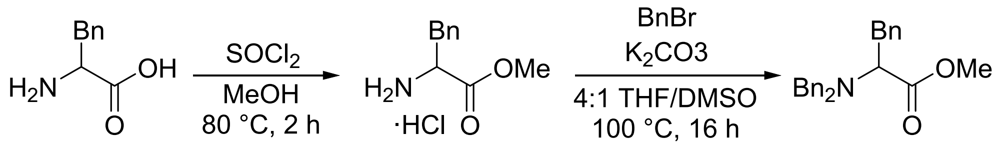

rac-**HT1**

Thionyl chloride (0.91 mL, 1.49 g, 12.5 mmol, 2.5 eq) was added dropwise to a stirring solution of DL-phenylalanine (826 mg, 5 mmol, 1.0 eq) in MeOH (10 mL) at 0 °C, the resulting solution was allowed to warm to rt and stirred and heated at 80 °C for 2 hours. After cooling to rt, the solvent was evaporated under reduced pressure to give DL-phenylalanine methyl ester hydrochloride (1.08 g, 100%) as a white solid which was used in the next step without further purifications, ^{1}H NMR (300 MHz, MeOD-d_4) δ 7.48–7.12 (m, 5H, Ph), 4.32 (dd, J = 7.5, 6.0 Hz, 1H, NCH), 3.81 (s, 3H, OMe), 3.27 (dd, *J* = 14.5, 6.0 Hz, 1H, $PhCH_AH_B$), 3.15 (dd, *J* = 14.5, 7.5 Hz, 1H, $PhCH_AH_B$).

Benzyl bromide (1.25 mL, 1.80 g, 10.5 mmol, 2.1 eq) was added to a stirring supsension of give DL-phenylalanine methyl ester hydrochloride (1.08 g, max) and K_2CO_3 (3.88 g, 17.5 mmol, 3.5 eq) in THF (7 mL) and DMSO (2 mL) at rt, the resulting solution was stirred and heated at 100 °C for 16 h. After cooling to rt, water (20 mL) and EtOAc (20 mL) was added and the two layers were separated, extracting the aqueous layer with EtOAc (2 × 20 mL). The combined organic layers were washed with brine, dried (Na_2SO_4) and evaporated under reduced pressure to give the crude product. Purification by flash column chromatography on silica with 70:30 petroleum ether-PhMe gave ester *rac*-**HT1** (947 mg, 53%) as a colourless oil, R_F (70:30 petroleum ether-PhMe) 0.2; IR (ATR) 3062, 3025, 2952, 2844, 1732 (C=O), 1656, 1601, 1493, 1453, 1438, 1376, 1303, 1250, 1213, 1168, 1117, 1070, 1055, 1028, 1001, 969, 958, 909 cm^{-1}; ^1H NMR (400 MHz, $CDCl_3$) δ 7.33–7.11 (m, 13H, Ph), 7.09–7.00 (m, 2H, Ph), 3.98 (d, J = 14.0 Hz, 2H, NCH_AH_B), 3.76 (s, 3H, OMe), 3.71 (dd, J = 8.5, 7.0 Hz, 1H, NCH), 3.58 (d, J = 14.0 Hz, 2H, NCH_AH_B), 3.16 (dd, J = 14.0, 7.0 Hz, 1H, $PhCH_AH_B$), 3.03 (dd, J = 14.0, 8.5 Hz, 1H, $PhCH_AH_B$); ^{13}C NMR (101 MHz, $CDCl_3$) δ 172.8 (C), 139.3 (C), 138.2 (C), 129.4 (CH), 128.7 (CH), 128.2 (2 × CH), 126.9 (CH), 126.3 (CH), 62.4 (CH), 54.5 (CH_2), 51.2 (CH_3), 35.8 (CH_2). Spectroscopic data consistent with those reported in the literature.[17]

(2S)-Methyl 2-(dibenzylamino)-3-phenylpropanoate (*S*)-HT1

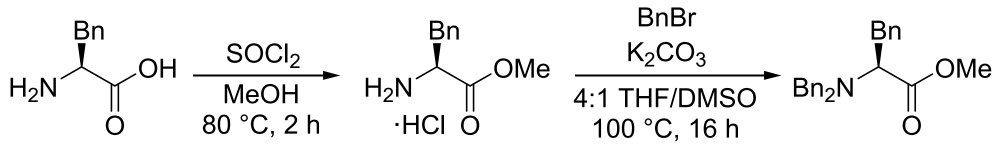

(*S*)-**HT1**

Thionyl chloride (3.64 mL, 5.96 g, 50 mmol, 2.5 eq) was added dropwise to a stirring solution of L-phenylalanine (3.30 g, 20 mmol, 1.0 eq) in MeOH (40 mL) at 0 °C, the resulting solution was allowed to warm to rt and stirred and heated at 80 °C for 2 hours. After cooling to rt, the solvent was evaporated under reduced pressure to give L-phenylalanine methyl ester hydrochloride (4.32 g, 100%) as a white solid which was used in the next step without further purifications.

Benzyl bromide (4.87 mL, 7.01 g, 41 mmol, 2.1 eq) was added to a stirring supsension of give DL-phenylalanine methyl ester hydrochloride (4.32 g, max) and K_2CO_3 (9.67 g, 70 mmol, 3.5 eq) in THF (27 mL) and DMSO (7 mL) at rt, the resulting solution was stirred and heated at 100 °C for 16 h. After cooling to rt, water (60 mL) and EtOAc (60 mL) was added and the two layers

were separated, extracting the aqueous layer with EtOAc (2 × 60 mL). The combined organic layers were washed with brine, dried (Na_2SO_4) and evaporated under reduced pressure to give the crude product. Purification by flash column chromatography on silica with 70:30 petroleum ether-PhMe gave ester *rac*-**HT1** (4.60 g, 64%) as a colourless oil, $[\alpha]_D^{18}$ –88.0 (c 2.2 in $CHCl_3$).

2-(Dibenzylamino)-*N*-methoxy-*N*-methyl-3-phenylpropanamide *rac*-HT2

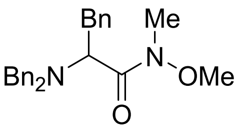

rac-**HT2**

n-BuLi (30 mL of a 2.5 M solution in hexanes, 75 mmol, 3.9 eq) was added dropwise to a stirring suspension of *N*,*O*-dimethylhydroxylamine hydrochloride (3.71 g, 38 mmol, 2.0 eq) in THF (50 mL) at –78 °C, the resulting solution was allowed to warm to rt and stirred at rt for 30 minutes. The solution was then cool to –78 °C and a solution of ester *rac*-**HT1** (6.83 g, 19 mmol, 1.0 eq) in THF (50 mL) was added. The resulting solution was stirred at –78 °C for 1 hour. After warming to rt, saturated $NH_4Cl_{(aq)}$ (50 mL) was added and the two layers were separated, extracting the aqueous layer with Et_2O (2 × 50 mL). The combined organic layers were dried ($MgSO_4$) and evaporated under reduced pressure to give the crude product. Purification by flash column chromatography on silica with 90:10 PhMe-Et_2O and then recrystallisation from hot Et_2O gave amide *rac*-**HT2** (4.37 g, 59%)as a white solid, m.p. 57-59 °C; R_F (90:10 PhMe-Et_2O) 0.3; IR (ATR) 3083, 3025, 3002, 2963, 2922, 2808, 1655, 1600, 1492, 1454, 1391, 1372, 1335, 1308, 1246, 1180, 1151, 1114, 1072, 1041, 1028, 989, 968, 953, 913 cm^{-1}; ^1H NMR (400 MHz, $CDCl_3$) δ 7.30–7.17 (m, 13H, Ph), 7.15 – 7.10 (m, 2H, Ph), 4.09 (br m, 1H, NCH), 3.97 (d, J = 14.5 Hz, 2H, NCH_AH_B), 3.77 (d, J = 14.5 Hz, 2H, NCH_AH_B), 3.26–3.17 (m, 1H, $PhCH_AH_B$), 3.13 (br s, 3H, NMe), 3.04 (dd, J = 13.5, 6.5 Hz, 1H, $PhCH_AH_B$), 2.95 (br s, 3H, OMe); ^{13}C NMR (101 MHz, $CDCl_3$) δ 173.5 (C), 140.2 (C), 138.8 (C), 129.6 (CH), 128.8 (CH), 128.3 (CH), 128.1 (CH), 126.8 (CH), 126.3 (CH), 60.8 (CH_2), 58.8 (CH), 54.6 (CH_3), 34.7 (CH_3), 31.8 (CH_2); MS (ESI) *m/z* 411 [$(M + Na)^+$, 5], 389 [$(M + H)^+$, 100], 300 [$(M – CONMeOMe)^+$, 14]; HRMS (ESI) *m/z* $C_{25}H_{28}N_2O_2$ ([M + H]$^+$) calcd for 389.2224, found 389.2241; CSP-HPLC: Chiralpak IA (98:2 hexane-*i*-PrOH, 1.0 $mLmin^{-1}$) (*S*)-**HT2**: 12.2 min, (*R*)-**HT2**: 13.0 min.

(2S)-2-(Dibenzylamino)-*N*-methoxy-*N*-methyl-3-phenylpropanamide (*S*)-HT2

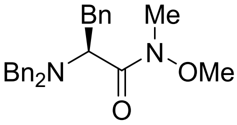

(*S*)-**HT2**

n-BuLi (20.8 mL of a 2.5 M solution in hexanes, 52 mmol, 3.9 eq) was added dropwise to a stirring suspension of *N*,*O*-dimethylhydroxylamine hydrochloride (2.54 g, 26 mmol, 2.0 eq) in THF (25 mL) at –78 °C, the resulting solution was allowed to warm to rt and stirred at rt for 30 minutes. The solution was then cool to –78 °C and a solution of ester (*S*)-**HT1** (4.68 g, 13 mmol, 1.0 eq) in THF (25 mL) was added. The resulting solution was stirred at –78 °C for 1 hour. After warming to rt, saturated $NH_4Cl_{(aq)}$ (25 mL) was added and the two layers were separated, extracting the aqueous layer with Et_2O (2 × 25 mL). The combined organic layers were dried ($MgSO_4$) and evaporated under reduced pressure to give the crude product. Purification by flash column chromatography on silica with 90:10 PhMe-Et_2O and then recrystallisation from hot Et_2O gave amide (*S*)-**HT2** (3.52 g, 70%, >99:1 er by CSP-HPLC) as a white solid, CSP-HPLC: Chiralpak IA (98:2 hexane-*i*-PrOH, 1.0 mLmin^{-1}) (*S*)-**HT2**: 11.5 min, (*R*)-**HT2**: 12.4 min; $[\alpha]_D^{19}$ –144.0 (c 1.0 in $CHCl_3$).

2-(Dibenzylamino)-1,3-diphenylpropan-1-one *rac*-HT3

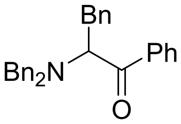

rac-**XX**

PhLi (10 mL of a 2.0 M solution in Bu_2O, 20 mmol, 4.0 eq) was added dropwise to a stirring solution of *rac*-**HT2** (1.94 g, 5 mmol, 1.0 eq) in THF (20 mL) at –78 °C, the resulting solution was stirred at –78 °C for 30 minutes. The resulting solution was allowed to warm to rt. Saturated $NH_4Cl_{(aq)}$ (10 mL) was added and the two layers were separated, extracting the aqueous layer with Et_2O (2 × 10 mL). The combined organic layers were dried ($MgSO_4$) and evaporated under reduced pressure to give the crude product. Purification by hot filtration and recrystallisation from hexane gave ketone *rac*-**HT3** (913 mg, 45%) as a yellow solid, m.p. 85-86 °C; IR (ATR) 3084, 3061, 3022, 3001, 2955, 2923, 2841, 2818, 1686 (C=O str), 1646, 1599, 1582, 1494, 1454, 1447, 1432, 1373, 1342, 1330, 1297, 1231, 1209, 1178, 1155, 1120,

1104, 1001, 991, 965, 956, 945, 926 cm^{-1}; ^{1}H NMR (400 MHz, $CDCl_3$) δ 7.44–7.35 (m, 3H, Ph), 7.27–7.02 (m, 17H, Ph), 4.47 (ddd, *J* = 9.5, 4.5, 2.0 Hz, 1H, NCH), 3.72 (d, *J* = 13.5 Hz, 2H, NCH_AH_B), 3.64 (d, *J* = 13.5 Hz, 2H, NCH_AH_B), 3.29 (ddd, J = 13.5, 9.5, 2.0 Hz, 1H, PhCH_AH_B), 3.05 (ddd, J = 13.5, 4.5, 2.0 Hz, 1H, PhCH_AH_B); ^{13}C NMR (101 MHz, $CDCl_3$) δ 199.7 (C), 139.2 (2 × C), 137.3 (C), 132.7 (C), 129.5 (CH), 129.2 (CH), 128.6 (CH), 128.4 (CH), 128.3 (2 × CH), 128.2 (CH), 127.2 (CH), 126.1 (CH), 62.9 (CH), 54.4 (CH_2), 30.3 (CH_2); MS (ESI) *m/z* 428 [$(M + Na)^+$, 14], 406 [$(M + H)^+$, 100]; HRMS (ESI) *m/z* $C_{29}H_{27}NO$ ([M + H]$^+$) calcd for 406.2165, found 406.2172; CSP-HPLC: Chiralpak IA (98:2 hexane-*i*-PrOH, 1.0 mLmin^{-1}) (*R*)-**HT3**: 7.47 min, (*S*)-**HT3**: 8.02 min.

(2S)-2-(Dibenzylamino)-1,3-diphenylpropan-1-one (*S*)-HT3

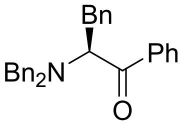

(*S*)-**HT3**

PhLi (16 mL of a 2.0 M solution in Bu_2O, 32 mmol, 4.0 eq) was added dropwise to a stirring solution of (*S*)-**HT2** (3.11 g, 5 mmol, 1.0 eq) in THF (25 mL) at –78 °C, the resulting solution was stirred at –78 °C for 30 minutes. The resulting solution was allowed to warm to rt. Saturated $NH_4Cl_{(aq)}$ (15 mL) was added and the two layers were separated, extracting the aqueous layer with Et_2O (2 × 15 mL). The combined organic layers were dried ($MgSO_4$) and evaporated under reduced pressure to give the crude product. Purification by hot filtration and recrystallisation from hexane gave ketone (*S*)-**HT3** (1.62 g, 50%, >99:1 er by CSP-HPLC) as a yellow solid, CSP-HPLC: Chiralpak IA (98:2 hexane-*i*-PrOH, 1.0 mLmin^{-1}) (*R*)-**HT3**: 7.15 min, (*S*)-**HT3**: 8.46 min, $[\alpha]_D^{19}$ –64.0 (c 1.0 in $CHCl_3$).

Determination of the configurational stability of lithiated 5 at –78 °C

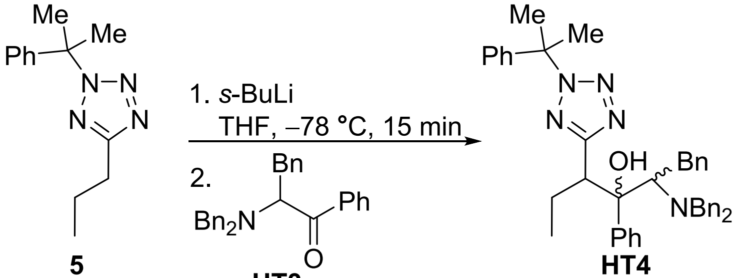

5 **HT3** **HT4**

rac-**HT3**: 65:35 dr
S-**HT3**: 60:40 dr

Using general procedure C, *s*-BuLi (0.6 mL of a 1.3 M solution in cyclohexane/hexane, 1.6 mmol, 1.6 eq) and tetrazole **5** (115 mg, 0.5 mmol, 1.0 eq) in THF (4 mL) at –78 °C for 15 minutes then a solution of *rac*-**HT3** (406 mg, 1 mmol, 2.0 eq) in THF (2 mL) gave the crude product as 65:35 diastereomeric mixture of **HT4**. A pure sample of one of the diastereomer of **HT4** was isolated by column chromatography on silica with 90:10 PhMe-petroleum ether→100 PhMe→90:10 PhMe-Et_2O as eluent, ^1H NMR (400 MHz, $CDCl_3$) δ 7.51–7.44 (m, 4H, Ph), 7.38 (t, J = 7.5 Hz, 4H, Ph), 7.34–7.10 (m, 13H, Ph), 7.05–6.89 (m, 3H, Ph), 6.59–6.35 (m, 2H, Ph), 4.26 (dd, *J* = 11.5, 4.0 Hz, 1H, TzCH), 4.05 (s, 1H, OH), 3.87 (dd, *J* = 8.0, 3.5 Hz, 1H, NCH), 3.49 (dd, *J* = 14.5, 3.5 Hz, 1H, NCHC$H_A$$H_B$), 3.26 (br m, 4H, NCH_2), 2.75 (dd, *J* = 14.5, 8.0 Hz, 1H, NCHC$H_A$$H_B$), 1.94 (s, 3H, C$Me_A$$Me_B$), 1.91 (s, 3H, C$Me_A$$Me_B$), 1.50 (ddq, J = 14.5, 11.5, 7.5 Hz, 1H, MeC$H_A$$H_B$), 0.72 (m, 1H, MeC$H_A$$H_B$), 0.26 (t, J = 7.5 Hz, 3H, *Me*CH_2); ^{13}C NMR (101 MHz, $CDCl_3$) δ 168.4 (C), 144.2 (C), 143.8 (C), 143.6 (C), 129.7 (CH), 128.6 (2 × CH), 128.4 (CH), 128.3 (2 × CH), 127.8 (CH), 127.4 (CH), 127.1 (CH), 126.5 (CH), 125.7 (CH), 124.2 (CH), 79.9 (C), 68.2 (C), 63.8 (CH), 44.2 (CH), 30.7 (2 × CH_2), 29.3 (CH_3), 29.2 (CH_3), 20.8 (2 × CH_2), 12.3 (CH_3); MS (ESI) *m/z* 658 [$(M + Na)^+$, 7], 636 [$(M + H)^+$, 100], 464 [$(M + H_3 – PhMe_2CN_4)^+$, 41]; HRMS (ESI) *m/z* $C_{21}H_{26}N_4O$ ($[M + H]^+$) calcd for 636.3697, found 636.3715.

Using general procedure C, *s*-BuLi (0.6 mL of a 1.3 M solution in cyclohexane/hexane, 1.6 mmol, 1.6 eq) and tetrazole **5** (115 mg, 0.5 mmol, 1.0 eq) in THF (4 mL) at –78 °C for 15 minutes then a solution of (*S*)-**HT3** (406 mg, 1 mmol, 2.0 eq) in THF (2 mL) gave the crude product as 60:40 diastereomeric mixture of **HT4**.

With *rac*-**HT3**

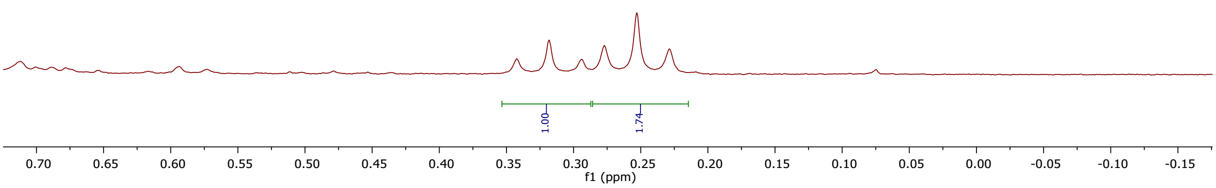

With (*S*)-

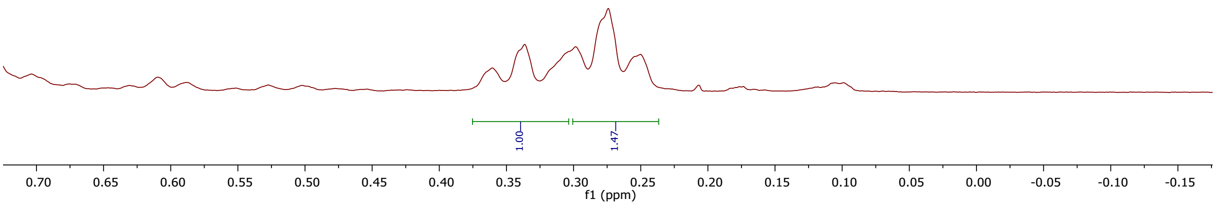

6. References

1. J. Y. F. Wong, J. M. Tobin, F. Vilela and G. Barker, *Chem. Eur. J.*, 2019, **25**, 12439-12445.
2. J. Y. F. Wong, A. Lewandowska, B. R. Trowse and G. Barker, *Org Lett*, 2019, **21**, 7069-7072.
3. R. J. Mudd, P. C. Young, J. A. Jordan-Hore, G. M. Rosair and A.-L. Lee, *J. Org. Chem.*, 2012, **77**, 7633-7639.
4. R. Houssin, J.-L. Bernier and J.-P. Hénichart, *Synthesis*, 1988, **1988**, 259-261.
5. D. Stead, P. O'Brien and A. Sanderson, *Org. Lett.*, 2008, **7**, 1409-1412.
6. I. Coldham, S. Raimbault, P. T. Chovatia, J. J. Patel, D. Leonori, N. S. Sheikh and D. T. E. Whittaker, *Chem. Commun.*, 2008, 4174-4176.
7. J. Davis, R. Moore, B. Imperiali, A. Pratt, K. Kobayashi, S. Masamune, A. Sinskey, C. Walsh, T. Fukui and K. Tomita, *J. Biol. Chem.*, 1987, **262**, 82-89.
8. Z. Yong, *CN Pat., to Mianyang Runtu Agricultural Tech C Ltd.,* CN/106866464/A, 2017
9. Y. Nakao, A. Yada and T. Hiyama, *J. Am. Chem. Soc.*, 2010, **132**, 10024-10026.
10. T. Fukuyama, K. Yamada, T. Nishikawa, D. Ravelli, M. Fagnoni and I. Ryu, *Chem. Lett.*, 2018, **47**, 207-209.
11. Y. Garcia, J. C. Hannam, T. Harrison, C. L. Hamblett, J. L. HubbsJanusz, J. Kulagowski, A. Madin, M. P. Ridgill and E. Seward, *Int. Appl., to Merck Sharp & Dohme Inc.*, WO2007/125364 A1, 2007.
12. W. S. Rasband, ImageJ, U. S. National Institutes of Health, Bethesda, Maryland, USA, https://imagej.nih.gov/ij/, 1997-2018.
13. C. A. Schneider, W. S. Rasband and K. W. Eliceiri, *Nat. Methods*, **9**, 2012, 671-675.
14. A. Zhakeyev, M. C. Jones, C. G. Thomson, J. M. Tobin, H. Wang, F. Vilela, J. Xuan, *Add. Manu.*, 2021, **31**, 101828.
15. O. Levenspiel, *Chemical Reaction Engineering*, Wiley, 3rd edn., New York, 1999.
16. V. Sans, N. Karbass, M. I. Burguete, E. García-Verdugo, S. V. Luis, *RSC Adv.*, 2012, **2**, 8721-8728.
17. T. Sun, W. Zhang, C. Zong, P. Wang and Y. Li, *J. Pept. Sci.*, 2010, **16**, 364-374.

7. $^{1}H/^{13}C/^{19}F$ NMR Spectra and CPS-HPLC Traces

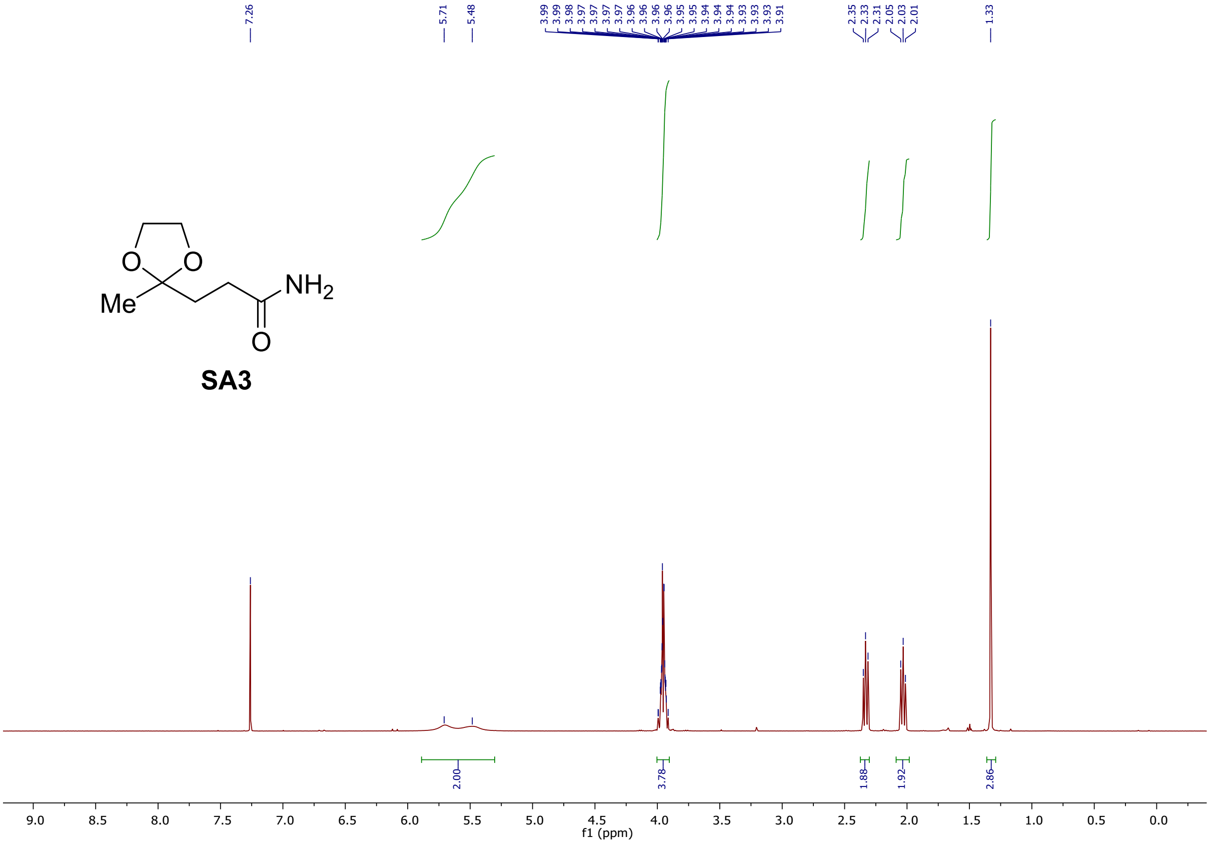

SA3

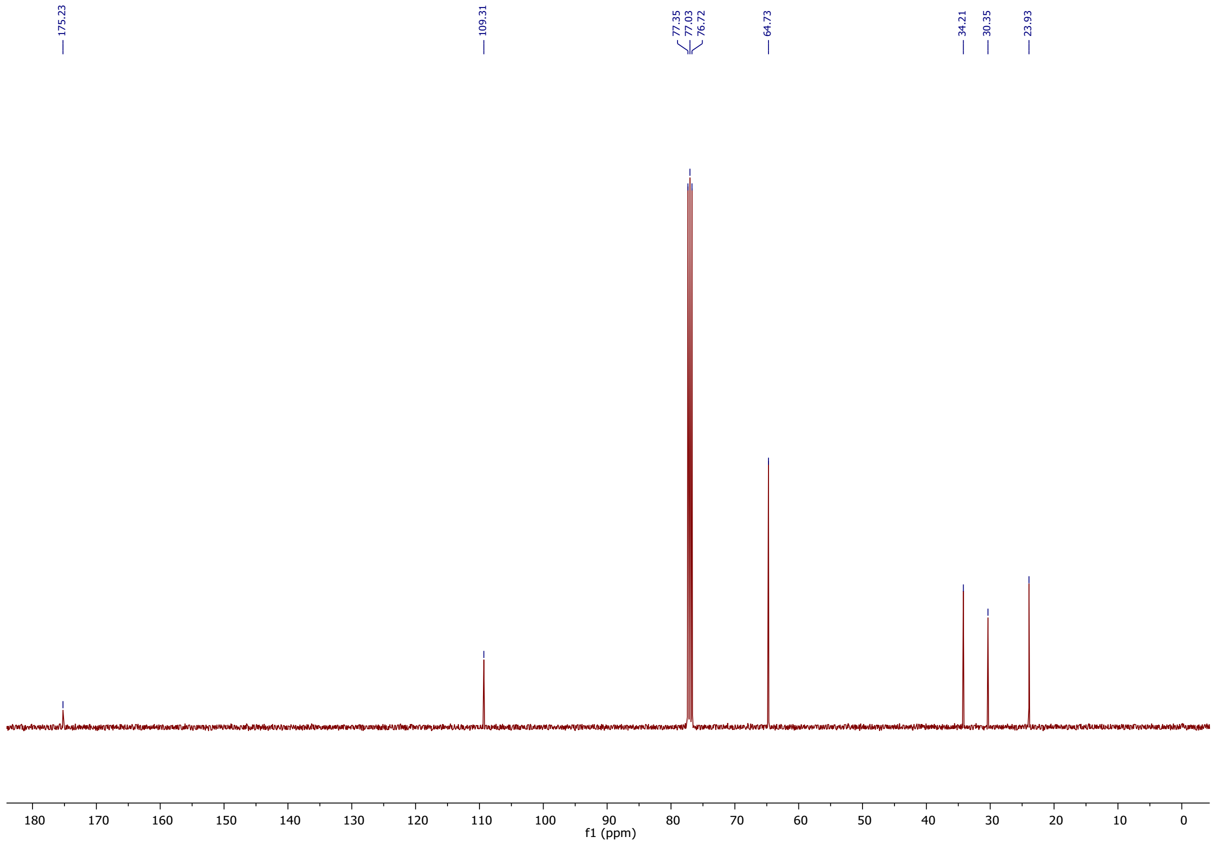
175.23
109.31
77.35
77.03
76.72
64.73
34.21
30.35
23.93
f1 (ppm)

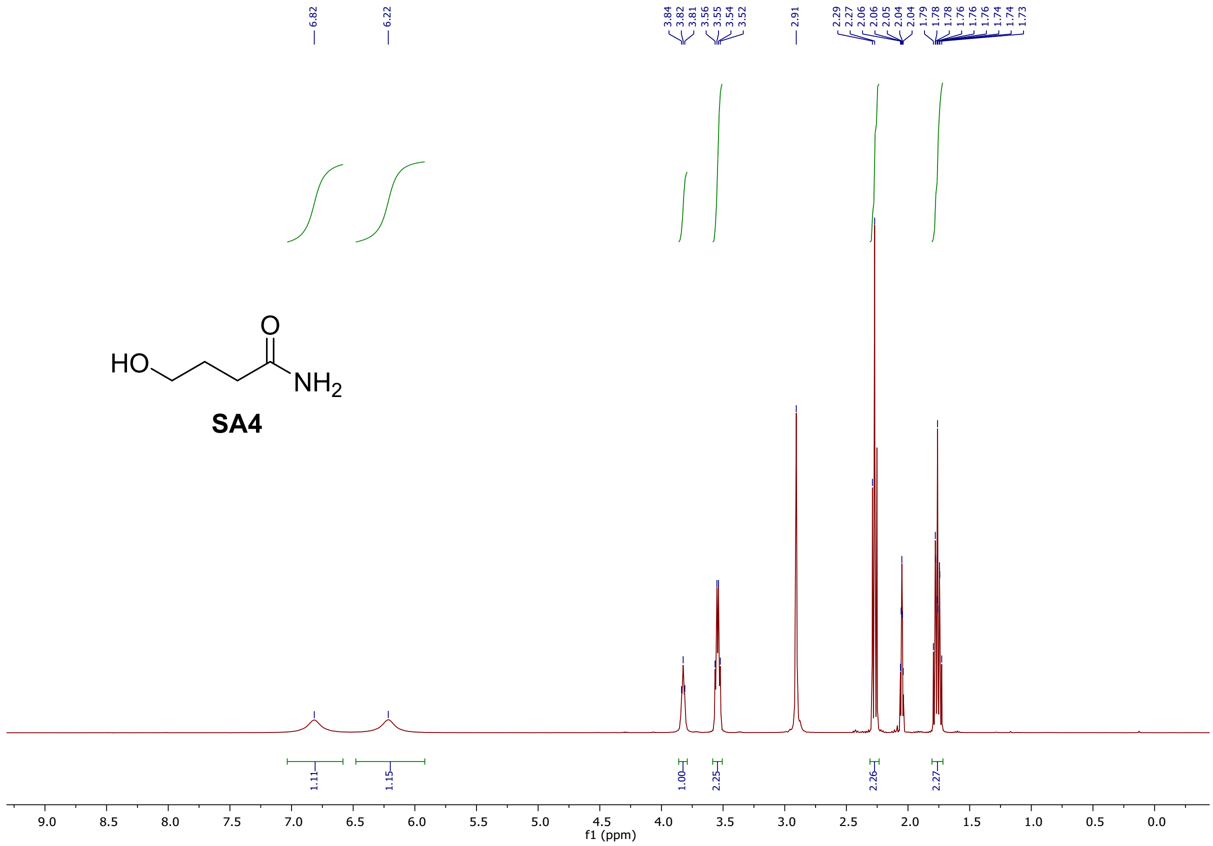
6.82
6.22
3.84
3.82
3.81
3.56
3.55
3.54
3.52
2.91
2.29
2.27
2.06
2.06
2.05
2.04
2.04
1.79
1.78
1.78
1.76
1.76
1.76
1.74
1.74
1.73
HO
O
NH2
SA4
1.11
1.15
1.00
2.25
2.26
2.27
f1 (ppm)

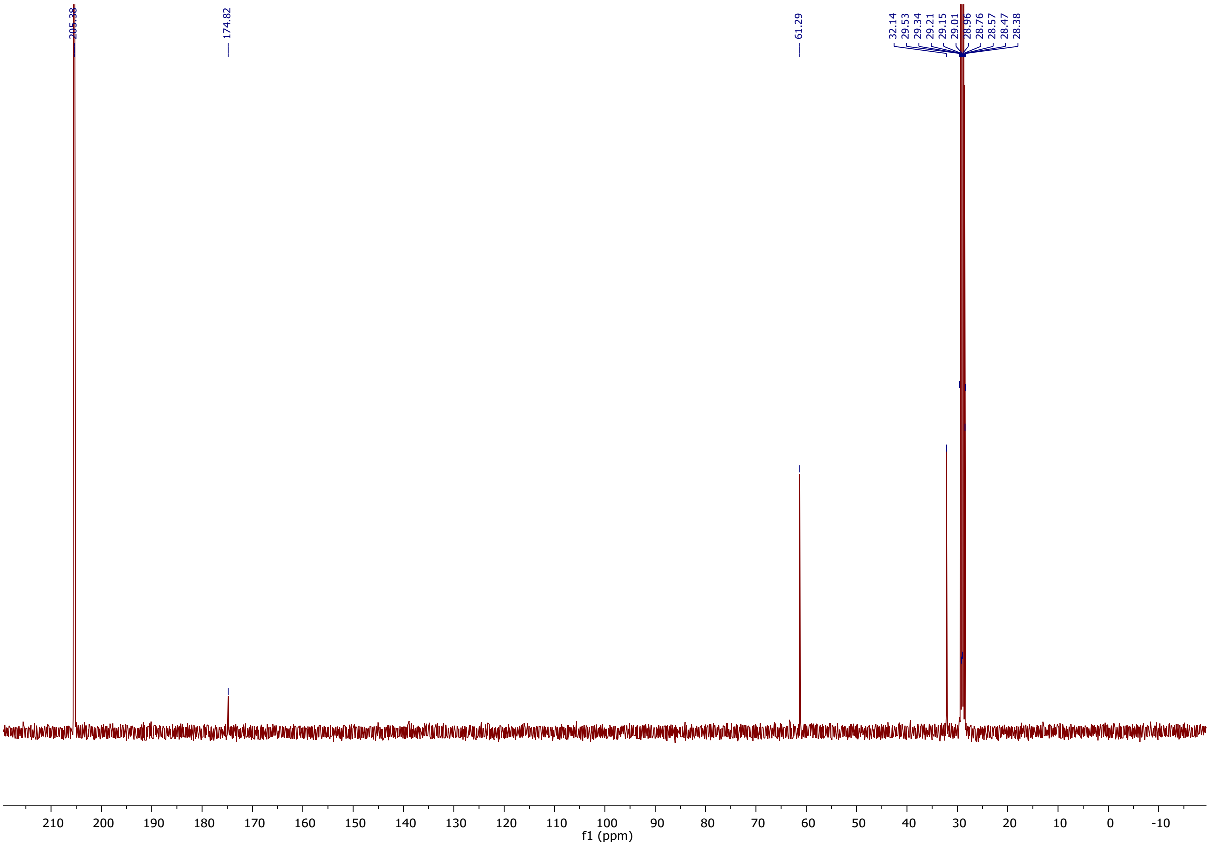
205.38
174.82
61.29
32.14
29.53
29.34
29.21
29.15
29.01
28.96
28.76
28.57
28.47
28.38
f1 (ppm)

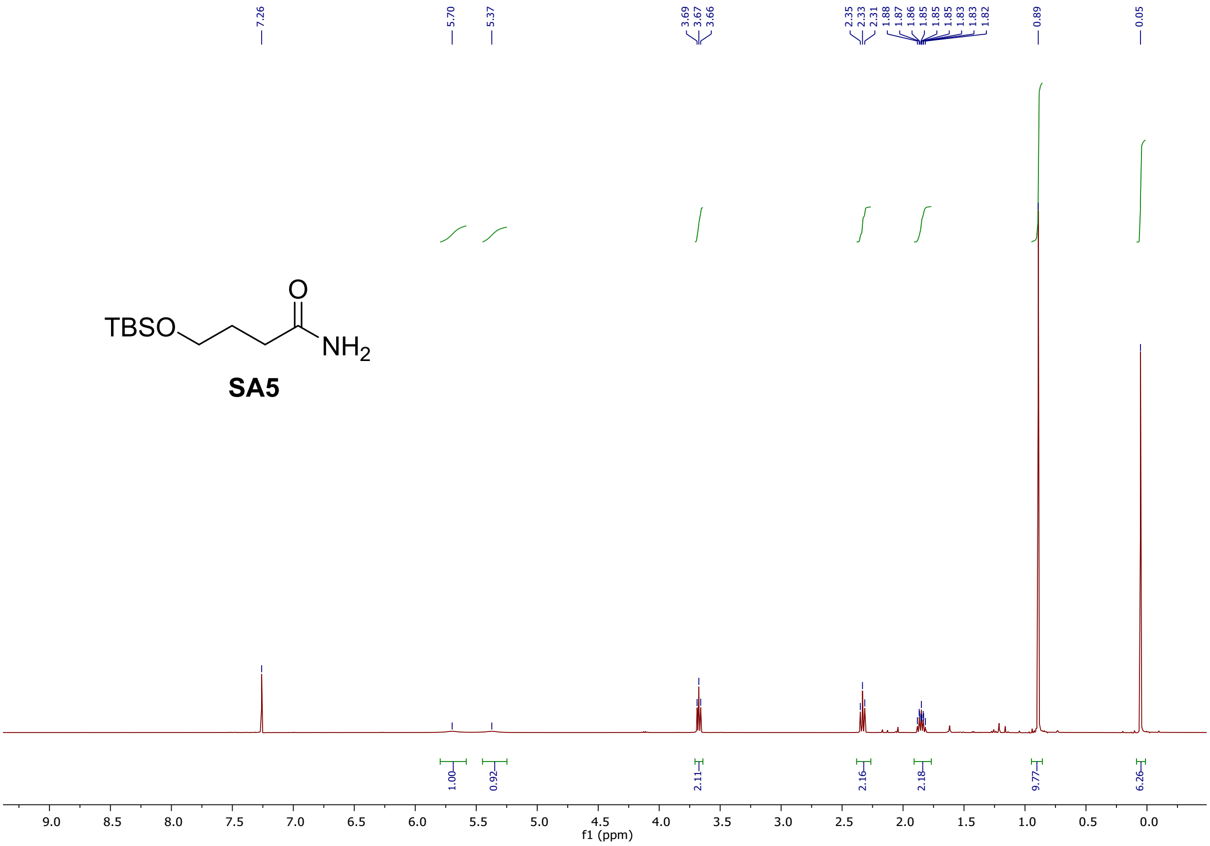
7.26
5.70
5.37
3.69
3.67
3.66
2.35
2.33
2.31
1.88
1.87
1.86
1.85
1.85
1.85
1.83
1.83
1.82
0.89
0.05
TBSO
O
NH2
SA5
1.00
0.92
2.11
2.16
2.18
9.77
6.26
f1 (ppm)

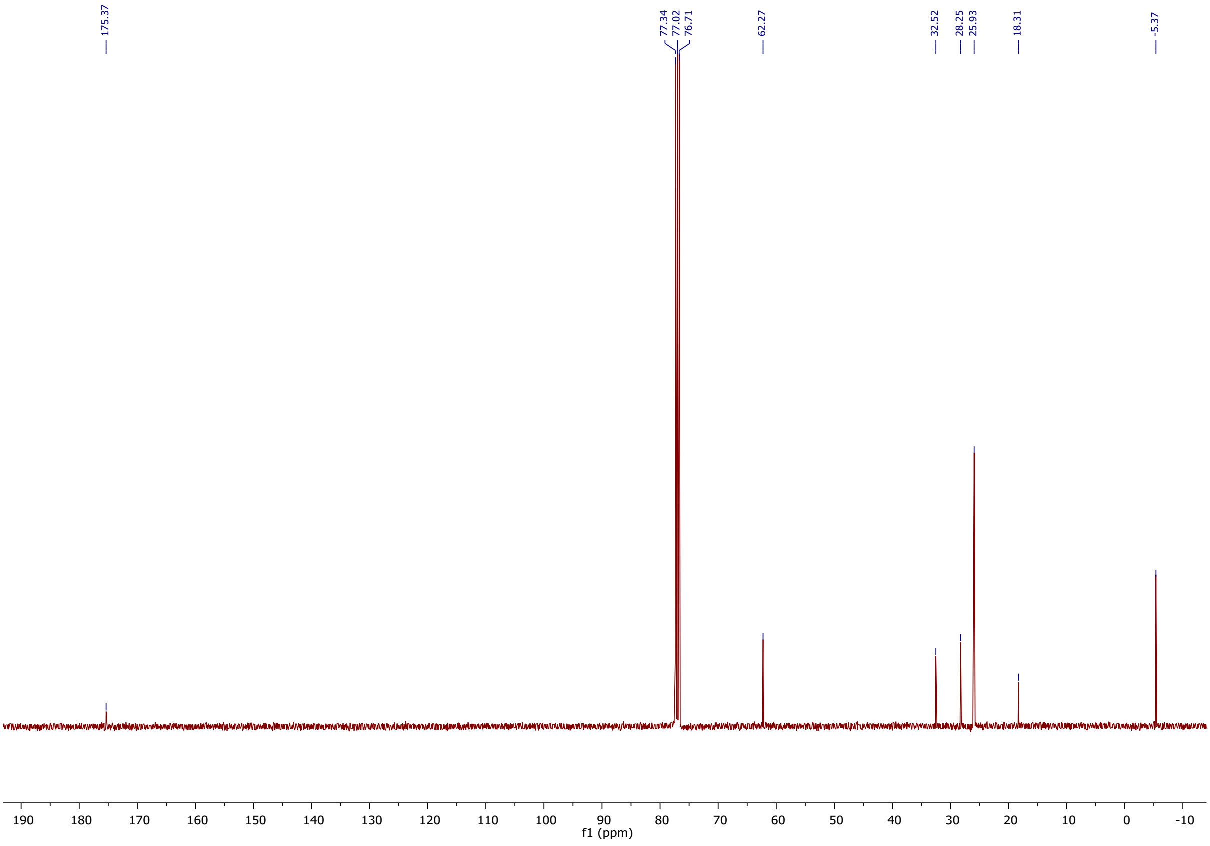
175.37
77.34
77.02
76.71
62.27
32.52
28.25
25.93
18.31
-5.37
f1 (ppm)

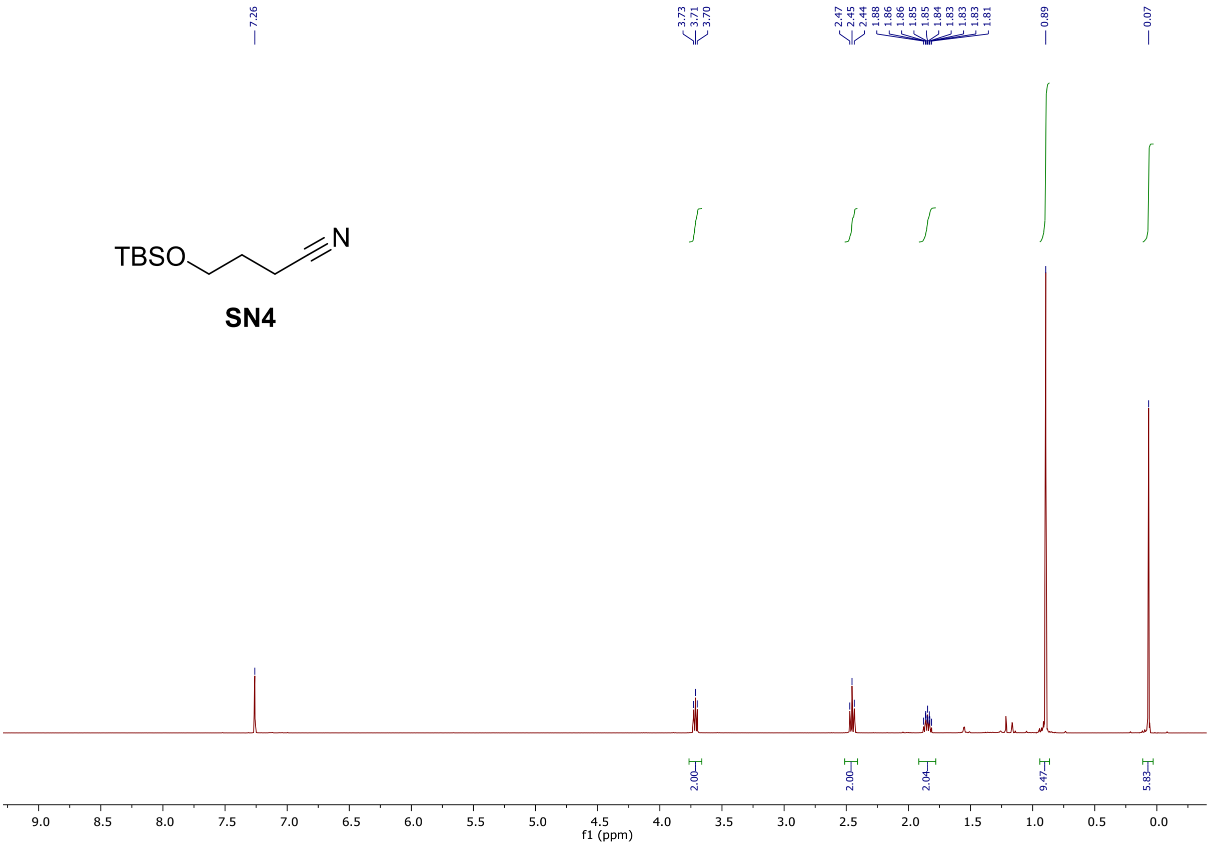
TBSO
N
SN4
7.26
3.73
3.71
3.70
2.47
2.45
2.44
1.88
1.86
1.86
1.85
1.85
1.84
1.83
1.83
1.83
1.81
0.89
0.07
2.00
2.00
2.04
9.47
5.83
f1 (ppm)

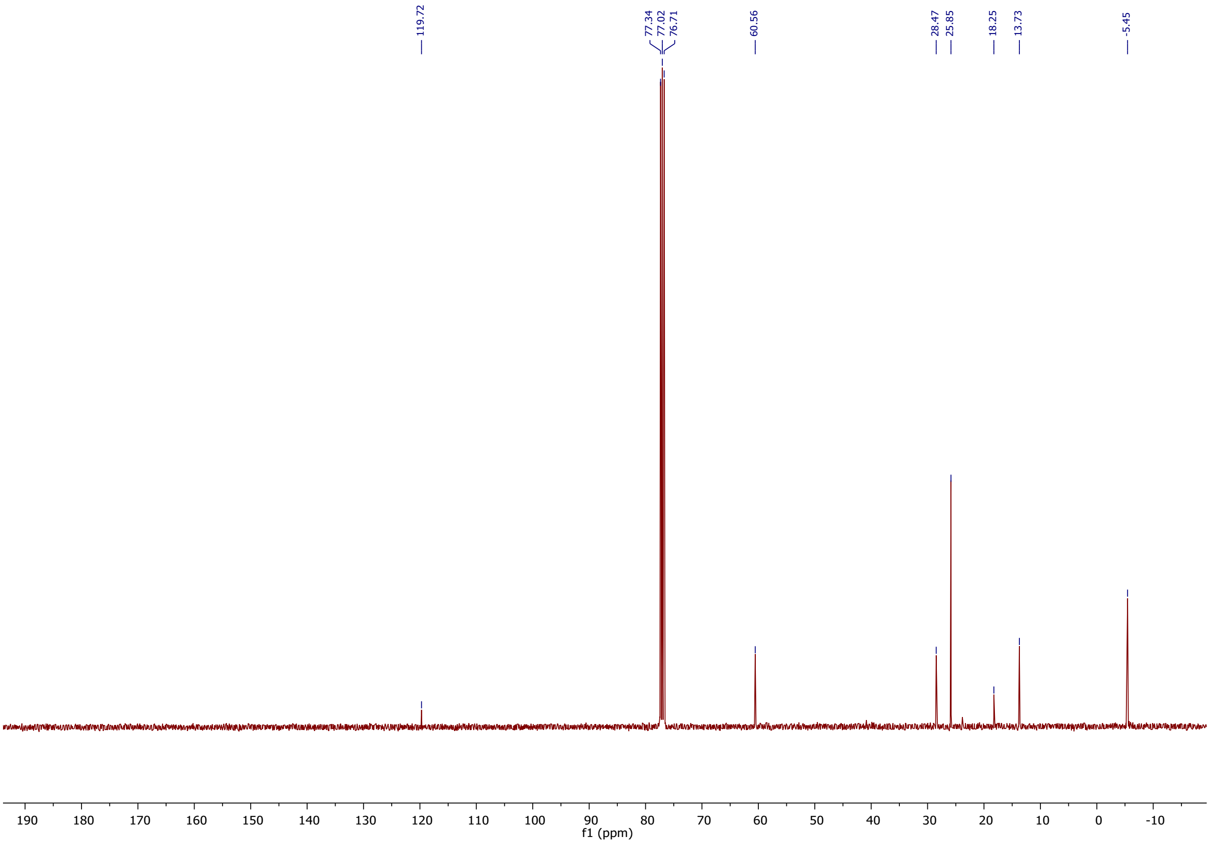
119.72
77.34
77.02
76.71
60.56
28.47
25.85
18.25
13.73
-5.45
190 180 170 160 150 140 130 120 110 100 90 80 70 60 50 40 30 20 10 0 -10
f1 (ppm)

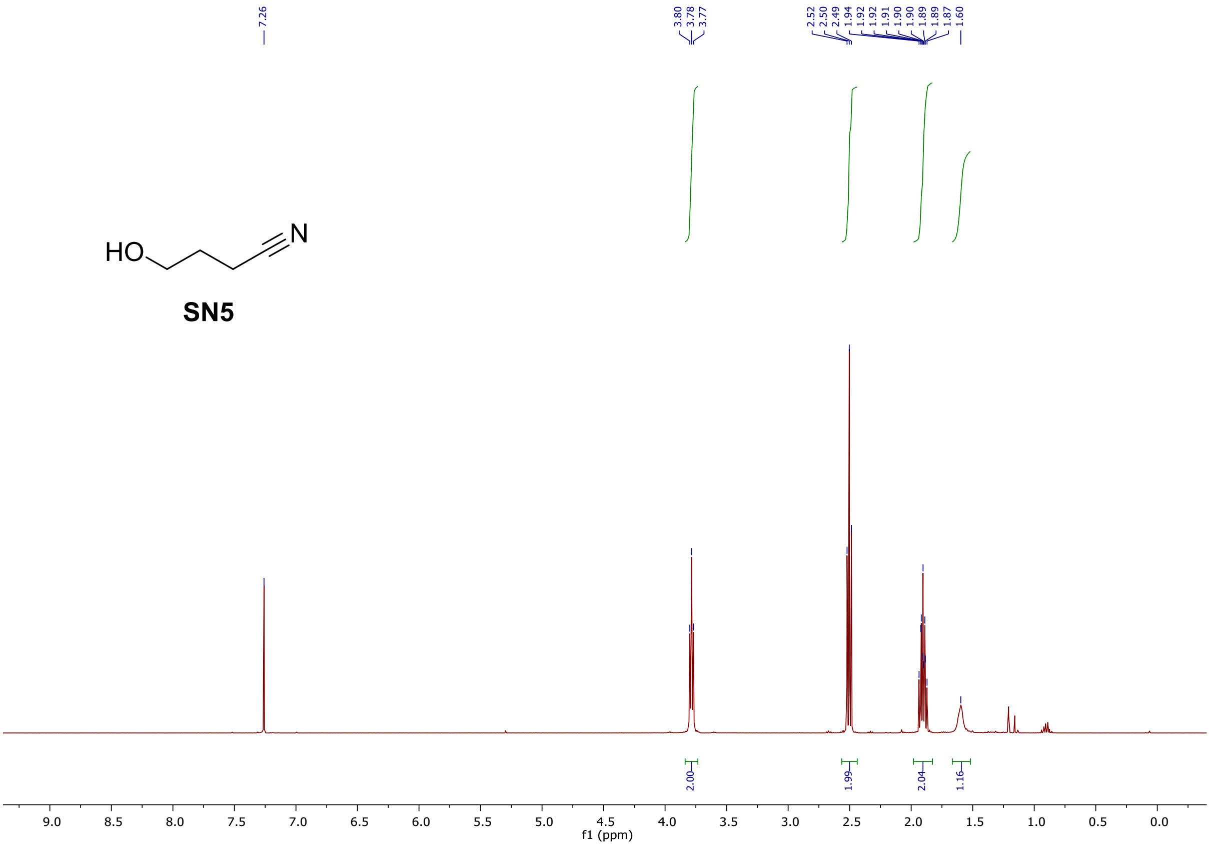
HO
N
7.26
3.80
3.78
3.77
2.52
2.50
2.49
1.94
1.92
1.92
1.91
1.90
1.90
1.89
1.89
1.87
1.60
2.00
1.99
2.04
1.16
9.0 8.5 8.0 7.5 7.0 6.5 6.0 5.5 5.0 4.5 4.0 3.5 3.0 2.5 2.0 1.5 1.0 0.5 0.0
f1 (ppm)

SN5

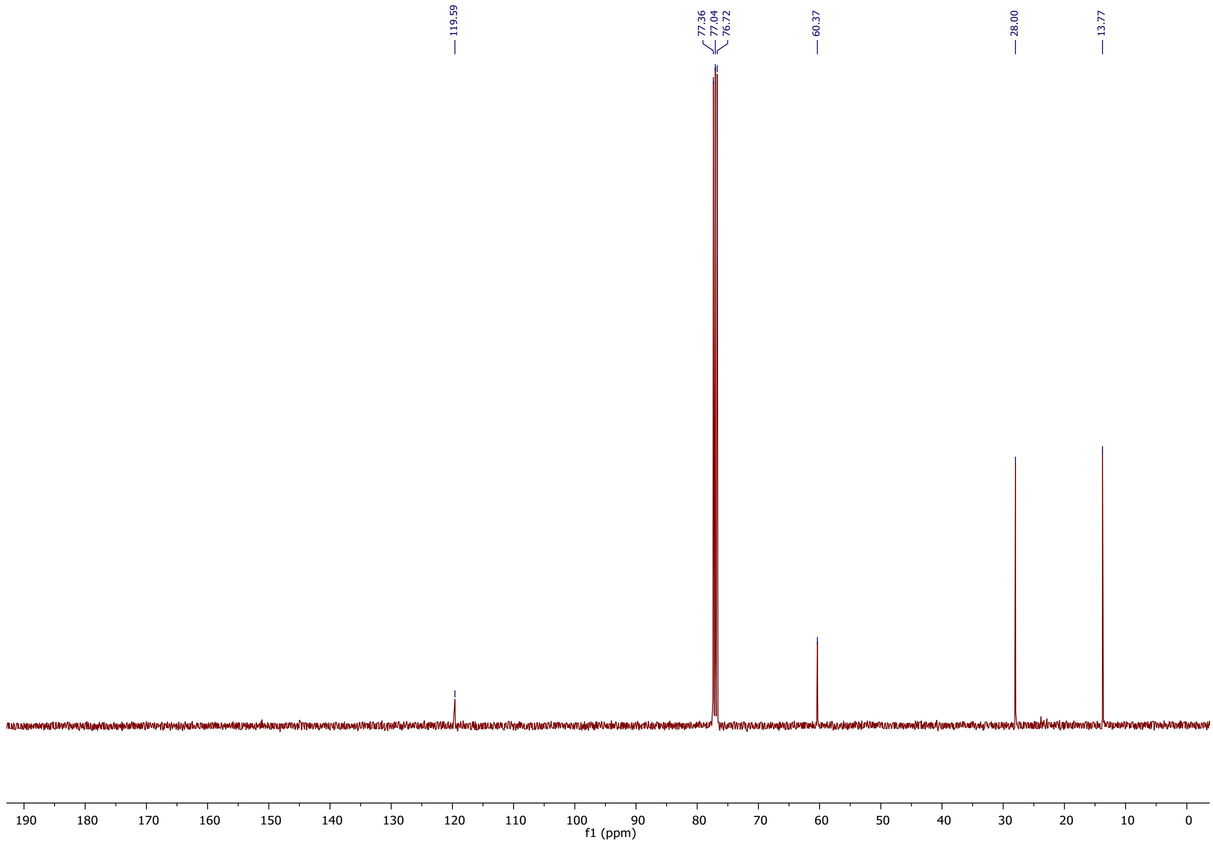
119.59
77.36
77.04
76.72
60.37
28.00
13.77
190 180 170 160 150 140 130 120 110 100 90 80 70 60 50 40 30 20 10 0
f1 (ppm)

SN6

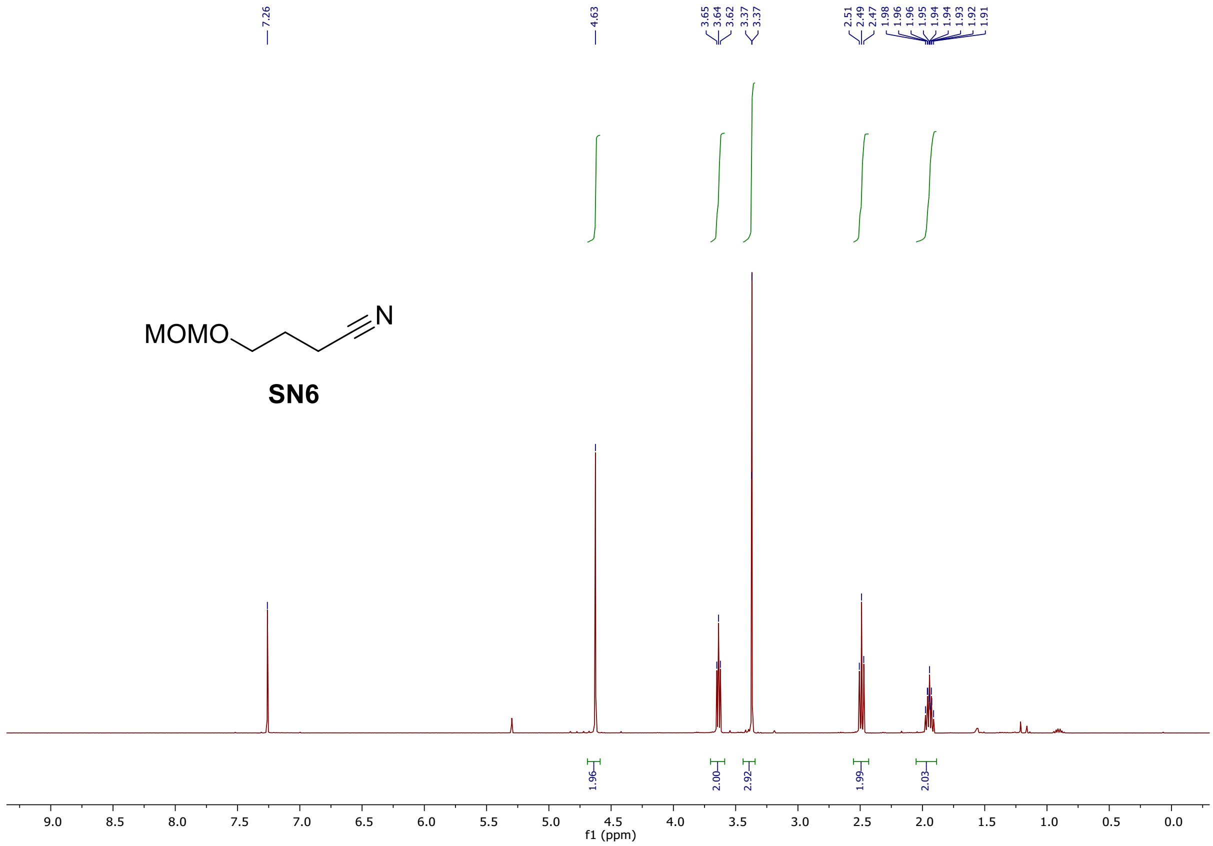
7.26
4.63
3.65
3.64
3.62
3.37
3.37
2.51
2.49
2.47
1.98
1.96
1.96
1.95
1.94
1.94
1.93
1.92
1.91
1.96
2.00
2.92
1.99
2.03
9.0 8.5 8.0 7.5 7.0 6.5 6.0 5.5 5.0 4.5 4.0 3.5 3.0 2.5 2.0 1.5 1.0 0.5 0.0
f1 (ppm)

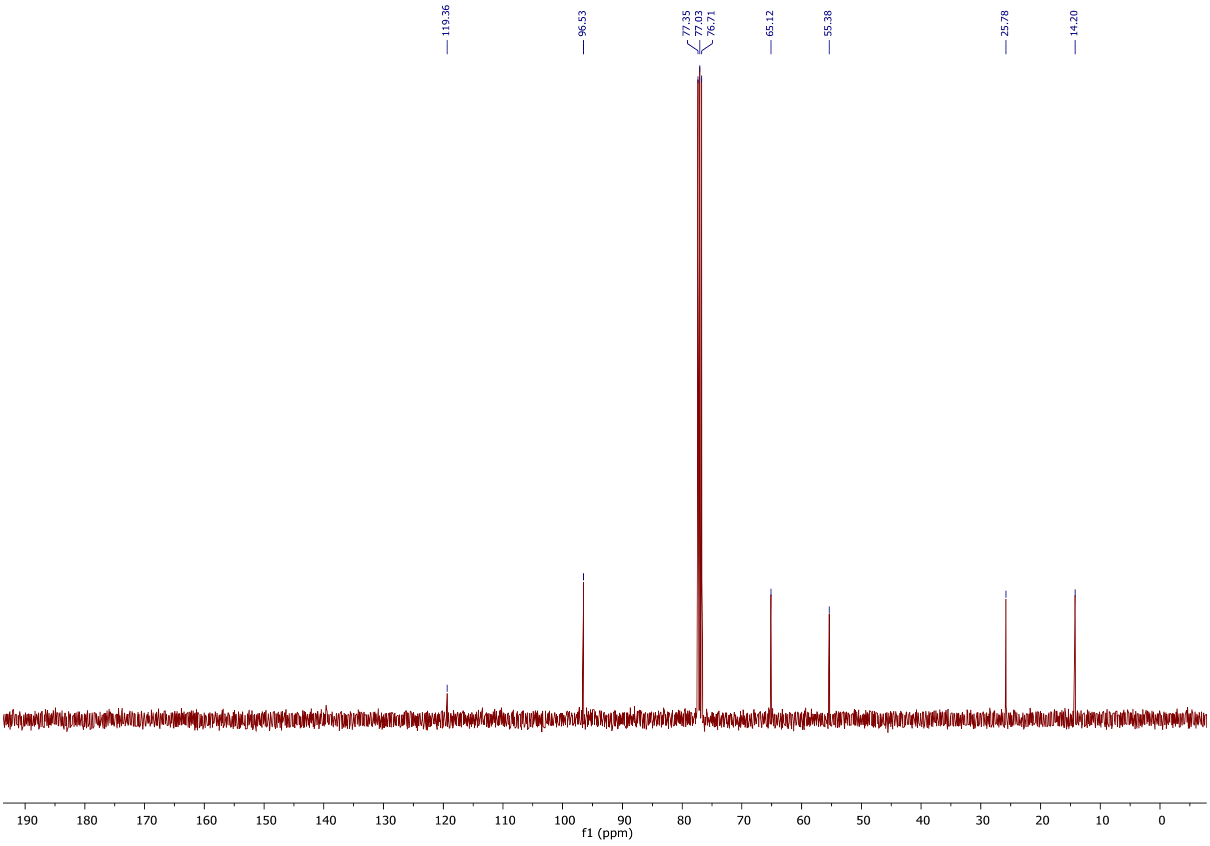
119.36
96.53
77.35
77.03
76.71
65.12
55.38
25.78
14.20
f1 (ppm)

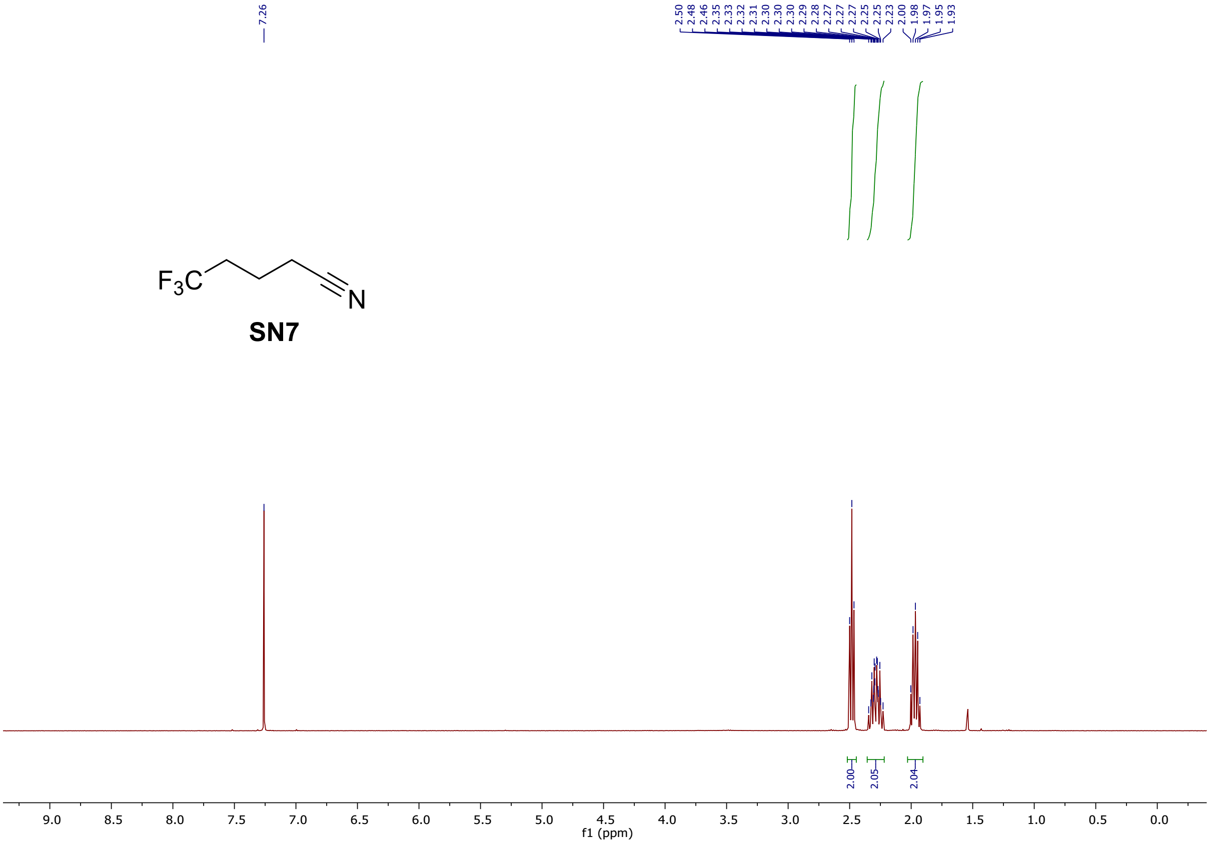
F3C
N
SN7
7.26
2.50
2.48
2.46
2.35
2.33
2.32
2.31
2.30
2.30
2.29
2.28
2.27
2.27
2.27
2.25
2.25
2.23
2.00
1.98
1.97
1.95
1.93
2.00
2.05
2.04
f1 (ppm)

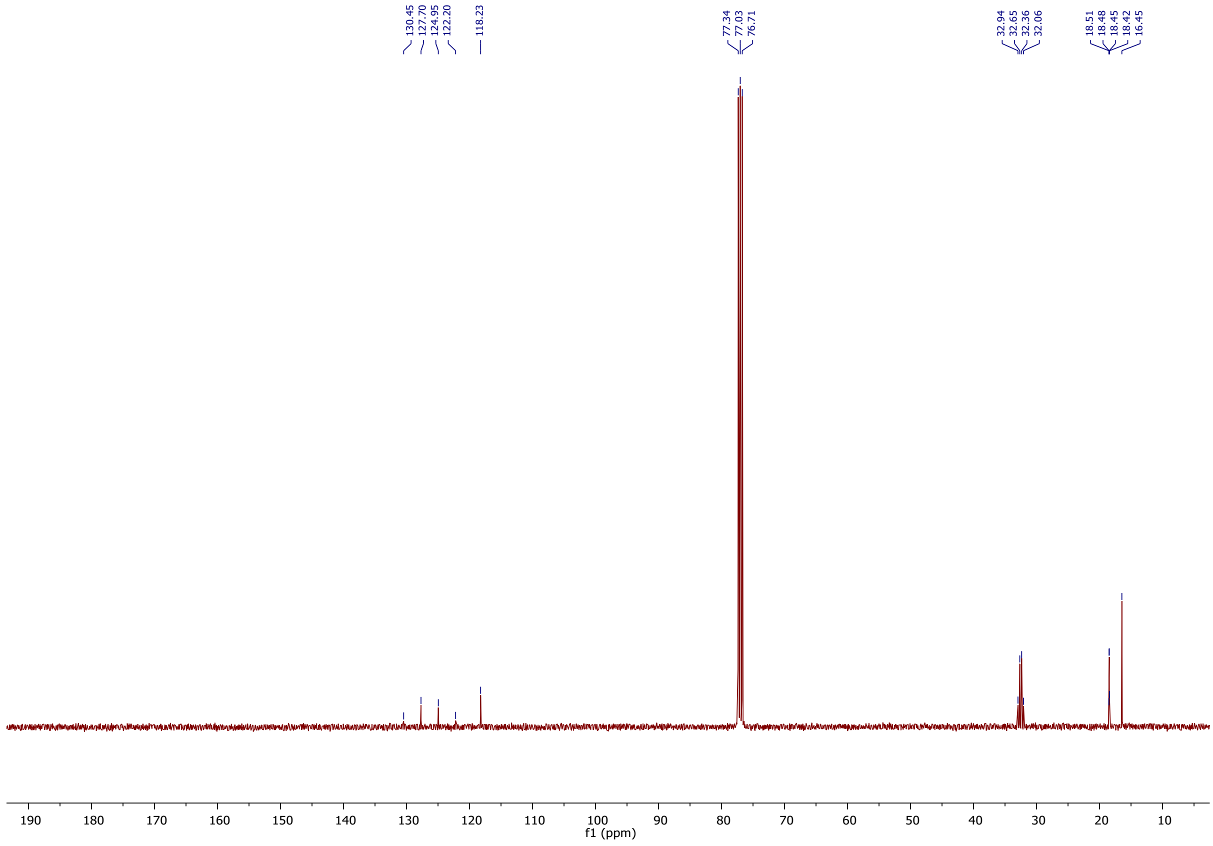
130.45
127.70
124.95
122.20
118.23
77.34
77.03
76.71
32.94
32.65
32.36
32.06
18.51
18.48
18.45
18.42
16.45
190 180 170 160 150 140 130 120 110 100 90 80 70 60 50 40 30 20 10
f1 (ppm)

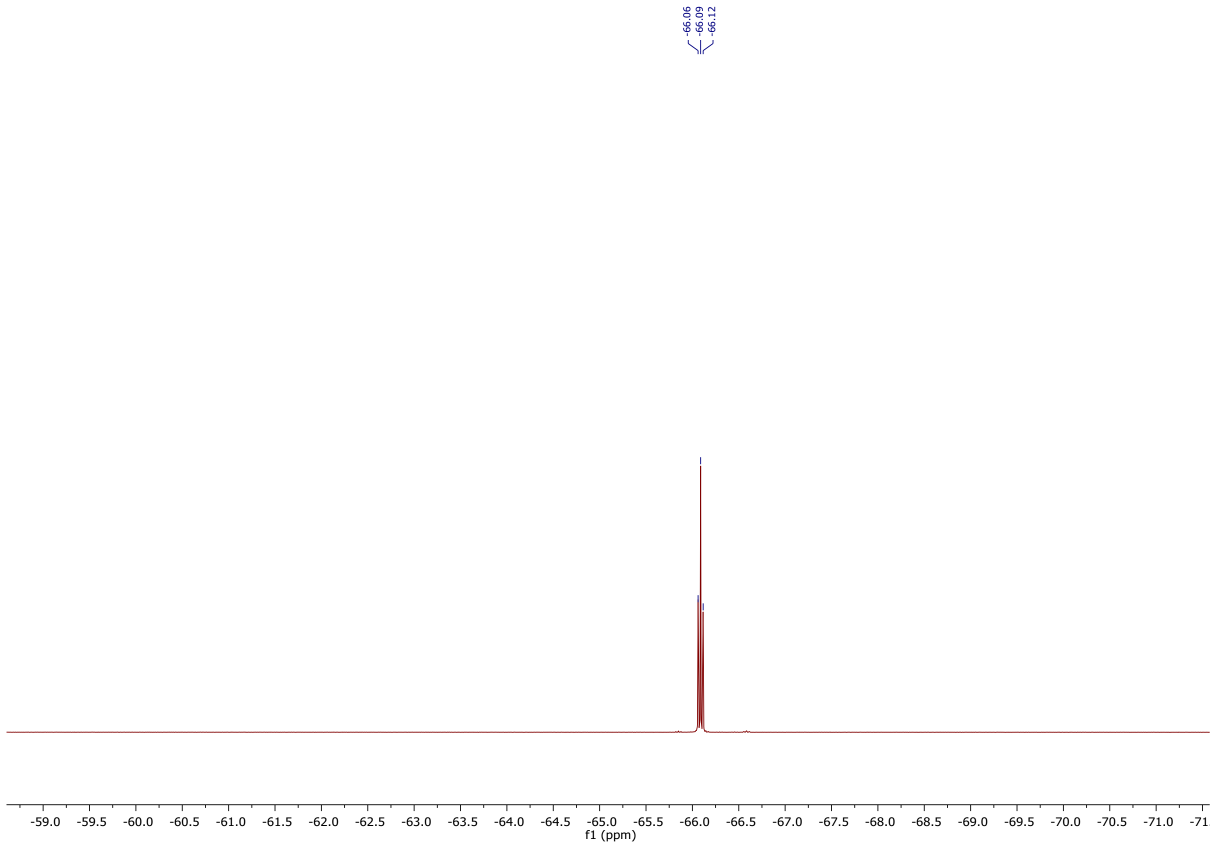
-66.06
-66.09
-66.12
-59.0 -59.5 -60.0 -60.5 -61.0 -61.5 -62.0 -62.5 -63.0 -63.5 -64.0 -64.5 -65.0 -65.5 -66.0 -66.5 -67.0 -67.5 -68.0 -68.5 -69.0 -69.5 -70.0 -70.5 -71.0 -71
f1 (ppm)

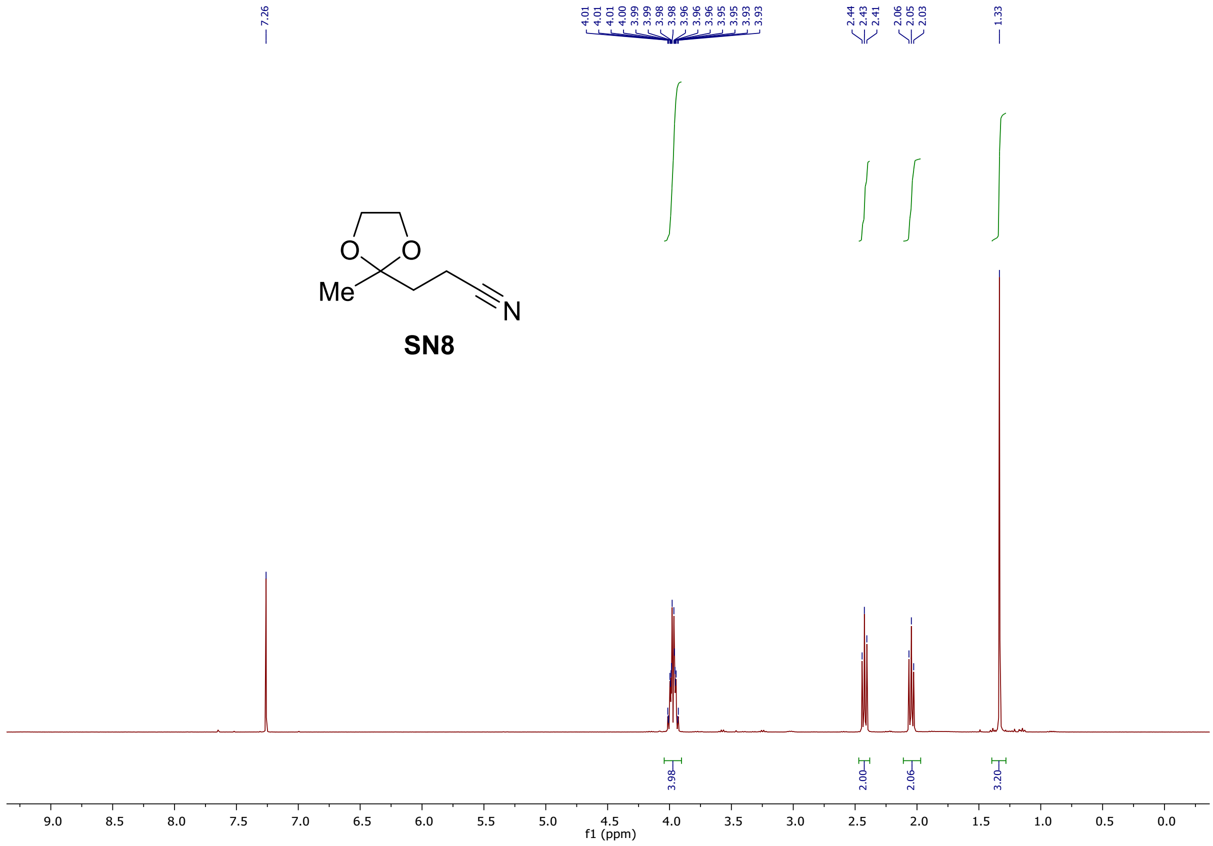
7.26
4.01
4.01
4.01
4.00
3.99
3.99
3.98
3.98
3.96
3.96
3.96
3.95
3.95
3.93
3.93
2.44
2.43
2.41
2.06
2.05
2.03
1.33
O
O
Me
N
SN8
3.98
2.00
2.06
3.20
9.0
8.5
8.0
7.5
7.0
6.5
6.0
5.5
5.0
4.5
4.0
3.5
3.0
2.5
2.0
1.5
1.0
0.5
0.0
f1 (ppm)

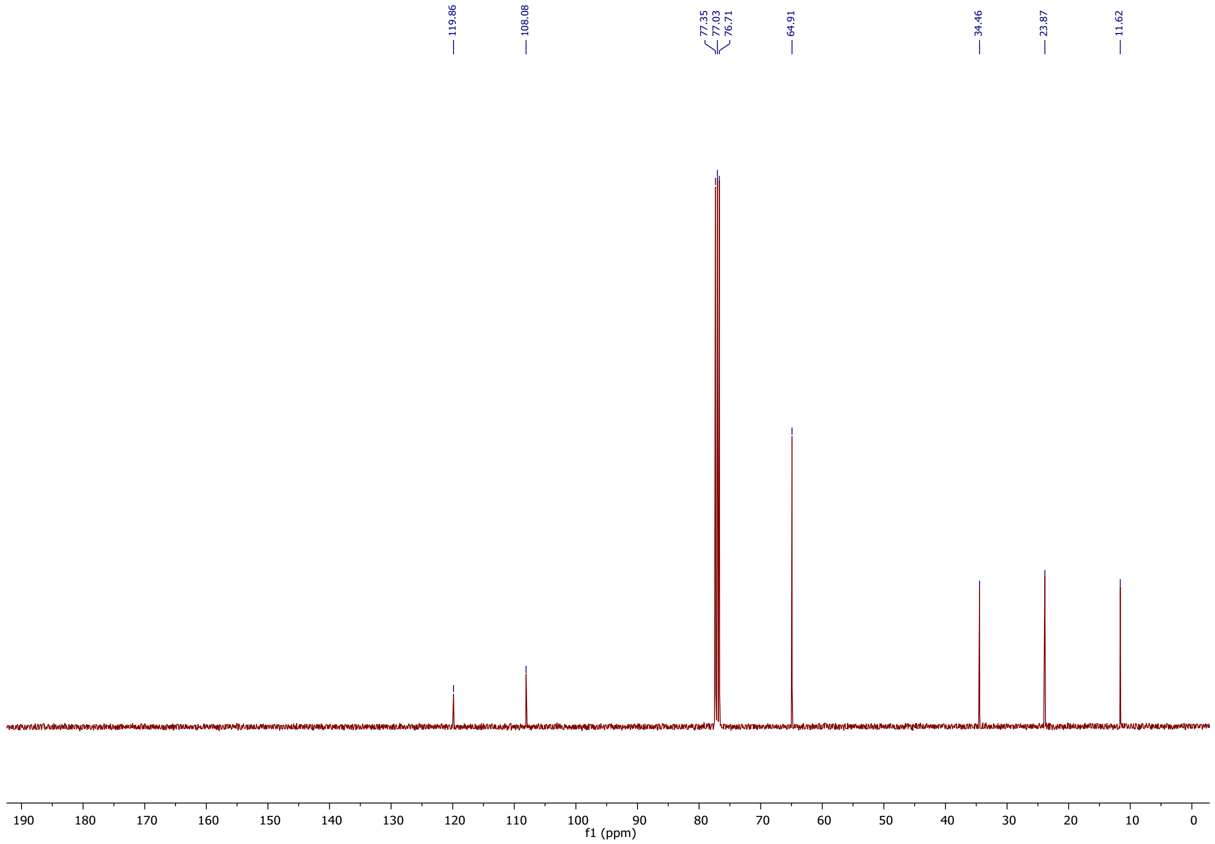
119.86
108.08
77.35
77.03
76.71
64.91
34.46
23.87
11.62
f1 (ppm)

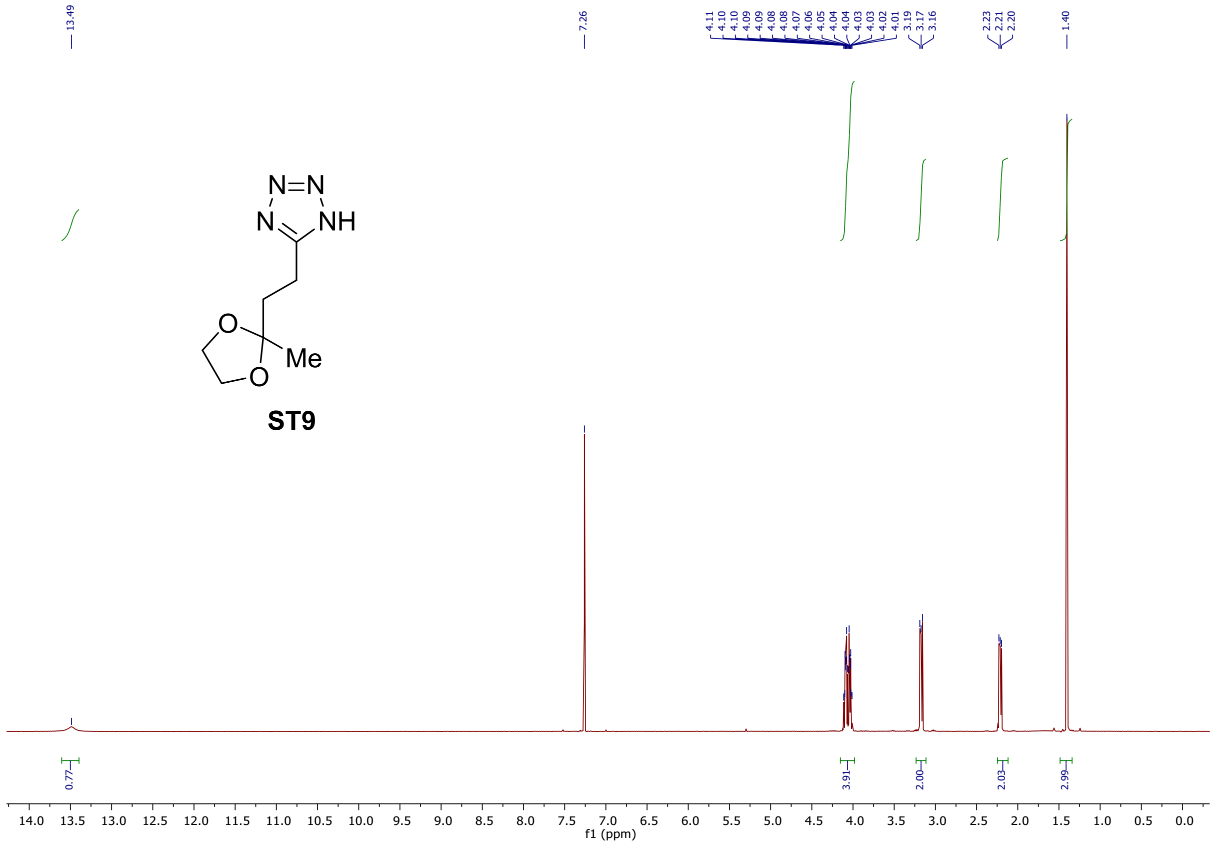
13.49
7.26
4.11
4.10
4.10
4.09
4.09
4.08
4.08
4.07
4.06
4.05
4.04
4.04
4.04
4.03
4.03
4.02
4.01
3.19
3.17
3.16
2.23
2.21
2.20
1.40
N=N
N
NH
O
O
Me
0.77
3.91
2.00
2.03
2.99
f1 (ppm)

ST9

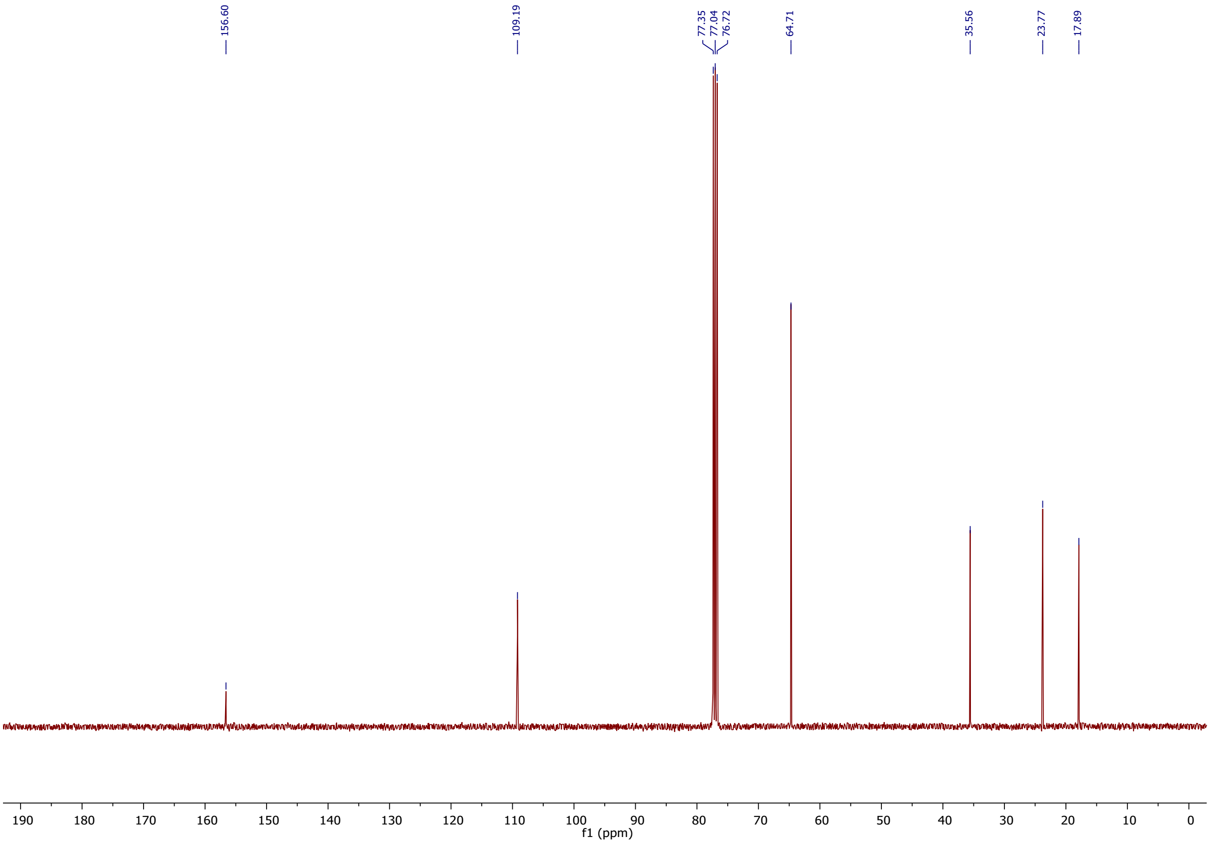
156.60
109.19
77.35
77.04
76.72
64.71
35.56
23.77
17.89
190 180 170 160 150 140 130 120 110 100 90 80 70 60 50 40 30 20 10 0
f1 (ppm)

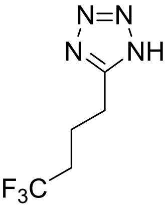
N=N
N
NH
F3C

ST10

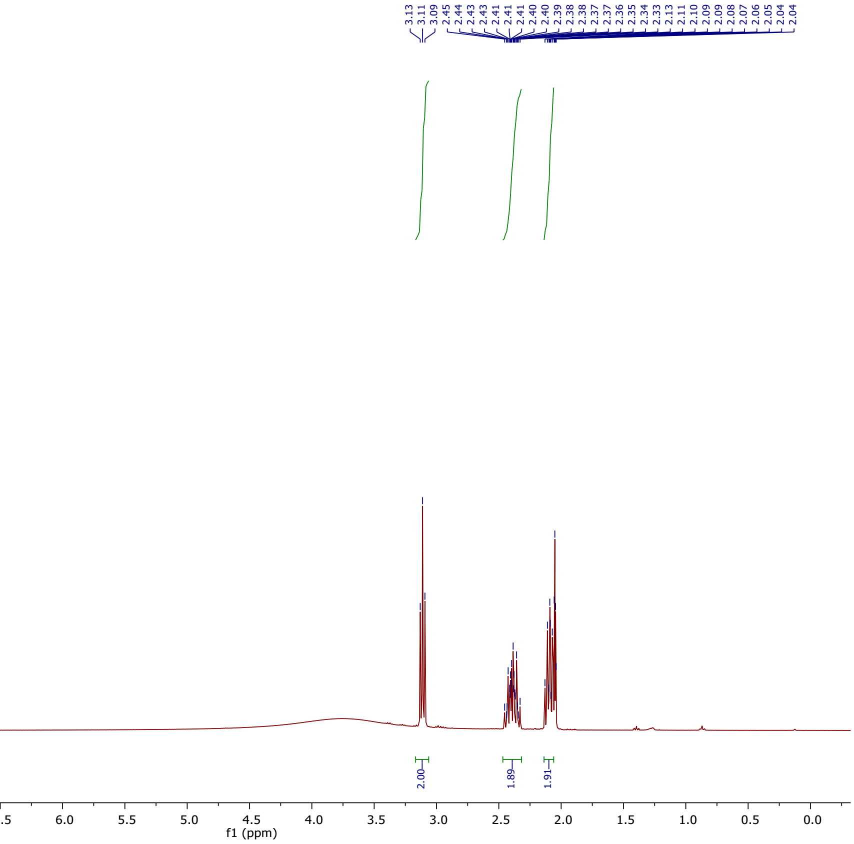
3.13
3.11
3.09
2.45
2.44
2.43
2.43
2.41
2.41
2.41
2.40
2.40
2.39
2.38
2.38
2.37
2.37
2.36
2.35
2.34
2.33
2.13
2.11
2.10
2.09
2.09
2.08
2.07
2.06
2.05
2.04
2.04
2.00
1.89
1.91
9.0 8.5 8.0 7.5 7.0 6.5 6.0 5.5 5.0 4.5 4.0 3.5 3.0 2.5 2.0 1.5 1.0 0.5 0.0
f1 (ppm)

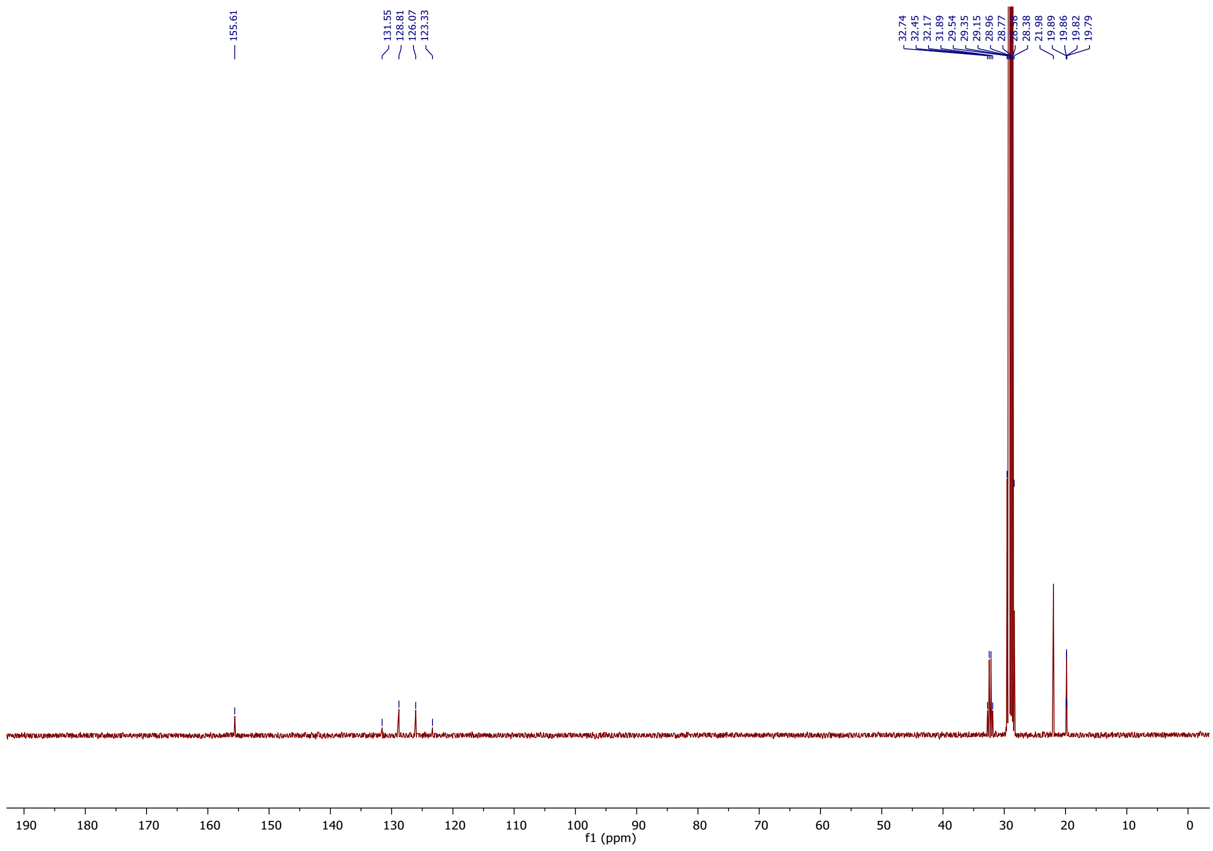
155.61
131.55
128.81
126.07
123.33
32.74
32.45
32.17
31.89
29.54
29.35
29.15
28.96
28.77
28.58
28.38
21.98
19.89
19.86
19.82
19.79
190 180 170 160 150 140 130 120 110 100 90 80 70 60 50 40 30 20 10 0
f1 (ppm)

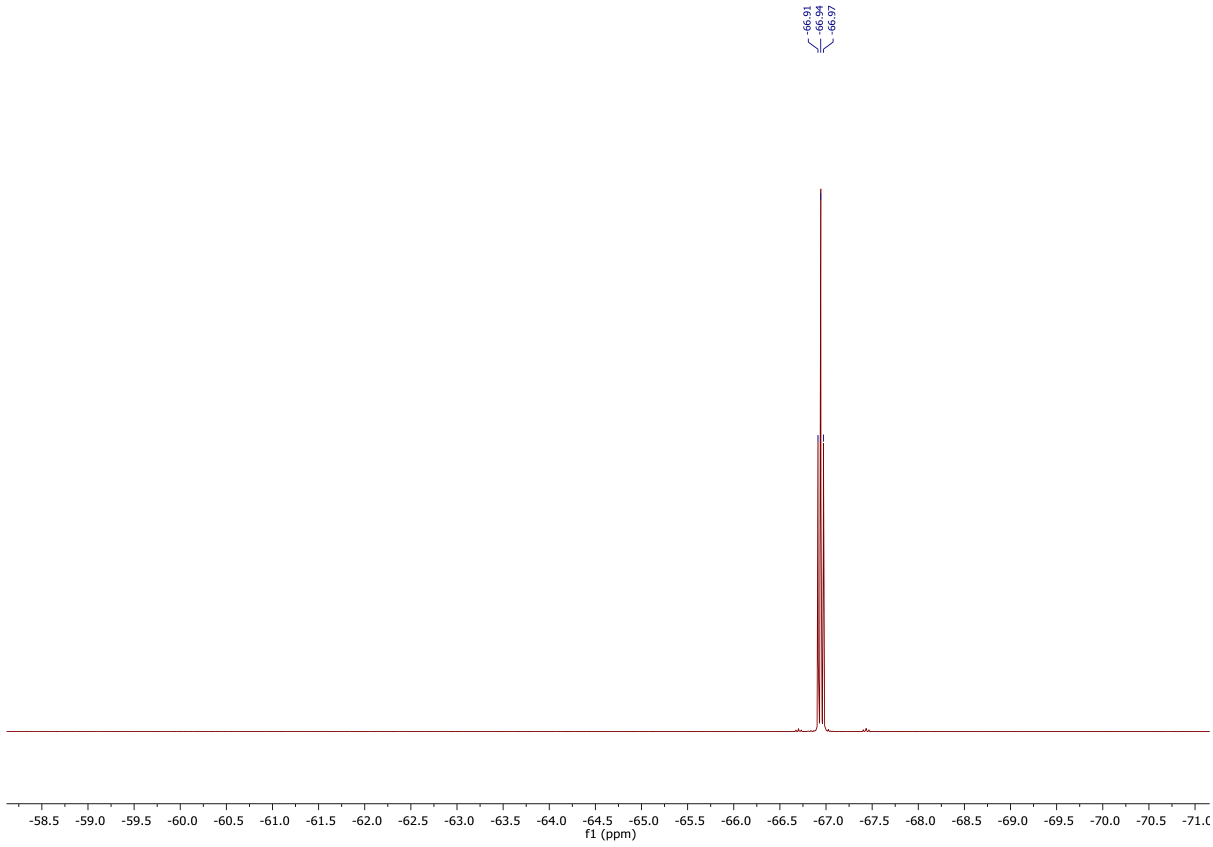
-66.91
-66.94
-66.97
-58.5 -59.0 -59.5 -60.0 -60.5 -61.0 -61.5 -62.0 -62.5 -63.0 -63.5 -64.0 -64.5 -65.0 -65.5 -66.0 -66.5 -67.0 -67.5 -68.0 -68.5 -69.0 -69.5 -70.0 -70.5
f1 (ppm)

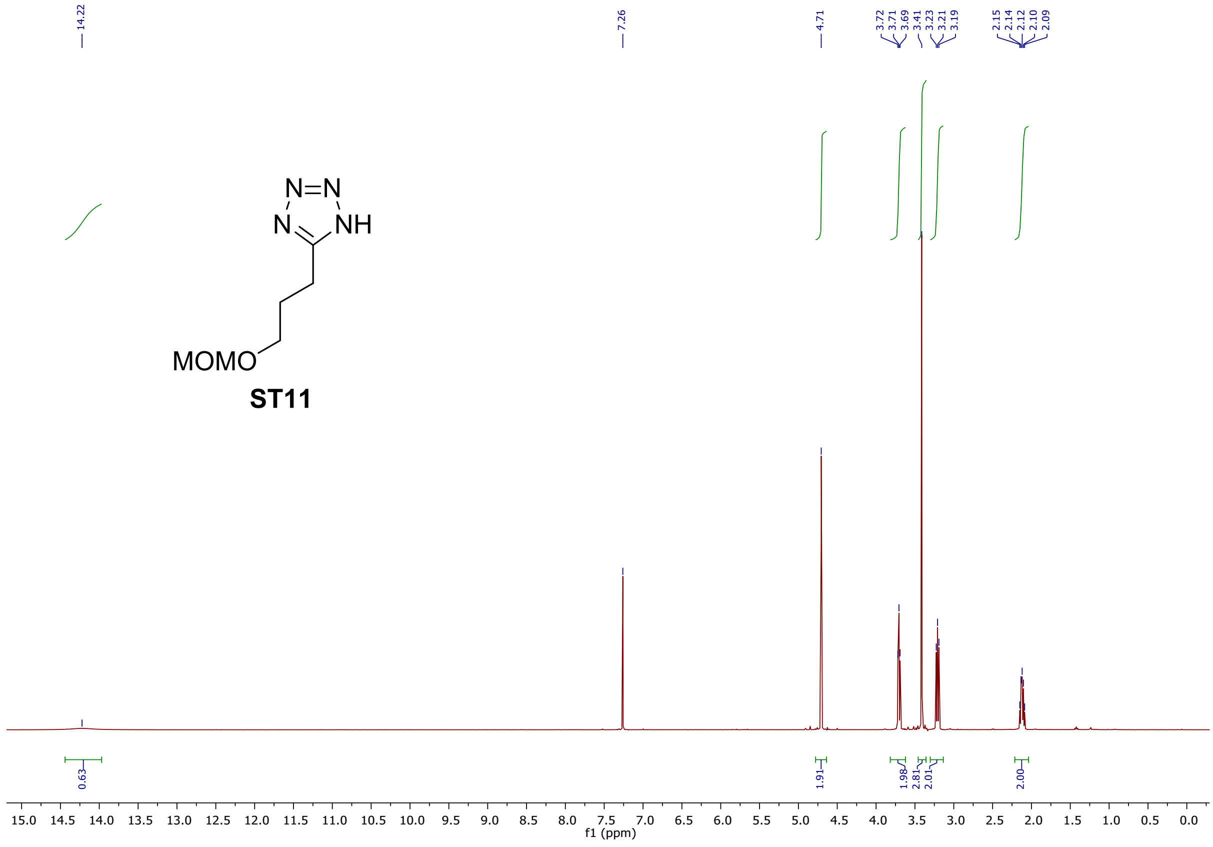
14.22
7.26
4.71
3.72
3.71
3.69
3.41
3.23
3.21
3.19
2.15
2.14
2.12
2.10
2.09
N=N
N
NH
MOMO
ST11
0.63
1.91
1.98
2.81
2.01
2.00
15.0 14.5 14.0 13.5 13.0 12.5 12.0 11.5 11.0 10.5 10.0 9.5 9.0 8.5 8.0 7.5 7.0 6.5 6.0 5.5 5.0 4.5 4.0 3.5 3.0 2.5 2.0 1.5 1.0 0.5 0.0
f1 (ppm)

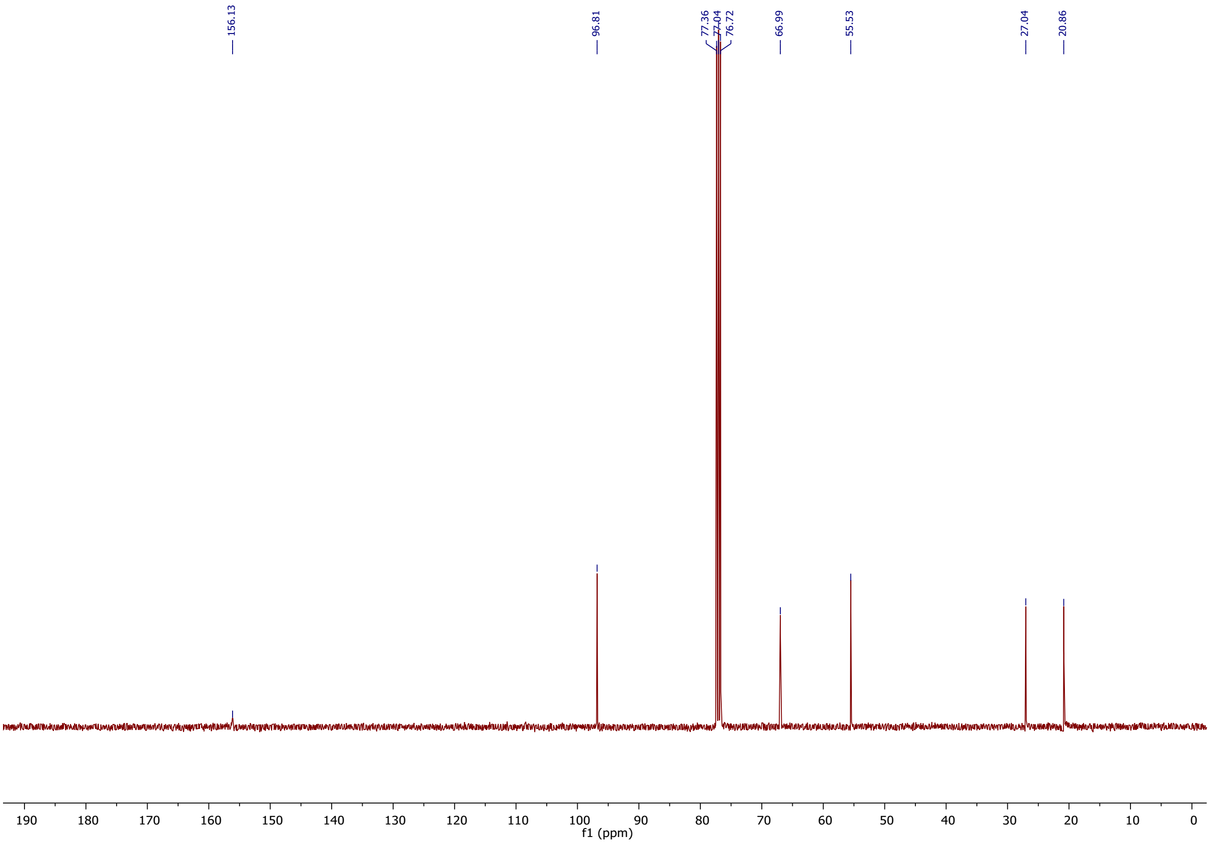
156.13
96.81
77.36
77.04
76.72
66.99
55.53
27.04
20.86
f1 (ppm)

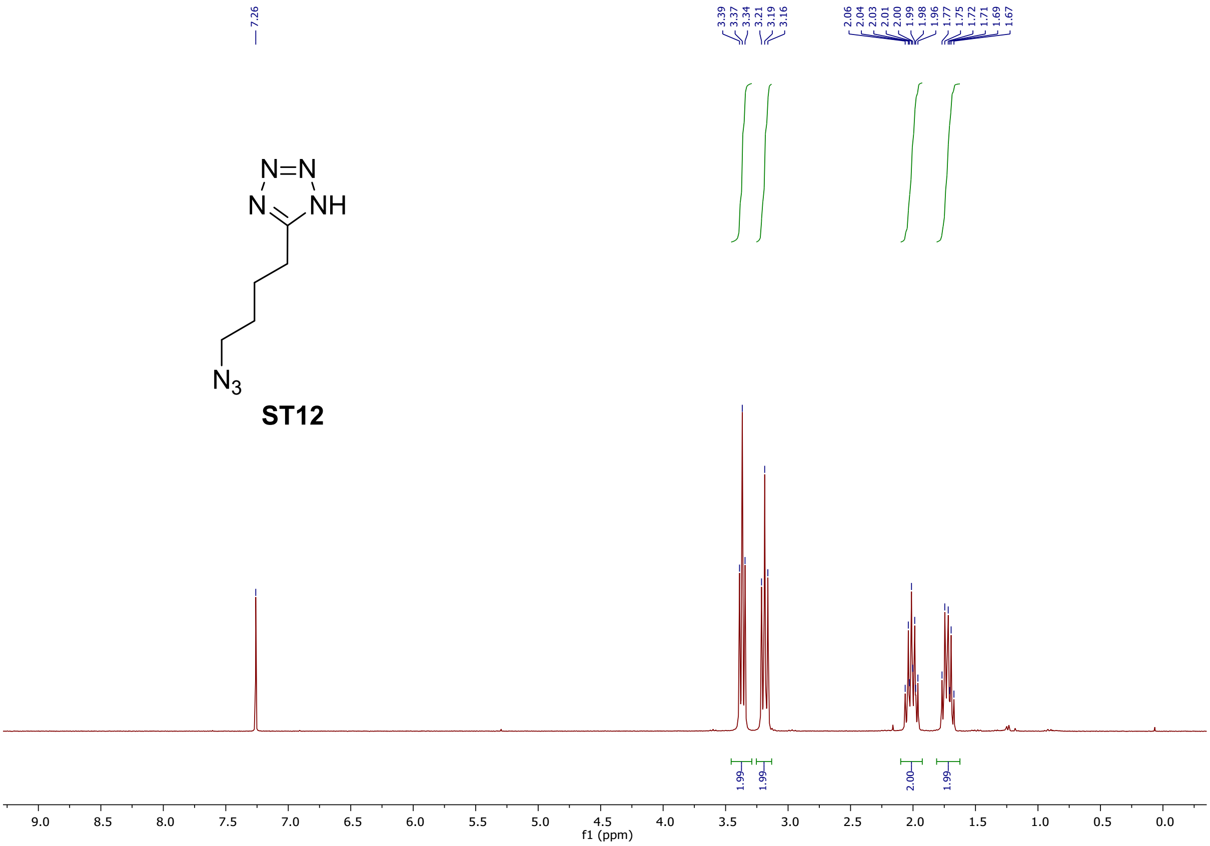
N=N
N
NH
N3
7.26
3.39
3.37
3.34
3.21
3.19
3.16
2.06
2.04
2.03
2.01
2.00
1.99
1.98
1.96
1.77
1.75
1.72
1.71
1.69
1.67
1.99
1.99
2.00
1.99
f1 (ppm)

ST12

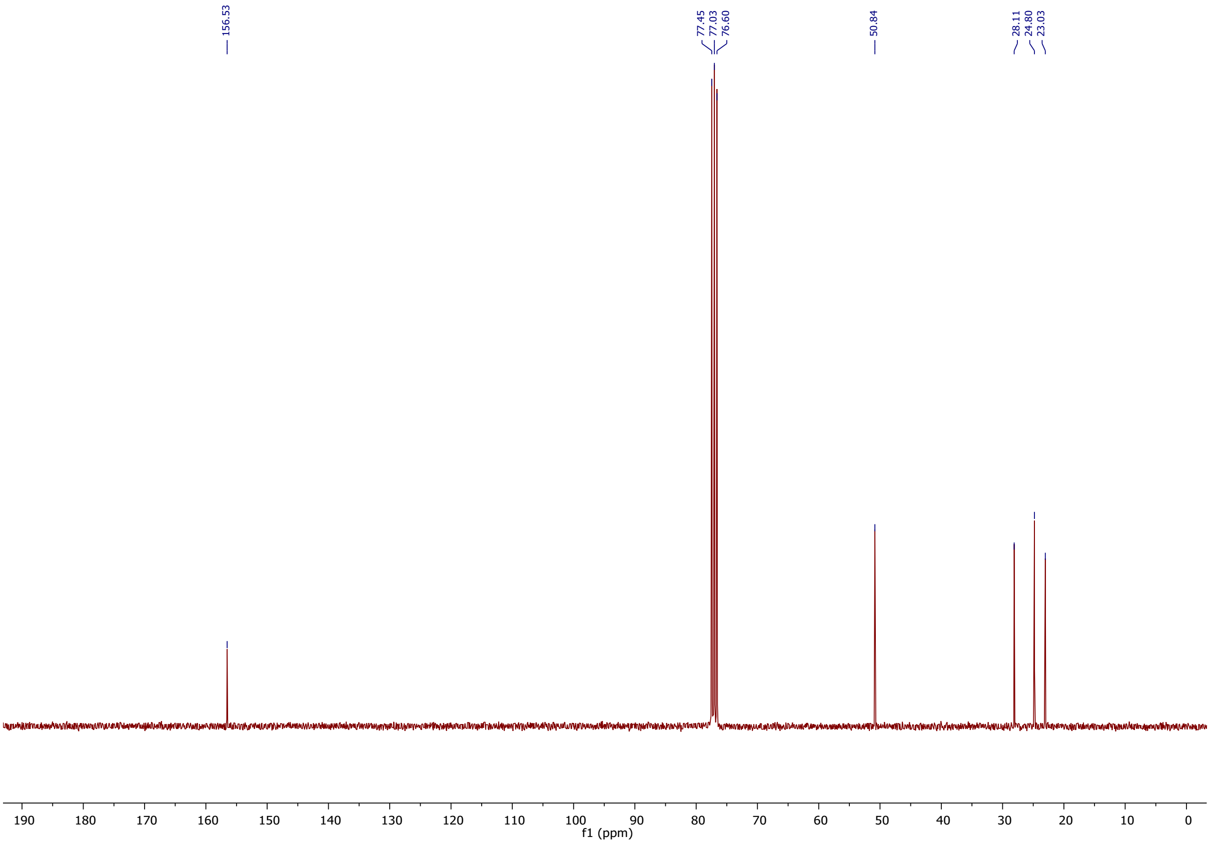
156.53
77.45
77.03
76.60
50.84
28.11
24.80
23.03
190 180 170 160 150 140 130 120 110 100 90 80 70 60 50 40 30 20 10 0
f1 (ppm)

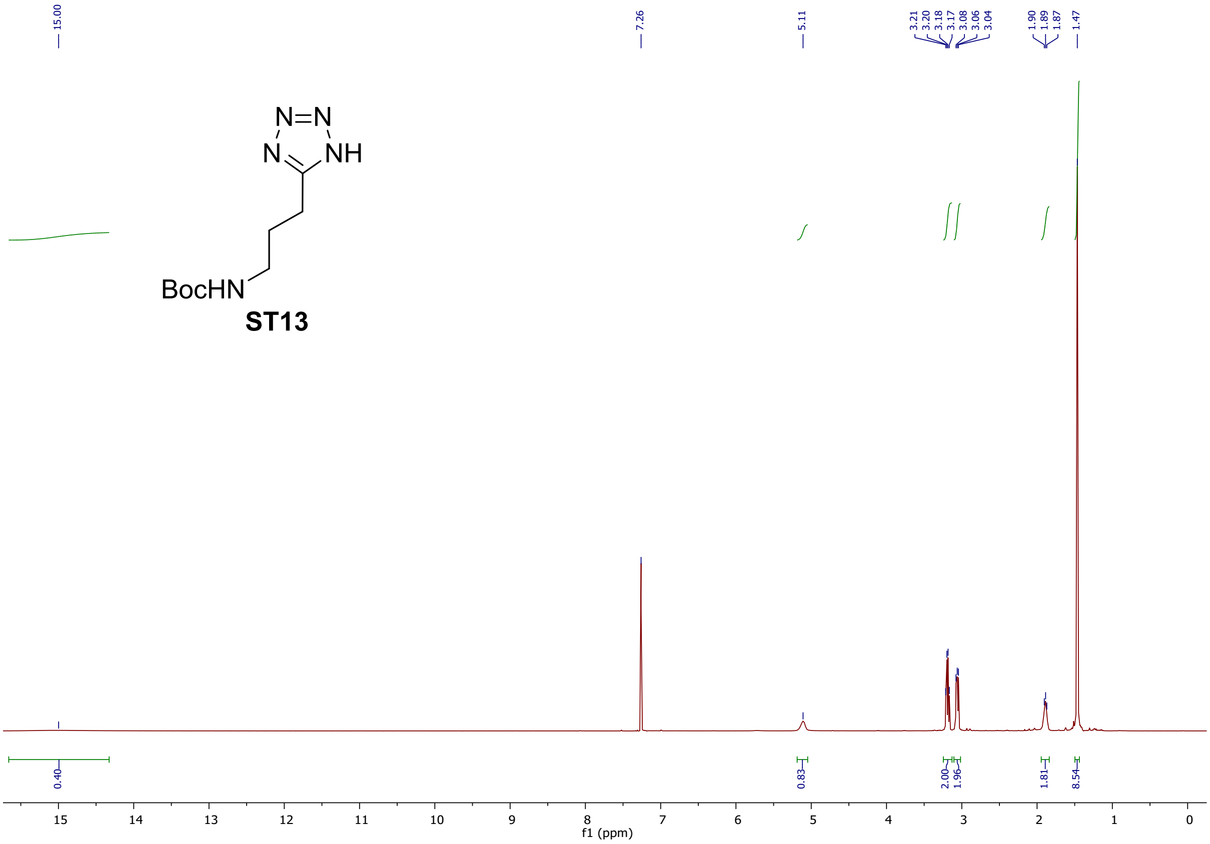
15.00
7.26
5.11
3.21
3.20
3.18
3.17
3.08
3.06
3.04
1.90
1.89
1.87
1.47
N=N
N
NH
BocHN
ST13
0.40
0.83
2.00
1.96
1.81
8.54
15 14 13 12 11 10 9 8 7 6 5 4 3 2 1 0
f1 (ppm)

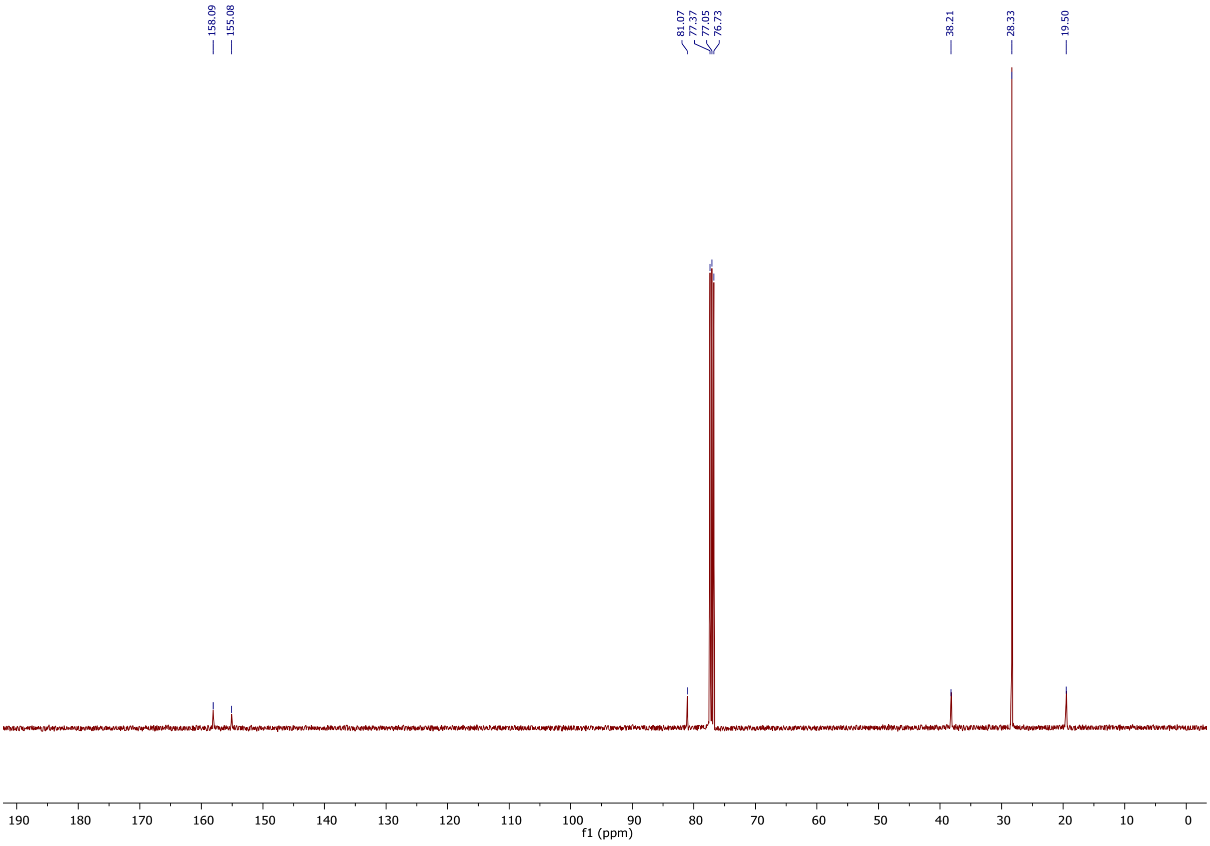
158.09
155.08
81.07
77.37
77.05
76.73
38.21
28.33
19.50
f1 (ppm)

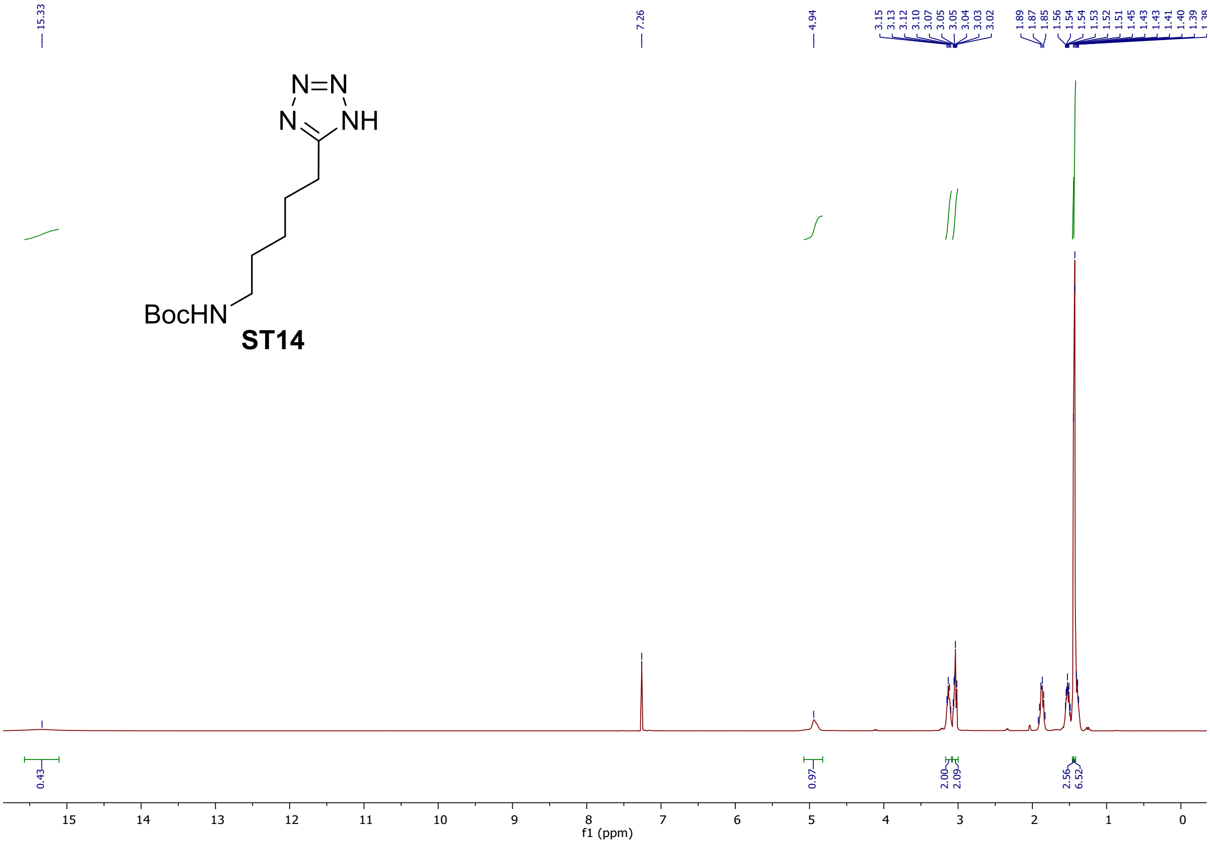
15.33
7.26
4.94
BocHN
ST14
0.43
0.97
2.00
2.09
2.56
6.52
f1 (ppm)

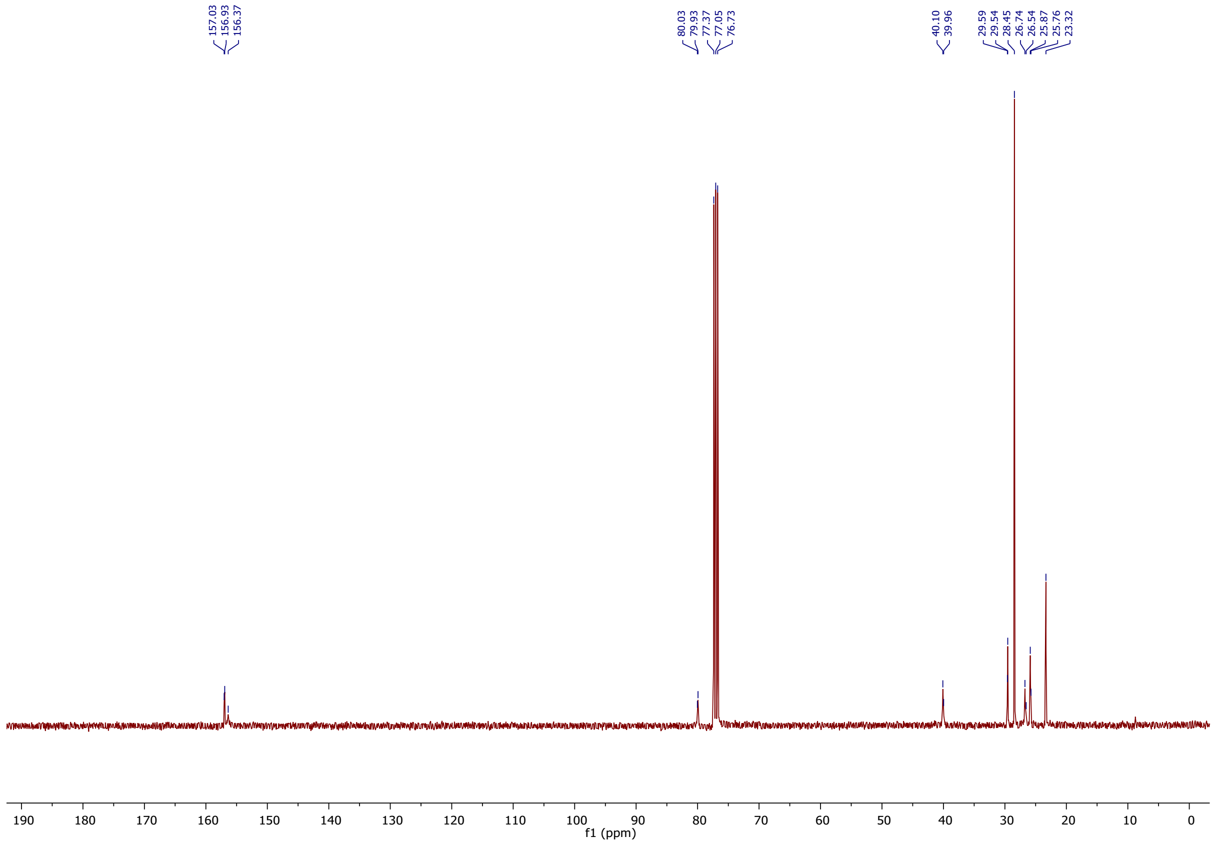
157.03
156.93
156.37
80.03
79.93
77.37
77.05
76.73
40.10
39.96
29.59
29.54
28.45
26.74
26.54
25.87
25.76
23.32
190 180 170 160 150 140 130 120 110 100 90 80 70 60 50 40 30 20 10 0
f1 (ppm)

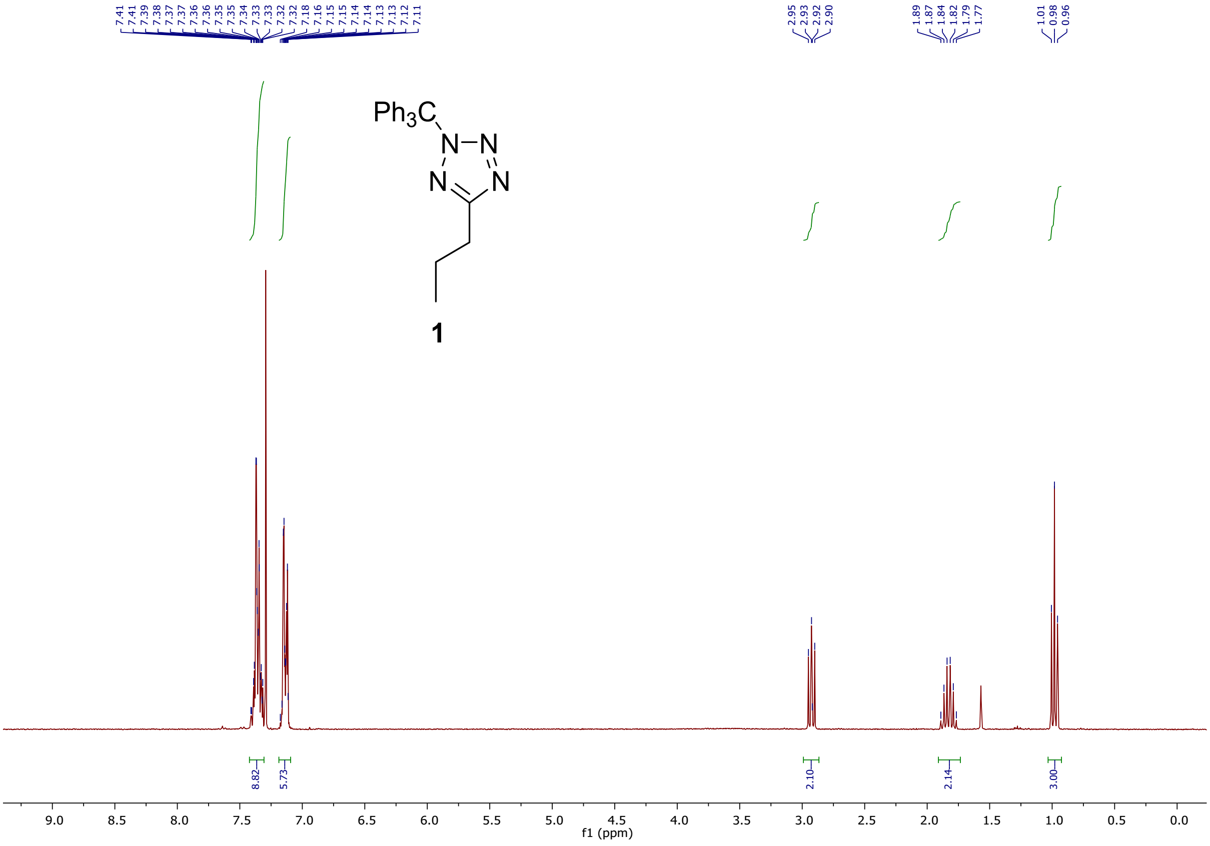
7.41 7.41 7.39 7.38 7.37 7.37 7.36 7.36 7.35 7.35 7.34 7.33 7.33 7.32 7.32 7.18 7.16 7.15 7.15 7.14 7.14 7.13 7.13 7.12 7.11
2.95 2.93 2.92 2.90
1.89 1.87 1.84 1.82 1.79 1.77
1.01 0.98 0.96
Ph3C
N
N
N
N
1
8.82
5.73
2.10
2.14
3.00
f1 (ppm)

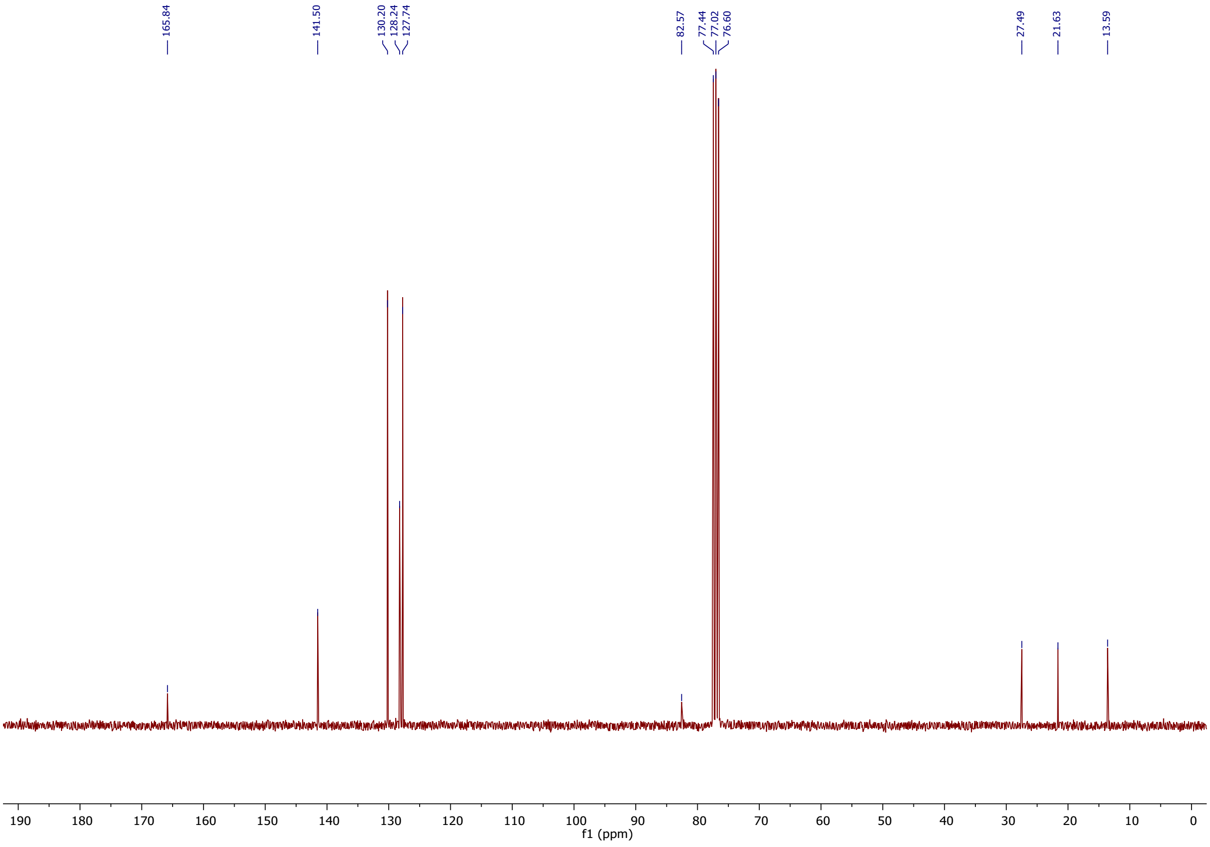
165.84
141.50
130.20 128.24 127.74
82.57
77.44 77.02 76.60
27.49
21.63
13.59
f1 (ppm)

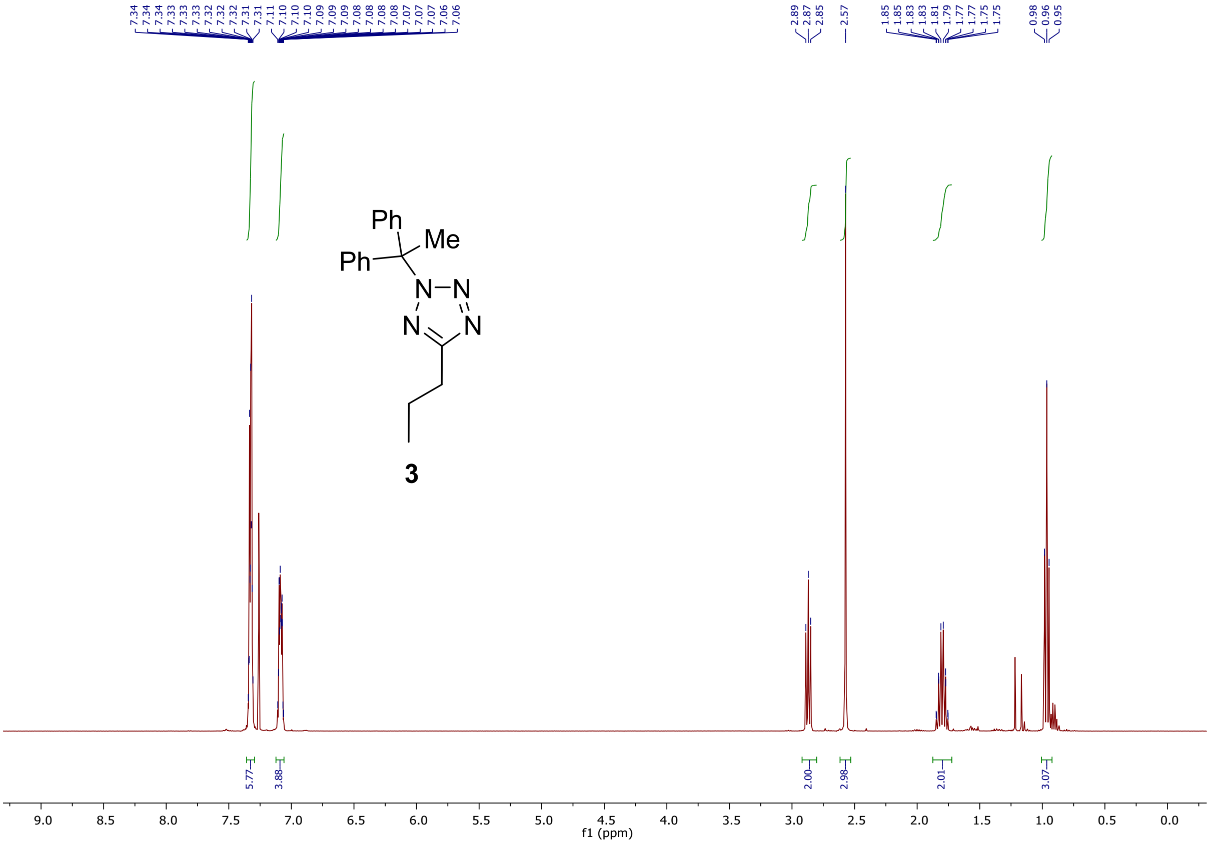
7.34 7.34 7.34 7.33 7.33 7.33 7.32 7.32 7.32 7.31 7.31 7.11 7.10 7.10 7.10 7.09 7.09 7.09 7.08 7.08 7.08 7.08 7.07 7.07 7.07 7.06 7.06
2.89 2.87 2.85
2.57
1.85 1.85 1.83 1.83 1.81 1.79 1.77 1.77 1.75 1.75
0.98 0.96 0.95
Ph
Me
Ph
N
N
N
N
3
5.77
3.88
2.00
2.98
2.01
3.07
9.0 8.5 8.0 7.5 7.0 6.5 6.0 5.5 5.0 4.5 4.0 3.5 3.0 2.5 2.0 1.5 1.0 0.5 0.0
f1 (ppm)

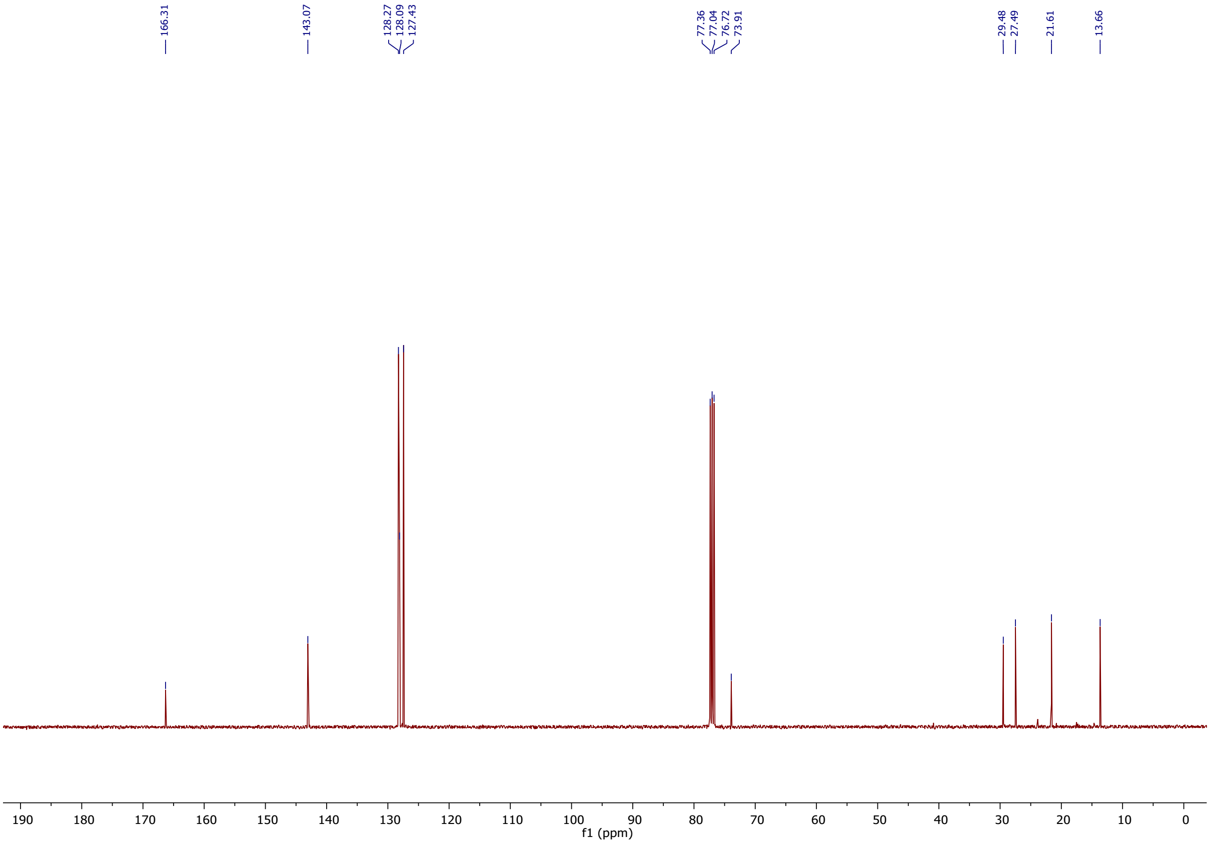
166.31
143.07
128.27 128.09 127.43
77.36 77.04 76.72 73.91
29.48 27.49
21.61
13.66
190 180 170 160 150 140 130 120 110 100 90 80 70 60 50 40 30 20 10 0
f1 (ppm)

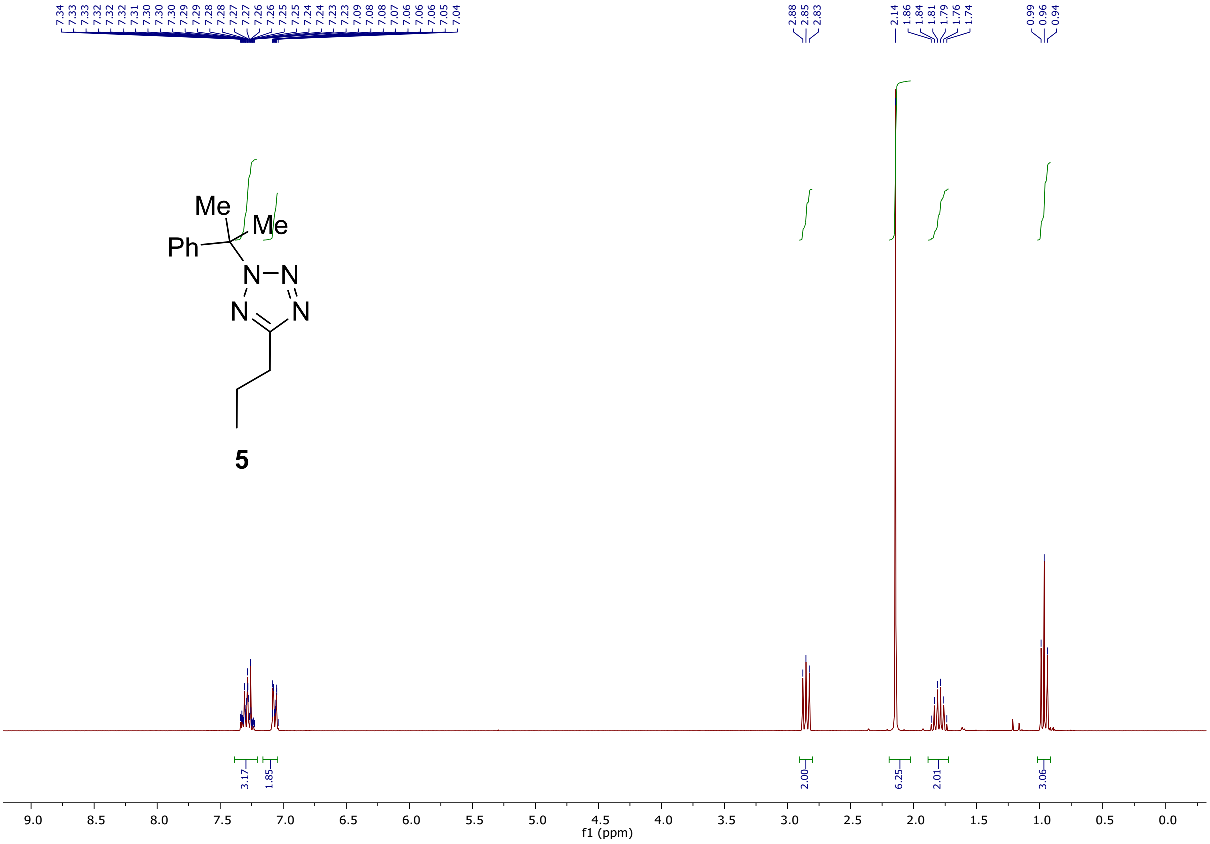

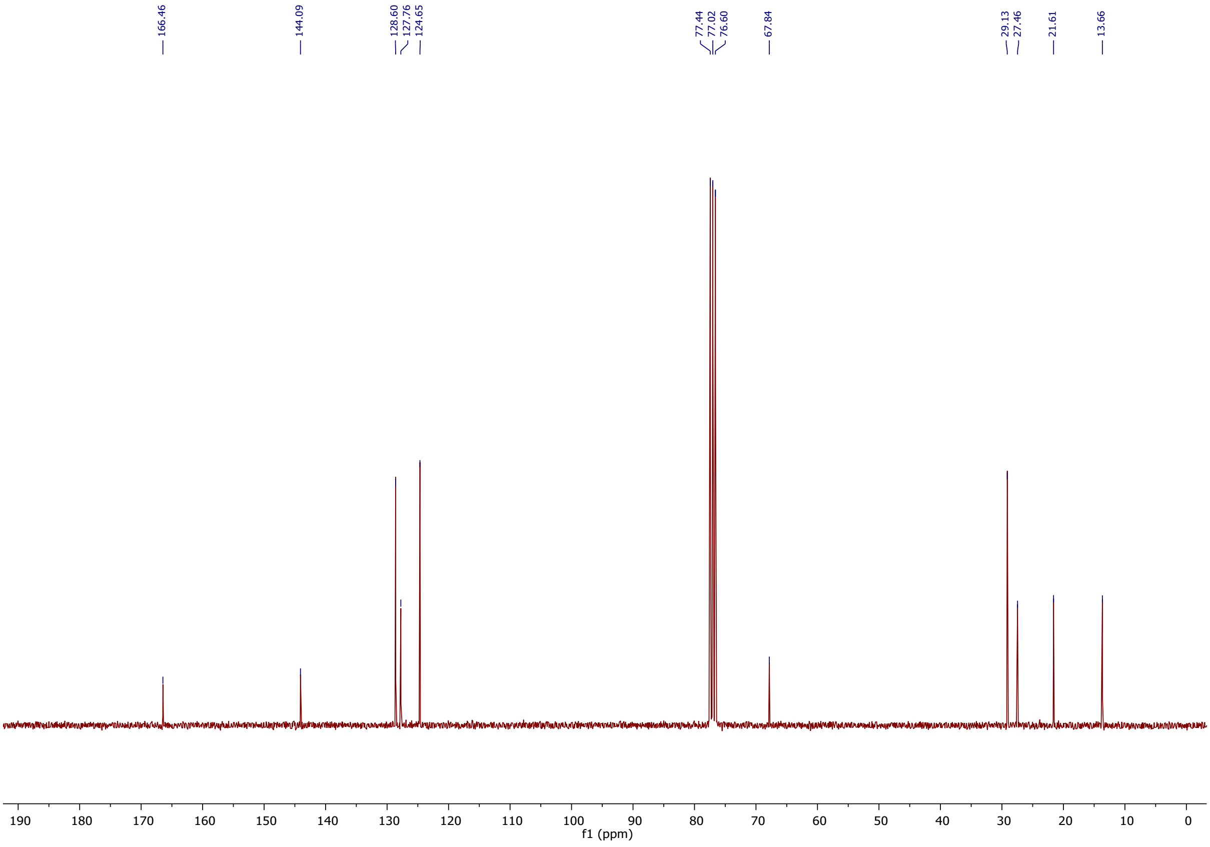

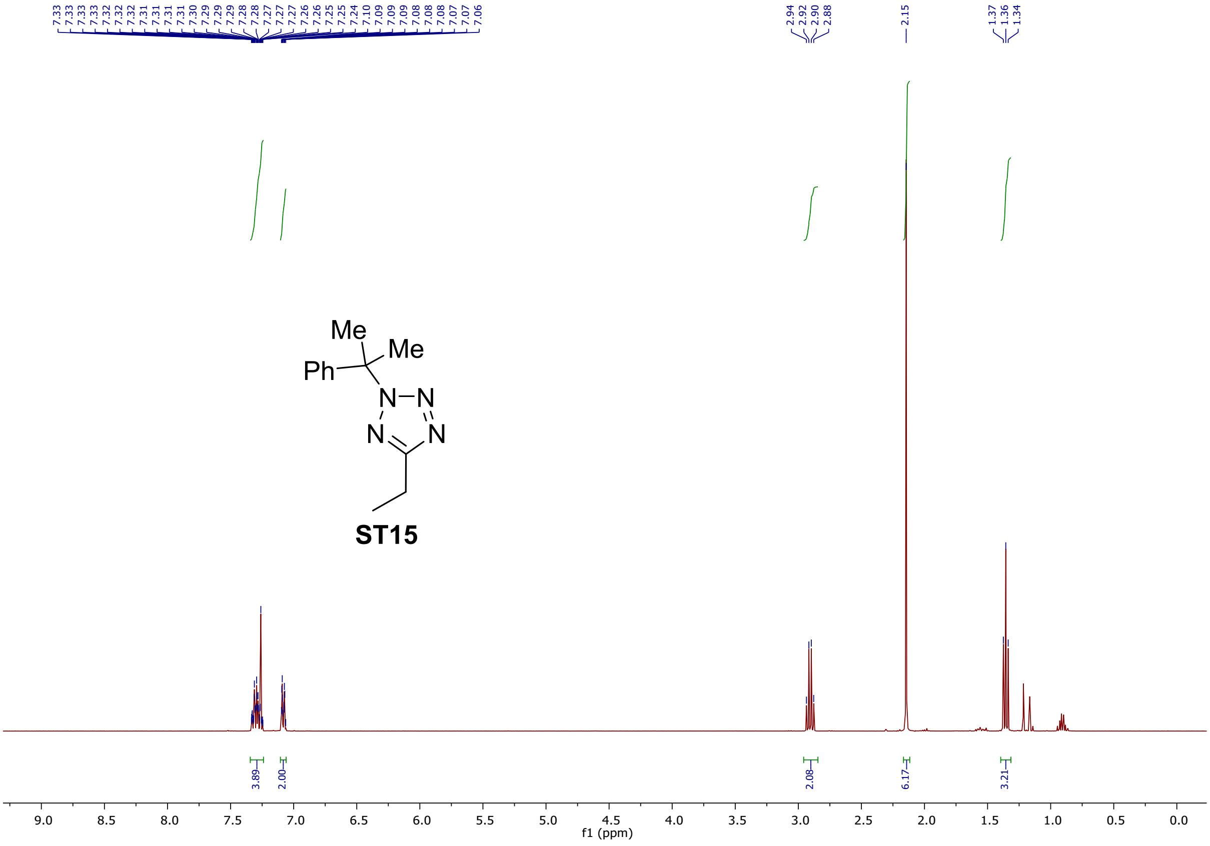

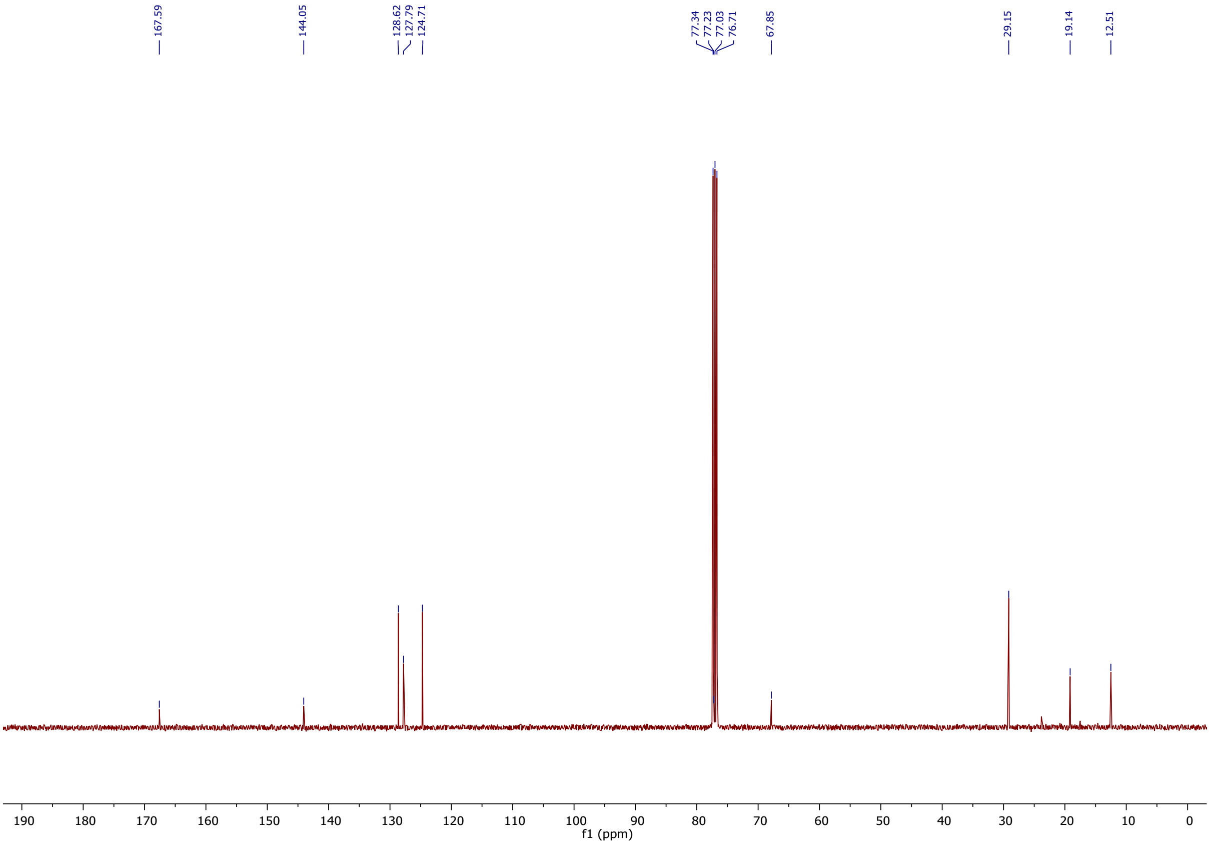

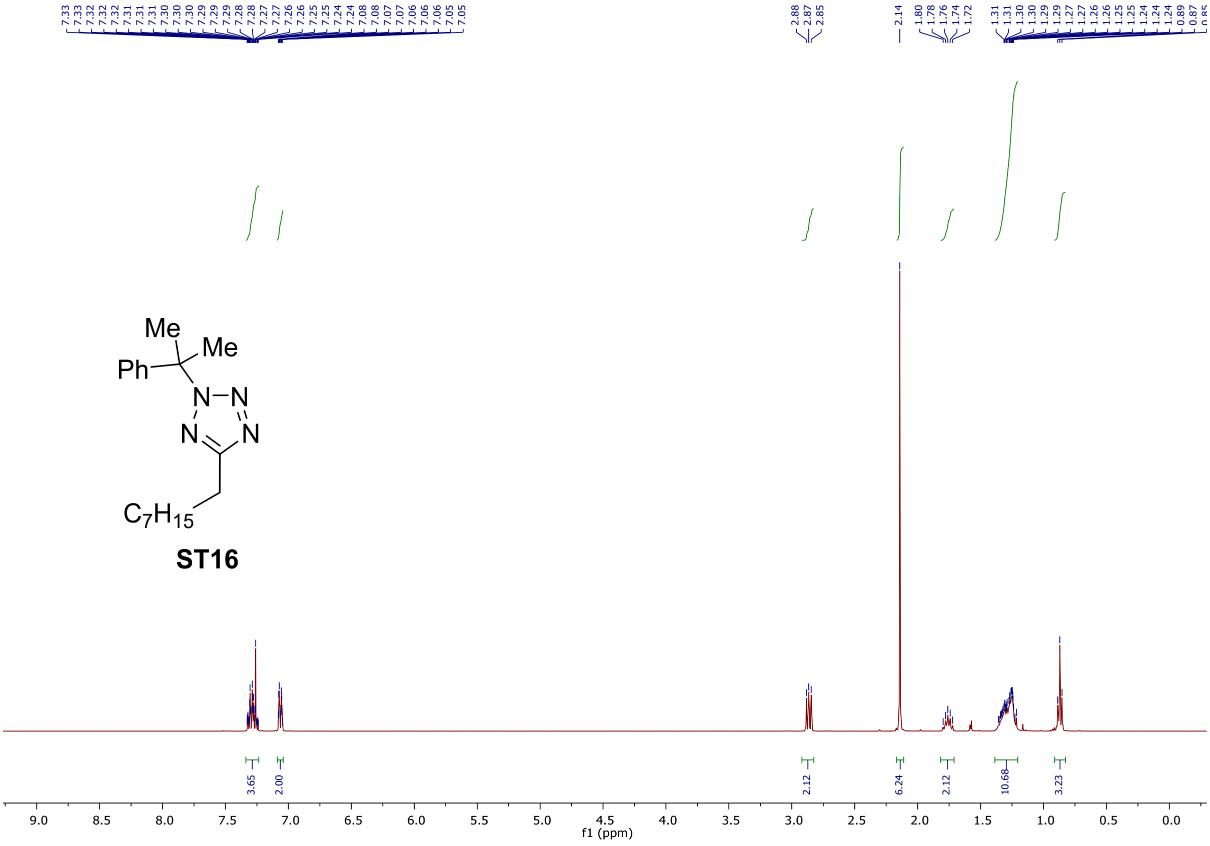

ST16

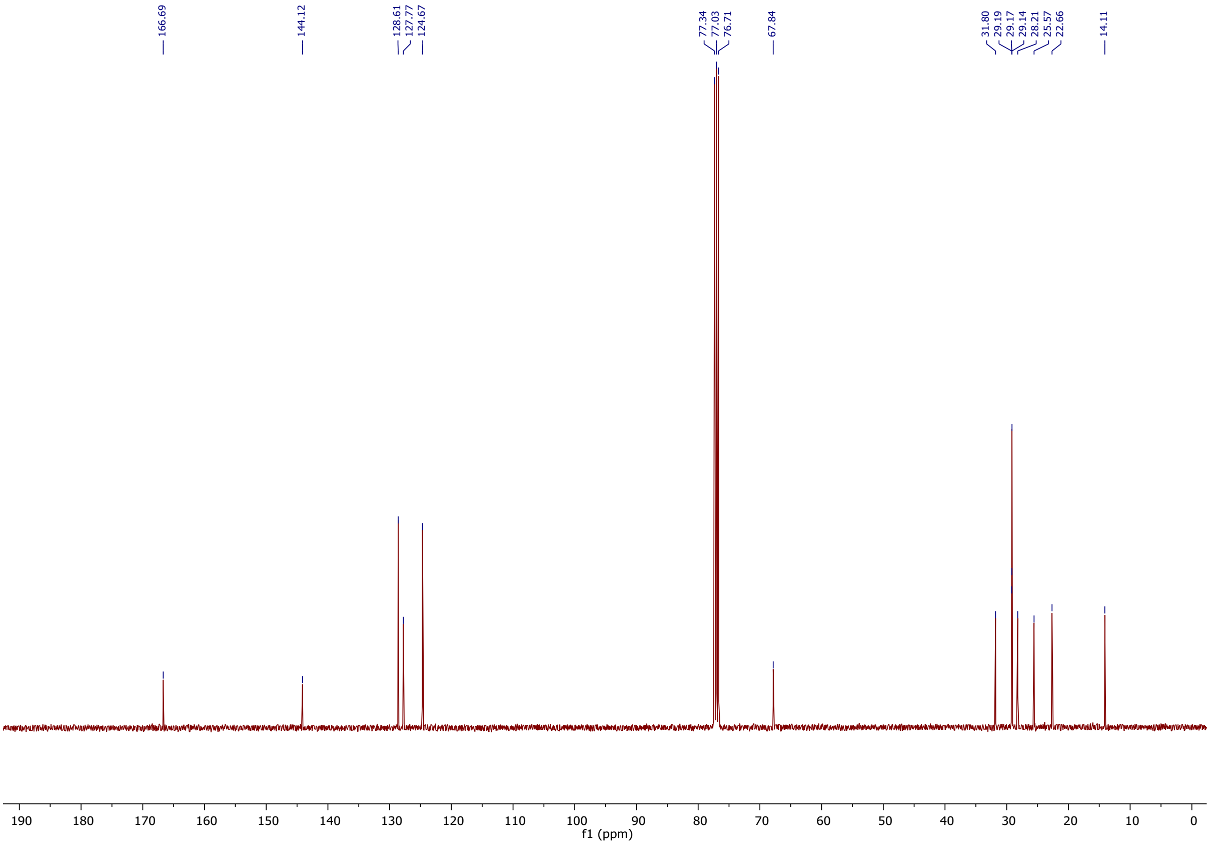

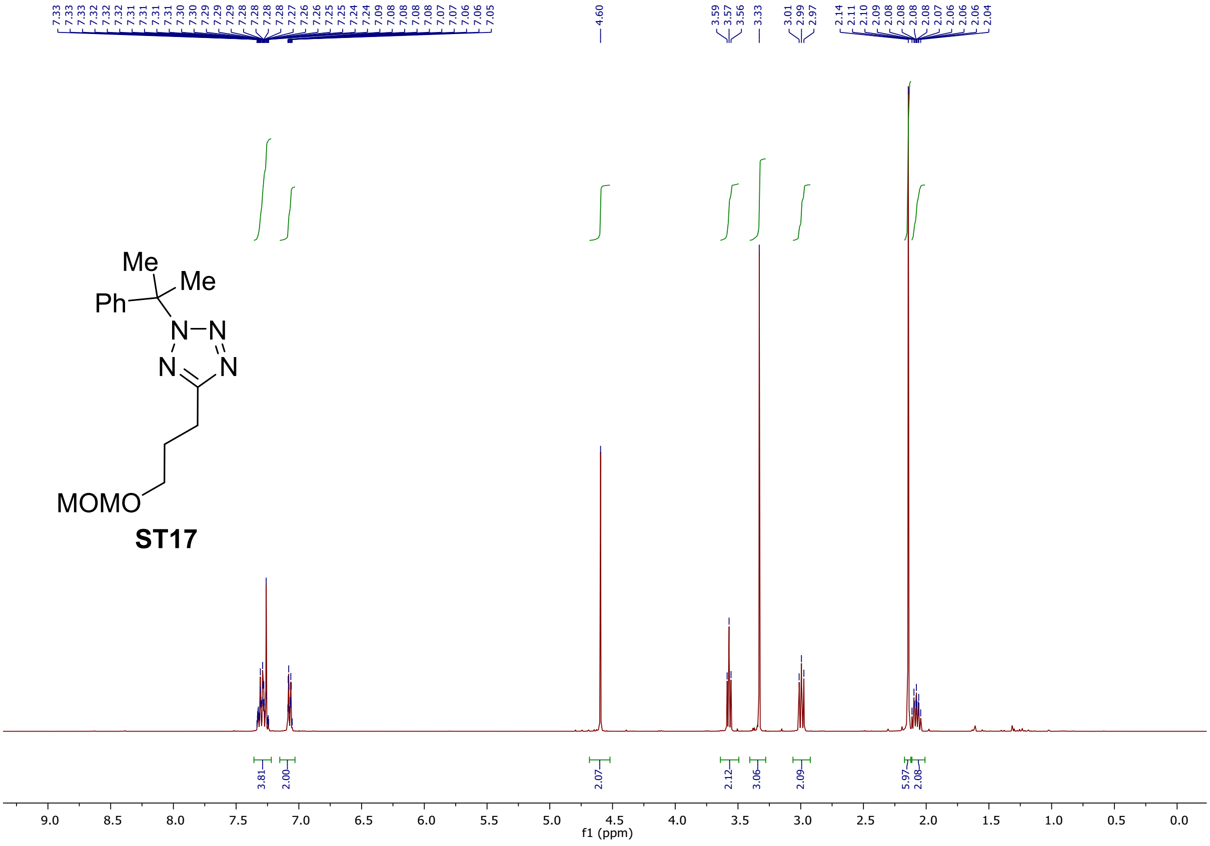
7.33
7.33
7.33
7.32
7.32
7.32
7.31
7.31
7.31
7.31
7.30
7.30
7.29
7.29
7.29
7.28
7.28
7.28
7.28
7.28
7.27
7.26
7.26
7.25
7.25
7.24
7.24
7.09
7.08
7.08
7.08
7.08
7.07
7.07
7.06
7.06
7.05
4.60
3.59
3.57
3.56
3.33
3.01
2.99
2.97
2.14
2.11
2.10
2.09
2.08
2.08
2.08
2.08
2.07
2.06
2.06
2.06
2.04
3.81
2.00
2.07
2.12
3.06
2.09
5.97
2.08
9.0 8.5 8.0 7.5 7.0 6.5 6.0 5.5 5.0 4.5 4.0 3.5 3.0 2.5 2.0 1.5 1.0 0.5 0.0
f1 (ppm)
Me
Me
Ph
N
N
N
N
MOMO

ST17

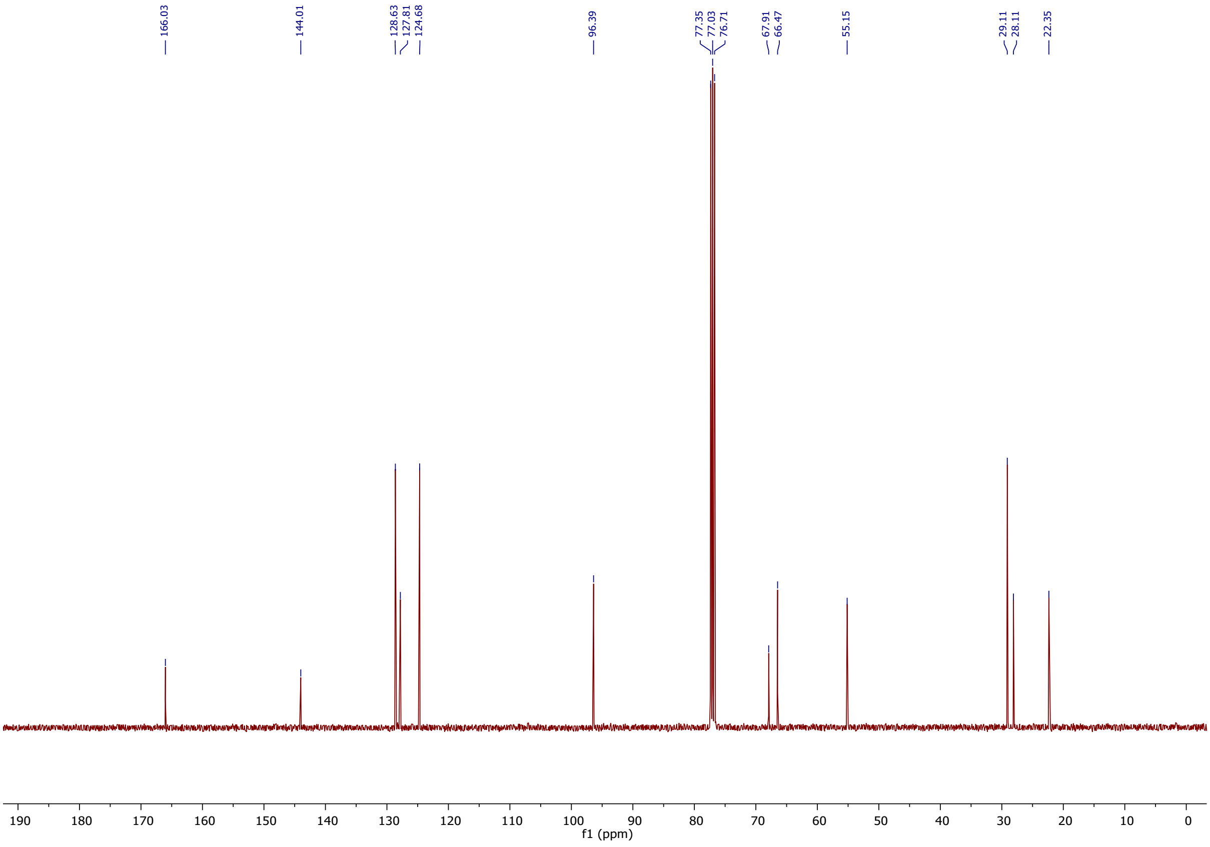
166.03
144.01
128.63
127.81
124.68
96.39
77.35
77.03
76.71
67.91
66.47
55.15
29.11
28.11
22.35
190 180 170 160 150 140 130 120 110 100 90 80 70 60 50 40 30 20 10 0
f1 (ppm)

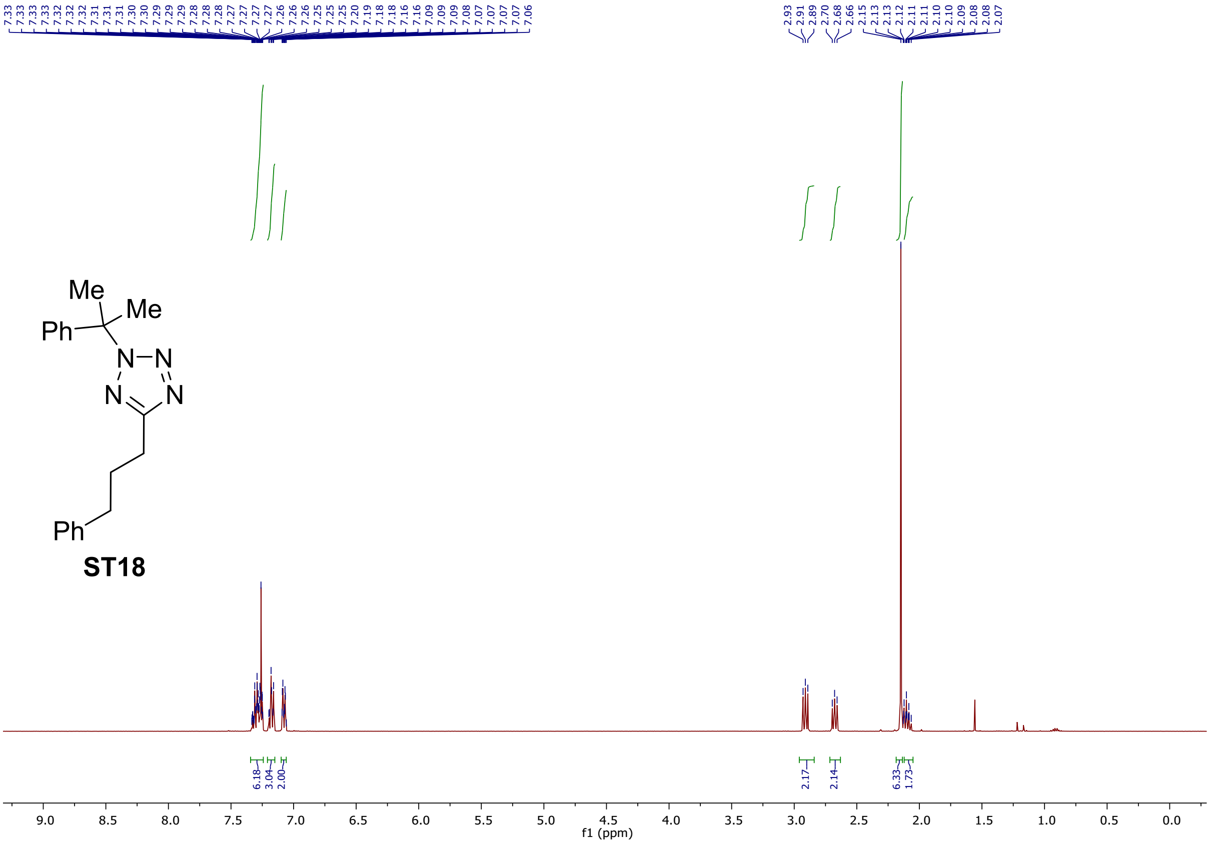
7.33 7.33 7.33 7.32 7.32 7.31 7.31 7.31 7.30 7.29 7.29 7.29 7.28 7.28 7.27 7.27 7.27 7.26 7.26 7.25 7.25 7.20 7.19 7.18 7.18 7.16 7.16 7.09 7.09 7.08 7.07 7.07 7.07 7.06
2.93 2.91 2.89 2.70 2.68 2.66 2.15 2.13 2.13 2.12 2.11 2.11 2.10 2.10 2.09 2.08 2.08 2.07
Me
Me
Ph
N
N
N
N
Ph
6.18
3.04
2.00
2.17
2.14
6.33
1.73
f1 (ppm)

ST18

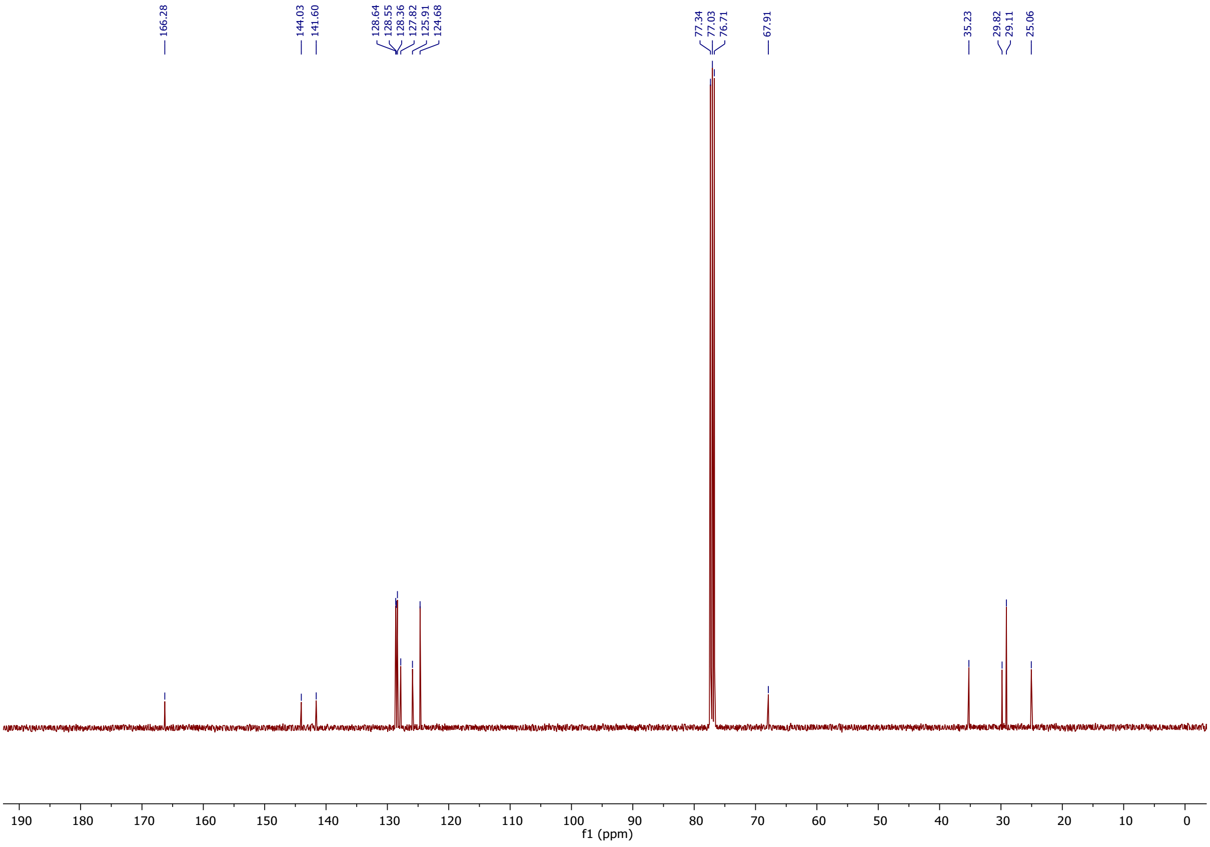
166.28
144.03 141.60
128.64 128.55 128.36 127.82 125.91 124.68
77.34 77.03 76.71
67.91
35.23
29.82 29.11
25.06
f1 (ppm)

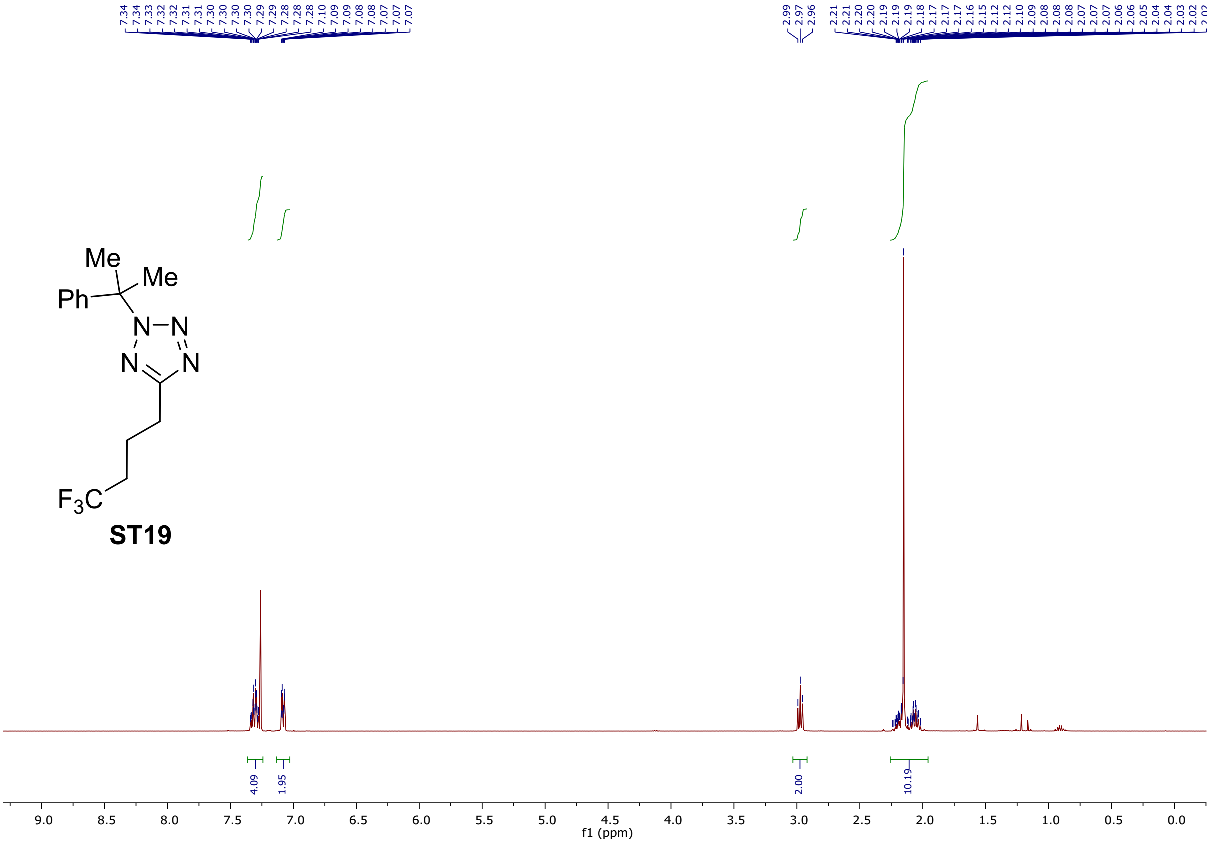

ST19

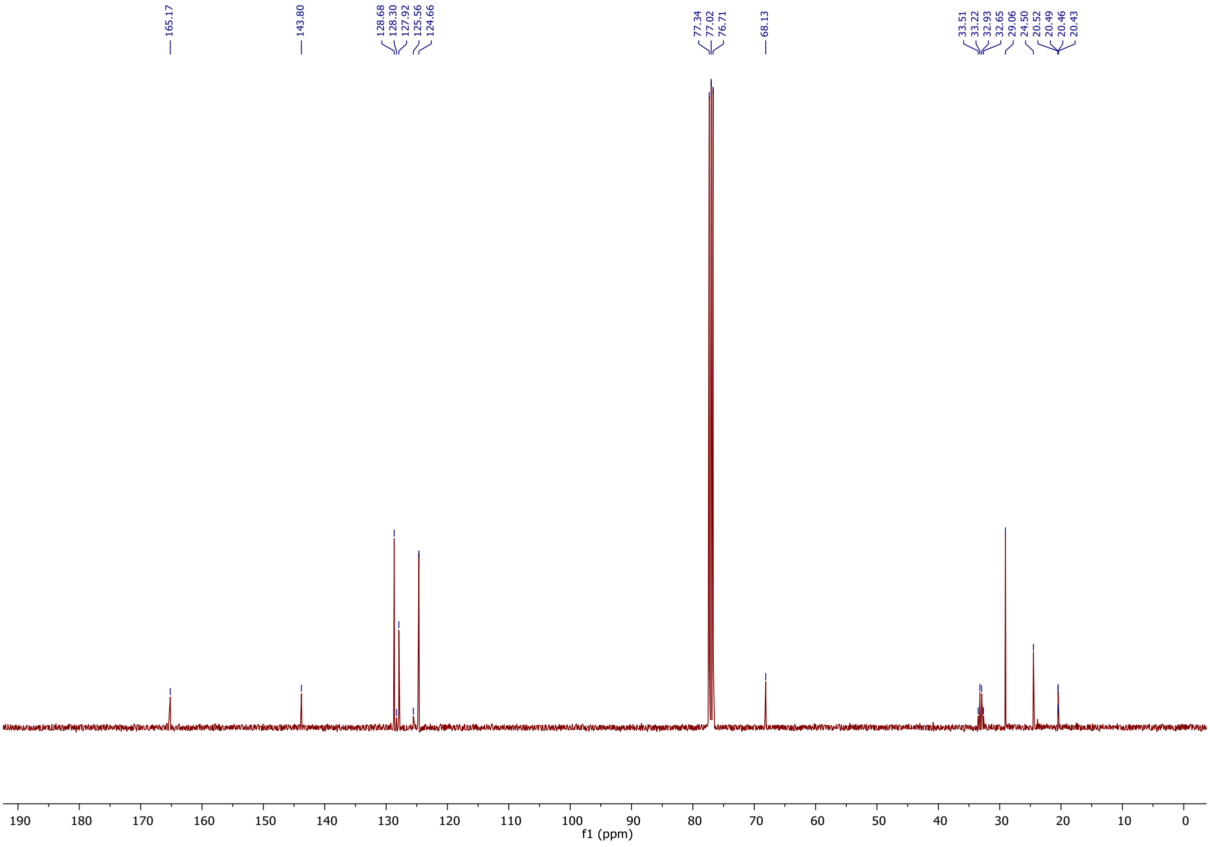

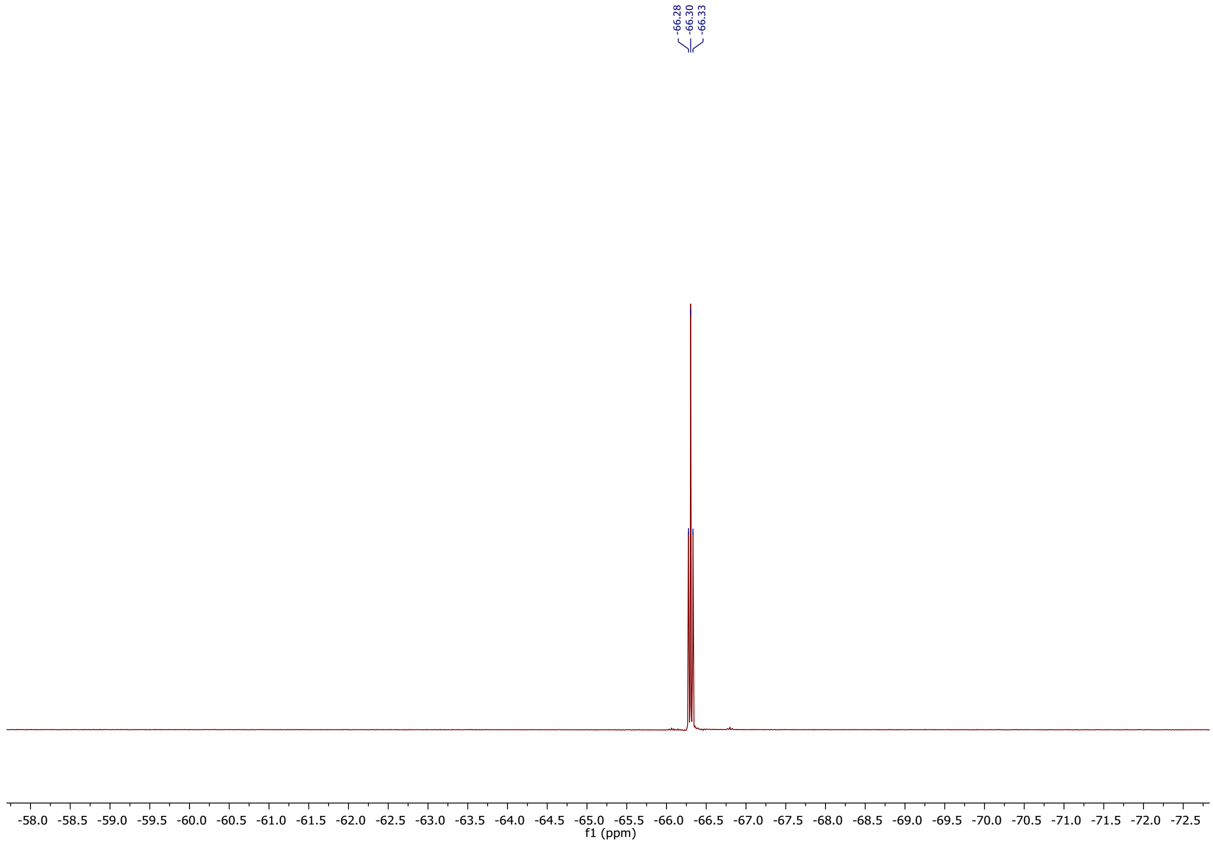
-66.28
-66.30
-66.33
-58.0 -58.5 -59.0 -59.5 -60.0 -60.5 -61.0 -61.5 -62.0 -62.5 -63.0 -63.5 -64.0 -64.5 -65.0 -65.5 -66.0 -66.5 -67.0 -67.5 -68.0 -68.5 -69.0 -69.5 -70.0 -70.5 -71.0 -71.5 -72.0 -72.5
f1 (ppm)

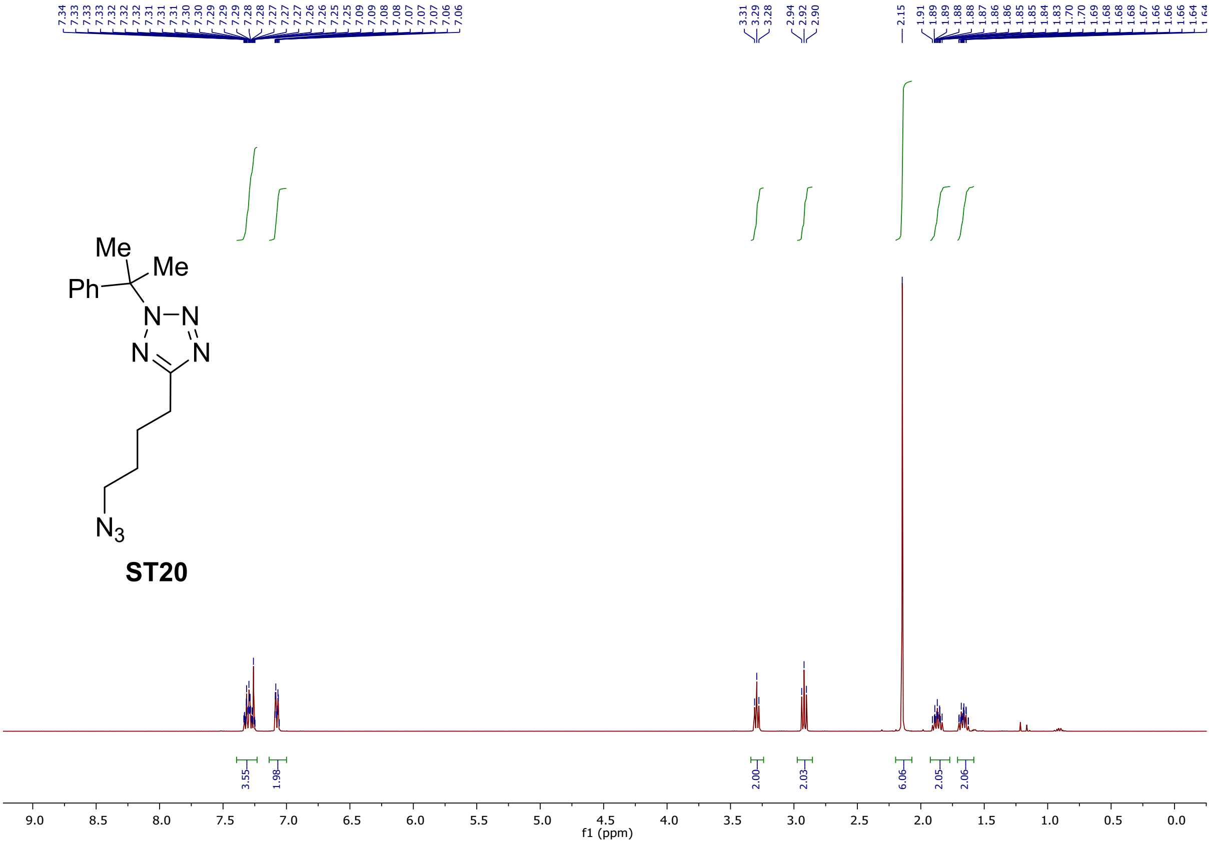
Me
Me
Ph
N
N
N
N
N3
ST20
f1 (ppm)

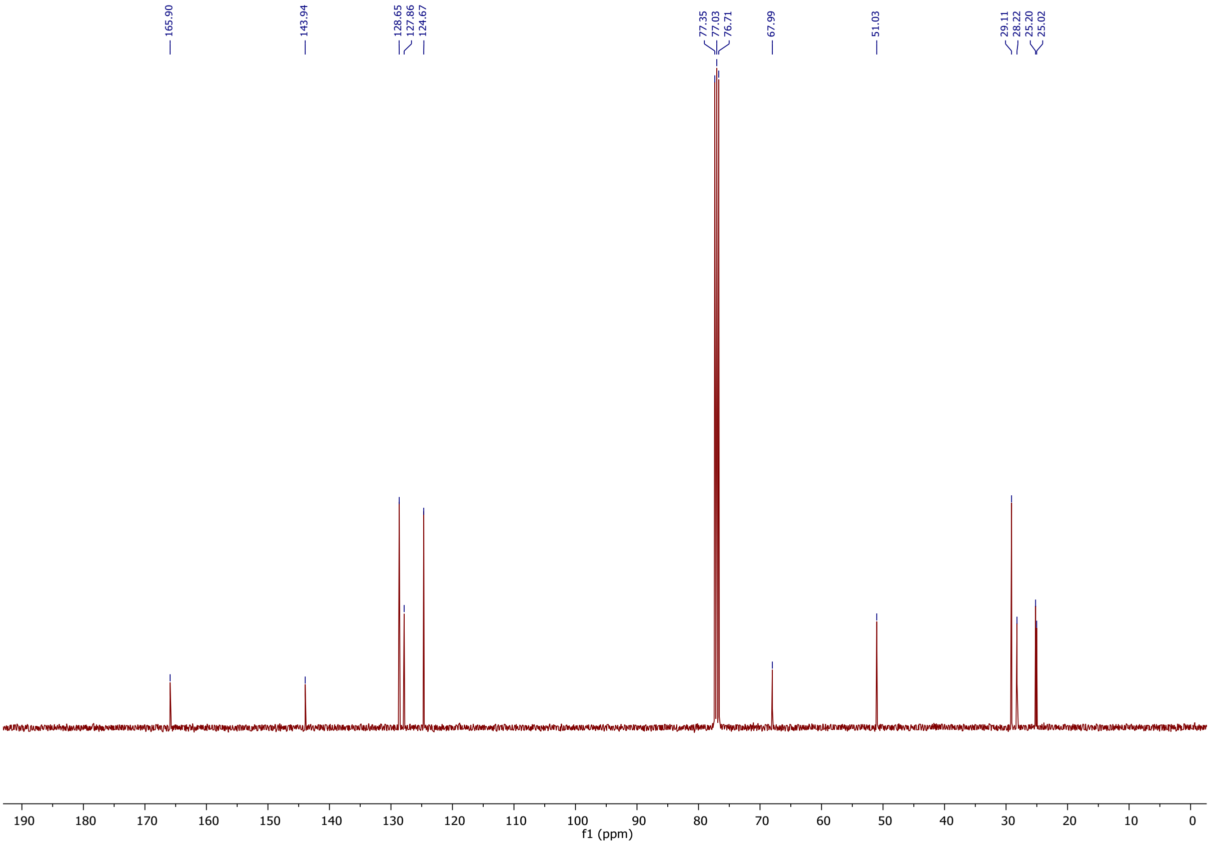
165.90
143.94
128.65
127.86
124.67
77.35
77.03
76.71
67.99
51.03
29.11
28.22
25.20
25.02
f1 (ppm)

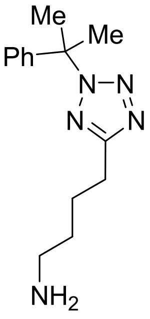
Me
Me
Ph
N
N
N
N
NH2

ST21

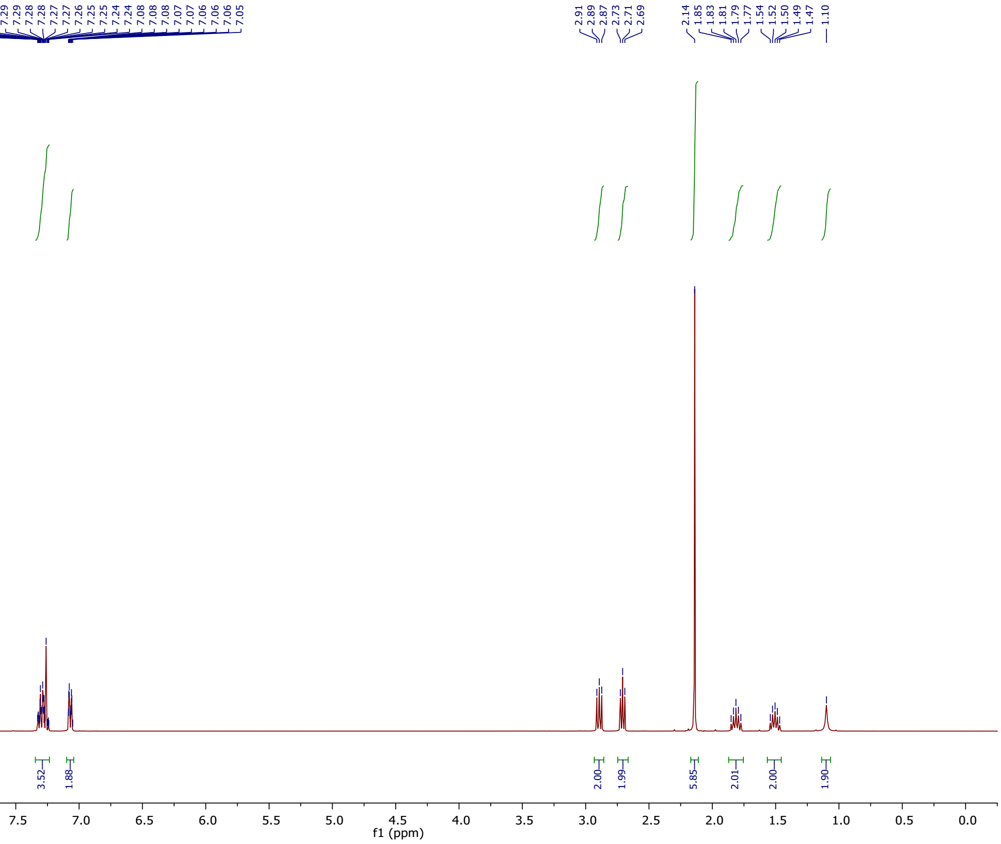
7.33 7.32 7.32 7.32 7.31 7.31 7.31 7.30 7.30 7.29 7.29 7.29 7.28 7.28 7.27 7.27 7.26 7.25 7.25 7.24 7.24 7.08 7.08 7.08 7.07 7.07 7.06 7.06 7.06 7.05
2.91 2.89 2.87 2.73 2.71 2.69
2.14 1.85 1.83 1.81 1.79 1.77 1.54 1.52 1.50 1.49 1.47 1.10
3.52 1.88 2.00 1.99 5.85 2.01 2.00 1.90
9.0 8.5 8.0 7.5 7.0 6.5 6.0 5.5 5.0 4.5 4.0 3.5 3.0 2.5 2.0 1.5 1.0 0.5 0.0
f1 (ppm)

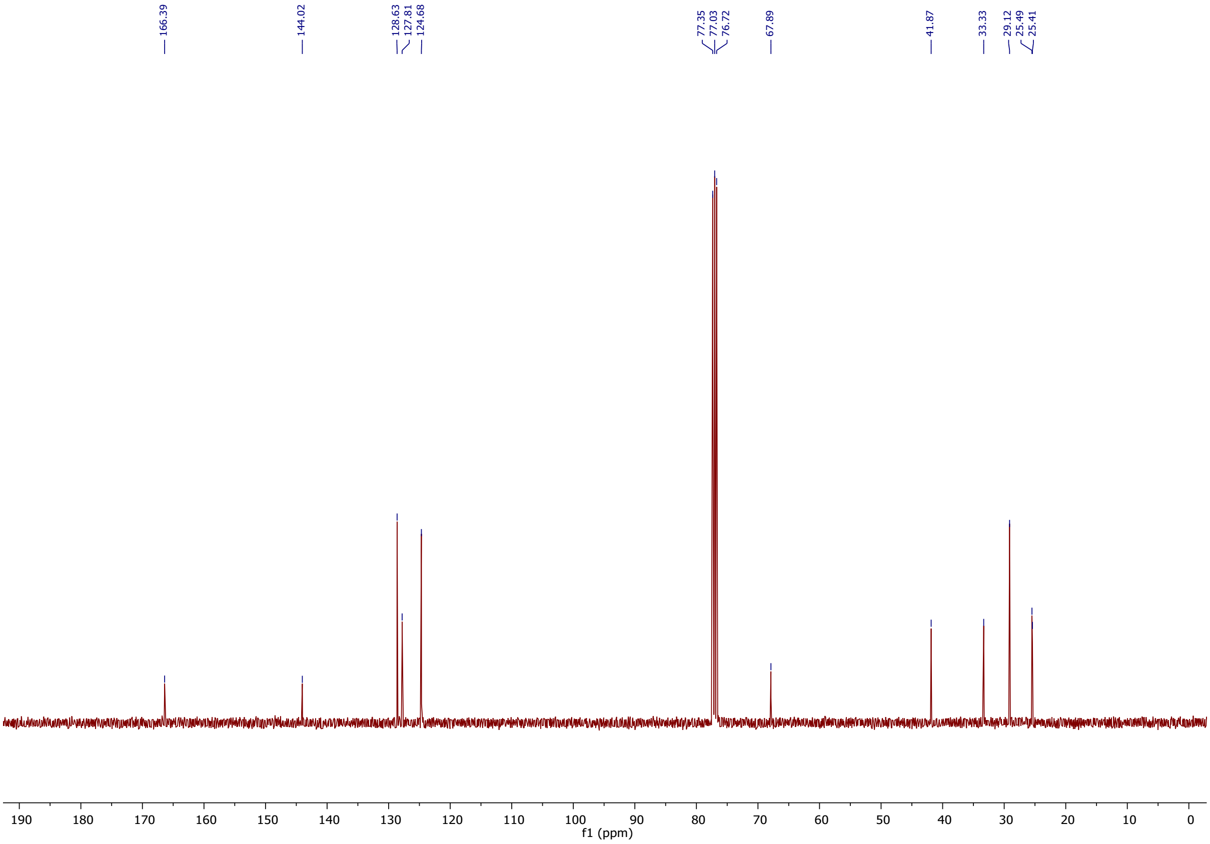
166.39
144.02
128.63 127.81 124.68
77.35 77.03 76.72
67.89
41.87
33.33
29.12 25.49 25.41
190 180 170 160 150 140 130 120 110 100 90 80 70 60 50 40 30 20 10 0
f1 (ppm)

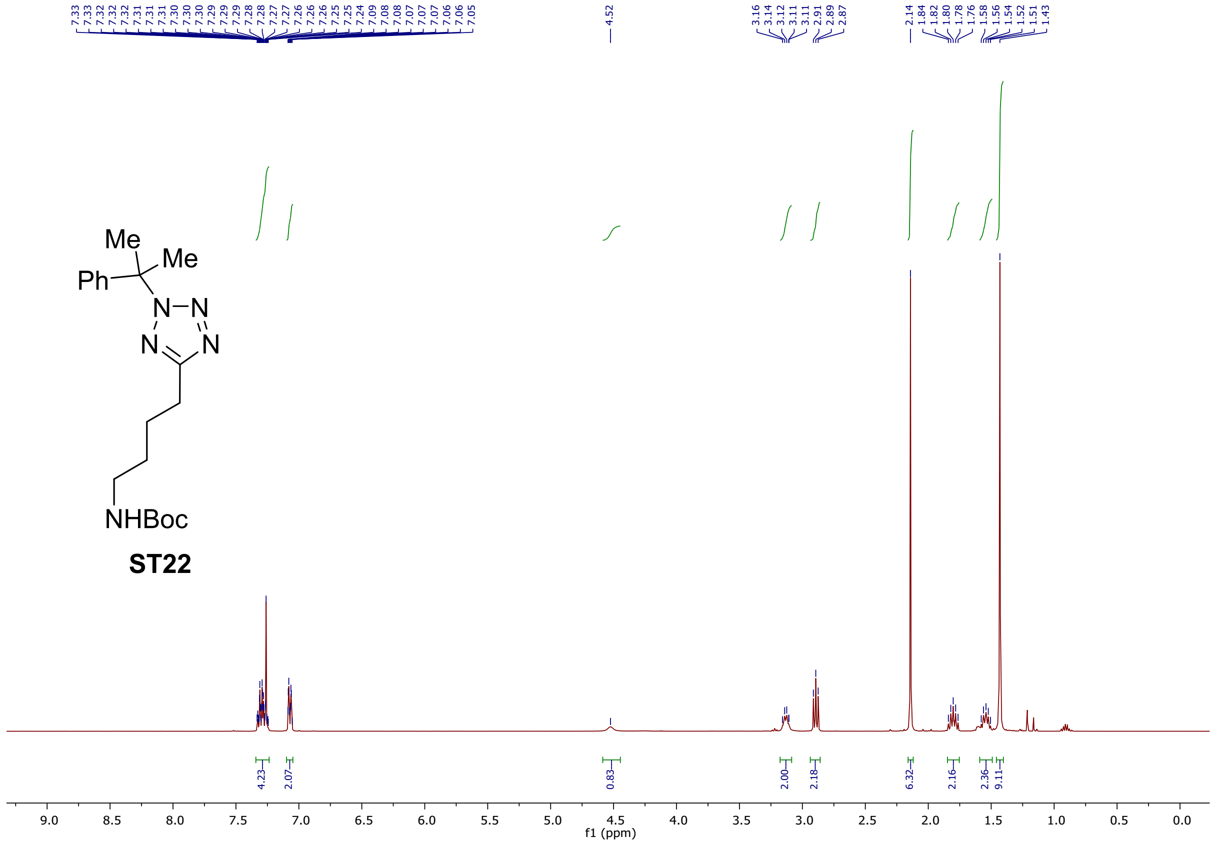
7.33 7.33 7.32 7.32 7.32 7.31 7.31 7.31 7.30 7.30 7.29 7.29 7.29 7.28 7.28 7.27 7.27 7.26 7.26 7.26 7.25 7.25 7.24 7.09 7.08 7.08 7.07 7.07 7.07 7.06 7.06 7.05
4.52
3.16 3.14 3.12 3.11 3.11 2.91 2.89 2.87
2.14 1.84 1.82 1.80 1.78 1.76 1.58 1.56 1.54 1.52 1.51 1.43
Me
Me
Ph
N
N
N
N
NHBoc
ST22
4.23
2.07
0.83
2.00
2.18
6.32
2.16
2.36
9.11
9.0 8.5 8.0 7.5 7.0 6.5 6.0 5.5 5.0 4.5 4.0 3.5 3.0 2.5 2.0 1.5 1.0 0.5 0.0
f1 (ppm)

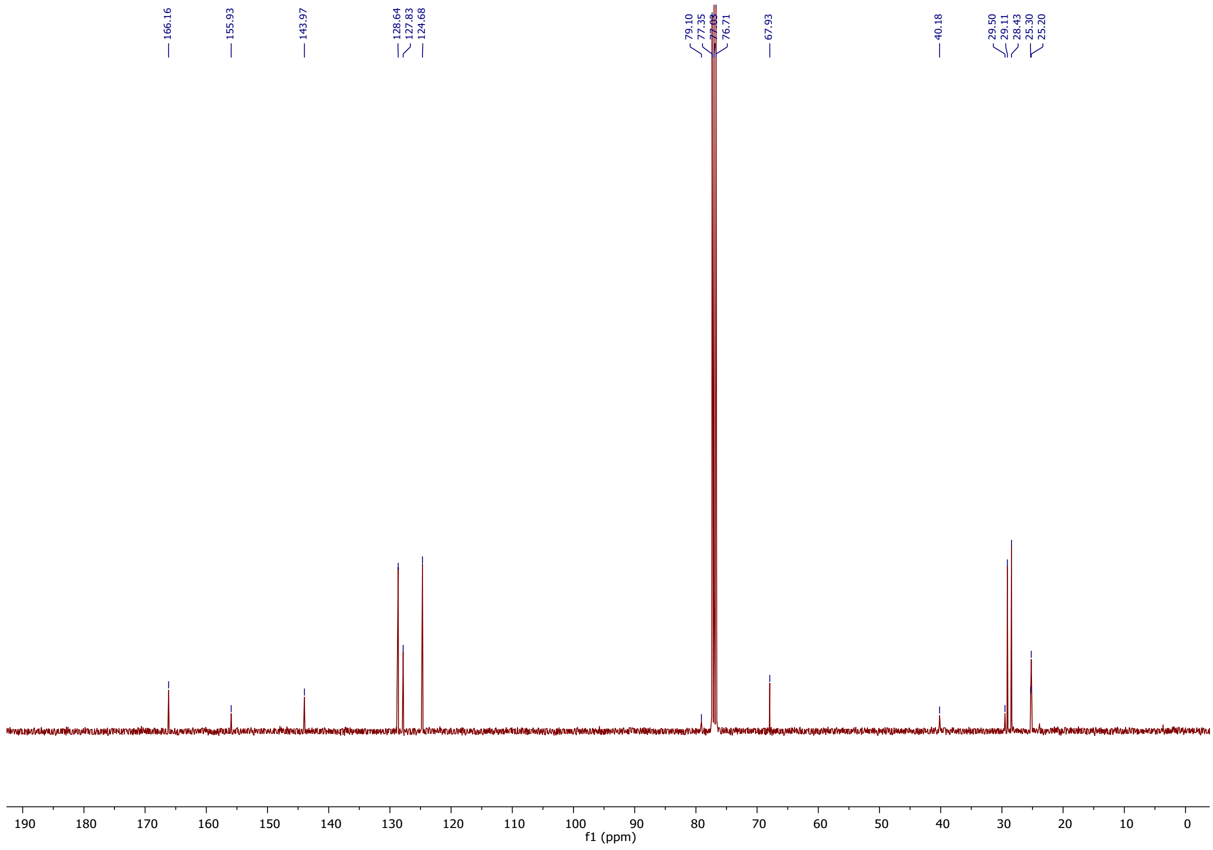
166.16
155.93
143.97
128.64 127.83 124.68
79.10 77.35 77.03 76.71
67.93
40.18
29.50 29.11 28.43 25.30 25.20
190 180 170 160 150 140 130 120 110 100 90 80 70 60 50 40 30 20 10 0
f1 (ppm)

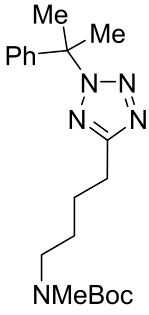
Me
Me
Ph
N–N
N
N
NMeBoc

33

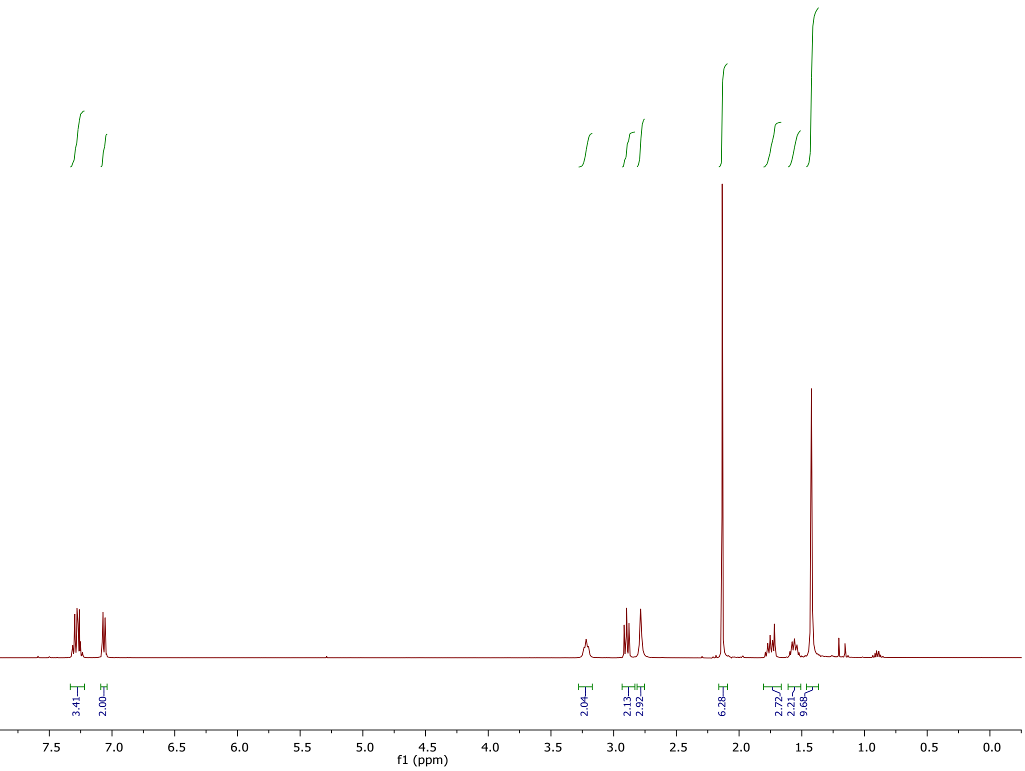
3.41
2.00
2.04
2.13
2.92
6.28
2.72
2.21
9.68
9.0 8.5 8.0 7.5 7.0 6.5 6.0 5.5 5.0 4.5 4.0 3.5 3.0 2.5 2.0 1.5 1.0 0.5 0.0
f1 (ppm)

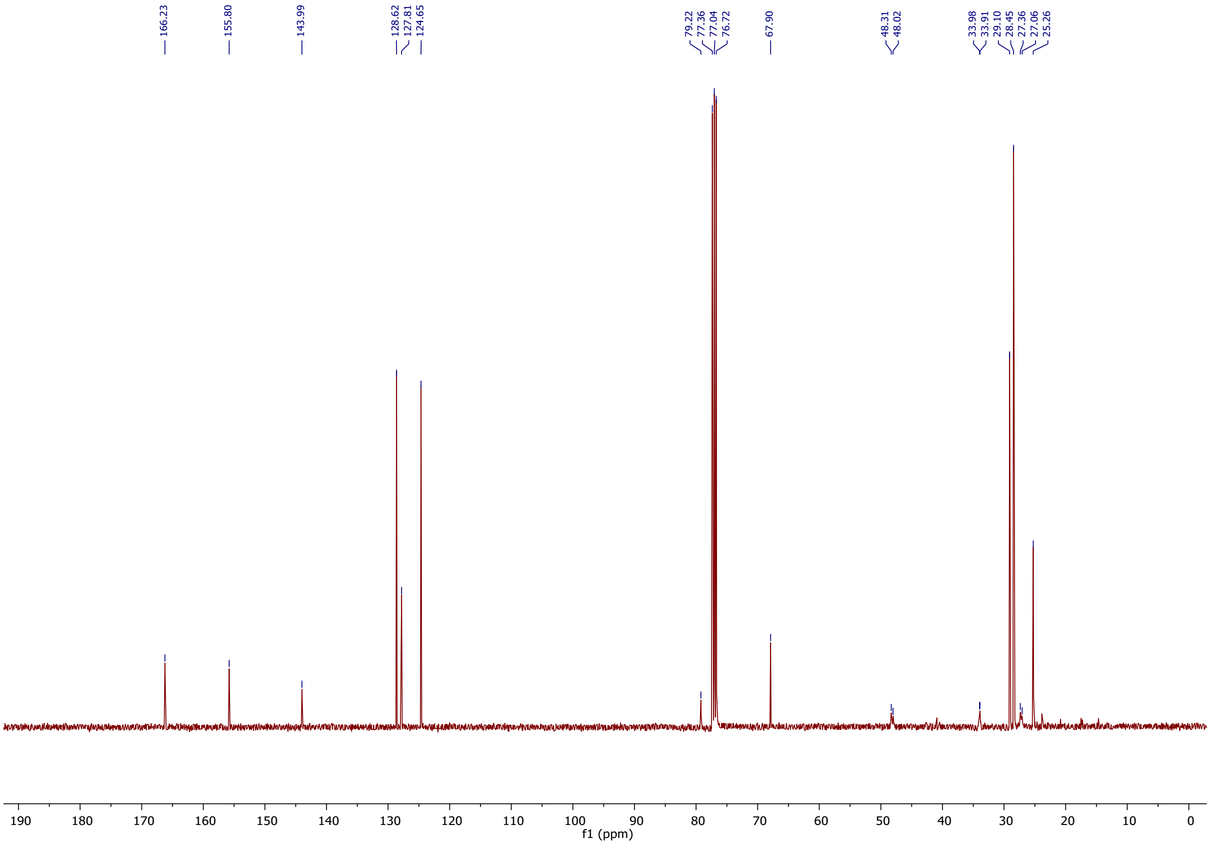
166.23
155.80
143.99
128.62
127.81
124.65
79.22
77.36
77.04
76.72
67.90
48.31
48.02
33.98
33.91
29.10
28.45
27.36
27.06
25.26
190 180 170 160 150 140 130 120 110 100 90 80 70 60 50 40 30 20 10 0
f1 (ppm)

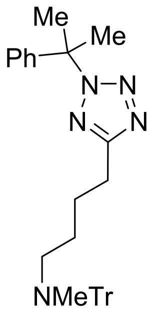
Me
Me
Ph
N
N
N
N
NMeTr

ST24

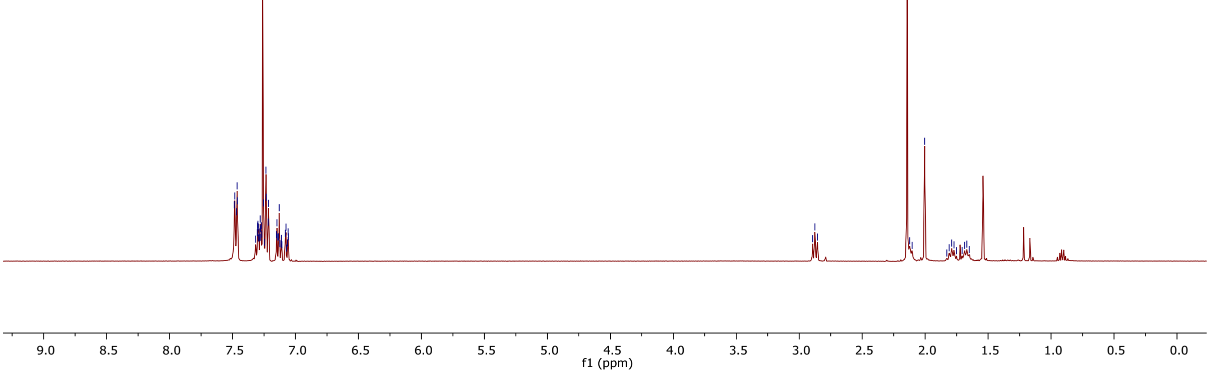
7.49
7.48
7.47
7.46
7.32
7.30
7.30
7.30
7.30
7.29
7.29
7.28
7.28
7.28
7.28
7.27
7.27
7.26
7.25
7.24
7.23
7.23
7.23
7.22
7.21
7.15
7.15
7.14
7.13
7.13
7.12
7.11
7.11
7.11
7.08
7.08
7.07
7.06
7.06
7.06
2.89
2.88
2.86
2.14
2.12
2.10
2.00
1.83
1.81
1.79
1.77
1.75
1.71
1.69
1.67
1.65
f1 (ppm)

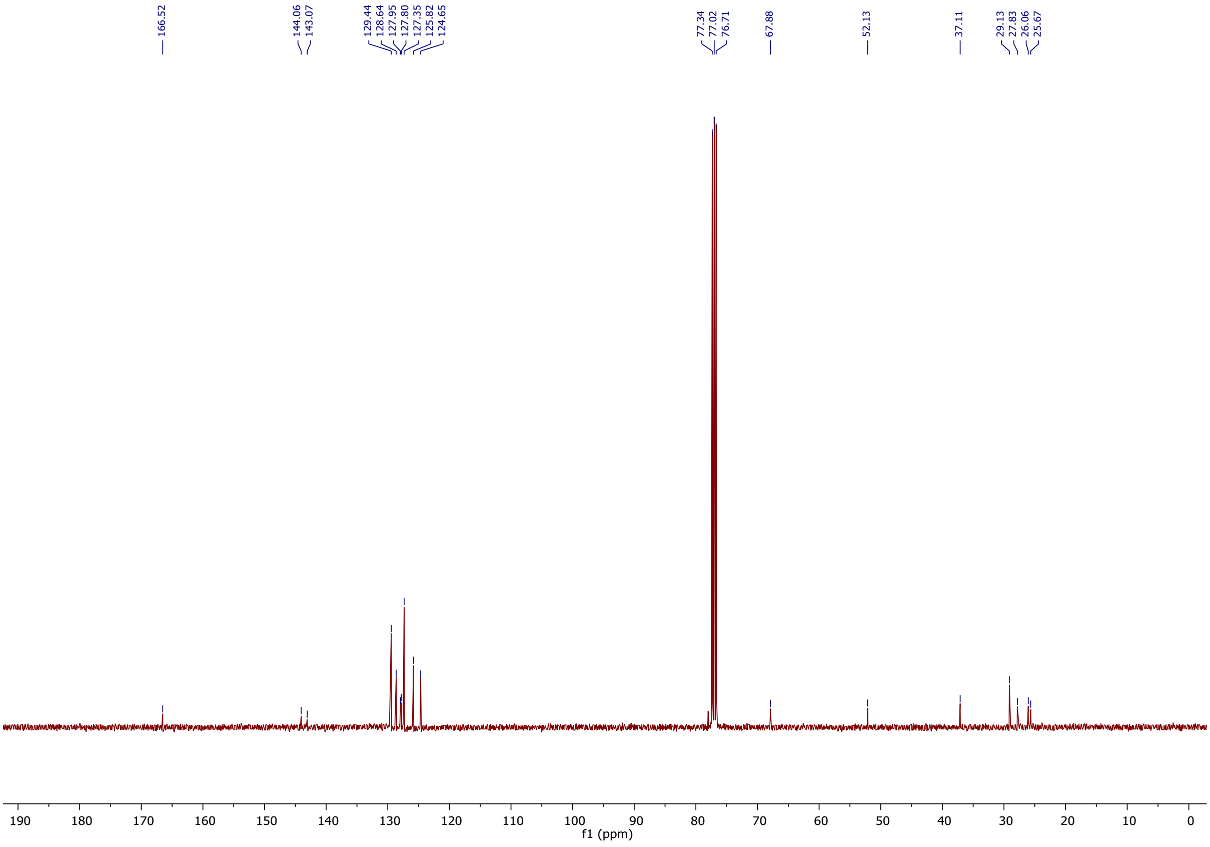
166.52
144.06
143.07
129.44
128.64
127.95
127.80
127.35
125.82
124.65
77.34
77.02
76.71
67.88
52.13
37.11
29.13
27.83
26.06
25.67
f1 (ppm)

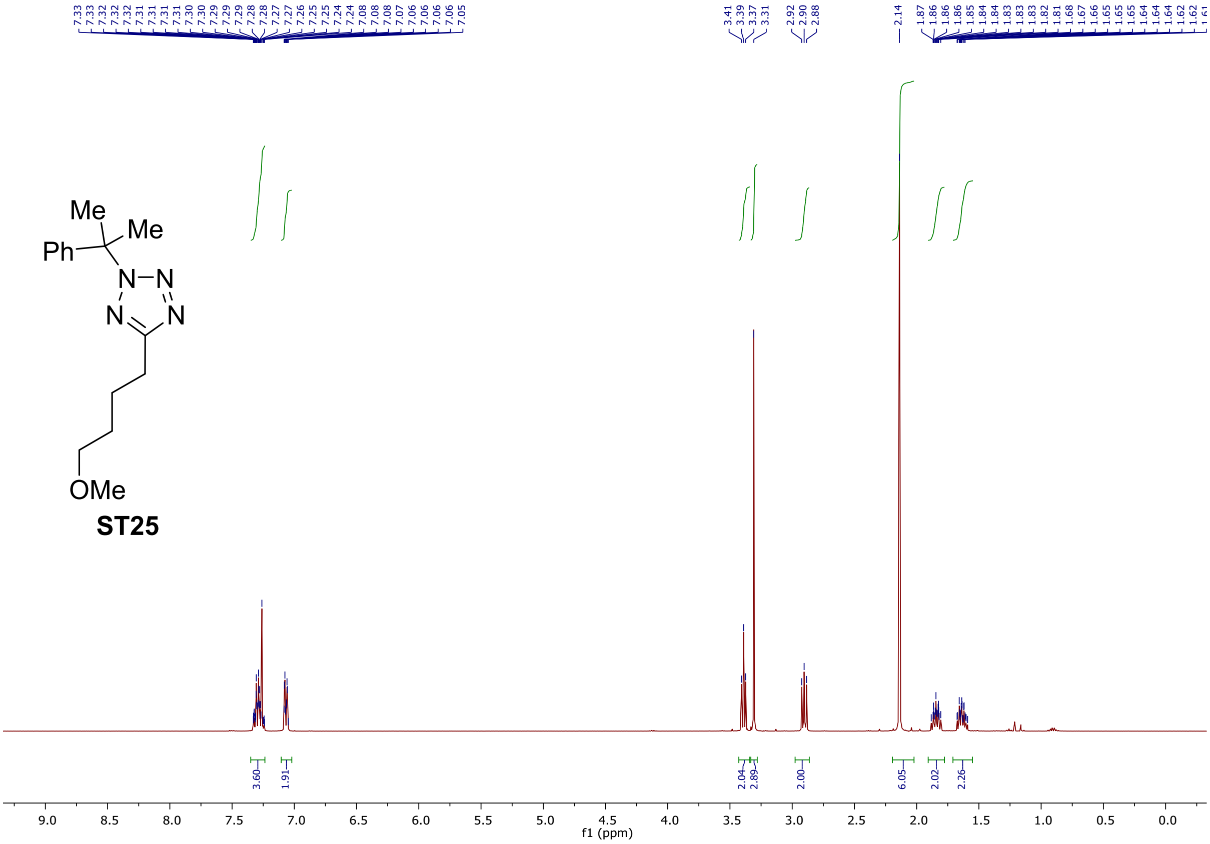
Me
Me
Ph
N
N
N
N
OMe
ST25
7.33
7.33
7.32
7.32
7.31
7.31
7.31
7.30
7.29
7.29
7.29
7.28
7.28
7.27
7.26
7.25
7.25
7.24
7.24
7.08
7.08
7.08
7.07
7.06
7.06
7.06
7.05
3.41
3.39
3.37
3.31
2.92
2.90
2.88
2.14
3.60
1.91
2.04
2.89
2.00
6.05
2.02
2.26
f1 (ppm)

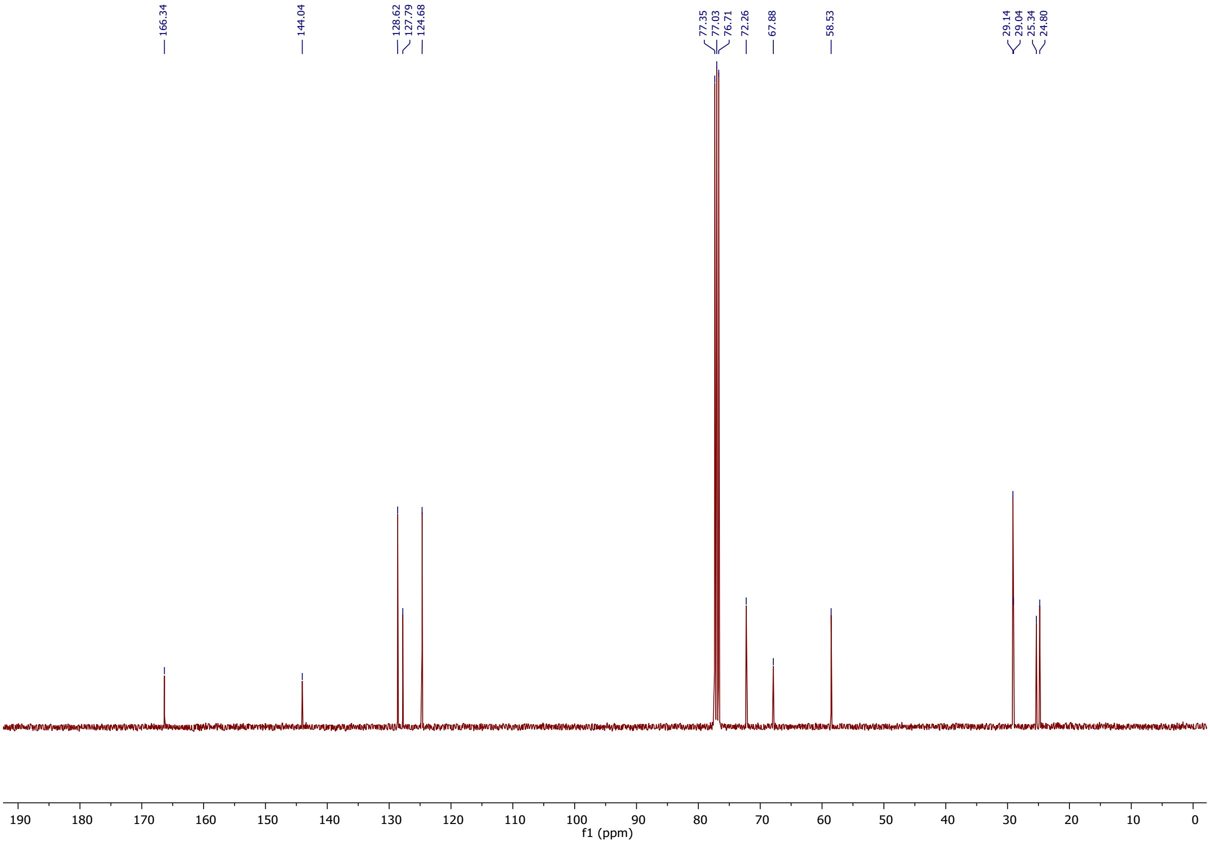
166.34
144.04
128.62
127.79
124.68
77.35
77.03
76.71
72.26
67.88
58.53
29.14
29.04
25.34
24.80
f1 (ppm)

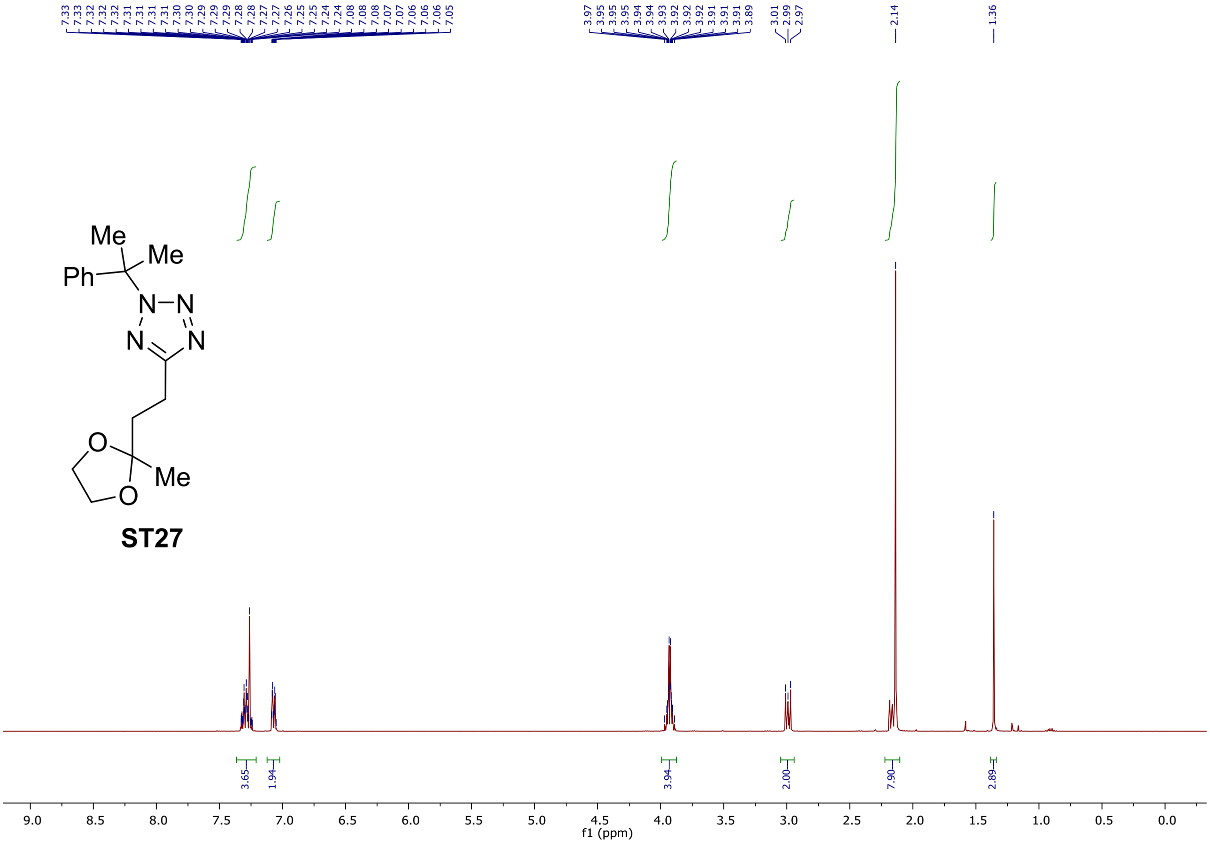
7.33 7.33 7.32 7.32 7.31 7.31 7.31 7.30 7.30 7.29 7.29 7.29 7.28 7.28 7.27 7.27 7.26 7.25 7.25 7.24 7.24 7.08 7.08 7.08 7.07 7.07 7.06 7.06 7.06 7.05
3.97 3.95 3.95 3.95 3.94 3.94 3.93 3.92 3.92 3.92 3.91 3.91 3.91 3.89
3.01 2.99 2.97
2.14
1.36
3.65
1.94
3.94
2.00
7.90
2.89
9.0 8.5 8.0 7.5 7.0 6.5 6.0 5.5 5.0 4.5 4.0 3.5 3.0 2.5 2.0 1.5 1.0 0.5 0.0
f1 (ppm)

ST27

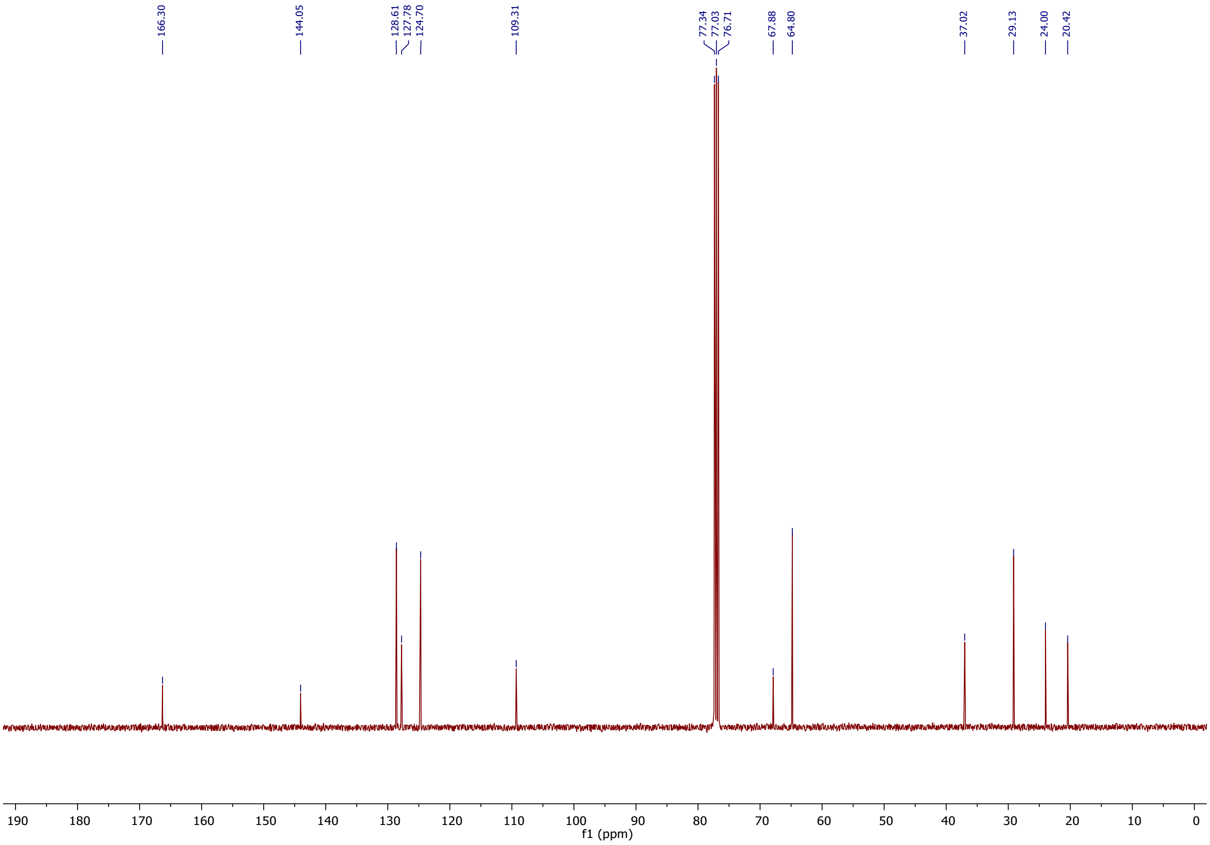
166.30
144.05
128.61 127.78 124.70
109.31
77.34 77.03 76.71
67.88
64.80
37.02
29.13
24.00
20.42
190 180 170 160 150 140 130 120 110 100 90 80 70 60 50 40 30 20 10 0
f1 (ppm)

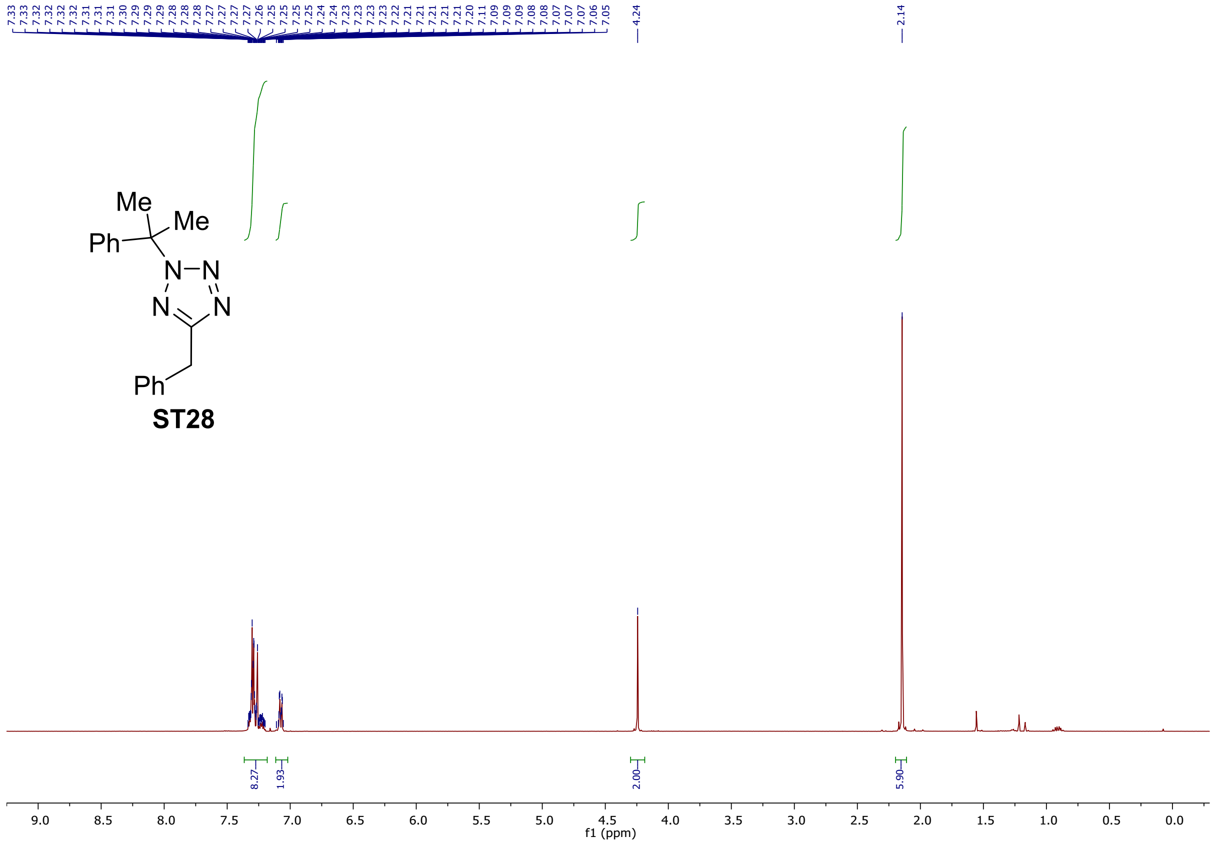

ST28

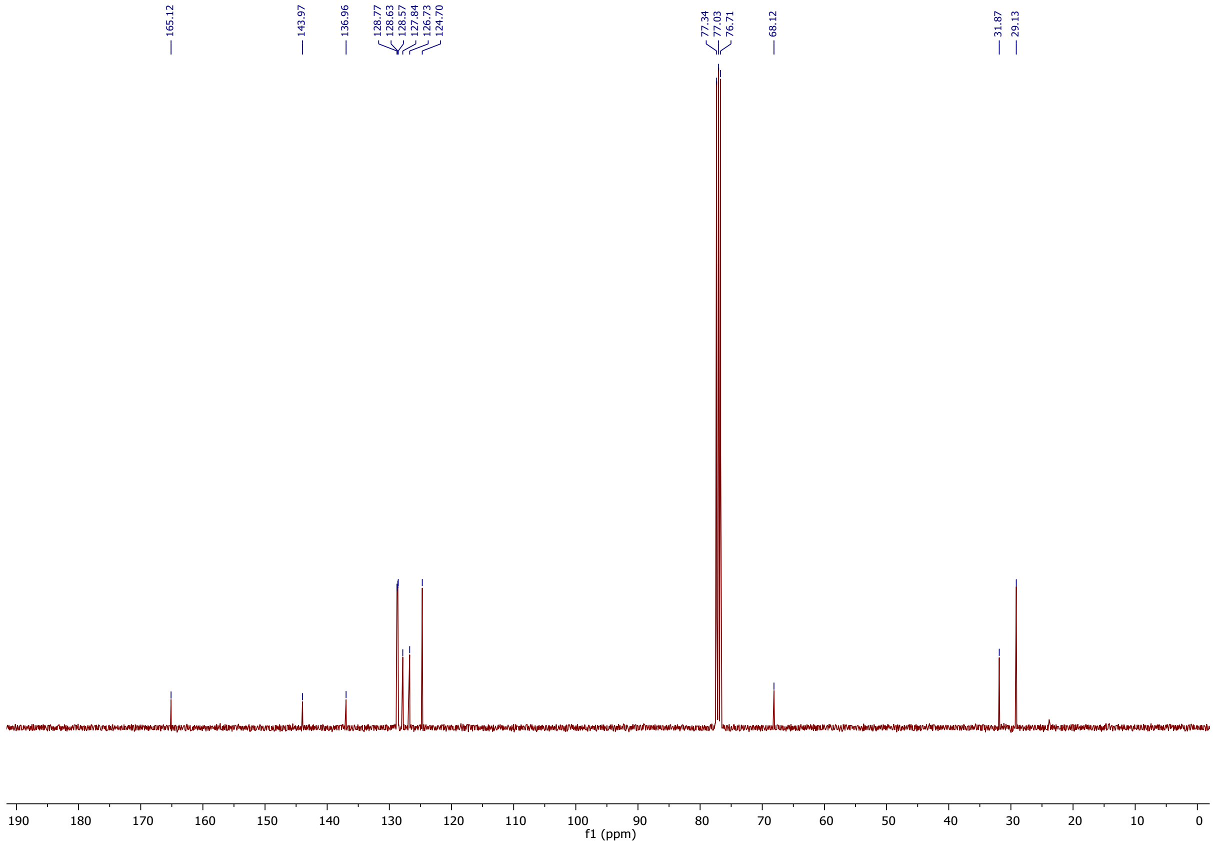

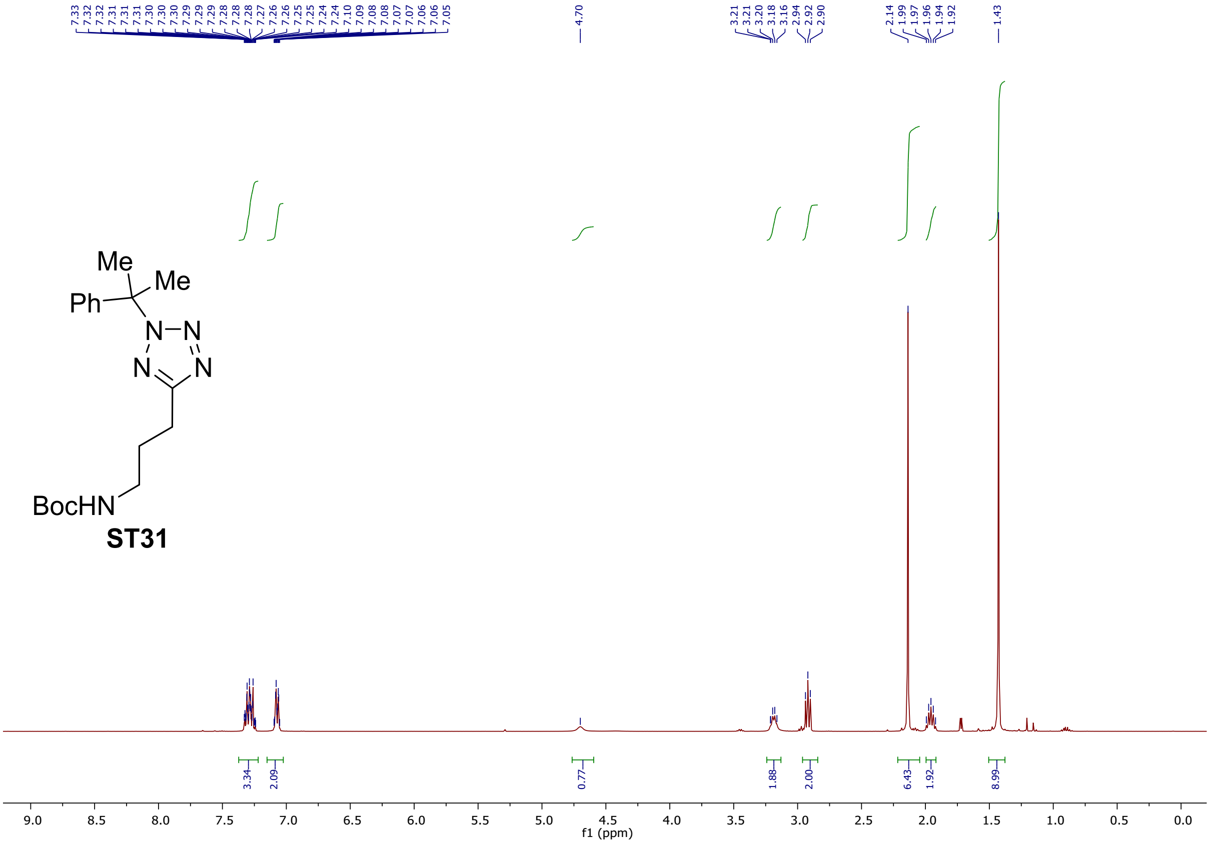
7.33
7.32
7.32
7.31
7.31
7.31
7.30
7.30
7.30
7.29
7.29
7.29
7.28
7.28
7.28
7.27
7.26
7.26
7.25
7.25
7.24
7.24
7.10
7.09
7.08
7.08
7.07
7.07
7.06
7.06
7.05
4.70
3.21
3.21
3.20
3.18
3.16
2.94
2.92
2.90
2.14
1.99
1.97
1.96
1.94
1.92
1.43
Me
Me
Ph
N
N
N
N
BocHN
ST31
3.34
2.09
0.77
1.88
2.00
6.43
1.92
8.99
f1 (ppm)

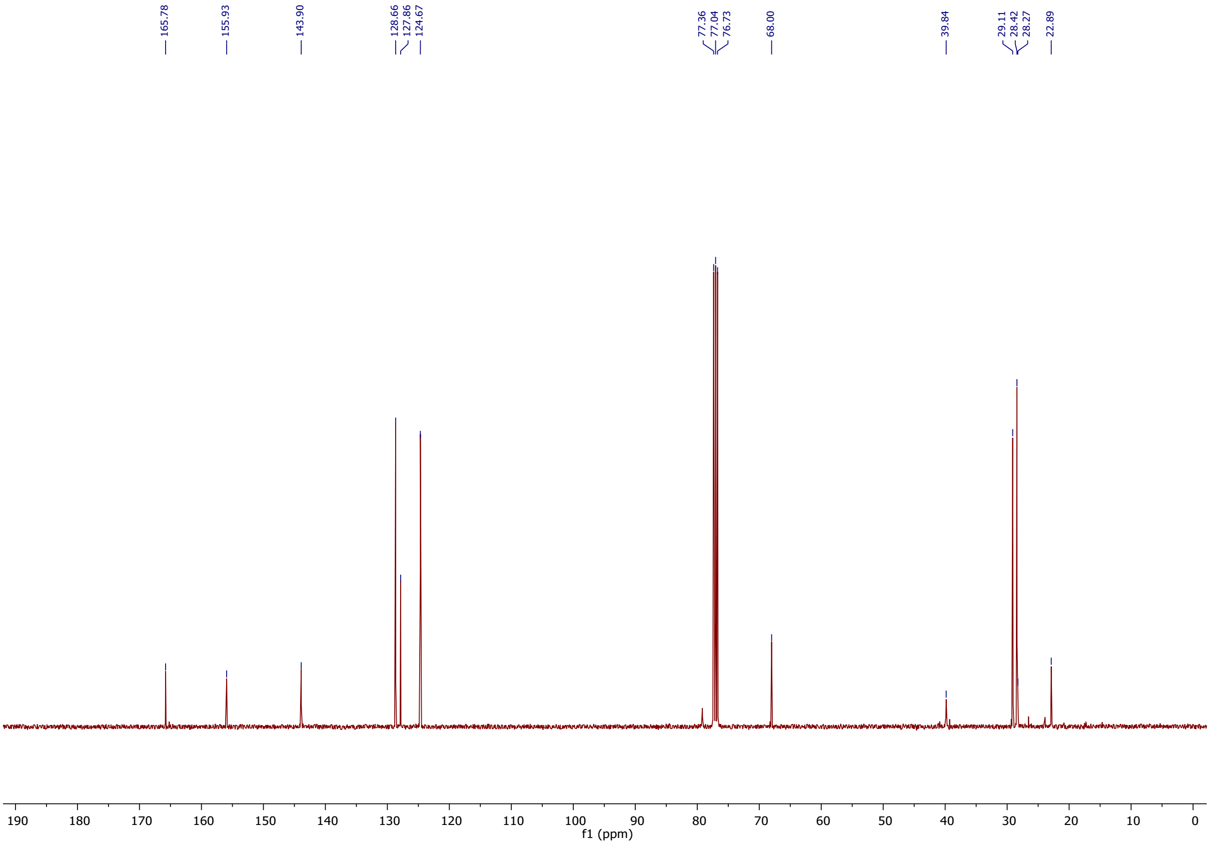
165.78
155.93
143.90
128.66
127.86
124.67
77.36
77.04
76.73
68.00
39.84
29.11
28.42
28.27
22.89
f1 (ppm)

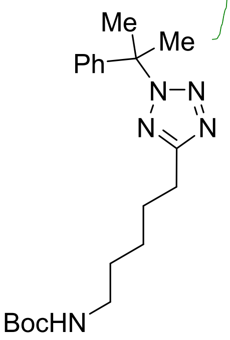
Me
Me
Ph
N
N
N
N
BocHN

ST32

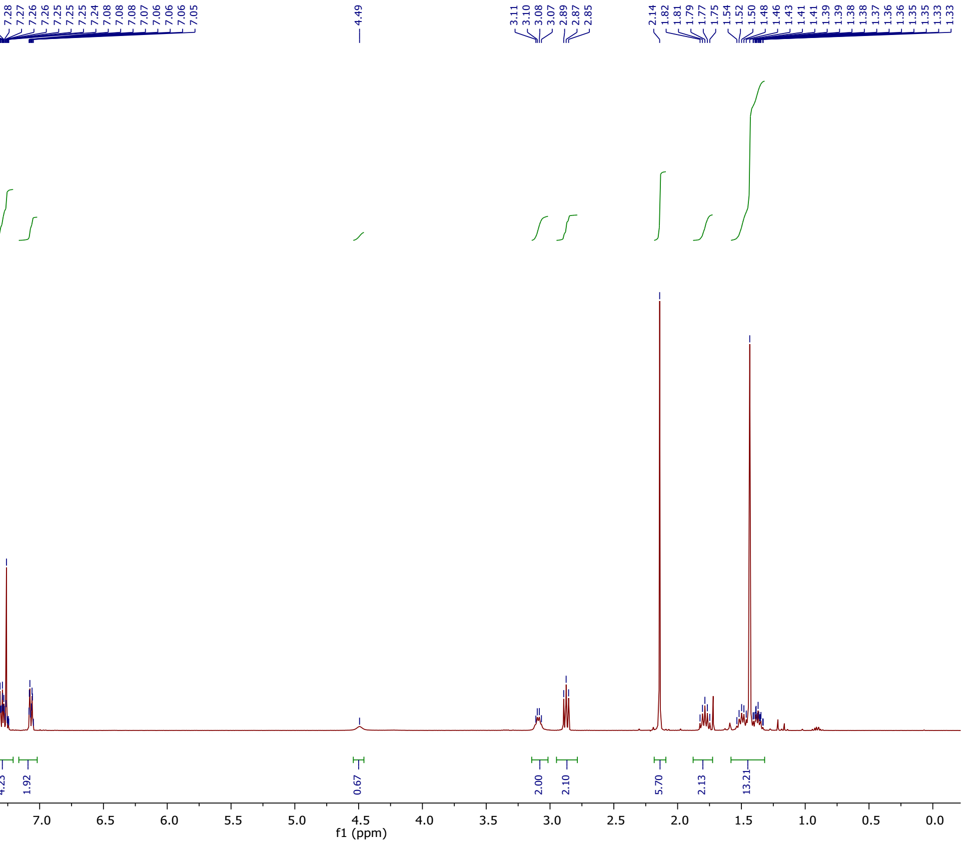
7.33 7.33 7.32 7.32 7.32 7.31 7.31 7.31 7.30 7.30 7.29 7.29 7.28 7.28 7.28 7.27 7.26 7.26 7.25 7.25 7.25 7.24 7.08 7.08 7.08 7.07 7.06 7.06 7.06 7.05
4.49
3.11 3.10 3.08 3.07 2.89 2.87 2.85
2.14 1.82 1.81 1.79 1.77 1.75 1.54 1.52 1.50 1.48 1.46 1.43 1.41 1.41 1.39 1.39 1.38 1.38 1.37 1.36 1.36 1.35 1.35 1.33 1.33
4.23
1.92
0.67
2.00
2.10
5.70
2.13
13.21
9.0 8.5 8.0 7.5 7.0 6.5 6.0 5.5 5.0 4.5 4.0 3.5 3.0 2.5 2.0 1.5 1.0 0.5 0.0
f1 (ppm)

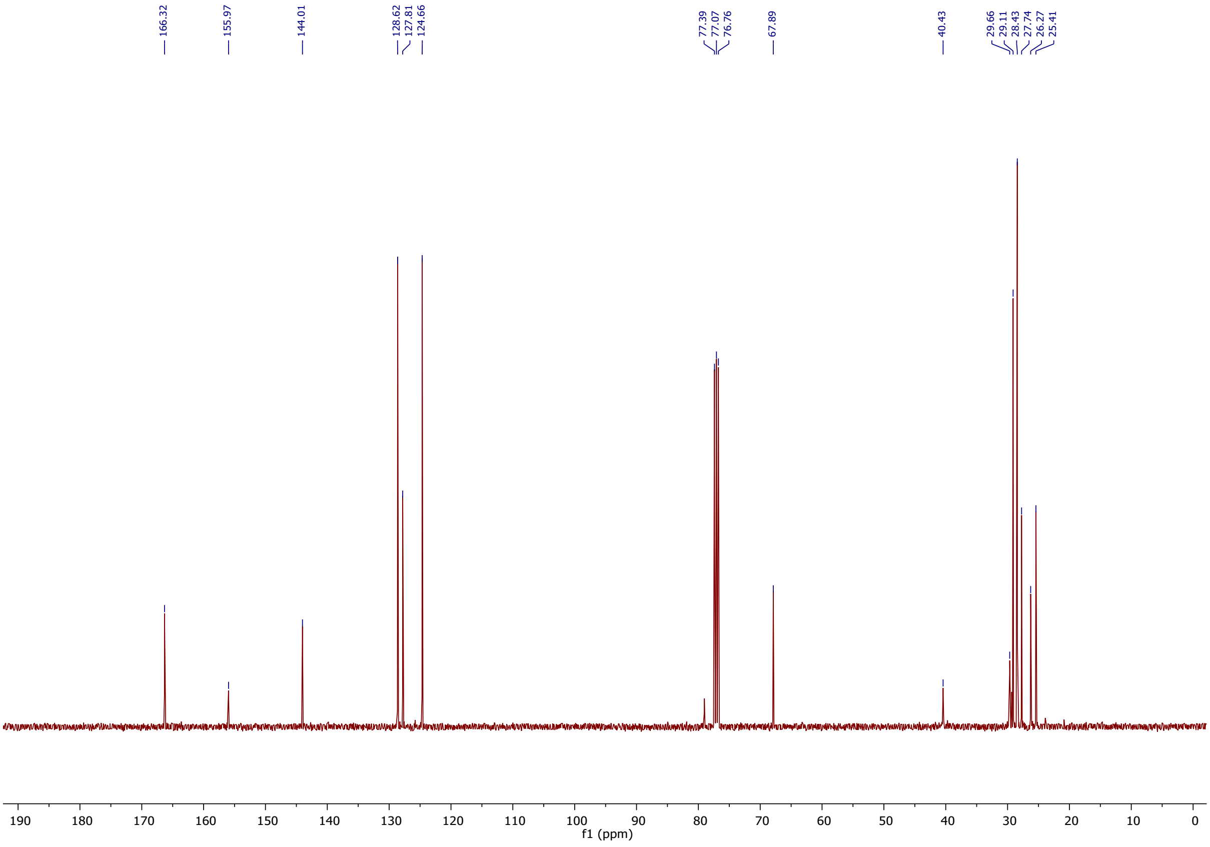
166.32
155.97
144.01
128.62 127.81 124.66
77.39 77.07 76.76
67.89
40.43
29.66 29.11 28.43 27.74 26.27 25.41
190 180 170 160 150 140 130 120 110 100 90 80 70 60 50 40 30 20 10 0
f1 (ppm)

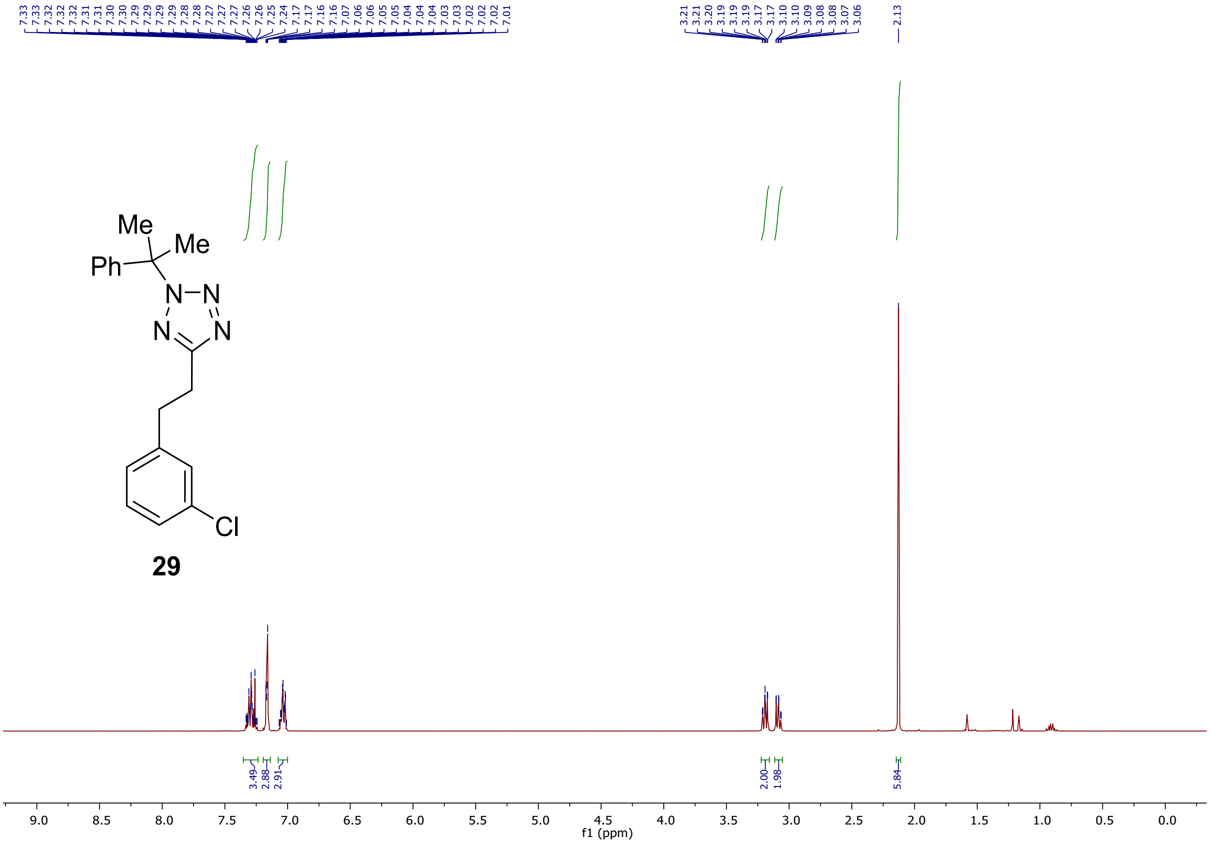
7.33
7.33
7.32
7.32
7.31
7.31
7.30
7.30
7.29
7.29
7.29
7.29
7.28
7.28
7.27
7.27
7.27
7.26
7.26
7.25
7.24
7.17
7.17
7.16
7.16
7.07
7.06
7.06
7.05
7.05
7.04
7.04
7.04
7.03
7.03
7.02
7.02
7.02
7.01
3.21
3.21
3.20
3.19
3.19
3.19
3.17
3.17
3.10
3.10
3.09
3.08
3.08
3.07
3.06
2.13
Me
Me
Ph
N
N
N
N
Cl
29
3.49
2.88
2.91
2.00
1.98
5.84
9.0
8.5
8.0
7.5
7.0
6.5
6.0
5.5
5.0
4.5
4.0
3.5
3.0
2.5
2.0
1.5
1.0
0.5
0.0
f1 (ppm)

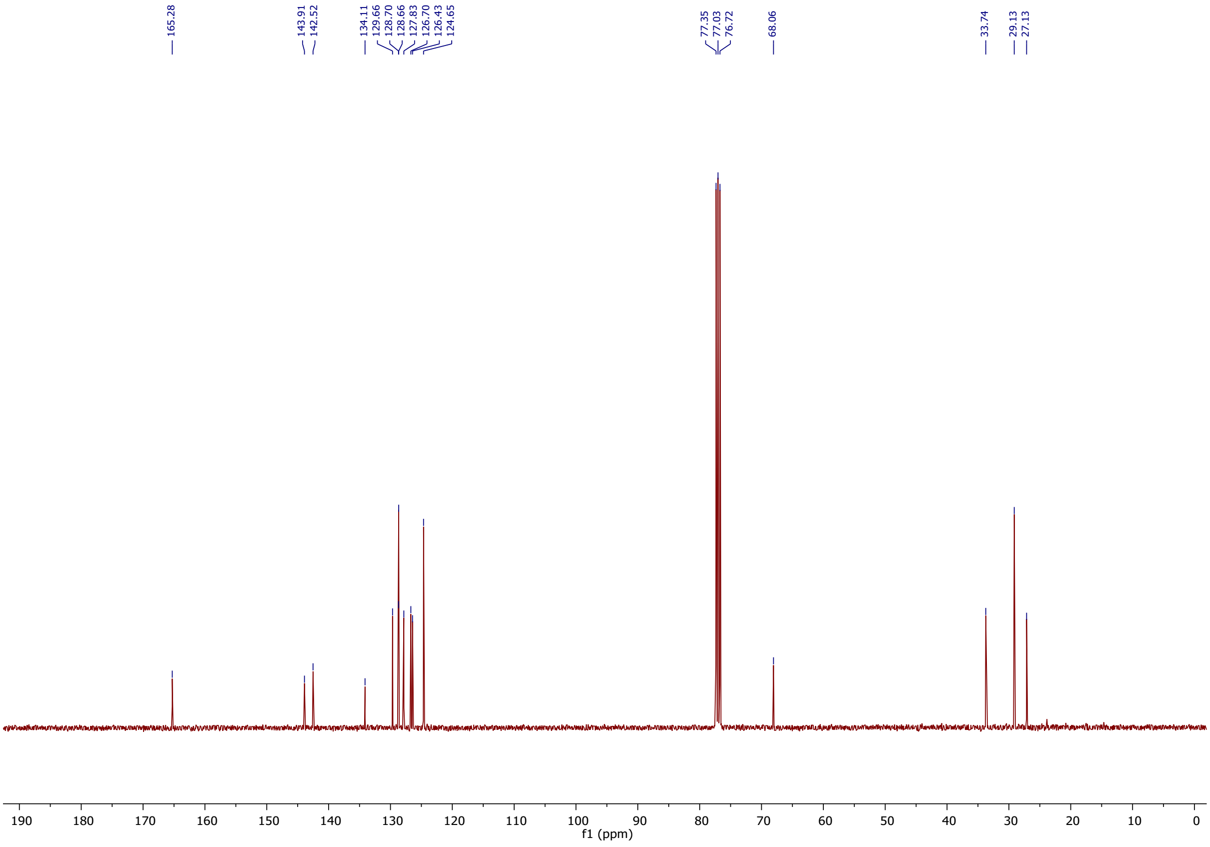
165.28
143.91
142.52
134.11
129.66
128.70
128.66
127.83
126.70
126.43
124.65
77.35
77.03
76.72
68.06
33.74
29.13
27.13
190
180
170
160
150
140
130
120
110
100
90
80
70
60
50
40
30
20
10
0
f1 (ppm)

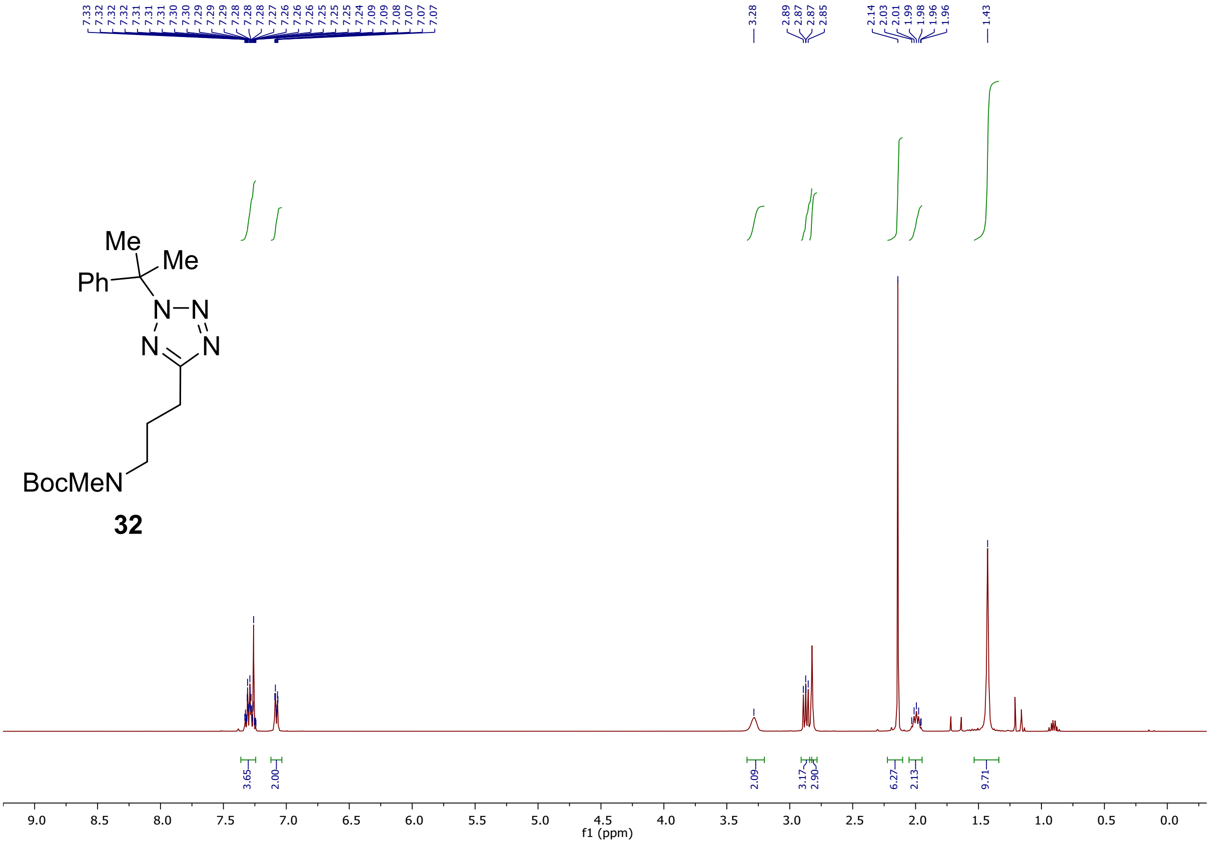
Me
Me
Ph
N
N
N
N
BocMeN
32
7.33 7.32 7.32 7.32 7.31 7.31 7.31 7.30 7.30 7.29 7.29 7.29 7.28 7.28 7.28 7.27 7.26 7.26 7.26 7.25 7.25 7.25 7.24 7.09 7.09 7.08 7.07 7.07 7.07
3.28
2.89 2.87 2.87 2.85
2.14 2.03 2.01 1.99 1.98 1.96 1.96
1.43
3.65
2.00
2.09
3.17
2.90
6.27
2.13
9.71
9.0 8.5 8.0 7.5 7.0 6.5 6.0 5.5 5.0 4.5 4.0 3.5 3.0 2.5 2.0 1.5 1.0 0.5 0.0
f1 (ppm)

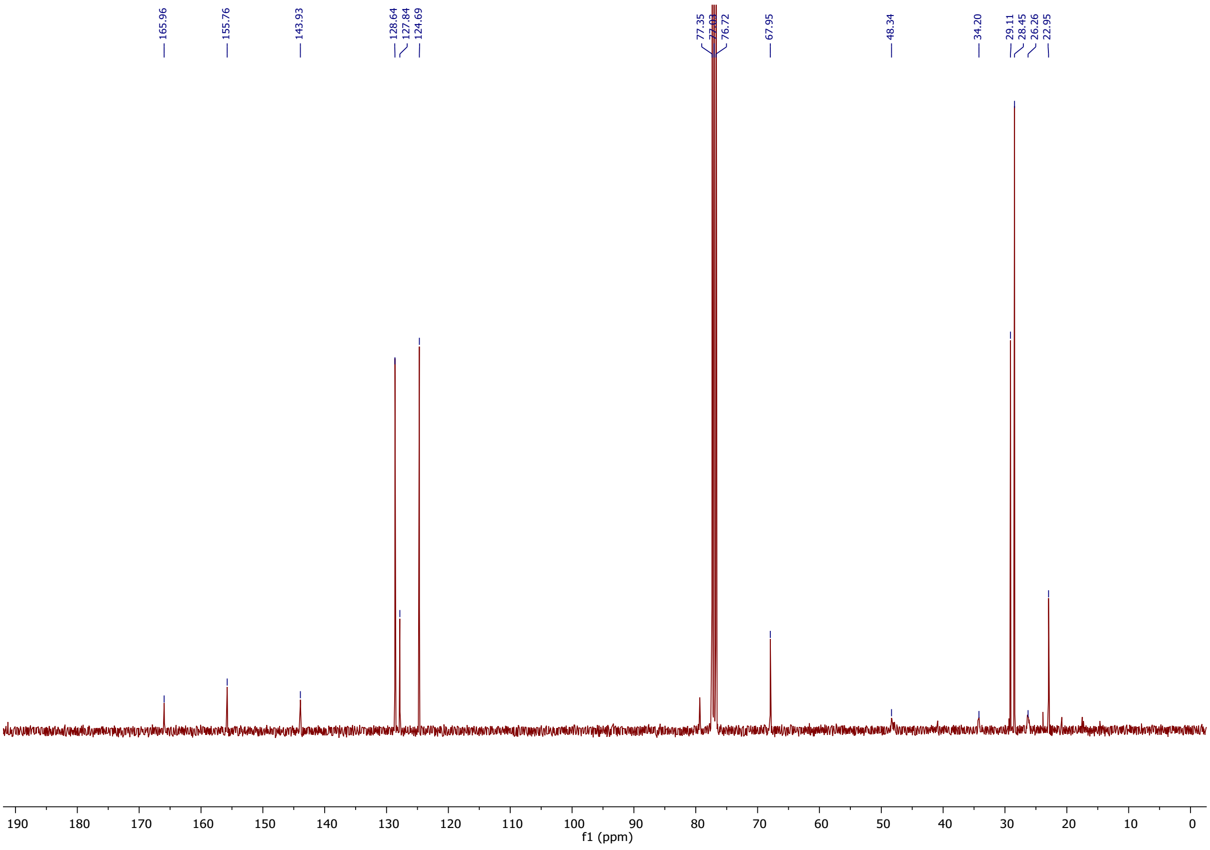
165.96
155.76
143.93
128.64 127.84 124.69
77.35 77.03 76.72
67.95
48.34
34.20
29.11 28.45 26.26 22.95
190 180 170 160 150 140 130 120 110 100 90 80 70 60 50 40 30 20 10 0
f1 (ppm)

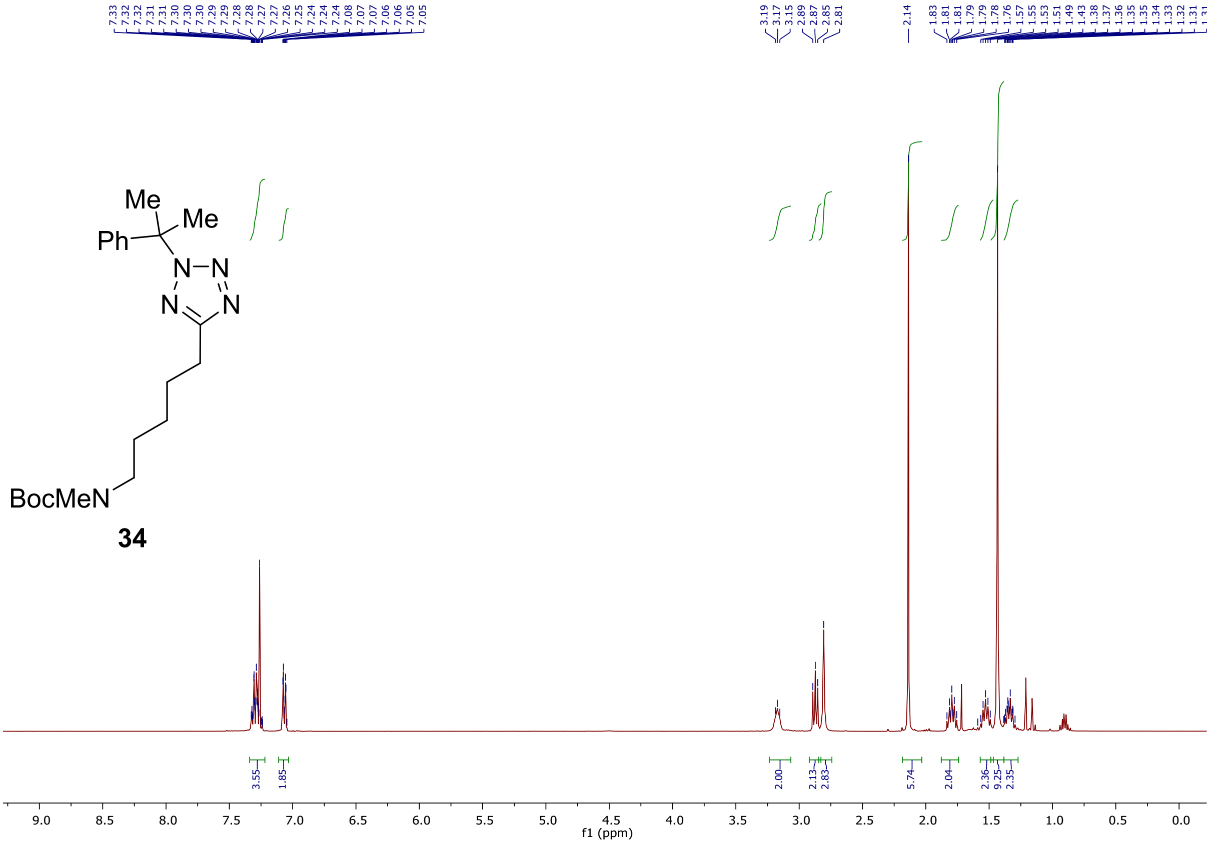

34

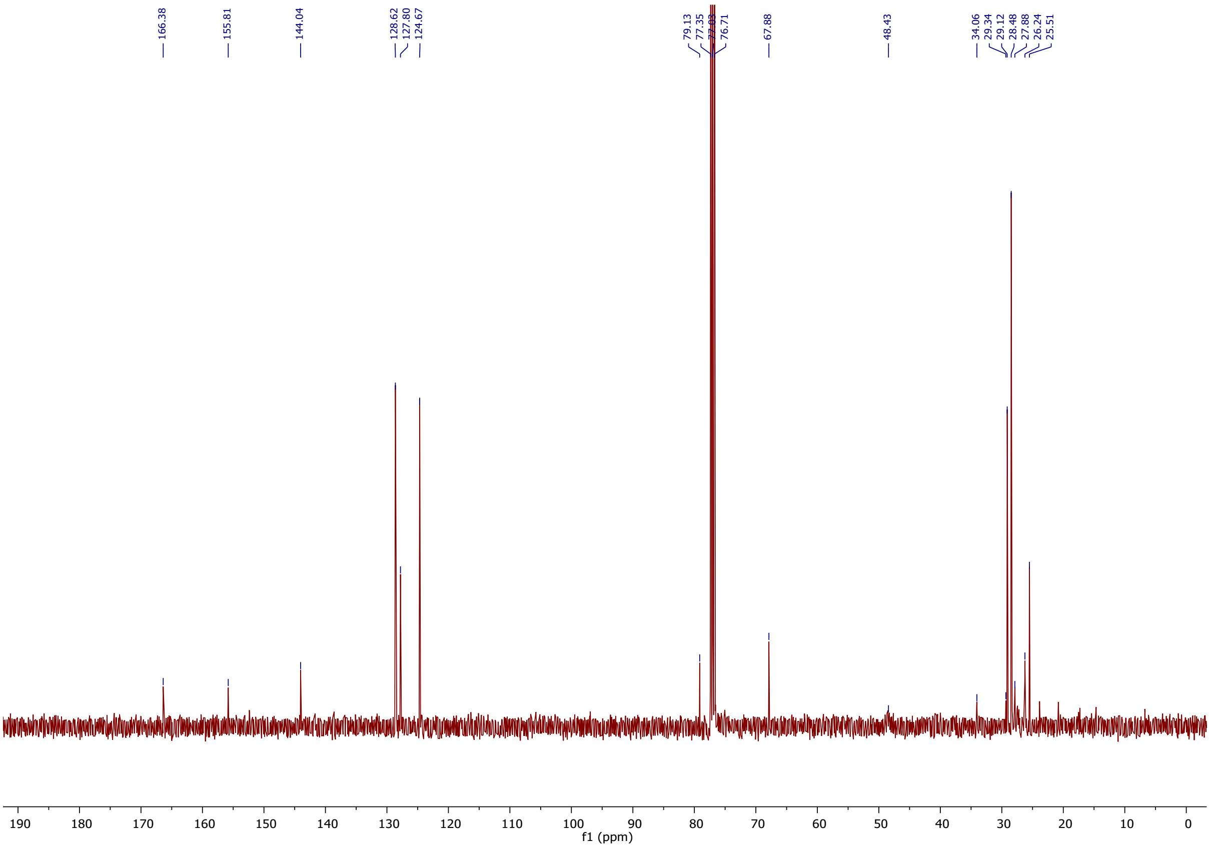

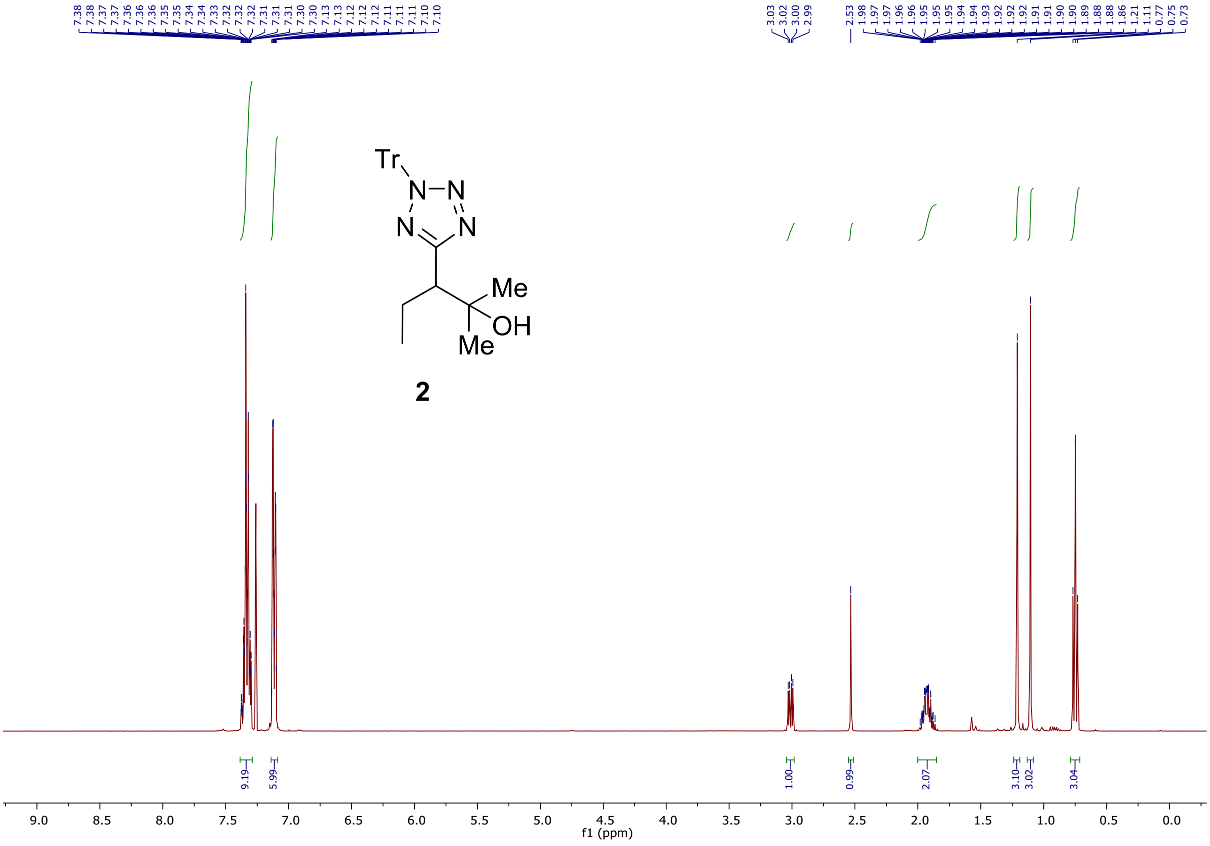
Tr
N
N
N
N
Me
OH
Me
2
9.19
5.99
1.00
0.99
2.07
3.10
3.02
3.04
f1 (ppm)

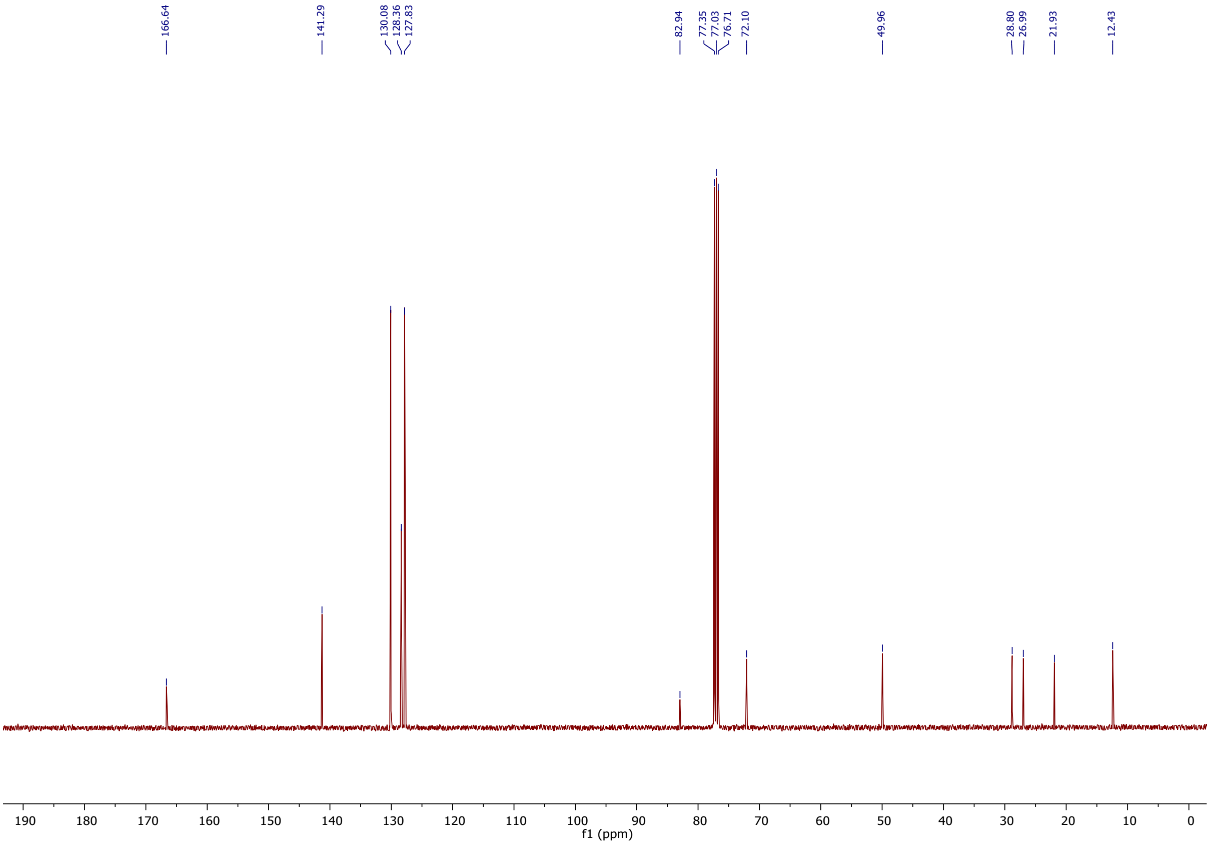
166.64
141.29
130.08
128.36
127.83
82.94
77.35
77.03
76.71
72.10
49.96
28.80
26.99
21.93
12.43
f1 (ppm)

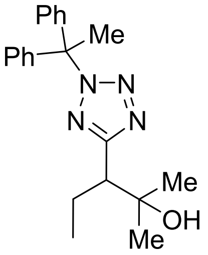

4

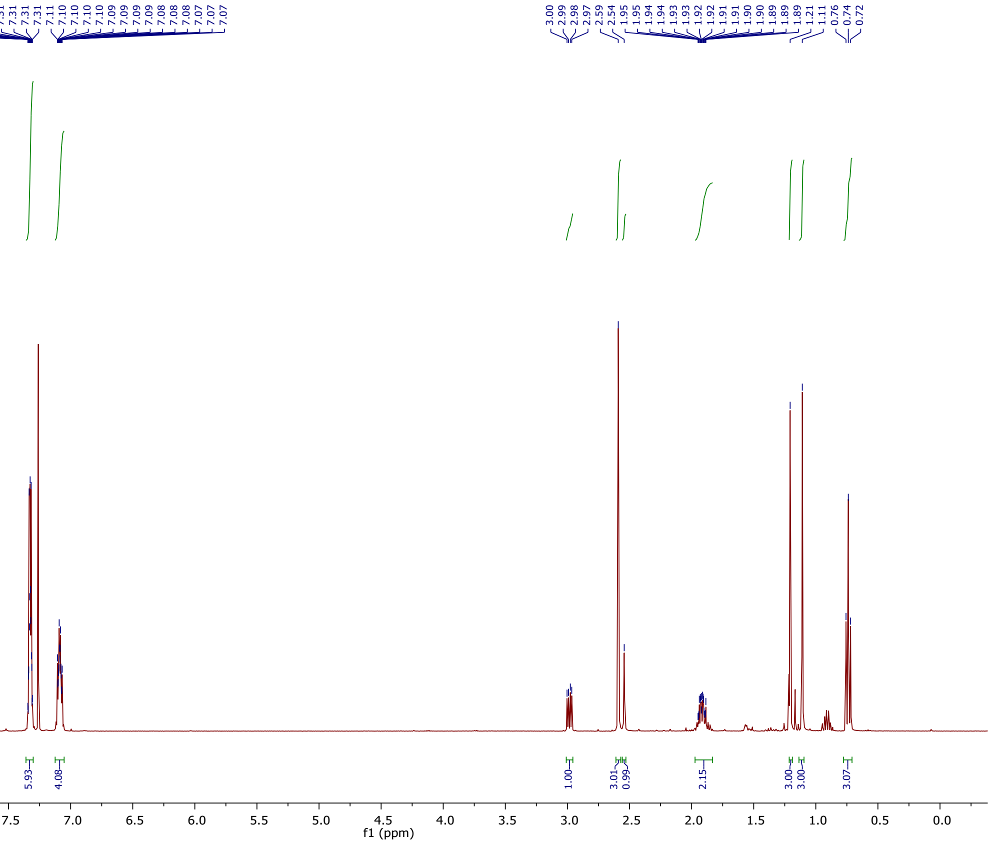

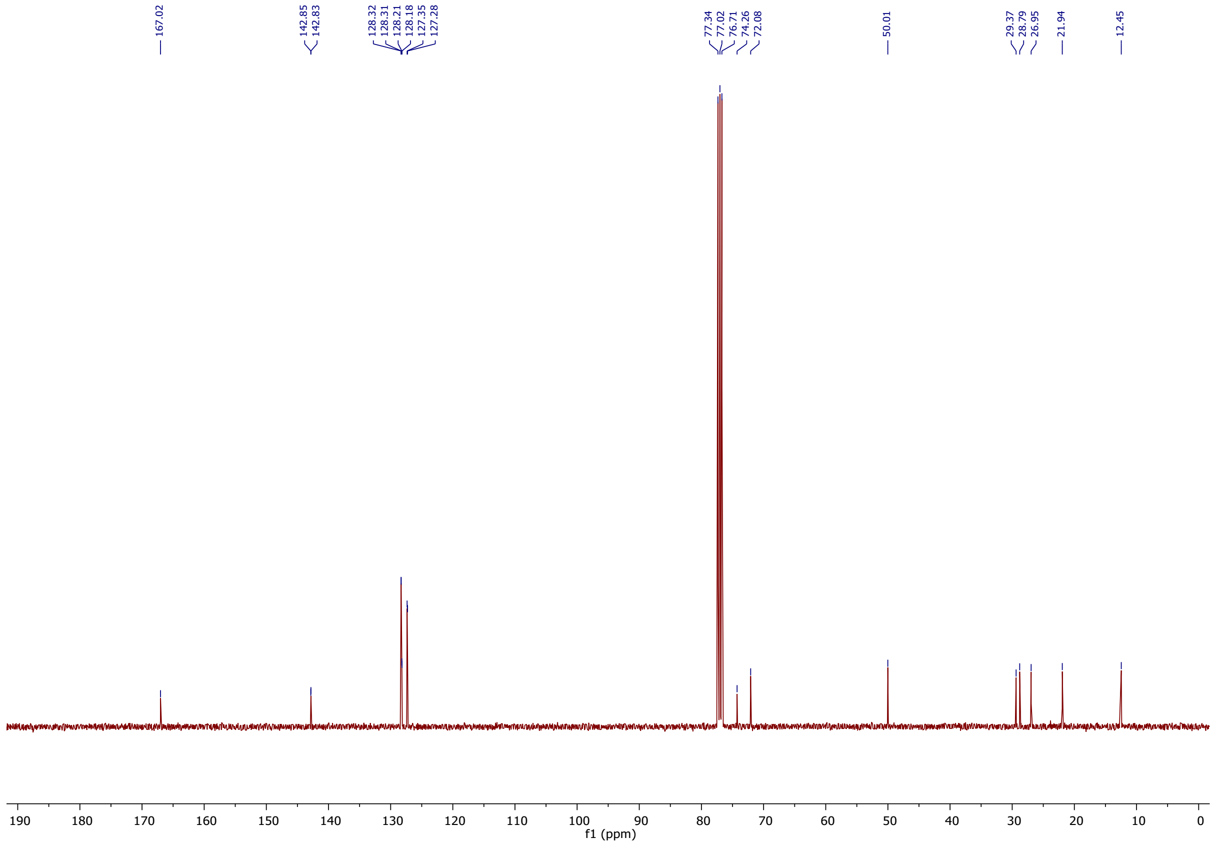

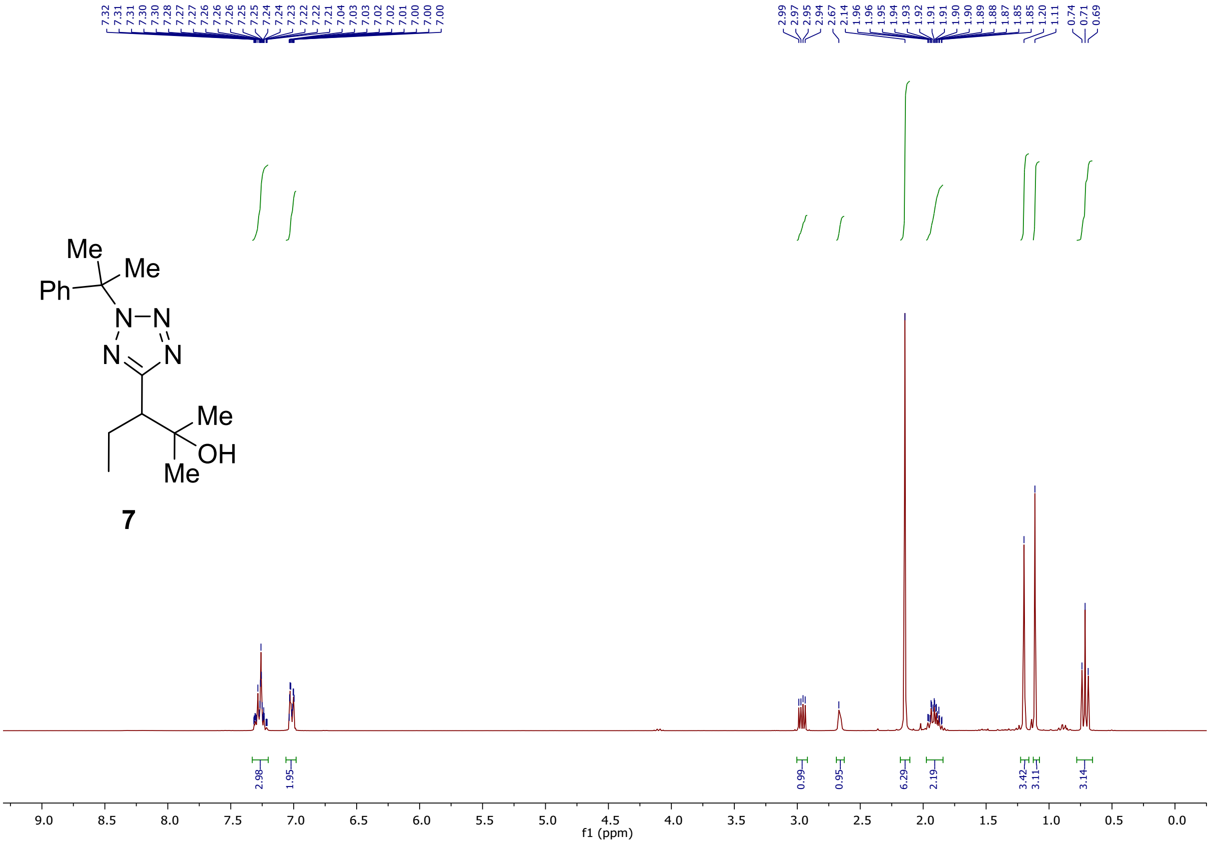
Me
Me
Ph
N
N
N
N
Me
OH
Me
7
f1 (ppm)

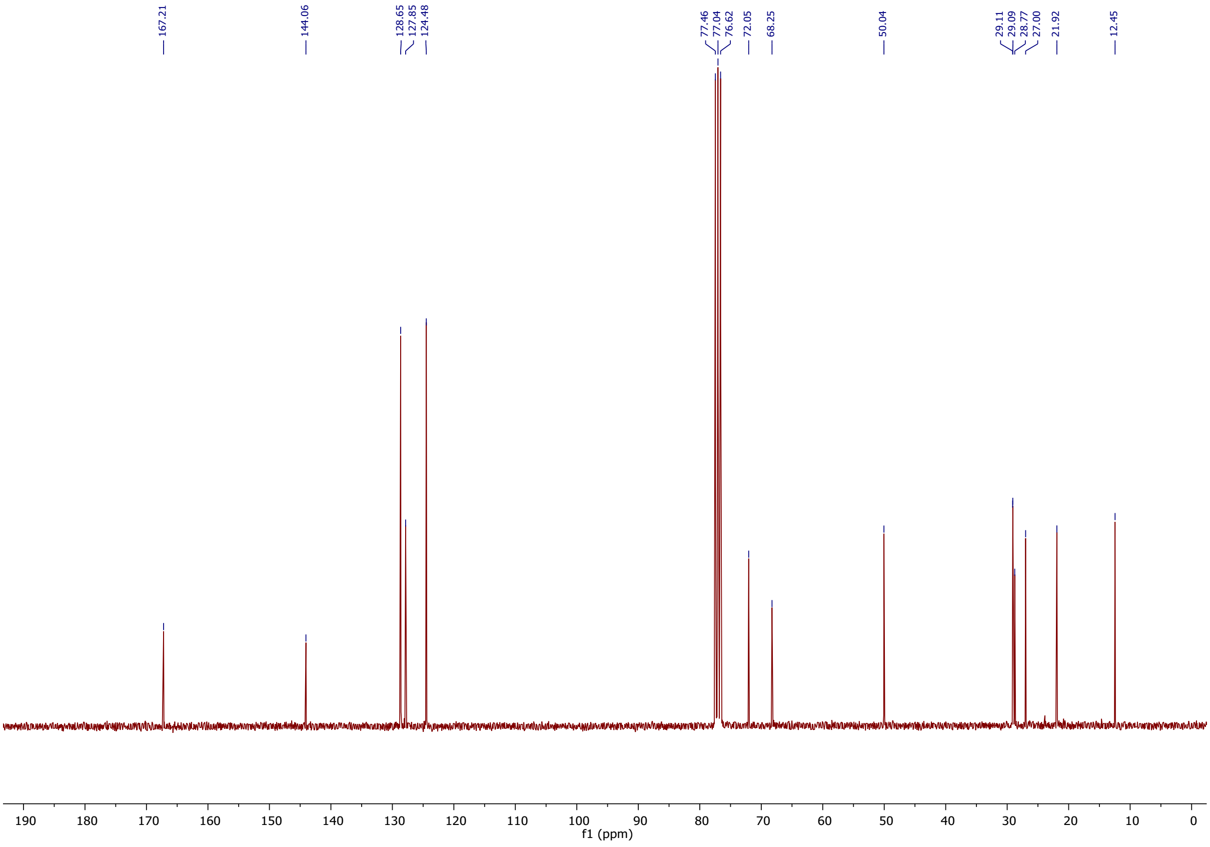
f1 (ppm)

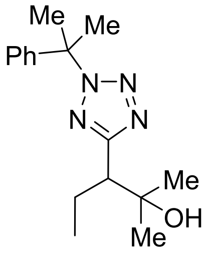

rac-**7**

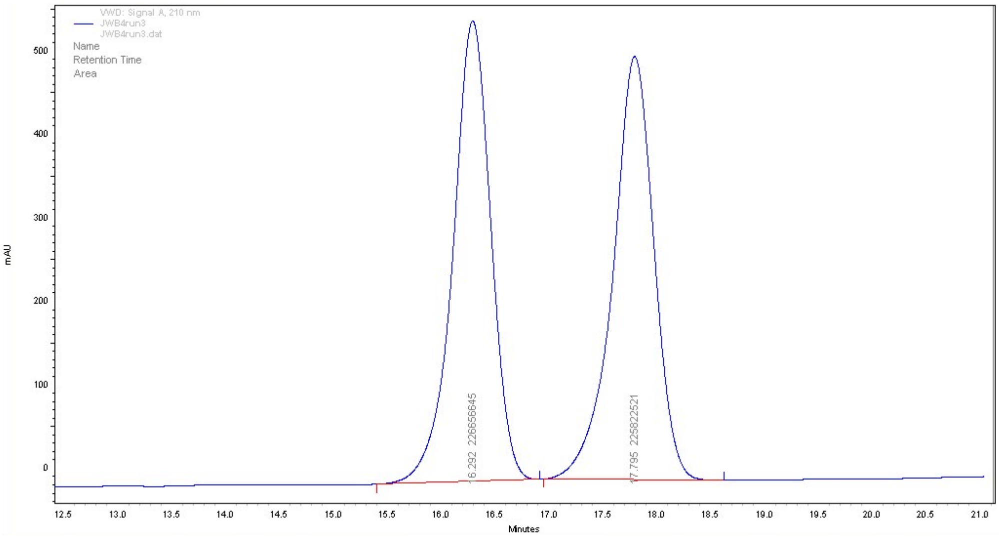

VWD: Signal A,
210 nm Results

Retention Time	Area	Area %	Height	Height %
16.292	226656645	50.09	9254261	52.11
17.795	225822521	49.91	8506465	47.89
Totals	452479166	100.00	17760726	100.00

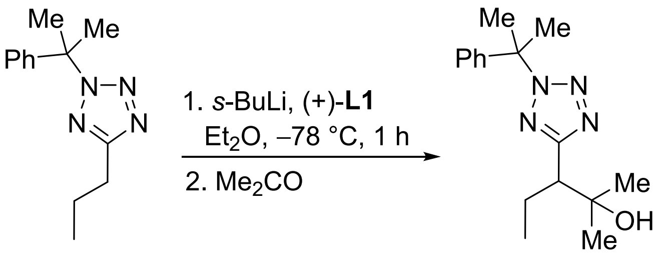

5 **7**

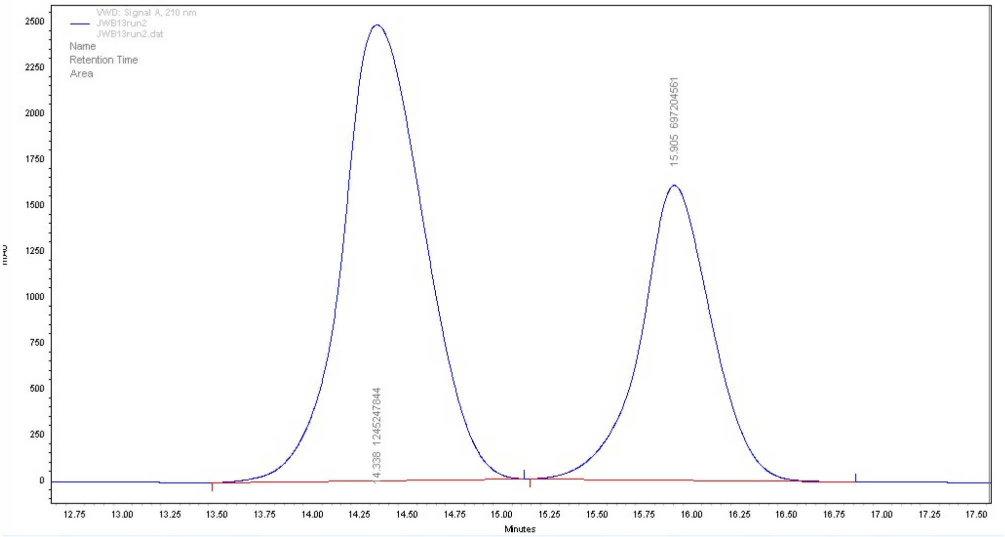

VWD: Signal A, 210 nm Results

Retention Time	Area	Area %	Height	Height %
14.338	1245247844	64.11	41636550	60.72
15.905	697204561	35.89	26935776	39.28
Totals	1942452405	100.00	68572326	100.00

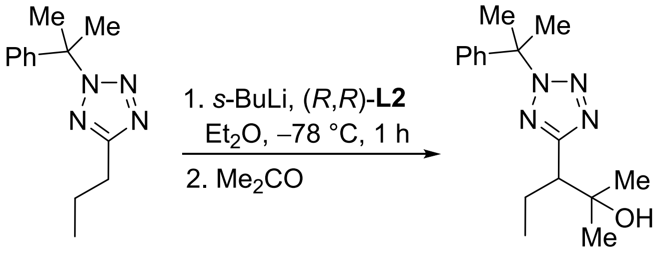

5 7

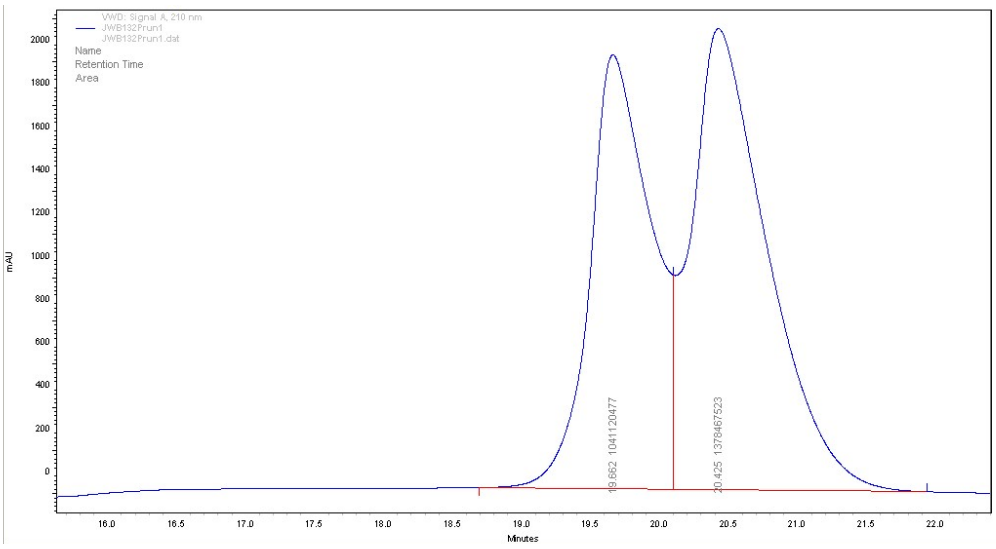

VWD: Signal A, 210 nm Results

Retention Time	Area	Area %	Height	Height %
19.662	1041120477	43.03	33755376	48.48
20.425	1378467523	56.97	35866028	51.52
Totals	2419588000	100.00	69621404	100.00

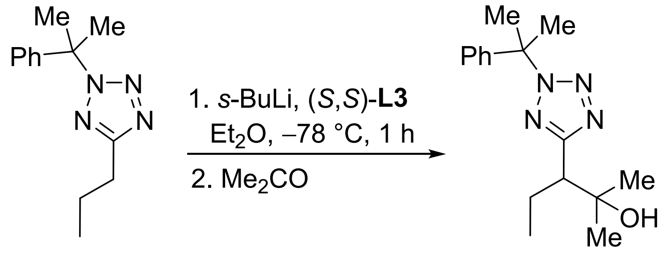

5 7

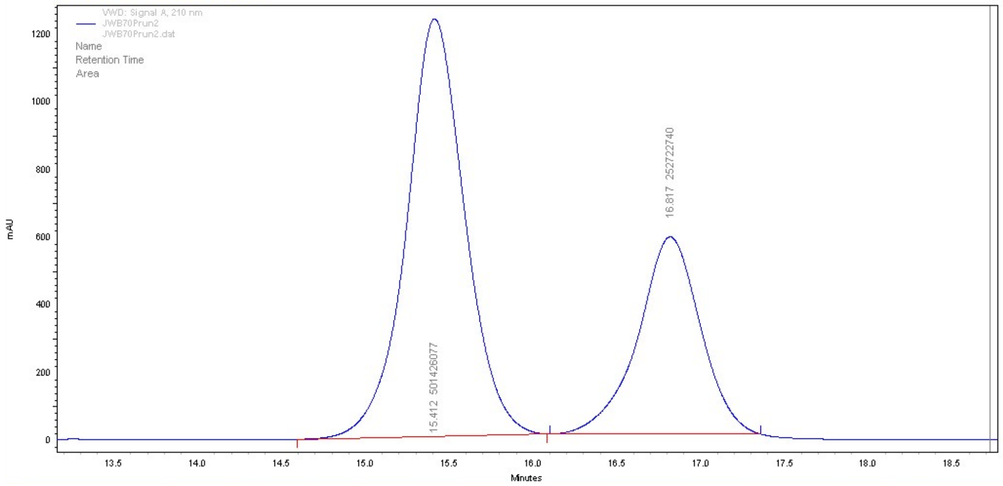

VWD: Signal A, 210 nm Results

Retention Time	Area	Area %	Height	Height %
15.412	501426077	66.49	20702061	67.94
16.817	252722740	33.51	9768513	32.06
Totals	754148817	100.00	30470574	100.00

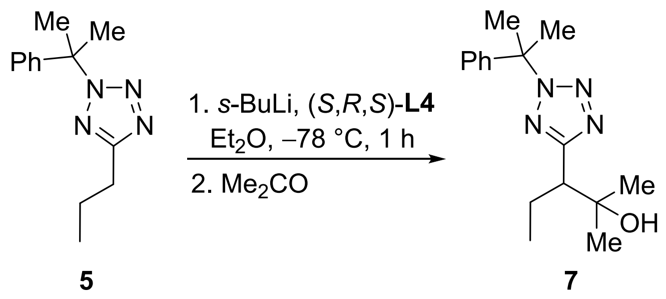

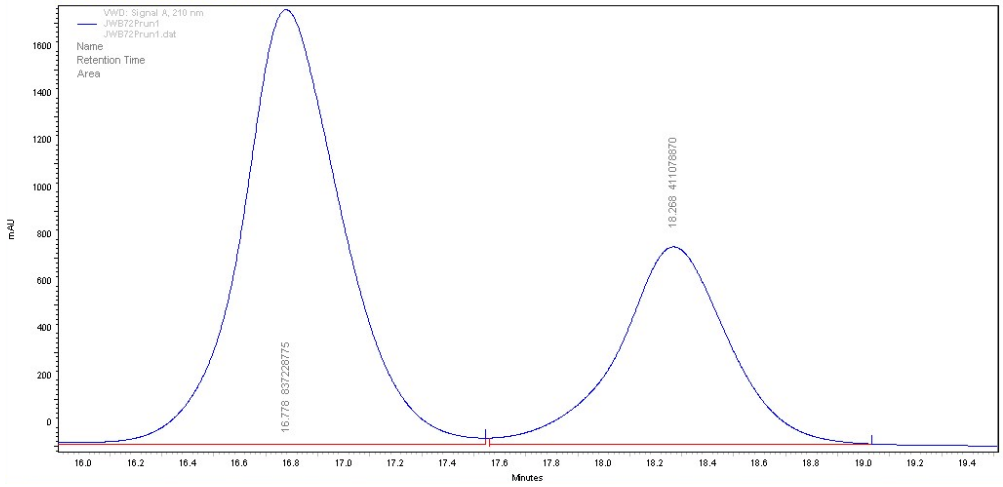

VWD: Signal A,
210 nm Results

Retention Time	Area	Area %	Height	Height %
16.778	837228775	67.07	30969856	68.78
18.268	411078870	32.93	14054822	31.22
Totals	1248307645	100.00	45024678	100.00

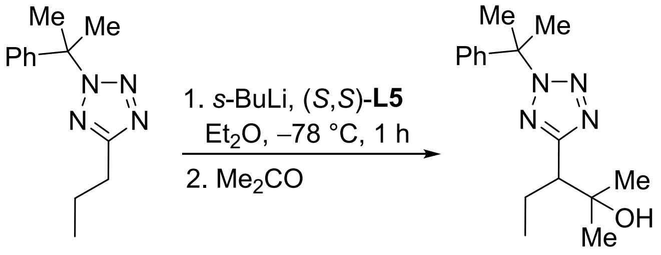

5 **7**

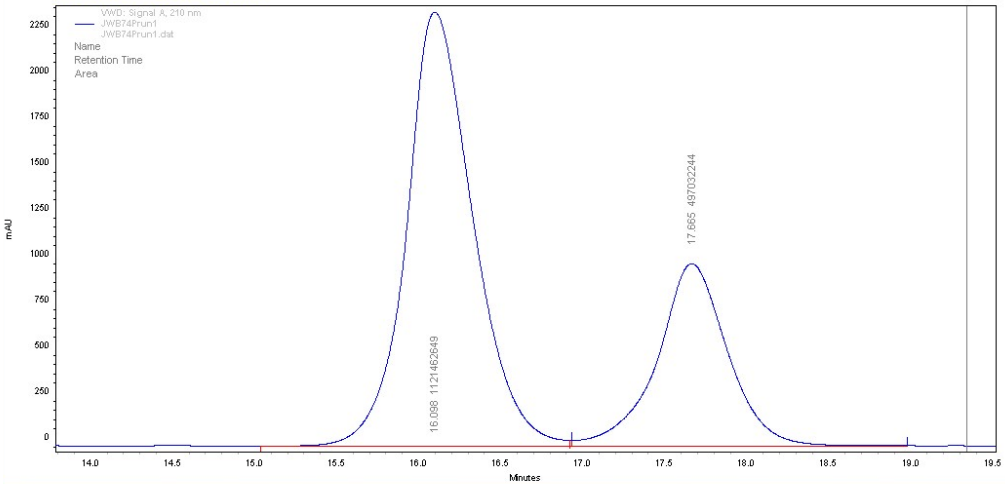

VWD: Signal A,
210 nm Results

Retention Time	Area	Area %	Height	Height %
16.098	1121462649	69.29	39725408	70.43
17.665	497032244	30.71	16680867	29.57
Totals	1618494893	100.00	56406275	100.00

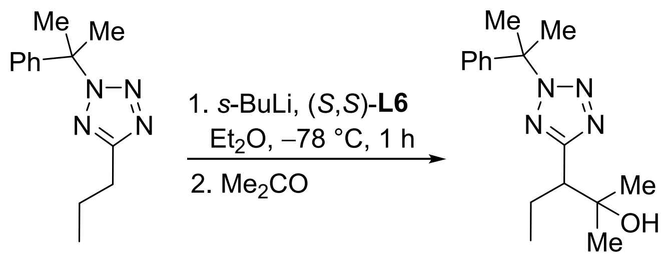

5 **7**

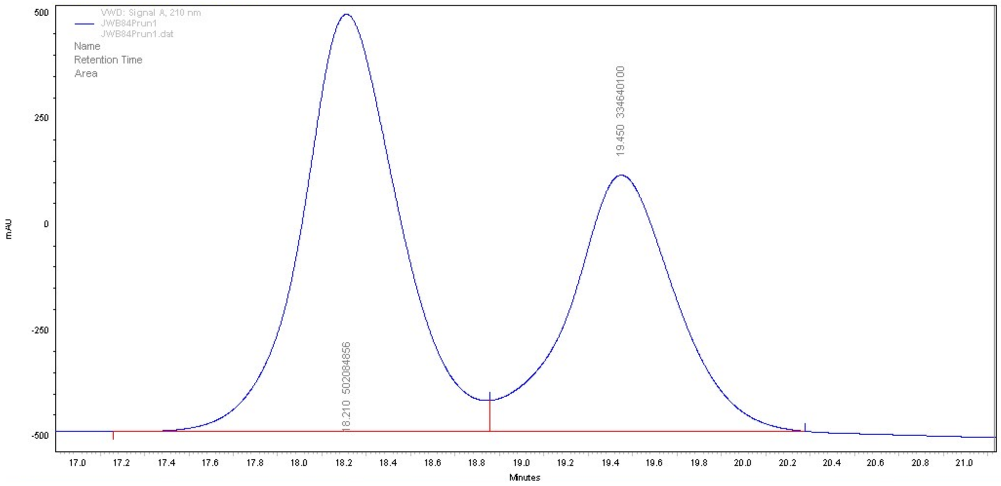

VWD: Signal A,
210 nm Results

Retention Time	Area	Area %	Height	Height %
18.210	502084856	60.01	16525736	61.95
19.450	334640100	39.99	10148542	38.05
Totals	836724956	100.00	26674278	100.00

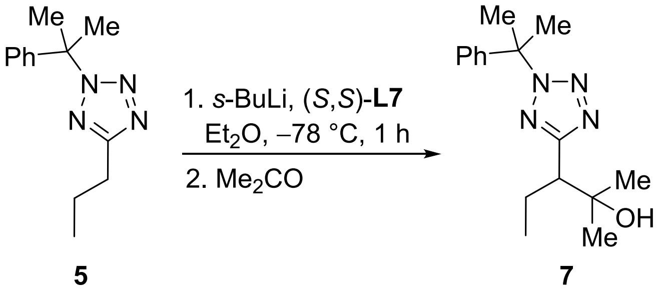

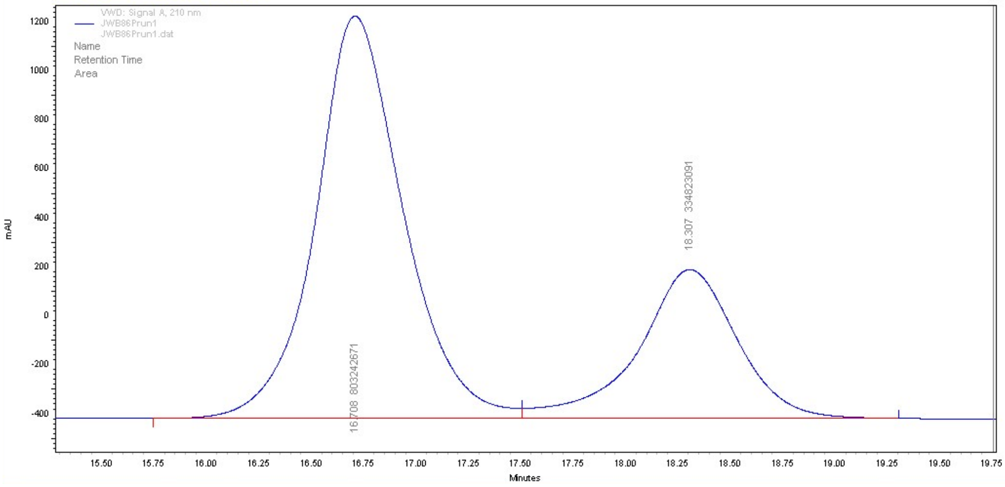

VWD: Signal A, 210 nm Results

Retention Time	Area	Area %	Height	Height %
16.708	803242671	70.58	27553298	73.01
18.307	334823091	29.42	10188077	26.99
Totals	1138065762	100.00	37741375	100.00

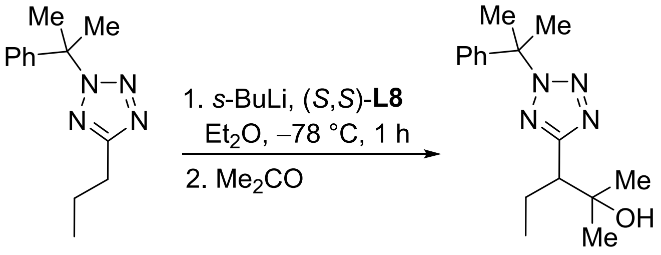

5 7

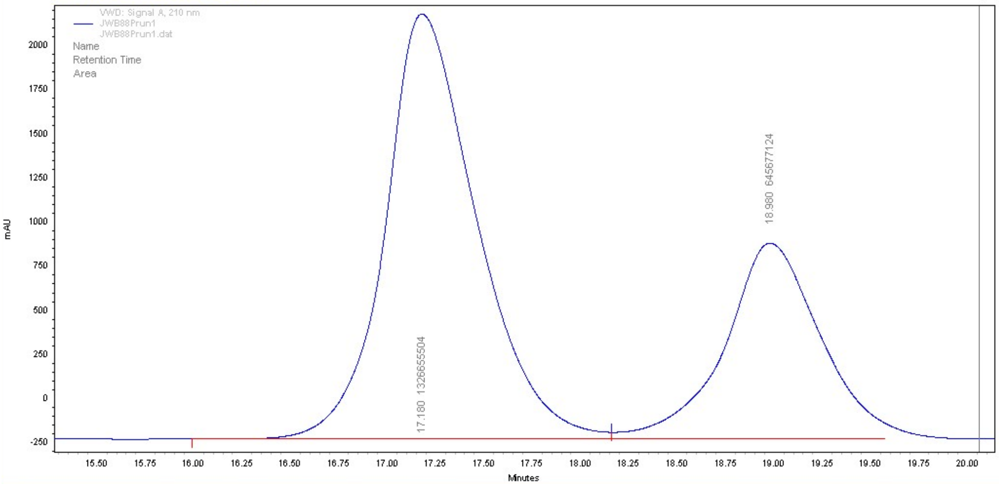

VWD: Signal A, 210 nm Results

Retention Time	Area	Area %	Height	Height %
17.180	1326655504	67.26	40381214	68.46
18.980	645677124	32.74	18602710	31.54
Totals	1972332628	100.00	58983924	100.00

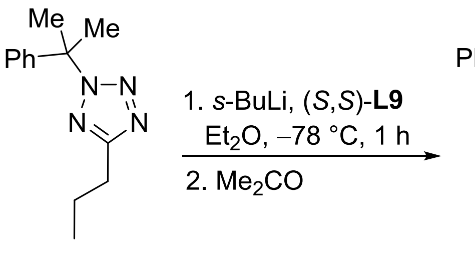

5 7

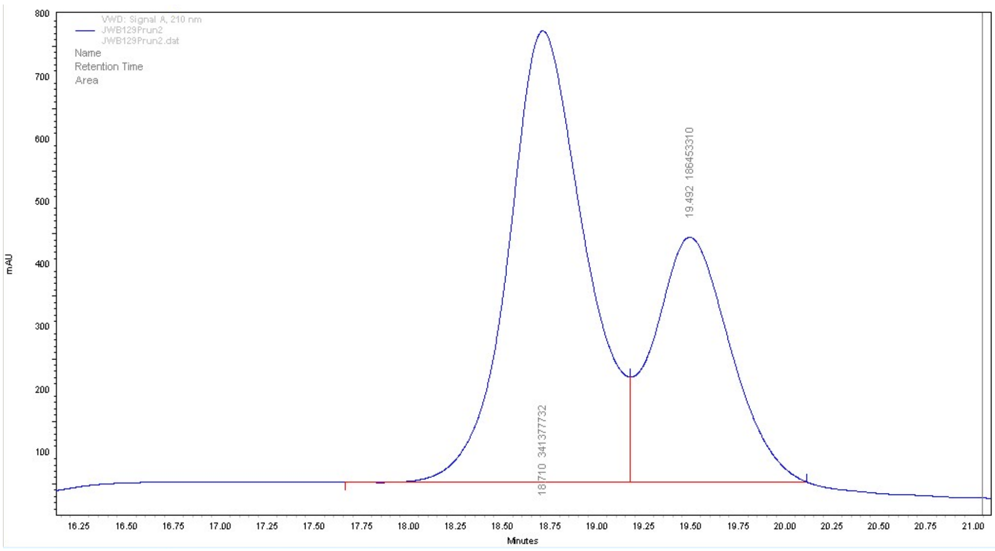

VWD: Signal A, 210 nm Results

Retention Time	
18.710	[illegible]
19.492	[illegible]
Totals	[illegible]

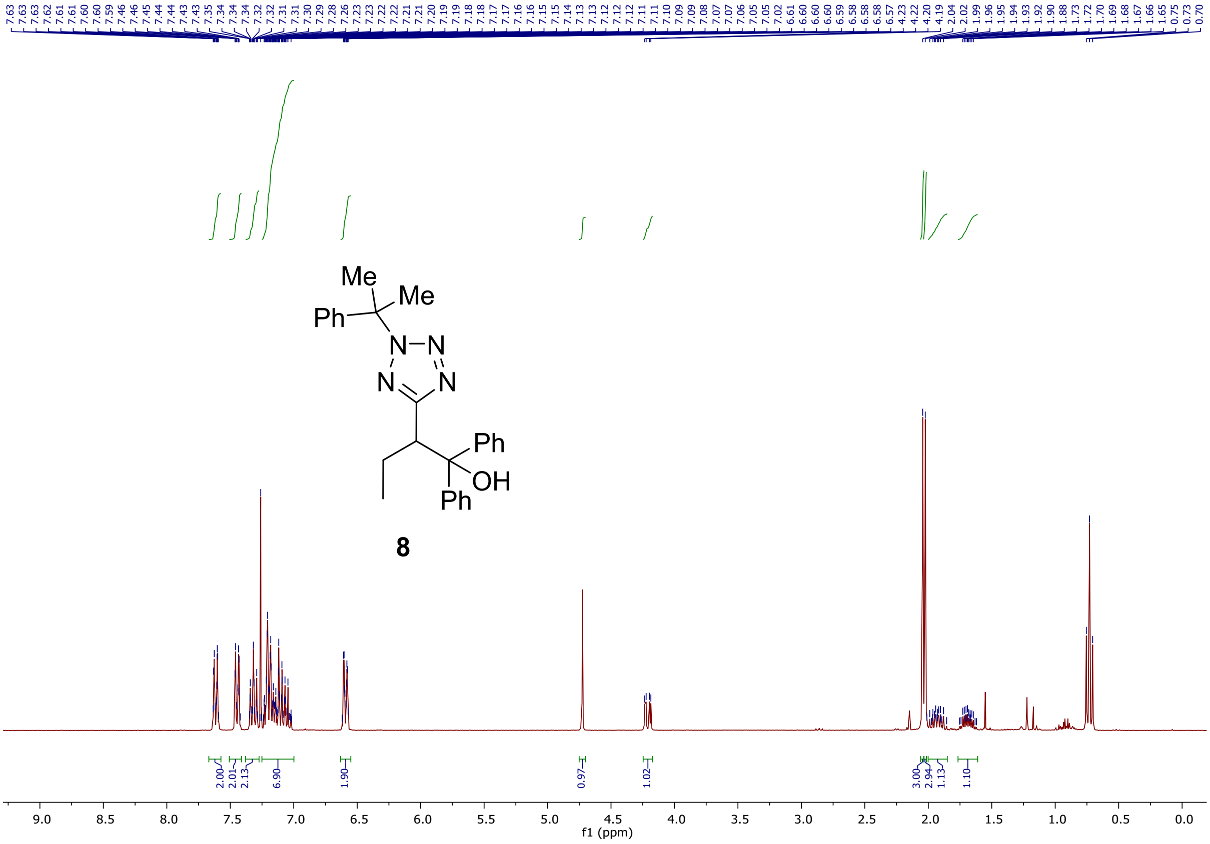

8

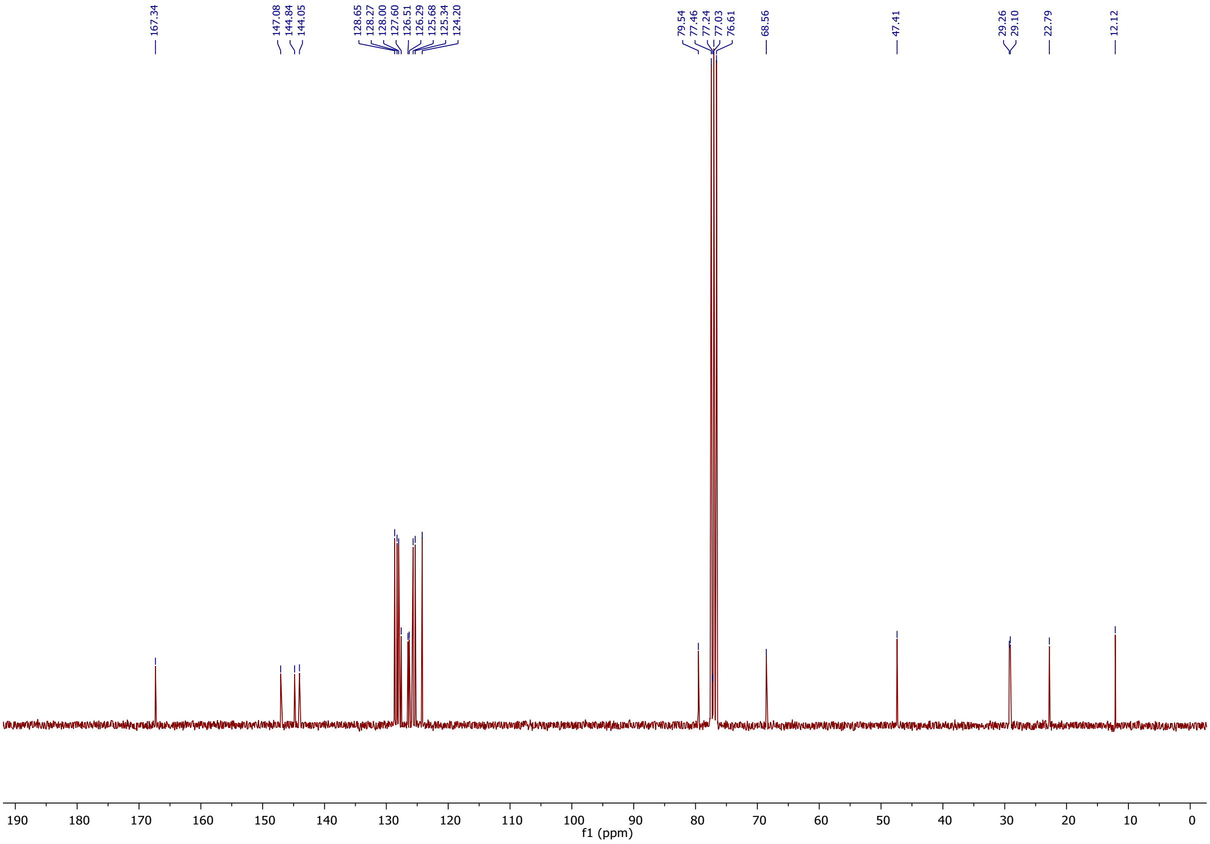
167.34
147.08
144.84
144.05
128.65
128.27
128.00
127.60
126.51
126.29
125.68
125.34
124.20
79.54
77.46
77.24
77.03
76.61
68.56
47.41
29.26
29.10
22.79
12.12
f1 (ppm)

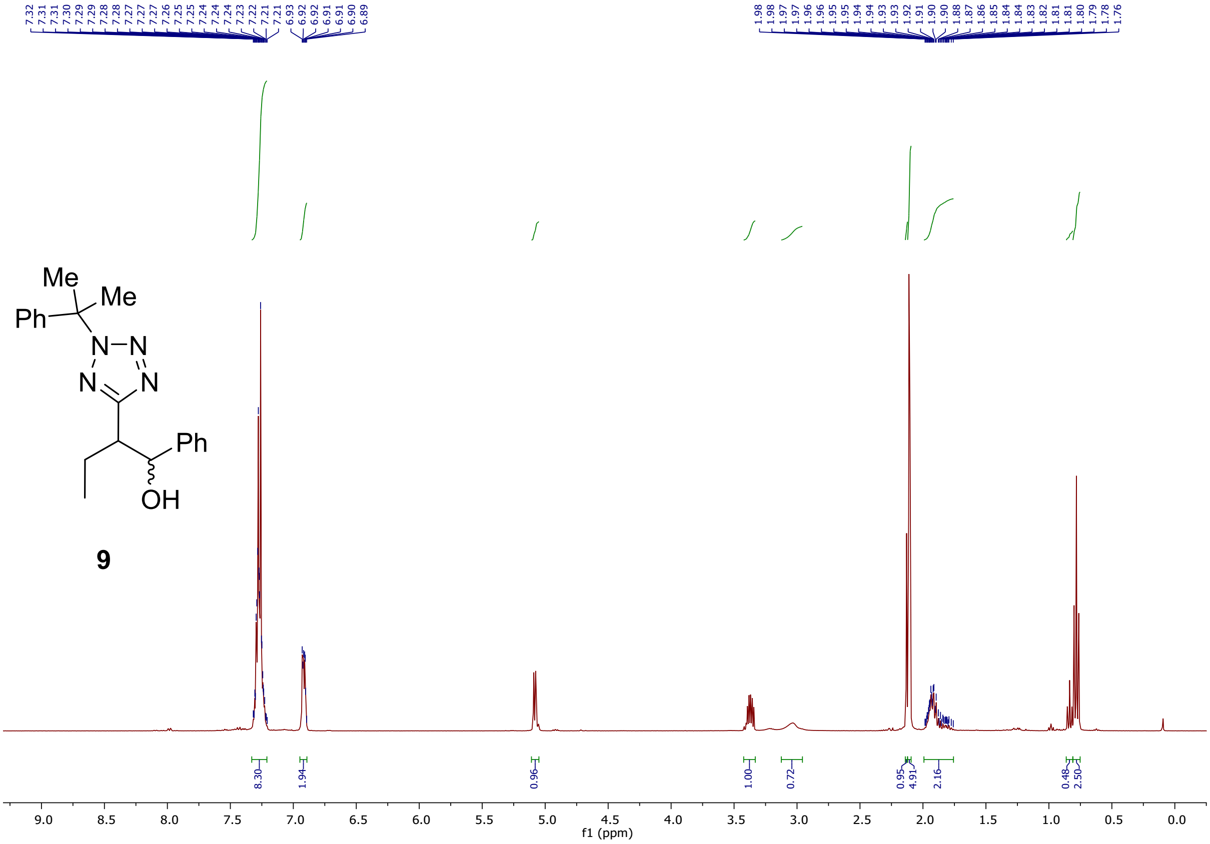
7.32
7.31
7.30
7.29
7.29
7.28
7.28
7.27
7.27
7.27
7.26
7.25
7.25
7.24
7.24
7.24
7.23
7.22
7.21
7.21
6.93
6.92
6.92
6.91
6.91
6.90
6.89
1.98
1.98
1.97
1.97
1.96
1.96
1.95
1.94
1.94
1.93
1.93
1.92
1.91
1.90
1.90
1.88
1.87
1.86
1.85
1.84
1.84
1.83
1.82
1.81
1.80
1.79
1.78
1.76
Me
Me
Ph
N
N
N
N
Ph
OH
9
8.30
1.94
0.96
1.00
0.72
0.95
4.91
2.16
0.48
2.50
f1 (ppm)

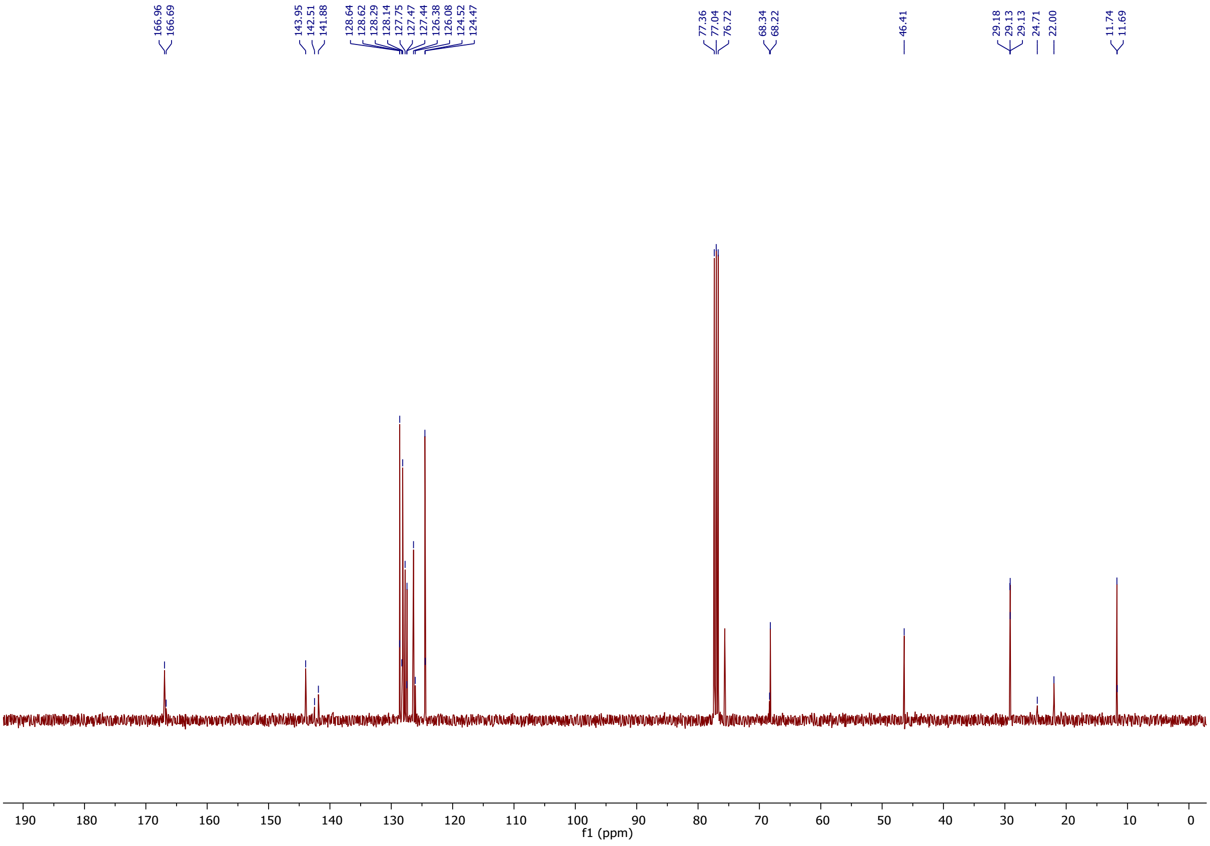
f1 (ppm)

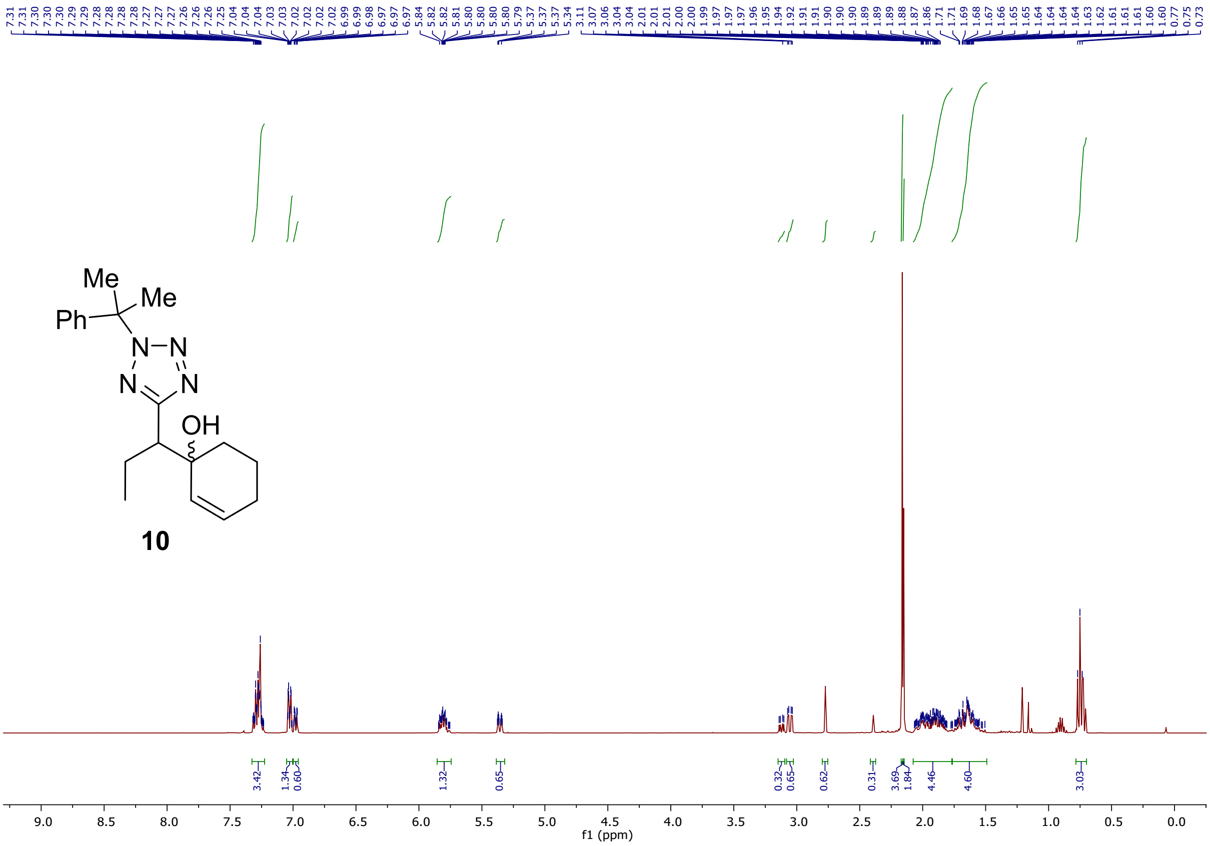
Me
Me
Ph
N
N
N
N
OH
10
f1 (ppm)

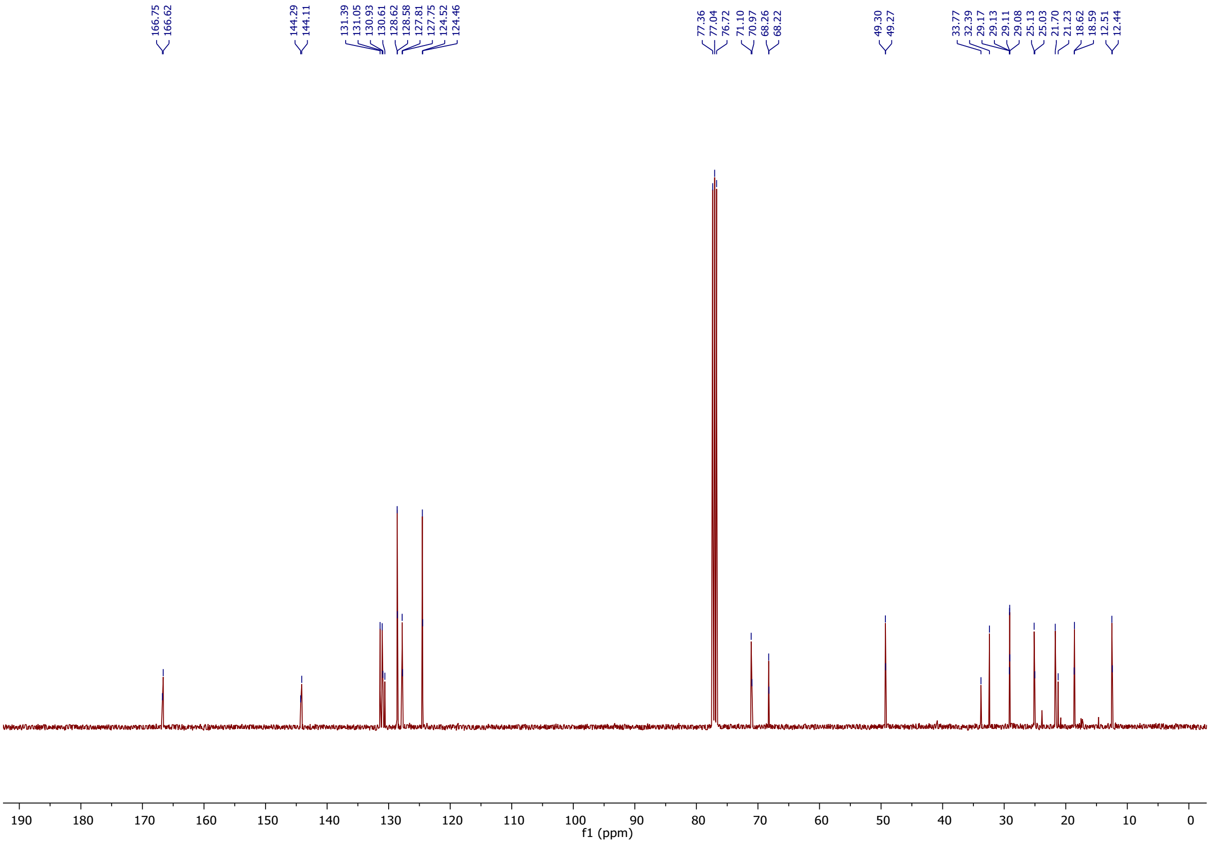
166.75
166.62
144.29
144.11
131.39
131.05
130.93
130.61
128.62
128.58
127.81
127.75
124.52
124.46
77.36
77.04
76.72
71.10
70.97
68.26
68.22
49.30
49.27
33.77
32.39
29.17
29.13
29.11
29.08
25.13
25.03
21.70
21.23
18.62
18.59
12.51
12.44
190 180 170 160 150 140 130 120 110 100 90 80 70 60 50 40 30 20 10 0
f1 (ppm)

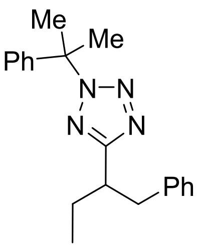
Me
Me
Ph
N
N
N
N
Ph

11

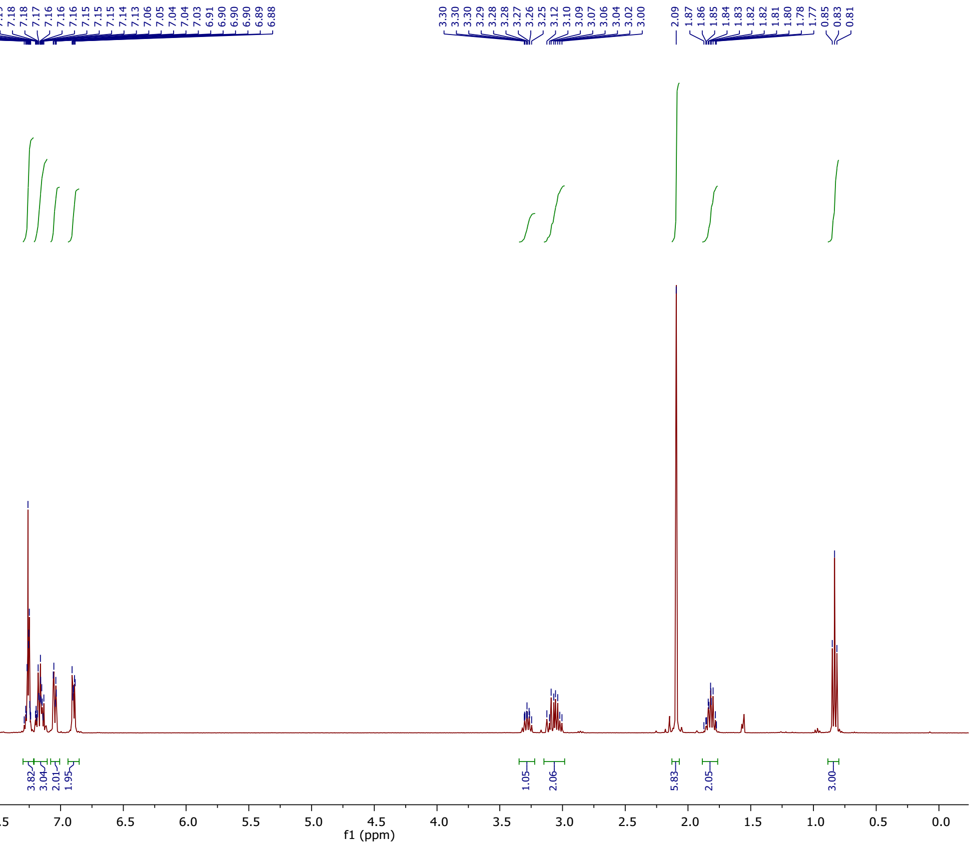
7.29
7.28
7.27
7.27
7.26
7.26
7.25
7.25
7.24
7.24
7.24
7.20
7.20
7.19
7.18
7.17
7.16
7.16
7.16
7.15
7.15
7.14
7.13
7.06
7.05
7.04
7.04
7.03
6.91
6.90
6.90
6.89
6.88
3.30
3.30
3.29
3.28
3.28
3.27
3.26
3.25
3.12
3.10
3.09
3.07
3.06
3.04
3.02
3.00
2.09
1.87
1.86
1.85
1.84
1.83
1.82
1.82
1.81
1.80
1.78
1.77
0.85
0.83
0.81
3.82
3.04
2.01
1.95
1.05
2.06
5.83
2.05
3.00
9.0 8.5 8.0 7.5 7.0 6.5 6.0 5.5 5.0 4.5 4.0 3.5 3.0 2.5 2.0 1.5 1.0 0.5 0.0
f1 (ppm)

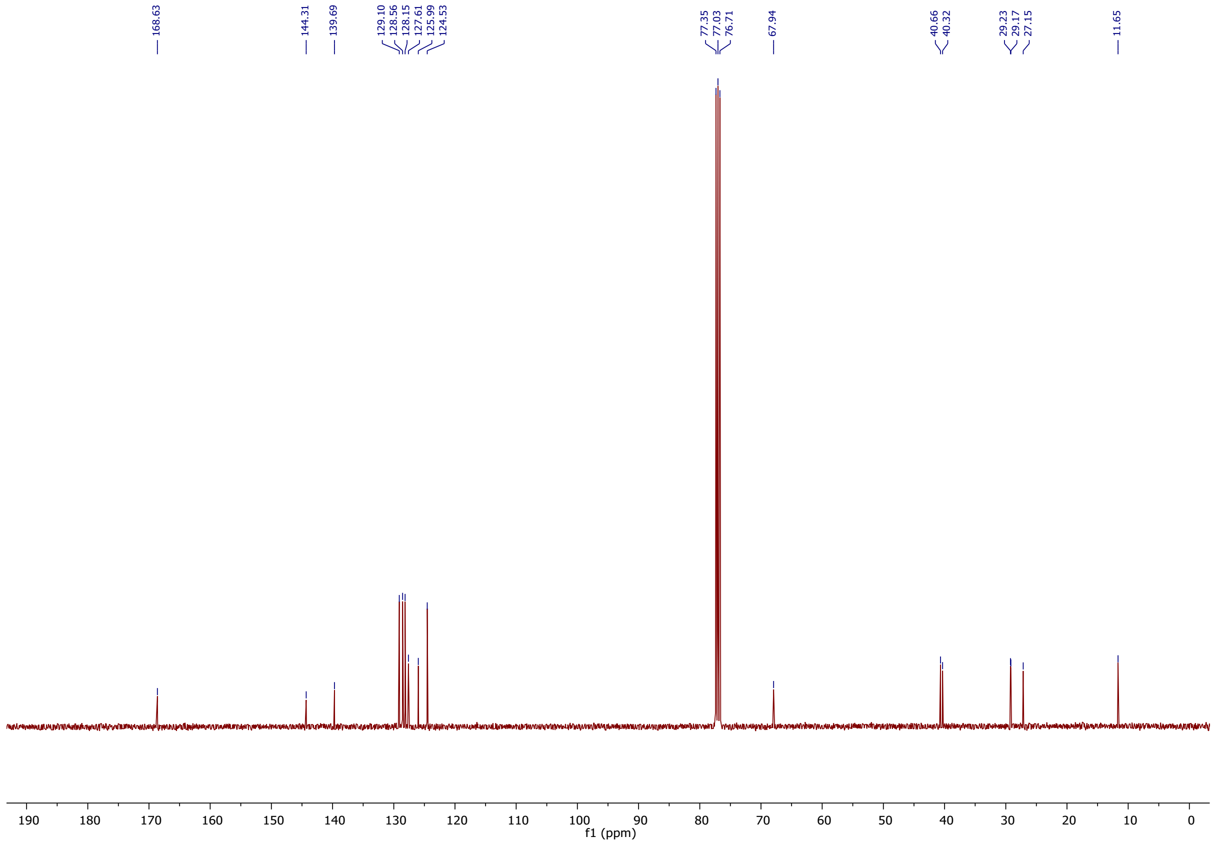
168.63
144.31
139.69
129.10
128.56
128.15
127.61
125.99
124.53
77.35
77.03
76.71
67.94
40.66
40.32
29.23
29.17
27.15
11.65
f1 (ppm)

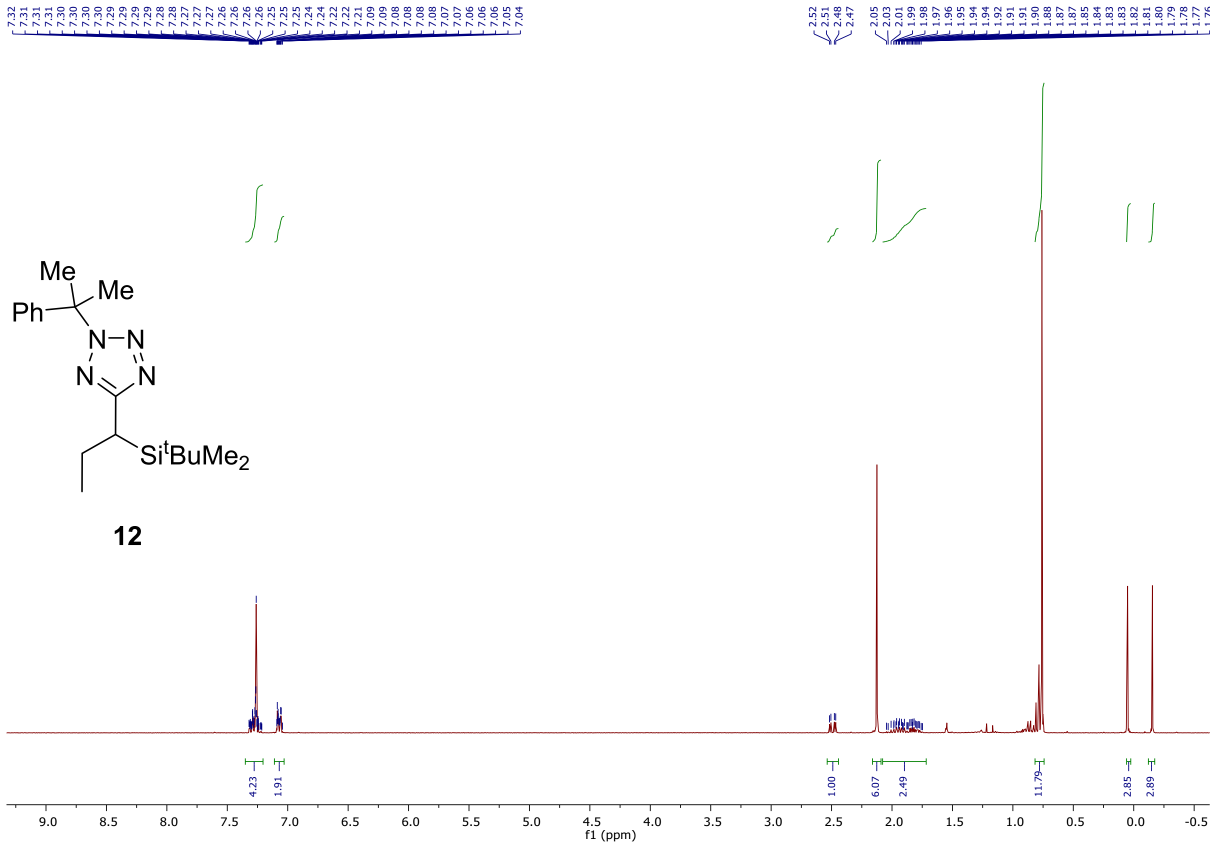
4.23
1.91
1.00
6.07
2.49
11.79
2.85
2.89
f1 (ppm)
Me
Me
Ph
N
N
N
N
Si^{t}BuMe$_2$

12

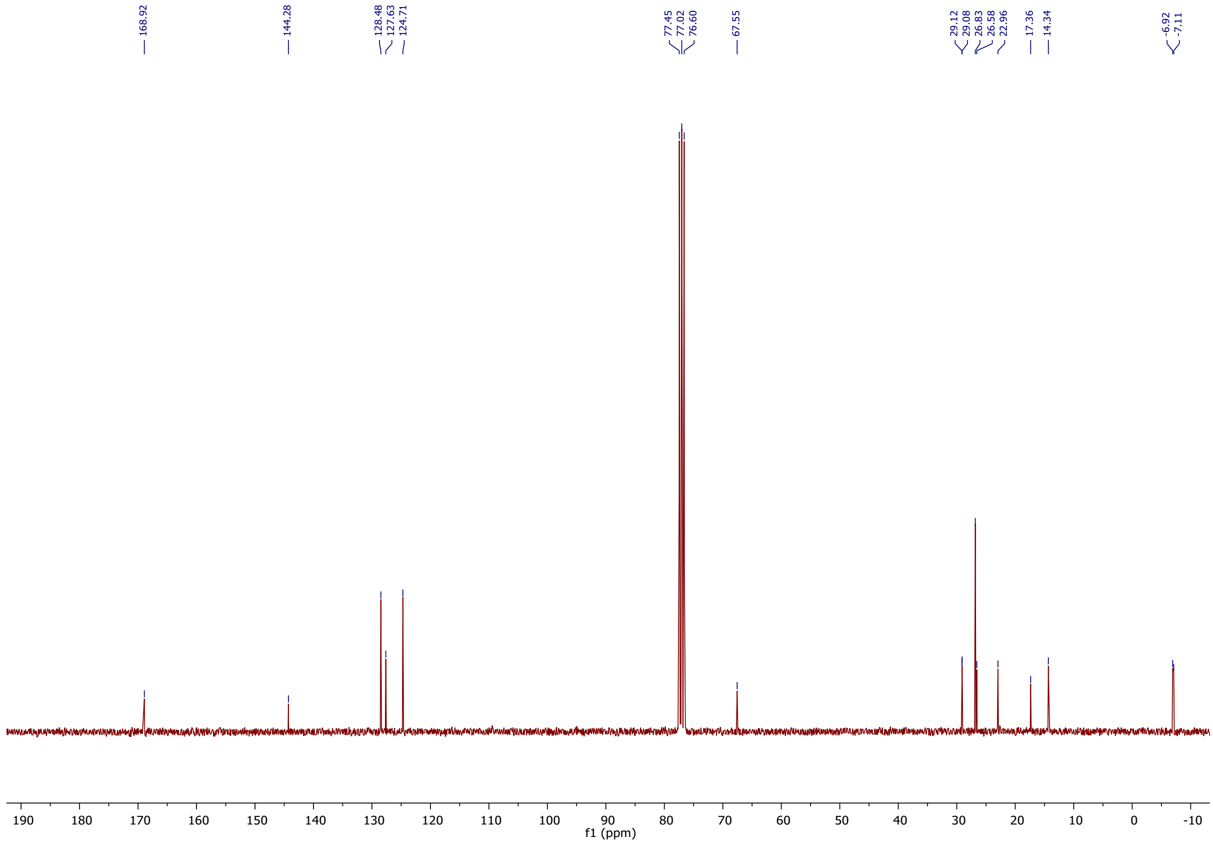
168.92
144.28
128.48
127.63
124.71
77.45
77.02
76.60
67.55
29.12
29.08
26.83
26.58
22.96
17.36
14.34
-6.92
-7.11
190 180 170 160 150 140 130 120 110 100 90 80 70 60 50 40 30 20 10 0 -10
f1 (ppm)

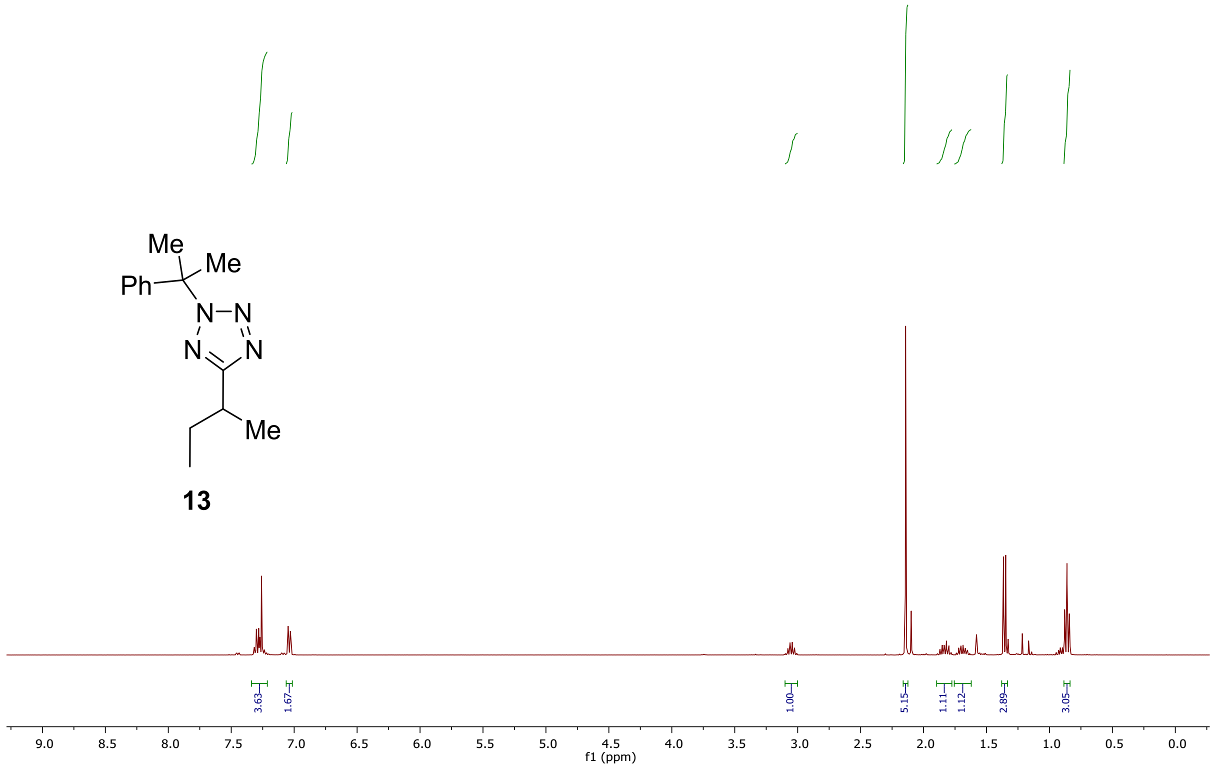
Me
Me
Ph
N
N
N
N
Me
13
3.63
1.67
1.00
5.15
1.11
1.12
2.89
3.05
9.0 8.5 8.0 7.5 7.0 6.5 6.0 5.5 5.0 4.5 4.0 3.5 3.0 2.5 2.0 1.5 1.0 0.5 0.0
f1 (ppm)

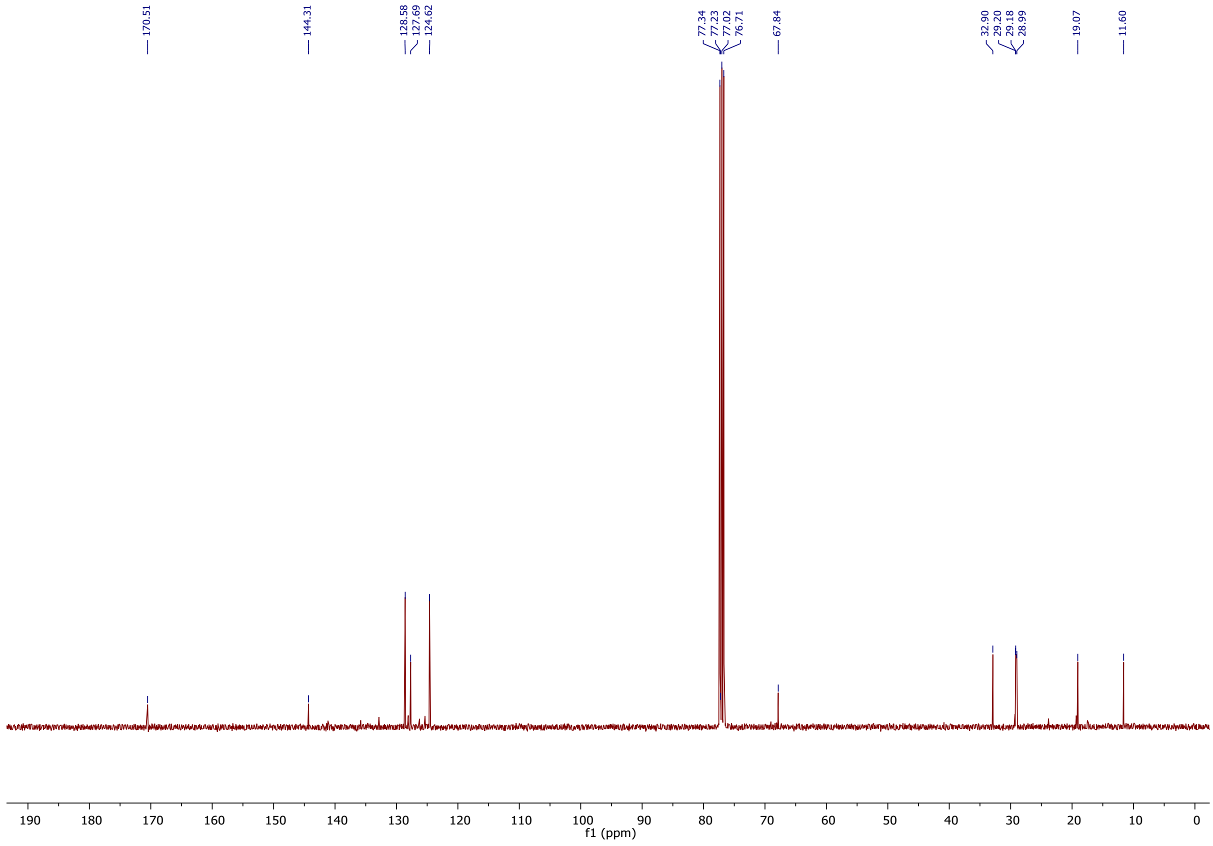
170.51
144.31
128.58
127.69
124.62
77.34
77.23
77.02
76.71
67.84
32.90
29.20
29.18
28.99
19.07
11.60
190 180 170 160 150 140 130 120 110 100 90 80 70 60 50 40 30 20 10 0
f1 (ppm)

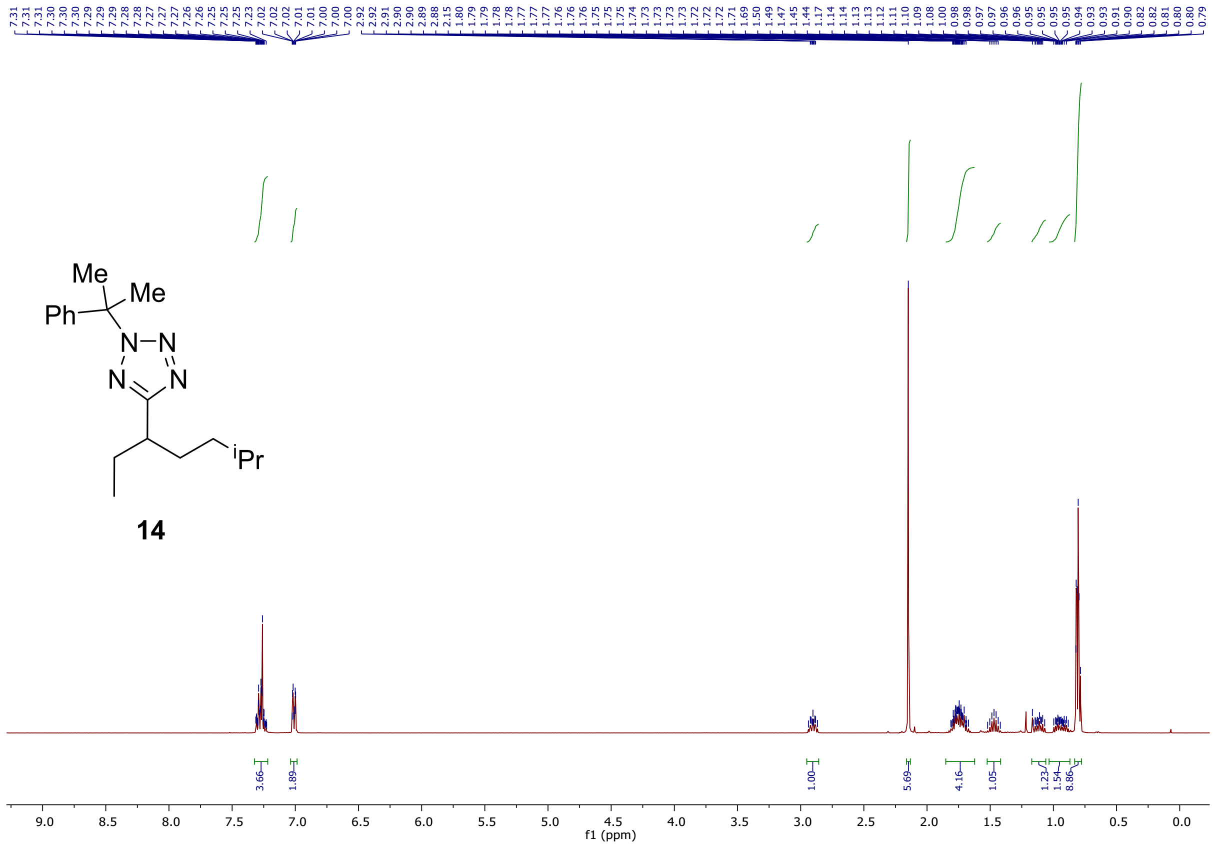
7.31 7.31 7.31 7.30 7.30 7.29 7.29 7.28 7.28 7.27 7.27 7.26 7.26 7.25 7.25 7.23 7.02 7.02 7.02 7.01 7.01 7.00 7.00
2.92 2.91 2.90 2.90 2.89 2.88 2.15 1.80 1.79 1.79 1.78 1.78 1.77 1.77 1.76 1.76 1.76 1.75 1.75 1.74 1.73 1.73 1.73 1.73 1.72 1.72 1.72 1.71 1.69 1.50 1.49 1.47 1.45 1.44 1.17 1.17 1.14 1.14 1.13 1.13 1.12 1.11 1.10 1.09 1.08 1.00 0.98 0.98 0.97 0.97 0.96 0.96 0.95 0.95 0.95 0.95 0.94 0.93 0.93 0.91 0.90 0.82 0.81 0.80 0.80 0.79
Me
Me
Ph
N
N
N
N
iPr
14
3.66
1.89
1.00
5.69
4.16
1.05
1.23
1.54
8.86
9.0 8.5 8.0 7.5 7.0 6.5 6.0 5.5 5.0 4.5 4.0 3.5 3.0 2.5 2.0 1.5 1.0 0.5 0.0
f1 (ppm)

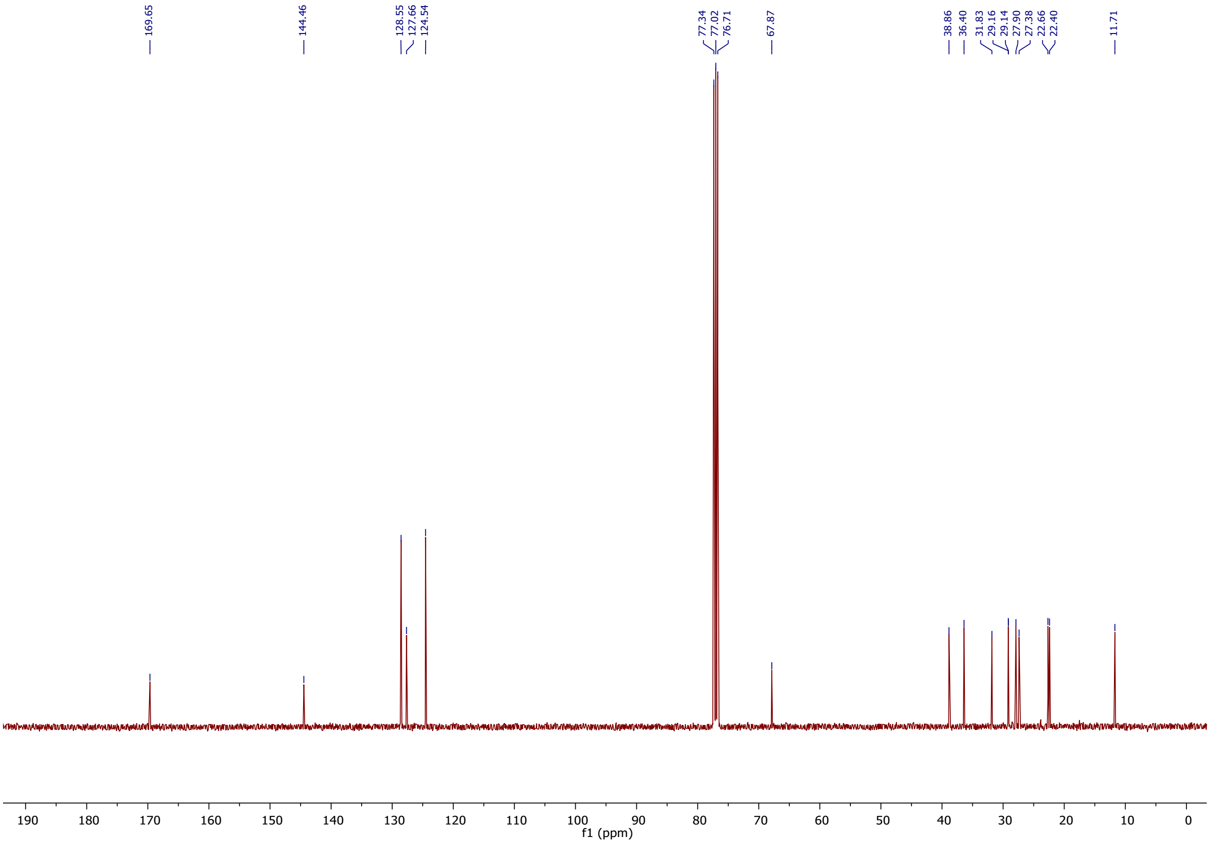
169.65
144.46
128.55
127.66
124.54
77.34
77.02
76.71
67.87
38.86
36.40
31.83
29.16
29.14
27.90
27.38
22.66
22.40
11.71
190 180 170 160 150 140 130 120 110 100 90 80 70 60 50 40 30 20 10 0
f1 (ppm)

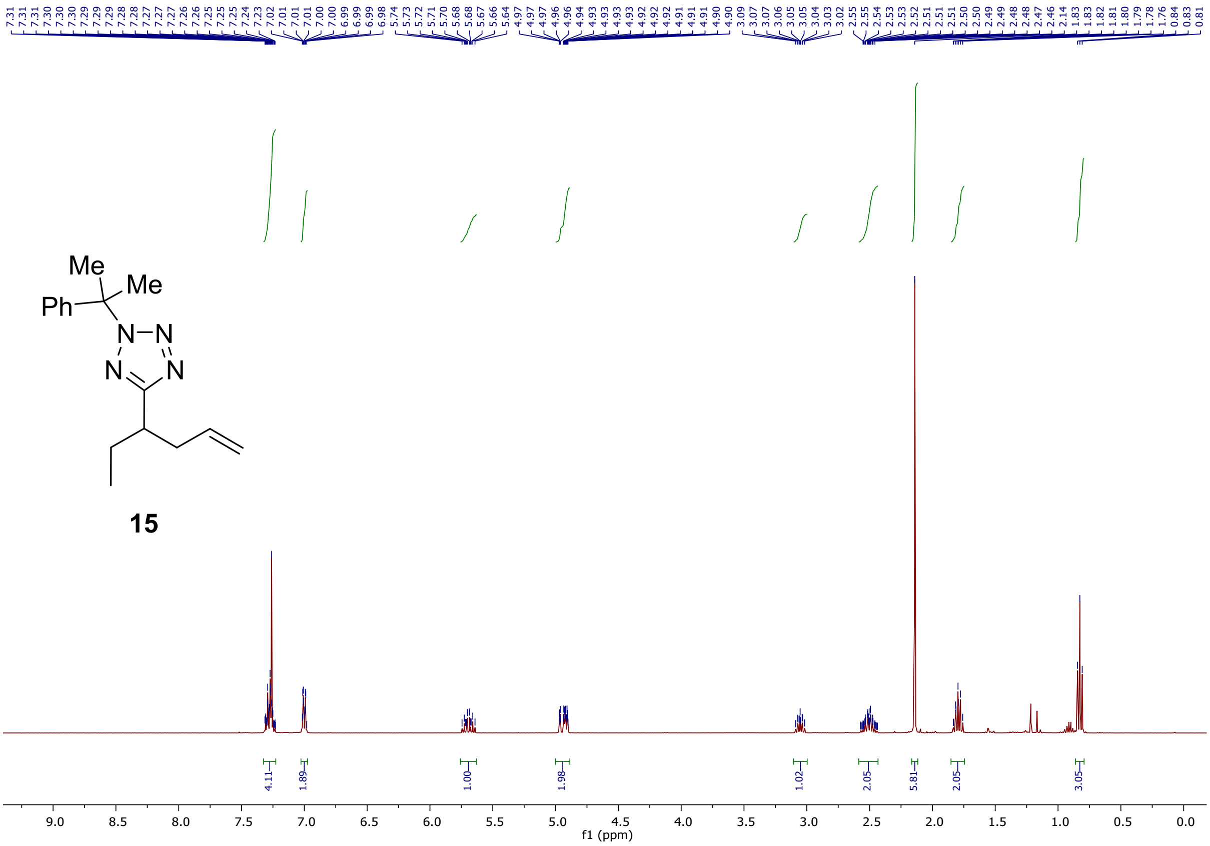
7.31 7.31 7.31 7.30 7.30 7.30 7.29 7.29 7.29 7.28 7.28 7.27 7.27 7.27 7.26 7.26 7.25 7.25 7.25 7.24 7.23 7.02 7.01 7.01 7.01 7.00 7.00 6.99 6.99 6.99 6.98 5.74 5.73 5.72 5.71 5.70 5.68 5.68 5.67 5.66 5.64 4.97 4.97 4.97 4.96 4.96 4.94 4.93 4.93 4.93 4.93 4.92 4.92 4.92 4.91 4.91 4.91 4.90 4.90 3.09 3.07 3.07 3.06 3.05 3.05 3.04 3.03 3.02 2.55 2.55 2.54 2.53 2.53 2.52 2.51 2.51 2.51 2.50 2.50 2.49 2.49 2.48 2.48 2.47 2.46 2.14 1.83 1.83 1.82 1.81 1.80 1.79 1.78 1.76 0.84 0.83 0.81
4.11
1.89
1.00
1.98
1.02
2.05
5.81
2.05
3.05
9.0 8.5 8.0 7.5 7.0 6.5 6.0 5.5 5.0 4.5 4.0 3.5 3.0 2.5 2.0 1.5 1.0 0.5 0.0
f1 (ppm)
Me
Me
Ph
N
N
N
N

15

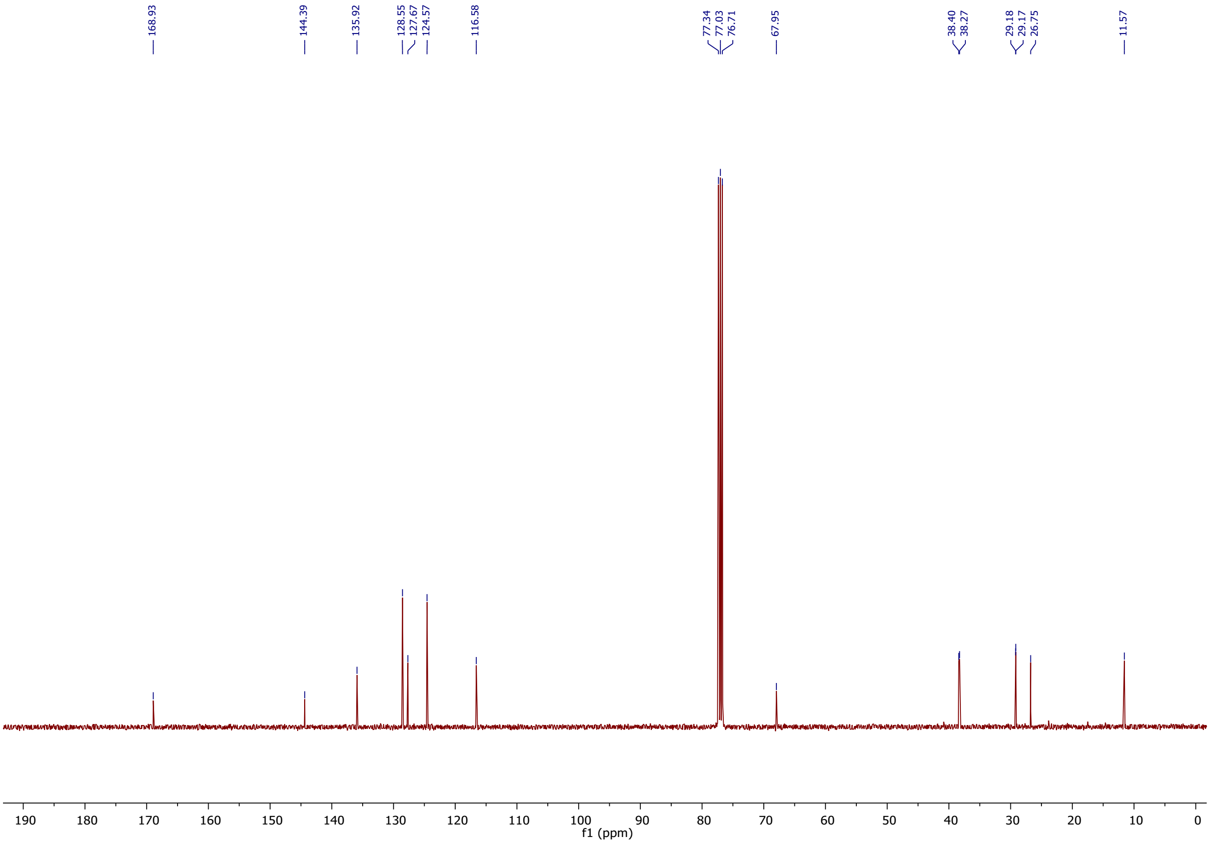
168.93
144.39
135.92
128.55
127.67
124.57
116.58
77.34
77.03
76.71
67.95
38.40
38.27
29.18
29.17
26.75
11.57
190 180 170 160 150 140 130 120 110 100 90 80 70 60 50 40 30 20 10 0
f1 (ppm)

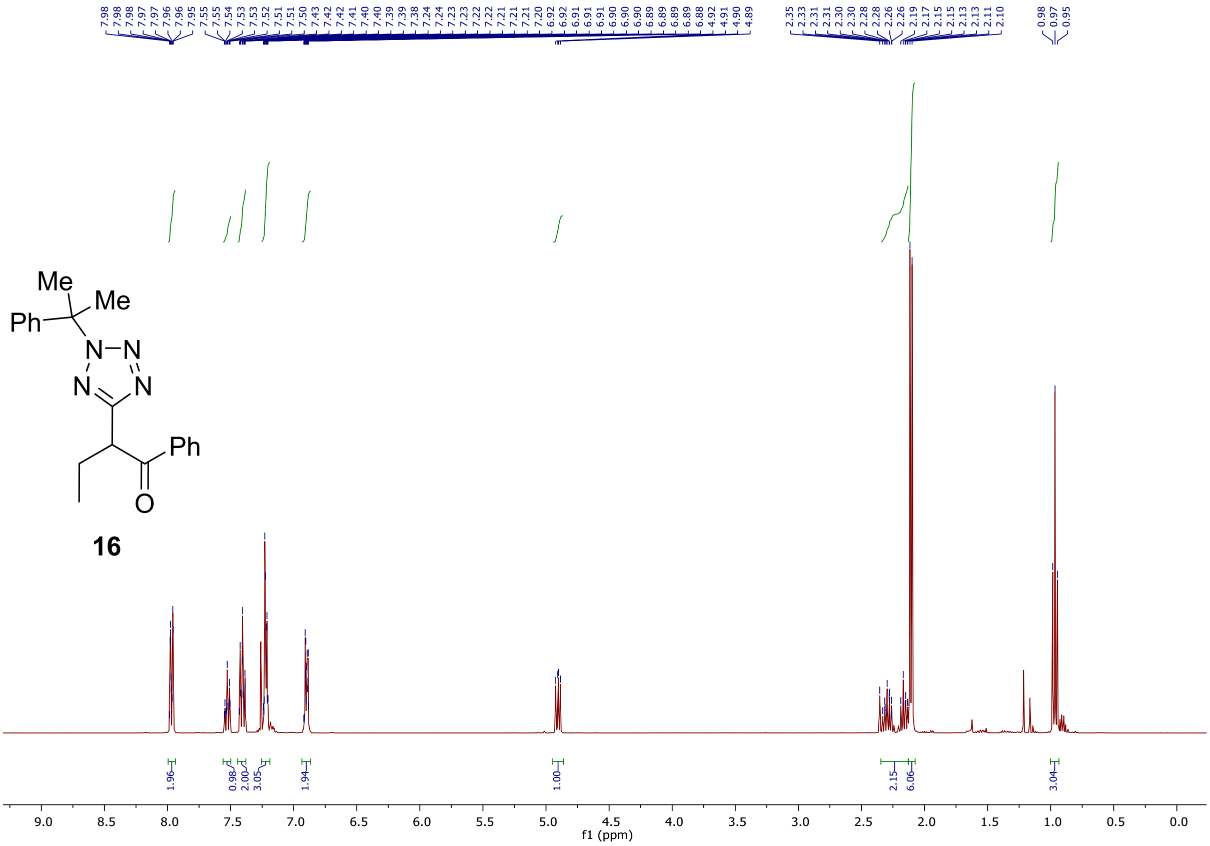
Me
Me
Ph
N
N
N
N
Ph
O
7.98 7.98 7.98 7.97 7.97 7.96 7.96 7.95 7.55 7.54 7.53 7.53 7.52 7.51 7.51 7.50 7.43 7.42 7.42 7.41 7.40 7.40 7.39 7.39 7.38 7.24 7.24 7.23 7.23 7.22 7.22 7.21 7.21 7.21 7.20 6.92 6.92 6.91 6.91 6.90 6.90 6.90 6.89 6.89 6.89 6.88 4.92 4.91 4.90 4.89
2.35 2.33 2.31 2.31 2.30 2.28 2.28 2.26 2.26 2.19 2.17 2.15 2.15 2.13 2.13 2.11 2.10
0.98 0.97 0.95
1.96
0.98
2.00
3.05
1.94
1.00
2.15
6.06
3.04
9.0 8.5 8.0 7.5 7.0 6.5 6.0 5.5 5.0 4.5 4.0 3.5 3.0 2.5 2.0 1.5 1.0 0.5 0.0
f1 (ppm)

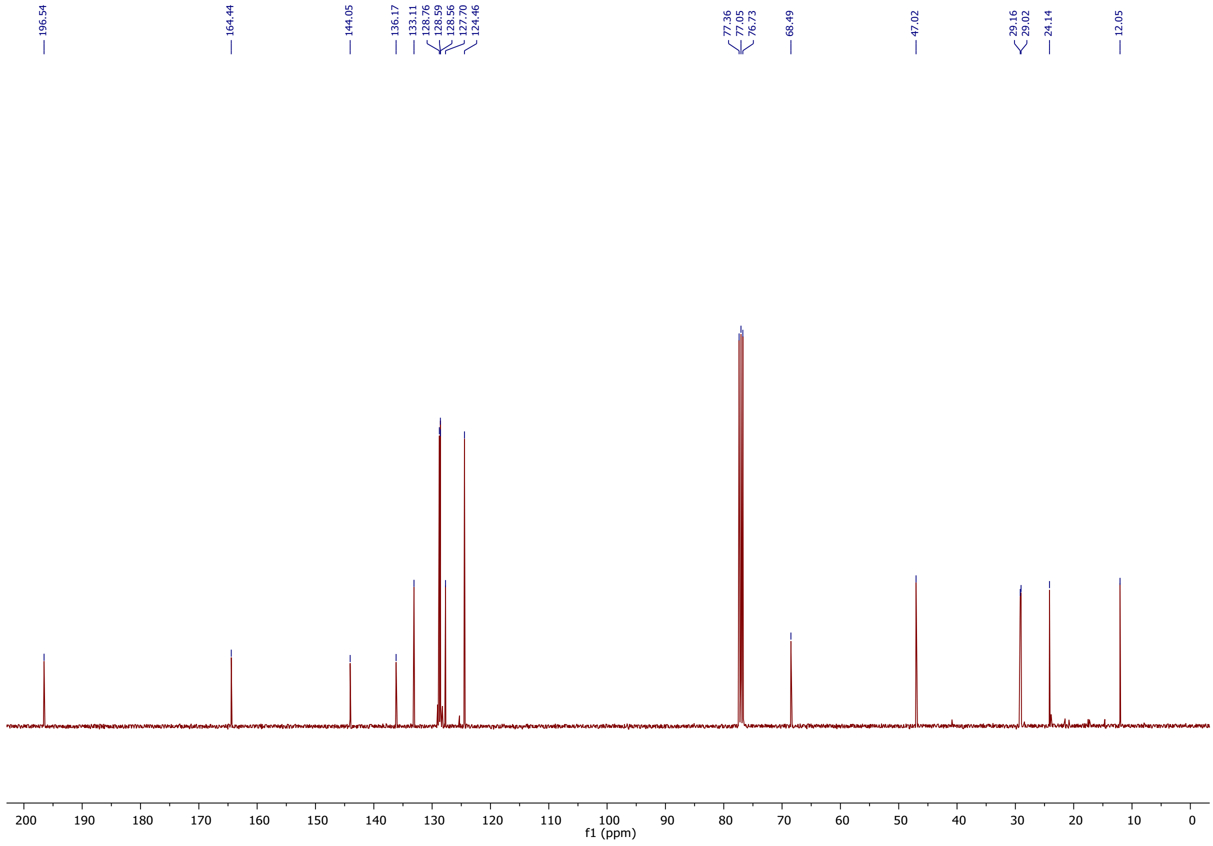
196.54
164.44
144.05
136.17
133.11
128.76
128.59
128.56
127.70
124.46
77.36
77.05
76.73
68.49
47.02
29.16
29.02
24.14
12.05
f1 (ppm)

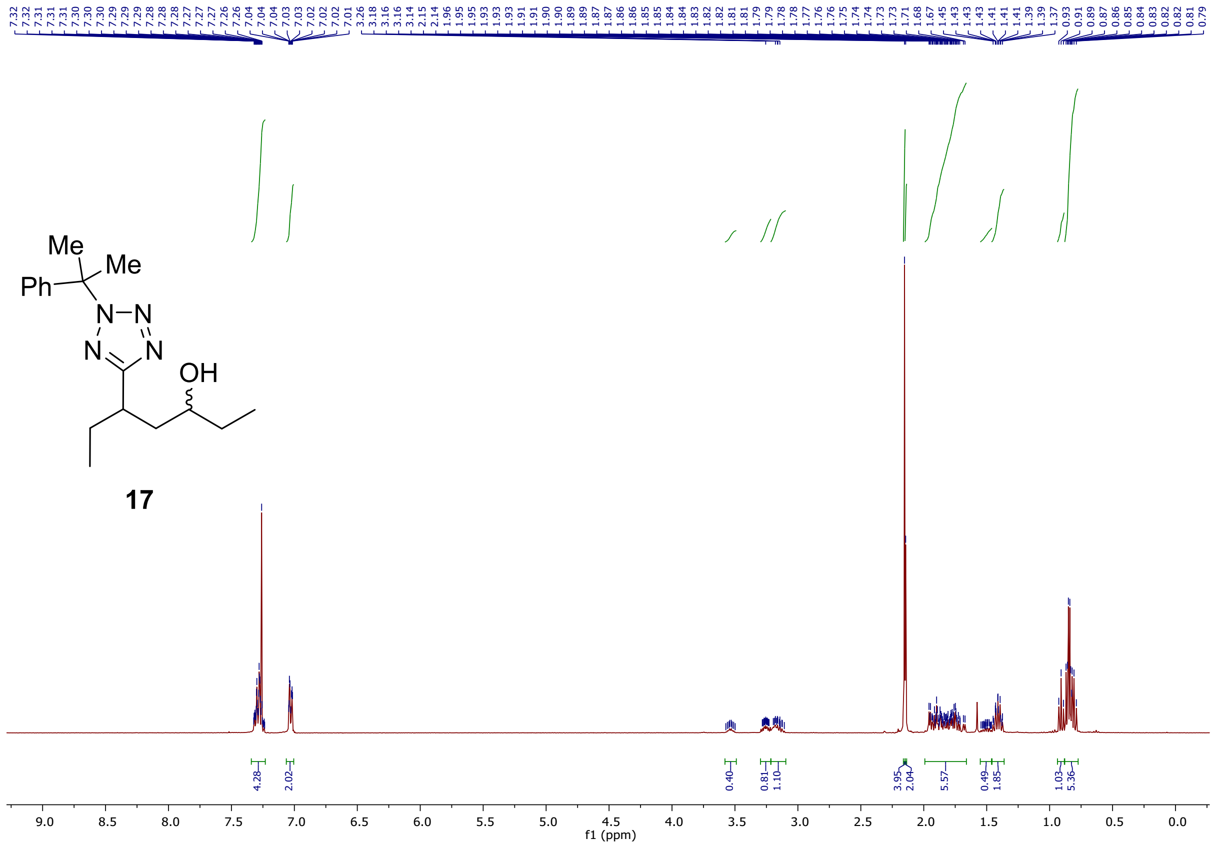
Me
Me
Ph
N
N
N
N
OH
17
4.28
2.02
0.40
0.81
1.10
3.95
2.04
5.57
0.49
1.85
1.03
5.36
f1 (ppm)

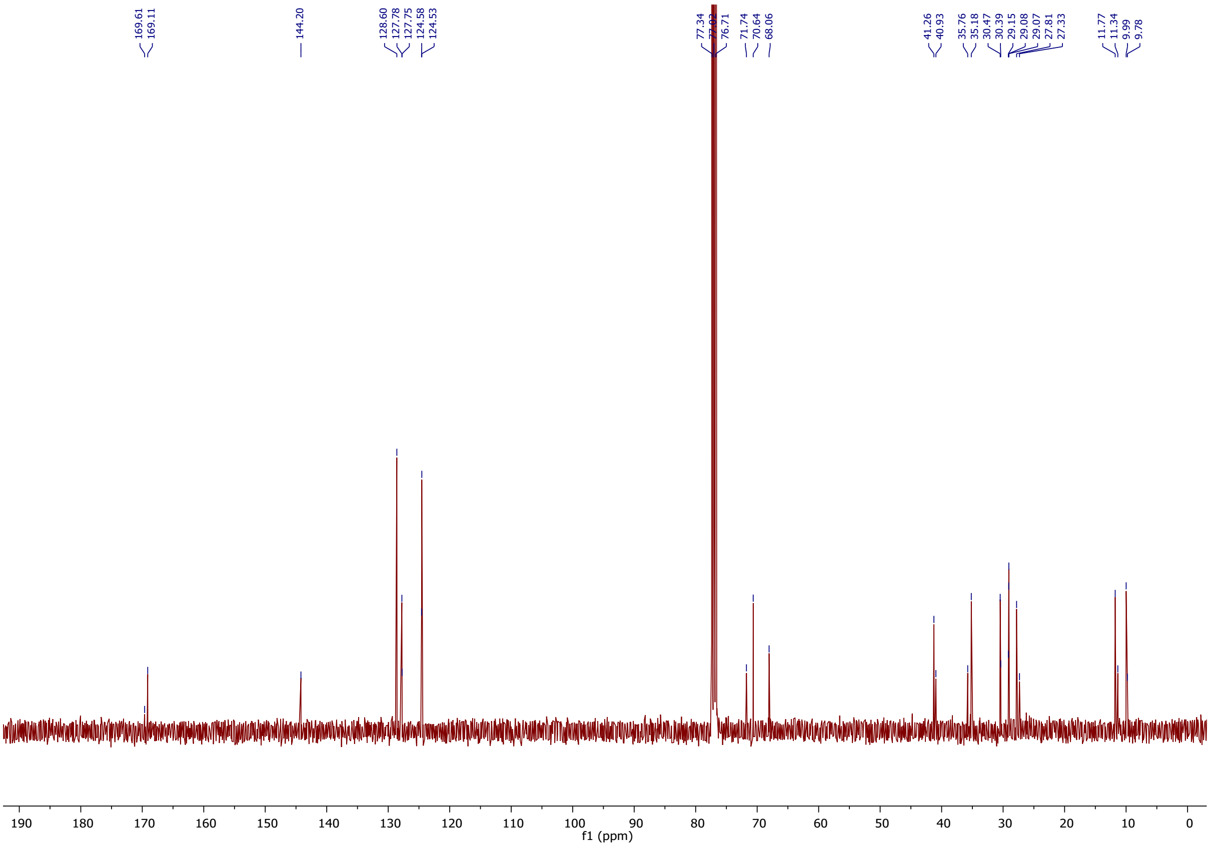
169.61
169.11
144.20
128.60
127.78
127.75
124.58
124.53
77.34
76.71
71.74
70.64
68.06
41.26
40.93
35.76
35.18
30.47
30.39
29.15
29.08
29.07
27.81
27.33
11.77
11.34
9.99
9.78
f1 (ppm)

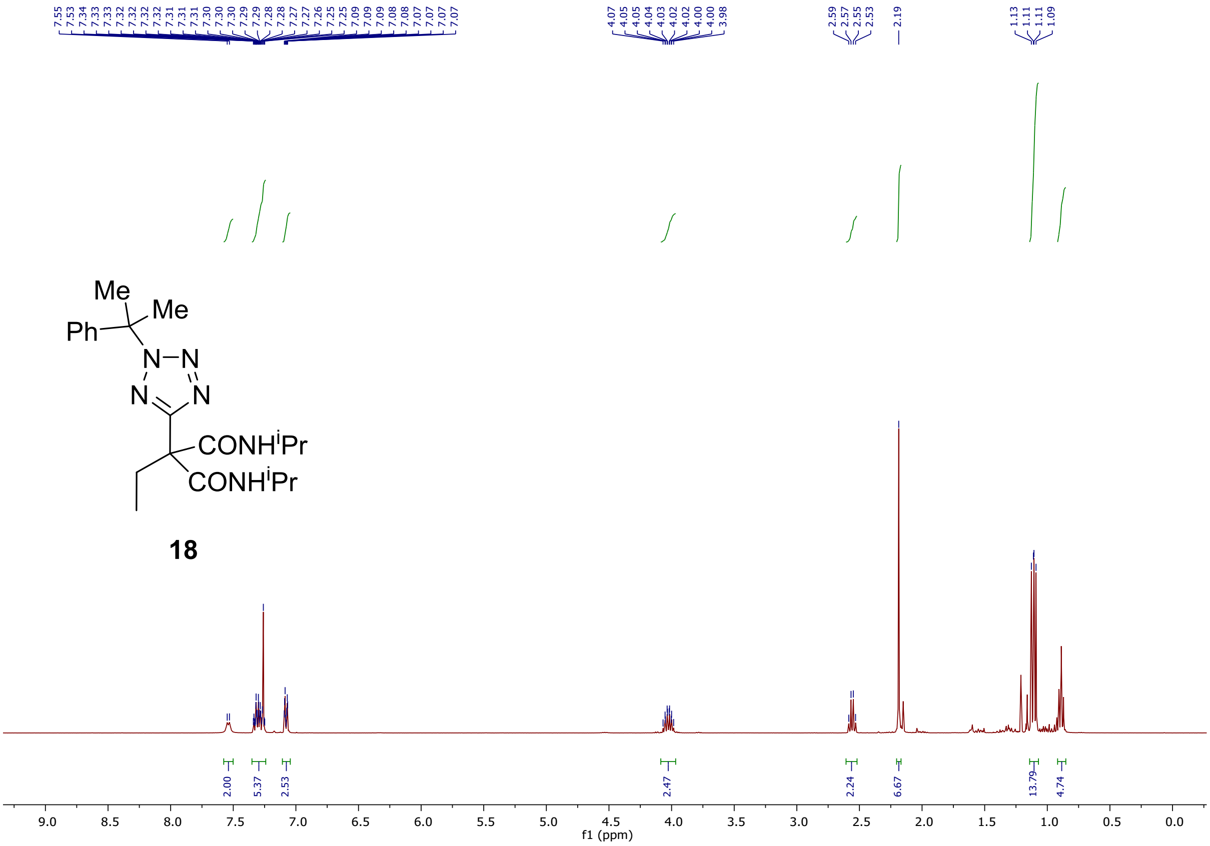
Me
Me
Ph
N
N
N
N
CONHiPr
CONHiPr

18

4.07
4.05
4.04
4.03
4.02
4.02
4.00
4.00
3.98
2.59
2.57
2.55
2.53
2.19
1.13
1.11
1.11
1.09
2.00
5.37
2.53
2.47
2.24
6.67
13.79
4.74
f1 (ppm)

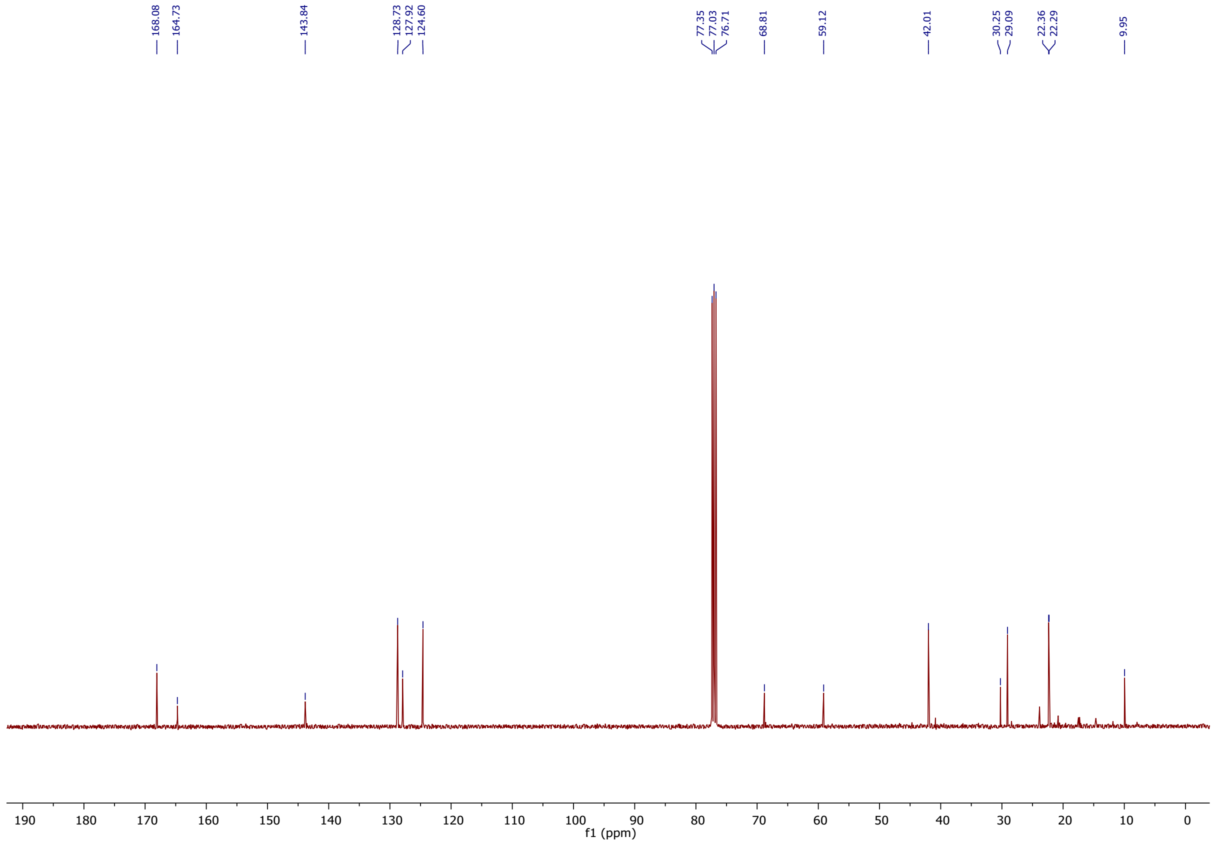
168.08
164.73
143.84
128.73
127.92
124.60
77.35
77.03
76.71
68.81
59.12
42.01
30.25
29.09
22.36
22.29
9.95
f1 (ppm)

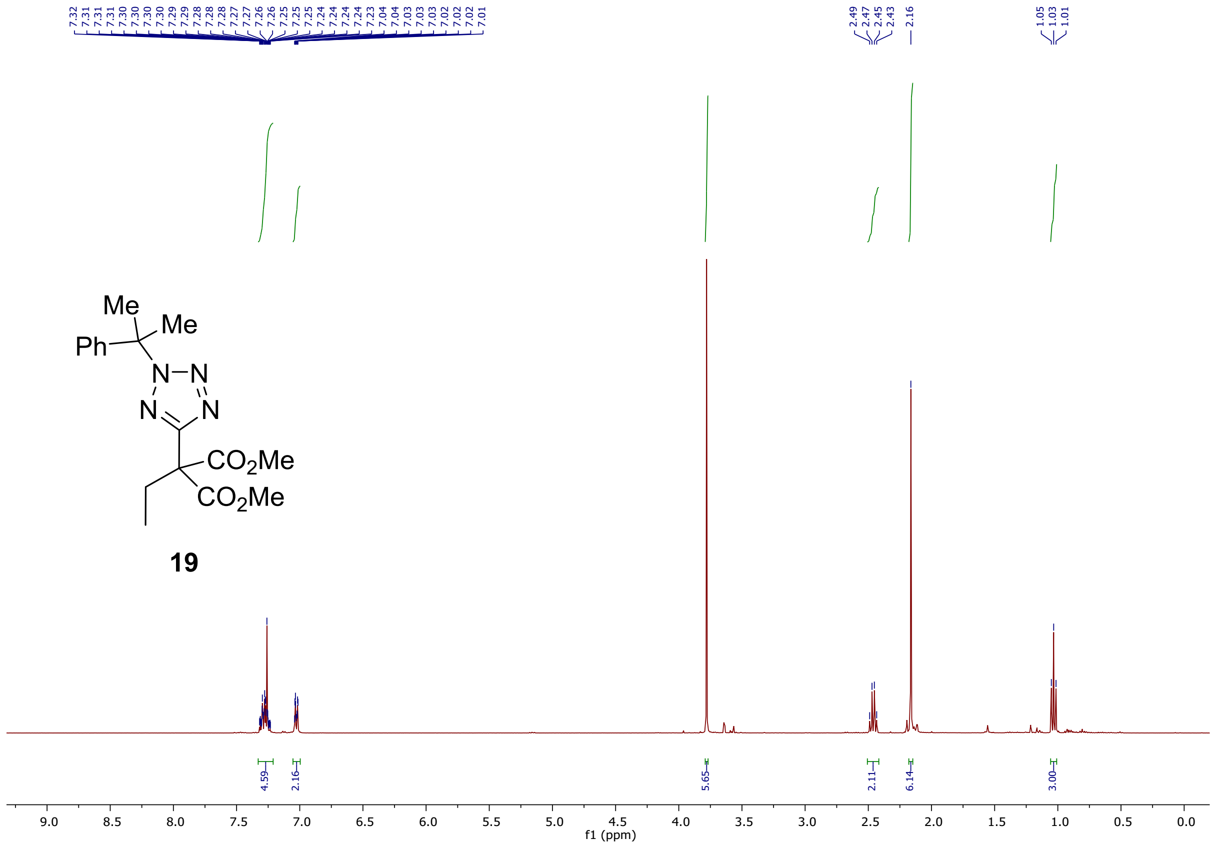
Me
Me
Ph
N
N
N
N
CO_2Me
CO_2Me
19
7.32
7.31
7.31
7.30
7.30
7.30
7.29
7.29
7.28
7.28
7.28
7.27
7.27
7.26
7.26
7.25
7.25
7.25
7.24
7.24
7.24
7.23
7.04
7.04
7.03
7.03
7.02
7.02
7.02
7.01
2.49
2.47
2.45
2.43
2.16
1.05
1.03
1.01
4.59
2.16
5.65
2.11
6.14
3.00
f1 (ppm)

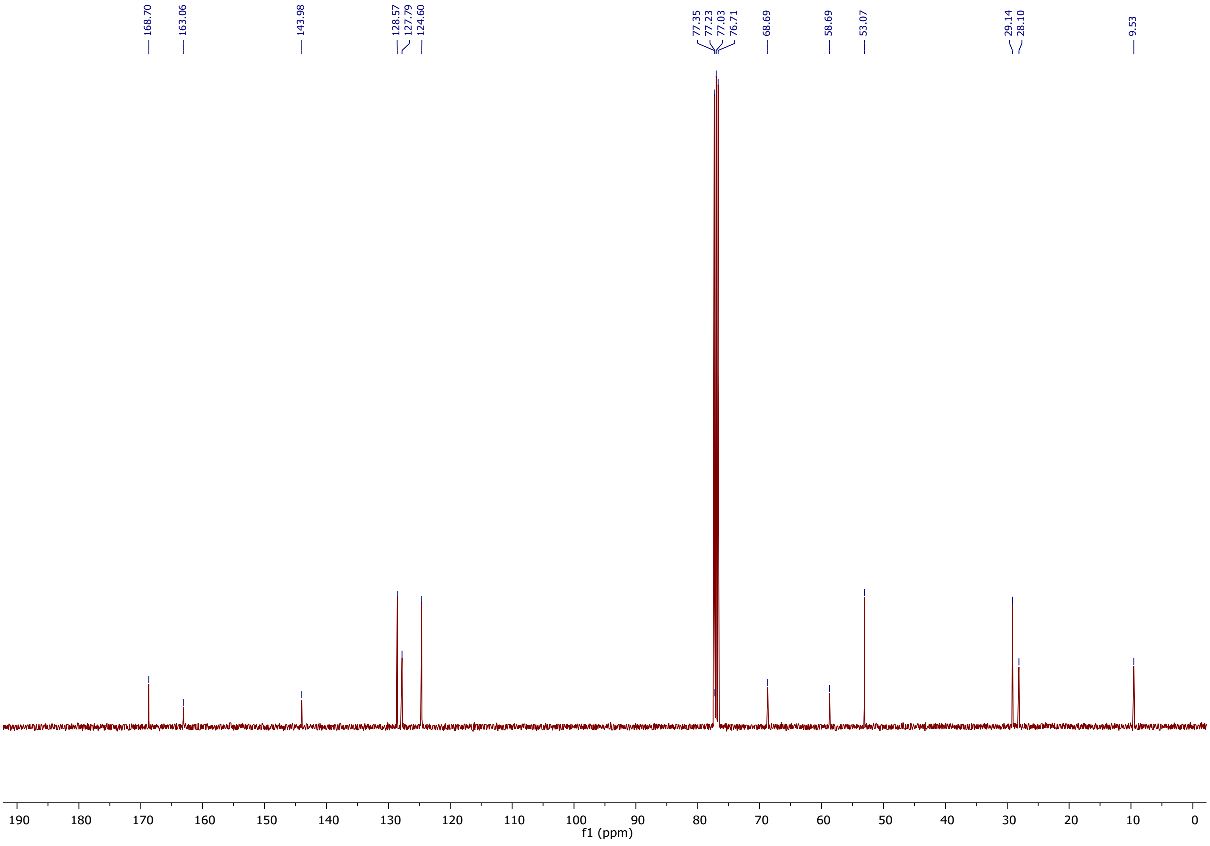

168.70
163.06
143.98
128.57
127.79
124.60
77.35
77.23
77.03
76.71
68.69
58.69
53.07
29.14
28.10
9.53
f1 (ppm)

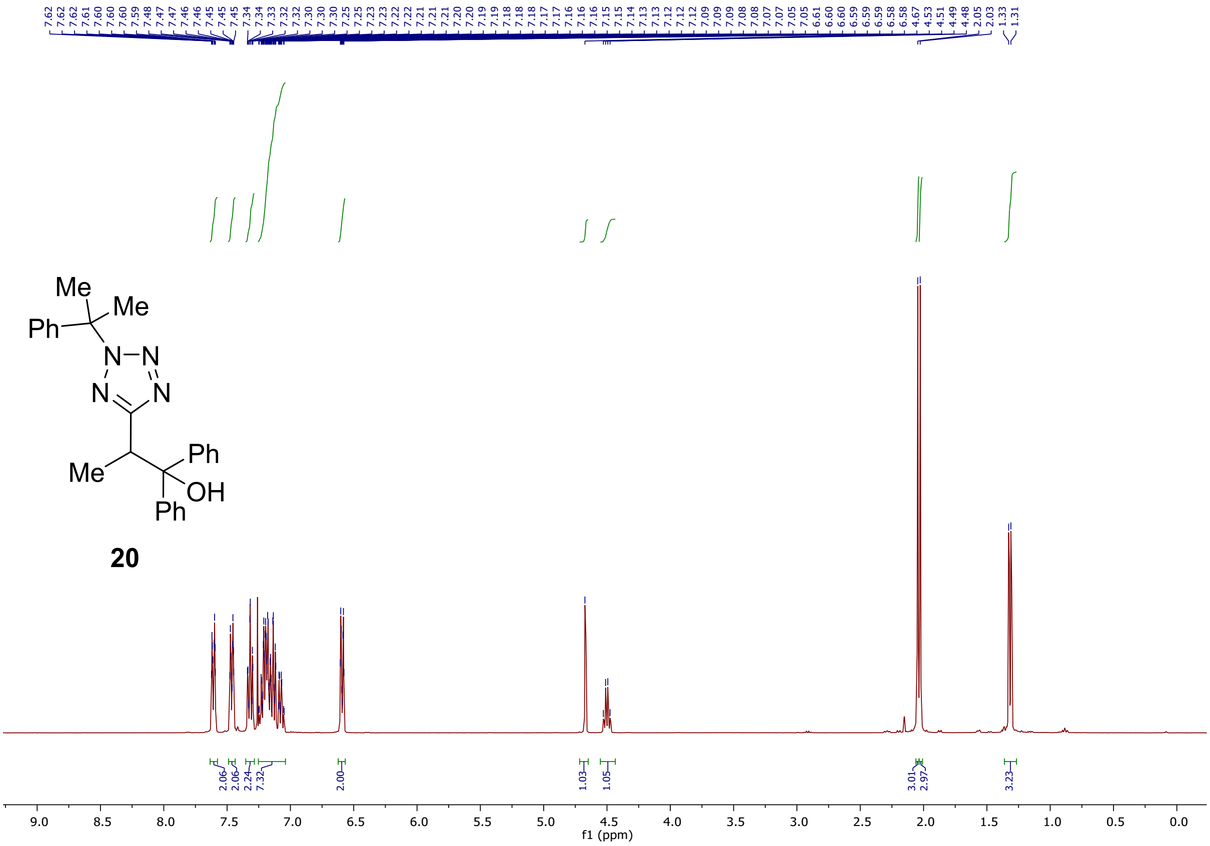

7.62 7.62 7.61 7.60 7.60 7.59 7.48 7.47 7.47 7.46 7.45 7.45 7.45 7.34 7.34 7.33 7.32 7.32 7.30 7.30 7.30 7.25 7.25 7.23 7.23 7.22 7.22 7.21 7.21 7.21 7.20 7.20 7.19 7.19 7.18 7.18 7.18 7.17 7.17 7.16 7.16 7.15 7.15 7.14 7.13 7.13 7.12 7.12 7.12 7.09 7.09 7.09 7.08 7.08 7.07 7.07 7.05 7.05 6.61 6.60 6.59 6.59 6.59 6.58 6.58 4.67 4.53 4.51 4.49 4.48 2.05 2.03 1.33 1.31
2.06
2.06
2.24
7.32
2.00
1.03
1.05
3.01
2.97
3.23
f1 (ppm)
Me
Me
Ph
N
N
N
N
Me
Ph
OH
Ph

20

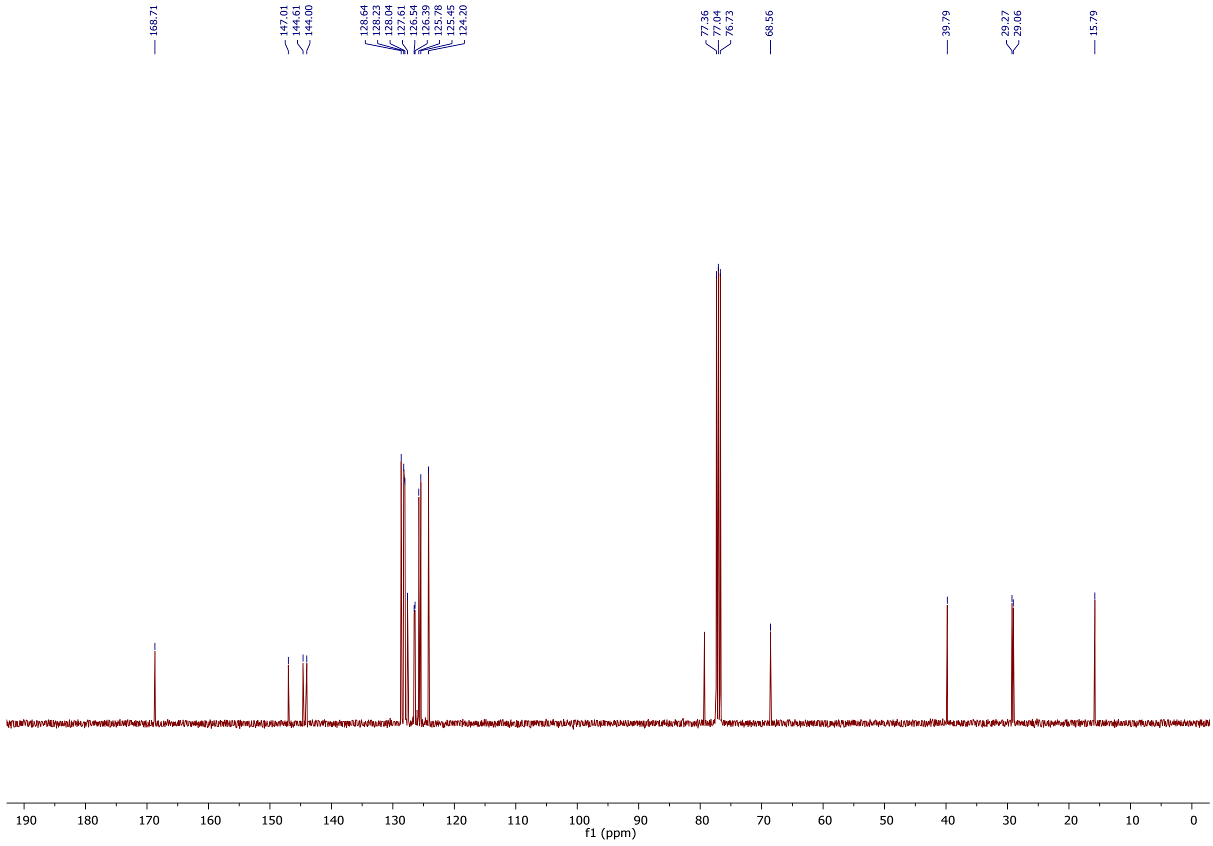

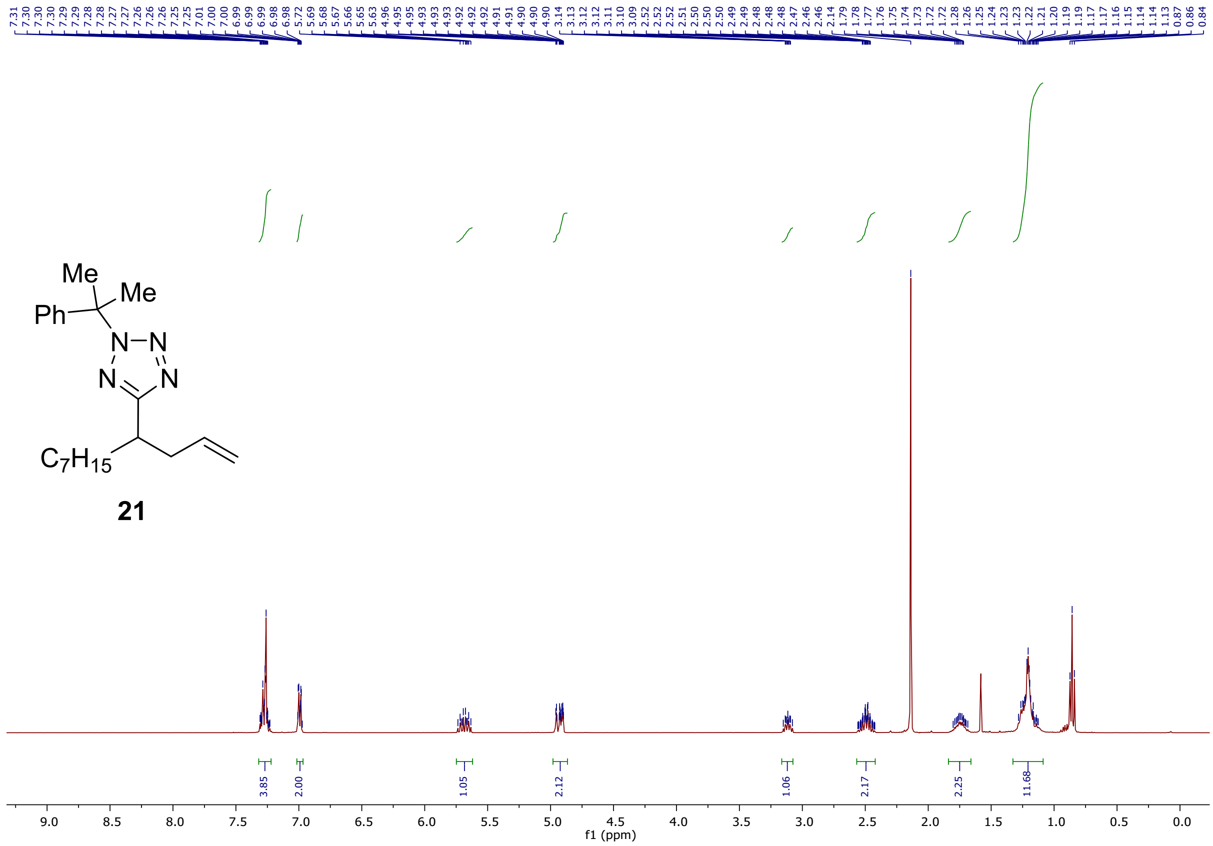

21

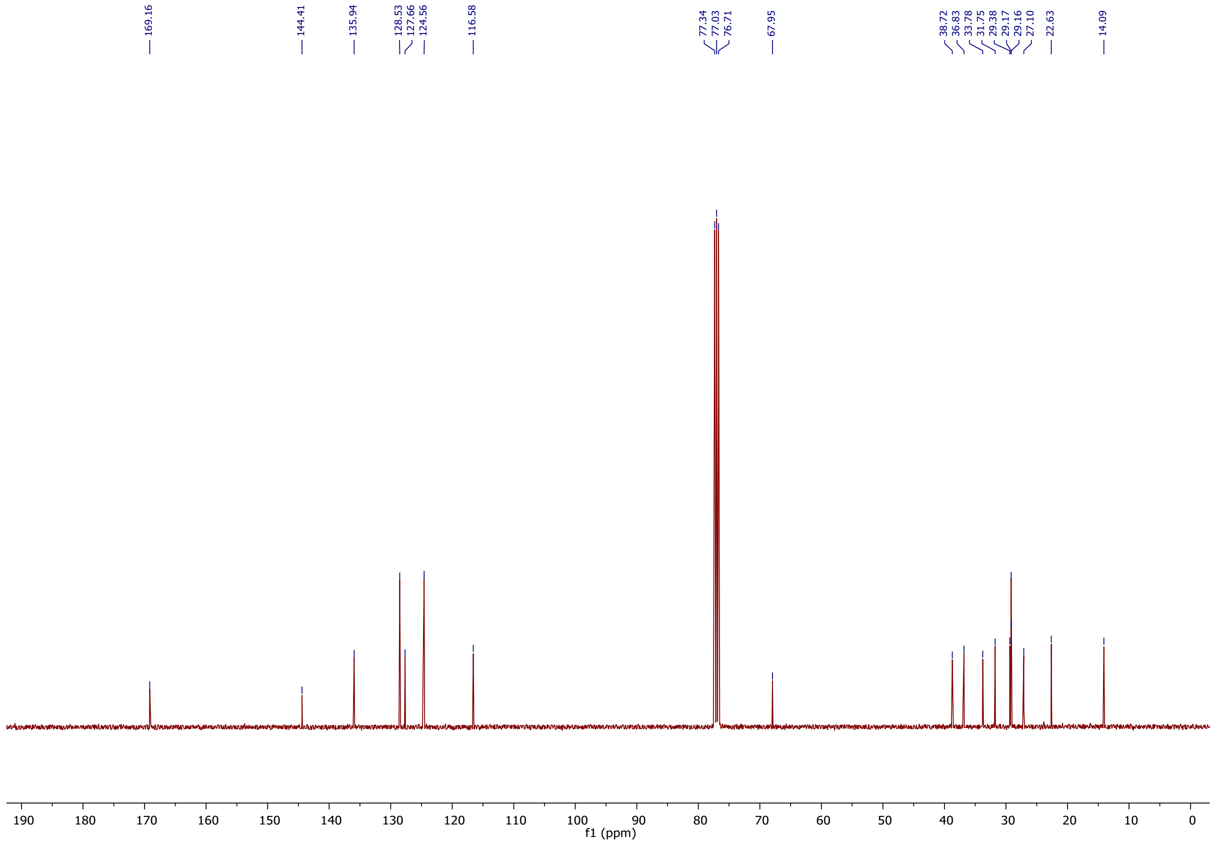
169.16
144.41
135.94
128.53
127.66
124.56
116.58
77.34
77.03
76.71
67.95
38.72
36.83
33.78
31.75
29.38
29.17
29.16
27.10
22.63
14.09
f1 (ppm)

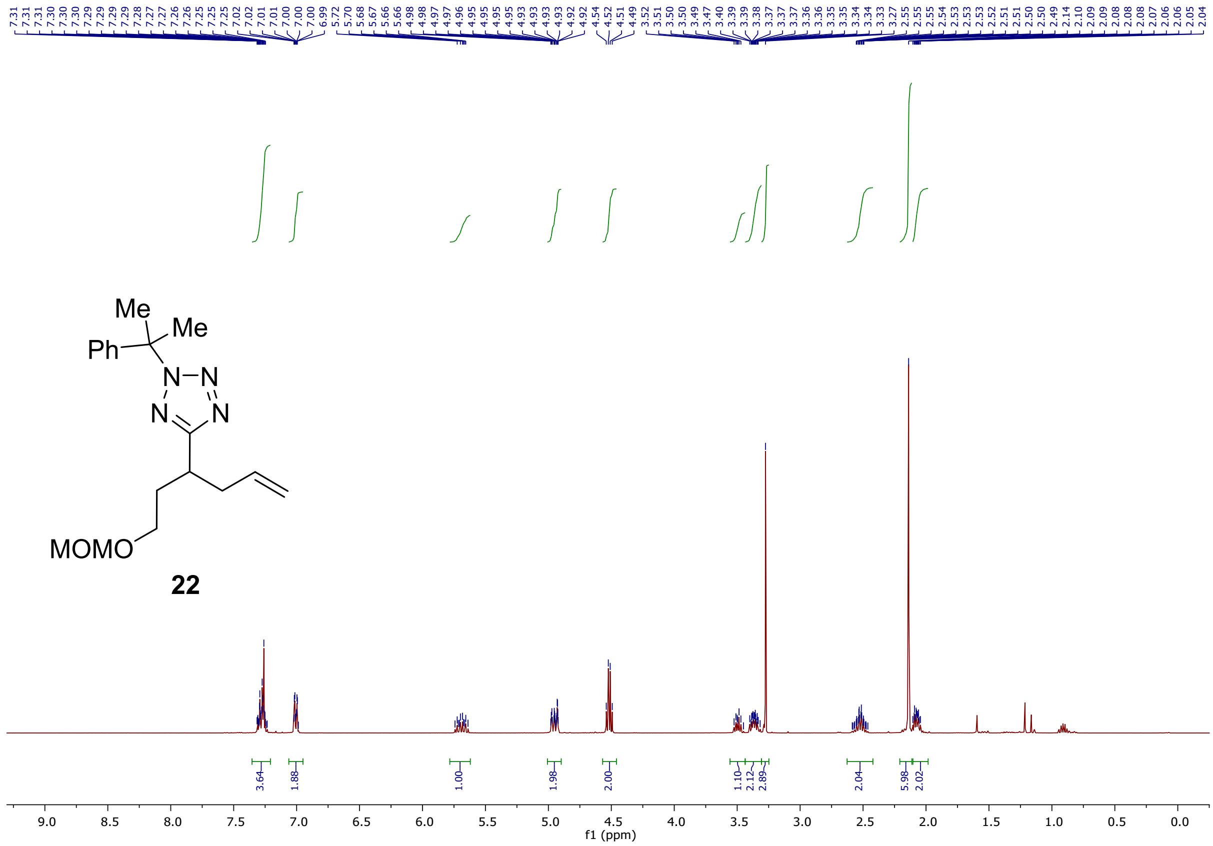
Me
Me
Ph
N
N
N
N
MOMO
22
3.64
1.88
1.00
1.98
2.00
1.10
2.12
2.89
2.04
5.98
2.02
f1 (ppm)

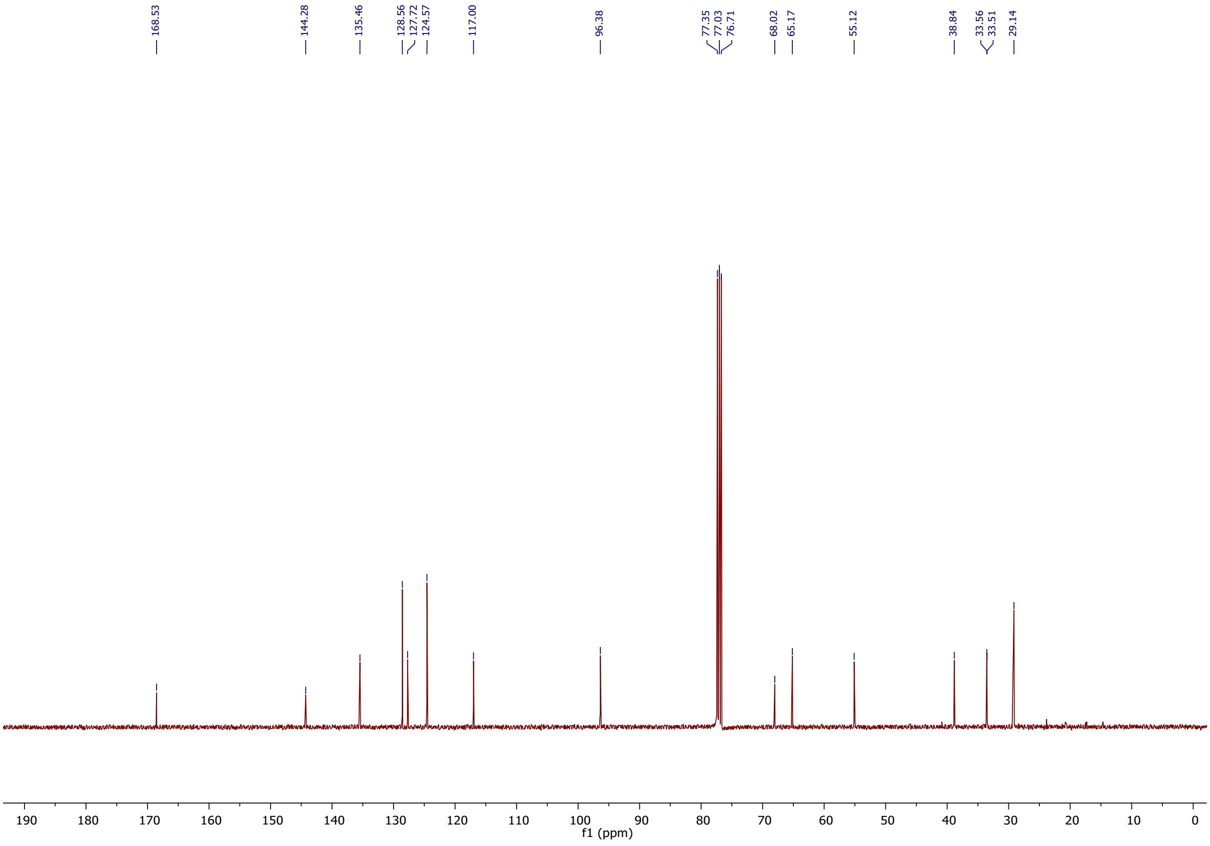
168.53
144.28
135.46
128.56
127.72
124.57
117.00
96.38
77.35
77.03
76.71
68.02
65.17
55.12
38.84
33.56
33.51
29.14
190 180 170 160 150 140 130 120 110 100 90 80 70 60 50 40 30 20 10 0
f1 (ppm)

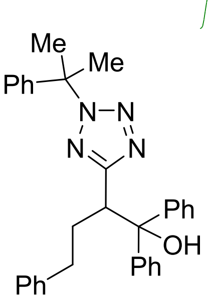
Me
Me
Ph
N
N
N
N
Ph
OH
Ph
Ph

23

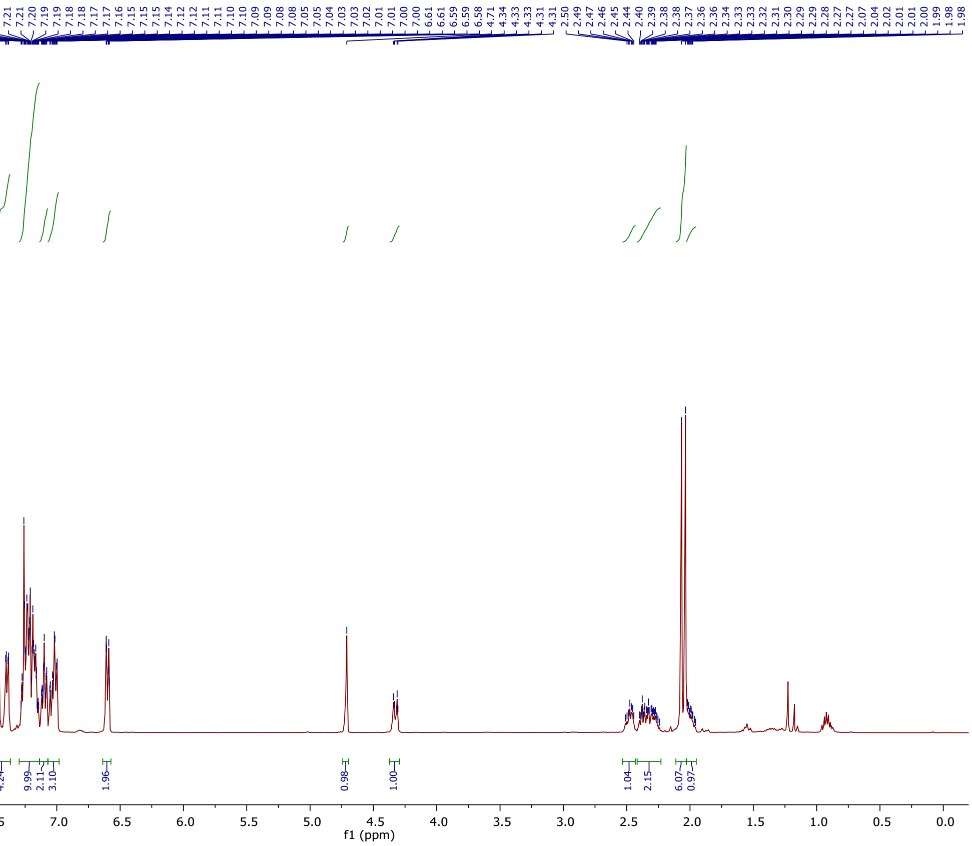
7.48 7.47 7.46 7.45 7.40 7.39 7.38 7.28 7.27 7.26 7.25 7.24 7.23 7.22 7.21 7.20 7.19 7.18 7.17 7.16 7.15 7.15 7.14 7.12 7.11 7.11 7.10 7.09 7.09 7.08 7.05 7.04 7.03 7.02 7.01 7.01 7.00 7.00 6.61 6.61 6.59 6.58 4.71 4.34 4.33 4.31 2.50 2.49 2.47 2.46 2.45 2.44 2.40 2.39 2.38 2.38 2.37 2.36 2.36 2.34 2.33 2.33 2.32 2.31 2.30 2.29 2.29 2.28 2.27 2.27 2.07 2.04 2.02 2.01 2.00 1.99 1.98 1.98
4.24
9.99
2.11
3.10
1.96
0.98
1.00
1.04
2.15
6.07
0.97
9.0 8.5 8.0 7.5 7.0 6.5 6.0 5.5 5.0 4.5 4.0 3.5 3.0 2.5 2.0 1.5 1.0 0.5 0.0
f1 (ppm)

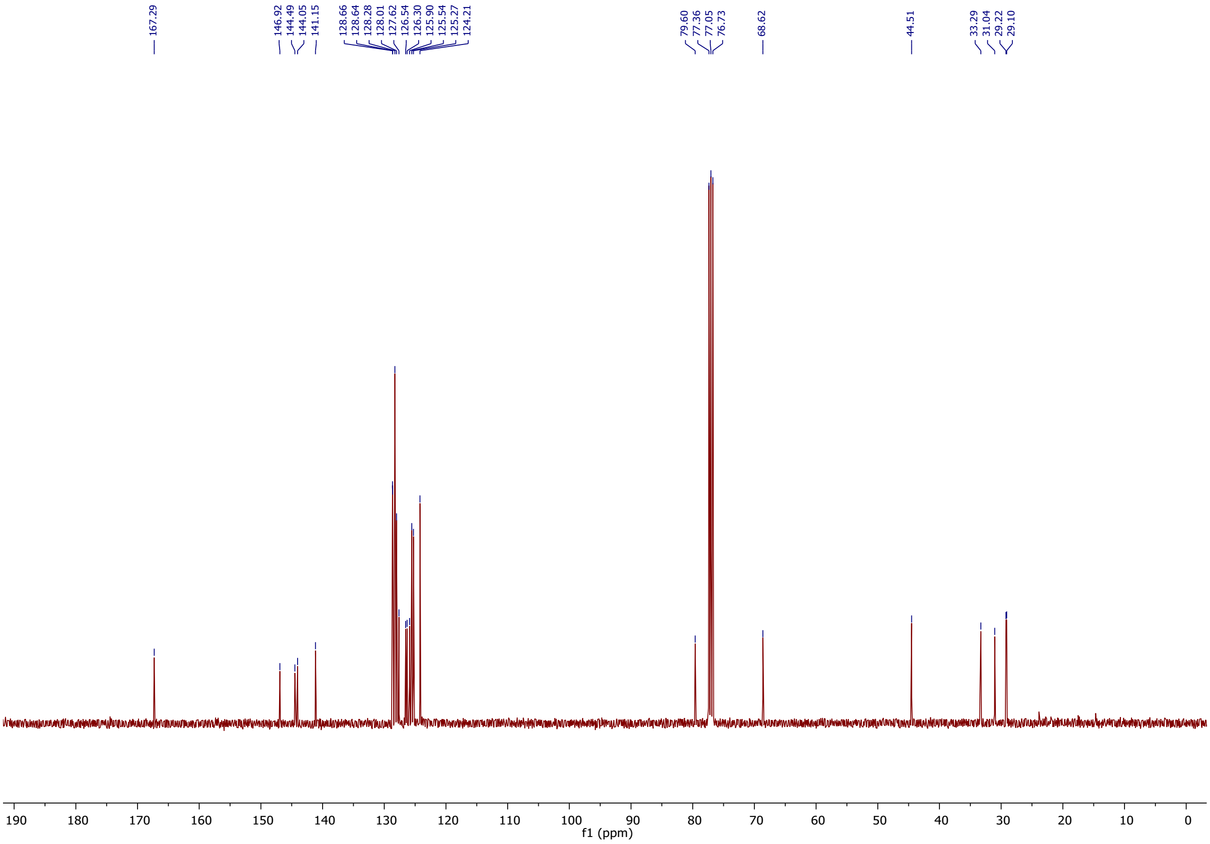

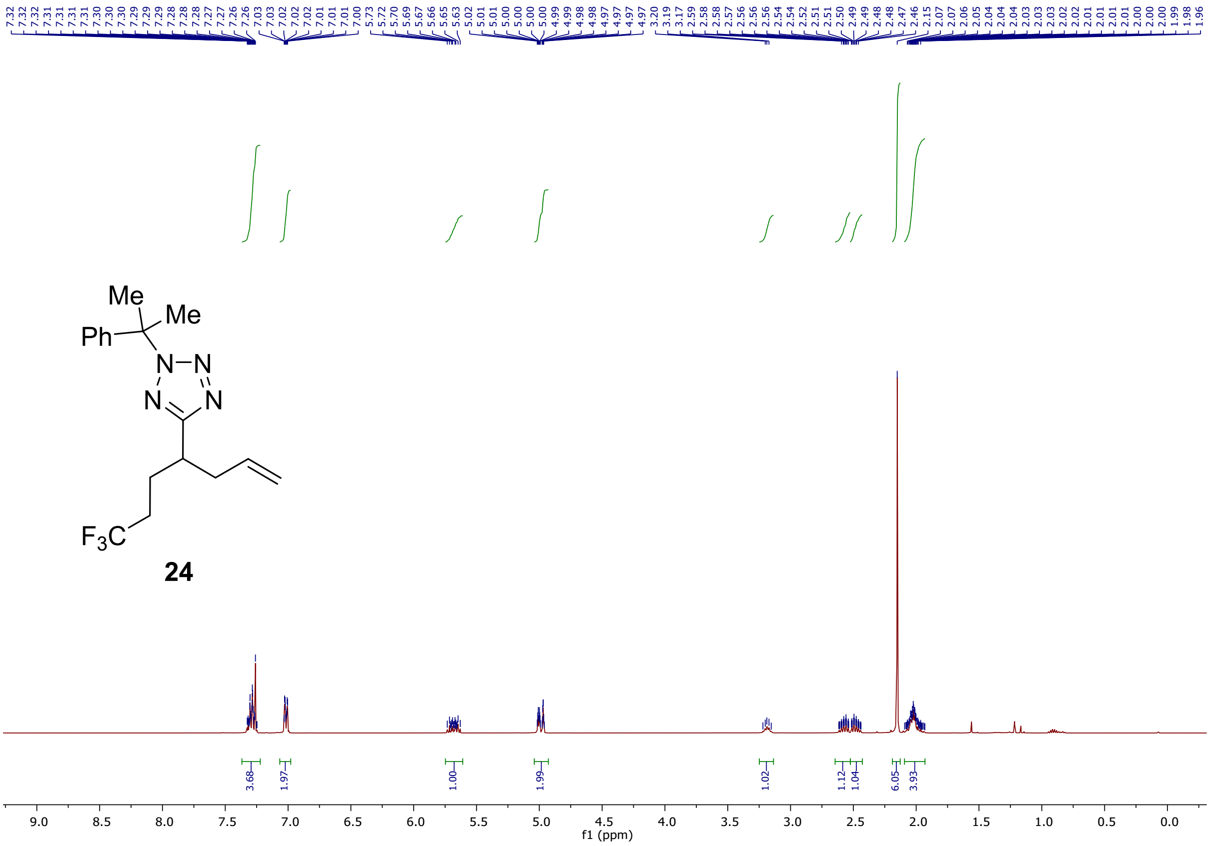

24

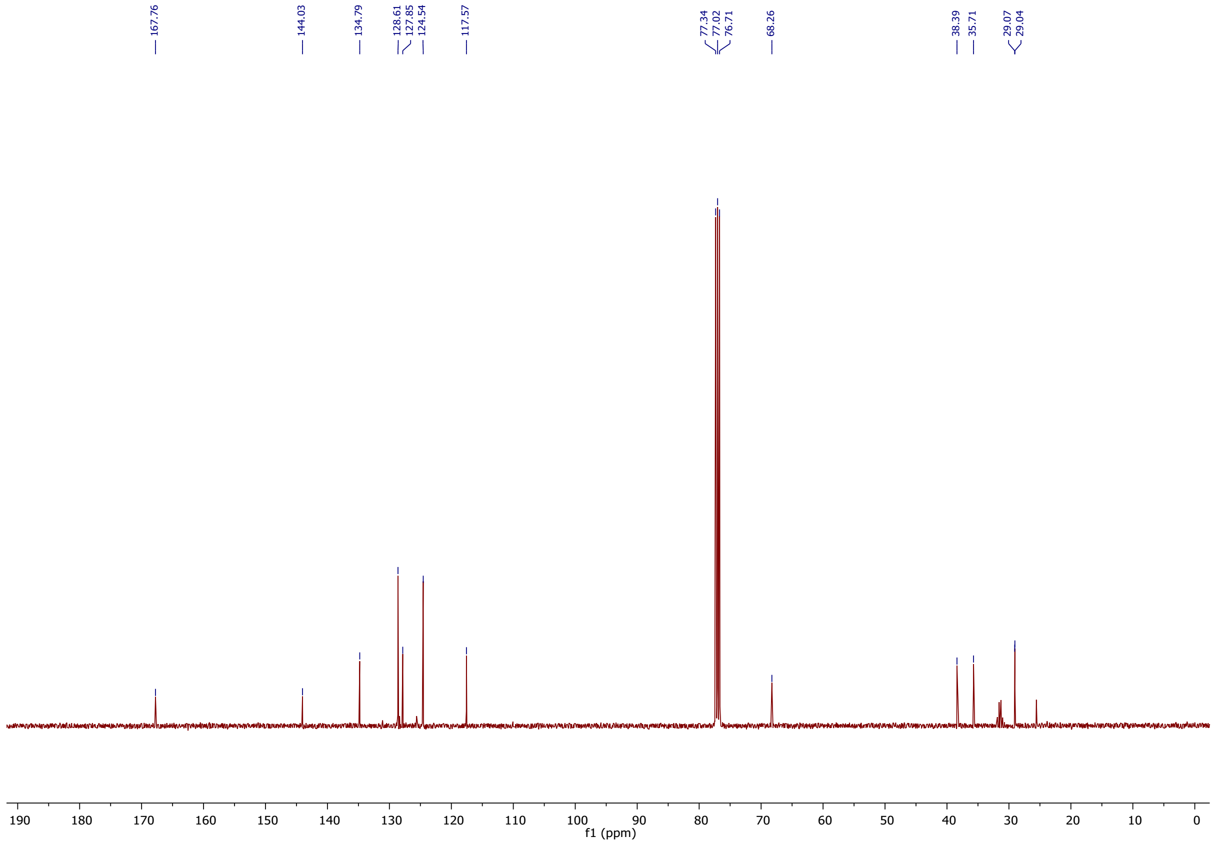
167.76
144.03
134.79
128.61
127.85
124.54
117.57
77.34
77.02
76.71
68.26
38.39
35.71
29.07
29.04
f1 (ppm)

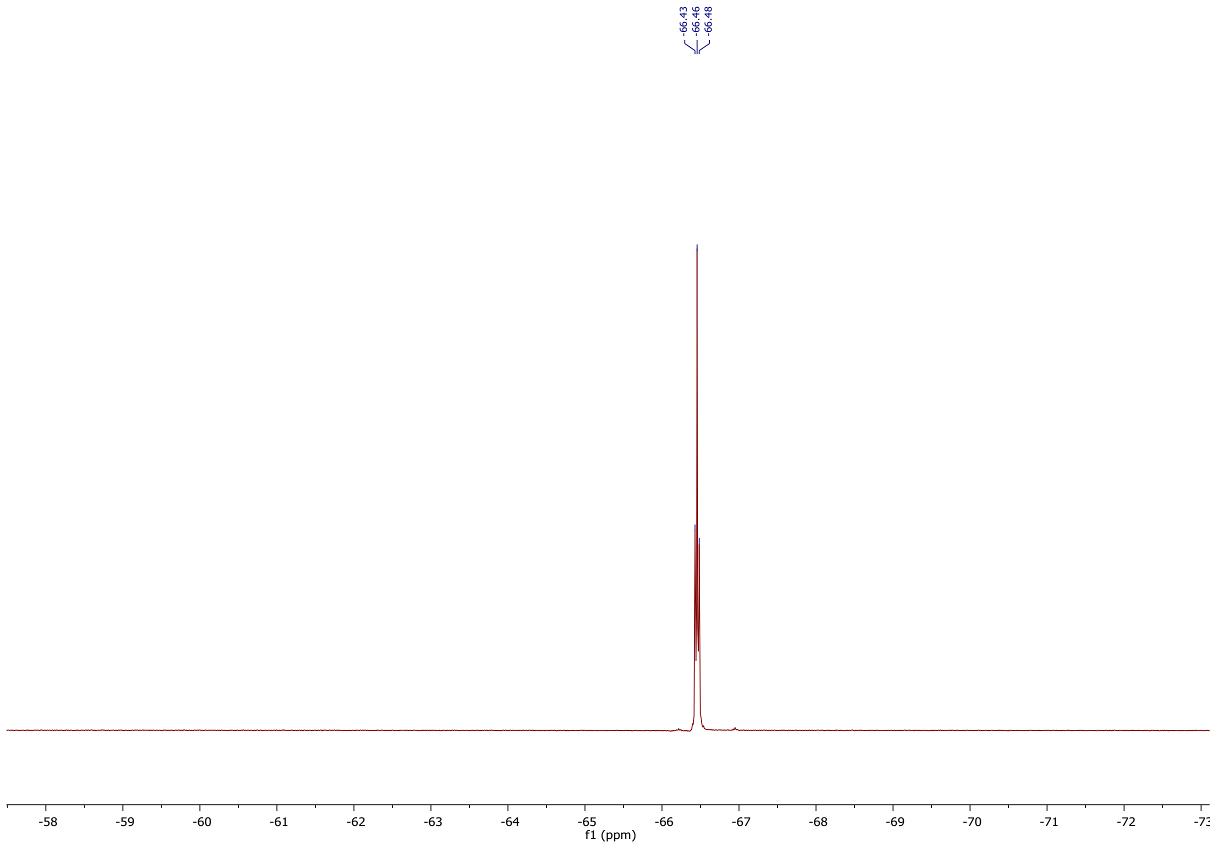
-66.43
-66.46
-66.48
f1 (ppm)

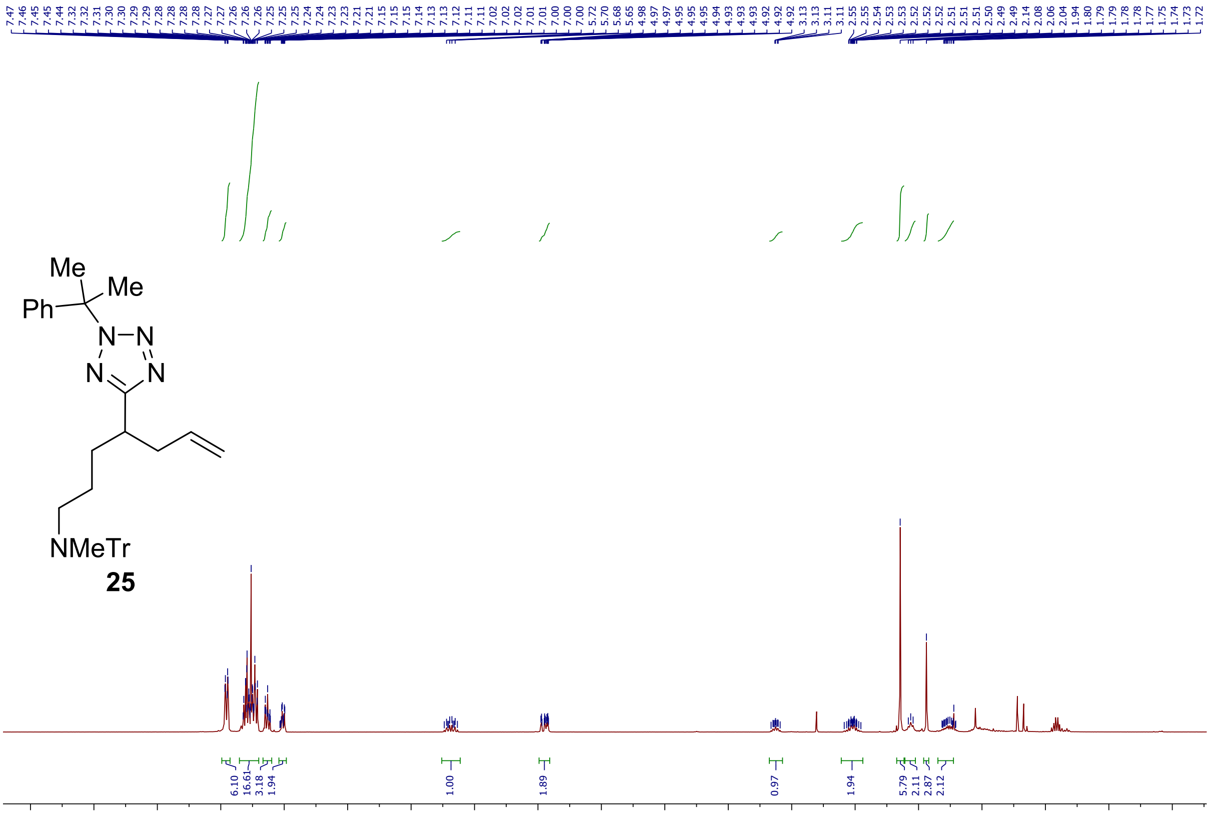
Me
Me
Ph
N
N
N
N
NMeTr
25
f1 (ppm)

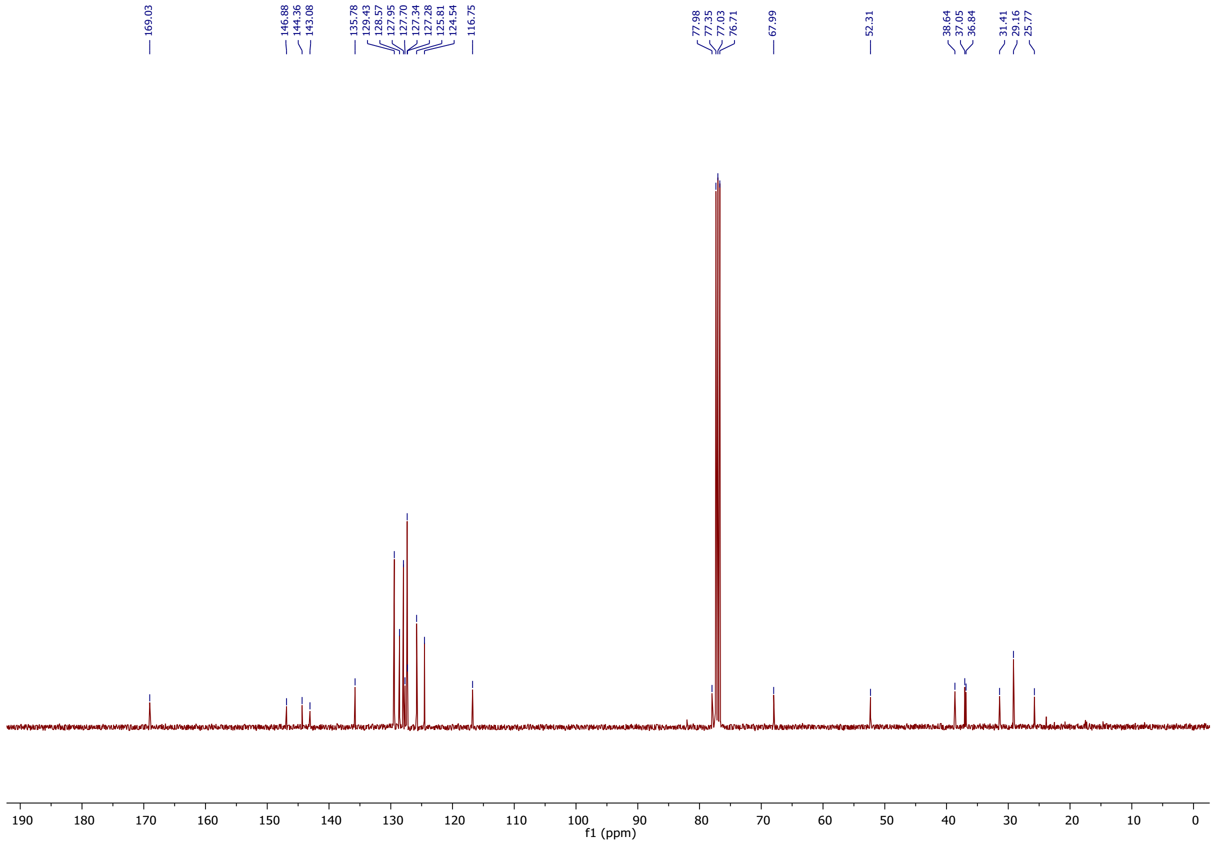
169.03
146.88
144.36
143.08
135.78
129.43
128.57
127.95
127.70
127.34
127.28
125.81
124.54
116.75
77.98
77.35
77.03
76.71
67.99
52.31
38.64
37.05
36.84
31.41
29.16
25.77
190 180 170 160 150 140 130 120 110 100 90 80 70 60 50 40 30 20 10 0
f1 (ppm)

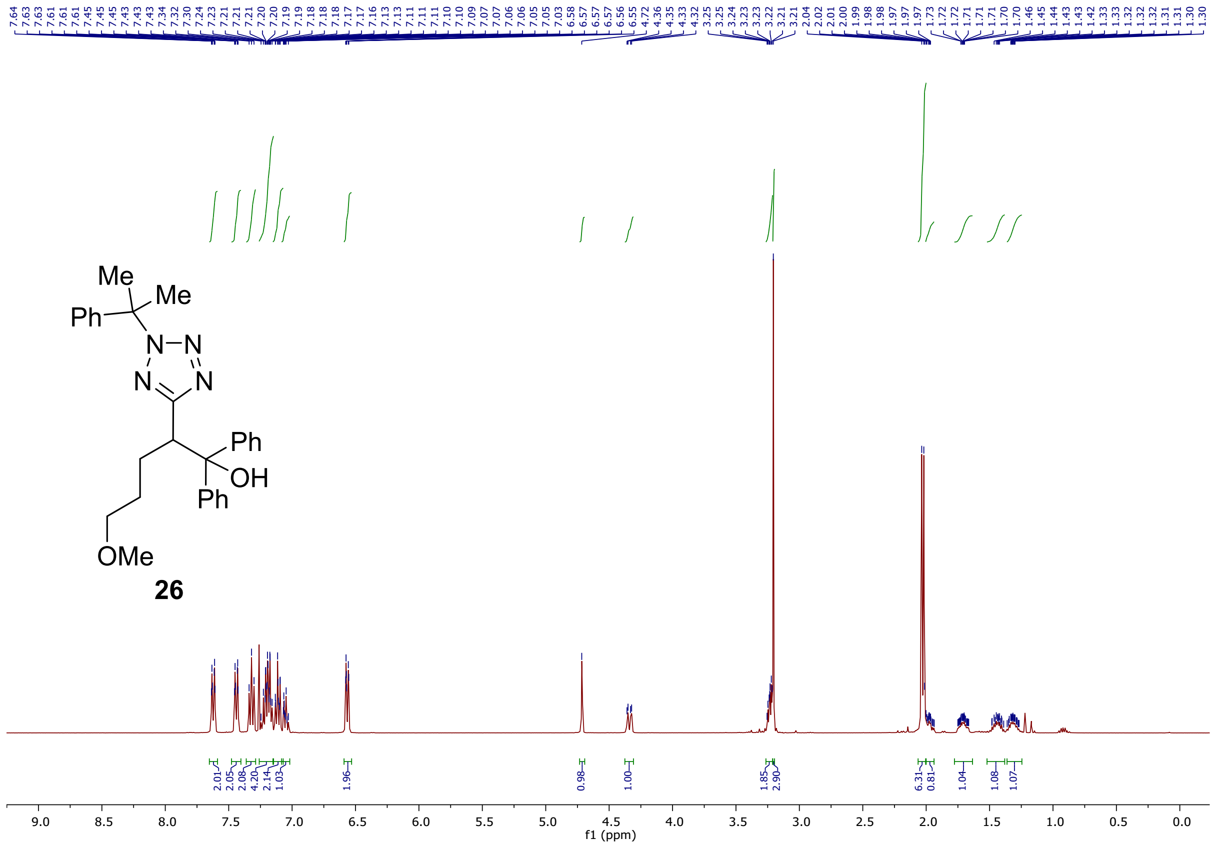
7.64 7.63 7.63 7.61 7.61 7.45 7.45 7.45 7.43 7.43 7.43 7.34 7.32 7.30 7.24 7.23 7.21 7.21 7.21 7.20 7.20 7.19 7.19 7.18 7.18 7.18 7.17 7.17 7.16 7.13 7.13 7.11 7.11 7.10 7.10 7.09 7.07 7.06 7.06 7.05 7.05 7.03 6.58 6.57 6.57 6.56 6.55 4.72 4.36 4.35 4.33 4.32 3.25 3.24 3.23 3.23 3.22 3.21 3.21 2.04 2.02 2.01 2.00 1.99 1.98 1.98 1.97 1.97 1.73 1.72 1.72 1.71 1.71 1.71 1.70 1.46 1.45 1.44 1.43 1.42 1.33 1.33 1.33 1.32 1.32 1.31 1.31 1.30 1.30
2.01 2.05 2.08 4.20 2.14 1.03 1.96 0.98 1.00 1.85 2.90 6.31 0.81 1.04 1.08 1.07
9.0 8.5 8.0 7.5 7.0 6.5 6.0 5.5 5.0 4.5 4.0 3.5 3.0 2.5 2.0 1.5 1.0 0.5 0.0
f1 (ppm)
Me
Me
Ph
N
N
N
N
Ph
OH
Ph
OMe

26

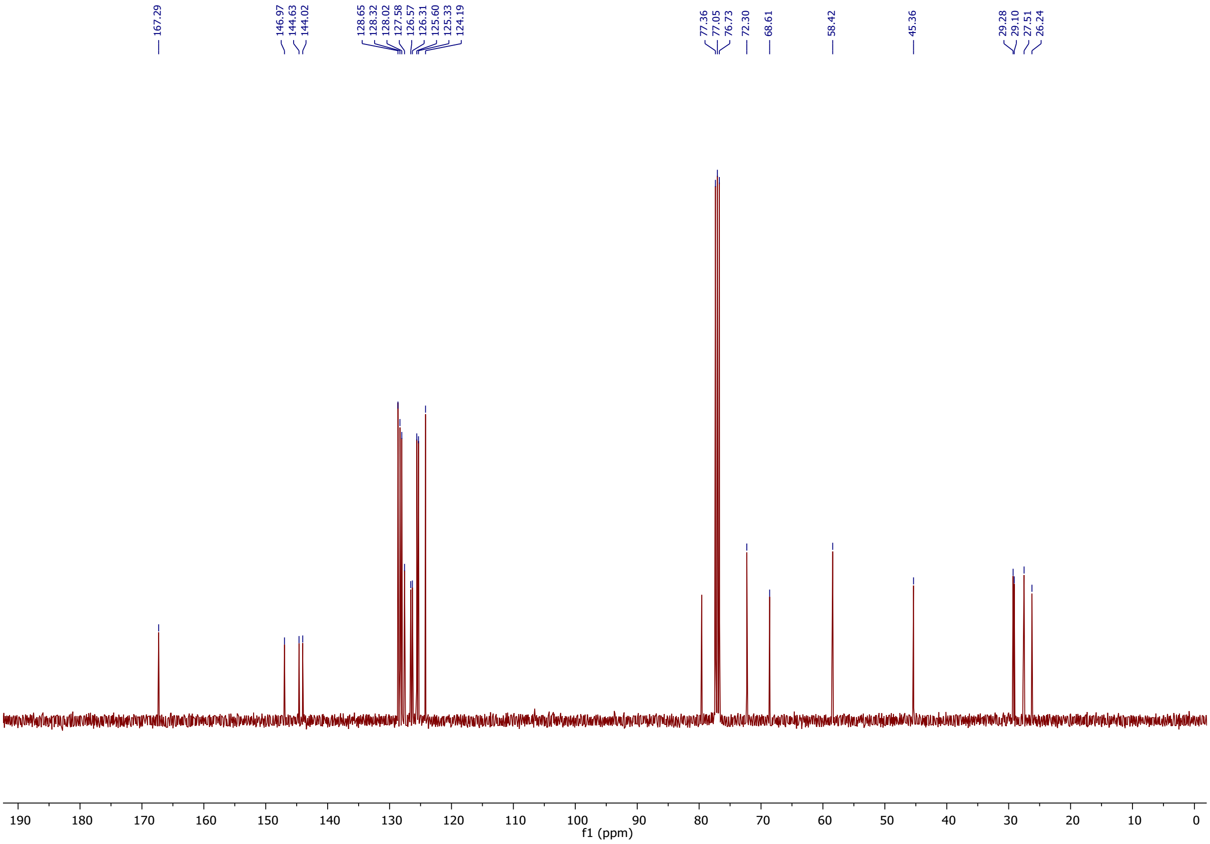
167.29
146.97
144.63
144.02
128.65
128.32
128.02
127.58
126.57
126.31
125.60
125.33
124.19
77.36
77.05
76.73
72.30
68.61
58.42
45.36
29.28
29.10
27.51
26.24
190 180 170 160 150 140 130 120 110 100 90 80 70 60 50 40 30 20 10 0
f1 (ppm)

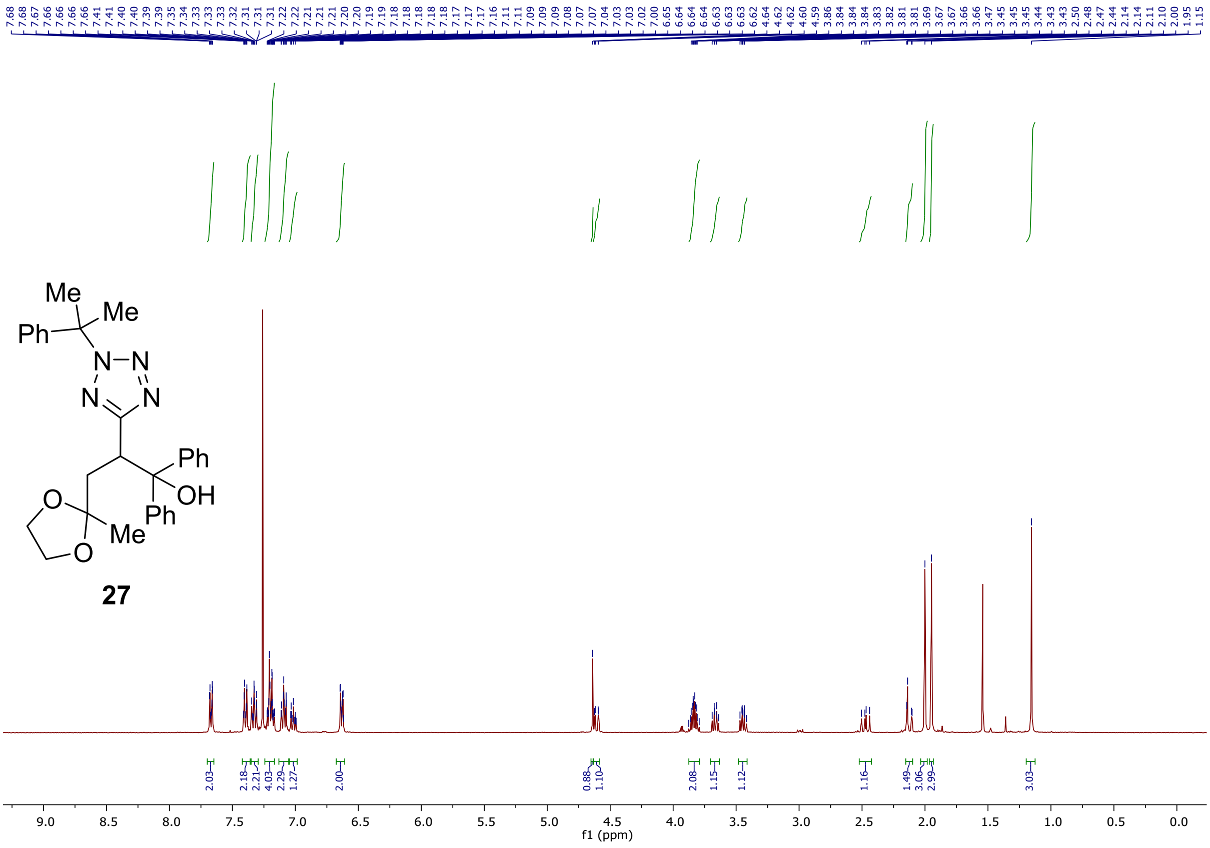
7.68 7.68 7.67 7.66 7.66 7.66 7.41 7.40 7.39 7.39 7.35 7.34 7.33 7.33 7.32 7.31 7.31 7.22 7.22 7.21 7.21 7.20 7.19 7.19 7.18 7.18 7.18 7.18 7.17 7.17 7.16 7.11 7.09 7.09 7.09 7.08 7.07 7.04 7.03 7.03 7.02 7.00 6.65 6.64 6.64 6.63 6.63 6.63 6.62 4.64 4.62 4.62 4.60 4.59 3.86 3.84 3.84 3.83 3.82 3.81 3.81 3.69 3.67 3.66 3.47 3.45 3.45 3.44 3.43 3.43 2.50 2.48 2.47 2.44 2.14 2.11 2.10 2.00 1.95 1.15
Me
Me
Ph
N
N
N
N
Ph
OH
Ph
O
O
Me
27
2.03
2.18
2.21
4.03
2.29
1.27
2.00
0.88
1.10
2.08
1.15
1.12
1.16
1.49
3.06
2.99
3.03
9.0 8.5 8.0 7.5 7.0 6.5 6.0 5.5 5.0 4.5 4.0 3.5 3.0 2.5 2.0 1.5 1.0 0.5 0.0
f1 (ppm)

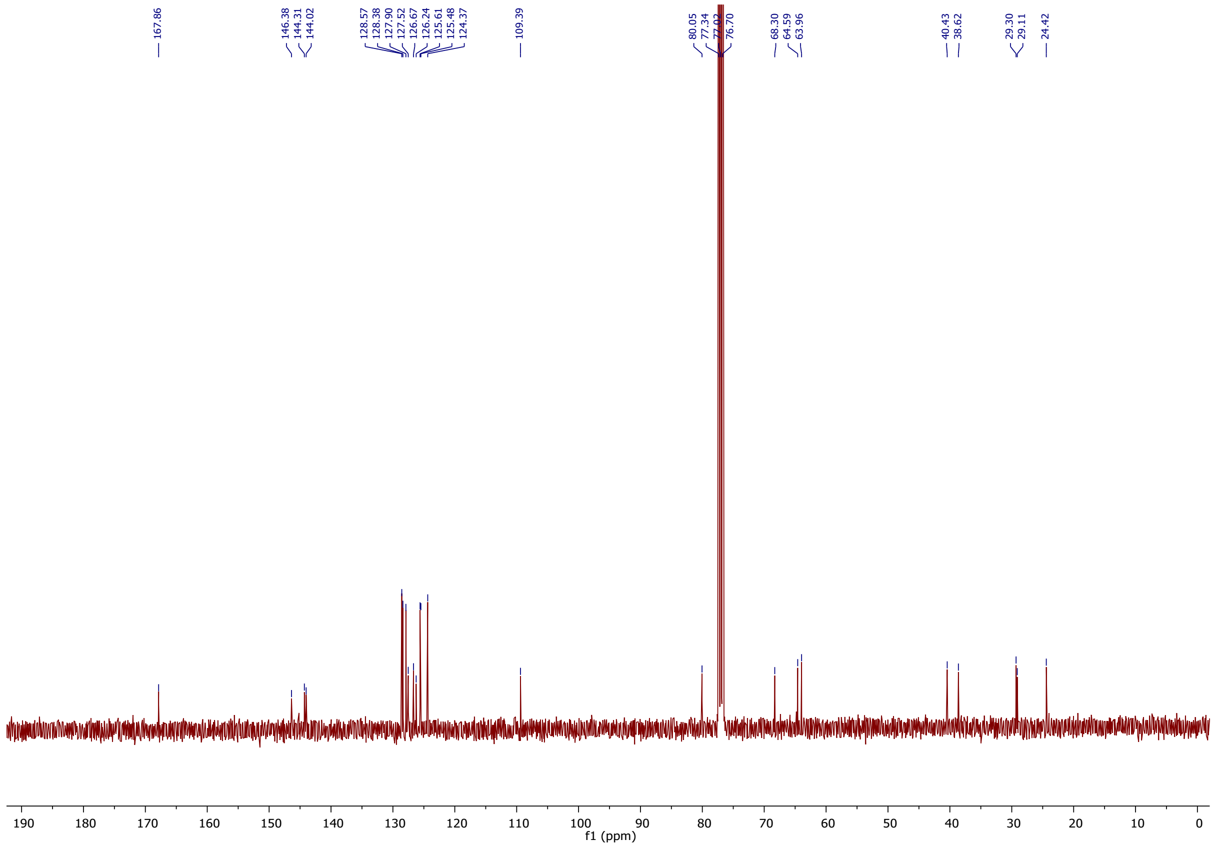
167.86
146.38
144.31
144.02
128.57
128.38
127.90
127.52
126.67
126.24
125.61
125.48
124.37
109.39
80.05
77.34
77.02
76.70
68.30
64.59
63.96
40.43
38.62
29.30
29.11
24.42
190 180 170 160 150 140 130 120 110 100 90 80 70 60 50 40 30 20 10 0
f1 (ppm)

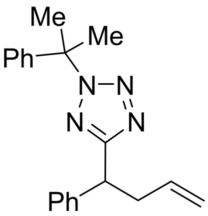
Me
Me
Ph
N
N
N
N
Ph

28

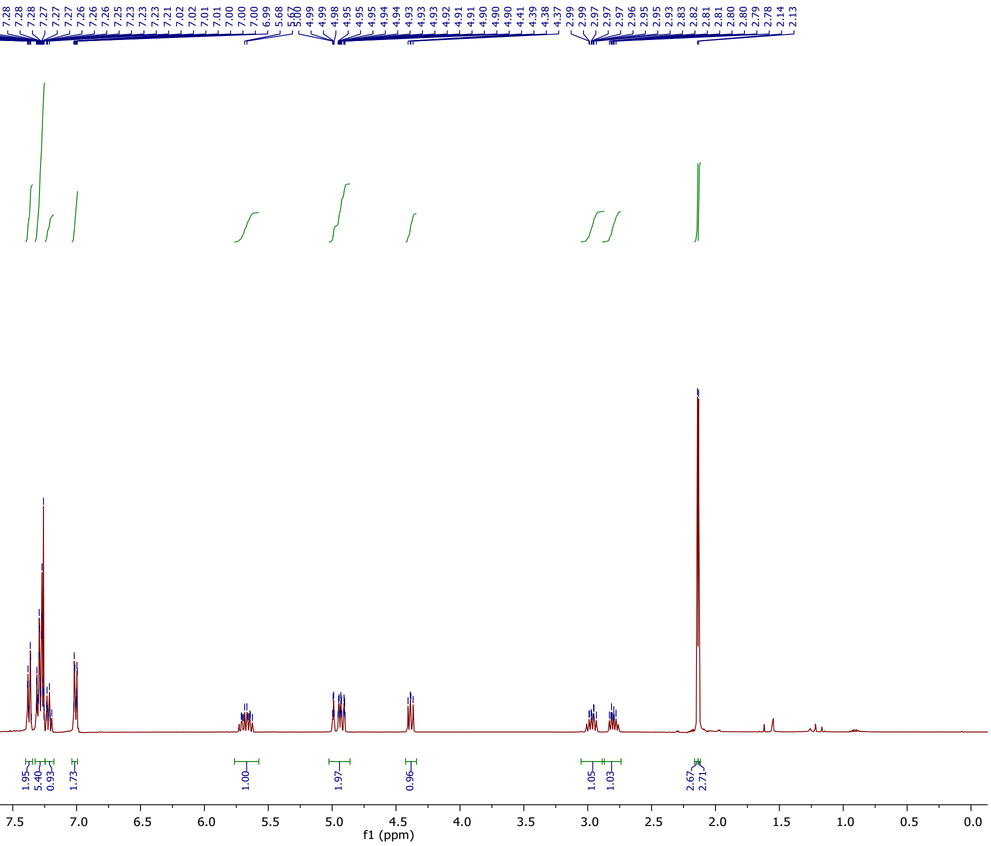
7.38 7.38 7.38 7.37 7.36 7.32 7.31 7.31 7.31 7.30 7.30 7.29 7.29 7.29 7.28 7.28 7.27 7.27 7.26 7.26 7.25 7.23 7.23 7.21 7.02 7.01 7.01 7.00 7.00 6.99 5.68 5.67 5.00 4.99 4.99 4.98 4.95 4.95 4.95 4.94 4.94 4.93 4.93 4.93 4.92 4.91 4.91 4.90 4.90 4.90 4.41 4.39 4.38 4.37 2.99 2.97 2.97 2.97 2.96 2.95 2.95 2.93 2.83 2.82 2.81 2.81 2.80 2.79 2.78 2.14 2.13
1.95
5.40
0.93
1.73
1.00
1.97
0.96
1.05
1.03
2.67
2.71
9.0 8.5 8.0 7.5 7.0 6.5 6.0 5.5 5.0 4.5 4.0 3.5 3.0 2.5 2.0 1.5 1.0 0.5 0.0
f1 (ppm)

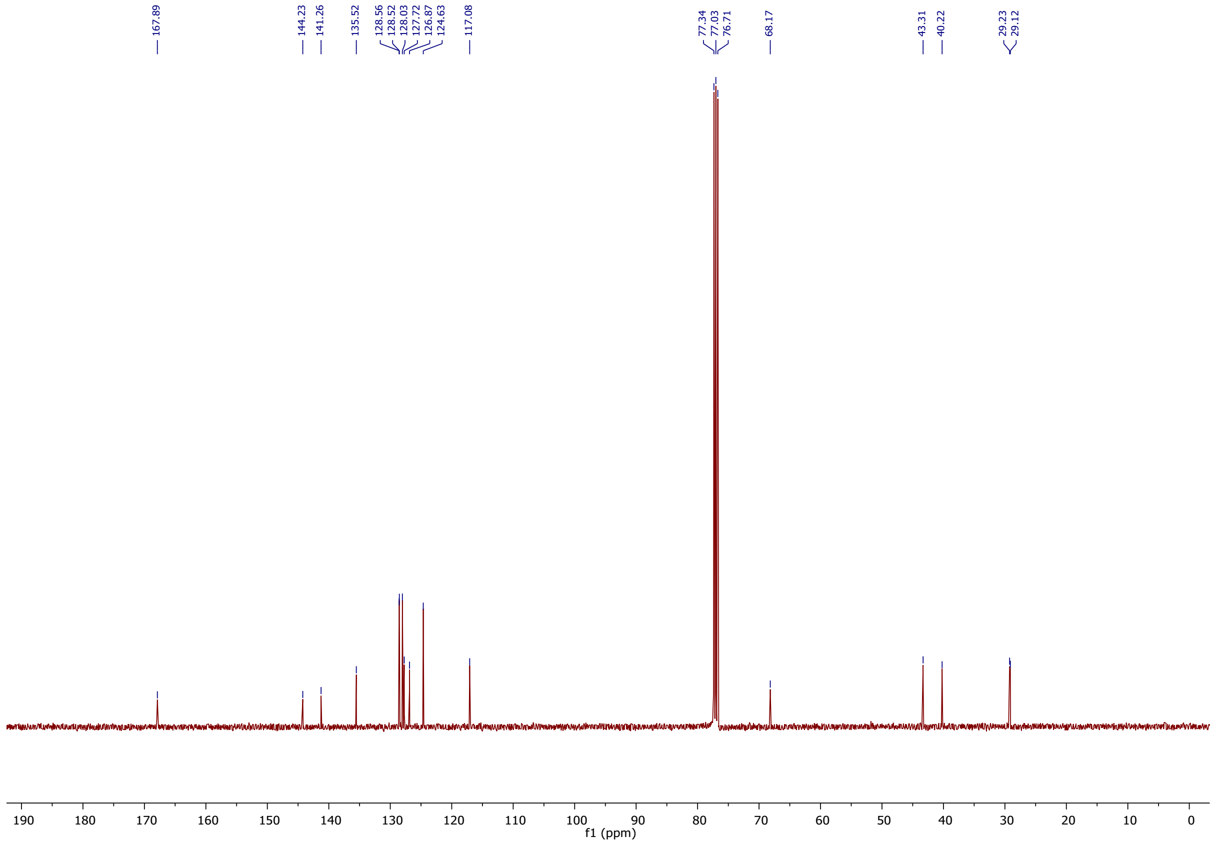
167.89
144.23
141.26
135.52
128.56
128.52
128.03
127.72
126.87
124.63
117.08
77.34
77.03
76.71
68.17
43.31
40.22
29.23
29.12
f1 (ppm)

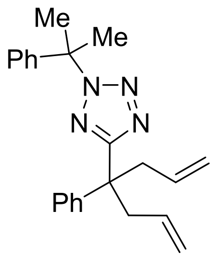
Me
Me
Ph
N
N
N
N
Ph

28'

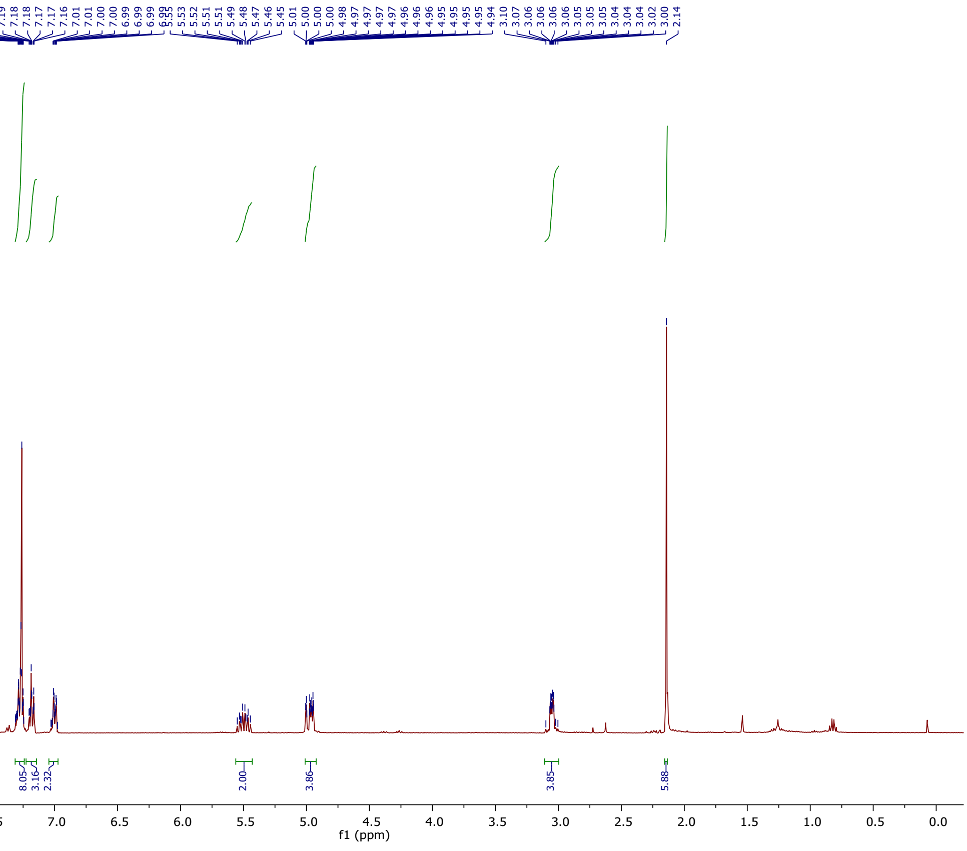
8.05
3.16
2.32
2.00
3.86
3.85
5.88
f1 (ppm)

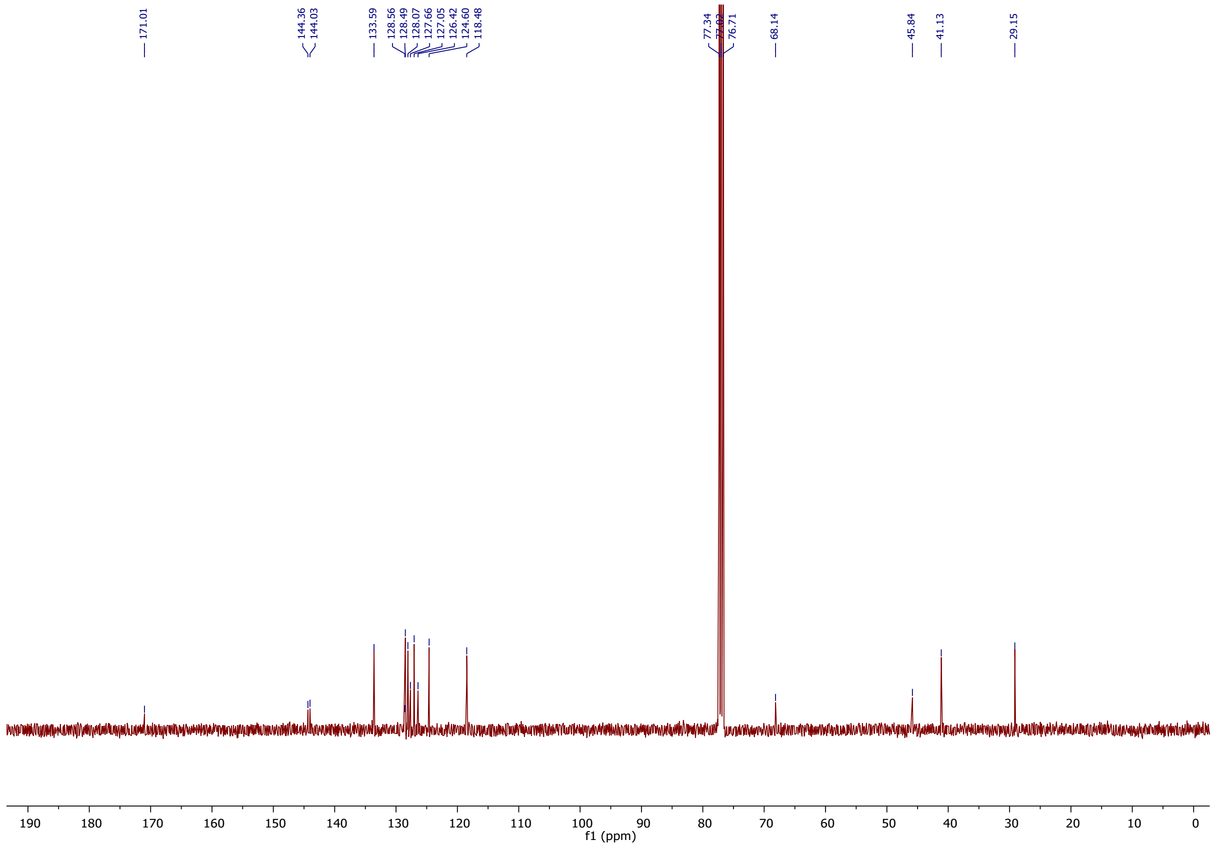
171.01
144.36
144.03
133.59
128.56
128.49
128.07
127.66
127.05
126.42
124.60
118.48
77.34
76.71
68.14
45.84
41.13
29.15
f1 (ppm)

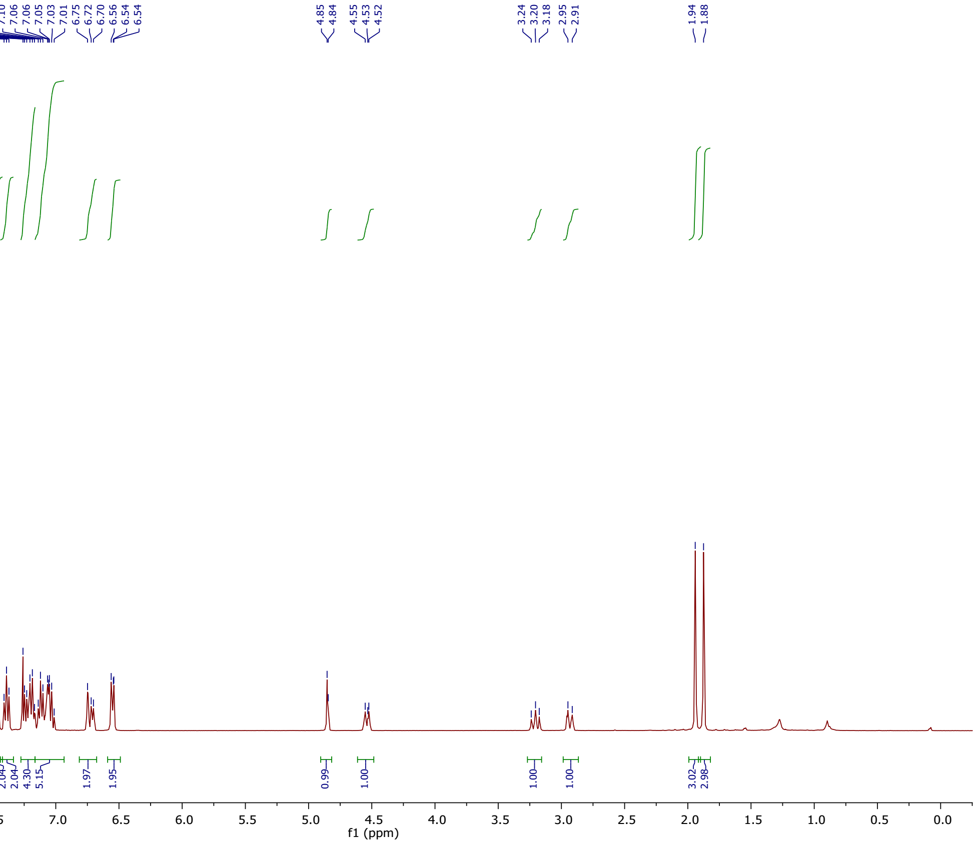
7.73
7.71
7.48
7.46
7.41
7.39
7.37
7.26
7.25
7.23
7.20
7.19
7.17
7.14
7.12
7.10
7.06
7.05
7.03
7.01
6.75
6.72
6.70
6.56
6.54
6.54
4.85
4.84
4.55
4.53
4.52
3.24
3.20
3.18
2.95
2.91
1.94
1.88
1.98
2.04
2.04
4.30
5.15
1.97
1.95
0.99
1.00
1.00
1.00
3.02
2.98
f1 (ppm)

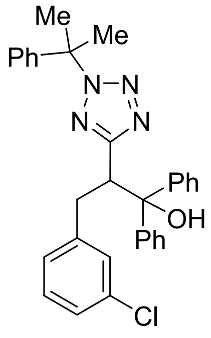
Me
Me
Ph
N
N
N
N
Ph
OH
Ph
Cl

30

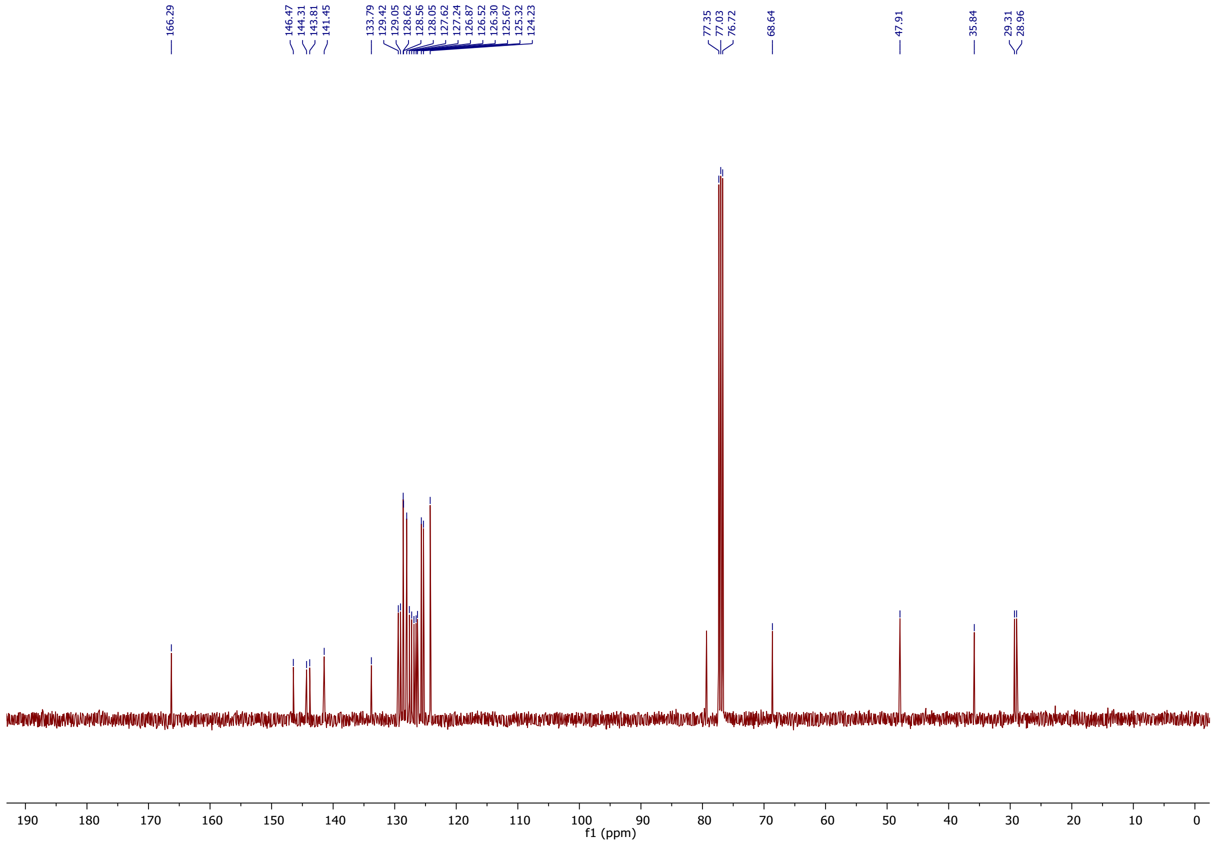
166.29
146.47
144.31
143.81
141.45
133.79
129.42
129.05
128.62
128.56
128.05
127.62
127.24
126.87
126.52
126.30
125.67
125.32
124.23
77.35
77.03
76.72
68.64
47.91
35.84
29.31
28.96
190 180 170 160 150 140 130 120 110 100 90 80 70 60 50 40 30 20 10 0
f1 (ppm)

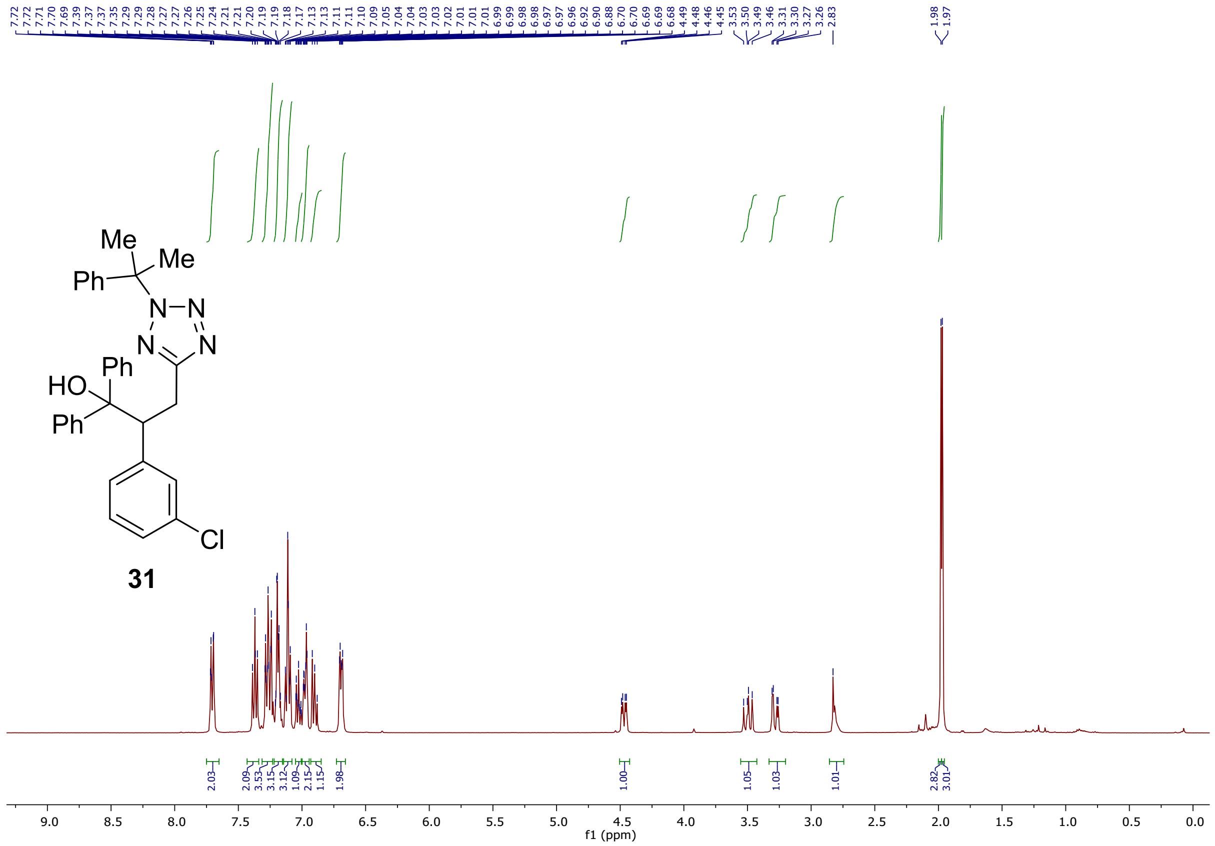
7.72 7.72 7.71 7.70 7.69 7.39 7.37 7.37 7.35 7.29 7.29 7.28 7.27 7.27 7.26 7.25 7.24 7.21 7.21 7.20 7.19 7.19 7.18 7.17 7.13 7.13 7.11 7.11 7.10 7.09 7.05 7.04 7.04 7.03 7.03 7.02 7.01 7.01 6.99 6.99 6.98 6.98 6.97 6.96 6.92 6.90 6.88 6.70 6.70 6.69 6.69 6.68 4.49 4.48 4.46 4.45 3.53 3.50 3.49 3.46 3.31 3.30 3.27 3.26 2.83
1.98
1.97
2.03 2.09 3.53 3.15 3.12 1.09 2.15 1.15 1.98 1.00 1.05 1.03 1.01 2.82 3.01
9.0 8.5 8.0 7.5 7.0 6.5 6.0 5.5 5.0 4.5 4.0 3.5 3.0 2.5 2.0 1.5 1.0 0.5 0.0
f1 (ppm)
Me
Me
Ph
N
N
N
N
Ph
HO
Ph
Cl

31

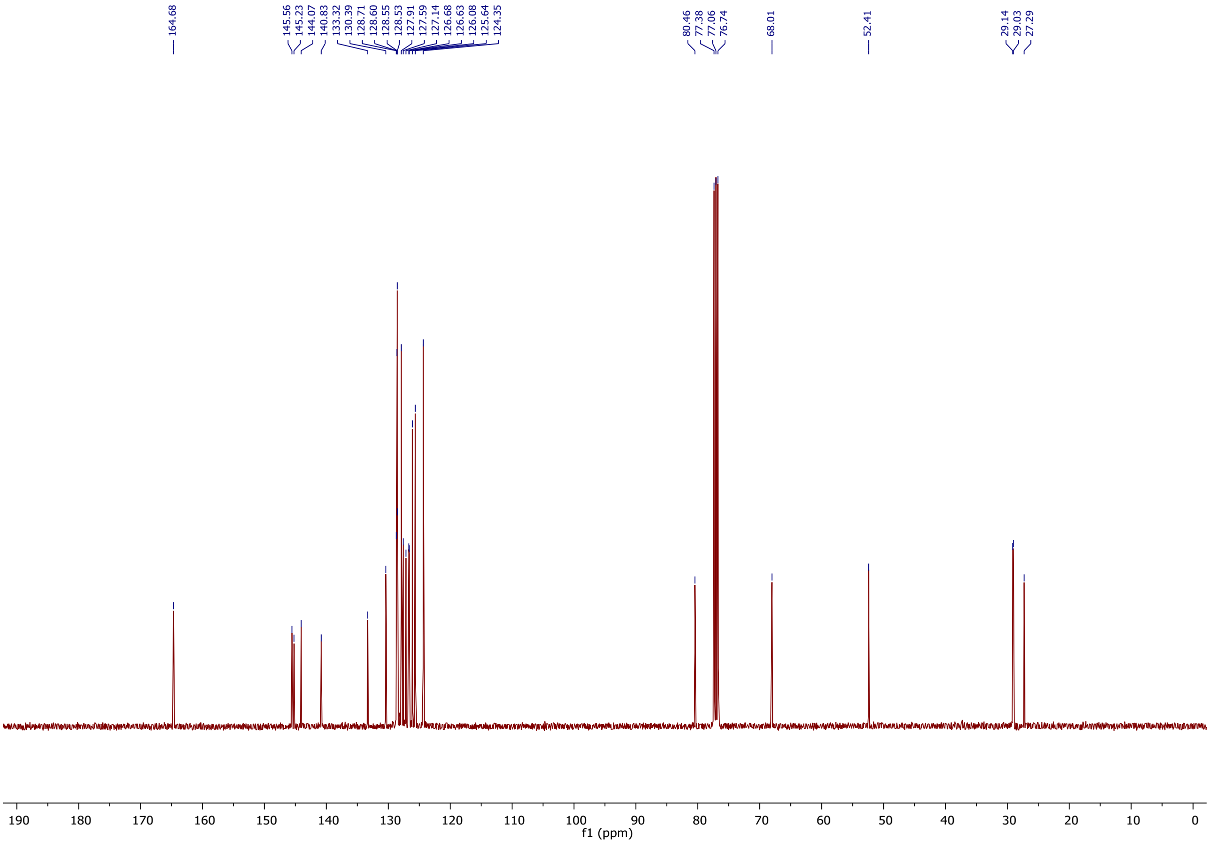
164.68
145.56
145.23
144.07
140.83
133.32
130.39
128.71
128.60
128.55
128.53
127.91
127.59
127.14
126.68
126.63
126.08
125.64
124.35
80.46
77.38
77.06
76.74
68.01
52.41
29.14
29.03
27.29
190 180 170 160 150 140 130 120 110 100 90 80 70 60 50 40 30 20 10 0
f1 (ppm)

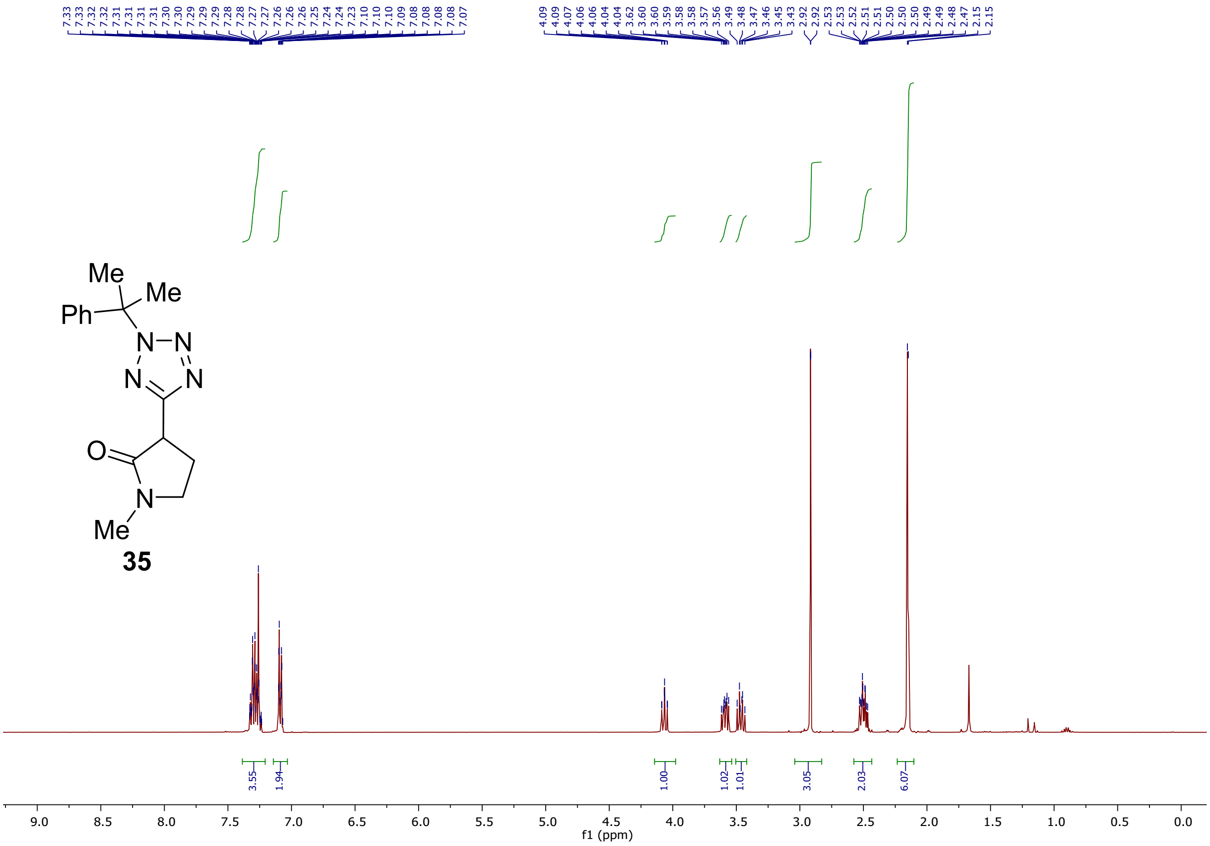
7.33
7.33
7.32
7.31
7.31
7.31
7.30
7.29
7.29
7.28
7.27
7.27
7.26
7.26
7.25
7.24
7.24
7.23
7.10
7.10
7.09
7.08
7.08
7.08
7.07
4.09
4.09
4.07
4.06
4.06
4.04
4.04
3.62
3.60
3.60
3.59
3.58
3.57
3.56
3.49
3.48
3.47
3.46
3.45
3.43
2.92
2.92
2.53
2.52
2.51
2.51
2.50
2.50
2.49
2.49
2.48
2.47
2.15
2.15
Me
Me
Ph
N
N
N
N
O
N
Me
35
3.55
1.94
1.00
1.02
1.01
3.05
2.03
6.07
9.0 8.5 8.0 7.5 7.0 6.5 6.0 5.5 5.0 4.5 4.0 3.5 3.0 2.5 2.0 1.5 1.0 0.5 0.0
f1 (ppm)

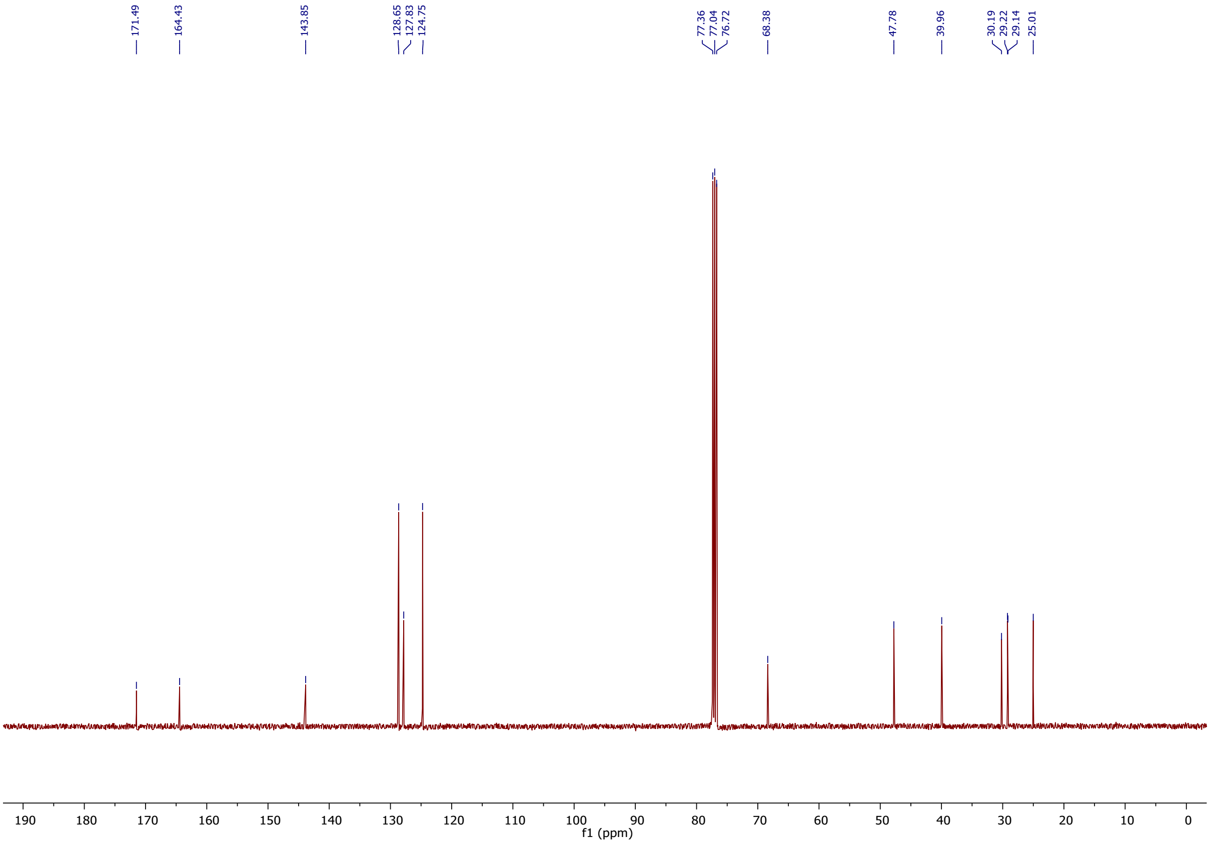
171.49
164.43
143.85
128.65
127.83
124.75
77.36
77.04
76.72
68.38
47.78
39.96
30.19
29.22
29.14
25.01
190 180 170 160 150 140 130 120 110 100 90 80 70 60 50 40 30 20 10 0
f1 (ppm)

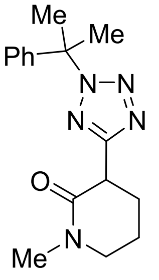
Me
Me
Ph
N
N
N
N
O
N
Me

36

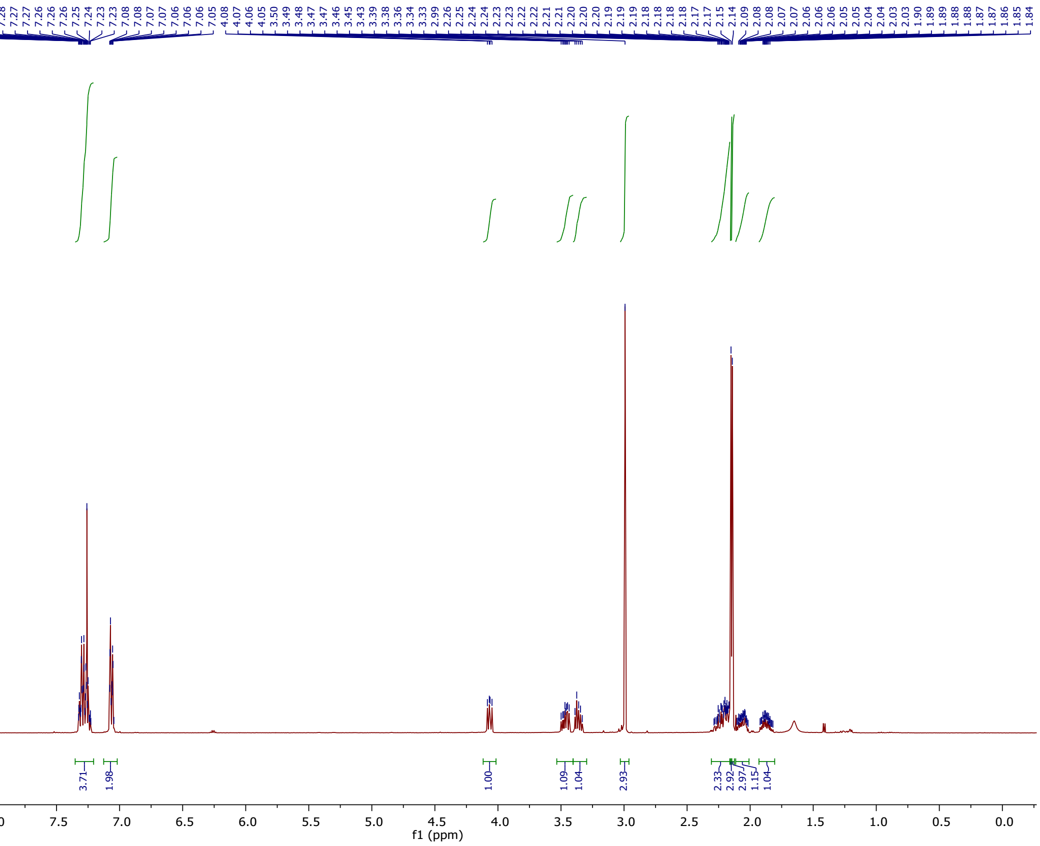
7.33 7.32 7.32 7.31 7.31 7.31 7.30 7.30 7.30 7.29 7.28 7.28 7.28 7.27 7.27 7.26 7.26 7.25 7.24 7.23 7.23 7.08 7.08 7.07 7.07 7.06 7.06 7.06 7.05 4.08 4.07 4.06 4.05 3.50 3.49 3.48 3.47 3.46 3.45 3.43 3.39 3.38 3.36 3.34 3.33 2.99 2.26 2.25 2.24 2.24 2.23 2.23 2.22 2.22 2.21 2.21 2.20 2.20 2.20 2.19 2.19 2.19 2.18 2.18 2.18 2.17 2.17 2.15 2.14 2.09 2.08 2.08 2.07 2.07 2.06 2.06 2.06 2.05 2.05 2.04 2.04 2.03 2.03 1.90 1.89 1.89 1.88 1.88 1.87 1.87 1.86 1.85 1.84
3.71
1.98
1.00
1.09
1.04
2.93
2.33
2.92
2.97
1.15
1.04
9.0 8.5 8.0 7.5 7.0 6.5 6.0 5.5 5.0 4.5 4.0 3.5 3.0 2.5 2.0 1.5 1.0 0.5 0.0
f1 (ppm)

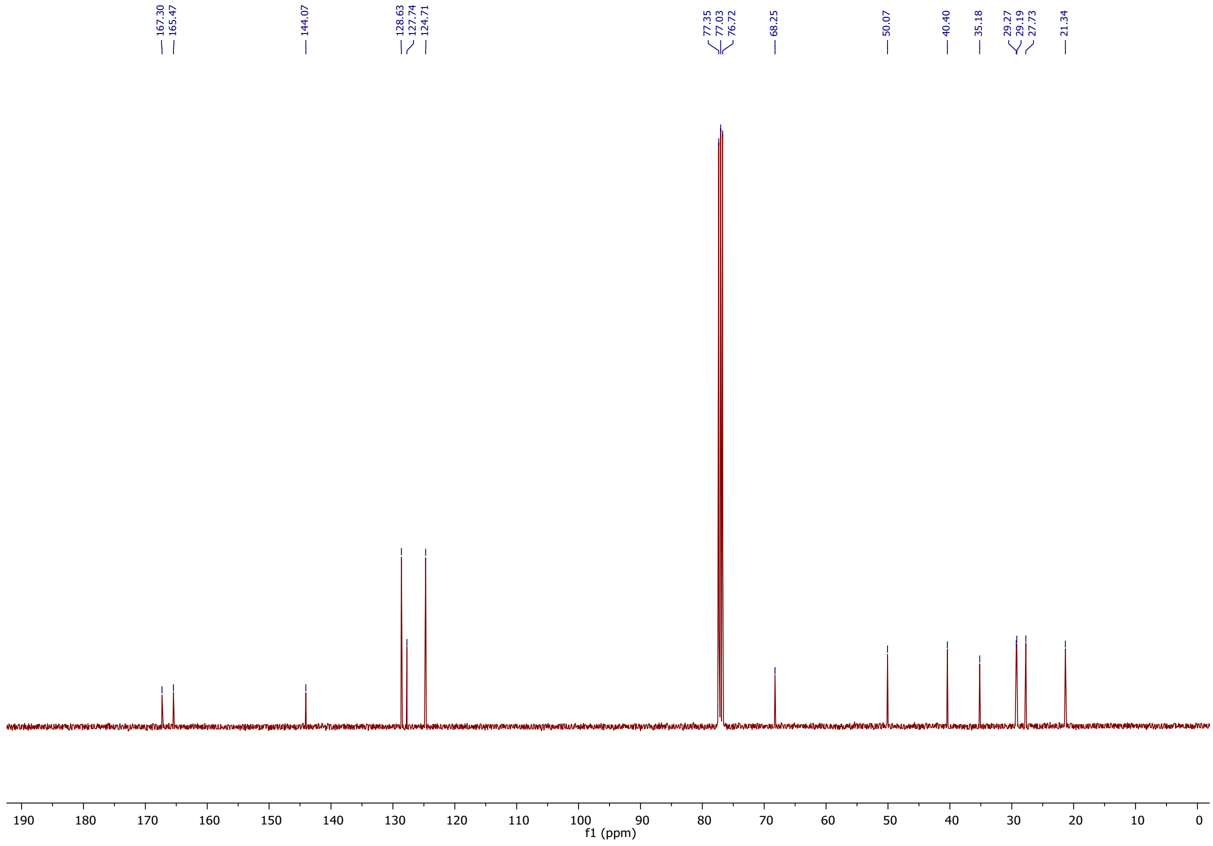

167.30
165.47
144.07
128.63
127.74
124.71
77.35
77.03
76.72
68.25
50.07
40.40
35.18
29.27
29.19
27.73
21.34
f1 (ppm)

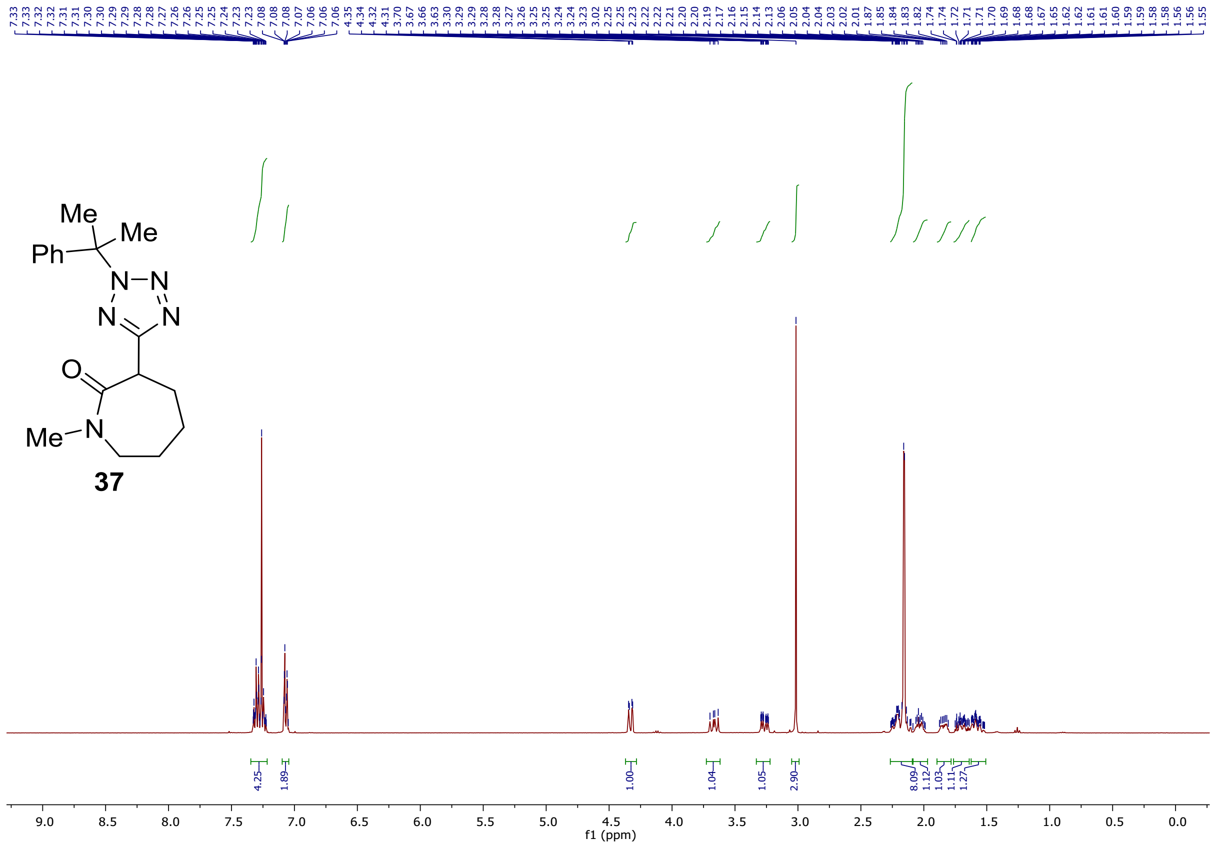

Me
Me
Ph
N
N
N
N
O
Me
N
37
f1 (ppm)

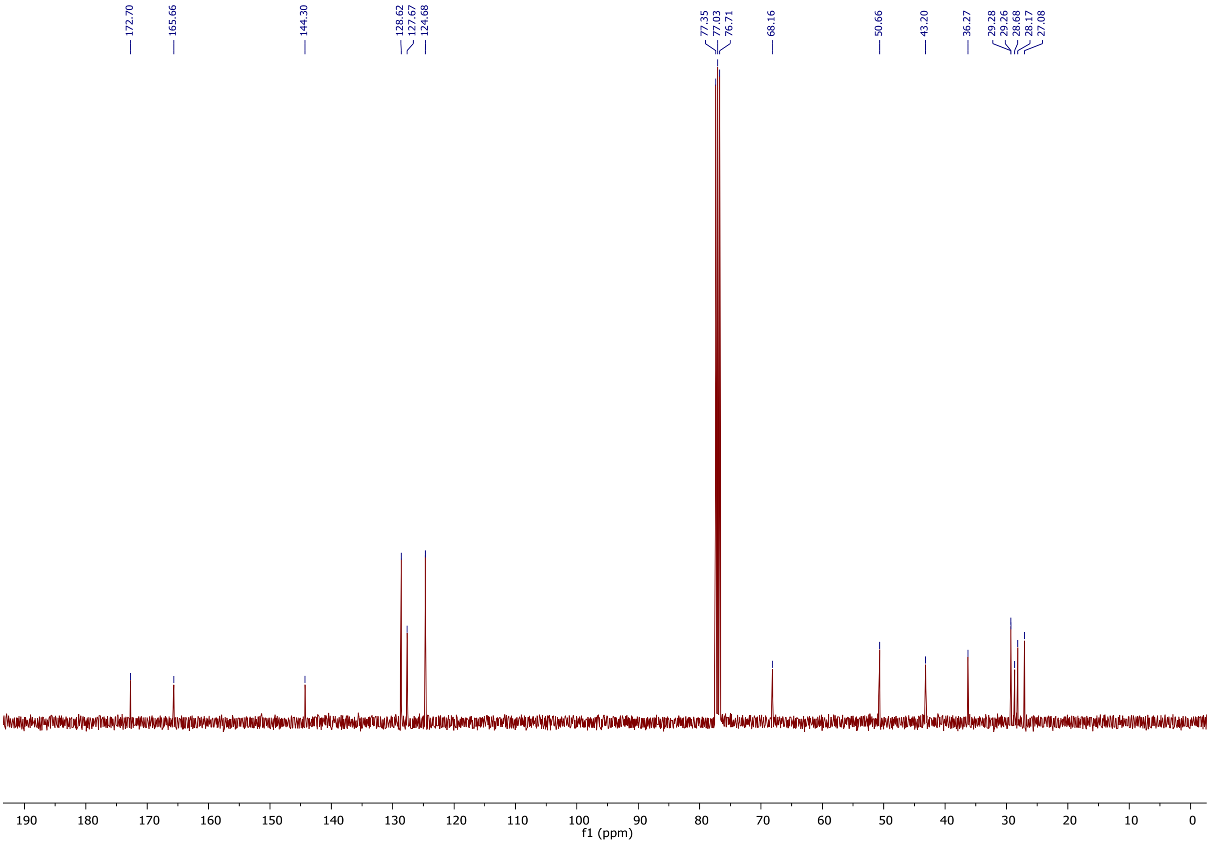
172.70
165.66
144.30
128.62
127.67
124.68
77.35
77.03
76.71
68.16
50.66
43.20
36.27
29.28
29.26
28.68
28.17
27.08
190 180 170 160 150 140 130 120 110 100 90 80 70 60 50 40 30 20 10 0
f1 (ppm)

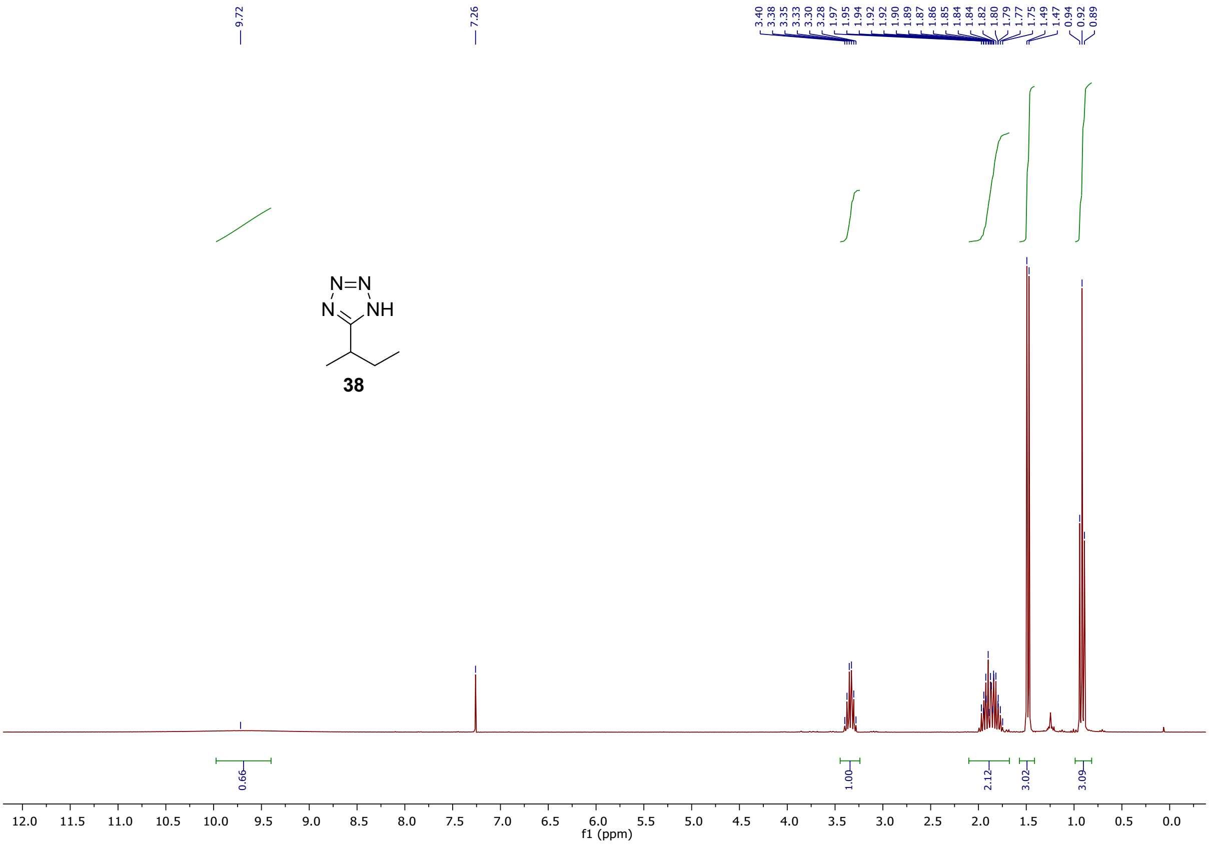
9.72
7.26
3.40
3.38
3.35
3.33
3.30
3.28
1.97
1.95
1.94
1.92
1.92
1.90
1.89
1.87
1.86
1.85
1.84
1.82
1.80
1.79
1.77
1.75
1.49
1.47
0.94
0.92
0.89
N=N
N
NH
38
0.66
1.00
2.12
3.02
3.09
12.0 11.5 11.0 10.5 10.0 9.5 9.0 8.5 8.0 7.5 7.0 6.5 6.0 5.5 5.0 4.5 4.0 3.5 3.0 2.5 2.0 1.5 1.0 0.5 0.0
f1 (ppm)

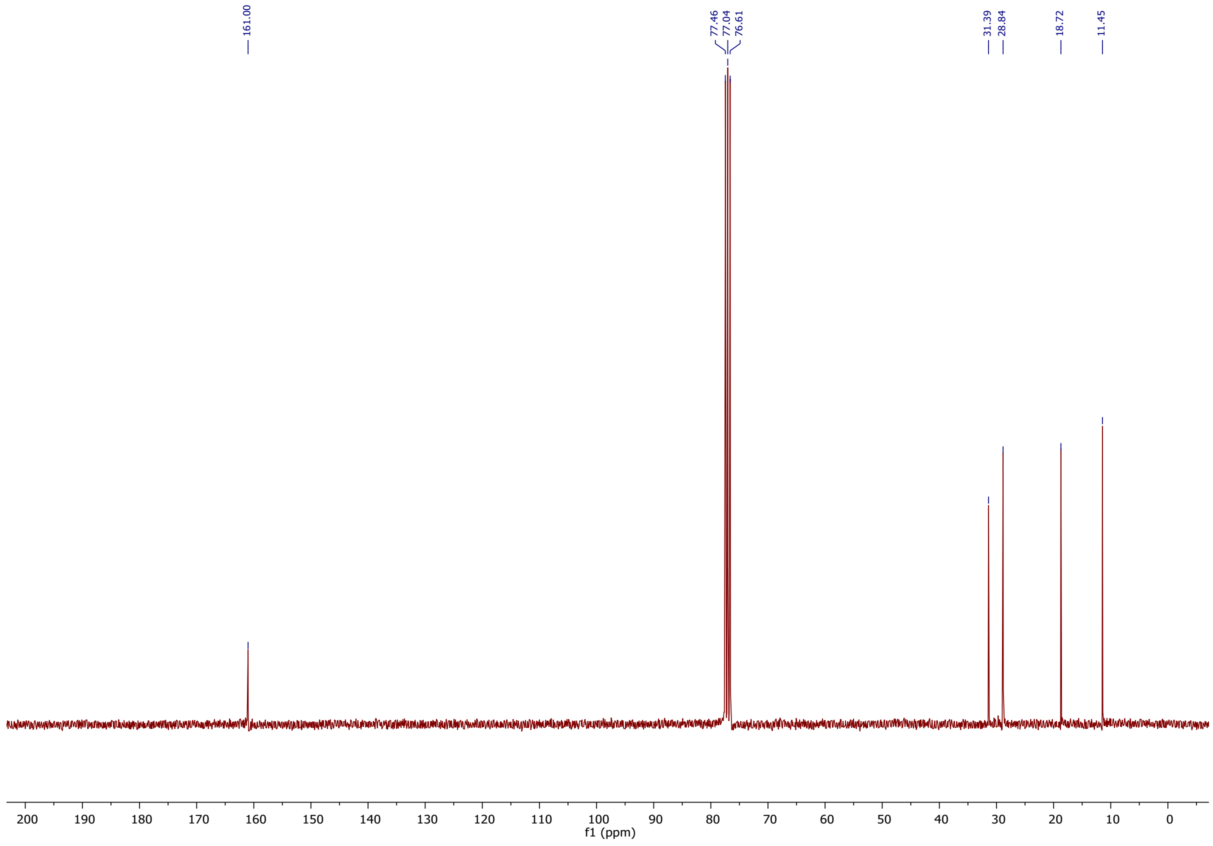
161.00
77.46
77.04
76.61
31.39
28.84
18.72
11.45
200 190 180 170 160 150 140 130 120 110 100 90 80 70 60 50 40 30 20 10 0
f1 (ppm)

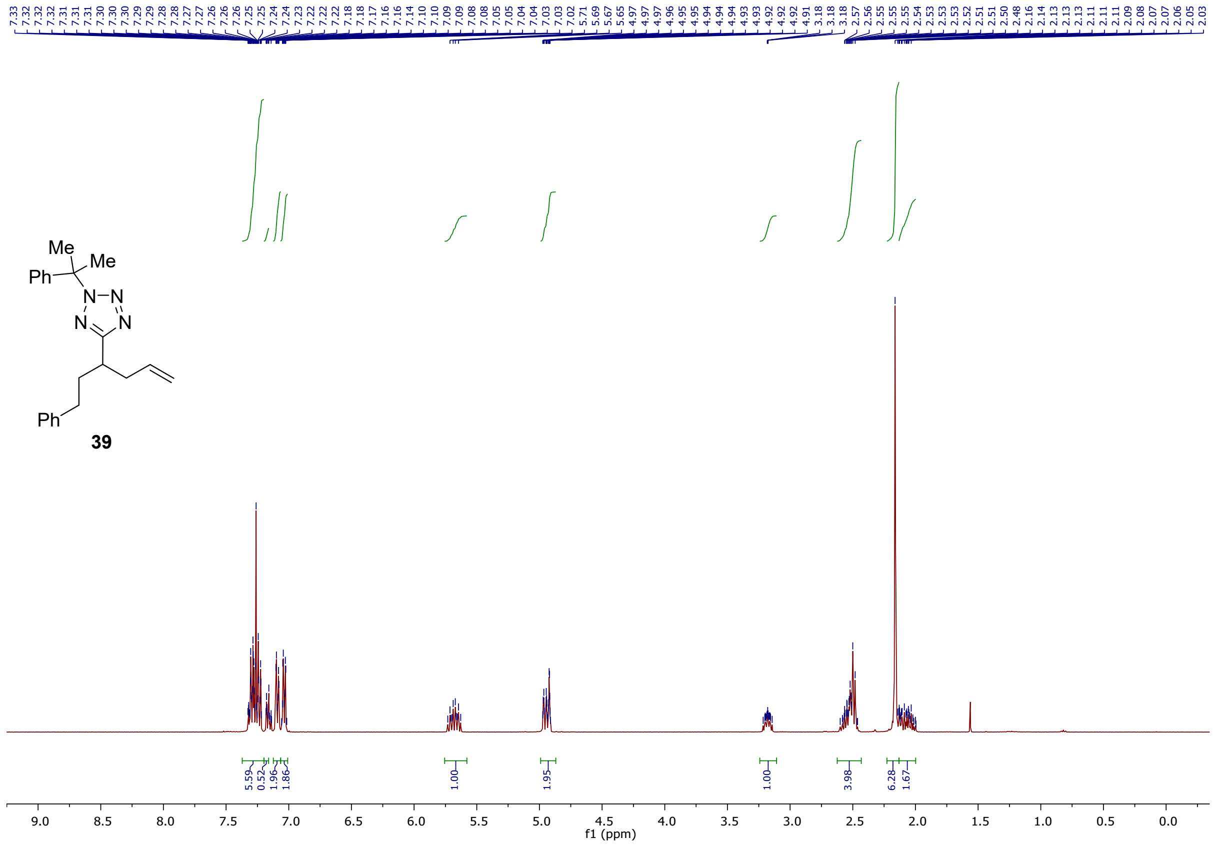
Me
Me
Ph
N
N
N
N
Ph
7.33 7.32 7.32 7.31 7.31 7.31 7.30 7.30 7.29 7.29 7.28 7.28 7.27 7.27 7.26 7.26 7.25 7.25 7.24 7.24 7.23 7.22 7.22 7.18 7.18 7.17 7.16 7.16 7.14 7.10 7.09 7.09 7.08 7.05 7.05 7.04 7.03 7.03 7.02 5.71 5.69 5.67 5.65 4.97 4.97 4.96 4.95 4.95 4.94 4.94 4.94 4.93 4.93 4.92 4.92 4.91 3.18 3.18 2.57 2.56 2.55 2.55 2.55 2.54 2.53 2.53 2.52 2.51 2.51 2.50 2.48 2.16 2.14 2.13 2.13 2.13 2.11 2.11 2.11 2.09 2.08 2.07 2.06 2.05 2.03
5.59 0.52 1.96 1.86 1.00 1.95 1.00 3.98 6.28 1.67
9.0 8.5 8.0 7.5 7.0 6.5 6.0 5.5 5.0 4.5 4.0 3.5 3.0 2.5 2.0 1.5 1.0 0.5 0.0
f1 (ppm)
39

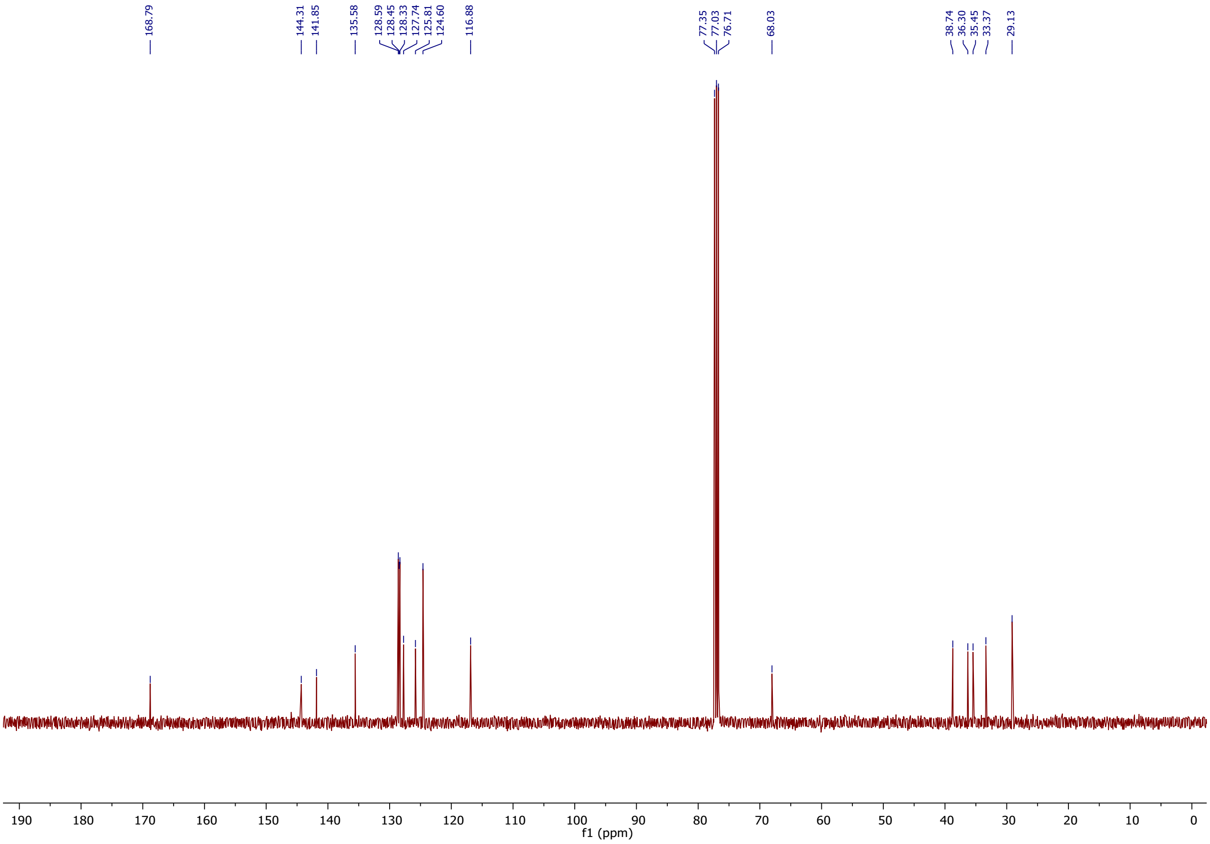
168.79
144.31
141.85
135.58
128.59
128.45
128.33
127.74
125.81
124.60
116.88
77.35
77.03
76.71
68.03
38.74
36.30
35.45
33.37
29.13
190 180 170 160 150 140 130 120 110 100 90 80 70 60 50 40 30 20 10 0
f1 (ppm)

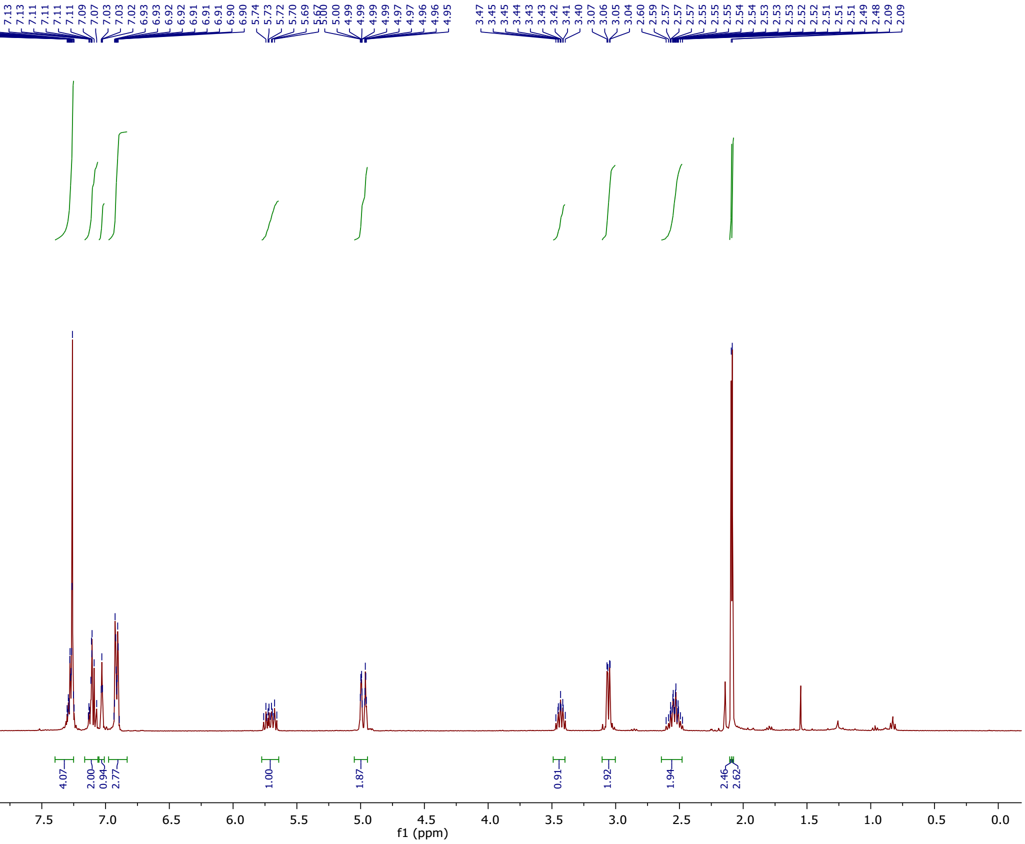
7.30
7.30
7.29
7.29
7.28
7.28
7.28
7.27
7.27
7.26
7.26
7.25
7.25
7.13
7.13
7.11
7.11
7.11
7.09
7.07
7.03
7.03
7.02
6.93
6.93
6.92
6.92
6.91
6.91
6.90
6.90
5.74
5.73
5.72
5.70
5.69
5.67
5.66
5.00
4.99
4.99
4.99
4.97
4.97
4.96
4.96
4.95
3.47
3.45
3.44
3.43
3.43
3.42
3.41
3.40
3.07
3.06
3.05
3.04
2.60
2.59
2.57
2.57
2.55
2.55
2.55
2.54
2.54
2.53
2.53
2.53
2.52
2.52
2.51
2.51
2.49
2.48
2.09
2.09
4.07
2.00
0.94
2.77
1.00
1.87
0.91
1.92
1.94
2.46
2.62
9.0 8.5 8.0 7.5 7.0 6.5 6.0 5.5 5.0 4.5 4.0 3.5 3.0 2.5 2.0 1.5 1.0 0.5 0.0
f1 (ppm)

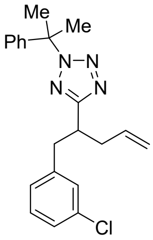
Me
Me
Ph
N
N
N
N
Cl

40

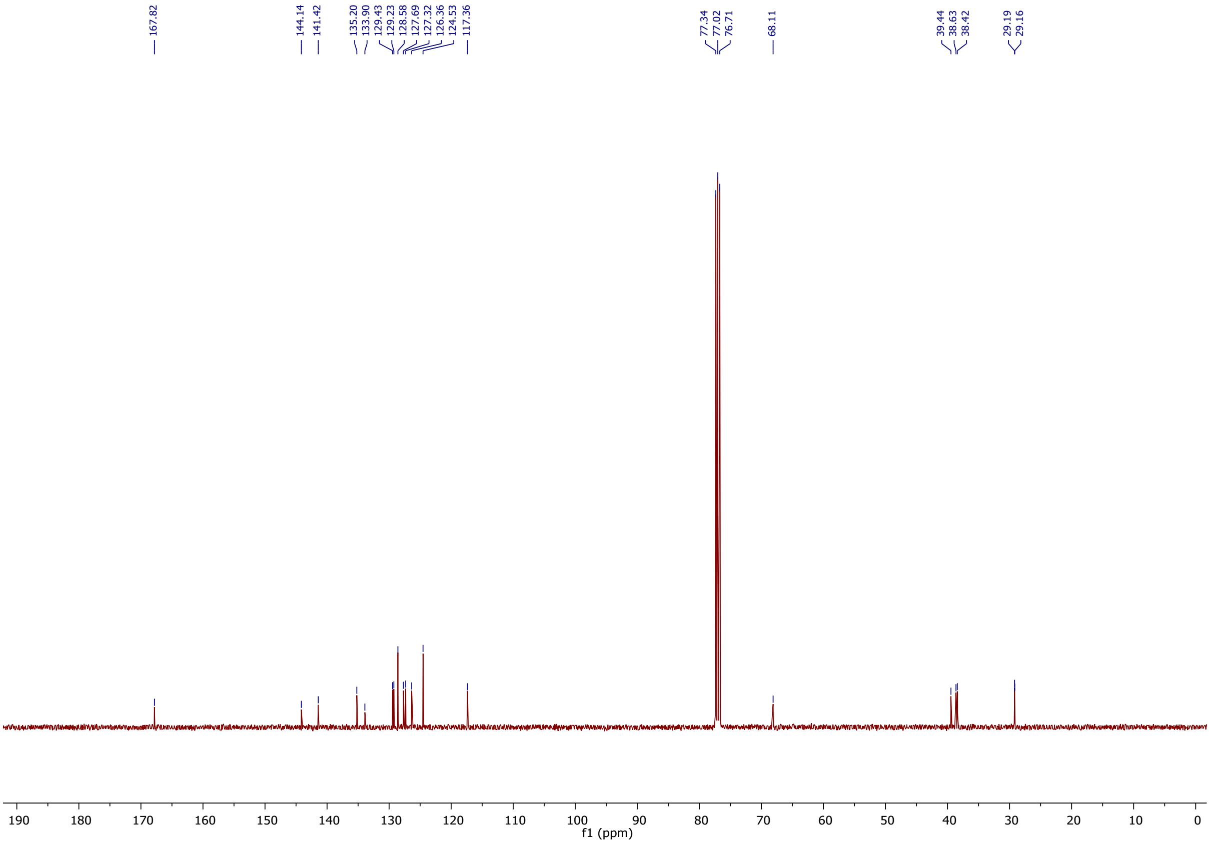
167.82
144.14
141.42
135.20
133.90
129.43
129.23
128.58
127.69
127.32
126.36
124.53
117.36
77.34
77.02
76.71
68.11
39.44
38.63
38.42
29.19
29.16
190 180 170 160 150 140 130 120 110 100 90 80 70 60 50 40 30 20 10 0
f1 (ppm)

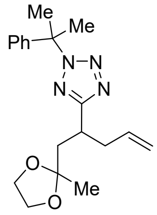
Me
Me
Ph
N
N
N
N
O
O
Me

41

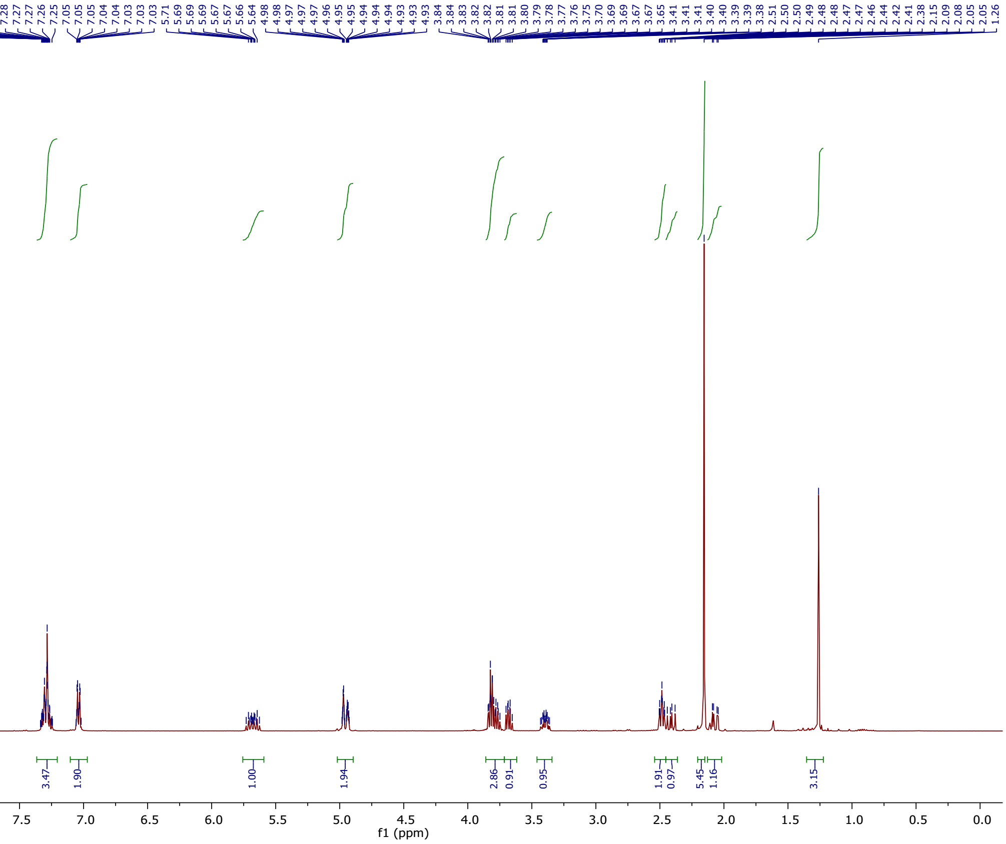
7.33 7.32 7.32 7.32 7.31 7.31 7.31 7.30 7.30 7.29 7.29 7.29 7.29 7.28 7.28 7.28 7.27 7.27 7.26 7.25 7.05 7.05 7.05 7.04 7.04 7.03 7.03 7.03 5.71 5.69 5.69 5.67 5.67 5.66 5.64 4.98 4.98 4.97 4.97 4.97 4.96 4.95 4.95 4.95 4.94 4.94 4.94 4.93 4.93 4.93 3.84 3.84 3.83 3.82 3.82 3.81 3.81 3.80 3.79 3.78 3.77 3.76 3.75 3.70 3.69 3.69 3.67 3.67 3.65 3.41 3.41 3.40 3.40 3.39 3.39 3.38 2.51 2.50 2.50 2.49 2.48 2.48 2.47 2.47 2.46 2.44 2.42 2.41 2.38 2.15 2.09 2.08 2.05 2.05 1.26
3.47
1.90
1.00
1.94
2.86
0.91
0.95
1.91
0.97
5.45
1.16
3.15
9.0 8.5 8.0 7.5 7.0 6.5 6.0 5.5 5.0 4.5 4.0 3.5 3.0 2.5 2.0 1.5 1.0 0.5 0.0
f1 (ppm)

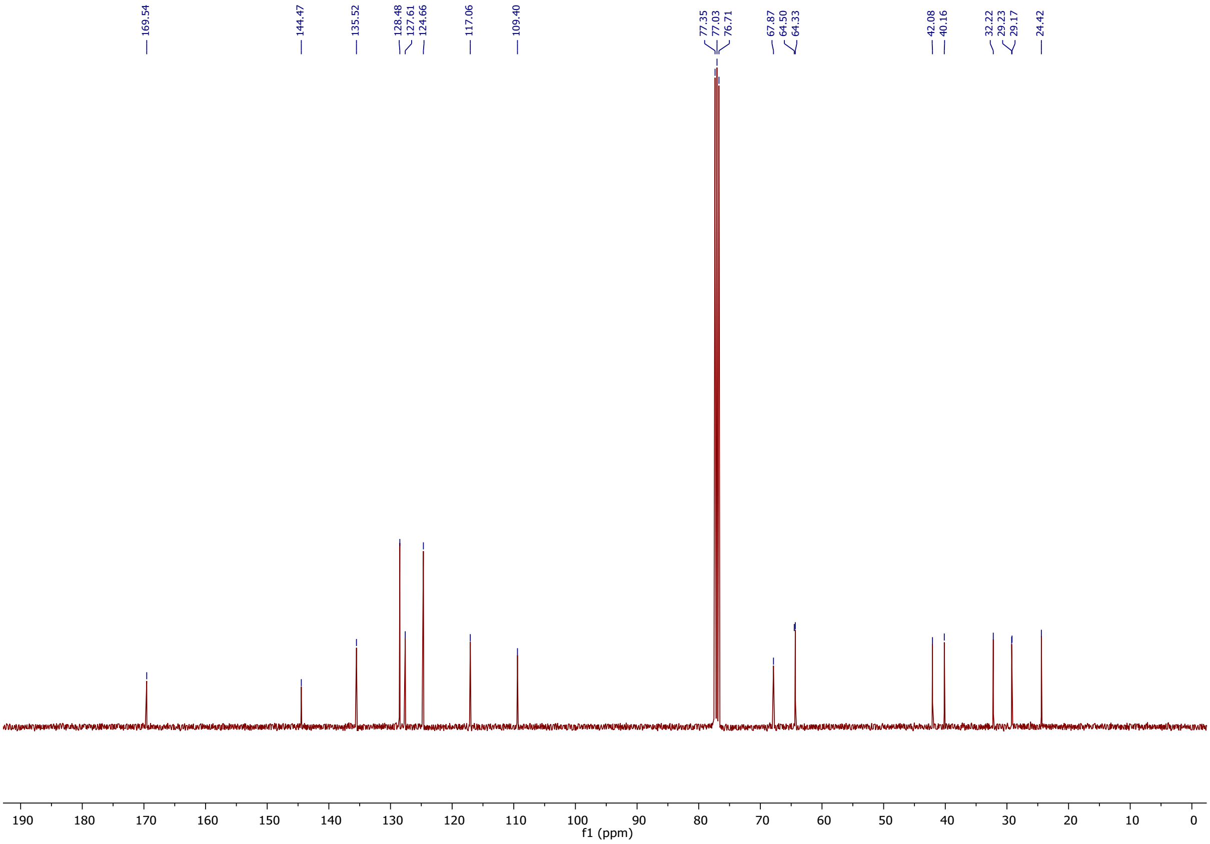
169.54
144.47
135.52
128.48
127.61
124.66
117.06
109.40
77.35
77.03
76.71
67.87
64.50
64.33
42.08
40.16
32.22
29.23
29.17
24.42
190 180 170 160 150 140 130 120 110 100 90 80 70 60 50 40 30 20 10 0
f1 (ppm)

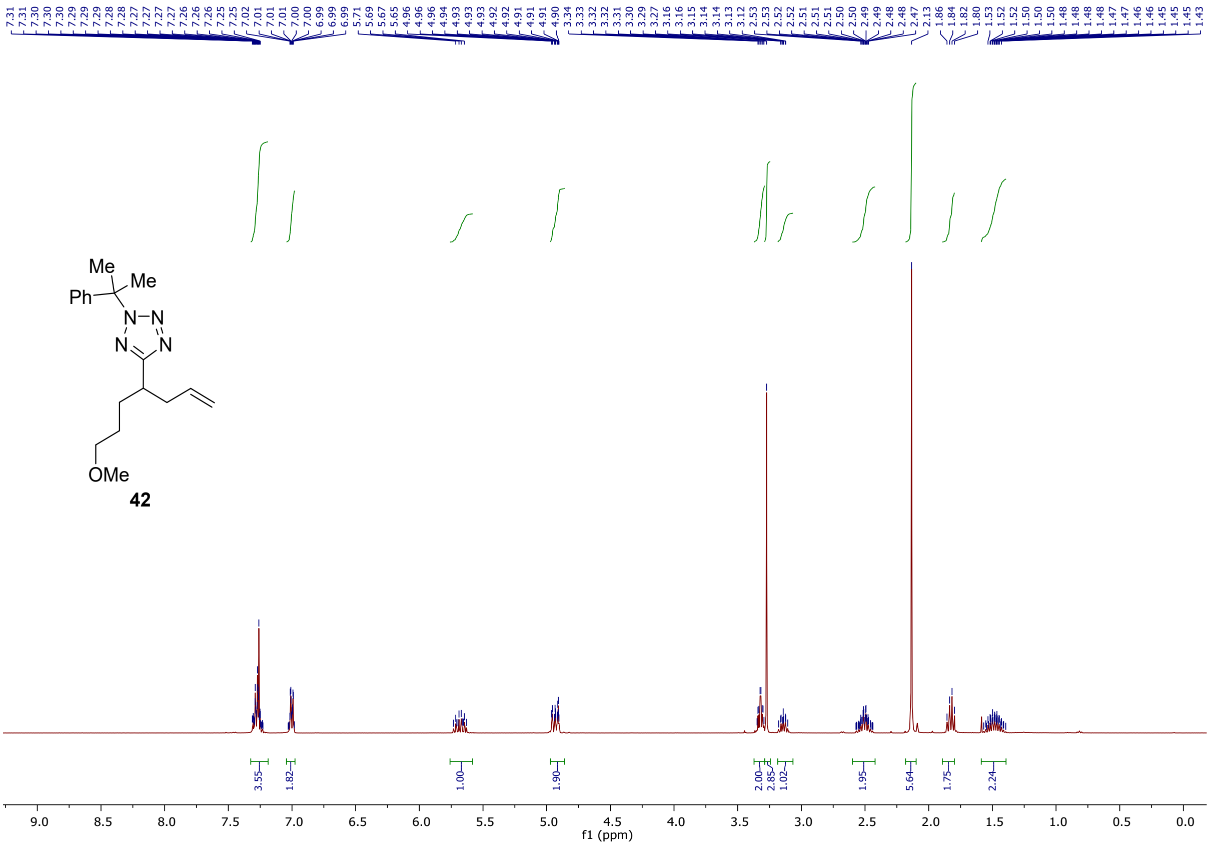
7.31 7.31 7.30 7.30 7.30 7.29 7.29 7.29 7.28 7.28 7.27 7.27 7.27 7.26 7.26 7.26 7.25 7.25 7.02 7.01 7.01 7.01 7.00 6.99 6.99 6.99 5.71 5.69 5.67 5.65 4.96 4.96 4.94 4.93 4.93 4.93 4.92 4.92 4.91 4.91 4.91 4.90 3.34 3.33 3.32 3.32 3.31 3.30 3.29 3.27 3.16 3.15 3.14 3.14 3.13 3.12 2.53 2.53 2.52 2.51 2.51 2.51 2.50 2.50 2.49 2.49 2.48 2.48 2.47 2.13 1.86 1.84 1.82 1.80 1.53 1.52 1.52 1.50 1.50 1.50 1.48 1.48 1.48 1.48 1.47 1.47 1.46 1.45 1.45 1.45 1.43
3.55
1.82
1.00
1.90
2.00
2.85
1.02
1.95
5.64
1.75
2.24
9.0 8.5 8.0 7.5 7.0 6.5 6.0 5.5 5.0 4.5 4.0 3.5 3.0 2.5 2.0 1.5 1.0 0.5 0.0
f1 (ppm)
Me
Me
Ph
N
N
N
N
OMe

42

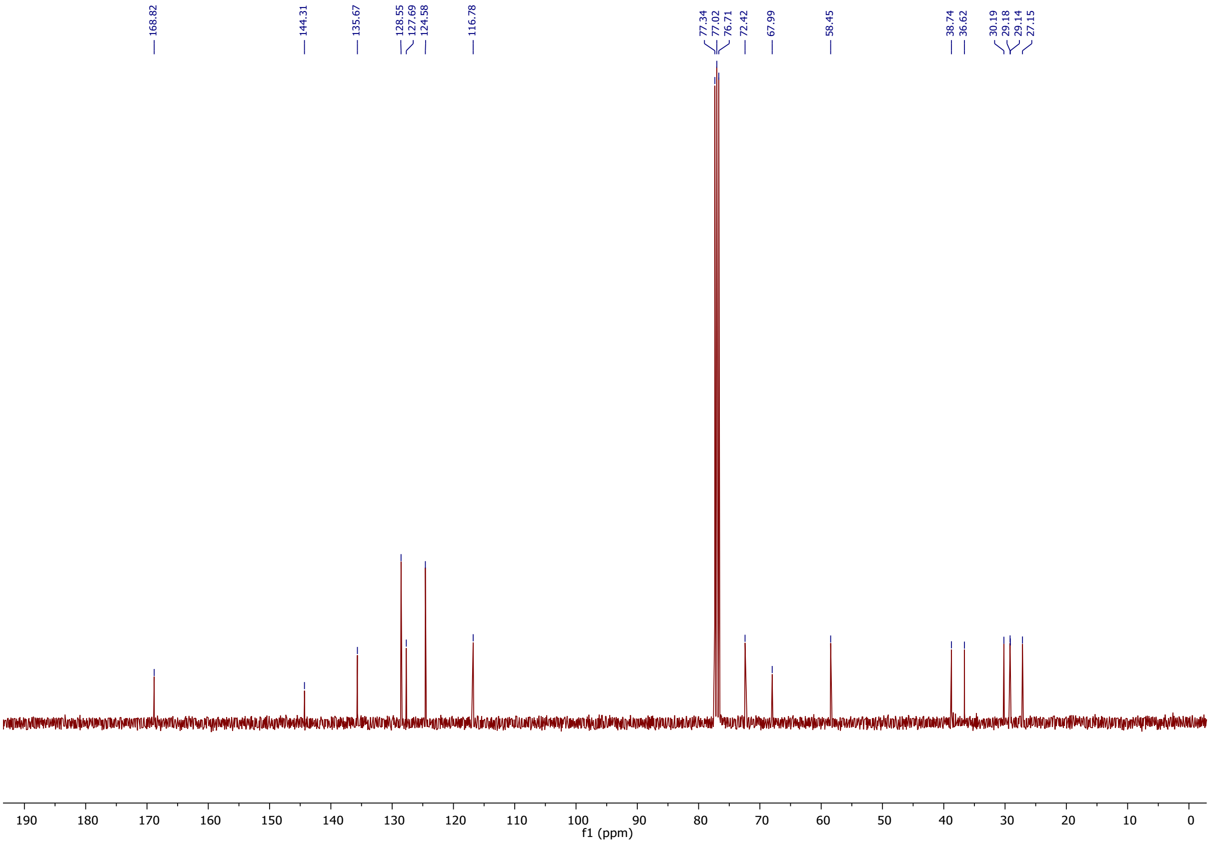
168.82
144.31
135.67
128.55
127.69
124.58
116.78
77.34
77.02
76.71
72.42
67.99
58.45
38.74
36.62
30.19
29.18
29.14
27.15
190 180 170 160 150 140 130 120 110 100 90 80 70 60 50 40 30 20 10 0
f1 (ppm)

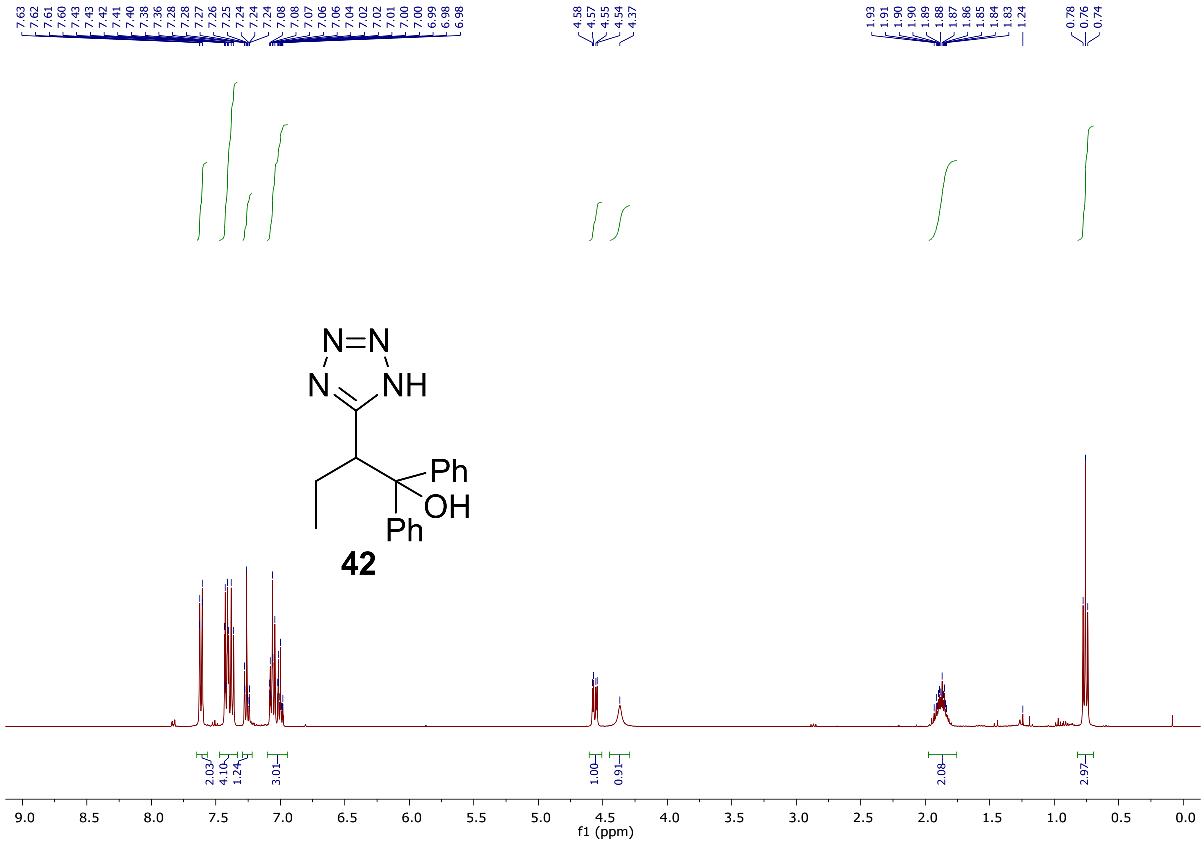
7.63 7.62 7.61 7.60 7.43 7.43 7.42 7.41 7.40 7.38 7.36 7.28 7.28 7.27 7.26 7.25 7.24 7.24 7.24 7.08 7.08 7.07 7.06 7.04 7.02 7.02 7.01 7.00 7.00 6.99 6.98 6.98
4.58 4.57 4.55 4.54 4.37
1.93 1.91 1.90 1.90 1.89 1.88 1.87 1.86 1.85 1.84 1.83 1.24
0.78 0.76 0.74
N=N
N
NH
Ph
OH
Ph
42
2.03 4.10 1.24 3.01 1.00 0.91 2.08 2.97
9.0 8.5 8.0 7.5 7.0 6.5 6.0 5.5 5.0 4.5 4.0 3.5 3.0 2.5 2.0 1.5 1.0 0.5 0.0
f1 (ppm)

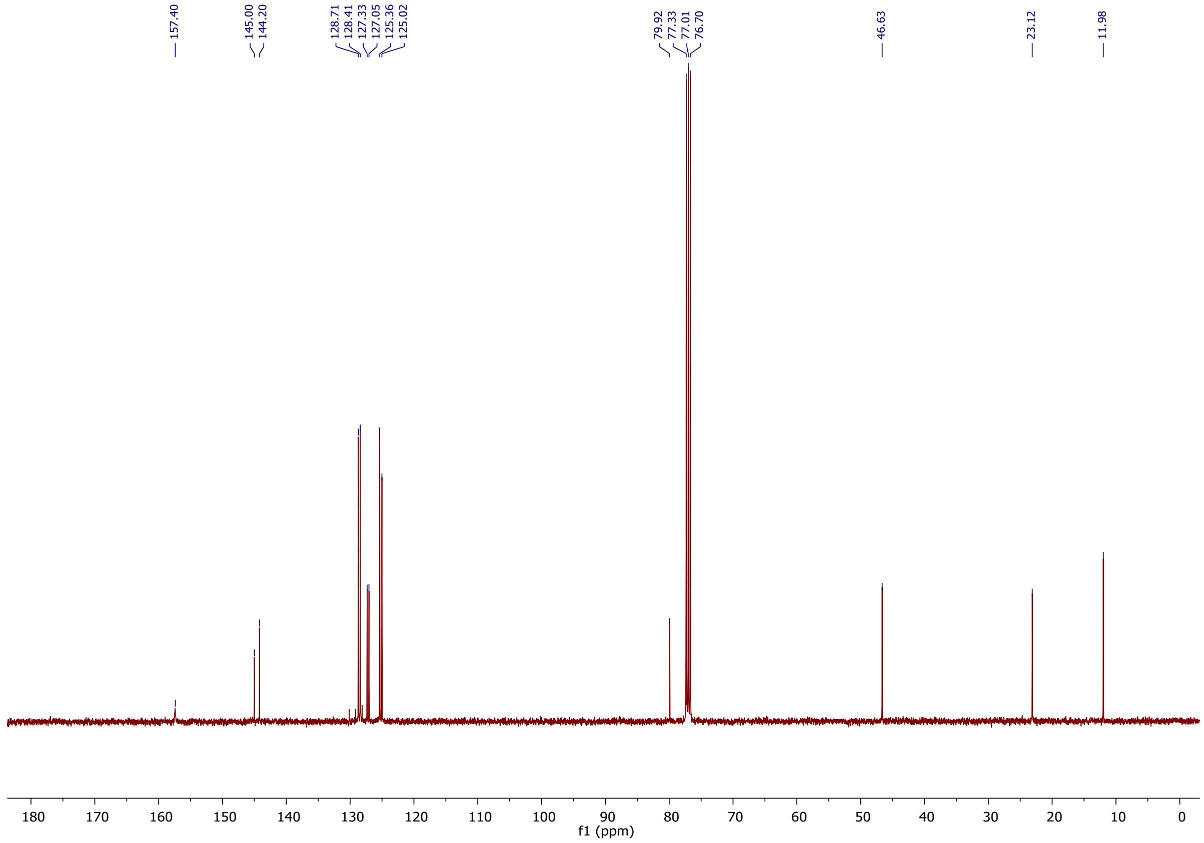
157.40
145.00
144.20
128.71
128.41
127.33
127.05
125.36
125.02
79.92
77.33
77.01
76.70
46.63
23.12
11.98
f1 (ppm)

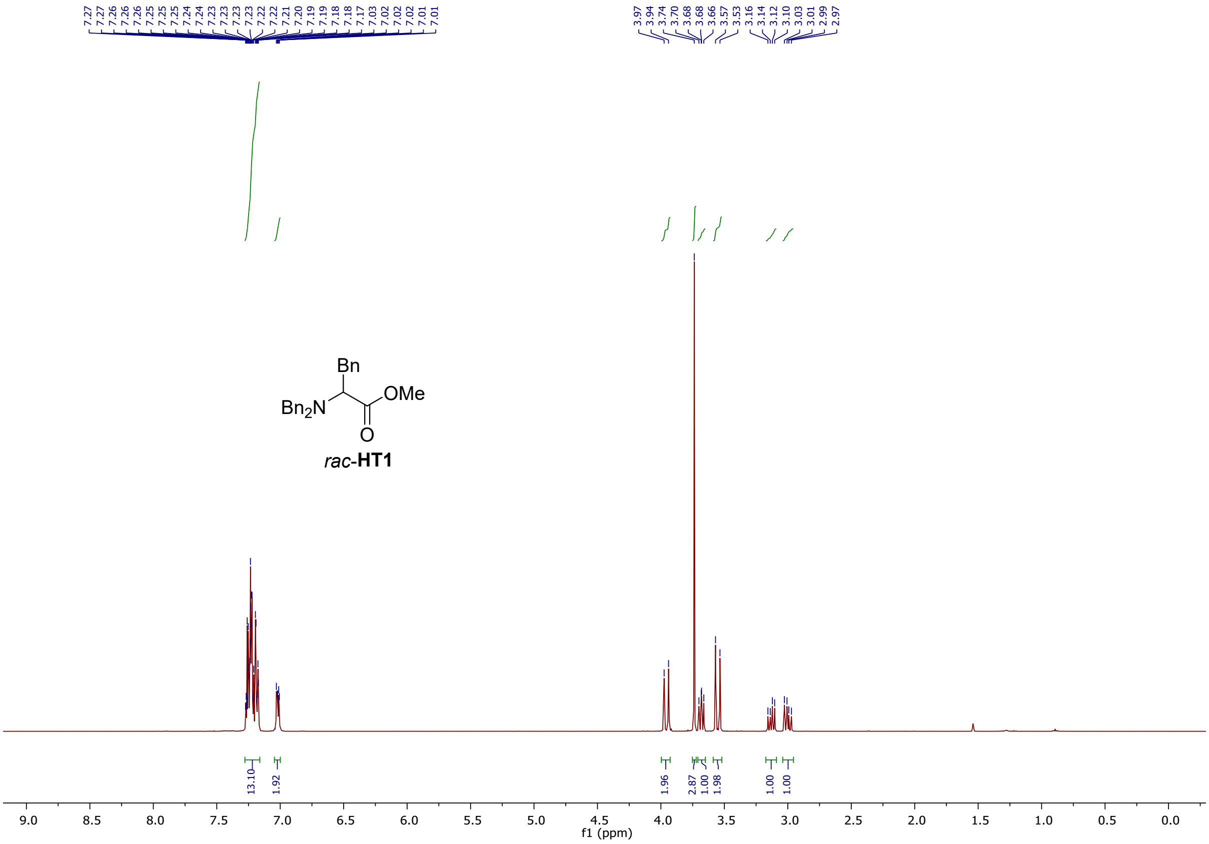
Bn
OMe
Bn2N
O
f1 (ppm)

rac-**HT1**

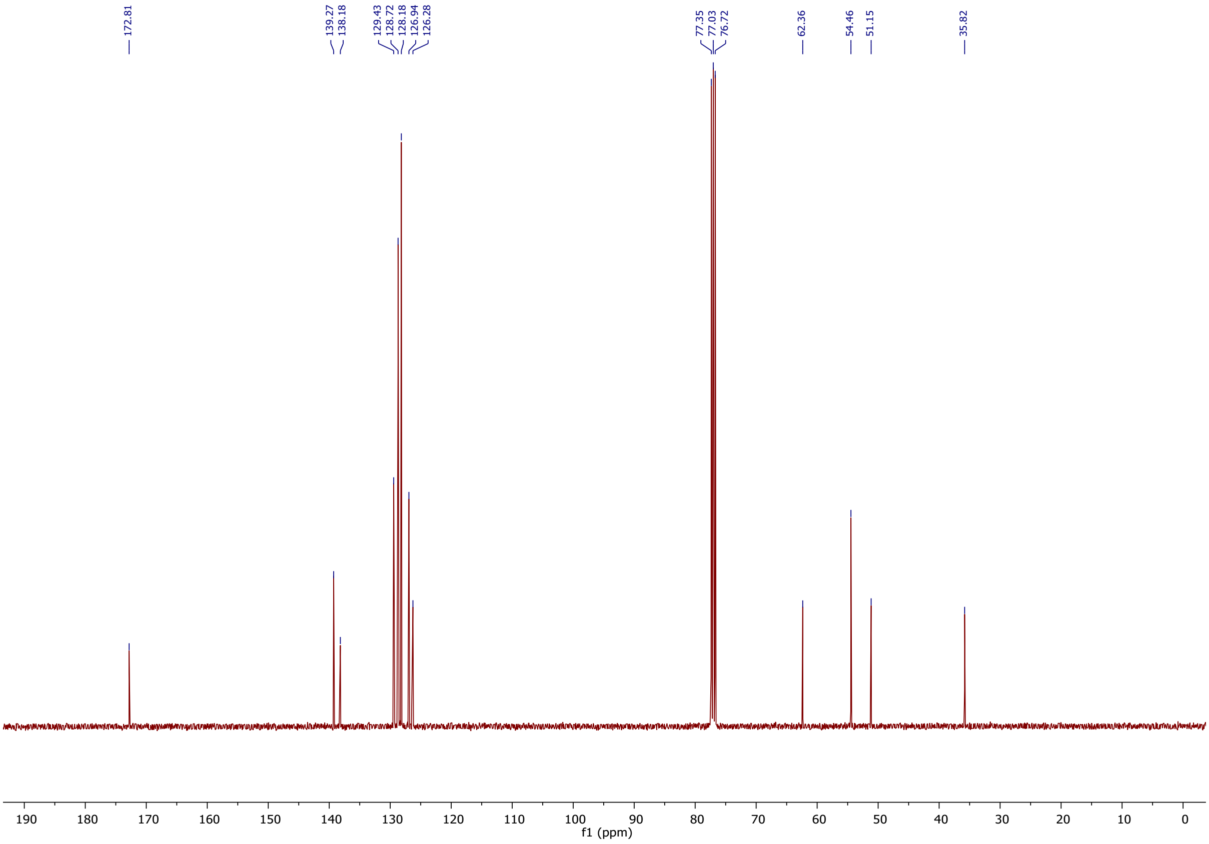
172.81
139.27
138.18
129.43
128.72
128.18
126.94
126.28
77.35
77.03
76.72
62.36
54.46
51.15
35.82
f1 (ppm)

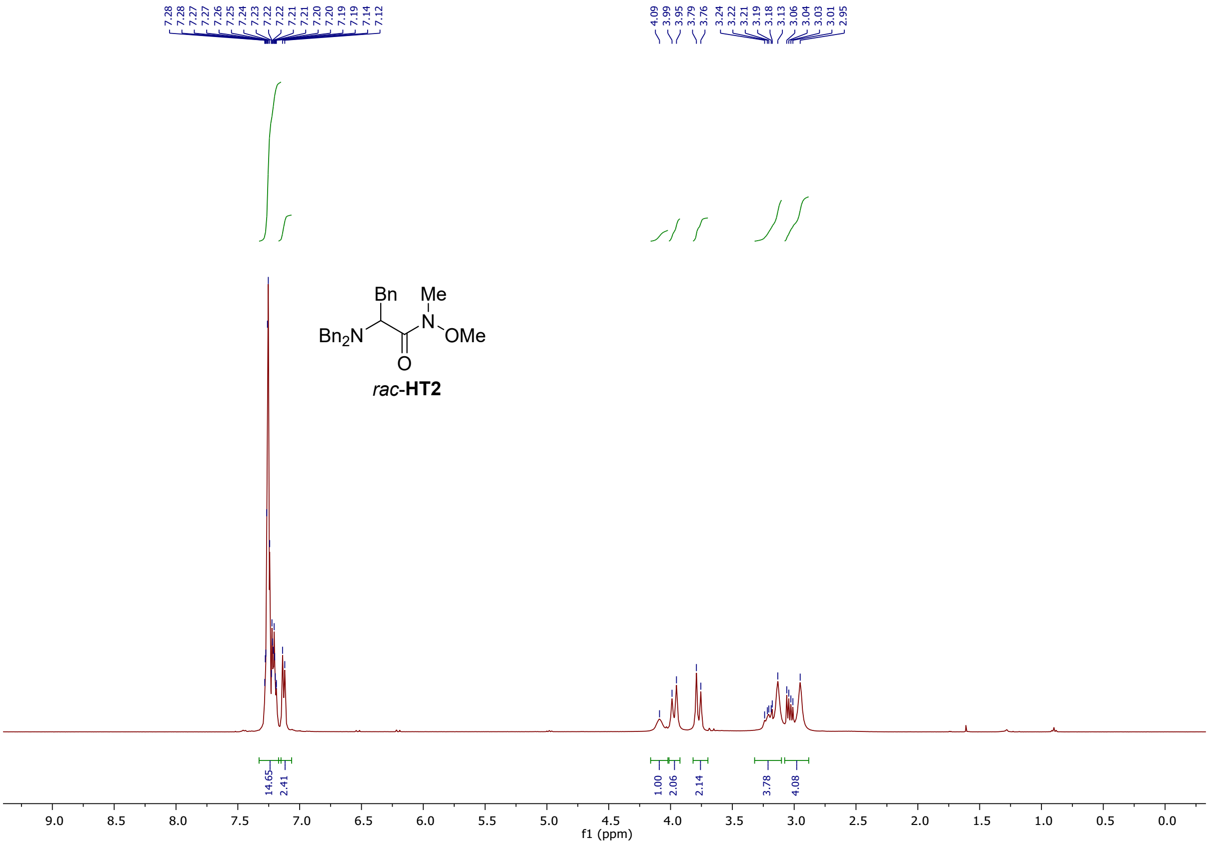
7.28
7.28
7.27
7.26
7.25
7.24
7.23
7.22
7.22
7.21
7.21
7.20
7.20
7.19
7.19
7.14
7.12
4.09
3.99
3.95
3.79
3.76
3.24
3.22
3.21
3.19
3.18
3.13
3.06
3.04
3.03
3.01
2.95
Bn
Me
Bn2N
N
OMe
O
rac-HT2
14.65
2.41
1.00
2.06
2.14
3.78
4.08
f1 (ppm)

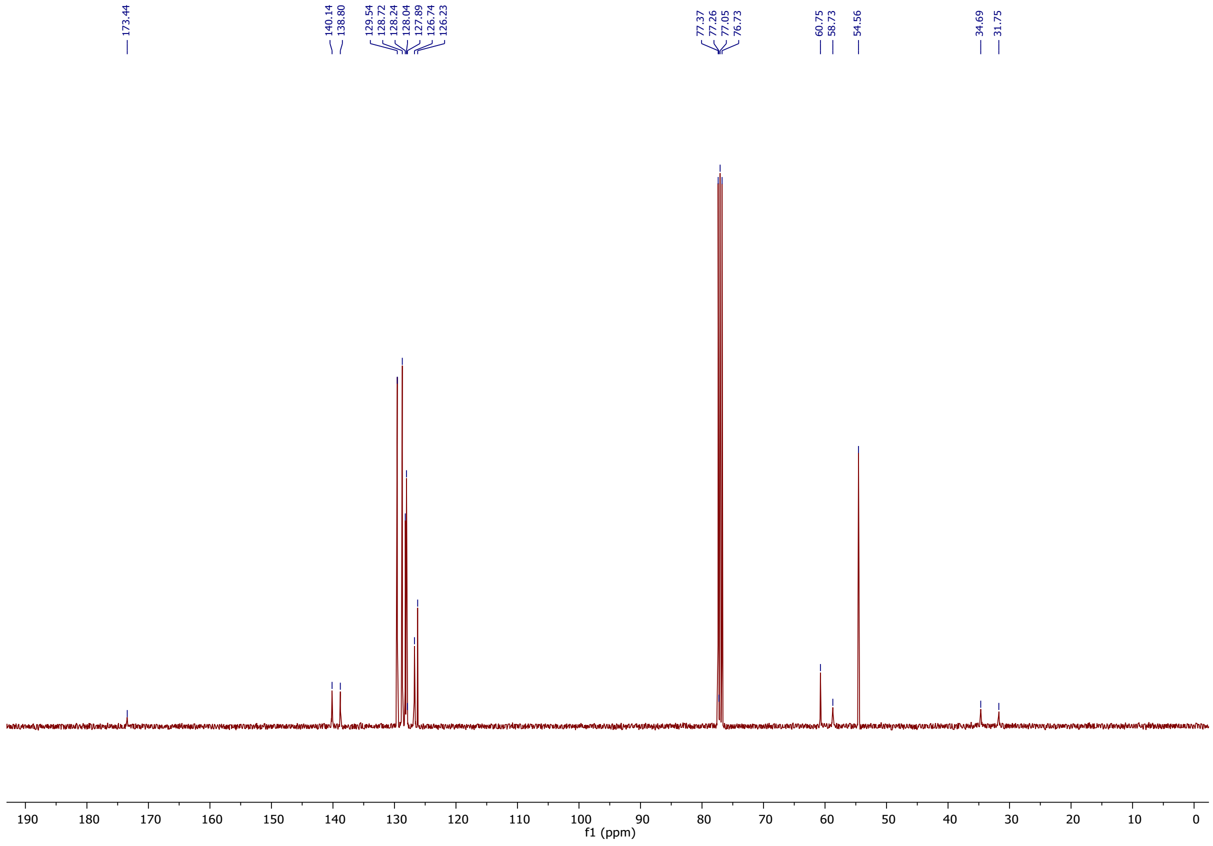
173.44
140.14
138.80
129.54
128.72
128.24
128.04
127.89
126.74
126.23
77.37
77.26
77.05
76.73
60.75
58.73
54.56
34.69
31.75
f1 (ppm)

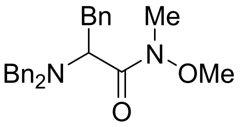

rac-**HT2**

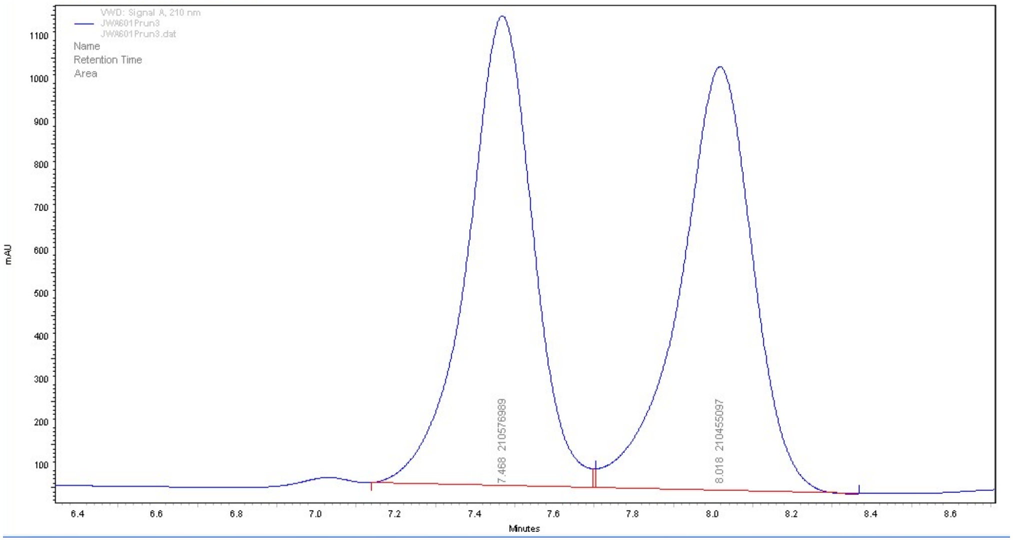
VWD: Signal A, 210 nm
JWA601Prun3
JWA601Prun3.dat
Name
Retention Time
Area
7.468 210576989
8.018 210455097
mAU
Minutes

VWD: Signal A, 210 nm Results

Retention Time	Area	Area %	Height	Height %
7.468	210576989	50.01	18341384	52.58
8.018	210455097	49.99	16544062	47.42
Totals	421032086	100.00	34885446	100.00

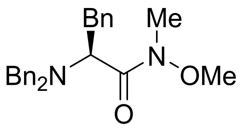

(*S*)-**HT2**

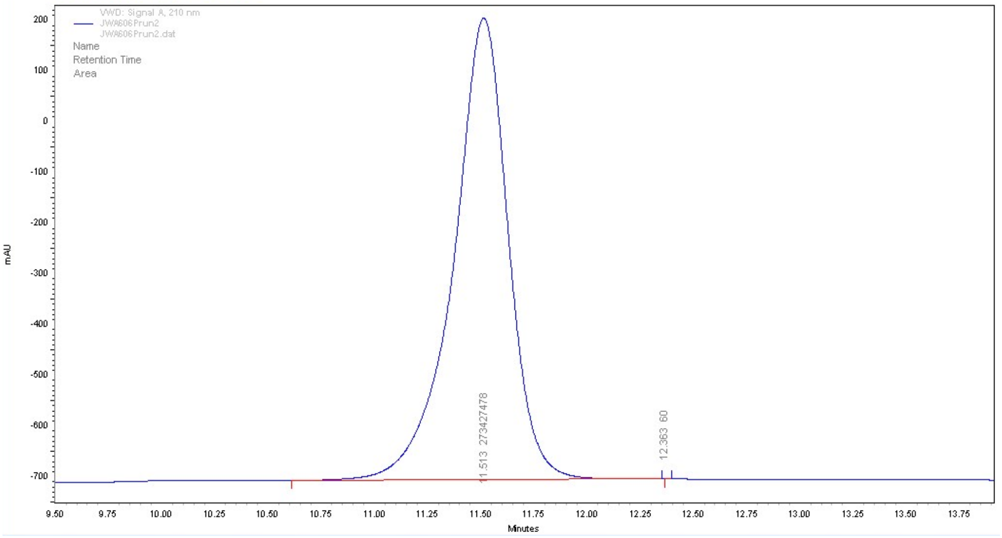

VWD: Signal A,
210 nm Results

Retention Time	Area	Area %	Height	Height %
11.513	273427478	100.00	15267698	100.00
12.363	60	0.00	0	0.00
Totals	273427538	100.00	15267698	100.00

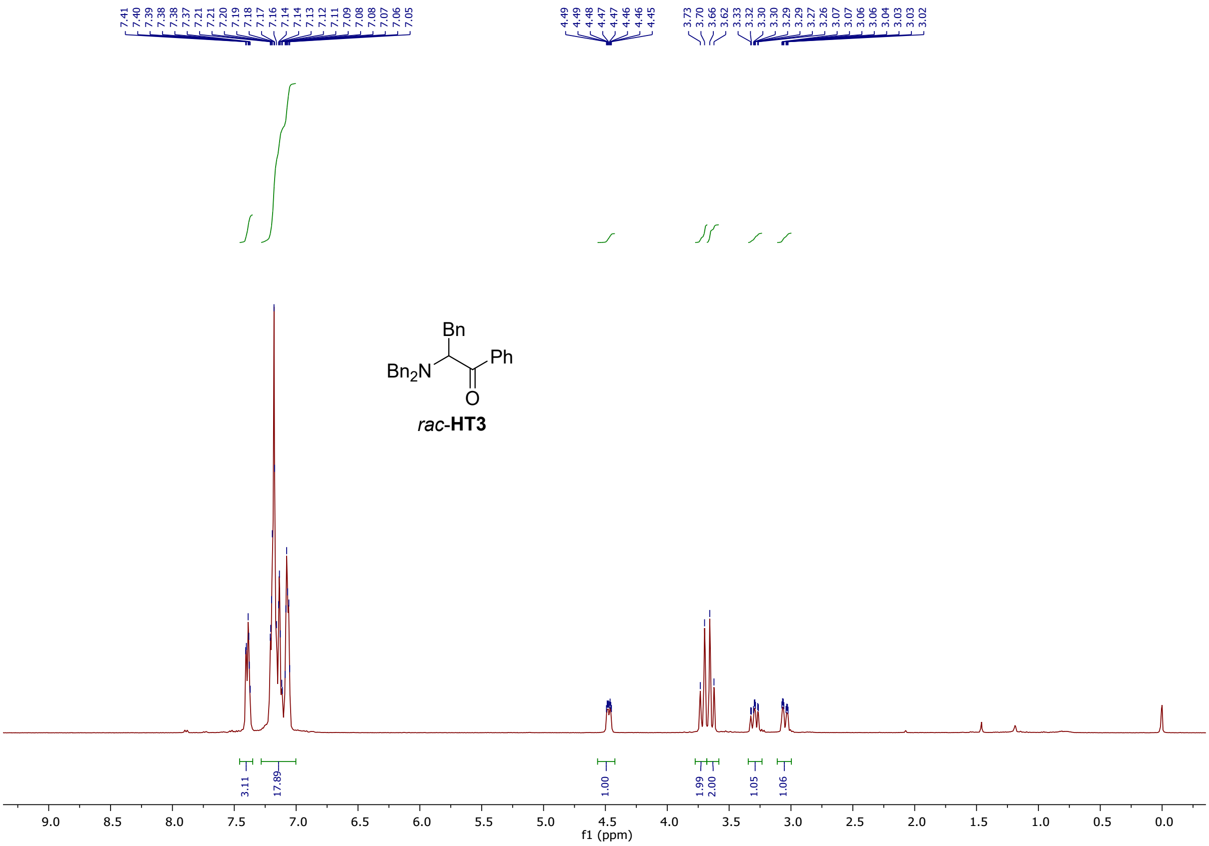

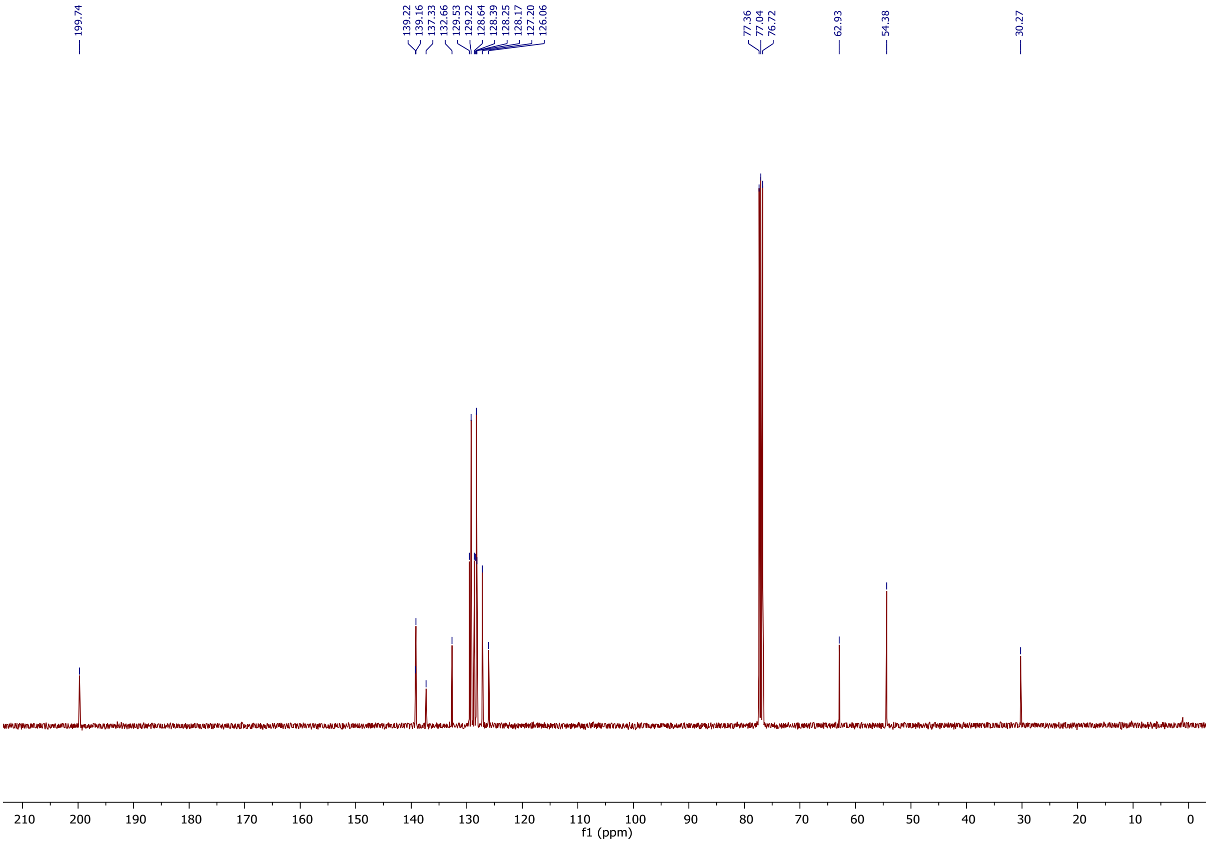
199.74
139.22
139.16
137.33
132.66
129.53
129.22
128.64
128.39
128.25
128.17
127.20
126.06
77.36
77.04
76.72
62.93
54.38
30.27
210 200 190 180 170 160 150 140 130 120 110 100 90 80 70 60 50 40 30 20 10 0
f1 (ppm)

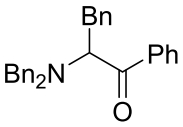
Bn
Bn2N
Ph
O

rac-**HT3**

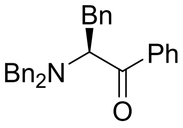
Bn
Ph
Bn_2N
O

(*S*)-**HT3**

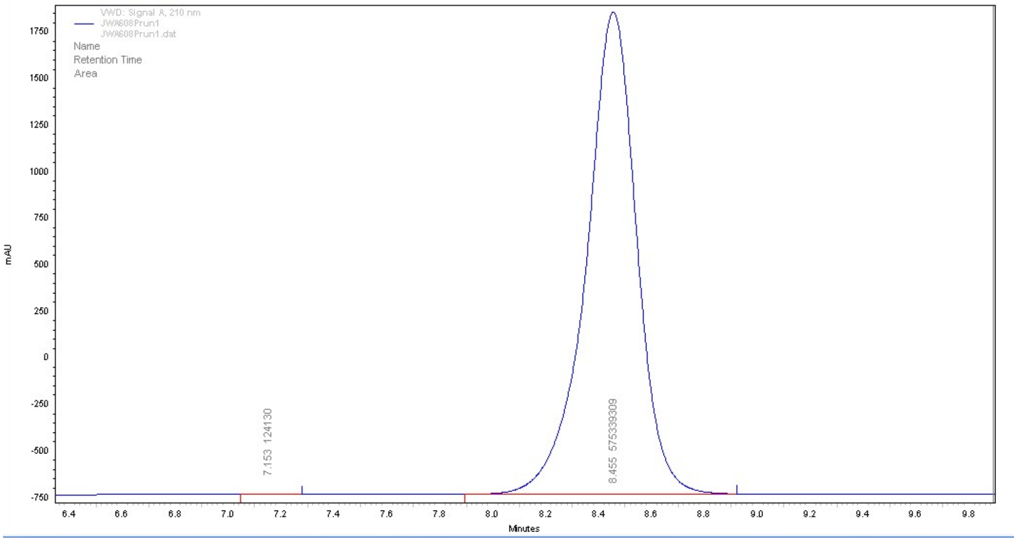
VWD: Signal A, 210 nm
JWA608Prun1
JWA608Prun1.dat
Name
Retention Time
Area
mAU
Minutes
7.153 124130
8.455 575339309

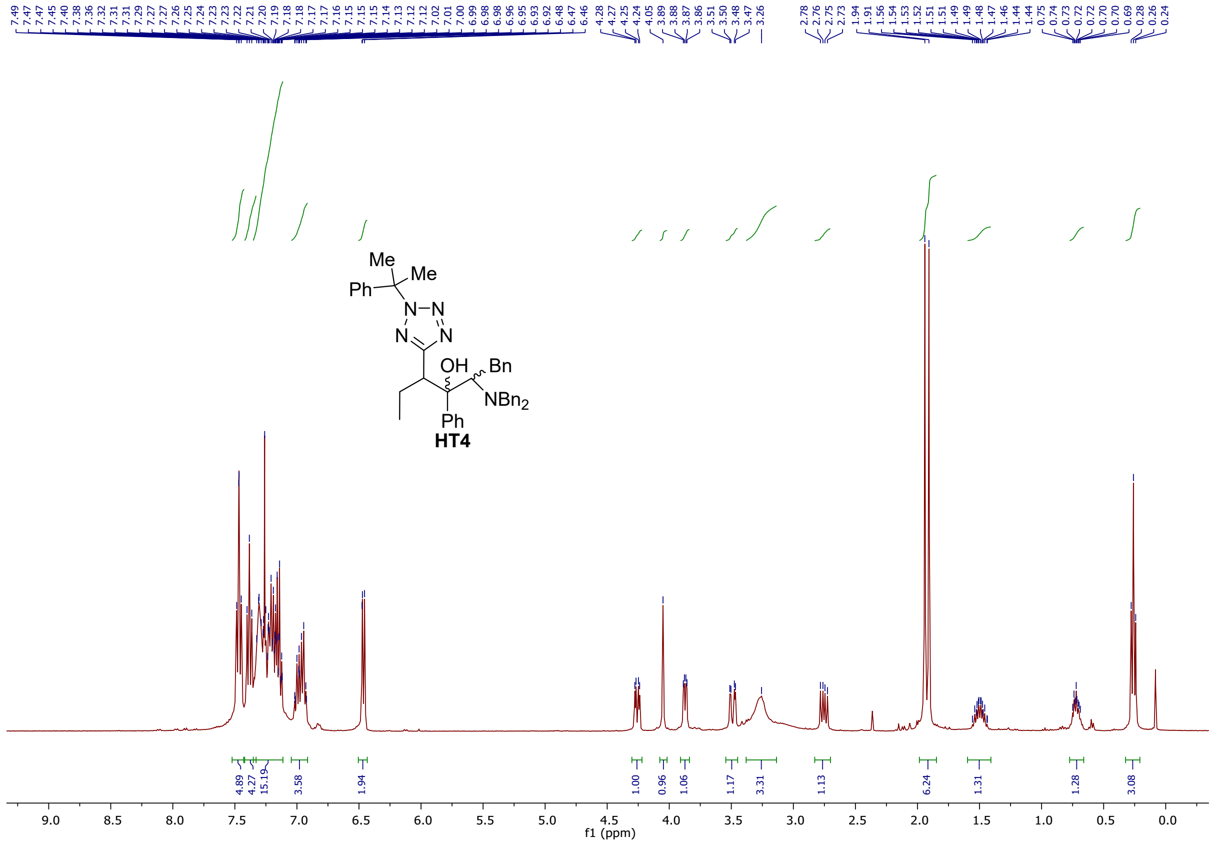
HT4
f1 (ppm)

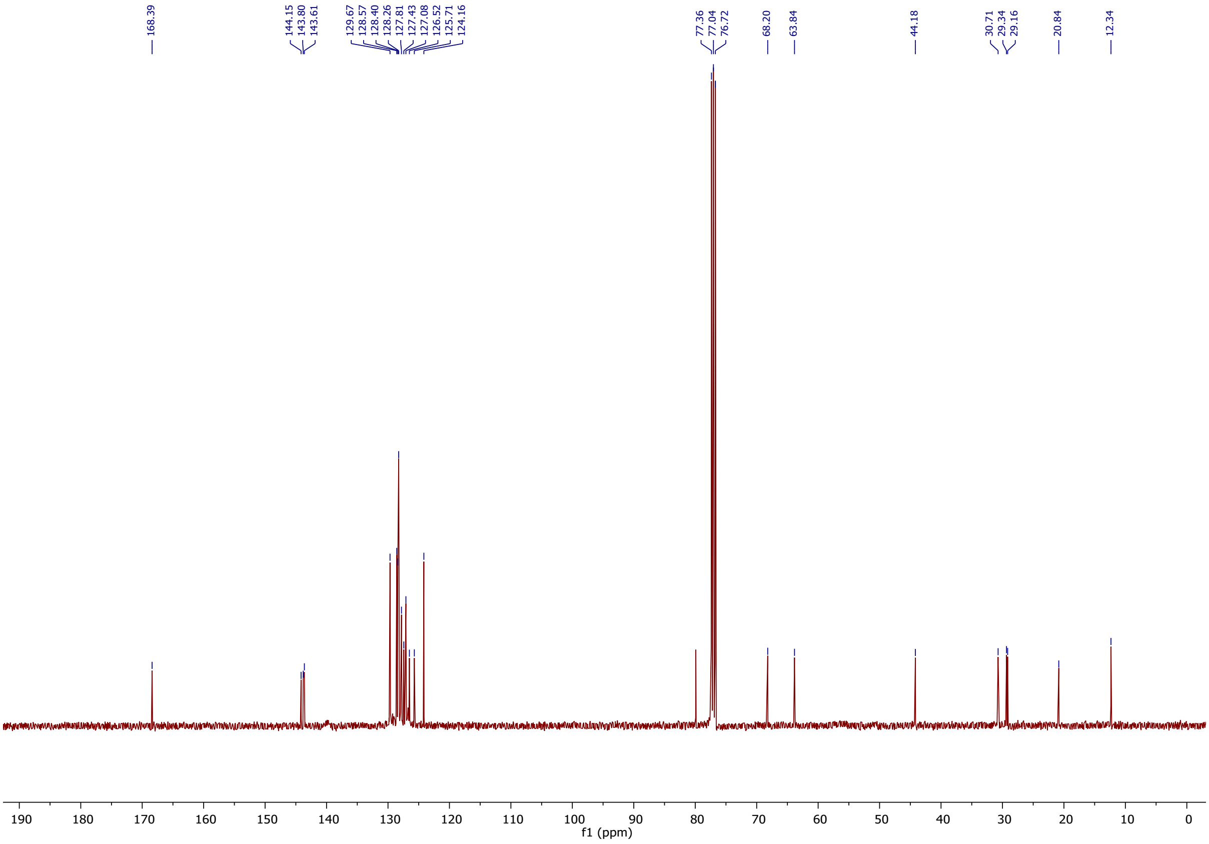
168.39
144.15
143.80
143.61
129.67
128.57
128.40
128.26
127.81
127.43
127.08
126.52
125.71
124.16
77.36
77.04
76.72
68.20
63.84
44.18
30.71
29.34
29.16
20.84
12.34
190 180 170 160 150 140 130 120 110 100 90 80 70 60 50 40 30 20 10 0
f1 (ppm)
